# A Phylogenetic Survey on the Structure of the HIV-1 Leader RNA Domain That Encodes the Splice Donor Signal

**DOI:** 10.3390/v8070200

**Published:** 2016-07-21

**Authors:** Nancy Mueller, Atze T. Das, Ben Berkhout

**Affiliations:** Laboratory of Experimental Virology, Department of Medical Microbiology, Center for Infection and Immunity Amsterdam (CINIMA), Academic Medical Center, University of Amsterdam, 1105 AZ Amsterdam, The Netherlands; n.muller@amc.uva.nl (N.M.); a.t.das@amc.uva.nl (A.T.D.)

**Keywords:** HIV-1 RNA, splicing regulation, repressive RNA structure, alternative RNA conformations, untranslated leader RNA

## Abstract

RNA splicing is a critical step in the human immunodeficiency virus type 1 (HIV-1) replication cycle because it controls the expression of the complex viral proteome. The major 5′ splice site (5′ss) that is positioned in the untranslated leader of the HIV-1 RNA transcript is of particular interest because it is used for the production of the more than 40 differentially spliced subgenomic mRNAs. HIV-1 splicing needs to be balanced tightly to ensure the proper levels of all viral proteins, including the Gag-Pol proteins that are translated from the unspliced RNA. We previously presented evidence that the major 5′ss is regulated by a repressive local RNA structure, the splice donor (SD) hairpin, that masks the 11 nucleotides (nts) of the 5′ss signal for recognition by U1 small nuclear RNA (snRNA) of the spliceosome machinery. A strikingly different multiple-hairpin RNA conformation was recently proposed for this part of the HIV-1 leader RNA. We therefore inspected the sequence of natural HIV-1 isolates in search for support, in the form of base pair (bp) co-variations, for the different RNA conformations.

## 1. Introduction

**Figure 1:**
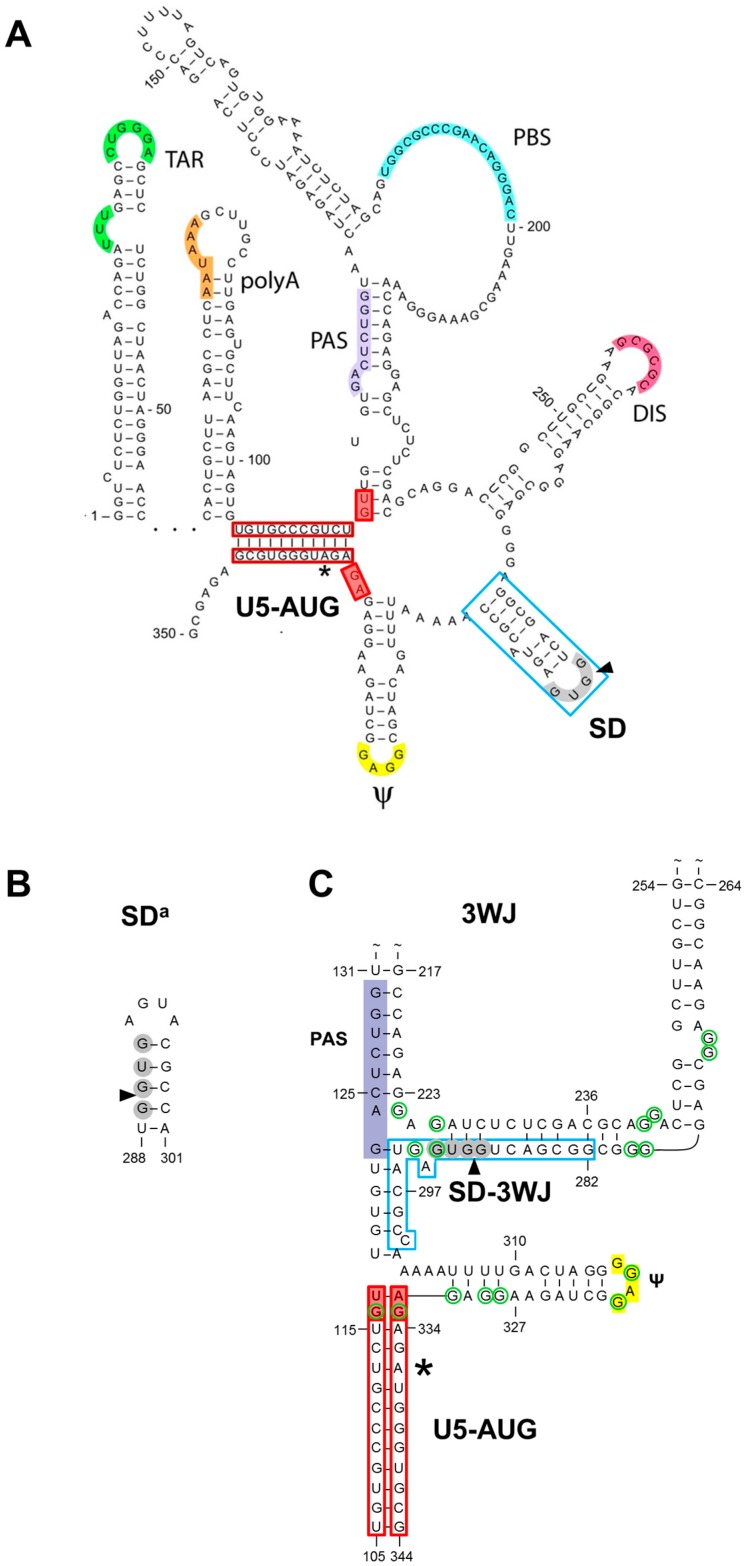
Proposed structures for the splice donor (SD) region in the human immunodeficiency virus type 1 (HIV-1**)** leader RNA. (**A**) The multiple hairpin structure in which the RNA folds the SD hairpin (▲, 5′ splice site cleavage site) and several other stem-loop structures that encode important replication signals (TAR, transacting responsive element; polyA, polyadenylation signal; PAS, primer activation signal; PBS, primer binding site; DIS, dimerization initiation signal; ψ, packaging signal); (**B**) The alternative SD^a^ hairpin structure. (**C**) The 3WJ structure in which the SD region interacts with upstream nucleotides (nts; SD-3WJ interaction); (**A**–**C**) The HIV-1 LAI prototype sequence is shown with the nts that form the U5-AUG long-distance interaction boxed in red (*, AUG start codon) and the nts that form the 2-bp U5-AUG extension in the 3WJ structure indicated with red filled boxes. In (**C**), the 17 unpaired or weakly-paired guanosines that have been suggested to serve as potential nucleocapsid binding sites are encircled in green.

The human immunodeficiency virus type 1 (HIV-1) untranslated leader RNA region can fold several stem-loop structures that have important regulatory functions, including the transacting responsive element (TAR), polyadenylation signal (polyA), dimerization initiation signal (DIS), splice donor (SD) and psi hairpins (Figure 1A) [1,2,3]. We here focus on the SD hairpin (nucleotides 282–300; boxed in blue in Figure 1A), which has been proposed to play a regulatory role in HIV-1 RNA splicing. However, two recent reports suggested a slightly different multiple-hairpin conformation of the leader RNA in which the SD region folds differently [4,5]. We will therefore present what is known about this SD hairpin and the alternative conformations, followed by a detailed phylogenetic analysis.

### 1.1. The SD Hairpin 

The presence of the SD hairpin was first suggested in 1993 based on the unusual migration of a 44 nucleotide (nt)-leader RNA molecule encoding the SD and psi sequences during native gel electrophoresis [6]. The SD hairpin structure was subsequently confirmed in multiple studies that used a variety of techniques, including Mfold RNA structure prediction, chemical probing and mutational analysis [7,8,9]. In addition, a base pair (bp) co-variation was described in the lower stem region (A^282^–U^300^ instead of G^282^–C^300^) of diverse HIV-1 isolates, thus providing some initial phylogenetic support for the proposed base pairing scheme [8]. An initial nuclear magnetic resonance (NMR) structure analysis of a short leader RNA fragment (nt 282–300) also supported the SD hairpin, but bp A^286^–U^295^ was suggested to form a novel base-triple platform with the bulged nt A^296^ [10]. SHAPE (selective 2′-hydroxyl acylation analyzed by primer extension)-based RNA structure probing of the complete HIV-1 RNA genome confirmed SD hairpin folding [11]. 

The analysis of mutant and revertant viruses provides an additional means to score the importance of specific RNA structures. For instance, we demonstrated that opening of the TAR hairpin is detrimental to HIV-1 replication. Revertant viruses that were selected upon long-term culturing of the TAR-mutated virus repaired this structure—not necessarily the sequence—by fixation of spontaneous mutations [12]. This forced evolution approach mimics the search for bp co-variations in a phylogenetic survey to identify biologically relevant RNA structures [13]. HIV-1 mutation studies indicated that the thermodynamic stability of the SD stem-loop structure is important for virus replication [14,15,16,17]. Stabilization of the hairpin caused a severe virus replication defect and decreased the splicing efficiency. Upon long-term culturing of these mutants, revertant viruses could be selected that acquired additional mutations that opened the stabilized hairpin, thus demonstrating that a too stable SD hairpin hampers HIV-1 replication [14]. More recently, we assessed whether splicing of HIV-1 RNA is suppressed by the wild-type (wt) SD structure. To do so, mutants with a destabilized structure were analyzed for the impact on virus replication and splicing efficiency. We observed that destabilization of the wt hairpin does increase the level of HIV-1 splicing [15,16,17] , which indicates that HIV-1 RNA splicing is indeed at least partially suppressed by the SD stem-loop structure that encompasses the 5′ splice site (5′ss). Like stabilization, destabilization of the SD hairpin reduced virus replication, suggesting that HIV-1 requires a sub-optimal level of splicing in order to express all viral transcripts and proteins in the amount required for optimal virus replication. There are other reports of splicing regulation by RNA structure [18,19,20,21,22,23,24,25,26,27,28,29]. In addition to local RNA structure, several sequence elements—especially binding sites for SR proteins and U1 small nuclear RNA (snRNA)—are known to regulate HIV-1 splicing [16,17,30]. 

### 1.2. The Alternative SD^a^ Hairpin

Although the original SHAPE-probing study supported the SD hairpin [11], a more recent SHAPE analysis by the same group of Kevin Weeks suggested an alternative hairpin structure [5]. This SD^a^ hairpin (Figure 1B) presents the 5′ss cleavage site in the base-paired stem and not in the single-stranded loop as in the prototype SD hairpin. In fact, the SD^a^ hairpin was proposed in early phylogenetic studies and also supported by chemical probing and RNA structure prediction software [31,32]. However, the SD hairpin was subsequently believed to represent the more likely RNA fold [7]. Interestingly, we also observed this alternative SD^a^ structure in SHAPE-probing experiments, but only for mutants in which the SD hairpin was destabilized [15]. Mfold analysis confirmed SD^a^ folding for these destabilized mutants, unlike the wt sequence that adopts the SD hairpin. 

### 1.3. The 3WJ Structure

A recent NMR study on a larger fragment of the HIV-1 leader RNA (nt 105–345) of the NL4-3 strain by the Michael Summers team suggested a rather different folding of the SD RNA region [4]. A tandem three-way junction (3WJ) structure was proposed for this RNA fragment (Figure 1C), in which the SD nucleotides (nts) interact with upstream leader sequences (interaction indicated with SD-3WJ interaction), in particular with nts that form the 5′ and 3′ sides of the primer binding site (PBS) stem in the earlier-proposed multiple-hairpin conformation (Figure 1A) [33]. The 3WJ structure contains the U5-AUG duplex that was previously described for the multiple-hairpin structure (boxed in red in Figure 1A,C), but this interaction is now extended with two base pairs (bps) compared to the original structure (filled red boxes). The original U5-AUG interaction is supported by HIV-1 mutational studies, phylogenetic (co-variation) analyses and biochemical assays (chemical probing and gel electrophoresis) [33,34]. The initial report [33] also demonstrated conservation of this U5-AUG interaction in different HIV-1, HIV-2 and simian immunodeficiency virus (SIV) isolates despite significant sequence variation. A role in translation regulation was previously proposed for the U5-AUG interaction as it masks the Gag start codon, but a subsequent mutational survey failed to provide support for such a mechanism [35].

The new NMR study proposes that the 3WJ structure is intrinsically involved in the regulation of different leader RNA processes, such as mRNA translation and packaging of the dimeric RNA genome in virions, but only the latter was tested experimentally. Analysis of NC protein binding and RNA packaging for wt and mutant RNAs suggested the importance of unpaired and weakly-paired “junction” guanosines that are exposed by the 3WJ scaffold (encircled in green in Figure 1C). The suggested translation regulatory function remains to be tested. 

### 1.4. In Search for Phylogenetic Support for SD, SD^a^ or 3WJ 

The recent controversy about the actual folding of the SD domain triggered us to perform an in depth phylogenetic analysis. This unbiased method was key for solving the structure of ribosomal RNA and many other complex RNA molecules [36,37,38]. Such an in silico analysis seems particularly valuable because different experimental HIV-1 RNA probing methods seem to yield different answers. A brief phylogenetic survey was performed in the recent NMR study by Keane et al. [4] and it was concluded that “the tandem three-way junction structure is highly conserved, and the rare variations that disrupt base pairing are due to transient polymorphisms”. We set out to perform a more detailed sequence variation analysis for the bps that constitute the SD, SD^a^ and 3WJ structures. 

## 2. Materials and Methods

The sequence of the nts involved in the proposed SD, SDa and 3WJ structures of all HIV-1 group M isolates described in the Los Alamos HIV sequence database (www.hiv.lanl.gov; Premade web alignment of complete sequences 2012; Figure S1) was compared with the corresponding sequence of the prototype subtype B LAI strain to identify nucleotide variations. Sequences with ambiguous nts, nt insertions and deletions, and nt variations that were observed only in a single virus isolate were excluded, as these may represent reverse transcription, PCR or sequencing errors.

## 3. Results

### 3.1. SD Phylogeny

**Figure 2:**
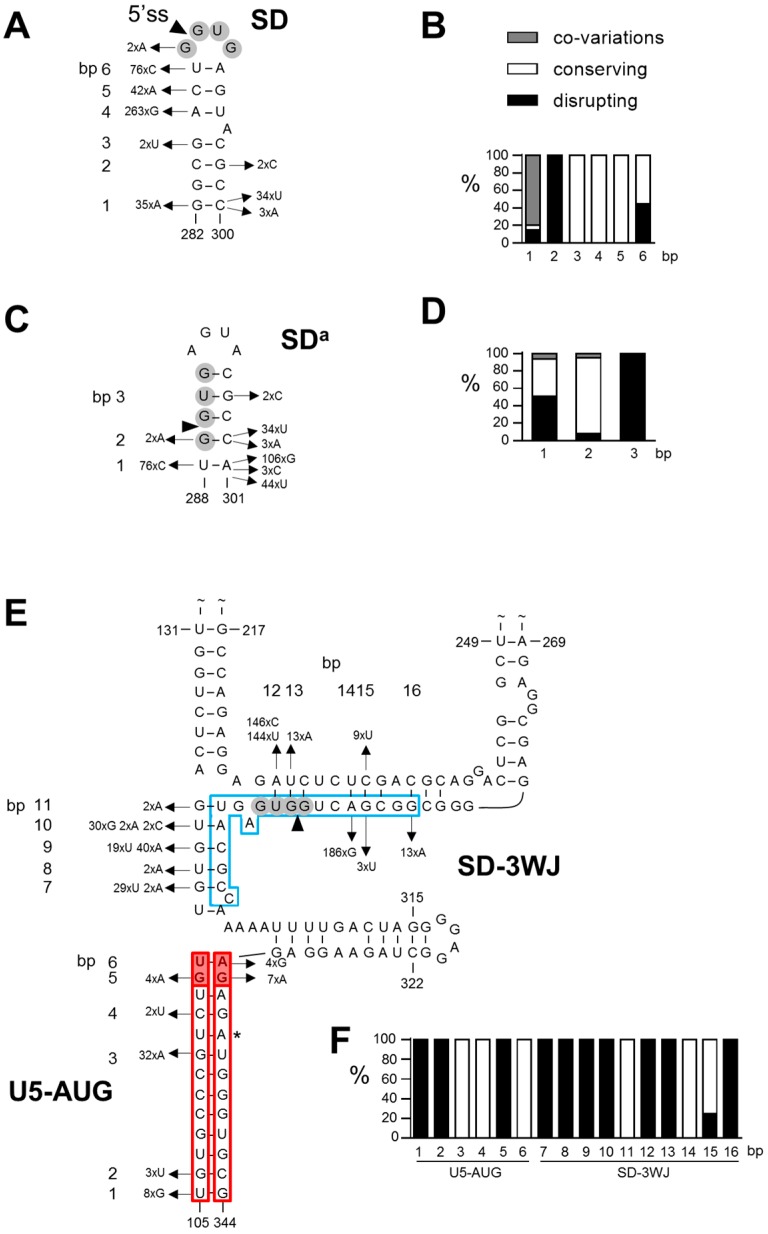
Phylogenetic analysis of the SD, SD^a^ and 3WJ structures. The sequence of the SD hairpin (**A,B**), SD^a^ hairpin (**C**,**D**) and 3WJ structure (U5-AUG and SD-3WJ interactions; **E**,**F**) in HIV-1 isolates described in the 2012 web alignment of the Los Alamos HIV-1 sequence database (Figure S1) were compared to the corresponding sequence in the HIV-1 LAI strain. Sequences with ambiguous nts, nt insertions and deletions and nt variations that were observed in a single virus isolate were excluded from the analysis. (**A**,**C**,**E**) Variations observed in base-paired nts (numbered bps) and their frequency in 1128 SD, 1135 SD^a^, 708 3WJ-SD and 462 U5-AUG sequences are shown. (**B**,**D**,**F**) The nt variations were scored as co-variation (grey bar), as structure conserving (all other variations that do allow Watson-Crick bp formation in the same or similar structure; white bar) and structure disrupting variations (not allowing bp formation in the same or similar structure; black bar). Co-variations were counted as single events.

**Figure 3:**
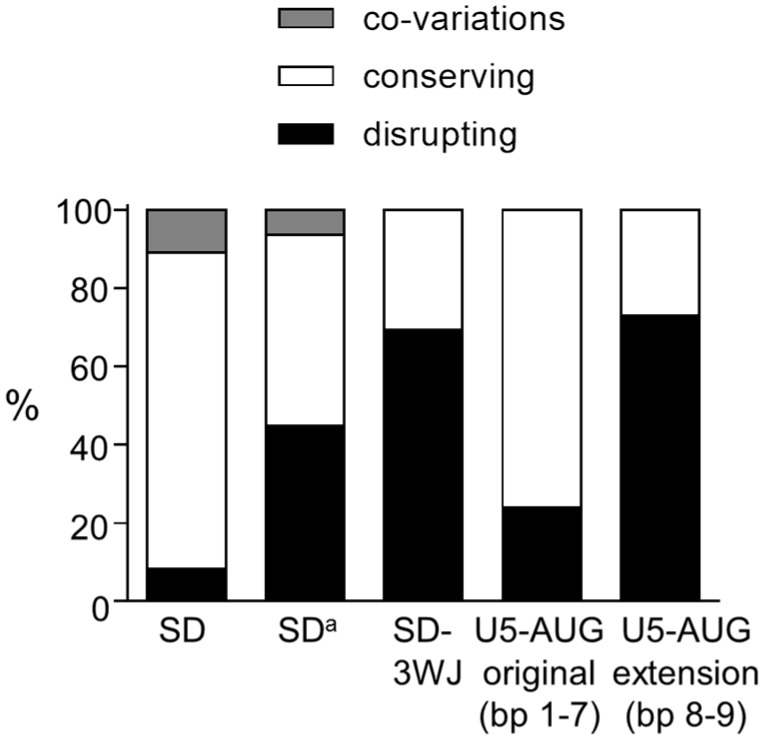
Phylogenetic analysis supports the SD hairpin structure. All nt variations observed in the base paired nts of the SD and SD^a^ hairpins, and the SD-3WJ interaction, U5-AUG duplex (bp 1–4) and U5-AUG duplex extension (bp 5–6) of the 3WJ structure were scored as co-variation (grey bar), as structure conserving (white bar) or as structure disrupting (black bar). Co-variations and double variations were counted as single events. The total frequency of each category is shown.

Comparison of the sequence of 1128 HIV-1 group M isolates with the prototype subtype B LAI strain resulted in the identification of 459 variations in the SD region (nt 282–300; Figure 2A). A large fraction of the variations affect base-paired nts (indicated with bp 1–6 in Figure 2A). In 32 isolates, a bp co-variation is observed at bp 1 (A-U instead of G-C bp) that strongly supports this interaction. The majority of the other variations also allow formation of the same bps. For example, the A286G change that is observed in 263 isolates causes a G-U bp instead of an A-U at bp 4. Other variations that open bps and that at first glance do not support the SD hairpin, result in a small realignment in the stem region and in the formation of a comparable SD hairpin structure. For example, the frequently observed double variation C287A/U288C opens two bps (bp 5 and 6: C^287^–G^294^ and U^288^–A^293^) but results in the formation of two alternative bps (A^287^–U^295^ and C^288^–G^294^). Some virus isolates hold multiple nt differences in this leader RNA domain, but as previously shown [15,17] most isolates can fold a similar SD hairpin structure. Only a minority of the variations does not allow Watson-Crick bp formation. For each bp at which nt variation was observed, we calculated the percentage of co-variations, structure conserving variations (all other variations that do allow bp formation in a SD hairpin structure) and structure disrupting variations (not allowing bp formation in a SD hairpin structure) (Figure 2B), which revealed that in particular the variation observed at bp 1, 3, 4 and 5 is compatible with the SD hairpin structure. Taken together, we observed 8% co-variations, 81% structure conserving variations, and only 11% disrupting variations in the SD region (Figure 3), which gives phylogenetic support for the existence of the SD hairpin. 

### 3.2. SD^a^ Phylogeny 

In the RNA region that folds the alternative SD^a^ hairpin (nt 288–301), 270 nt variations were observed in 1135 isolates that affect one of the bps in the stem (bp 1–3 in Figure 2C,D). At bp 1, a co-variation is observed in 14 isolates (U-A to G-C), which represents ~7% of the observed variation at this position (Figure 2D). Approximately 43% of the variation at this position is structure conserving and 51% is structure disrupting (Figure 2D). Co-variation is also observed in 2 isolates at bp 2 (G-C to A-U), while most of the sequence variation at this position is structure conserving. At bp 3, only structure disrupting variation is observed. In total, 6% of the variations in the SD^a^ region represent bp co-variations and 49% are structure conserving (Figure 3). A large fraction (45%) of the variations disrupts the SD^a^ structure, while this level was much smaller for the SD hairpin structure (only 8%; Figure 3). 

### 3.3. 3WJ Phylogeny 

We similarly analyzed the interaction between the SD sequence and the upstream nts (SD-3WJ domain) and the extended U5-AUG interaction in the 3WJ structure. Analysis of the SD-3WJ nts yielded 642 variations in 708 isolates that affected 10 of the 12 putative bps in this region (bp 7–16 in Figure 2E,F). No bp co-variations were observed. For 7 of the 12 bps, all observed variation is structure disrupting. Only at bp 11 and 14, all sequence variation is structure conserving. However, the frequently observed A286G change that is responsible for the variation at bp 14 also supports the SD hairpin structure (Figure 2A, bp 4). In total, 69% of the SD-3WJ variations are structure disrupting and only 31% are structure conserving (Figure 3).

We observed 59 variations in 462 extended U5-AUG sequences that affected only 6 of the 13 putative bps (bp 1–6 in Figure 2E,F). Also in this domain no co-variations were observed. The variation observed at bp 3 and 4 in the original duplex and at bp 6 in the U5-AUG extension is structure conserving. Only disrupting variation is seen at bp 1 and 2 in the original duplex and at bp 5 in the extended structure. In total, only 24% of the variation in the original U5-AUG duplex (bp 1–4) is structure disrupting, whereas this percentage is 73% for the duplex extension (bp 5–6) (Figure 3). Taken together, our phylogenetic analysis does not support the interaction between the SD nts and upstream sequences (SD-3WJ) or the U5-AUG duplex extension (bp 8–9) that are characteristic for the 3WJ structure [33]. 

## 4. Discussion

This broad phylogenetic analysis of the three proposed structures for the SD region indicates that the observed sequence variation is highly compatible with the SD hairpin structure, less with the SD^a^ structure and only poorly with the 3WJ folding (Figure 3). In particular, no bp co-variations were observed in the proposed SD-upstream nts interaction in the 3WJ structure. Possibly, such sequence variation may occur less frequently in tertiary RNA structure motifs like 3WJ than in relatively simple hairpin structures. However, bp co-variations have been described for other tertiary RNA folds like pseudoknots [39,40,41,42]. In fact, the proposed 3WJ structure is supported exclusively by NMR data [4]. Even though it was mentioned by Keane et al. [4] that the new 3WJ structure is consistent with available SHAPE structure probing data, this provides only non-exclusive support as the same data were previously used to support a different RNA structure [43]. Although the NMR study represents a tour de force concerning the size of the RNA fragment studied (155 nts), a caveat of the study is the use of multiple truncations (deletion of the TAR and polyA hairpins and the apical PBS domain). This mono-disciplinary data set seems too restricted to recall existing RNA structure models for the HIV-1 leader RNA.

Some of the confusion about the folding of this leader RNA domain may come from the fact that all of the proposed foldings are semi-stable. For example, MFold RNA structure analysis predicts a thermodynamic stability of −7.1 and −3.6 kcal/mol for the SD and SD^a^ hairpins, respectively. These hairpins are much less stable than some of the other leader RNA hairpins, like TAR (−24.8 kcal/mol) and polyA (−15.3 kcal/mol). We cannot formally disproof the SD^a^ and 3WJ structures and it remains of interest to test their candidate functions, possibly as part of a riboswitch, as multiple structures may co-exist or distinct structures may exist under different conditions. Such a riboswitch has been proposed for other leader RNA domains [1,44,45]. Distinct structures may exist under different conditions to play a specific role in one of the many leader RNA functions in the HIV-1 replication cycle. Accordingly, different SD domain structures may be adopted at different stages of the HIV-1 replication cycle, e.g. as nuclear versus cytoplasmic RNA or upon packaging of the viral RNA genome in virion particles, possibly induced by coating with the viral nucleocapsid (NC) protein. Functional tests by means of a mutational analysis are seriously hampered by the exquisite sequence and structural requirements of these leader RNA domains [46].

Although the SD sequence analysis was informative, we previously obtained much stronger phylogenetic support for other HIV-1 RNA structures. For instance, we documented multiple co-variations and even clustered co-variations in a single duplex for other leader domains like the TAR and polyA hairpins, thus providing very strong phylogenetic support for the existence of these structures [47,48,49,50,51,52]. We did not see such prominent co-variations in the SD region of the HIV-1 genome. This may indicate that the SD hairpin is less important for virus replication than the TAR and polyA hairpins. This may also indicate a stronger sequence requirement in SD than TAR. Mutation-reversion analysis of the lower stem of the TAR hairpin indeed indicated that maintenance of the structure is more critical than the sequence requirement [12]. Conservation of the SD sequence is likely related to the presence of the U1 snRNA and SR protein binding sites [16,17,30,53]. These sequence requirements may have prevented the evolution of more informative sequence variation. 

In summary, the SD hairpin structure is not only supported by the results of different experimental analyses, including virus mutant/revertant analysis, RNA structure probing, gel electrophoresis and NMR analysis [6,7,8,9,10,11], but this structure is also best in line with the phylogenetic survey presented here. Our recent functional studies demonstrated that the SD hairpin has a regulatory function in the control of HIV-1 splicing and that both the structure and sequence of the hairpin are critical for this function.

## Supplementary Materials

The following are available online at www.mdpi.com/1999-4915/8/7/200/s1, Figure S1. Alignment of all HIV-1 group M sequences described in the Los Alamos HIV sequence database (Premade web alignment of complete sequences 2012; www.hiv.lanl.gov) and used in the phylogenetic analysis.
