# Supplementary material for: A Phylogenetic Survey on the Structure of the HIV-1 Leader RNA Domain That Encodes the Splice Donor Signal
Source: Viruses. 2016 Jul 21;8(7):200. doi: 10.3390/v8070200 (PMC4974535; doi:10.3390/v8070200)
Supplement: Supplementary file 1 [file viruses-08-00200-s001.pdf]

Supplementary Materials: A Phylogenetic Survey on the Structure of the HIV-1 Leader RNA Domain That Encodes the Splice Donor Signal

Nancy Mueller, Atze T. Das and Ben Berkhout

```
                                       ----U5/AUG----
                                                     SD/3WJ-
                                  ....|....| ....|....| ....|....| ....|....| ....|....| ....|....| ....|....| ....|....| ....|....| ....|....|
                                      5          15         25         35         45         55         65         75         85         95
B.FR.83.HXB2_LAI_IIIB_BRU.K034    TAGTGTGTGC CCGTCTGTTG TGTGA----- ---------C TCTGGTAA-C TAGAGATCCC TCAGACCCTT --TTAGTCAG T-GTGGAA-- AATCTCTAGC
A1.CD.02.02CD_KTB035.AM000055     TAGTGTGTGC CCTTCTGTTG TGTGC----- ---------C TTTGGTAA-C TAAGGATCCC TCAGCCCCCT --TTAAAAGA A-GTGAA--- AATCTCTACC
A1.CD.97.97CD_KCC2.AM000053       TGGTGTGTGC CCGTCTGTTG TGTGA----- ---------C TCTGGTAA-C TAGAGATCCC TCAGACCACC ---TTAGCAG A-GTGAAA-- AATCTCTAGC
A1.ES.05.X1608_8.FJ670519         TAGTGTGTGC CCGTCTGTTG TATGA----- ---------C TCTGGTAA-C TAGAGATCCC TCAGACCACT --GGAGACC- --GTGCGAA- AATCTCTAGC
A1.IT.02.60000.EU861977           TAGTGTGTGC CCGTCTGTTG TGTRA----- ---------C TCTGGTAA-C TAGAGATCCC TCAGACCACT --CTAGACTG T-GTAAAA-- AATCTCTAGC
A1.KE.01.ML1901PCR2.EU110095      ---------- ---------- ---------- ---------- ---------- ---------- ---------- ---------- ---------- ----------
A1.KE.01.ML1945.EU110088          ---------- ---------- ---------- ---------- ---------- ---------- ---------- ---------- ---------- ----------
A1.KE.02.ML1990.EU110092          ---------- ---------- ---------- ---------- ---------- ---------- ---------- ---------- ---------- ----------
A1.KE.02.ML2014.EU110094          ---------- ---------- ---------- ---------- ---------- ---------- ---------- ---------- ---------- ----------
A1.KE.86.ML170_1986.AF539405      ---------- ---------- ---------- ---------- ---------- ---------- ---------- ---------- ---------- ----------
A1.KE.94.Q23_17.AF004885          TAGTGTGTGC CCGTCTGTTG TGTGA----- ---------C TCTGGTAA-C TAGAGATCCC TCAGACCACT --CTAGACGG T-GTGAA--- AATCTCTAGC
A1.KE.97.ML013_2.AY322185         TAGTGTGTGC CCGTCTGTTG TTTGA----- ---------C TCTGGTAA-C TAGAGATCCC TCAGACCACT --CTAGACGG T-GTAAA--- AATCTCTAGC
A1.KE.97.ML605_3.AY322190         TAGTGTGTGC CCGTCTGTTG TCTGA----- ---------C TCTGGTAA-C TAGAGATCCC TCAGACCACT --CTAGACTG T-GTAAA--- AATCTCTAGC
A1.KE.97.ML752.AY322193           TAGTGTGTGC CCATTTGTTG TAAGA----- ---------C TCTGGTAA-C TAGAGATCCC TCAGACCACT --CTAGACGG T-GTAAA--- AATCTCTAGC
A1.RU.00.RU00051.EF545108         ---------- ---------- ---------- ---------- ---------- ---------- ---------- ---------- ---------- ----------
A1.RU.05.RU_560_1125_JA.JQ2928    ---------- ---------- ---------- ---------- ---------- ---------- ---------- ---------- ---------- ----------
A1.RU.06.RU_915_1016.JQ292896     TAGTGTGTGC CCGTCTGTTG TATGA----- ---------C TCTGGTAA-C TAGAGATCCC TCAGACCACT --CTAGGCGG T-GTAAA--- AATCTCTAGC
A1.RU.06.RU_915_1035.JQ292897     TAGTGTGTGC CCGTCTGTTG TGTGA----- ---------C TCTGGTAA-C TAGAGATCCC TCAGACCACT --CTAGGCGG T-GTAAA--- AATCTCTAGC
A1.RU.06.RU_915_1038.JQ292898     TAGTGTGTGC CCGTCTGTTG TATGA----- ---------C TCTGGTAA-C TAGAGATCCC TCAGACCACT --CTAGGCGG T-GTAAA--- AATCTCTAGC
A1.RU.06.RU_915_1041.JQ292899     TAGTGTGTGC CCGTCTGTTG TGTGA----- ---------C TCTGGTAA-C TAGAGATCCC TCAGACCACT --CTAGGCGG T-GTAAA--- AATCTCTAGC
A1.RU.06.RU_SP_B_049.JQ292900     TAGTGTGTGC CCGTCTGTTG TGTGA----- ---------C TCTGGTAA-C TAGAGATCCC TCAGACCACT --CTAGGCGG T-GTAAA--- AATCTCTAGC
A1.RU.07.Irkutsk_5.JQ292891       ---------- ---------- ---------- ---------- ---------- ---------- ---------- ---------- ---------- ----------
A1.RU.08.PokA1Ru.FJ864679         TAGTGTGTGC CCGTCTGTTG TGTGA----- ---------C TCTGGTAA-C TAGAGATCCC TCAGACCACT --CTAGGCGG T-GTAAA--- AATCTCTAGC
A1.RU.08.RUA001.JQ292893          TAGTGTGTGC CCGTCTGTTG TATGA----- ---------C TCTGGTAA-C TAGAGATCCC TCAGACCACT --CTAGACAG T-GTAAA--- AATCTCTAGC
A1.RU.08.RUA007.JQ292894          TAGTGTGTGC CCGTCTGTTG TGTGA----- ---------C TTTGGTAA-C TAGAGATCCC TCAGACCACT --YTAGGCGG T-GTAAA--- AATCTCTAGC
A1.RU.10.10RU6617.JX500696        TAGTGTGTGC CCATCTGTTG TATGA----- ---------C TCTGGTGA-C TAGAGATCCC TCAGACCACT --CTAGGCGG T-GTAA---- AATCTCTAGC
A1.RU.10.10RU6792.JX500695        TAGTGTGTGC CCGTCTGTTG TGTGA----- ---------C TCTGGTAA-C TAGAGATCCC TCAGACCACT --CTAGGCAG T-GTAAA--- AATCTCTAGC
A1.RU.11.11RU6950.JX500694        TAGTGTGTGC CCGTCTGTTG TATGA----- ---------C TCTGGTAA-C TAGAGATCCC TCAGACCACT --CTAGGCGG T-GTAAA--- AATCTCTAGC
A1.RW.07.pR463F.JX236677          TAGTGTGTGC CCGTCTGTTG TTTGA----- ---------C TCTGGTAA-C TAGAGATCCC TCAGACCACT --CTAGACTG A-GTAAA--- AATCTCTAGC
A1.RW.07.pR880F.JX236678          CAGTGTGTGC CCGTCTGTTG TATGA----- ---------C TCTGGTAA-C TAGAGATCCC TCAGACCACT --CTAGACTG T-GCAAA--- AATCTCTAGC
A1.RW.92.92RW008.AB253421         TAGTGTGTGC CCGTCTGTTG TGTGA----- ---------C TCTGGTAA-C TAGAGATCCC TCAGACCACT --CTAGATTG A-GTAAA--- AATCTCTAGC
A1.RW.92.92RW025A.AB287376        TAGTGTGTGC CCGTCTGTTG TGTGA----- ---------C TCTGGTAA-C TAGAGATCCC TCAGACCACT --CTAGACTG A-GTAAA--- AATCTCTAGC
A1.RW.93.93RW037A.AB287379        TAGTGTGTGC CCGTCTGTTG TATGA----- ---------C TCTGGTAA-C TAGAGATCCC TCAGACCACT --CTAGACTG A-GTAAA--- AATCTCTAGC
A1.SE.95.UGSE8131.AF107771        ---------- ---------- ---------- ---------- ---------- ---------- ---------- ---------- ----A----- AATCTCTAGC
A1.TZ.97.97TZ03.AF361873          ---------- ---------- ---------- ---------- ---------- ---------- ---------- ---------- ---------- ----------
A1.UA.00.98UA0116.AF413987        TAGTGTGTGC CCGTCTGTTG TATGA----- ---------C TCTGGTAA-C TAGAGATCCC TCAGACTACT --CTAGGCAG T-GTAAA--- AATCTCTAGC
A1.UG.07.p191084.JX236669         TAGTGTGTGC CCGTCTGTTG TATGA----- ---------C TCTGGTAA-C TAGAGATCCC TCAGACTACT --CTAGACAG T-GTAAA--- AATCTCTAGC
A1.UG.07.p191845.JX236671         TAGTGTGTGC CCGTCTGTTG TGTGA----- ---------C TCTGGTAA-C TAGAGATCCC TCAGACCACT --CTAGACTG T-ATAAA--- AATCTCTAGC
A1.UG.07.p9004SDM.JX236676        CAGTGTGTGC CCGTCTGTTG TATGA----- ---------C TCTGGTAA-C TAGAGATCCC TCAGACCACT --CTAGACTT A-GTAAA--- AATCTCTAGC
A1.UG.85.U455_U455A.M62320        TAGTGTGTGC CCGTCTGTTA TTTGA----- ---------C TCTGGTAA-C TAGAGATCCC TCAGACCACT --ATAGACTG T-GTAAA--- AATCTCTAGC
A1.UG.92.92UG037.AB253429         TAGTGTGTGC CCGTCTGTTT TGTGA----- ---------C TCTGGTAA-C TAGAGATCCC TCAGACCACT --CTAGACTG A-GTAAA--- AATCTCTAGC
A1.UG.92.UG029.AB098333           TAGTGTGTGC CCGTCTGTTG TGTGA----- ---------C TCTGGTAA-C TAGAGATCCC TCAGACCACT --CTAGAAGG T-GTAAA--- AATCTCTAGC
A1.UG.x.UG031.AB098331            TAGTGTGTGC CCGTCTGTTG TTTGA----- ---------C TCTGGTAA-C TAGAGATCCC TCAGACCACT --CTAGACAG T-GTAAA--- AATCTCTAGC
A1.UG.x.UG275.AB485632            CAGTGTGTGC CCGTCTGTTG TATGA----- ---------C TCTGGTAA-C TAGAGATCCC TCAGATCACT --CTAGACTG T-GTAAA--- AATCTCTAGC
A1.ZA.04.04ZASK162B1.DQ396400     ---------- ---TCTGTTG TGTGG----- ---------C TCTGGTAA-C TAGAGATCCC TCAGACCACT --CTAGACTG A-GTAGA--- AATCTCTAGC
A2.CD.97.97CDKTB48.AF286238       ---------- ---------- ---------- ---------- ---------- ---------- ---------- ---------- ---------- ----------
A2.CY.94.94CY017_41.AF286237      ---------- ---------- ---------- ---------- ---------- ---------- ---------- ---------- ---------- ------TTGA
B.AU.86.MBC200.AF042100           TAGTGTGTGC CCGTCTGTTG TGTGA----- ---------C TCTGGTAA-C TAGAGATCCC TCAGACCCTT --TTAGTCAG T-GTGGAA-- AATCTCTAGC
B.AU.87.MBC925.AF042101           TAGTGTGTGC CCGTCTGTTG TGTGA----- ---------C TCTGGTAA-C TAGAGATCCC TCAGACCCTT --TTAGTCAG T-GTGGAA-- AATCTCTAGC
B.AU.95.C24.AF538304              TAGTGTGTGC CCGTCTGTTG TGTGA----- ---------C TCTGGTAA-C TAGAGATCCC TCAGACCCTT --TTAGTCAG T-GTGGAG-- AATCTCTAGC
B.AU.95.C42.AF538305              TAGTGTGTGC CCGTCTGTTG TGTGA----- ---------C TCTGGTAT-C TAGAGATCCC TCAGACCAAT --TTAGTCAG T-GTGGAA-- AATCTCTAGC
B.AU.95.C76.AF538306              TAGTGTGTGC CCGTCTGTTG TGTGA----- ---------C TCTGGTAG-C TAGAGATCCC TCAGACCCTT --TTAGTCAG T-GTGGAA-- AATCTCTAGC
B.AU.95.C92.AF538307              TAGTGTGTGC CCGTCTGTTG TGTGA----- ---------C TCTGGTAA-C TAGAGATCCC TCAGACCCTT --TTAGTCAG T-GTGGAG-- AATCTCTAGC
B.AU.95.MBCC54.AF042103           ---------- ---------- ---------- ---------- ---------- ---------- ---------- ---------- ---------- ----------
B.AU.96.MBCC98.AF042104           ---------- ---------- ---------- ---------- ---------- ---------- ---------- ---------- ---------- ----------
B.AU.96.MBCD36.AF042105           ---------- ---------- ---------- ---------- ---------- ---------- ---------- ---------- ---------- ----------
B.AU.99.1181.AF538302             TAGTGTGTGC CCGTCTGTTG TGTGA----- ---------C TCTGGTAG-C TAGAGATCCC TCAGACCCTT --TAATTCCG T-GTAGAA-- AATCTCTAGC
B.AU.x.2870718.AY857022           ---------- ---------- ---------- ---------- ---------- ---------- ---------- ---------- ---------- ----------
B.AU.x.9125091.AY857165           ---------- ---------- ---------- ---------- ---------- ---------- ---------- ---------- ---------- ----------
B.AU.x.VH_VHPCR.AF146728          ---------- ---------- ---------- ---------- ---------- ---------- ---------- ---------- ---------- ----------
B.BO.09.DEMB09BO001.JX140656      ---------- ---------- ---------- ---------- ---------- ---------- ---------- ---------- ---------- ----------
B.BR.02.02BR008.DQ358808          ---------- ---------- ---------- ---------- ---------- ---------- ---------- --------AG T-GTGGAA-- AATCTCTAGC
B.BR.02.02BR011.DQ358809          TAGTGTGTGC CCGTCTGTTG TGTGA----- ---------C TCTGGTAA-C TAGAGATCCC TCAGACCATT --TAAGTCAG T-GTGGAA-- AATCTCTAGC
B.BR.02.02BR013.DQ358810          TAGTGTGTGC CCGTCTGTTG TGTGA----- ---------C TCTCGTATAC TAGAGATCCC TCAGACC-TT --TTAGTCAG T-GTGGAAA- AATCTCTAGC
B.BR.02.02BR1013.JN692432         TAGTGTGTGC CCGTCTGTTG TGTGA----- ---------C TCTGGTAA-C TAGAGATCCC TCAGACCCTT --AAAGTCAG T-GTGGAAA- AATCTCTAGC
B.BR.02.02BR2025.JN692435         TAGTGTGTGC CCGTCTGTTG TGTGA----- ---------C TCTGGTAA-C TAGAGATCCC TCAGACCCTT --TTAGTCAG T-GTGGAA-- AATCTCTAGC
B.BR.02.02BR2032.JN692439         ---------- ---------- ---------- ---------- ---------- ---------- ---------- ---------- ---------- ----------
B.BR.02.02BR2033.JN692440         AGGTGTGTGC CCGTCTGTTG TTTGA----- ---------C TCTGGTAA-C TAGAGATCCC TCAGACCATC --TTAGACAG T-GGAAA--- AATCTCTAGC
B.BR.02.02BR2041.JN692443         ATAGTAGTGC CCGTCTGTTG TGTGA----- ---------C TCTGGTAA-C TAGAGATCCC TCAGACCC-- --TATTAGTC A-GTGTGGAA AATCTCTAGC
B.BR.02.02BR2042.JN692444         ---------C CCGTCTGATT TGTGA----- ---------C TCTGGTAA-C TAGAGATCCC TCAGACACTT --TTAGTTCG T-GTGGAA-- AATCTCTAGC
B.BR.02.04BR1064.JN692433         TAGTGTGTGC CCGTCTGTTG TGTGA----- ---------C TCTGGTAA-C TAGAGATCCC TCAGACACCT --TTAGTCAG T-GTGGAAA- AATCTCTAGC
B.BR.02.05BR1094.JN692470         ------GTGC CCGTCTGATT TGTGA----- ---------C TCTGGTAA-C TAGAGATCCC TCAGACACTT --TTAGTTCG T-GTGGAA-- AATCTCTAGC
B.BR.03.03BR1020.JN692445         TAGTGTGTGC CCGTCTGTTG TGTGA----- ---------C TCTGGTAA-C TAGAGATCCC TCAGACCTTT --AAAGTCAG T-GTGGAA-- AATCTCTAGC
B.BR.03.03BR1046.JN692447         TAGTGTGTGC CCGTCTGTTG TGTGA----- ---------C TCTGGTAA-C TAGAGATCCC TCAGACCCAC --TTAGTCCG A-GTGAAA-- AATCTCTAGC
B.BR.03.BREPM1023.EF637057        ---------- ---------- ---------- ---------- TCTGGTAA-C TAGAGATCCC TCAGACCCTT --TTAGTCAG T-GTGGAA-- AATCTCTAGC
B.BR.03.BREPM1024.EF637056        ---------- ---------- ---------- ---------- ---------- ---------- -------AAT --TTAGTCAG T-GTGGAA-- AATCTCTAGC
B.BR.03.BREPM1027.EF637054        ---------- ---------- ---------- ---------- ---------- ---------- ---------- ---------- ---------- -ATCTCTAGC
B.BR.03.BREPM1028.EF637053        ---------- ---------- ---------- ---------- ---------- ---------- ---------- ---------- ---------- ----------
B.BR.03.BREPM1032.EF637051        ---------- ---------- ---------- ---------- ---------- ---------- ---------- ---------- ---------- ----------
B.BR.03.BREPM1033.EF637050        ---------- ---------- ---------- ---------- ---------- ---------- -----CCATT --TAGATCAG T-GTGGAA-- AATCTCTAGC
B.BR.03.BREPM1035.EF637049        ---------- ---------- ---------- ---------- ---------- ---------- ---------- ---------- ---------- ----------
B.BR.03.BREPM1038.EF637048        ---------- ---------- ---------- ---------- -ATGGTAA-C TAGAGATCCC TCAGACCATT --CTAGTCAG T-GTGGAA-- AATCTCTAGC
B.BR.03.BREPM1040.EF637047        ---------- ---------- ---------- ---------- ---------- ---------- --AGACCAAT --TTAGTCAG T-GTGGAA-- AATCTCTAGC
B.BR.03.BREPM2012.EF637046        ---------- ---------- ---------- ---------- ---------- ---------- -------TT- --TTAGTCAG T-GTGGAA-- AATCTCTAGC
B.BR.04.04BR1047.JN692450         ---------- ---------- ---------- ---------- ---------- ---------- -CAGACCATT --TTAGTCAG T-GTGAA--- AATCTCTAGC
B.BR.04.04BR1049.JN692451         TAGTGTGTGC CCGTCTGTTG TGTGA----- ---------C TCTGGTAA-C TAGAGATCCC TCAGACCCTT --TTAGTCAG T-GTGGAA-- AATCTCTAGC
```

```
B.BR.04.04BR1051.JN692452         TAGTGTGTGC CCGTCTGTTG TGTGA----- ---------C TCTGGTAA-C TAGAGATCCC TCAGACACTT --GTAGTCAG T-GTGGAA-- AATCTCTAGC
B.BR.04.04BR1054.JN692453         ---------- ---------- ---------- ---------- ---------C TGGAGATCCC TCAGACCCAT --TTAGTCA- --GTGTGGAA AATCTCTAGC
B.BR.04.04BR1055.JN692454         TAGTGTGTGC CCGTCTGTTG TGTAA----- ---------C TCTAGTAA-C TAAAAATCCC TCAAACCCTT --TTAGTCAG T-GTGGAA-- AATCTCTAGC
B.BR.04.04BR1057.JN692455         TAGTGTGTGC CCGTCTGTTG TGTGA----- ---------C TCTGGTAA-C TAGAGATCCC TCAGACCTTT --CAGTCAGT --GCGGAAA- AATCTCTAGC
B.BR.04.04BR1068.JN692457         ---------- ---------- ---------- ---------- ---------C TAGAGATCCC TCAGACATTG --TAGTCAGT --GTGGAAA- AATCTCTAGC
B.BR.04.BREPM1066.FJ195090        TAGTGTGTGC CCGTCTGTTG TGTGA----- ---------C TCTGGTAA-C TAGAGATCCC TCAGACACT- --TTAGTCAG T-GTGGAA-- AATCTCTAGC
B.BR.04.BREPM1070.FJ195086        ---------- --TTCGGTGG TGTGA----- ---------T TCTGGTAC-C TAGAGTTCCT TCAGACCTTA --TTGTTCAG T-TGGGAA-- ATTCTTTGCC
B.BR.05.05BR1077.JN692460         ---------- ---------- ---------- ---------- ---------- ---------- ---------- ---------- ---------- ----------
B.BR.05.05BR1078.JN692461         ---------- ---------- ---------- ---------- ---------- ---------- ---------- ---------- ---------- ----------
B.BR.05.05BR1079.JN692462         ---------C CCGTCTGTTG TGTGA----- ---------C TCTGGTAA-C TAGAGATCCC TCAGACCCTT --TTAGTCAG T-GTGGAA-- AATCTCTAGC
B.BR.05.05BR1080.JN692463         ---------- ---------- ---------- ---------- ---------- ---------- ---------- ---------- ---------- ----------
B.BR.05.05BR1082.JN692465         TAGTGTGTGC CCGTCTGTTG TTTGA----- ---------C TCTGGTAA-C TAGAGATCCC TCAGACCATT --TTAGTCAG T-GTGGAA-- AATCTCTAGC
B.BR.05.05BR1089.JN692467         ---------- ---------- ---------- ---------- ---------- ---------- ---------- ---------- ---------- ----------
B.BR.05.05BR1092.JN692468         TTATGTGTGC CCGTCTGTTG TGTGA----- ---------C TCTGGTAA-C TAGAGATCCC TCAGACCAAT --TTAGTCAG T-GTGGAA-- AATCTCTAGC
B.BR.05.05BR1095.JN692471         TAGTGTGTGC CCGTCTGTTG TGTGA----- ---------C TCTGGTAA-C TAGAGATCCC TCAGACCTTT --GTAGTCAG T-GTGGAA-- AATCTCTAGC
B.BR.05.05BR1101.JN692473         TAGTGTGTGC CCGTCTGTTG TGTGA----- ---------C TCTGGTAA-C TAGAGATCCC TCAGACTCTT --CTAGTCAG T-GTGGAA-- AATCTCTAGC
B.BR.05.05BR1104.JN692474         TTATGTGTGC CCGTCTGTTG TGTGA----- ---------C TCTGGTAA-C TAGAGATCCC TCAGACCCTT --TAAGTCAG T-GTGGAA-- AATCTCTAGC
B.BR.05.05BR1107.JN692475         TAGTGTGTGC CCGTCTGTTG TGTGA----- ---------C TCTGGTAA-C TAGAGATCCC TCAGACCATT --TAAGTCAG T-GTGGAA-- AATCTCTAGC
B.BR.05.BREPM1081.FJ195091        TAGTGTGTGC CCGTCTGTTG TGTGA----- ---------C TCTGGTAA-C TAGAGATCCC TCAGACACTT --TTAATCCG T-GTGGAA-- AATCTCTAGC
B.BR.05.BREPM1084.FJ195088        ---------- ---------- ---------- ---------- ---------- -------CCC TCAGACCCTT --TTAGTCAG T-GTGGAA-- AATCTCTAGC
B.BR.05.BREPM1093.FJ195089        TAGTGTGTGC CCGTCTGTTG TGTGA----- ---------C TCTGGTAA-C TAGAGATCCC TCAGACACTT --TTAGTCAG T-GTGGAA-- AATCTCTAGC
B.BR.06.06BR1115.JN692479         TAGTGTGTGC CCGTCTGTTG TGTGA----- ---------C TCTGGTAA-C TAGAGATCCC TCAGACCATT --TTAGTCAG A-GTGGAA-- AATCTCTAGC
B.BR.06.06BR1119.JN692480         ---------- ---------- ---------- ---------- ---------- --GAGATCCC TCAGACCCTT --TTAGTCAG T-GTGGAA-- AATCTCTAGC
B.CA.06.502_1027_wg01.JF320413    TAGTGTGTGC CCGTCTGTTG TGTGA----- ---------C TCTGGTAAAC TAGAGATCCC TCAGACCC-- --TTTTTAGT CAGTGTGGAA AATCTCTAGC
B.CA.06.502_1799_FL02.JF320427    ---------- ---------- ---------- ---------- --TGGTAA-C TAGAGATCCC TCAGACCTTT ---TAGTCAG T-GTGGAA-- AATCTCTAGC
B.CA.07.502_1191_03.JF320424      ---------- ---------- ---------- ---------- --TGGTAA-C TAGAGATCCC TCAGACCATT --TTAGTCAG T-GTGGAA-- AATCTCTAGC
B.CA.96.WC10C_4.AY314056          -AGTGTGTGC CCGTCTGTTG TGTGA----- ---------C TCTGGTAA-C TAGAGATCCC TCAGACTCTT --TTAGTCAG T-GTGGAA-- AATCTCTAGC
B.CA.97.CANB3FULL.AY779553        ---------- ---------- ---------- ---------- ---------- ---------- ---------- ---------- ---------- ----------
B.CH.03.HIV_CH_BID_V4470_2003.    ---------- ---------- ---------- ---------- ---------- ---------- ---------- ---------- ---------- ----------
B.CN.01.CNHN24.AY180905           ---------- ---------- ---------- ---------- ---------- ---------- ---------- ---------- ----GATT-- AATCTCTAGC
B.CN.02.02HNsc11.DQ007903         TAGTGTGTGC CCGTCTGTTG TGTGA----- ---------C TCTGGTAA-C TAGAGATCCC TCAGAACCTA --TTAGTCAG T-GTGGAA-- AATCTCTAGC
B.CN.03.SHXDC0081.JF932492        TAGTGTGTGC CCGTCTGTTG TGTGA----- ---------C TCTGGTAG-C TAGAGATCCC TCAGACCCTA --ATAGTCAG T-GTGGAA-- AATCTCTAGC
B.CN.05.05CNHB_hp3.DQ990880       TAGTGTGTGC CCGTCTGTTG TGTGA----- ---------C TCTGGTAA-C TAGAGATCCC TCAGACCCTT ---TAGTTTG T-GTGGAA-- AATCTCTAGC
B.CN.06.CC056.JF932482            ---------C CCGTCTGTTG TGTGA----- ---------C TCTGGTAA-C TAGAGATCCC TCAGACCCTT --TTAGTCAG T-GTGGAA-- AATCTCTAGC
B.CN.07.AH070011.JF932468         ---------- ---------- ---------- ---------- ---------- ---------- ---------- ---------- ---------- ----------
B.CN.07.AH070014.JF932469         ---------- CCGTCTGTTG TGTGA----- ---------C TCTGGTAA-C TAGAGATCCC TCAGACCTTT ---TAGTCAG T-GTGGAA-- AATCTCTAGC
B.CN.07.AH070017.JF932470         ---------- ---------- ----A----- ---------C TCTGGTAA-C TAGAGATCCC TCAGACACTT --TTAGTCAG T-GTGGAA-- AATCTCTAGC
B.CN.07.AH070018.JF932471         --------GC CCGTCTGTTG TGTGA----- ---------C TCTGGTAG-C TAGAGATCCC TCAGAACAGT --TTAGTTAG T-GTGGAA-- AATCTCTAGC
B.CN.07.AH070057.JF932472         ---------- -CGTCTGTTG TGTGA----- ---------C TCTGGTAA-C TAGAGATCCC TCAGACCCTT --TTAGTCAG T-GTGGAA-- AATCTCTAGC
B.CN.07.BJ070030.JF932473         --GTGTGTGC CCGTCTGTTG TGTGA----- ---------C TCTGGTAA-C TAGAGATCCC TCAGACACTT --CTAGTCAG T-GTGGAA-- AATCTCTAGC
B.CN.07.CBJC261.JF932474          --------GC CCGTCTGTTG TGTGA----- ---------C TCTGGTAG-C TAGAGATCCC TCAGAACCTT --TTAGTCAG T-GTGGAA-- AATCTCTAGC
B.CN.07.CBJC392.JF932475          --------GC CCGTCTGTTG TGTGA----- ---------C TCTGGTAA-C TAGAGATCCC TCAGACCCAT --TTAGTCAG T-GTGGAA-- AATCTCTAGC
B.CN.07.CBJC394.JF932476          ---------- ---------- ---------- ---------- ---------- ---------- ---------- ---------- ---------- --TCTCTAGC
B.CN.07.CBJC396.JF932477          ---------C CCGTCTGTTG TGTGA----- ---------C TCTGGTAG-C TAGAGATCCC TCAGAACCTT --TTAGTTAG T-GTGGAA-- AATCTCTAGC
B.CN.07.GS070017.JF932484         ---------- ---------- ---------- ---------- ---------- ---------- ---------- ---------- ---------- ----------
B.CN.07.GZ070002.JF932485         ---------- ---------- ---------- ---------- ---------- ---------- ---------- ---------- ---------- ----------
B.CN.07.GZ070030.JF932486         TAGTGTGTGC CCGTCTGTTG TGTGA----- ---------C TCTGGTAA-C TAGAGATCCC TCAGACCTTT ---TAGTCAG T-GTGGAA-- AATCTCTAGC
B.CN.07.HB070006.JF932487         ---------- ---------- ---------- ---------- ---------- ---------- ---------- ---------- ---------- ----------
B.CN.07.HB070022.JF932488         --------GC CCGTCTGTTG TGTGA----- ---------C TCTGGTAG-C TAGAGATCCC TCAGACCCTT --TTAGTCAG T-GTGGAA-- AATCTCTAGC
B.CN.07.HB070035.JF932489         TAGTGTGTGC CCGTCTGTTG TGTGA----- ---------C TCTGGTAA-C TAGAGATCCC TCAGACCCTT --TTAGTCAG T-GTGGAAA- AATCTCTAGC
B.CN.07.JL070038.JF932490         ---------- ---------- ---------- ---------- ---------- ---------- ---------- ---------- ---------- ----------
B.CN.07.SX070080.JF932493         ---------- ---------- ---------- ---------- ---------- ---------- ---------- ---------- ---------- ----------
B.CN.07.hb070025.JF932499         TAGTGTGTGC CCGTCTGTTG TGTGA----- ---------C TCTGGTAA-C TAGAGATCCC TCAGACCCTT --TTAGTCAG T-GTGGAA-- AATCTCTAGC
B.CN.07.hen1345.JF932500          ------GTGC CCGTCTGTTG TGTGA----- ---------C TCTGGTAA-C TAGAGATCCC TCAGACCTTT ---TAGTCAG T-GTGGAA-- AATCTCTAGC
B.CN.08.CBJC476.JF932478          TAGTGTGTGC CCGTCTGTTG TGTGA----- ---------C TCTGGTAG-C TAGAGATCCC TCAGACCTTT ---TAGTCAG T-GTGGAA-- AATCTCTAGC
B.CN.08.CBJC489.JF932479          ---------C CCGTCTGTTG TGTGA----- ---------C TCTGGTAA-C TAGAGATCCC TCAGAACCGT --GTAGTTAG T-GTGGAA-- AATCTCTAGC
B.CN.08.CBJC500.JF932480          TAGTGTGTGC CCGTCTGTTG TGTGA----- ---------C TCTGGTAA-C TAGAGATCCC TCAGACCCTT --TTAGTCAG T-GTGGAA-- AATCTCTAGC
B.CN.08.CBJC503.JF932481          TAGTGTGTGC CCGTCTGTTG TGTGA----- ---------C TCTGGTAA-C TAGAGATCCC TCAGACTCTT ---TAGTCAG T-GTGGAA-- AATCTCTAGC
B.CN.08.cbjc468.JF932498          TAGTGTGTGC CCGTCTGTTG TGTGA----- ---------C TCTGGTAA-C TAGAGATCCC TCAGACCCCT --TTAGTCAG T-GTGGAA-- AATCTCTAGC
B.CN.09.YN09P0014.JF932494        ---------C CCGTCTGTTG TGTGA----- ---------C TCTGGTAA-C TAGAGATCCC TCAGACCCTT --TTAGTCAG T-GTGGAA-- AATCTCTAGC
B.CN.09.ZK042.JF932497            ---------C CCGTCTGTTG TGTGA----- ---------C TCTGGTAA-C TAGAGATCCC TCAGAACCTT --TTAGTCTG T-GTGGAA-- AATCTCTAGC
B.CN.10.DEMB10CN002.JX140658      ---------- ---------- ---------- ---------- ---------- ---------- ---------- ---------- ---------- ----------
B.CN.98.YN9802.JF932495           ---------- ---------- ---------- ---------- ---------- ---------- ---------- ---------- ---------- ----------
B.CN.98.YN9838.JF932496           ---------- ---------- ---------- ---------- ---------- ---------- ---------- ---------- ---------- ----------
B.CN.99.plwj.GU177863             TAGTGTGTGC CCGTCTGTTG TGTGA----- ---------C TCTGGTAA-C TAGAGATCCC TCAGACCTTT ---TAGTCAG T-GTGGAA-- AATCTCTAGC
B.CN.x.RL42.U71182                ---------- ---------- ---------- ---------- ---------- ---------- ---------- ---------- ---------- AATCTCTAGC
B.CU.99.Cu19.AY586542             TAGTGTGTGC CCGTCTGTTG TGTGA----- ---------C TCTGGTAA-C TAGAGATCCC TCAGACCTTC --TTAGTCAG T-GTGGAA-- AATCTCTAGC
B.CU.99.Cu43.AY586543             TAGTGTGTGC CCGTCTGTTG TGTGA----- ---------C TCTGGTAA-C TAGAGATCCC TCAGACCTTC --TTAGTCAG T-GTGGAA-- AATCTCTAGC
B.DE.86.D31.U43096                TAGTGTGTGC CCGTCTGTTG TGTGA----- ---------C TCTGGTAA-C TAGAGATCCC TCAGACCCTT --TTAGTCAG T-GTGGAA-- AATCTCTAGC
B.DE.86.HAN.U43141                TAGTGTGTGC CCGTCTGTTG TGTGA----- ---------C TCTGGTAG-C TAGAGATCCC TCAGATCCTT --TTAGTCAG T-GTGGAA-- AATCTCTAGC
B.DK.01.CTL_016.EF514704          ---------- ---------- ---------- ---------- ---------- ---------- ---------- ---------- ---------- ----------
B.DK.01.CTL_017.EF514705          ---------- ---------- ---------- ---------- ---------- ---------- ---------- ---------- ---------- ---------G
B.DK.01.CTL_018.EF514706          ---------- ---------- ---------- ---------- ---------- ---------- ---------- ---------- ---------- ----------
B.DK.01.CTL_023.EF514707          ---------- ---------- ---------- ---------- ---------- ---------- ---------- ---------- ---------- ----------
B.DK.01.CTL_030.EF514708          ---------- ---------- ---------- ---------- ---------- ---------- ---------- ---------- ---------- --------TT
B.DK.01.CTL_033.EF514709          ---------- ---------- ---------- ---------- ---------- ---------- ---------- ---------- ---------- ----------
B.DK.01.CTL_035.EF514710          ---------- ---------- ---------- ---------- ---------- ---------- ---------- ---------- ---------- ----------
B.DK.01.CTL_041.EF514711          ---------- ---------- ---------- ---------- ---------- ---------- ---------- ---------- ---------- ----------
B.DK.01.CTL_043.EF514712          ---------- ---------- ---------- ---------- ---------- ---------- ---------- ---------- ---------- ----------
B.DK.04.PMVL_012.EF514699         ---------- ---------- ---------- ---------- ---------- ---------- ---------- ---------- ---------- ----------
B.DK.04.PMVL_013.EF514700         ---------- ---------- ---------- ---------- ---------- ---------- ---------- ---------- ---------- ----------
B.DK.04.PMVL_018.EF514697         ---------- ---------- ---------- ---------- ---------- ---------- ---------- ---------- ---------- ----------
B.DK.04.PMVL_025.EF514702         ---------- ---------- ---------- ---------- ---------- ---------- ---------- ---------- ---------- ----------
B.DK.04.PMVL_027.EF514698         ---------- ---------- ---------- ---------- ---------- ---------- ---------- ---------- ---------- ----------
B.DK.04.PMVL_039.EF514703         ---------- ---------- ---------- ---------- ---------- ---------- ---------- ---------- ---------- ----------
B.DK.04.PMVL_049.EF514701         ---------- ---------- ---------- ---------- ---------- ---------- ---------- ---------- ---------- ----------
B.DK.07.PMVL_011.FJ694790         ---------- ---------- ---------- ---------- ---------- ---------- ---------- ---------- ---------- ----------
B.ES.05.X1890.EU786672            ------GTGC CCGTCTGTTG TTTGA----- ---------C TCTGGTAT-C TAGAGATCCC TCAGACCCAT --TTAGTCAG T-GTGGAA-- AATCTCTAGC
B.ES.06.X1959.EU786675            ---------- ---------- ---------- ---------- ---------- ---------- ---------- ---------- ---------- ----TCTAGC
B.ES.06.X2102.EU786677            -------TGC CCGTCTGTTG TGTGA----- ---------C TCTGGTAA-C TAGAGATCCC TCAGACCCTT --TTAGTCAG T-GTGGAA-- AATCTCTAGC
B.ES.07.X2149.EU786678            ------GTGC CCGTCTGTTG TGTGA----- ---------C TCTGGTAA-C TAGAGATCCC TCAGACCATT --TTAGTCAG T-GTGGAA-- AATCTCTAGC
B.ES.07.X2210_3.EU786679          -------TGC CCGTCTGTTG TGTGA----- ---------C TCTGGTAA-C TAGAGATCCC TCAGACACAG --CTAGTCAG T-GTGGAA-- TATCTCTAGC
B.ES.07.X2231.EU786680            -------TGC CCGTCTGTTG TGTGA----- ---------C TCTGGTAA-C TAGAGATCCC TCAGACCACT --TTAGTCCG T-GTGGAA-- AATCTCTAGC
B.ES.08.ES_X2515_3.GQ372988       TAGTGTGTGC CCGTCTGTTG TGTGA----- ---------C TCTGGTAA-C TAGAGATCCC TCAGACCTTT --T-AGTCAG T-GTGGAA-- AATCTCTAGC
B.ES.08.ES_X2556_3.GQ372990       TAGTGTGTGC CCGTCTGTTG TGTGA----- ---------C TCTGGTAA-C TAGAGATCCC TCAGACCCTT --TTAGTCAG T-GTGGAA-- AATCTCTAGC
B.ES.08.P2008.FJ670531            -AAGGGTTGC CCGTCTGTTG TGTGA----- ---------C TCTGGTAA-C TAGAGATCCC TCAGACCCTT --TAAGTCAG T-GTGGAA-- AATCTCTAGC
B.ES.08.X2425_2.FJ670525          -------TGC CCGTCTGTTG TGTGA----- ---------C TCTGGTAA-C TAGAGATCCC TCAGACACTT --GTAGTCTG T-GTAGAA-- AATCTCTAGC
B.ES.08.X2510_2.FJ853622          -----TGTGC CCGTCTGTTG TGTGA----- ---------C TCTGGTAA-C TAGAGATCCC TCAGACCCTT --TTAGTCAG T-GTGGAA-- AATCTCTAGC
B.ES.08.X2555_2.GU362883          TAGTGTGTGC CCGTCTGTTG TGTGA----- ---------C TCTGGTAA-C TAGAGATCCC TCAGACCCTT --TTAGTCAG T-GTGGAA-- AATCTCTAGC
B.ES.08.X2574_2.GU362886          -AGTGTGTGC CCGTCTGTTG TGTGA----- ---------C TCTGGTAA-C TAGAGATCCC TCAGACCCTT --TTAGTCAG T-GTGGAA-- AATCTCTAGC
B.ES.09.P2149_3.GU362881          TAGTGTGTGC CCGTCTGTTG TGTGA----- ---------C TCTGGTAA-C TAGAGATCCC TCAGACCATT ---TAGTCAG T-GTGGAAA- AATCTCTAGC
B.ES.09.X2689_2.GU362885          -AGTGTGTGC CCGTCTGTTG TGTGA----- ---------C TCTGGTAA-C TAGAGATCCC TCAGACTCTT --GTAGTTAG T-GTGGAA-- AATCTCTAGC
B.ES.89.U61.DQ854716              TAGTGTGTGC CCGTCTATTG TGTGA----- ---------C TCTGGTAG-C TAGAGATCCC TTCAGACCAA ATTTAGTCAG T-GTGAAA-- AATCTCTAGC
B.FR.05.DEMB05FR001.JX140652      ---------- ---------- ---------- ---------- ---------- ---------- ---------- ---------- ---------- ----------
B.FR.08.DEMB08FR002.JX140654      ---------- ---------- ---------- ---------- ---------- ---------- ---------- ---------- ---------- ----------
```

B.GA.88.OYI_397.M26727	TAGTGTGTGC	CCGTCTGTTG	TGTGA-----	---------C	TCTGGTAA-C	TAGAGATCCC	TCAGACCCTT	--TTAGTCAG	T-GTGGAA--	AATCTCTAGC
B.GB.05.MM45d213_GN1.HM586212	----------	----------	----------	----------	----------	----------	----------	----------	----------	--TCTCTAGC
B.GB.83.CAM1.D10112	TAGTGTGTGC	CCATCTGTTG	TGTGA-----	---------C	TCTGGTAA-C	TAGAGATCCC	TCAGACCCTT	--TTAGTCAG	T-GTGGAA--	AATCTCTAGC
B.GB.86.GB8_46R.AJ271445	TAGTGTGTGC	CCGTCTGTTG	TGTGA-----	---------C	TCTGGTAG-C	TAGAGATCCC	TCAGACCCTT	--TTAGTCAG	T-GTAGAA--	AATCTCTAGC
B.GB.x.MANC.U23487	TAGTGTGTGC	CCGTCTGTTG	TGTGA-----	---------C	TCTGGTAA-C	TAGAGATCCC	TCAGACCCTT	--TTAGTCAG	T-GTGGAA--	AATCTCTAGC
B.HK.06.HK003.FJ460500	----------	----------	----------	----------	----------	----------	----------	----------	----------	----------
B.HK.06.HK004.FJ460501	----------	----------	----------	----------	----------	----------	----------	----------	----------	----------
B.IN.x.11807.EF694037	----------	----------	----------	----------	----------	----------	----------	----------	----------	----------
B.IT.05.SG1.DQ672623	TAGTGTGTGC	CCGTCTGTTG	TGTGA-----	---------C	TCTGGTAA-C	TAGAGATCCC	TCAGACCCTT	--GTAGTCAG	T-GTGGAA--	AATCTCTAGC
B.JP.00.DR2508.AB289588	TAGTGTGTGC	CCGTCTGTTG	TGTGA-----	---------C	TCTGGTAA-C	TAGAGATCCC	TCAGACCTCG	--GTAGTCAG	T-GTAGAA--	AATCTCTAGC
B.JP.01.DR388.AB289590	TAGTGTGTGC	CCGTCTGTTG	TGTGA-----	---------C	TCTGGTAA-C	TAGAGATCCC	TCAGACCATT	--TAAGTCAG	T-GTGGAA--	AATCTCTAGC
B.JP.04.04JPDR6075B.AB221125	TAGTGTGTGC	CCGTCTGTTG	TGTGA-----	---------C	TCTGGTAA-C	TAGAGATCCC	TCAGACCCTT	--TTAGTCAG	T-GTGGAA--	AATCTCTAGC
B.JP.04.DR5913.AB480696	TAGTGTGTGC	CCGTCTGTTG	TGTGA-----	---------C	TCTGGTAT-C	TAGAGATCCC	TCAGACACTT	--TTAGTCAG	T-GTGGAA--	AATCTCTAGC
B.JP.04.DR6089.AB286955	TAGTGTGTGC	CCGTCTGTTG	TGTGA-----	---------C	TCTGGTAA-C	TAGAGATCCC	TCAGACCATT	--TAAGTCAG	T-GTGGAA--	AATCTCTAGC
B.JP.04.DR6174.AB480692	TAGTGTGTGC	CCGTCTGTTG	TGTGA-----	---------C	TCTGGTAA-C	TAGAGATCCC	TCAGACCCTT	--TAAGTCAG	T-GTGGAA--	AATCTCTAGC
B.JP.04.DR6175.AB480694	TAGTGTGTGC	CCGTCTGTTG	TGTGA-----	---------C	TCTGGTAA-C	TAGAGATCCC	TCAGACATTT	--ATAGTCAG	T-GTGGAA--	AATCTCTAGC
B.JP.05.DR6538.AB287363	TAGTGTGTGC	CCGTCTGTTG	TGTGA-----	---------C	TCTGGTAA-C	TAGAGATCCC	CCAGACCCTT	--TTAGTCAG	T-GTGGAA--	AATCTCTAGC
B.JP.05.DR6737.AB287364	TAGTGTGTGC	CCGTCTGTTG	TGTGA-----	---------C	TCTGGTAA-C	TAGAGATCCC	TCAGACCATT	--TTAGTCCG	T-GTGGAA--	AATCTCTAGC
B.JP.05.DR7060.AB287366	TAGTGTGTGC	CCGTCTGTTG	TGTGA-----	---------C	TCTGGTAA-C	TAGAGATCCC	TCAGACATTT	--GTAGTCAG	T-GTGGAA--	AATCTCTAGC
B.JP.05.DR7065.AB287368	TAGTGTGTGC	CCATCTGTTG	TGTGA-----	---------C	TCTGGTAG-C	TAGAGATCCC	TCAGATATCT	--TTAGTCCG	T-GTGGAAA-	AATCTCTAGC
B.JP.98.DR1120.AB480698	TAGTGTGTGC	CCGTCTGTTG	TGTGA-----	---------C	TCTGGTAA-C	TAGAGATCCC	TCAGACCCTT	--TTAGTCAG	T-GTGGAA--	AATCTCTAGC
B.JP.99.DR1348.AB287370	TAGTGTGTGC	CCGTCTGTTG	TGTGA-----	---------C	TCTGGTAA-C	TAGAGATCCC	TCAGACCCTT	--TTAGTCAG	T-GTGGAA--	AATCTCTAGC
B.JP.x.DR1673.AB564745	TAGTGTGTGC	CCGTCTGTTG	TGTGA-----	---------C	TCTGGTAA-C	TAGAGATCCC	TCAGACC-TT	--TTAGTCAG	T-GTGGAA--	AATCTCTAGC
B.JP.x.DR1712.AB604946	TAGTGTGTGC	CCGTCTGTTG	TGTGA-----	---------C	TCTGGTAA-C	TAGAGATCCC	TCAGACCA-T	--TTAGTCAG	T-GTGGAA--	AATCTCTAGC
B.JP.x.DR1777.AB604948	TAGTGTGTGC	CCGTCTGTTG	TGTGA-----	---------C	TCTGGTAA-C	TAGAGATCCC	TCAGACTATT	--TTAGTCAG	T-GTGAAA--	AATCTCTAGC
B.JP.x.JRC03B.AB565496	TAGTGTGTGC	CCGTCTGTTG	TGTGA-----	---------C	TCTGGTAA-C	TAGAGATCCC	TCAGACCCTT	--TTAGTCAG	T-GTGGAA--	AATCTCTAGC
B.JP.x.JRC05B.AB565497	TAGTGTGTGC	CCGTCTGTTG	TGTGA-----	---------C	TCTGGTAA-C	TAGAGATCCC	TCAGACACTT	--GTAGTCAG	T-GTGAAA--	AATCTCTAGC
B.JP.x.JRC65B.AB565502	TAGTGTGTGC	CCGTCTGTTG	TGTGA-----	---------C	TCTGGTAA-C	TAGAGATCCC	TCAGACCCTT	--TTAGTCAG	T-GTGGAA--	AATCTCTAGC
B.JP.x.pJPDR0796B02.AB565478	TAGTGTGTGC	CCGTCTGGTA	TTTGA-----	---------C	TCTGGTAA-C	TAGAGATCCC	TCAGACCCCT	--TTAGTCAG	T-GTGGAA--	AATCTCTAGC
B.JP.x.pJRC57B09.AB641836	TAGTGTGTGC	CCGTCTGTTG	TGTGA-----	---------C	TCTGGTAA-C	TAGAGATCCC	TCAGACCCTT	--TTAGTCAG	T-GTGGAA--	AATCTCTAGC
B.KR.02.02OSG1.JQ429433	TAGTGTGTGC	CCGTCTGTTG	TGTGA-----	---------C	TCTGGTAA-C	TAGAGATCCC	TCAGACACTT	--GTAGTCAG	T-GTGGAA--	AATCTCTAGC
B.KR.03.03HJY8.JQ316131	TAGTGTGTGC	CCGTCTGTTG	TGTGA-----	---------C	TCTGGTAA-C	TAGAGATCCC	TCAGACCAAT	--TTAGTCAG	T-GTGGAA--	AATCTCTAGC
B.KR.03.03KDE11.JQ316128	TAGTGTGTGC	CCGTCTGTTG	TGTGA-----	---------C	TCTGGTAA-C	TAGAGATCCC	TCAGACCCTT	--TTAGTCAG	T-GTGGAA--	AATCTCTAGC
B.KR.03.03KGS5.JQ316132	TAGTGTGTGC	CCGTCTGTTG	TGTGA-----	---------C	TCTGGTAA-C	TAGAGATCCC	TCAGACCTTT	---TAGTCAG	T-GTGGAA--	AATCTCTAGC
B.KR.03.03LSH1.JQ316127	TAGTGTGTGC	CCATCTGTTG	TGTGA-----	---------C	TCTGGTAA-C	TAGAGATCCC	TCAAACCCTT	--TTAGTCAG	T-GTGAAA--	AATCTCTAGC
B.KR.03.03YGS3.JQ316135	TAGTGTGTGC	CCGTCTGTTG	TGTGA-----	---------C	TCTGGTAA-C	TAGAGATCCC	TCAGACCCTT	--TTAGTCAG	T-GTGGAA--	AATCTCTAGC
B.KR.04.04CWS5.JQ316133	TAGTGTGTGC	CCGTCTGTTG	TGTGA-----	---------C	TCTGGTAA-C	TAGAGATCCC	TCAGACCCTT	--TTAGTCAG	T-GTGGAA--	AATCTCTAGC
B.KR.04.04KJS8.JQ316130	TAGTGTGTGC	CCGTCTGTTG	TGTGA-----	---------C	TCTGGTAA-C	TAGAGATCCC	TCAGACCCTT	--TTAGTCAG	T-GTGGAA--	AATCTCTAGC
B.KR.04.04KMH5.DQ295193	TAGTGTGTGC	CCGTCTGTTG	TGTGA-----	---------C	TCTGGTAA-C	TAGAGATCCC	TCAGACCAAT	--TTAGTCAG	T-GTGGAA--	AATCTCTAGC
B.KR.04.04KMK5.JQ316126	TAGTGTGTGC	CCGTCTGTTG	TGTGA-----	---------C	TCTGGTAA-C	TAGAGATCCC	TCAGACCCTT	--TTAGTCAG	T-GTGGAA--	AATCTCTAGC
B.KR.04.04LHS6.AY839827	TAGTGTGTGC	CCATCTGTTG	TGTGACTCCG	GTACTTAGAC	TCTGGTAA-C	TAGAGATCCC	TCAGAC-CTT	--TTAGTCAG	T-GTGGAA--	AATCTCTAGC
B.KR.04.04LSK7.DQ295192	TAGTGTGTGC	CCGTCTGTTG	TGTGA-----	---------C	TCTGGTAA-C	TAGAGATCCC	TCAGACCCTT	--TTAGTCAG	T-GTGGAA--	AATCTCTAGC
B.KR.04.04WK7_HIV_1_wk.DQ29519	TAGTGTGTGC	CCGTCTGTTG	TGTGA-----	---------C	TCTGGTAA-C	TAGAGATCCC	TCAGACCCTT	--TTAGTCAG	T-GTGGAA--	AATCTCTAGC
B.KR.05.05CSR3.DQ837381	TAGTGTGTGC	CCGTCTGTTG	TGTGA-----	---------C	TCTGGTAA-C	TAGAGATCCC	TCAGACAATT	--TTAGTCAG	T-GTGGAA--	AATCTCTAGC
B.KR.05.05YJN2.JQ316134	TAGTGTGTGC	CCGTCTGTTG	TGTGA-----	---------C	TCTGGTAA-C	TAGAGATCCC	TCAGACCCTT	--TTAGTCAG	T-GTGGAA--	AATCTCTAGC
B.KR.07.07KYY4.JQ341411	TAGTGTGTGC	CCGTCTGTTG	TGTGA-----	---------C	TCTGGTAA-C	TAGAGATCCC	TCAGACACTT	--TTAGTTAG	T-GTGGAA--	AATCTCTAGC
B.KR.99.99HYH2.JQ316129	TAGTGTGTGC	CCGTCTGTTG	TGTGA-----	---------C	TCTGGTAA-C	TAGAGATCCC	TCACACCATT	--TTAGTCAG	T-GTGGAA--	AATCTCTAGC
B.MM.99.mSTD101.AB097870	----------	----------	----------	----------	----------	----------	----------	----------	--------A-	AATCTCTAGC
B.NL.00.671_00T36.AY423387	TAGTGTGTGC	CCGTCTGTTA	TGTGA-----	---------C	TCTGGTAA-C	TAGAGATCCC	TCAGACCCTT	--TTAGTCAG	T-GTGGAA--	AATCTCTAGC
B.NL.86.3202A21_ACH3202A21.U34	TAGTGTGTGC	CCGTCTGTTG	TGTGA-----	---------C	TCTGGTAA-C	TAGAGATCCC	TCAGACCCTT	--TTAGTCAG	T-GAGGAA--	AATCTCTAGC
B.NL.96.H434_42_A1.AY970948	TAGTGTGTGC	CCGTCTGTTG	TGTGA-----	---------C	TCTGGTAA-C	TAGAGATCCC	TCAGACCCTT	--TTAGTCAG	T-ATGGAA--	AATCTCTAGC
B.PE.06.502_0491_wg5.JF320183	----------	----------	----------	----------	--TGGTAA-C	TAGAGATCCC	TCTGACCCTT	--TTAGTCAG	T-GTGGAA--	AATCTCTAGC
B.PE.06.502_0524_FL04.JF320008	----------	----------	----------	----------	----------	----------	----------	----------	----------	----------
B.PE.06.502_0648_FL02.JF320215	----------	----------	----------	----------	----------	----------	----------	----------	----------	----------
B.PE.06.502_0841_FL04.JF320208	----------	----------	----------	----------	--TGGTAA-C	TAGAGATCCC	TCAGACCCTT	--TTAGTCAG	T-GTGGAA--	AATCTCTAGC
B.PE.06.502_2794_FL05.JF320244	----------	----------	----------	----------	--TGGTAA-C	TAGAGATCCC	TCAGACCCTT	--AAAGTCAG	T-GTGGAA--	AATCTCTAGC
B.PE.07.502_0525_wg5.JF320191	--GTGTGTGC	CCGTCTGTTG	TGTGA-----	---------C	TCTGGTAA-C	TAGAGATCCC	TCAGACCCTT	--AAAGTCAA	T-GTGGAA--	AATCTCTAGC
B.PE.07.502_1047_wg5.JF320226	----------	----------	----------	----------	--GGGTAA-C	TAGAGATCCC	TCAGACCT--	--TTAAAGTC	A-GTGTGGAA	AATCTCTAGC
B.PE.07.502_1399_wg4.JF320013	----------	----------	----------	----------	--TGGTAA-C	TAGAGATCCC	TCAGACCACT	--TTAGTCAG	T-GTGGAA--	AATCTCTAGC
B.PE.07.502_2254_FL6.JF320018	----------	----------	----------	----------	--TGGTAG-C	TAGAGATCCC	TCAGACCCTT	--TTAGTCAG	T-GTGGAA--	AATCTCTAGC
B.PE.07.502_2349_wg2.JF320028	----------	----------	----------	----------	--TGGTAA-C	TAGAGATCCC	TCAGACCCTT	--AAAGTCAG	T-GTGGAA--	AATCTCTAGT
B.PE.07.502_2622_wg1.JF320189	----------	----------	----------	----------	--TGGTAA-C	TAGAGATCCC	TCAGACCATT	--TTAGTCCG	A-GAGAA---	AATCTCTAGC
B.PE.07.502_2649_wg8.JF320019	----------	----------	----------	----------	---------C	TAGAGATCCC	TCAGACCCTT	--TTAGTCAG	T-GTGGAA--	AATCTCTAGC
B.RU.04.04RU128005.AY682547	TAGTGTGTGC	CCGTCTGTTG	TGTGA-----	---------C	TCTGGTAA-C	TAGAGATCCC	TCAGACCCTT	--TTAGTCAG	T-GTRGAA--	AATCTCTAGC
B.RU.04.04RU129005.AY751406	TAGTGTGTGC	CCGTCTGTTG	TGTGA-----	---------C	TCTGGTAA-C	TAGAGATCCC	TCAGACCATT	--TTAGTCAG	T-GTGGAA--	AATCTCTAGC
B.RU.04.04RU139089.AY751407	----------	----------	----------	----------	----------	----------	----------	----------	----------	----------
B.RU.04.04RU139095.AY819715	----------	----------	----------	----------	----------	----------	----------	----------	----------	----------
B.RU.09.09RU4457.JX500709	TAGTGTGTGC	CCGTCTGTTG	TGTGA-----	---------C	TCTGGTAA-C	TAGAGATCCC	TCAGACCATT	--TAAGTCAG	T-GTGGAA--	AATCTCTAGC
B.RU.10.10RU6629.JX500707	TAGTGTGTGC	CCGTCTGTTG	TGTGA-----	---------C	TCTGGTAA-C	TAGAGATCCC	TCAGACCATT	--TTAGTCAG	T-GTGGAA--	AATCTCTAGC
B.RU.11.11RU21n.JX500708	TAGTGTGTGC	CCGTCTGTTG	TGTGA-----	---------C	TCTGGTAA-C	TAGAGATCCC	TCAGACTCTT	--TTAGTCAG	T-GTGGAA--	AATCTCTAGC
B.TH.06.AA010a_WG3.JX446800	----------	----------	----------	----------	--TGGTAA-C	TAGAGATCCC	TCAGACCCTT	--AAAGTCAG	T-GTGGAA--	AATCTCTAGC
B.TH.07.AA040a_WG11.JX447156	----------	----------	----------	----------	----------	----------	----------	----------	----------	----------
B.TH.90.BK132.AY173951	----------	----------	----------	----------	----------	----------	----------	----------	----------	----------
B.TW.94.TWCYS_LM49.AF086817	TAGTGTGTGC	CCGTCTGTTG	TGTGA-----	---------C	TCTGGTAA-C	TAGAGATCCC	TCAGACCATT	--TTAGTCAG	T-GTGGAA--	AATCTCTAGC
B.US.00.14294_1.DQ853436	TAGTGTGTGC	CCGTCTGTTG	TGTGA-----	---------C	TCTGGTAG-C	TAGAGATCCC	TCAGATCCTT	--TTAGTTAG	C-GTAGAA--	AATCTCTAGC
B.US.00.ES1_20.EF363123	TAGTGTGTGC	CCGTCTGTTG	TGTGA-----	---------C	TCTGGTAA-C	TAGAGATCCC	TCAGACCCTT	--TTAGTCAG	T-GTGGAA--	AATCTCTAGC
B.US.00.RHPA_TF1.JN944917	TAGTGTGTGC	CCGTCTGTTG	TGTGA-----	---------C	TCTGGTAA-C	TAGAGATCCC	TCAGACCTTT	---TAGTCAG	T-GTGGAA--	AATCTCTAGC
B.US.00.THRO_TF1.JN944930	TAGTGTGTGC	CCGTCTGTTG	TGTGA-----	---------C	TCTGGTAT-C	TAGAGATCCC	TCAGACCCTA	--TTAGTCAG	T-GTGGAA--	AATCTCTAGC
B.US.00.WITO_flB3.FJ496169	----------	----------	----------	----------	--TGGTAA-C	TAGAGATCCC	TCAGACCATT	--TTAGTCAG	T-GTGGAA--	AATCTCTAGC
B.US.01.REJO_TF1.JN944911	TAGTGTGTGC	CCGTCTGTTG	TGTGA-----	---------C	TCTGGTAA-C	TAGAGATCCC	TCAGACTCTT	--TTAGTCAG	T-GTGGAA--	AATCTCTAGC
B.US.01.TRJO_flA1.FJ496151	----------	----------	----------	----------	--TGGTAA-C	TAGAGATCCC	TCAGATCATT	--TTAGTCAG	T-GTGGAA--	AATCTCTAGC
B.US.02.04013226_2_flH11.FJ496	----------	-------TGG	TAATA-----	---------C	TCTGGTAA-C	TAGAGATCCC	TCAGACCTTT	---TAGTCAG	T-GTGGAA--	AATCTCTAGC
B.US.04.ES10_53.EF363127	TAGTGTGTGC	CCGTCTGTTG	TGTGA-----	---------C	TCTGGTAA-C	TAGAGATCCC	TCAGACCCTT	--TTAGTCAG	T-GTGGAA--	AATCTCTAGC
B.US.04.ES4_24.EF363124	TAGTGTGTGC	CCGTCTGTTG	TGTGA-----	---------C	TCTGGTAA-C	TAGAGATCCC	TCAGACCCTT	--TTAGTCAG	T-GTGGAA--	AATCTCTAGC
B.US.04.ES8_43.EF363126	TAGTGTGTGC	CCGTCTGTTG	TGTGA-----	---------C	TCTGGTAA-C	TAGAGATCCC	TCAGACCATT	--TTAGTCAG	T-GTGGAA--	AATCTCTAGC
B.US.04.SAMI_WGA1.EU547186	----------	----------	----------	----------	--TGGTAA-C	TAGAGATCCC	TCAGACCTTT	--TTAGTCAG	T-GTGGAA--	AATCTCTAGC
B.US.04.USPI71101EI7y04051pcWG	----------	----------	----------	----------	--TGGTAA-C	TAGAGATCCC	TCAGACCCTT	--TTAGTTAG	T-GTGGAA--	AATCTCTAGC
B.US.04.USPI83747EI6y04121pcWG	--GTGTGTGC	CCGTCTGTTG	TGTGA-----	---------C	TCTGGTAA-C	TAGAGATCCC	TCAGACCTTT	---TAGTCAG	T-GTGGAAA-	AATCTCTAGC
B.US.05.04013396_0_flE6.FJ4960	----------	----------	----------	----------	--TGGTAA-C	TAGAGATCCC	TCAGACCCTT	--TTAGTCAG	T-GTGGAA--	AATCTCTAGC
B.US.05.502_0223_wg1.JF320059	----------	----------	----------	----------	----------	----------	----------	--------AG	T-GTGGAA--	AATCTCTAGC
B.US.05.502_1400_FL02.JF320043	----------	----------	----------	----------	----------	----------	----------	----------	-TGTGGAA--	AATCTCTAGC
B.US.05.502_2136_FL02.JF320185	TAGTGTGTGC	CCGTCTGTTG	TGTGA-----	---------C	TCTGGTAA-C	TAGAGATCCC	TCAGACCATT	--TTAGTCAG	T-GTGGAA--	AATCTCTAGC
B.US.05.502_2495_wg02.JF320054	----------	----------	----------	----------	----------	----------	----------	----------	---TGGAAA-	AATCTCTAGC
B.US.05.CR0307R.FJ469717	----------	----------	----------	----------	----------	----------	----------	----------	----------	----TCTAGC
B.US.05.USPI38417EI33y05051pcW	----------	----------	----------	----------	--TGGTAA-C	TAGAGATCCC	TCAGACCATC	--TTAGTCAG	T-GTGGAA--	AATCTCTAGC
B.US.05.USPI88403EI14y05121pcW	----------	----------	----------	----------	--TGGTAA-C	TAGAGATCCC	TCAGAC--TC	--TAAGTCAG	T-GTGGAAA-	AATCTCTAGC
B.US.06.502_0341_FL05.JF320003	----------	----------	----------	----------	--TGGTAA-C	TAGAGATCCC	TCAGACCCTT	--TTAGTCAG	T-GTGGAA--	AATCTCTAGC
B.US.06.502_1046_FL04.JF320564	--GTGTGTGC	CCGTCTGTTG	TGTGA-----	---------C	TCTGGTAA-C	TAGAGATCCC	TCAGACCATC	--TTAGACAG	T-GTAGAA--	AATCTCTAGC
B.US.06.502_1174_FL09.JF320053	----------	----------	----------	----------	--TGGTAA-C	TAGAGATCCC	TCAGACCCTT	--TTAGTCAG	T-GTGGAA--	AATCTCTAGC
B.US.06.502_1211_FL01.JF320151	----------	----------	----------	----------	--TGGTAA-C	TAGAGATCCC	TCAGACCACC	--TTAGTCAG	T-GGGAAA--	AATCTCTAGC
B.US.06.502_1619_FL06.JF320126	----------	----------	----------	----------	--TGGTAA-C	TAGAGATCCC	TCAGACCATT	--TTAGTCAG	T--TGGAA--	AATCTCTAGC
B.US.06.502_2667_FL03.JF320145	----------	----------	----------	----------	----------	----------	----------	--------GT	--GTGGAA--	AATCTCTAGC
B.US.06.CH106_TF1.JN944897	TAGTGTGTGC	CCGTCTGTTG	TGTGA-----	---------C	TCTGGTAA-C	TAGAGATCCC	TCAGACCACT	--TTAGTCCG	G-GTGGAA--	AATCTCTAGC
B.US.06.CH40E_flA1.FJ495818	----------	----------	----------	----------	--TGGTAA-C	TAGAGATCCC	TCAGACCCTT	--TTAGTCAG	T-GTGGAA--	AATCTCTAGC
B.US.06.CH58E_flA9.FJ495941	----------	----------	----------	----------	--TGGTAA-C	TAGAGATCCC	TCAGACCCTT	--TTAGTCAG	T-GTGGAA--	AATCTCTAGC

```
B.US.06.CH77E_flA1.FJ496000          ---------- ---------- ---------- ---------- --TGGTAA-C TAGAGATCCC TCAGACCTTT ---TAGTCAG T-GTGGAA-- AATCTCTAGC
B.US.07.502_0364_wg2.JF320563        ---------- ---------- ---------- ---------- ----GTAA-C TAGAGATCCC TCAGACCATT --TTAGTCAG T-GTGGAA-- AATCTCTAGC
B.US.07.502_1115_wg1.JF320045        ---------- ---------- ---------- ---------- --TGGTAG-C TAGAGATCCC TCAGACCCTT --TTAGTCAG T-GTGGAA-- AATCTCTAGC
B.US.07.502_1478_wg4.JF320150        ---------C CCGTCTGTTG TGTGA----- ---------C TCTGGTAA-C TAGAGATCCC TCAGACCCTT --TTAGTCAG T-GTGGAA-- AATCTCTAGC
B.US.07.502_1897_wg6.JF320182        TAGTGTGTGC CCGTCTGTTG TGTGA----- ---------C TCTGGTAA-C TAGAGATCCC TCAGACCAAT --TTAGTCAG G-GTGGAA-- AATCTCTAGC
B.US.07.502_2289_05.JF320197         ---------- ---------- ---------- ---------- --TGGTAA-C TAGAGATCCC TCAGACCCTT --TTAGTCAG T-GTGGAA-- AATCTCTAGC
B.US.09.C1P.GU733713                 TAGTGTGTGC CCGTCTGTTG TGTGA----- ---------C TCTGGTAA-C TAGAGATCCC TCAGACCATT --TTAGTCAG T-GTGGAA-- AATCTCTAGC
B.US.09.DEMB09US002.JX140657         ---------- ---------- ---------- ---------- ---------- ---------- ---------- ---------- ---------- ----------
B.US.10.CP1.JN397365                 TAGTGTGTGC CCGTCTGTTG TGTGA----- ---------C TCTGGTAA-C TAGAGATCCC TCAGACCCTT ---TAGTCAG T-GTGGAA-- AATCTCTAGC
B.US.10.VC1.JN397364                 TAGTGTGTGC CCGTCTGTTG TGTGA----- ---------C TCTGGTAA-C TAGAGATCCC TCAGACCCTT ---TAGTCAG T-GTGGAA-- AATCTCTAGC
B.US.11.ES38.JN397362                TAGTGTGTGC CCGTCTGTTG TTTGA----- ---------C TCTGGTAA-C TAGAGATCCC TCAGACTCTC --GTAGTCAG T-GTGAAA-- AATCTCTAGC
B.US.83.5018_83.AY835777             TAGTGTGTGC CCGTCTGTTG TGTGA----- ---------C TCTGGTAA-C TAGAGATCCC TCAGACCCTT --TTAGTCAG T-GTGGAA-- AATCTCTAGC
B.US.83.5157_83.AY835781             TAGTGTGTGC CCGTCTGTTG TGTGA----- ---------C TCTGGTAA-C TAGAGATCCC TCAGACCCTT --TTAGTCAG T-GTGGAA-- AATCTCTAGC
B.US.83.RF_HAT3.M17451               TAGTGTGTGC CCGTCTGTTG TGTGA----- ---------C TCTGGTAA-C TAGAGATCCC TCAGACCTCT --TTAGTCAG T-GTGGAA-- AATCTCTAGC
B.US.83.SF2_LAV2_ARV2.K02007         TAGTGTGTGC CCGTCTGTTG TGTGA----- ---------C TCTGGTAA-C TAGAGATCCC TCAGACCCTT --TTAGTCAG T-GTGGAAA- AATCTCTAGC
B.US.84.5019_84.AY835779             TAGTGTGTGC CCGTCTGTTG TGTGA----- ---------C TCTGGTAA-C TAGAGATCCC TCAGACCCTT --TTAGTCAG T-GTGGAA-- AATCTCTAGC
B.US.84.MNCG_MN.M17449               TAGTGTGTGC CCGTCTGTTA TGTGA----- ---------C TCTGGTAG-C TAGAGATCCC TCAGATCCTT --TTAGGCAG T-GTGGAA-- AATCTCTAGC
B.US.84.NY5CG.M38431                 TAGTGTGTGC CCGTCTGTTG TGTGA----- ---------C TCTGGTAA-C TAGAGATCCC TCAGACCCTT --TTAGTCAG T-GTGGAA-- AATCTCTAGC
B.US.84.SF33.AY352275                TAGTGTGTGC CCGTCTGTTG TGTGA----- ---------C TCTGGTAT-C TAGAGATCCC TCAGACCCTT --TTAGTCAG T-GTGGAA-- AATCTCTAGC
B.US.85.5077_85.AY835769             TAGTGTGTGC CCGTCTGTTG TGTGA----- ---------C TCTGGTAA-C TAGAGATCCC TCAGACCCTT --TTAGTCAG T-GTGGAA-- AATCTCTAGC
B.US.85.Ba_L.AB221005                TAGTGTGTGC CCGTCTGTTG TGTGA----- ---------C TCTGGTAA-C TAGAGATCCC TCAGACCCTT --TTAGTCAG T-GTGGAA-- AATCTCTAGC
B.US.86.5084_86.AY835775             TAGTGTGTGC CCGTCTGTTG TGTGA----- ---------C TCTGGTAA-C TAGAGATCCC TCAGACCCTT --TTAGTCAG T-GTGGAA-- AATCTCTAGC
B.US.86.5096_86.AY835749             TAGTGTGTGC CCGTCTGTTG TGTGA----- ---------C TCTGGTAA-C TAGAGATCCC TCAGACCCTT --TTAGTCAG T-GTGGAA-- AATCTCTAGC
B.US.86.5127_86.AY835774             TAGTGTGTGC CCGTCTGTTG TGTGA----- ---------C TCTGGTAA-C TAGAGATCCC TCAGACCCTT --TTAGTCAG T-GTGGAA-- AATCTCTAGC
B.US.86.AD87_ADA.AF004394            TAGTGTGTGC CCGTCTGTTG TGTGA----- ---------C TCTGGTAA-C TAGAGATCCC TCAGACCCTT --TTAGTCAG T-GTGGAA-- AATCTCTAGC
B.US.86.JRFL_JR_FL.U63632            ---------- ---------- ---------- ---------- ---------- ---------- ---------- ---------- ---------- ----------
B.US.86.YU_2.M93258                  TAGTGTGTGC CCGTCTGTTG TGTGA----- ---------C TCTGGTAA-C TAGAGATCCC TCAGACCCTT --TTAGTCAG T-GTGGAA-- AATCTCTAGC
B.US.87.5113_87.AY835758             TAGTGTGTGC CCGTCTGTTG TGTGA----- ---------C TCTGGTAA-C TAGAGATCCC TCAGACCCTT --TTAGTCAG T-GTGGAA-- AATCTCTAGC
B.US.87.BC_BCSG3.L02317              TAGTGTGTGC CCGTCTGTTG TGTGA----- ---------C TCTGGTAG-C TAGAGATCCC TCATACCCTT --TTAGTCAG T-GTGGAA-- AATCTCTAGC
B.US.88.5160_88.AY835763             TAGTGTGTGC CCGTCTGTTG TGTGA----- ---------C TCTGGTAA-C TAGAGATCCC TCAGACCCTT --TTAGTCAG T-GTGGAA-- AATCTCTAGC
B.US.88.WR27.AF286365                TAGTGTGTGC CCGTCTGTTG TGTGA----- ---------C TCTGGTAA-C TAGAGATCCC TCAGACCCTT --TTAGTCAG T-GTGGAA-- AATCTCTAGC
B.US.89.P896_89_6.U39362             TAGTGTGTGC CCGTCTGTTG TGTGA----- ---------C TCTGGTAG-C TAGAGATCCC TCAGACCATC --CTAGTTAG T-GTAGAA-- AATCTCTAGC
B.US.90.US1.AY173952                 ---------- ---------- ---------- ---------- ---------- ---------- ---------- ---------- ---------- ----------
B.US.90.US2.AY173953                 ---------- ---------- ---------- ---------- ---------- ---------- ---------- ---------- ---------- ----------
B.US.90.US3.AY173954                 ---------- ---------- ---------- ---------- ---------- ---------- ---------- ---------- ---------- ----------
B.US.90.US4.AY173955                 ---------- ---------- ---------- ---------- ---------- ---------- ---------- ---------- ---------- ----------
B.US.90.WCIPR.U69591                 TAGTGTGTGC CCGTCTGTTG TGTGA----- ---------C TCTGGTAA-C TAGAGATCCC TCAGACCCTT --TTAGTCAG T-GTGGAA-- AATCTCTAGC
B.US.90.WEAU160_GHOSH.U21135         TAGTGTGTGC CCGTCTGTTG TGTGA----- ---------C TCTGGTAA-C TAGAGATCCC TCAGACCATT --TTAGTCAG T-GTGGAA-- AATCTCTAGC
B.US.91.5048_91.AY835761             TAGTGTGTGC CCGTCTGTTG TGTGA----- ---------C TCTGGTAA-C TAGAGATCCC TCAGACCCTT --TTAGTCAG T-GTGGAA-- AATCTCTAGC
B.US.91.DH12_3.AF069140              TAGTGTGTGC CCGTCTGTTG TGTGA----- ---------C TCTGGTAA-C TAGAGATCCC TCAGACCATT --TTAGTCAG T-GTGGAA-- AATCTCTAGC
B.US.91.SUMA_flC5.FJ496145           ---------- ---------- ---------- ---------- --TGGTAA-C TAGAGATCCC TCAGACCAA- --TTAGTCAG T-GTGGAA-- AATCTCTAGC
B.US.93.WCD32P0793.DQ487188          TAGTGTGTGC CCGTCTGTTG TGTGA----- ---------C TCTGGTAG-C TAGAGATCCC TCAGACCATA ----AGTCAG T-GTGGAA-- AATCTCTAGC
B.US.93.WCM32P0793.DQ487190          TAGTGTGTGC CCGTCTGTTG TGTGA----- ---------C TCTGGTAG-C TAGAGATCCC TCAGACCCTT ----AGTCAG T-GTGGAA-- AATCTCTAGC
B.US.94.5082_94.AY835773             TAGTGTGTGC CCGTCTGTTG TGTGA----- ---------C TCTGGTAA-C TAGAGATCCC TCAGACCCTT --TTAGTCAG T-GTGGAA-- AATCTCTAGC
B.US.95.5073_95.AY835768             TAGTGTGTGC CCGTCTGTTG TGTGA----- ---------C TCTGGTAA-C TAGAGATCCC TCAGACCCTT --TTAGTCAG T-GTGGAA-- AATCTCTAGC
B.US.95.USPI90770EI72y95091pcW       ---------- ---------- ---------- ---------- --TGGTAA-C TAGAGATCCC TCAGACCTTT --ATAGTCAG T-GTGGAA-- AATCTCTAGC
B.US.96.5155_96.AY835753             TAGTGTGTGC CCGTCTGTTG TGTGA----- ---------C TCTGGTAA-C TAGAGATCCC TCAGACCCTT --TTAGTCAG T-GTGGAA-- AATCTCTAGC
B.US.96.USPI55751EI32y96071pcW       ---------- ---------- ---------- ---------- --TGGTAA-C TAGAGATCCC TCAGACCCTT ---TAGTCAG T-GTGGAA-- AATCTCTAGC
B.US.97.ARES2.AB078005               TAGTGTGTGC CCGTCTGTTG TGTGA----- ---------C TCTGGTAA-C TAGAGATCCC TCAGACCCTT ---TAGTCAG T-GTGGAA-- AATCTCTAGC
B.US.98.15384_1.DQ853463             TAGTGTGTGC CCGTCTGTTG TGTGA----- ---------C TCTGGTAG-C TAGAGATCCC TCAGACCCTT --TTAGTTAG C-GTAGAA-- AATCTCTAGC
B.US.98.98USHVTN1925c1.AY56010       ---------- ---------- ---------- ---------- --TGGTAA-C TAGAGATCCC TCAGACCCTT --TTAGTCAG T-GTGGAA-- AATCTCTAGC
B.US.98.98USHVTN3605c9.AY56010       ---------- ---------- ---------- ---------- --TGGTAA-C TAGAGATCCC TCAGACCCTT --TTAGTCAG T-GTGGAA-- AATCTCTAGC
B.US.98.98USHVTN8229c6.AY56010       ---------- ---------- ---------- ---------- --TGGTAA-C TAGAGATCCC TCAGACC-TT --TTAGTCAG T-ATAGAAA- AATCTCTAGC
B.US.98.98USHVTN941c1.AY560110       ---------- ---------- ---------- ---------- --TGGTAA-C TAGAGATCCC TCAGACCATT --TTAGTCAG T-ATGGAA-- AATCTCTAGC
B.US.98.WC3_0498_4.EF175212          TAGTGTGTGC CCGTCTGTTG TGTGA----- ---------C TCTGGTAA-C TAGAGATCCC TCAGACTCTT --TTAGTCAG T-GTGAAA-- AATCTCTAGC
B.US.x.CR0059T.FJ469694              ---------- ---------- ---------- ---------- ---------- ---------- ---------- ---------- ---------- ----TCTAGC
B.US.x.sample_C_BID_D617.JX503       ---------- ---------- ---------- ---------- ---------- ---------- ---------- ---------- ---------- ---GGGGTGT
B.VE.10.DEMB10VE001.JX140659         ---------- ---------- ---------- ---------- ---------- ---------- ---------- ---------- ---------- ----------
B.ZA.03.03ZAPS045MB2.DQ396398        ---------- ---TCTGTTG TGTGA----- ---------C TCTGGTAA-C TAGAGATCCC TCAGACCTTT ---AAAGTCA G-TAGAAA-- AATCTCTAGC
C.BR.02.02BR2022.JN692434            TAGTGTGTGC CCGTCTGTTG TGTGA----- ---------C TCTGGTAA-C TAGAGATCCC TCAGACACCT --ATTGTCAG T-GTGGAA-- AATCTCTAGC
C.BR.04.04BR013.AY727522             TAGTGTGTGC CCGTCTGTTG TGTGA----- ---------C TCTGGTAG-C TAGAGATCCC TCAGATACCT --TTTGTCAG T-GTGGAA-- AATCTCTAGC
C.BR.04.04BR021.AY727523             TAGTGTGTGC CCGTCTGTTG TGTGA----- ---------C TCTGGTAA-C TAGAGATCCC TCAGACGCCT --TTCGTCAG T-GTGGAA-- AATCTCTAGC
C.BR.04.04BR038.AY727524             TAGTGTGTGC CCGTCTGTTG TGTGA----- ---------C TCTGGTAG-C TAGAGATCCC TCAGATACTT --TTTGTCCG T-GTGGAA-- AATCTCTAGC
C.BR.04.04BR073.AY727525             TAGTGTGTGC CCGTCTGTTG TGTGA----- ---------C TCTGGTAA-C TAGAGATCCC TCAGACACAT --TTTGTCCG T-GTGGAA-- AATCTCTAGC
C.BR.07.DEMC07BR003.JX140663         ---------- ---------- ---------- ---------- ---------- ---------- ---------- ---------- ---------- ----------
C.BR.92.BR025_d.U52953               ---------- ---------- ---------- ---------- ---------- ---------- ---------- ---------- ---------- ----------
C.BR.98.98BR004.AF286228             ---------- ---------- ---------- ---------- ---------- ---------- ---------- ---------- ---------- ----------
C.BW.00.00BW07621.AF443088           ---------- ---------- ---------- ---------- ---------- ---------- ---------- ---------- ---------- --TCTCTAGC
C.BW.00.00BW076820.AF443089          ---------- ---------- ---------- ---------- ---------- ---------- ---------- ---------- ---------- --TCTCTAGC
C.BW.00.00BW087421.AF443090          ---------- ---------- ---------- ---------- ---------- ---------- ---------- ---------- ---------- --TCTCTAGC
C.BW.00.00BW147127.AF443091          ---------- ---------- ---------- ---------- ---------- ---------- ---------- ---------- ---------- --TCTCTAGC
C.BW.00.00BW16162.AF443092           ---------- ---------- ---------- ---------- ---------- ---------- ---------- ---------- ---------- --TCTCTAGC
C.BW.00.00BW1686.AF443093            ---------- ---------- ---------- ---------- ---------- ---------- ---------- ---------- ---------- --TCTCTAGC
C.BW.00.00BW17593.AF443094           ---------- ---------- ---------- ---------- ---------- ---------- ---------- ---------- ---------- --TCTCTAGC
C.BW.00.00BW17732.AF443095           ---------- ---------- ---------- ---------- ---------- ---------- ---------- ---------- ---------- TCTCTAGCG-
C.BW.00.00BW17835.AF443096           ---------- ---------- ---------- ---------- ---------- ---------- ---------- ---------- ---------- --TCTCTAGC
C.BW.00.00BW17956.AF443097           ---------- ---------- ---------- ---------- ---------- ---------- ---------- ---------- ---------- --TCTCTAGC
C.BW.00.00BW18113.AF443098           ---------- ---------- ---------- ---------- ---------- ---------- ---------- ---------- ---------- --TCTCTAGC
C.BW.00.00BW18595.AF443099           ---------- ---------- ---------- ---------- ---------- ---------- ---------- ---------- ---------- --TCTCTAGC
C.BW.00.00BW18802.AF443100           ---------- ---------- ---------- ---------- ---------- ---------- ---------- ---------- ---------- --TCTCTAGC
C.BW.00.00BW192113.AF443101          ---------- ---------- ---------- ---------- ---------- ---------- ---------- ---------- ---------- --TCTCTAGC
C.BW.00.00BW20361.AF443102           ---------- ---------- ---------- ---------- ---------- ---------- ---------- ---------- ---------- --TCTCTAGC
C.BW.00.00BW20636.AF443103           ---------- ---------- ---------- ---------- ---------- ---------- ---------- ---------- ---------- --TCTCTAGC
C.BW.00.00BW20872.AF443104           ---------- ---------- ---------- ---------- ---------- ---------- ---------- ---------- ---------- --TCTCTAGC
C.BW.00.00BW2127214.AF443105         ---------- ---------- ---------- ---------- ---------- ---------- ---------- ---------- ---------- --TCTCTAGC
C.BW.00.00BW22767.AF443107           ---------- ---------- ---------- ---------- ---------- ---------- ---------- ---------- ---------- --TCTCTAGC
C.BW.00.00BW38193.AF443108           ---------- ---------- ---------- ---------- ---------- ---------- ---------- ---------- ---------- --TCTCTAGC
C.BW.00.00BW38428.AF443109           ---------- ---------- ---------- ---------- ---------- ---------- ---------- ---------- ---------- --TCTCTAGC
C.BW.00.00BW38713.AF443110           ---------- ---------- ---------- ---------- ---------- ---------- ---------- ---------- ---------- --TCTCTAGC
C.BW.00.00BW3876_9.AF443111          ---------- ---------- ---------- ---------- ---------- ---------- ---------- ---------- ---------- --TCTCTAGC
C.BW.00.00BW3886_8.AF443112          ---------- ---------- ---------- ---------- ---------- ---------- ---------- ---------- ---------- --TCTCTAGC
C.BW.00.00BW3891_6.AF443113          ---------- ---------- ---------- ---------- ---------- ---------- ---------- ---------- ---------- --TCTCTAGC
C.BW.00.00BW3970_2.AF443114          ---------- ---------- ---------- ---------- ---------- ---------- ---------- ---------- ---------- --TCTCTAGC
C.BW.00.00BW5031_1.AF443115          ---------- ---------- ---------- ---------- ---------- ---------- ---------- ---------- ---------- TCTCTAGCAG
C.BW.96.96BW01B03.AF110959           TAGTGTGTGC CCGTCTGTTG TGTGA----- ---------C TCTGGTAA-C TAAAGATCCC TCAAACCCTT ---TTGGTTG T-GTGGAA-- AATCTCTAGC
C.BW.96.96BW0402.AF110962            TAGTGTGTGC CCGTCTGTTG TGTGA----- ---------C TCTGGTAA-C TAGAGATCCC TCAGACCCTT --CGCGGTAG T-GTGGAA-- AATCTCTAGC
C.BW.96.96BW0502.AF110967            TAGTGTGTGC CCGTCTGATG TGTGA----- ---------C TCTGGTAA-C TAGAGATCCC TCAGAACCTT ---TTGGTAG T-GTGGAA-- AATCTCTAGC
C.BW.96.96BW06.AF290028              TAGTGTGTGC CCATCTGTTA TATGG--A-- ---------G TCTGGTAA-C TAGAGATCCC TCAGGCCCTT ---TTGGTAG T-GAGGAA-- AATCTCTAGC
C.BW.96.96BW1104.AF110969            ---------- ---------- ---------- ---------- ---------- ---------- ---------- -----GGTAG T-GTGGAA-- AATCTCTAGC
C.BW.96.96BW1210.AF110972            ---------- ---------- ---------- ---------- ---------- ---------- ---------- -----GGTAG T-GTGGAA-- AATTTCTAGC
C.BW.96.96BW15C02.AF110974           TAGTGTGTGC CCGTCTGTTG TGTGA----- ---------C TCTGGTAA-C TAAAGATCCC TCAGACCCTT --TTTGGTAT T-GTGGAA-- AATCTCTAGC
C.BW.96.96BW16B01.AF110976           TAGTGTGTGC CCGTCTGTTG TGTGA----- ---------C TCTGGTAA-C TAGAGATCCC TCAGACCTT- --TTTGGTAG T-GTGGAA-- AATCTCTAGC
C.BW.96.96BW17.AF110980              ---------- ---------- ---------- ---------- ---------- ---------- ---------- ---------- ---------- --TCTCTAGC
C.BW.96.96BWM032.AF443075            ---------- ---------- ---------- ---------- ---------- ---------- ---------- ---------- ---------- --TCTCTAGC
C.BW.96.96BWMO1_5.AF443074           ---------- ---------- ---------- ---------- ---------- ---------- ---------- ---------- ---------- --TCTCTAGC
```

```
C.BW.98.98BWMC122.AF443076      ---------- ---------- ---------- ---------- ---------- ---------- ---------- ---------- ---------- --TCTCTAGC
C.BW.98.98BWMC134.AF443077      ---------- ---------- ---------- ---------- ---------- ---------- ---------- ---------- ---------- --TCTCTAGC
C.BW.98.98BWMC14A3.AF443078     ---------- ---------- ---------- ---------- ---------- ---------- ---------- ---------- ---------- --TCTCTAGC
C.BW.98.98BWMO1410.AF443079     ---------- ---------- ---------- ---------- ---------- ---------- ---------- ---------- ---------- --TCTCTAGC
C.BW.98.98BWMO18D5.AF443080     ---------- ---------- ---------- ---------- ---------- ---------- ---------- ---------- ---------- --TCTCTAGC
C.BW.98.98BWMO36A5.AF443081     ---------- ---------- ---------- ---------- ---------- ---------- ---------- ---------- ---------- --TCTCTAGC
C.BW.98.98BWMO37D5.AF443082     ---------- ---------- ---------- ---------- ---------- ---------- ---------- ---------- ---------- --TCTCTAGC
C.BW.99.99BW393212.AF443083     ---------- ---------- ---------- ---------- ---------- ---------- ---------- ---------- ---------- --TCTCTAGC
C.BW.99.99BW46424.AF443084      ---------- ---------- ---------- ---------- ---------- ---------- ---------- ---------- ---------- --TCTCTAGC
C.BW.99.99BW4745.AF443085       ---------- ---------- ---------- ---------- ---------- ---------- ---------- ---------- ---------- --TCTCTAGC
C.BW.99.99BW47547.AF443086      ---------- ---------- ---------- ---------- ---------- ---------- ---------- ---------- ---------- --TCTTTAGC
C.BW.99.99BWMC168.AF443087      ---------- ---------- ---------- ---------- ---------- ---------- ---------- ---------- ---------- --TCTCTAGC
C.DJ.x.DJ259.AB485643           TAGTGTGTGC CCGTCTGTTG TGTGA----- ---------C TCTGGTAA-C TAGAGATCCC TCAGACTTCT --TTTGTCCG T-GTGGAA-- AATCTCTAGC
C.DK.01.CTL_015.EF514713        ---------- ---------- ---------- ---------- ---------- ---------- ---------- ---------- ---------- ---------T
C.ES.06.X1936.EU786673          TAGTGTGTGC CCGTCTGTTG TGTGA----- ---------C TCTGGTAA-C TAGAGATCCC TCAGACCCTT ---GTGGTAG T-GTGGAA-- AATCTCTAGC
C.ES.07.X2118_2.EU884500        -----TGTGC CCGTCTGTTG TGTGA----- ---------C TCTGGTAA-C TAGAGATCCC TCAGACCCTT ---TTGGTTG T-GTGGAA-- AATCTCTAGC
C.ES.08.X2363_2.EU786681        ---------- ----CTGTTG TGTGA----- ---------C TCTGGTAG-C TAGAGATCCC TCAGATCAAT --TTTGCTCT T-GTGGAA-- AATCTCTAGC
C.ET.86.ETH2220.U46016          ---------- ---------- ---------- ---------- ---------- ---------- ---------- ---------- --------A- AATCTCTAGC
C.IL.98.98IS002.AF286233        ---------- ---------- ---------- ---------- ---------- ---------- ---------- ---------- ---------- ----------
C.IN.03.D24.EF469243            CAGTGTGTGC CCGTCTGTTG TGTGA----- ---------C TCTGGTAT-C TAGAGATCCC TCAGACCCTT ---TTGGTAG T-GTGGAA-- AATCTCTAGC
C.IN.93.93IN101.AB023804        CAGTGTGTGC CCGTCTATTG TGTGA----- ---------C TCTGGTAA-C TAGAGATCCC TCAGACCCTT ---TTGGTAG T-GTGGAA-- AATCTCTAGC
C.IN.93.93IN904.AF067157        ---------- ---------- ---------- ---------- ---------- ---------- ---------- ---------- --------A- AATCTCTAGC
C.IN.93.93IN999.AF067154        ---------- ---------- ---------- ---------- ---------- ---------- ---------- ---------- --------A- AATCTCTAGC
C.IN.94.94IN11246.AF067159      ---------- ---------- ---------- ---------- ---------- ---------- ---------- ---------- --------A- AATCTCTAGC
C.IN.94.94IN476.AF286223        ---------- ---------- ---------- ---------- ---------- ---------- ---------- ---------- ---------- ----------
C.IN.95.95IN21068.AF067155      ---------- ---------- ---------- ---------- ---------- ---------- ---------- ---------- --------A- AATCTCTAGC
C.IN.98.98IN012.AF286231        ---------- ---------- ---------- ---------- ---------- ---------- ---------- ---------- ---------- ----------
C.IN.98.98IN022.AF286232        ---------- ---------- ---------- ---------- ---------- ---------- ---------- ---------- ---------- ----------
C.IN.99.01IN565_10.AY049708     ---------- ---------- ---------- ---------- ---------- ---------- ---------- ---------- -----AAA-- AATCTCTAGC
C.IN.x.VB39.EF694033            ---------- ---------- ---------- ---------- ---------- ---------- ---------- ---------- ---------- ---------C
C.IN.x.VB49.EF694036            ---------- ---------- ---------- ---------- ---------- ---------- ---------- ---------- --------A- AATCTCTAGC
C.MM.99.mIDU101_3.AB097871      ---------- ---------- ---------- ---------- ---------- ---------- ---------- ---------- --------A- AATCTCTAGC
C.TZ.98.98TZ013.AF286234        ---------- ---------- ---------- ---------- ---------- ---------- ---------- ---------- ---------- ----------
C.TZ.98.98TZ017.AF286235        ---------- ---------- ---------- ---------- ---------- ---------- ---------- ---------- ---------- ----------
C.ZA.00.1069MB.AY838567         ---------- ---TCTGTTG TGTGA----- ---------C TCTGGTAA-C TAGAGATCCC TCAGACCATT --TGTGGTAG T-GTGGAA-- AATCTCCAGC
C.ZA.00.1119MB.AY463229         TAGTGTGTGC CCGTCTGTTG TGTGA----- ---------C TCTGGTAT-C TAGAGATCCC TCAGACCATT ---TTGGTAG T-GTGGAA-- AATCTCTAGC
C.ZA.00.1134MB.AY463217         TAGTGTGTGC CCGTCTGTTG TGTGG----- ---------C TCTGGTAA-C TAGAGATCCC TCAGACCTTT --TTGGT-AG T-GTGGAA-- AATCTCTAGC
C.ZA.00.1157M3M.AY585266        ---------- ---------- ---------- ---------- ---------- ---------- ---------- --CTGGTAAC T-AGAGAA-- AATCTCTAGC
C.ZA.00.1162MB.AY463224         TAGTGTGTGC CCGTCTGTTG TGTGA----- ---------C TCTGGTAA-C TAGAGATCCC TCAGACCCTT --TGTGGTAG T-GTGGAA-- AATCTCTAGC
C.ZA.00.1165MB.AY463230         TAGTGTGTGC CCGTCTGTTG TGTGG----- ---------C TCTGGTAA-C TAGAGATCCC TCAGACCTTT --ATTGGTAG T-GTGGAA-- AATCTCTAGC
C.ZA.00.1168MB.AY463231         TAGTGTGTGC CCGTCTGTTG TGTGG----- ---------C TCTGGTAA-C TAGAGATCCC TCAGACCCTT --GTTGGTAG T-GTGGAA-- AATCTCTAGC
C.ZA.00.1170MB.AY463225         TAGTGTGTGC CCGTCTGTTG TGTGA----- ---------C TCTGGTAA-C TAGAGATCCC TCAGACCTTT ---TTGGCAG T-ATGGAA-- AATCTCTAGC
C.ZA.00.1171MB.AY463232         TAGTGTGTGC CCGTCTGTTG TGTGA----- ---------C TCTGGTAA-C TAGAGATCCC TCAGACCCTT --TGTGGTAG T-GTGGAA-- AATCTCTAGC
C.ZA.00.1176MB.AY463218         ---------- ---------- ---------- ---------C TCTGGTAA-C TAGAGATCCC TCAGACCTAT --TTTGGTAG T-GTGGAA-- AATCTCTAGC
C.ZA.00.1178MB.AY463233         TAGTGTGTGC CCGTCTGTTG TGTGG----- ---------C TCTGGTAA-C TAGAGATCCC TCAGACCCTT --GTTGGTAG T-GAA----- AATCTCTAGC
C.ZA.00.1184MB.AY838566         ---------- ---TCTGTTG TGTGG----- ---------C TCTGGTAA-C TAGAGATCCC TCAGACCCTT --TGTGGTAG T-GTGGAA-- AATCTCTAGC
C.ZA.00.1189MB.AY838565         ---------- ---TCTGTTG TGTGG----- --------AC TCTGGTAA-C TAGAGATCCC TCAGACCCTT -TATTGGTAG T-GTGGAAA- AATCTCTAGC
C.ZA.00.1192M3M.AY463219        TAGTGTGTGC CCGTCTGTTG TGTGA----- ---------C TCTGGTAA-C TAGAGATCCC TCAGACCTTT ---TTGGTAG T-GTGGAA-- AATCTCTAGC
C.ZA.00.1195MB.AY463220         TAGTGTGTGC CCGTCTGTTG TGTGG----- ---------C TCTGGTAA-C TAGAGATCCC TCAGACCCTT --GTTGGTAG T-GCGGAA-- AATCTCTAGC
C.ZA.00.1197MB.AY463234         TAGTGTGTGC CCGTCTGTTG TGTGA----- ---------C TCTGGTAA-C TAGAGATCCC TCAGACCTTT ---TTGGTAG T-GTGGAA-- AATCTCTAGC
C.ZA.00.1210MB.AY463221         ---------- ---------- ---------- ---------C TCTGGTAA-C TAGAGATCCC TCAGACCTTT --TGTGGTAG A-GTGGAA-- AATCTCTAGC
C.ZA.00.1214MB.AY463236         TAGTGTGTGC CCGTCTGTTG TGTGA----- ---------C TCTGGTAA-C TAGAGATCCC TCAGACCATT --CGTGGTAG T-GTGGAA-- AATCTCTAGC
C.ZA.00.1217MB.AY463226         TAGTGTGTGC CCGTCTGTTG TGTGA----- ---------C TCTGGTAA-C TAGAGATCCC TCAGACCCTT --TTTGGTAG T-GTGGAA-- AATCTCTAGC
C.ZA.00.1225MB.AY463227         TAGTGTGTGC CCGTCTGTTG TGTGG----- ---------C TCTGGTAA-C TAGAGATCCC TCAGACCCTT --GTTGGTAT T-GTGGCA-- AATCTCTAGC
C.ZA.00.1228MB.AY463222         TAGTGTGTGC CCGTCTGTTG TGTGG----- ---------C TCTGGTAA-C TAGAGATCCC TCAGACCATT ---TTGGTAG T-GTGGAA-- AATCTTTAGC
C.ZA.00.J112MA.AY838568         ---------- ---TCTGTTG TGTGG----- ---------C TCTGGTAA-C TAGAGATCCC TCAGACCAAC --TTTGGTAG T-GTGGAA-- AATCTCTAGC
C.ZA.00.J38MA.AY463228          TAGTGTGTGC CCGTCTGTTG TGTGA----- ---------C TCTGGTAA-C TAGAGATCCC TCAGACCATT ---TTGGAAG T-GTGGAA-- AATCTCTAGC
C.ZA.01.01ZATM45.AY228557       TAGTGTGTGC CCATCTGTTG TGTGACTCTG GTCTGTGA-C TCTGGTAA-C TAGAGATCCC TCAGACCCTT --TGTGGTAG T-GTGGAA-- AATCTCTAGC
C.ZA.01.2134MB.AY463237         TAGTGTGTGC CCGTCTGTTG TGTGA----- ---------C TCTGGTAG-C TAGAGATCCC TCAGACCCTT --TGTGGTAG T-GTGGAA-- AATCTCTAGC
C.ZA.01.J54Ma.AY463223          TAGTGTGTGC CCGTCTGTTG TGTGG----- ---------C TCTGGTAA-C TAGAGATCCC TCAGACTCTT --TGTGGTAG T-GCGGAA-- AATCTCTAGC
C.ZA.02.02ZAPS001MB1.DQ275648   ---------- ---TCTGTTG TGTGG----- ---------C TCTGGTAA-C TAGAGATCCC TCAGACCAAT ---TTGGTAG T-GTGGAA-- AATCTCTAGC
C.ZA.02.02ZAPS005MB1.DQ351235   ---------- ---TCTGTTG TGTGG----- ---------C TCTGGTAG-C TAGAGATCCC TCAGATCCTT --AGTGGTAG T-GTGGAAA- AATCTCTAGC
C.ZA.02.02ZAPS006MB1.DQ351220   ---------- ---TCTGTTG TGTGG----- ---------C TCTGGTAA-C TAGAGATCCC TCAGACCCTT --CGTGGTAG T-GTGGAA-- AATCTCTAGC
C.ZA.02.02ZAPS008MB1.DQ275647   ---------- ---TCTGTTG TGTGG----- ---------C TCTGGTAA-C TAGAGATCCC TCAGACCAAT --TTTGGTAG T-GTGGAA-- AATCTCTAGC
C.ZA.02.02ZAPS013MB1.DQ351222   ---------- ---TCTGTTG TGTGA----- ---------C TCTGGGAA-C TAGAGATCCC TCAGACCTTT ---GCGGCTG T-GTGGAA-- AATCTCTAGC
C.ZA.02.02ZAPS014MB1.DQ351218   ---------- ---TCTGTTG TGTGA----- ---------C TCTGGTAA-C TAGAGATCCC TCAGACCCTT --TGTGGTAG T-GTGGAA-- AATCTCTAGC
C.ZA.02.02ZAPS015MB1.DQ369995   ---------- ---TCTGTTG TGTGA----- ---------C TCTGGTAA-C TAGAGATCCC TCAGACCAAT --TTTGGTAG T-GTGGAA-- AATCTCTAGC
C.ZA.03.03ZAPS017MB1.DQ351224   ---------- ---TCTGTTG TGTGA----- ---------C TCTGGTAA-C TAGAGATCCC TCAGACCAAG --TTTGGTAG T-GTGGAA-- AATCTCTAGC
C.ZA.03.03ZAPS020MB1.DQ275653   ---------- ---TCTGTTG TGTGG----- ---------C TCTGGTAA-C TAGAGATCCC TCAGACACTT --GTTGGTTG T-GTGGAA-- AGTCTCTAGC
C.ZA.03.03ZAPS021MB1.DQ369978   ---------- ---TCTGTTG TGTGG----- ---------C TCTGGTAA-C TAGAGATCCC TCAGACCCTT --TTTGGTTG T-GTGGAA-- AATCTCTAGC
C.ZA.03.03ZAPS023MB1.DQ351225   ---------- ---TCTGTTG TGTGA----- ---------C TCTGGTAA-C TAGAGATCCC TCAGACCAAC --TTTGGTAG T-GTGGAA-- AATCTCTAGC
C.ZA.03.03ZAPS024MB1.DQ396367   ---------- ---TCTGTTG TGTGA----- ---------C TCTGGTAG-C TAGAGATCCC TCAGACCCTT ---TTGGTAG T-GTGGAA-- AATCTCTAGC
C.ZA.03.03ZAPS025MB1.DQ351226   ---------- ---TCTGTTG TGTGA----- ---------C TCTGGTAA-C TAGAGATCCC TCAGACCCTT --GTTGGTAG T-GTGGAA-- AATCTCTAGC
C.ZA.03.03ZAPS026MB1.DQ369985   ---------- ---TCTGTTG TGTGA----- ---------C TCTGGTAA-C TAGAGATCCC TCAGACCCTT --TGTGGTAG T-GTGGAA-- AATCTCTAGC
C.ZA.03.03ZAPS027MB1.DQ351223   ---------- ---TCTGTTG TGTGG----- ---------C TCTGGTAA-C TAGAGATCCC TCAGACCCTT --AGTGGTAG T-GTGGAA-- AATCTCTAGC
C.ZA.03.03ZAPS030MB1.DQ369996   ---------- ---TCTGTTG TGTGA----- ---------C TCCGGTAA-C TAGAGATCCC TCAGACCCTT --GTTGGTAG T-GTGGAA-- AATCTCTAGC
C.ZA.03.03ZAPS032MB1.DQ445633   ---------- ---TCTGTTG TGTGG----- ---------C TCTGGTAA-C TAGAGATCCC TCAGACCCTT --TTTGGTAG T-GTGGAA-- AATCTCTAGC
C.ZA.03.03ZAPS034MB1.DQ369979   ---------- ---TCTGTTG TGTGG----- ---------C TCTGGGAA-C TAGAGATCCC TCAGACCCTT --TTTGGCTG T-GTGGAA-- AATCTCTAGC
C.ZA.03.03ZAPS042MB1.DQ369977   ---------- ---TCTGTTG TGTGG----- ---------C TCTGGTAA-C TAGAGATCCC TCAGACCCTT --TGTGGTAG T-GTGGAA-- AATCTCTAGC
C.ZA.03.03ZAPS043MB1.DQ351227   ---------- ---TCTGTTG TGTGA----- ---------C TCTGGTAA-C TAGAGATCCC TCAGACCCTT ---TTGGTAG T-GTGGAA-- AATCTCTAGC
C.ZA.03.03ZAPS044MB1.DQ396384   ---------- ---TCTGTTG TGTGG----- ---------C TCTGGTAA-C TAGAGATCCC TCAGACCCTT ---TTGGTTG T-GTGGAA-- AATCTCTAGC
C.ZA.03.03ZAPS046MB1.DQ369984   ---------- ---TCTGTTG TGTGG----- ---------C TCTGGTAA-C TAGAGATCCC TCAGACCCTT --TTTGGTAG T-GTGGAA-- AATCTCTAGC
C.ZA.03.03ZAPS048MB1.DQ396364   ---------- ---TCTGTTG TGTGG----- ---------C TCTGGTAA-C TAGAGATCCC TCAGACCTTT ---TTGGTAG T-GTGGAA-- AATCTCTAGC
C.ZA.03.03ZAPS049MB1.DQ369986   ---------- ---TCTGTTG TGTGG----- ---------C TCTGGTAA-C TAGAGATCCC TCAGACCAAG --TTTGGTAG C-GTGGAA-- AATCTCTAGC
C.ZA.03.03ZAPS050MB1.DQ369980   ---------- ---TCTGTTG TGTGG----- ---------C TCTGGTAA-C TAGAGATCCC TCAGACCTTT ---TGGGTTG T-GTGGAA-- AATCTCTAGC
C.ZA.03.03ZAPS051MB1.DQ396385   ---------- ---TCTGTTG TGTGG----- ---------C TCTGGTAA-C TAGAGATCCC TCAGACCCTT --TTTGGTAG T-GTGGAA-- AATCTCTAGC
C.ZA.03.03ZAPS052MB1.DQ369987   ---------- ---TCTGTTG TGTGA----- ---------C TCTGGTAA-C TAGAGATCCC TCAGACCAAT --ATTGGTAG T-GTGGAA-- AATCTCTAGC
C.ZA.03.03ZAPS054MB2.DQ369988   ---------- ---TCTGTTG TGTGG----- ---------C TCTGGTAA-C TAGAGATCCC TCAGACCCAT --TGTGGTAG T-GTGGAA-- AATCTCTAGC
C.ZA.03.03ZAPS055MB1.DQ396373   ---------- ---TCTGTTG TGTGG----- ---------C TCTGGTAA-C TAGAGATCCC TCAGACTCCT --TTTGGTAG T-TTGGAA-- AATCTCTAGC
C.ZA.03.03ZAPS056MB1.DQ396374   ---------- ---TCTGTTG TGTGG----- ---------C TCTGGTAA-C TAGAGATCCC TCAGACCCTT --TTTGGTAG T-GTGGAA-- AATCTCTAGC
C.ZA.03.03ZAPS057MB2.DQ369989   ---------- ---TCTGTTG TGTGA----- ---------C TCTGGTAA-C TAGAGATCCC TCAGACCCTT --TTTGGTAG T-GTGGAA-- AATCTCTAGC
C.ZA.03.03ZAPS059MB2.DQ445634   ---------- ---TCTGTTG TGTGG----- ---------C TCTGGTAA-C TAGAGATCCC TCAGACCCTT --GTTGGTAG T-GTGGAA-- AATCTCTAGC
C.ZA.03.03ZAPS063MB1.DQ396388   ---------- ---TCTGTTG TGTGG----- ---------C TCTGGTAA-C TAGAGATCCC TCAGACCAAC --TTTGGTAG T-GTGGAA-- AATCTCTAGC
C.ZA.03.03ZAPS066MB2.DQ396375   ---------- ---TCTGTTG TGTGA----- ---------C TCTGGTAA-C TAGAGATCCC TCAGACCCTT --TTTGGTAG T-GTGGAA-- AATCTCTAGC
C.ZA.03.03ZAPS067MB2.DQ396389   ---------- ---TCTGTTG TGTGG----- ---------C TCTGGTAA-C TAGAGATCCC TCAGACCAAT --AGTGGTAG T-GTGGAA-- AATCTCTAGC
C.ZA.03.03ZAPS071MB1.DQ396376   ---------- ---TCTGTTG TGTGG----- ---------C TCTGGTAA-C TAGAGATCCC TCAGACCCTT --TGTGGTAG T-ATGGAA-- AATCTCTAGC
C.ZA.03.03ZAPS073MB1.DQ275649   ---------- ---TCTGTTG TGTGA----- ---------C TCTGGTAA-C TAGAGATCCC TCAGACCATT --TGTGGTAG T-GTGGAA-- AATCTCTAGC
C.ZA.03.03ZAPS074MB2.DQ351228   ---------- ---TCTGTTG TGTGG----- ---------C TCTGGTAA-C TAGAGATCCC TCAGACCCAT --TTTGGTAG T-GTGGAA-- AATCTCTAGC
C.ZA.03.03ZAPS077B1.DQ093591    ---------- ---TCTGTTG TGTGA----- ---------C TCTGGTAA-C TAGAGATCCC TCAGACCATT ---TTGGTAG T-GTGGAA-- AATCTCTAGC
C.ZA.03.03ZAPS079B1.DQ093592    ---------- ---TCTGTTG TGTGA----- ---------C TCTGGTAA-C TAGAGATCCC TCAGACCCTT --GTTGGTAG T-GTGGAA-- AATCTCTAGC
C.ZA.03.03ZAPS081MB1.DQ351219   ---------- ---TCTGTTG TGTGG----- ---------C TCTGGTAA-C TAGAGATCCC TCAGACCCTT --TTTGGTGG T-GTGGAA-- AATCTCTAGC
C.ZA.03.03ZAPS083MB1.DQ351229   ---------- ---TCTGTTG TGTGG----- ---------C TCTGGTAA-C TAGAGATCCC TCAGACCCTT ---TTGGTAG T-GTGGAA-- AATCTCTAGC
C.ZA.03.03ZAPS086MB1.DQ275654   ---------- ---TCTGTTG TGTGA----- ---------C TCTGGTAA-C TAGAGATCCC TCAGACCTTT --GTTGGTAG T-GTGGAA-- AATCTCTAGC
C.ZA.03.03ZAPS088MB1.DQ275651   ---------- ---TCTGTTG TGTGG----- ---------C TCTGGTAA-C TAGAGATCCC TCAGACCCTT --TTGGGTAG T-GTGGAA-- AATCTCTAGC
C.ZA.03.03ZAPS089MB1.DQ351216   ---------- ---TCTGTTG TGTGG----- ---------C TCTGGTAA-C TAGAGATCCC TCAGACCCTT --TTTGGTAG T-GTGGAA-- AATCTCTAGC
```

C.ZA.03.03ZAPS091MB1.DQ275645	----------	---TCTGTTG	TGTGA-----	---------C	TCTGGTAA-C	TAGAGATCCC	TCAGACCATT	---TTGGTAG	T-GTGGAA--	AATCTCTAGC
C.ZA.03.03ZAPS094MB1.DQ396377	----------	---TCTGTTG	TGTGG-----	---------C	TCTGGTAA-C	TAGAGATCCC	TCAGACCCTT	--TTTGGTAG	T-GTGGAA--	AATCTCTAGC
C.ZA.03.03ZAPS095MB1.DQ275652	----------	---TCTGTTG	TGTGA-----	---------C	TCTGGTAA-C	TAGAGATCCC	TCAGACCCTT	--TTTGGTAG	T-GTGGAA--	AATCTCTAGC
C.ZA.03.03ZAPS097MB1.DQ351230	----------	---TCTGTTG	TGTGA-----	---------C	TCTGGTAA-C	TAGAGATCCC	TCAGACCCTT	--TGTGGTAG	T-GTGGAA--	AATCTCTAGC
C.ZA.03.03ZAPS099MB1.DQ275655	----------	---TCTGTTG	TGTGA-----	---------C	TCTGGTAA-C	TAGAGATCCC	TCAGACCAGT	---TTGGTAG	T-GTGGAA--	AATCTCTAGC
C.ZA.03.03ZAPS103MB2.DQ275656	----------	---TCTGTTG	TGTGG-----	---------C	TCTGGTAA-C	TAGAGATCCC	TCAGACCAAC	--TTTGGTAG	C-GTGGAA--	AATCTCTAGC
C.ZA.03.03ZAPS104MB1.DQ369990	----------	---TCTGTTG	TGTGA-----	---------C	TCTGGTAA-C	TAGAGATCCC	TCAGACCTTT	---TTGGTAG	C-GCGGAA--	AATCTCTAGC
C.ZA.03.03ZAPS105MB2.DQ445632	----------	---TCTGTTG	TGTGA-----	---------C	TCTGGTAA-C	TAGAGATCCC	TCAGACCTTT	--CTTGGTAG	T-GTGGAA--	AATCTCTAGC
C.ZA.03.03ZAPS108MB1.DQ396378	----------	---TCTGTTG	TGTGA-----	---------C	TCTGGTAA-C	TAGAGACCCC	TCAGACCCTT	--TTTGGTAG	T-GTGGAA--	AATCTCTAGC
C.ZA.03.03ZAPS112MB2.DQ396386	----------	---TCTGTTG	TGTGA-----	---------C	TCTGGTAA-C	TAGAGATCCC	TCAGACCCTT	--TGTGGTAG	A-GTGGAA--	AATCTCTAGC
C.ZA.03.03ZAPS113MB2.DQ396365	----------	---TCTGTTG	TGTGA-----	---------C	TCTGGTAA-C	TAGAGATCCC	TCAGACCATT	--CGTGGTAG	T-GTGGAA--	AATCTCTAGC
C.ZA.03.03ZAPS116MB1.DQ445635	----------	---TCTGTTG	TGTGA-----	---------C	TCTGGTAA-C	TAGAGATCCC	TCAGACCAAT	--TTTGGTAG	T-GTGGAA--	AATCTCTAGC
C.ZA.03.03ZAPS118MB1.DQ396368	----------	---TCTGTTG	TGTGG-----	---------C	TCTGGTAA-C	TAGAGATCCC	TCAGAACCTT	--TTTGGTAG	T-GTGGAA--	AATCTCTAGC
C.ZA.03.03ZAPS122MB1.DQ396370	----------	---TCTGTTG	TGTGA-----	---------C	TCTGGTAA-C	TAGAGATCCC	TCAGACCCTT	--TTGGGTAG	T-GTGGAA--	AATCTCTAGC
C.ZA.03.03ZAPS123MB1.DQ396369	----------	---TCTGTTG	TGTGG-----	---------C	TCTGGTAA-C	TAGAGATCCC	TCAGACCCTT	--GTTGGTAG	T-GTGGAA--	AATCTCTAGC
C.ZA.03.03ZAPS124MB1.DQ369976	----------	---TCTGTTG	TGTGA-----	---------C	TCTGGTAA-C	TAGAGATCCC	TCAGACCCTT	--TGTGGTAG	T-GTGGAA--	AATCTCTAGC
C.ZA.03.03ZAPS125MB1.DQ396390	----------	---TCTGTTG	TGTGG-----	---------C	TCTGGTAA-C	TAGAGATCCC	TCAGACCAAG	--TTGGTTCG	T-GTGGAA--	AATCTCTAGC
C.ZA.03.03ZAPS126MB1.DQ275657	----------	---TCTGTTG	TGTGA-----	---------C	TCTGGTAA-C	TAGAGATCCC	TCAGACCCTT	--TTTGGTTG	T-GTGGAA--	AATCTCTAGC
C.ZA.03.03ZAPS128MB1.DQ275643	----------	---TCTGTTG	TGTGA-----	---------C	TCTGGTAA-C	TAGAGATCCC	TCAGACCTTT	---TTGGTAG	T-AAGGAA--	AATCTCTAGC
C.ZA.03.03ZAPS130MB1.DQ275658	----------	---TCTGTTG	TGTGA-----	---------C	TCTGGTAA-C	TAGAGATCCC	TCAGACCCTT	--TGAGGTAG	T-GTGGAA--	AATCTCTAGC
C.ZA.03.03ZAPS131MB1.DQ396380	----------	---TCTGTTG	TGTGG-----	---------C	TCTGGTAA-C	TAGAGATCCC	TCAGACCATT	--TCTGGTAG	T-GTGGAA--	AATCTCTAGC
C.ZA.03.03ZAPS133MB1.DQ275646	----------	---TCTGTTG	TGTGA-----	---------C	TCTGGTAA-C	TAGAGATCCC	TCAGATCCTT	---TTGGTAG	T-GTGGAA--	AATCTCTAGC
C.ZA.03.03ZAPS136MB1.DQ351231	----------	---TCTGTTG	TGTGA-----	---------C	TCTGGTAA-C	TAGAGATCCC	TCAGACCAAT	--TTTGGTAG	T-GTGGAA--	AATCTCTAGC
C.ZA.03.03ZAPS140MB1.DQ369981	----------	---TCTGTTG	TGTGA-----	---------C	TCTGGTAA-C	TAGGGATCCC	TCAGACCCTT	--TTTGGTAG	T-GTGGAA--	AATCTCTAGC
C.ZA.03.03ZAPS143MB1.DQ396391	----------	---TCTGTTG	TGTGA-----	---------C	TCTGGTAA-C	TAGAGATCCC	TCAGACCATT	--GTAGGTAG	T-GTGGAA--	AATCTCTAGA
C.ZA.03.03ZAPS151MB1.DQ396392	----------	---TCTGTTG	TGTGA-----	---------C	TCTGGTAA-C	TAGAGATCCC	TCAGACCCTT	--TTAGTCAG	T-GTGGAA--	AATCTCTAGC
C.ZA.03.03ZAPS152MB1.DQ396399	----------	---TCTGTTG	TGTGA-----	---------C	TCTGGTAG-C	TAGAGATCCC	TCAGATCCTT	--TTTGGTAG	T-GTGGAA--	AATCTCTAGC
C.ZA.03.03ZAPS155MB1.DQ396371	----------	---TCTGTTG	TGTGG-----	---------C	TCTGGTAA-C	TAGAGATCCC	TCAGACCTCT	--TTTGGTAG	T-GTGGAA--	AATCTCTAGC
C.ZA.03.03ZASK005B2.DQ011175	----------	---TCTGTTG	TGTGA-----	---------C	TCTGGTAA-C	TAGAGATCCC	TCAGACCAGT	--TTTGGGAG	T-GTGGAA--	AATCTCTAGC
C.ZA.03.03ZASK006B2.AY878056	----------	---TCTGTTG	TGTGA-----	---------C	TCTGGTAA-C	TAGAGATCCC	TCAGACCCTT	--TTTGGTAG	T-GTGGAA--	AATCTCTAGC
C.ZA.03.03ZASK010B2.DQ164104	----------	---TCTGTTG	TGTGA-----	---------C	TCTGGTAA-C	TAGAGGTCCC	TCAGACCCTT	---TGGGTAT	T-GTGGAA--	AATCTCTAGC
C.ZA.03.03ZASK011B2.AY901965	----------	---TCTGTTG	TGTGA-----	---------C	TCTGGTAA-C	TAGAGATCCC	TCAGACCAAC	--TTTGGTAG	T-GTGGAA--	AATCTCTAGC
C.ZA.03.03ZASK013B2.DQ275660	----------	----------	----A-----	---------C	TCTGGTAA-C	TAGAGATCCC	TCAGACCCTT	--TTTGGTAG	T-GTGGAA--	AATCTCTAGC
C.ZA.03.03ZASK016MB2.DQ351233	----------	---TCTGTTG	TGTGA-----	---------C	TCTGGTAA-T	TAGAGATCCC	TCAGACCAAC	--TTTGGTAG	T-GTGGAA--	AATCTCTAGC
C.ZA.03.03ZASK019B2.AY878063	----------	---TCTGTTG	TGTGA-----	---------C	TCTGGTAA-C	TAGAGATCCC	TCAGACTCTT	--TTGGTTAG	T-GTGGAA--	AATCTCTAGC
C.ZA.03.03ZASK020B2.AY878064	----------	---TCTGTTG	TGTGA-----	---------C	TCTGGTAA-C	TAGAGATCCC	TCAGACCTTT	--TTGGTAGT	--GTGGA---	AATCTCTAGC
C.ZA.03.03ZASK026B2.DQ011165	----------	---TCTGTTG	TGTGA-----	---------C	TCTGGTAA-C	TAGAGATCCC	TCAGACCAAT	---TTGGTAG	T-GTGGAA--	AATCTCTAGC
C.ZA.03.03ZASK034B1.AY878065	----------	----------	----------	----------	----------	----------	----------	----------	--TTGGGA--	AATCTCTAGC
C.ZA.03.03ZASK036B1.AY901966	----------	---TCTGTTG	TGTGA-----	---------C	TCTGGTAA-C	TAGAGATCCC	TCAGACCCTT	--TTTGGTAG	T-GTGGAA--	AATCTCTAGC
C.ZA.03.03ZASK039B2.AY878068	----------	---TCTGTTG	TGTGA-----	---------C	TCTGGTAG-C	TAGAGATCCC	TCAGACCCTT	--TTTGGTAG	T-GTGGAA--	AATCTCTAGC
C.ZA.03.03ZASK058B2.AY901967	----------	---TCTGTTG	TGTGA-----	---------C	TCTGGTAA-C	TAGAGATCCC	TCAGACCAAT	--TGTGTTAG	T-GTGGAA--	AATCTCTAGC
C.ZA.03.03ZASK061B1.AY901968	----------	---TCTGTTG	TGTGA-----	---------C	TCTGGTAA-C	TAGAGATCCC	TCAGACCATT	---TTGGTAG	T-GTGGAA--	AATCTCTAGC
C.ZA.03.03ZASK062B1.DQ164113	----------	---TCTGTG-	TGTGA-----	---------C	TCTGGTAA-C	TAGAGATCCC	TCAGACCCTT	--TGTGGTAG	T-GTGG----	AATCTCTAGC
C.ZA.03.03ZASK066B1.AY901969	----------	---TCTGTTG	TGTGA-----	---------C	TCTGGTAA-C	TAGAGATCCC	TCAGACCCTT	---TTGGTAG	T-GTGGAA--	AATCTCTAGC
C.ZA.03.03ZASK067B1.DQ275642	----------	---TCTGTTG	TGTGA-----	---------C	TCTGGTAA-C	TAGAGATCCC	TCAGACCTTT	---TTGGTAG	T-GTGGAA--	AATCTCTAGC
C.ZA.03.03ZASK072B1.DQ093593	----------	---TCTGTTG	TGTGA-----	---------C	TCTGGTAA-C	TAGAGGTCCC	TCAGACCCTT	--TTTGGTAG	T-GTGGAA--	AATCTCTAGC
C.ZA.03.03ZASK073B1.AY901970	----------	---TCTGTTG	TGTGA-----	---------C	TCTGGTAA-C	TAGAGATCCC	TCAGACCATT	---TTGGTAG	C-GTGGAA--	AATCTCTAGC
C.ZA.03.03ZASK076B1.AY901975	----------	---TCTGTTG	TGTGA-----	---------C	TCTGGTAA-C	TAGAGATCCC	TCAGACCATT	--CGTGGTAG	T-GTGGAA--	AATCTCTAGC
C.ZA.03.03ZASK078B1.AY901971	----------	---TCTGTTG	TGTGA-----	---------C	TCTGGTAA-C	TAGAGATCCC	TCAGACCAAC	--TTTGGTAG	T-GTGGAA--	AATCTCTAGC
C.ZA.03.03ZASK084B1.AY901981	----------	---TCTGTTG	TGTGA-----	---------C	TCTGGTAA-C	TAGAGATCCC	TCAGACCCTT	---TTGGTAG	C-GTGGAA--	AATCTCTAGC
C.ZA.03.03ZASK092B1.AY878057	----------	---TCTGTTG	TGTGA-----	---------C	TCTGGTAA-C	TAGAGATCCC	TCAGACCCTC	--GTTGGTAG	T-GCGGAA--	AATCTCTAGC
C.ZA.03.03ZASK094B1.AY878070	----------	---TCTGTTG	TGTGA-----	---------C	TCTGGTAA-C	TAGAGATCCC	TCAGACTCAT	--TTGAGTAG	T-GTGGAA--	AATCTCTAGC
C.ZA.03.03ZASK097B1.AY878060	----------	---TCTGTTG	TGTGA-----	---------C	TCTGGTAA-C	TAGAGATCCC	TCAGACCCTT	--GTTGGTAG	T-GTGGAA--	AATCTCTAGC
C.ZA.03.03ZASK098B1.AY878061	----------	---TCTGTTG	TGTGA-----	---------C	TCTGGTAA-C	TAGAGATCCC	TCAGACCTTT	---TTGGTAG	T-GTGGAA--	AATCTCTAGC
C.ZA.03.03ZASK103B1.DQ164106	----------	---TCTGTTG	TGTGA-----	---------C	TCTGGTAA-C	TAGAGATCCC	TCAGACCAAT	--TTTGGTAG	T-GTGGAA--	AATCTCTAGC
C.ZA.03.03ZASK104B1.DQ396395	----------	---TCTGTTG	TGTGG-----	---------C	TCTGGTAA-C	TAGAGATCCC	TCAGACCATT	---TTGGTAG	T-GTGGAA--	AATCTCTAGC
C.ZA.03.03ZASK107B1.DQ056410	----------	---TCTGTTG	TGTGA-----	---------C	TCTGGTAA-C	TAGAGATCCC	TCAGACCAAC	--TTTGGTAG	T-GTGGAA--	AATCTCTAGC
C.ZA.03.03ZASK110B1.DQ056411	----------	---TCTGTTG	TGTGA-----	---------C	TCTGGTAA-C	TAGAGATCCC	TCAGACCCTT	--TTTGGTAG	T-GTGGAA--	AATCTCTAGC
C.ZA.03.03ZASK111B1.DQ056404	----------	---TCTGTTG	TGTGA-----	---------C	TCTGGTAA-C	TAGAGATCCC	TCAGACCCTT	---GTGGTAG	T-GTGGAA--	AATCTCTAGC
C.ZA.03.03ZASK113B1.DQ351237	----------	---TCTGTTG	TGTGA-----	---------C	TCTGGTAA-C	TAGAGATCCC	TCAGACCATT	--TCTGGTTA	GTGTGGAA--	AATCTCTAGC
C.ZA.03.03ZASK117B1.DQ056408	----------	---TCTGTTG	TGTGA-----	---------C	TCTGGTAA-C	TAGAGATCCC	TCAGACCATT	---TTGGCAG	T-GTGGAA--	AATCTCTAGC
C.ZA.03.03ZASK118B1.DQ011169	----------	---TCTGTTG	TGTGA-----	---------C	TCTGGTAA-C	TAGAGATCCC	TCAGACCCTT	--TGTGGTAG	T-GTGGAA--	AATCTCTAGC
C.ZA.03.03ZASK120B1.DQ011176	----------	---TCTGTTG	TGTGA-----	---------C	TCTGGTAT-C	TAGAGGTCCC	TCAGACCCTT	---GTGGTAG	T-GTGGAA--	AATCTCTAGC
C.ZA.03.03ZASK211B1.DQ093601	----------	---TCTGTTG	TGTGA-----	---------C	TCTGGTAA-C	TAGAGATCCC	TCAGACCTTT	--TGTGGTAG	T-GTGGAA--	AATCTCTAGC
C.ZA.03.03ZASK212B.DQ978981	----------	---TCTGTTG	TGTGA-----	---------C	TCTGGTAA-C	TAGAGATCCC	TCAGACCCTT	---TTGGTAG	T-GTGGAA--	AATCTCTAGC
C.ZA.03.03ZASK213B1.DQ093607	----------	---TCTGTTG	TGTGA-----	---------C	TCTGGTAA-C	TAGAGATCCC	TCAGACCCTT	--TGTGGTAG	T-GTGGAA--	AATCTCTAGC
C.ZA.03.03ZASK215M6W.DQ275661	----------	---TCTGTTG	TGTGA-----	---------C	TCTGGTAA-C	TAGAGATCCC	TCAGACCCTT	--TTGGTTCG	T-GTGGAA--	AATCTCTAGC
C.ZA.03.03ZASK223B1.DQ093597	----------	---TCTGTTG	TGTGA-----	---------C	TCTGGTAA-C	TAGAGATCCC	TCAGACCAAC	--TTTGGTAG	T-GTGGAA--	AATCTCTAGC
C.ZA.03.03ZASK224MB1.DQ275664	----------	---TCTGTTG	TGTGA-----	---------C	TCTGGTAG-C	TAGAGATCCC	TCAGACCATT	---TTGGTAG	T-GTGGAA--	AATCTCTAGC
C.ZA.03.03ZASK226B1.DQ164108	----------	---TCTGTTG	TGTGA-----	---------C	TCTGGTAA-C	TAGAGATCCC	TCAGATCCTT	--TTTGGCTG	T-GTGGAA--	AATCTCTAGC
C.ZA.03.03ZASK232B1.DQ093589	----------	---TCTGTTG	TGTGA-----	---------C	TCTGGTAA-C	TAGAGATCCC	TCAGACCCTT	--CGTGGTAG	T-GTGGAA--	AATCTCTAGC
C.ZA.03.03ZASK233B1.DQ351234	----------	---TCTGTTG	TGTGA-----	---------C	TCTGGTAA-C	TAGAGATCCC	TCAGACTCTT	--TGTAGTAG	T-GTGGAA--	AATCTCTAGC
C.ZA.03.04ZAPS194MB1.DQ275650	----------	---TCTGTTG	TGTGA-----	---------C	TCTGGTAA-C	TAGAGATCCC	TCAGACCCTT	--TGTGGTGG	T-GTGGAA--	AATCTCTAGC
C.ZA.03.04ZASK082B1.AY901972	----------	---TCTGTTG	TGTGA-----	---------C	TCTGGTAA-C	TAGAGATCCC	TCAGACCATT	--TGTGGTAG	T-GTGAAA--	AATCTCTAGC
C.ZA.03.SK023B2.AY772690	----------	---TCTGTTG	TGTGA-----	---------C	TCTGGTAA-C	TAGAGATCCC	TCAGACCCAT	--TTTGGTAG	T-GTGGAA--	AATCTCTAGC
C.ZA.03.SK029B2.AY772691	----------	---TCTGTTG	TGTGA-----	---------C	TCTGGTAA-C	TAGAGATCCC	TCAGACCATT	---TTGGTAG	T-GTGGAA--	AATCCCTAGC
C.ZA.03.SK033B2.AY772692	----------	---TCTGTTG	TGTGA-----	---------C	TCTGGTAA-C	TAGAGATCCC	TCAGACCCTT	--TTTGGTTG	T-GTGGAA--	AATCTCTAGC
C.ZA.03.SK040B1.AY703908	----------	---TCTGTTG	TGTGA-----	---------C	TCTGGTAA-C	TAGAGATCCC	TCAGACCCTT	--TGTGGTAG	T-GTGGAA--	AATCTCTAGC
C.ZA.03.SK041B1.AY772693	----------	----------	----------	----------	----------	----------	----------	----------	--TTGGGA--	AATCTCTAGC
C.ZA.03.SK043B1.AY772700	----------	---TCTGTTG	TGTGA-----	---------C	TCTGGTAA-C	TAGAGATCCC	TCAGACCCTT	--TGTGGTAG	T-GTGGAA--	AATCTCTAGC
C.ZA.03.SK065B1.AY772694	----------	---TCTGTTG	TGTGA-----	---------C	TCTGGTAA-C	TAGAGATCCC	TCAGACCAAA	--AGCGGTAG	T-GTGGAA--	AATCTCTAGC
C.ZA.03.SK112B1.AY772695	----------	---TCTGTTG	TGTGA-----	---------C	TCTGGTAA-C	TAGAGATCCC	TCAGACCCTT	--TTTGGTAG	T-GTGGAA--	AATCTCTAGC
C.ZA.03.SK116B1.AY772696	----------	---TCTGTTG	TGTGA-----	---------C	TCTGGTAA-C	TAGAGATCCC	TCAGACCCTT	--TGTGGCAG	T-GTGGAA--	AATCTCTAGA
C.ZA.04.04ZAPS157MB1.DQ351232	----------	---TCTGTTG	TGTGA-----	---------C	TCTGGTAA-C	TAGAGATCCC	TCAGACCATT	---TTGGTAG	T-GTGGAA--	AATCTCTAGC
C.ZA.04.04ZAPS160B1.DQ164107	----------	---TCTGTTG	TGTGA-----	---------C	TCTGGTAA-C	TAGAGATCCC	TCAGATCCTT	--TTTGGTAG	T-GTGAAA--	AATCTCTAGC
C.ZA.04.04ZAPS161B1.DQ164115	----------	---TCTGTTG	TGTGA-----	---------C	TCTGGTAA-C	TAGAGATCCC	TCAGACCTTT	--TGTGTTAG	T-GTGGAA--	AATCTCTAGC
C.ZA.04.04ZAPS165MB1.DQ351221	----------	---TCTGTTG	TGTGA-----	---------C	TCTGGTAA-C	TAGAGATCCC	TCAGACCTAT	--ATTGGTAG	T-GTGGAA--	AATCTCTAGC
C.ZA.04.04ZAPS168B1.DQ164121	----------	---TCTGTTG	TGTGA-----	---------C	TCTGGTAT-C	TAGAGATCCC	TCAGACCTTT	---TTGGTAG	T-GTGGAA--	AATCTCTAGC
C.ZA.04.04ZAPS169MB1.DQ396393	----------	---TCTGTTG	TGTGA-----	---------C	TCTGGTAG-C	TAGAGATCCC	TCAGAACCTT	---TTGGCAG	T-GTGGAA--	AATCTCTAGC
C.ZA.04.04ZAPS172MB1.DQ275659	----------	---TCTGTTG	TGTGA-----	---------C	TCTGGTAT-C	TAGAGATCCC	TCAGACCCTT	--GTTGGTAG	T-GTGGAA--	AATCTCTAGC
C.ZA.04.04ZAPS177MB1.DQ396394	----------	---TCTGTTG	TGTGA-----	---------C	TCTGGTAA-C	TAGAGATCCC	TCAGACCAAT	--CTTGGTGG	T-GTGGAA--	AATCTCTAGC
C.ZA.04.04ZAPS188B1.DQ164122	----------	---TCTGTTG	TGTGA-----	---------C	TCTGGTAA-C	TAGAGATCCC	TCAGACCTTT	--TGAGGTAG	T-GTGGAA--	AATCTCTAGC
C.ZA.04.04ZAPS189B1.DQ164117	----------	---TCTGTTG	TGTGA-----	---------C	TCTGGTAA-C	TAGAGATCCC	TCAGACCAGT	---TTGGTAG	T-GTGGAA--	AATCTCTAGC
C.ZA.04.04ZAPS190B1.DQ093602	----------	---TCTGTTG	TGTGA-----	---------C	TCTGGTAA-C	TAGAGATCCC	TCAGACCTTT	--TGAGGTAG	T-GTGGAA--	AATCTCTAGC
C.ZA.04.04ZAPS195B1.DQ164118	----------	---TCTGTTG	TGTGA-----	---------C	TCTGGTAA-C	TAGAGATCCC	TCAGACCCTT	--TTTGGTAG	T-GTGGAA--	AATCTCTAGC
C.ZA.04.04ZAPS197MB1.DQ369997	----------	---TCTGTTG	TGTGA-----	---------C	TCTGGTAA-C	TAGAGATCCC	TCAGACCCTT	--CGTGGTAG	T-GTGGAA--	AATCTCTAGC
C.ZA.04.04ZAPS198MB1.DQ445637	----------	---TCTGTTG	TGTGA-----	---------C	TCTGGTAA-C	TAGAGATCCC	TCAGATCAAT	--TTTGGTCG	T-GTGGAA--	AATCTCTAGC
C.ZA.04.04ZAPS199B1.DQ164110	----------	---TCTGTTG	TGTGA-----	---------C	TCTGGTAA-C	TAGAGATCCC	TCAGACCATT	--TGTGGCAG	T-GTGGAA--	AATTTCTAGC
C.ZA.04.04ZAPS202B1.DQ093598	----------	---TCTGTTG	TGTGA-----	---------C	TCTGGTAA-C	TAGAGATCCC	TCAGACCTTT	---TTGGTAG	T-GTGGAA--	AATCTCTAGC
C.ZA.04.04ZAPS205B1.DQ093599	----------	---TCTGTTG	TGTGA-----	---------C	TCTGGTAA-C	TAGAGATCCC	TCAGACCCTT	---TTGGTAG	T-GTGGAA--	AATCTCTAGC
C.ZA.04.04ZAPS206B1.DQ164126	----------	---TCTGTTG	TGTGA-----	---------C	TCTGGTAA-C	TAGAGATCCC	TCAGACCAAT	--TTTGGTAG	T-GTGGAA--	AATCTCTAGC
C.ZA.04.04ZAPS208B1.DQ093603	----------	---TCTGTTG	TGTGA-----	---------C	TCTGGTAA-C	TAGAGATCCC	TCAGACCCTT	--TGTGGTAG	T-GTGGAA--	AATCTCTAGC
C.ZA.04.04ZAPS214B1.DQ093588	----------	---TCTGTTG	TGTGA-----	---------C	TCCGGTAA-C	TAGAGATCCC	TCAGACCCTT	---TTGGTAG	T-GTGGAA--	AATCTCTAGC
C.ZA.04.04ZAPS216B1.DQ093600	----------	---TCTGTTG	TGTGA-----	---------C	TCTGGTAA-C	TAGAGATCCC	TCAGACCCTT	--TGTGGTAG	T-GTGGAA--	AATCTCTAGC
C.ZA.04.04ZAPS217B1.DQ164119	----------	---TCTGTTG	TGTGA-----	---------C	TCTGGTAA-C	TAGAGATCCC	TCAGACCATT	---TTGGTAG	T-GTGGAA--	AATCTCTAGC

C.ZA.04.04ZASK031B2.AY901974	----------	---TCTGTTG	TGTGA-----	---------C	TCTGGTAA-C	TAGAGATCCC	TCAGACCCTT	--TGTGGTAG	T-GTGGAA--	AATCTCTAGC
C.ZA.04.04ZASK083B2.DQ093585	----------	---TCTGTTG	TGTGA-----	---------C	TCTGGTAA-C	TAGAGATCCC	TCAGACCATT	---TTGGTAG	T-GTGGAA--	AATCTCTAGC
C.ZA.04.04ZASK127B1.DQ011172	----------	---TCTGTTG	TGTGA-----	---------C	TCTGGTAA-C	TAGAGATCCC	TCAGACCCTT	---TTGGTAG	T-GTGGAA--	AATCTCTAGC
C.ZA.04.04ZASK128B1.DQ093586	----------	---TCTGTTG	TTTGA-----	---------C	TCTGGTAT-C	TAGAGATCCC	TCAGACCAAT	--TTTGGTAG	T-GTGGAA--	AATCTCTAGC
C.ZA.04.04ZASK131B1.DQ056412	----------	---TCTGTTG	TGTGA-----	---------C	TCTGGTAA-C	TAGAGATCCC	TCAGACCCTT	---TTGGTAG	T-GTGGAA--	AATCTCTAGC
C.ZA.04.04ZASK132B1.DQ093594	----------	---TCTGTTG	TGTGA-----	---------C	TCTGGTAA-C	TAGAGATCCC	TCAGACCAAT	---TTGGTAG	T-GTGGAA--	AATCTCTAGC
C.ZA.04.04ZASK135B1.DQ011166	----------	----------	----------	----------	----------	----------	----------	----------	----------	--TCTCTAGC
C.ZA.04.04ZASK136B1.DQ011177	----------	---TCTGTTG	TGTGA-----	---------C	TCTGGTAA-C	TAGAGATCCC	TCAGACCATT	--TGTGGTAG	T-GTGGAA--	AATCTCTAGC
C.ZA.04.04ZASK139B1.AY878072	----------	---TCTGTTG	TGTGA-----	---------C	TCTGGTAA-C	TAGAGATCCC	TCAGACCCTT	--TGTGGTAG	T-GTGGAA--	AATCTCTAGC
C.ZA.04.04ZASK142B1.DQ011167	----------	---TCTGTTG	TGTGA-----	---------C	TCTGGTAA-C	TAGAGATCCC	TCAGACCCTT	--TGTGGTAG	T-GTGGAA--	AATCTCTAGC
C.ZA.04.04ZASK145B1.AY901976	----------	---TCTGTTG	TGTGA-----	---------C	TCTGGTAA-C	TAGAGATCCC	TCAGACCCTT	--GTTGATAG	T-GTGGAA--	AATCTCTAGC
C.ZA.04.04ZASK146.AY772699	----------	---TCTGTTG	TGTGA-----	---------C	TCTGGTAA-C	TAGAGATCCC	TCAGACCTTC	--AGTGTTAG	T-GTGGAA--	AATCTCTAGC
C.ZA.04.04ZASK147B1.DQ164114	----------	----------	----A-----	---------C	TCTGGTAA-C	TAGAGATCCC	TCAGACCCAT	--TTTGGTAG	T-GTGGAA--	AATCTCTAGC
C.ZA.04.04ZASK148B1.DQ093590	----------	---TCTGTTG	TGTGA-----	---------C	TCTGGTAA-C	TAGAGATCCC	TCAGACCATT	---TTGGTAG	T-GTGGAA--	AATCTCTAGC
C.ZA.04.04ZASK150B1.DQ011178	----------	---TCTGTTG	TGTGA-----	---------C	TCTGGTAA-C	TAGAGATCCC	TCAGACTCCT	--TTTGGTAG	T-GTGAAA--	AATCTCTAGC
C.ZA.04.04ZASK151B1.AY901977	----------	---TCTGTTG	TGTGA-----	---------C	TCTGGTAA-C	TAGAGATCCC	TCAGACCCCT	--TGTGGTAG	T-GTGGAA--	AATCTCTAGC
C.ZA.04.04ZASK154B1.AY878071	----------	---TCTGTTG	TGTGA-----	---------C	TCTGGTAA-C	TAGAGATCCC	TCAGACCCTT	---TTGGTAG	T-GTGGAA--	AATCTCTAGC
C.ZA.04.04ZASK155B1.AY901978	----------	---TCTGTTG	TGTGA-----	---------C	TCTGGTAACT	AGAGATCCCT	CAGACCCCAA	--GTGGGTAA	GTGTGGAA--	AATCTCTAGC
C.ZA.04.04ZASK156B1.DQ011171	----------	---TCTGTTG	TGTGA-----	---------C	TCTGGTAA-C	TAGAGATCCC	TCAGACCCTT	--GTTGGTAG	T-GTGGAA--	AATCTCTAGC
C.ZA.04.04ZASK159B1.DQ011179	----------	---TCTGTTG	TGTGA-----	---------C	TCTGGTAA-C	TAGAGATCCC	TCAGACCTTT	---TTGGCCG	T-GTGGAA--	AATCTCCAGC
C.ZA.04.04ZASK160B1.DQ011173	----------	---TCTGTTG	TGTGA-----	---------C	TCTGGTAA-C	TAGAGATCCC	TCAGACCCTT	---TTGGTAG	T-GTGGAA--	AATCTCTAGC
C.ZA.04.04ZASK161B1.DQ011170	----------	---TCTGTTG	TGTGA-----	---------C	TCTGGTAA-C	TAGAGATCCC	TCAGACCCTT	--TGTGGTAG	T-GTGGAA--	AATCTCTAGC
C.ZA.04.04ZASK163B1.AY901979	----------	---TCTGTTG	TGTGA-----	---------C	TCTGGTAA-C	TAGAGATCCC	TCAGACCCTT	--TGTGGTAG	T-GTGGAA--	AATCTCTAGC
C.ZA.04.04ZASK164B1.DQ056405	----------	---TCTGTTG	TGTGA-----	---------C	TCTGGTAA-C	TAGAGATCCC	TCAGACCCTT	--GTTGGTAG	T-GTGGAA--	AATCTCTAGC
C.ZA.04.04ZASK165B1.DQ396387	----------	---TCTGTTG	TGTGA-----	---------C	TCTGGTAA-C	TAGAGATCCC	TCAGACCAAT	---TTGGTAG	T-GTGGAA--	AATCTCTAGC
C.ZA.04.04ZASK167B1.DQ164127	----------	---TCTGGTG	TGTGA-----	---------C	TCTGGTAT-C	TAGAGATCCC	TCAGACCAAC	--TTTGGTAG	T-GTGGAA--	AATCTCTAGC
C.ZA.04.04ZASK168B1.AY878058	--TCTGTTGT	GTGACTCTGG	TAATA-----	---------C	TCTGGTAA-C	TAGAGATCCC	TCAGACCCTT	--GTTGGTTG	C-GTGGAA--	AATCTCTAGC
C.ZA.04.04ZASK169B1.DQ396381	----------	---TCTGTTG	TATGA-----	---------C	TCTGGTAA-C	TAGAGATCCC	TCAGACCAAT	--TTTGGTAG	T-GTGGAA--	AATCTCTAGC
C.ZA.04.04ZASK170B1.DQ093595	----------	---TCTGTTG	TGTGA-----	---------C	TCTGGTAA-C	TAGAGATCCC	TCAGACCAAT	--TGTGGTAG	T-GTGGAA--	AATCTCTAGC
C.ZA.04.04ZASK171B1.DQ351217	----------	---TCTGTTG	TGTGA-----	---------C	TCTGGTAT-C	TAGAGATCCC	TCAGACCAAC	--TTTGGTAG	T-GTGGAA--	AATCTCTAGC
C.ZA.04.04ZASK172B1.DQ369998	----------	---TCTGTTG	TGTGA-----	---------C	TCTGGTAA-C	TGGAGATCCC	TCAGACCCTT	--GTTGGTAG	T-GTGGAA--	AATCTCTAGC
C.ZA.04.04ZASK173B1.DQ093604	----------	---TCTGTTG	TGTGA-----	---------C	TCTGGTAA-C	TAGAGATCCC	TCAGACCCTT	--TGTGGTAG	T-GTGGAA--	AATCTCTAGC
C.ZA.04.04ZASK174B1.AY901980	----------	---TCTGTTG	TGTGA-----	---------C	TCTGGTAA-C	TAGAGATCCC	TCAGACCCTT	--TTTGGTAG	T-GTGGAA--	AATCTCTAGC
C.ZA.04.04ZASK175B1.DQ164129	----------	---TCTGTTG	TGTGA-----	---------C	TCTGGTAA-C	TAGAGATCCC	TCAGACCTT-	--TTTGGTTG	T-GTGGAA--	AATCTCTAGC
C.ZA.04.04ZASK176B1.DQ056416	----------	---TCTGTTG	TGTGA-----	---------C	TCTGGTAA-C	TAGAGATCCC	TCAGACCAAC	--TTTGGTAG	T-GTGGAA--	AATCTCTAGC
C.ZA.04.04ZASK178B1.DQ093587	----------	---TCTGTTG	TGTGA-----	---------C	TCTGGTAA-C	TAGAGATCCC	TCAGACCCTT	--GTTGGTAG	T-GTGGAA--	AATCTCTAGC
C.ZA.04.04ZASK180B1.AY878059	----------	---TCTGTTG	TGTGA-----	---------C	TCTGGTAA-C	TAGAGATCCC	TCAGATCCTT	--TTTGGTAG	T-GTGGAA--	AATCTCTAGC
C.ZA.04.04ZASK181B1.AY878062	----------	---TCTGATG	TGTGA-----	---------C	TCTGGTAA-C	TAGAGATCCC	TCAGACCCTT	---GTGGTAG	T-GTGGAA--	AATCTCTAGC
C.ZA.04.04ZASK182B1.AY878054	----------	---TCTGTTG	TGTGA-----	---------C	TCTGGTAA-C	TAGAGATCCC	TCAGACCCTT	--TGTGGTAG	T-GTGGAA--	AATCTCTAGC
C.ZA.04.04ZASK183B1.AY878055	----------	---TCTGTTG	TGTGA-----	---------C	TCTGGTAA-C	TAGAGATCCC	TCAGACCCTT	--TTTGGTAG	T-GTGGAA--	AATCTCTAGC
C.ZA.04.04ZASK184B1.DQ056418	----------	---TCTGTTG	TGTGA-----	---------C	TCTGGTAA-C	TAGAGATCCC	TCAGACCAAC	--TTCGGTAG	T-GTGGAA--	AATCTCTAGC
C.ZA.04.04ZASK185B1.DQ011174	----------	---TCTGTTG	TGTGA-----	---------C	TCTGGTAA-C	TAGAGATCCC	TCAGACCCTT	--TTTGGTAG	T-GTGGAA--	AATCTCTAGC
C.ZA.04.04ZASK190B1.DQ056409	----------	---TCTGTTG	TGTGA-----	---------C	TCTGGTAA-C	TAGAGATCCC	TCAGACCAGT	--TTTGGCAG	T-GTGGA---	AATCTCTAGC
C.ZA.04.04ZASK191B1.DQ369993	----------	---TCTGTTG	TGTGG-----	---------C	TCTGGTAA-C	TAGAGATCCC	TCAGACTATC	--TTTAGTTG	T-GTGGAA--	AATCTCTAGC
C.ZA.04.04ZASK192B1.DQ396382	----------	---TCTGTTG	TGTGA-----	---------C	TCTGGTAA-C	TAGAGATCCC	TCAGACCATT	---TTGGTAG	T-GTGGAA--	AATCTCTAGC
C.ZA.04.04ZASK193B1.DQ396396	----------	---TCTGTTG	TGTGG-----	---------C	TCTGGTAA-C	TAGGGATCCC	TCAGACCTTT	----TGGTAG	T-GTGGAA--	AATCTCTAGC
C.ZA.04.04ZASK196B1.DQ056413	----------	---TCTGTTG	TGTGA-----	---------C	TCTGGTAA-C	TAGAGATCCC	TCAGACCCTT	--GTTGGTAG	T-GTGGAA--	AATCTCTAGC
C.ZA.04.04ZASK200B1.DQ396383	----------	---TCTGTTG	TGTGG-----	---------C	TCTGGTAA-C	TAGAGATCCC	TCAGACCCTT	--TTTGGTTG	T-GTGGAA--	AATCTCTAGC
C.ZA.04.04ZASK201B1.DQ396397	----------	---TCTGTTG	TGTGG-----	---------C	TCTGGTAA-C	TAGAGATCCC	TCAGACCAAC	--TTTGGTAG	T-GTGGAA--	AATCTCTAGC
C.ZA.04.04ZASK202B1.DQ011180	----------	---TCTGTTG	TGTGA-----	---------C	TCTGGTAA-C	TGGAGATCCC	TCAGACCCTT	--TTTGGTTG	T-GTGGAA--	AATCTCTAGC
C.ZA.04.04ZASK204B1.DQ056414	----------	---TCTGTTG	TGTGA-----	---------C	TCTGGTAA-C	TAGAGATCCC	TCAGACCAAT	--TGTGGTAG	T-GTGGAA--	AATCTCTAGC
C.ZA.04.04ZASK206B1.DQ056415	--TCTGTTGT	GTGACTCTGG	TAATA-----	---------C	TCTGGTAA-C	TAGAGATCCC	TCAGACCCT-	--AGTGGTAT	T-GTGGAA--	AATCTCTAGC
C.ZA.04.04ZASK208B1.DQ056406	----------	---TCTGTTG	TGTGA-----	---------C	TCTGGTAA-C	TAGAGATCCC	TCAGACCCTT	--TTTGGTAG	T-GTGGAA--	AATCTCTAGC
C.ZA.04.04ZASK217B1.DQ056417	----------	---TCTGTTG	TGTGA-----	---------C	TCTGGTAA-C	TAGAGATCCC	TCAGACCTTT	---TTGGCAG	T-GTGGAA--	AATCTCTAGC
C.ZA.04.04ZASK234B1.DQ093605	----------	---TCTGTTG	TGTGA-----	---------C	TCTGGTAA-C	TAGAGATCCC	TCAGACCATT	---TTGGTAG	T-GTGGAA--	AATCTCTAGC
C.ZA.04.CAP30_5w_F4.GQ999973	TAGTGTGTGC	CCGTCTGTTG	TGTGA-----	---------C	TCTGGTAA-C	TAGAGATCCC	TCAGACCCTT	--TTTGGTAG	T-GTGGAA--	AATCTCTAGC
C.ZA.04.CAP61_8w_F3.GQ999975	TAGTGTGTGC	CCGTCTGTTG	TGTGA-----	---------C	TCTGGTAA-C	TAGAGATCCC	TCAGACCAAT	--GTGGTAGT	G-TGGAA---	AATCTCTAGC
C.ZA.04.SK133B1.AY772698	----------	---TCTGTTG	TGTGA-----	---------C	TCTGGTAA-C	TAGAGATCCC	TCAGACTACT	--TGTGGCTG	T-GTGGAA--	AATCTCTAGC
C.ZA.04.SK134B1.AY703909	----------	---TCTGTTG	TGTGA-----	---------C	TCTGGTAA-C	TAGAGATCCC	TCAGACCCTT	--TGTGGTAG	T-GTGGAA--	AATCTCTAGC
C.ZA.04.SK140B1.AY901973	----------	---TCTGTTG	TGTGA-----	---------C	TCTGGTAA-C	TAGAGATCCC	TCAGACCATT	---TTGGTAG	T-GTGGAA--	AATCTCTAGC
C.ZA.04.SK143B1.AY703910	----------	---TCTGTTG	TGTGA-----	---------C	TCTGGTAA-C	TAGAGATCCC	TCAGACCCTT	---GTGGTAG	T-GTGGAA--	AATCTCTAGC
C.ZA.04.SK144B1.AY703911	----------	---TCTGTTG	TGTGA-----	---------C	TCTGGTAA-C	TAGAGATCCC	TCAGACCCTT	---TTGGTAG	T-GCGGAA--	AATCTCTAGC
C.ZA.05.05ZAPSK240B1.DQ369991	----------	---TCTGTTG	TGTGG-----	---------C	TCTGGTAA-C	TAGAGATCCC	TCAGACCCTT	---TTGGTAG	T-GTGGAA--	AATCTCTAGC
C.ZA.05.05ZASK243B1.DQ396372	----------	---TCTGTTG	TGTGG-----	---------C	TCTGGTAA-C	TAGAGATCCC	TCAGACCATT	---TTGGTAG	T-GTGGAA--	AATCTCTAGC
C.ZA.05.05ZASK244B1.DQ369992	----------	---TCTGTTG	TGTGG-----	---------C	TCTGGTAA-C	TAGAGATCCC	TCAGACCATT	---TTGGTAG	T-GTGGAA--	AATCTCTAGC
C.ZA.05.05ZASK245B1.DQ369982	----------	---TCTGTTG	TGTGG-----	---------C	TCTGGTAA-C	TAGAGATCCC	TCAGACCAAT	--TTTGGTAG	T-GTGGAA--	AATCTCTAGC
C.ZA.05.05ZASK246B1.DQ369983	----------	---TCTGTTG	TGTGG-----	---------C	TCTGGTAA-C	TAGAGATCCC	TCAGACCCTT	--TTTGGTAG	T-GTGGAA--	AATCTCTAGC
C.ZA.05.05ZASK247B1.DQ369994	----------	---TCTGTTG	TGTGA-----	---------C	TCTGGTAA-C	TAGAGATCCC	TCAGATCATT	---TTGGTAG	T-GTGGAA--	AATCTCTAGC
C.ZA.05.CAP174_4w.GQ999981	TAGTGTGTGC	CCGTCTGTTG	TGTGA-----	---------C	TCTGGTAA-C	TAGAGATCCC	TCAGACCAAC	--TTTGGTAG	T-GTGGAA--	AATCTCTAGC
C.ZA.05.CAP206_8w_F1.GQ999982	TAGTGTGTGC	CCGTCTGTTG	TGTGA-----	---------C	TCTGGTAA-C	TAGAGATCCC	TCAGACCCTT	--TGTGGTGT	G-TGGAA---	AATCTCTAGC
C.ZA.05.CAP210_5w.GQ999983	TAGTGTGTGC	CCGTCTGTTG	TGTGA-----	---------C	TCTGGTAG-C	TAGAGATCCC	TCAGATCCTT	--TTGTCAGA	G-TGGAA---	AATCTCTAGC
C.ZA.05.CAP228_8w_F2.GQ999984	----------	----------	----------	----------	----------	----------	----------	T-TTTGGTAG	T-GTGGAA--	AATCTCTAGC
C.ZA.05.CAP229_7w.GQ999985	-AGTGTGTGC	CCGTCTGTTG	TGTGA-----	---------C	TCTGGTAA-C	TAGAGATCCC	TCAGACCCTT	--TTTGGTAG	T-GTGGAA--	AATCTCTAGC
C.ZA.05.CAP239_5w_F1.GQ999991	TAGTGTGTGC	CCCTTTGATG	TGTGA-----	---------C	TCTGGTAA-C	TAGAGATCCC	TCAGACCAAT	--TGTGGTAG	T-GTGGAA--	AATCTCTAGC
C.ZA.05.CAP244_8w_F1.GQ999986	TAGTGTGTGC	CCGTCTGTTG	TGTGA-----	---------C	TCTGGTAA-C	TAGAGATCCC	TCAGACCCTT	--TTGGTAGT	G-TGGAA---	AATCTCTAGC
C.ZA.05.CAP248_9w.GQ999987	-AGTGTGTGC	CCGTCTGTTG	TGTGA-----	---------C	TCTGGTAA-C	TAGAGATCCC	TCAGACCCTT	--GTTGGTAG	T-GTGGAA--	AATCTCTAGC
C.ZA.05.CAP255_8w_F1.GQ999988	----------	----------	----------	----------	GCGCTAAC-C	TAGAGATCCC	TCAGACCTTT	--TGTGGTAG	T-GTGGAA--	AATCTCTAGC
C.ZA.05.CAP256_6w.GQ999989	TAGTGTGTGC	CCGTCTGTTG	TGTGA-----	---------C	TCTGGTAA-C	TAGAGAACCC	TCAGACCATT	--TGTGGTAG	T-GTGGAA--	AATCTCTAGC
C.ZA.05.CAP257_7w_F1.GQ999990	TAGTGTGTGC	CCGTCTGTTG	TGTGA-----	---------C	TCTGGTAA-C	TAGAGATCCC	TCAGACCAGT	--TTGGTAGT	G-CGGAA---	AATCTCTAGC
C.ZA.05.CAP45_5w_F1.GQ999974	TAGTGTGTGC	CCGTCTGTTG	TGTGA-----	---------C	TCTGGTAA-C	TAGAGATCCC	TCAGACCCTT	--TTTGGTAG	T-GTGGAA--	AATCTCTAGC
C.ZA.05.CAP63_5w_F4.GQ999976	TAGTGTGTGC	CCGTCTGTTG	TGTGA-----	---------C	TCTGGTAA-C	TAGAGATCCC	TCAGATCCTT	T--TTGGTAG	T-GTGGAA--	AATCTCTAGC
C.ZA.05.CAP65_6w.GQ999977	TAGTGTGTGC	CCGTCTGTTG	TGTGA-----	---------C	TCTGGTAA-C	TAGAGATCCC	TCAGACCAAC	--TTTGGTAG	T-GTGGAA--	AATCTCTAGC
C.ZA.05.CAP84_3w_F2.GQ999978	TAGTGTGTGC	CCGTCTGTTG	TGTGA-----	---------C	TCTGGTAA-C	TAGAGATCCC	TCAGACCTCT	--TTGGTAGT	G-TGGAA---	AATCTCTAGC
C.ZA.05.CAP85_5w_F1.GQ999979	TAGTGTGTGC	CCGTCTGTTG	TGTGA-----	---------C	TCTGGTAA-C	TAGAGATCCC	TCAGACCTTT	--TTGGTAGT	G-TGGAA---	AATCTCTAGC
C.ZA.05.CAP88_5w_F2.GQ999980	TAGTGTGTGC	CCGTCTGTTG	TGTGA-----	---------C	TCTGGTAA-C	TAGAGATCCC	G---------	----------	--GAA-----	AATCTCTAGC
C.ZA.05.CAP8_3w_F2.GQ999972	TAGTGTGTGC	CCGTCTGTTG	TGTGA-----	---------C	TCTGGTAA-C	TAGAGATCCC	TCAGACCCTT	--TGTGGTAG	T-GTGGAA--	AATCTCTAGC
C.ZA.07.DEMC07ZA011.JX140664	----------	----------	----------	----------	----------	----------	----------	----------	----------	----------
C.ZA.08.DEMC08ZA011.JX140666	----------	----------	----------	----------	----------	----------	----------	----------	----------	----------
C.ZA.09.DEMC09ZA008.JX140667	----------	----------	----------	----------	----------	----------	----------	----------	----------	----------
C.ZA.09.DEMC09ZA009.JX140668	----------	----------	----------	----------	----------	----------	----------	----------	----------	----------
C.ZA.10.DEMC10ZA001.JX140669	----------	----------	----------	----------	----------	----------	----------	----------	----------	----------
C.ZA.90.pZAC_R3714.JN188292	TAGTGTGTGC	CCGTCTGTTG	TGTGA-----	---------C	TCTGGTAA-C	TAGAGATCCC	TCAGACCCTA	--TTTGGTAG	T-GTGGAA--	AATCTCTAGC
C.ZA.97.97ZA003.AY118165	----------	----------	----------	----------	----------	----------	----------	----------	----------	----------
C.ZA.97.97ZA009.AY118166	----------	----------	----------	----------	----------	----------	----------	----------	----------	----------
C.ZA.97.97ZA012.AF286227	----------	----------	----------	----------	----------	----------	----------	----------	----------	----------
C.ZA.98.98ZA445.AY158533	----------	----------	----------	----------	----------	----------	----------	----------	----------	----------
C.ZA.98.98ZA502.AY158534	----------	----------	----------	----------	----------	----------	----------	----------	----------	----------
C.ZA.98.98ZA528.AY158535	----------	----------	----------	----------	----------	----------	----------	----------	----------	----------
C.ZA.98.TV001.AY162223	TAGTGTGTGC	CCATCTGTTG	TGTGA-----	---------C	TCTGGTAA-C	TAGAGATCCC	TCAGACCCTT	--TGTGGTAG	T-GTGGAA--	AATCTCTAGC
C.ZA.98.TV002.AY162224	TAGTGTGTGC	CCATCTGTTG	TGTGACTCTG	GTAA-----C	TCTGGTAA-C	TAGAGATCCC	TCAGACCCTT	--TGTGGTAG	T-GTGGAA--	AATCTCTAGC
C.ZA.98.TV012.AY162225	TAGTGTGTGC	CCGTCTGTTG	TGTGA-----	---------C	TCTGGTAA-C	TAGAGATCCC	TCAGACCACT	--TGTGGTGT	--GTGGAA--	AATCTCTAGC
C.ZA.99.99ZACM9.AF411967	TAGTGTGTGC	CCGTCTGTTG	TGTGA-----	---------C	TCTGGTAA-C	TAGAGATCCC	TCAGACCCTT	--TTTGGTAG	T-GTGGAA--	AATCTCTAGC
C.ZA.99.99ZALT21.EU293446	TAGTGTGTGC	CCGTCTGTTG	TGTGA-----	---------C	TCTGGTAA-C	TAGAGATCCC	TCAGACCATT	---TTGGTAG	T-GTGGAA--	AATCTCTAGC
C.ZA.99.99ZALT39.EU293447	TAGTGTGTGC	CCGTCTGTTG	TGTGA-----	---------C	TCTGGTAA-C	TAGAGATCCC	TCAGACCCTT	--TGTGGTAG	T-GTGGAA--	AATCTCTAGC
C.ZA.99.99ZALT42.EU293448	TAGTGTGTGC	CCGTCTGTTG	TGTGA-----	---------C	TCTGGTAA-C	TAGAGATCCC	TCAGACCAAT	---TTGGTAG	T-GTGGAA--	AATCTCTAGC

```
C.ZA.99.99ZALT45.EU293449      TAGTGTGTGC CCGTCTGTTG TGTGA----- ---------C TCTGGTAA-C TAGAGATCCC TCAGACCCTT --CGTGGTAG T-GTGGAA-- AATCTCTAGC
C.ZA.99.99ZALT46.EU293450      TAGTGTGTGC CCGTCTGTTG TGTGA----- ---------C TCTGGTAA-C TAGAGATCCC TCAGACCCTT ---TTTGGTG T-GTGGAA-- AATCTCTAGC
C.ZA.99.99ZALT4.EU293444       TAGTGTGTGC CCGTCTGTTG TGTGA----- ---------C TCTGGTAA-C TAGAGATCCC TCAGACCAAC --TTTGGTAG T-GTGGAA-- AATCTCTAGC
C.ZA.99.99ZALT5.EU293445       TAGTGTGTGC CCGTCTGTTG TGTGA----- ---------C TCTGGTAA-C TAGAGATCCC TCAGACCAAT --TTTGGCAG T-GTGGAA-- AATCTCTAGC
C.ZA.99.99ZATM10.AY228556      TAGTGTGTGC CCGTCTGTTG TGTGA----- ---------C TCTGGTAA-C TAGAGATCCC TCAGACCCTT --TGTGGTAG T-GTGGAA-- AATCTCTAGC
C.ZA.99.ZASW7.AF411966         TAGTGTGTGC CCGTCTGTTG TGTGA----- ---------C TCTGGTAA-C TAGAGATCCC TCAGACCCTT --TTTGGTAG T-GTGGAA-- AATCTCTAGC
C.ZM.02.02ZM108.AB254141       TAGTGTGTGC CCGTCTGTTG TGTGA----- ---------C TCTGGTAA-C TAGAGATCCC TCAGACCTTT --TTTGGTAG T-GTGGAA-- AATCTCTAGC
C.ZM.02.02ZM110.AB254142       TAGTGTGTGC CCGTCTGTTG TGTGA----- ---------C TCTGGTAA-C TAGAGATCCC TCAGACCTTT ---GTGTTAG T-GTGGAA-- AATCTCTAGC
C.ZM.02.02ZM114.AB254146       TAGTGTGTGC CCGTCTGTTG TGTGA----- ---------C TCTGGTAA-C TAGAGATCCC TCAGACCAAT ---TTGGTAG T-GTGGAA-- AATCTCTAGC
C.ZM.02.02ZM115.AB254148       TAGTGTGTGC CCGTCTGTTG TGTGA----- ---------C TCTGGTAA-C TAGAGATCCC TCAGACCCTT --GT-GGCAT T-GTGGAAA- AATCTCTAGC
C.ZM.02.02ZMBC.AB254149        TAGTGTGTGC CCGTCTGTTG TGTGA----- ---------C TCTGGTAA-C TAGAGATCCC TCAGACCTTT ---GTGGTAG T-GCGGAA-- AATCTCTAGC
C.ZM.02.02ZMDB.AB254153        TAGTGTGTGC CCGTCTGTTG TGTGA----- ---------C TCTGGTAA-C TAGAGATCCC TCAGACCAAT ---TTGGTAG T-GTGGAA-- AATCTCTAGC
C.ZM.02.02ZMJC.AB254155        TAGTGTGTGC CCATCTGTTG TGTGA----- ---------C TCTGGTAA-C TAGAGATCCC TCAAACCAAA --AGGTGTGC --GTGGAA-- AATCTCTAGC
C.ZM.03.ZM246F_flA10.FJ496186  ---------- ---------- ---------- ---------- --TGGTAA-C TAGAGATCCC TCAGACCATT --TTGG-CAG T-GTGGAA-- AATCTCTAGC
C.ZM.03.ZM247F_flA1.FJ496195   ---------- ---------- ---------- ---------- --TGGTAA-C TAGAGATCCC TCAGACCATT --TTGG-TAG T-GTGGAA-- AATCTCTAGC
C.ZM.03.ZM249M_flC1.FJ496209   ---------- ---------- ---------- ---------- --TGGTAA-C TAGAGATCCC TCAGACCTTT ---TAGGTAG T-GTGGAA-- AATCTCTAGC
C.ZM.89.ZAM18.AB485645         TAGTGTGTGC CCGTCTGTTG TGTGA----- ---------C TCTGGTAA-C TAGAGATCCC TCAGACCCTT --TGTGGTAG T-GTGAAA-- AATCTCTAGC
C.ZM.96.96ZM651.AF286224       ---------- ---------- ---------- ---------- ---------- ---------- ---------- ---------- ---------- ----------
C.ZM.96.96ZM751.AF286225       ---------- ---------- ---------- ---------- ---------- ---------- ---------- ---------- ---------- ----------
C.ZM.x.HIV1084i.AY805330       TAGTGTGTGC CCGTCTGTTG TGTGA----- ---------C TCTGGTAA-C TAGAGATCCC TCAGACCCGT --TGTGGTAG T-GTGGAA-- AATCTCTAGC
D.CD.83.ELI.K03454             TAGTGTGTGC CCGTCTGTTG TGTGA----- ---------C TCTGGTAA-C TAGAGATCCC TCAGACCCCT --TTAGTCAG A-GTGGAA-- AATCTCTAGC
D.CD.83.NDK.M27323             TAGTGTGTGC CCGTCTGTTG TGTGA----- ---------C TCTGGTAA-C TAGAGATCCC TCAGATCCGT --TTAGTCCG A-GTGAAA-- AATCTCTAGC
D.CD.84.84ZR085.U88822         TAGTGTGTGC CCGTCTGTTT TGTGA----- ---------C TCTGGTAA-C TAGAGATCCC TCAGACAGCT --TTAGTCCG G-GTGGAA-- AATCTCTAGC
D.CD.85.Z2Z6_Z2_CDC_Z34.M22639 TAGTGTGTGC CCGTCTGTTG TGTGA----- ---------C TCTGGTAA-C TAGAGATCCC TCAGACCCCT --TTAGTCAG A-GTGGAA-- AATCTCTAGC
D.CM.10.DEMD10CM009.JX140670   ---------- ---------- ---------- ---------- ---------- ---------- ---------- ---------- ---------- ----------
D.KE.93.MB2059.AF133821        CAGTGTGTGC CCATCTGTTG TGTGA----- ---------C TCTGGTAA-C TAGAGATCCC TCAGACCTCT --TTAGTCAG T-GTAAAA-- AATCTCTAGC
D.KE.97.ML415_2.AY322189       TAGTGTGTGC CCGTCTGTTG TGTGA----- ---------C TCTGGTAA-C TAGAGATCCC TCAGACCTCT --GTAGTCAG T-GTGAA--- AATCTCTAGC
D.KR.04.04KBH8.DQ054367        TAGTGTATGC CCGTCTGTTG TGTAA----- ---------C TCTGGTAA-C TAGAGATCCC TCAGACCTCG --TTGTCAG T-GTAAAA-- AATCTCTCGC
D.SN.90.SE365.AB485648         TAGTGTGTGC CCATCTGTTG TGTGA----- ---------C TCTGGTAA-C TAGAGATCCC TCAGACCCTT --TTAGTCAG T-GTGGAA-- AATCTCTAGC
D.UG.05.p190049.JX236668       TAGTGTGTGC CCGTCTGTTG TATGA----- ---------C TCTGGTAA-C TAGAGATCCC TCAGACCCTT --TTAGTCAG T-GTGAA--- AATCTCTAGC
D.UG.07.p191647.JX236670       TAGTGTGTGC CCATCTGTTG TGTGA----- ---------C TCTGGTAA-C TAGAGATCCC TCAGACCTCT --TTGGTCAG T-GTAGAA-- AATCTCTAGC
D.UG.07.p191882.JX236673       TAGTGTGTGC CCGTCTGTTG TGTGA----- ---------C TCTGGTAA-C TAGAGATCCC TCAGACCTCT --TTAGTCAG T-GTGGAA-- AATCTCTAGC
D.UG.07.pSC191727.JX236679     TAGTGTGTGC CCATCTGTTG TGTGA----- ---------C TCTGGTAAAC TAGAGATCCC TCAGACCTCT --TTAGTCAG T-GTGGAA-- AATCTCTAGC
D.UG.08.p191859.JX236672       TAGTGTGTGC CCATCAATTG TGTGA----- ---------C TCTGGTAT-C TAGAGATCCC TCAGACCTCT --TTAGTCCG G-GTGGAA-- AATCTCTAGC
D.UG.91.UG270.AB485650         TAGTGTGTGC CCATCTGTTG TATGA----- ---------C TCTGGTAA-C TAGAGATCCC TCAGACCTCT --TTAGTCAG T-GTGGAA-- AATCTCTAGC
D.UG.92.92UG001.AJ320484       TAGTGTGTGC CCGTCTGTTG TGTGA----- ---------C TCTGGTAA-C TAGAGATCCC TCAGACCTCT --TTAGTCAG T-GTGGAA-- AATCTCTAGC
D.UG.94.94UG114.U88824         ---------- ---------- ---------- ---------- ---------- ---------- ---------- ---------- ---------- ----------
D.ZA.84.R2.AY773338            ---------- ---------- ---------- ---------- ---------- ---------- ---------- ---------- ---------- ----------
D.ZA.85.R286.AY773340          ---------- ---------- ---------- ---------- ---------- ---------- ---------- ---------- ---------- ----------
D.ZA.86.R482.AY773341          ---------- ---------- ---------- ---------- ---------- ---------- ---------- ---------- ---------- ----------
D.ZA.90.R1.EF633445            ---------- ---------- ---------- ---------- ---------- ---------- ---------- ---------- ---------- ----------
F1.AR.02.ARE933.DQ189088       ---------- ---------- ---------- ---------- ---------- ---------- ---------- ---------- ---------- ----------
F1.BE.93.VI850.AF077336        ---------- ---------- ---------- ---------- ---------- ---------- ---------- ---------- ---------- ----------
F1.BR.02.02BR082.FJ771006      TAGTGTGTGC CCGTCTGTTG TGTGA----- ---------C TCTGGTAA-C TAGAGATCCC TCAGACCATT --TTAGTCAG T-GTGGAA-- AATCTCTAGC
F1.BR.02.02BR170.FJ771007      ---------- --GTCTGTTG TGTGA----- ---------C TCTGGTAA-C TAGAGATCCC TCAGACCATT --TTAGTCAG T-GTGGAA-- AATCTCTAGC
F1.BR.06.06BR564.FJ771008      TAATGTGTGC CCGTCTGTTG TGTGA----- ---------C TCTGGTAA-C TAGAGATCCC TCAGACCATC --TTAGTCAG T-GTGGAA-- AATCTCTAGC
F1.BR.06.06BR579.FJ771009      TAGTGTGTGC CCGTCTGTTG TGTGA----- ---------C TCTGGTAA-C TAGAGATCCC TCAGACCTTT ---TAGTCAG T-GTGGAA-- AATCTCTAGC
F1.BR.07.07BR844.FJ771010      TAGTGTGTGC CCGTCTGTTG TGTGA----- ---------C TCTGGTAA-C TAGAGATCCC TCAGACCATT --TTAGTCAG T-GTGGAA-- AATCTCTAGC
F1.BR.89.BZ126.AY173957        ---------- ---------- ---------- ---------- ---------- ---------- ---------- ---------- ---------- ---------C
F1.BR.93.93BR020_1.AF005494    ---------- ---------- ---------- ---------- ---------- ---------- ---------- ---------- ---------- ----------
F1.DE.x.MVP_30846.EU446022     ---------- ---------- ---------- ---------- ---------- ---------- ---------- ---------- ---------- ----------
F1.ES.02.ES_X845_4.FJ670516    TAAGGGGTGC CCGTCTGTTG TGTGA----- ---------C TCTGGTAA-C TAGAGATCCC TCAGACCATT --TTAGTCAG A-GTGTAA-- AATCTCTAGC
F1.ES.11.DEMF110ES001.JX140671 ---------- ---------- ---------- ---------- ---------- ---------- ---------- ---------- ---------- ----------
F1.ES.x.P1146.DQ979023         ---------- ---------- ---------- ---------- ---------- ---------- ---------- ---------- ---------- ----------
F1.ES.x.X1093_2.DQ979025       TAGTGTGTGC CCGTCTGTTG TGTGA----- ---------C TCTGGTAA-C TAGAGATCCC TCAGACCATT --TTAGTCAG T-GTGGAA-- AATCTCTAGC
F1.ES.x.X1670.DQ979024         -------TGC CCGTCTGTTG TGTGA----- ---------C TCTGGTAA-C TAGAGATCCC TCAGACCTTT --TTAGTCCG T-GTGGAA-- AATCTCTAGC
F1.FI.93.FIN9363.AF075703      ---------- ---------- ---------- ---------- ---------- ---------- ---------- ---------- ---------- ----------
F1.RO.96.BCI_R07.AB485658      TAGTGTGTGC CCGTCTGTTG TTTGA----- ---------C TCTGGTAA-C TAGAGATCCC TCAGACCATT --TTAGTCAG T-GTGGAA-- AATCTCTAGC
F1.RU.08.D88_845.GQ290462      TAGTGTGTGC CCGTCTGTTG TGTGA----- ---------C TCTGGTAA-C TAGAGATCCC TCAGACCATT --TTAGTCAG T-GTGGAA-- AATCTCTAGC
F2.CM.10.DEMF210CM007.JX140673 ---------- ---------- ---------- ---------- ---------- ---------- ---------- ---------- ---------- ----------
G.BE.96.DRCBL.AF084936         TAGTGTGTGC CCGTCTGTTG TGTGA----- ---------C TCTGGTAA-C TAGAGATCCC TCAGACCACT --CTAGATAG T-GTAAA--- AATCTCTAGC
G.CM.01.A1786.FJ389367         TAGTGTGTGC CCGTCTGTTG TGTGA----- ---------C TCTGGTAA-C TAGAGATCCC TCAGACCACT --CTAGACAG T-GTAAA--- AATCTCTAGC
G.CM.04.178_15.FJ389363        TAGTGTGTGC CCGTCTGTTG TGCGA----- ---------C TCTGGTAA-C TAGAGATCCC TCAGACCACT --CTAGATAG T-GTAAA--- AATCTCTAGC
G.CM.04.314_40.FJ389364        TAGTGTGTGC CCGTCTGTTT TGTGA----- ---------C TCTGGTAA-C TAGAGATCCC TCAGACCACT --CTARACAG T-GTAAA--- AATCTCTAGC
G.CM.04.515_28.FJ389365        TAGTGTGTGC CCGTCTGTTG TGTGA----- ---------C TCTGGTAA-C TAGAGATCCC TCAGACCACT --CTAGACTG A-GTAAA--- AATCTCTAGC
G.CM.04.944_5.FJ389366         TAGTGTGTGC CCGTCTGTTG TGTGA----- ---------C TCTGGTAA-C TAGAGATCCC TCAGACCACT --CRAGACAC T-GTAAA--- AATCTCTAGC
G.CM.10.DEMG10CM008.JX140676   ---------- ---------- ---------- ---------- ---------- ---------- ---------- ---------- ---------- ----------
G.CM.96.96CMABB55.AY772535     TAGTGTGTGC CCGTCTGTTG TGTGA----- ---------C TCTGGTAA-C TAGAGATCCC TCAGACCACT --CTAGACAG T-GTAAA--- AATCTCTAGC
G.CN.06.sh52.HM067749          TAGTGTGGGC CCGTCTGTTG TGTGA----- ---------C TCTGGTAA-C TAGAGATCCC TCAGGACCAC T-CTAGACAG T-GTAAA--- AATCTCTAGC
G.CU.99.Cu74.AY586547          TGGTGTGTGC CCGTCTGTTG TGTGA----- ---------C TCTGGTAA-C TAGAGATCCC TCAGACCACT --CTAGACGG T-GTAAA--- AATCTCTAGC
G.CU.99.Cu85.AY586548          TAGTGTGTGC CCGTCTGTTG TGTGA----- ---------C TCTGGTAA-C TAGAGATCCC TCAGACCACT --CTAGAYAG T-GTAAA--- AATCTCTAGC
G.CU.99.Cu87.AY586549          TAGTGTGTGC CCGTCTGTTG TGTGA----- ---------C TCTGGTAA-C TAGAGATCCC TCAGACCACT --CTAGACAG T-GTAAA--- AATCTCTAGC
G.ES.05.P962.EU786670          -------TGC CCGTCTGTTG TGTGA----- ---------C TCTGGTAA-C TAGAGATCCC TCAGACCACT --CTAGACAG A-ATAAA--- AATCTCTAGC
G.ES.08.P1981_2.FJ670530       TAAGGGGTGC CCGTCTGTTG TGTGA----- ---------C TCTGGTAA-C TAGAGATCCC TCAGACCACT --CTAGGTA- --GTGTAAA- AATCTCTAGC
G.ES.09.X2634_2.GU362882       TAGTGTGTGC CCGTCTGTTG TGTGA----- ---------C TCTGGTAA-C TAGAGATCCC TCAGACCACT --CTAGTGA- --GTGTAAA- AATCTCTAGC
G.ES.99.X138.AF450098          ---------- -------TTG TGTGA----- ---------C TCTGGTAA-C TAGAGATCCC TCAGACCACT --CTAGATAG T-GTAAA--- AATCTCTAGC
G.GH.03.03GH175G.AB287004      TAGTGTGTGC CCGTCTGTTG TGTGA----- ---------C TCTGGTAA-C TAGAGATCCC TCAGACCACT --CTAGACAG T-GTAAA--- AATCTCTAGC
G.KE.93.HH8793_12_1.AF061641   ---------- ---------- ---------- ---------- ---------- ---------- ---------- ---------- --GGCTTA-- AATCTCTAGC
G.NG.92.92NG083_JV10832.U88826 ---------- ---------- ---------- ---------- ---------- ---------- ---------- ---------- ---------- ----------
G.PT.x.PT2695.AY612637         TAGTGTGTGC CCGTCTGTTG TGTGA----- ---------C TCTGGTAA-C TAGAGATCCC TCAGACCACT --CTAGATAG T-GTAAA--- AATCTCTAGA
G.PT.x.PT3037.FR846408         TAGTGTGTGC CCGTCTGTTG TGTGA----- ---------C TCTGGTAA-C TAGAGATCCC TCAGACCACT --CTAGACAG T-GTAAA--- AATCTCTAGC
G.PT.x.PT3306.FR846409         TAGTGTGTGC CCGTCTGTTG TGTGA----- ---------C TCTGGTAA-C TAGAGATCCC TCAGACCACT --CTAAACAG T-GTAAA--- AATCTCTAGC
G.PT.x.PT988.FR846410          TAGTGTGTGC CCGTCTGTTG TGTGA----- ---------C TCTGGTAA-C TAGAGATCCC TCAGACCACC --CTAGATAG T-GTAAA--- AATCTCTAGC
G.SE.93.SE6165_G6165.AF061642  ---------- ---------- ---------- ---------- ---------- ---------- ---------- ---------- --GGCTTA-- AATCTCTAGC
H.BE.93.VI991.AF190127         ---------- ---------- ---------- ---------- ---------- ---------- ---------- ---------- --------A- AATCTCTAGC
H.BE.93.VI997.AF190128         ---------- ---------- ---------- ---------- ---------- ---------- ---------- ---------- ---------- ----------
H.CF.90.056.AF005496           ---------- ---------- ---------- ---------- ---------- ---------- ---------- ---------- ---------- ----------
H.GB.00.00GBAC4001.FJ711703    TAGTGTGTGC CCGTCTGTTG TGTGA----- ---------C TCTGGTAW-C TAGAGATCCC TCAGACCATT --YTAGTCAG T-GTGGAA-- AATCTCTAGC
J.CM.04.04CMU11421.GU237072    TAGTGTGTGC CCGTCTGTTG TGTGA----- ---------C TCTGGTAA-C TAGAGATCCC TCAGACC-TT --TTAGTCAG T-GTGGAA-- AATCTCTAGC
J.SE.93.SE9280_7887.AF082394   ---------- ---------- ---------- ---------- ---------- ---------- ---------- ---------- ---------- ----------
J.SE.94.SE9173_7022.AF082395   ---------- ---------- ---------- ---------- ---------- ---------- ---------- ---------- ---------- ----------
01_AE.CF.90.90CF11697.AF197340 TGGTGTGTGC CCGTCTGTGA TAGGA----- ---------C TCTGGTAA-C TAGAGATCCC TCAGACCACT --CTAGACTG A-GTAAA--- AATCTCTAGC
01_AE.CF.90.90CF4071.AF197341  TGGTGTGTGC CCGTCTGTGA TAGGA----- ---------C TCTGGTAA-C TAGAGATCCC TCAGACCACT --CAAGACTG A-GTAAA--- AATCTCTAGC
01_AE.CF.90.90CR402_CAR_E_4002 TGGTGTGTGC CCGTCTGTGT TAGGA----- ---------C TCTGGTAA-C TAGAGATCCC TCAGACCACT --CTAGACTG A-GTAAA--- AATCTCTAGC
01_AE.CN.05.FJ051.DQ859178     CGGTGTGTGC CCATCTGTGT TAGGA----- ---------C TCTGGTAA-C TAGAGATCCC TCAGACCACT --CTAGACTG A-GTAAA--- AATCTCTAGC
01_AE.CN.05.FJ053.DQ859179     TGGTGTGTGC CCGTCTGTGT TAGGA----- ---------C TCTGGTAA-C TAGAGATCCC TCAGACCACT --CTAGATTG A-GTAAA--- AATCTCTAGC
01_AE.CN.05.Fj052.EF036528     TGGTGTGTGC CCGTCTGTGT TTGGA----- ---------C TCTGGTAA-C TAGAGATCCC TCAGACCACT --CTAGACTA A-GTAAA--- AATCTCTAGC
01_AE.CN.05.Fj055.EF036527     TGGTGTGTGC CCATCTGTGT TAGGA----- ---------C TCTGGTAA-C TAGAGATCCC TCAGACCACT --CTAGACTG A-GTAAA--- AATCTCTAGC
01_AE.CN.05.Fj056.EF036529     TGGTGTGTGC CCATCTGTGT TAGGA----- ---------C TCTGGTAA-C TAGAGATCCC TCAGACCACT --CCAGACTG A-GTAAA--- AATCTCTAGC
01_AE.CN.05.Fj057.EF036530     TGGTGTGTGC CCATCTGTGT TAGGA----- ---------C TCTGGTAA-C TAGAGATCCC TCAGACCACT --CTAGACTG A-GTAAA--- AATCTCTAGC
01_AE.CN.05.Fj065.EF036534     TGGTGTGTGC CCGTCTGTGT TAGGA----- ---------C TCTGGTAA-C TAGAGATCCC TCAGACCACT --CTAGACTG A-GTAAA--- AATCTCTAGC
01_AE.CN.05.Fj066.EF036535     TGGTGTGTGC CCATCTGTGT TAGGA----- ---------C TCTGGTAA-C TAGAGATCCC TCAGACCACT --CTAGACTG A-GTAAA--- AATCTCTAGC
01_AE.CN.06.FJ054.DQ859180     TGGTGTGTGC CCGTCTGTGT TAGGA----- ---------C TCTGGTAA-C TAGAGATCCC TCAGACACTG --CA-GMCTG A-GTAAA--- AATCTCTAGC
01_AE.CN.06.Fj062.EF036531     TGGTGTGTGC CCATCTGTGT TAGGA----- ---------C TCTGGTAA-C TAGAGATCCC TCAGACCACT --CTAGACTG A-GTAAA--- AATCTCTAGC
01_AE.CN.06.Fj063.EF036532     TGGTGTGTGC CCGTCTGTGT TAGGA----- ---------C TCTGGTAA-C TAGAGATCCC TCAGACCACT --CTAGACTA A-GTAAA--- AATCTCTAGC
```

01_AE.CN.06.Fj064.EF036533	TAGTGTGTGC	CCGTCTGTGT	TAGGA-----	---------C	TCTAGTAA-C	TAGAGATCCC	TCAGACCACT	--CTAGACTA	A-GTAAA---	AATCTCTAGC
01_AE.HK.04.HK001.DQ234790	----------	----------	----------	----------	----------	----------	----------	----------	----------	----------
01_AE.JP.x.DR0492.AB253423	TGGTGTGTGC	CCGTCTGTGT	TAGGA-----	---------C	TCTGGTAA-C	TAGAGATCCC	TCAGACCACT	--CTAGACTG	A-GTAAA---	AATCTCTAGC
01_AE.JP.x.DR2594.AB253660	TGGTGTGTGC	CCATTTGTGT	CAGGA-----	---------C	TCTGGTAA-C	TAGAGATCCC	TCAGACCACT	--CTAGACTG	A-GTAAA---	AATCTCTAGC
01_AE.JP.x.DR6824.AB253426	TGGTGTGTGC	CCATCTGTGT	TAGGA-----	---------C	TCTGGTAA-C	TAGAGATCCC	TCAGACCACT	--CTAGACTG	A-GTAAA---	AATCTCTAGC
01_AE.JP.x.JRC77AE.AB565504	TGGTGTGTGC	CCGTCTGTGT	TAGGA-----	---------C	TCTGGTAA-C	TAGAGATCCC	TCAGACCACT	--CTAGACTG	A-GTAAA---	ATTCTCTAGC
01_AE.TH.04.AA027a_wg4.JX44702	----------	----------	----------	----------	----------	----------	----------	----------	----------	----------
01_AE.TH.04.AA075a_WG7.JX44754	-------TGC	CCGTCTGTGT	TAGGA-----	---------C	TCTGGTAA-C	TAGAGATCCC	TCAGACCACT	--CTAGACTG	A-GTAA----	AATCTCTAGC
01_AE.TH.04.BKM.DQ314732	TGGTGTGTGC	CCGTCTGTGT	TTGGA-----	---------C	TCTGGTAA-C	TAGAGATCCC	TCAGACCACT	--CTAGACTG	A-GTAAA---	AATCTCTAGC
01_AE.TH.05.AA004a_wg4a.JX4467	----------	----------	----------	----------	--TGGTAA-C	TAGAGATCCC	TCAGACCACT	--CTAGACTG	A-GAAA----	AATCTCTAGC
01_AE.TH.05.AA033a_wg6a.JX4470	----------	----------	----------	----------	--TGGTAA-C	TAGAGATCCC	TCAGACCACT	--CTAGACTG	A-GTAAA---	AATCTCTAGC
01_AE.TH.05.AA049a_WG13.JX4472	----------	----------	----------	----------	----------	----------	----------	----------	----------	----------
01_AE.TH.05.AA064a_WG2.JX44741	----------	----------	----------	----------	----------	----------	----------	----------	----------	----------
01_AE.TH.05.AA079a_WG4.JX44759	----------	----------	----------	----------	----------	----------	----------	----------	----------	----------
01_AE.TH.05.AA101a_WG1.JX44793	----------	----------	----------	----------	----------	----------	----------	----------	----------	----------
01_AE.TH.05.AA107a_wg4.JX44802	----------	----------	----------	----------	----------	----------	----------	----------	----------	----------
01_AE.TH.06.AA002a_WG1.JX44666	--------GC	CCGTCTGTGT	TAGGA-----	---------C	TCTGGTAA-C	TAGAGATCCC	TCAGACCACT	--CTAGACTG	A-GTAAA---	AATCTCTAGC
01_AE.TH.06.AA017a_wg1.JX44689	----------	----------	----------	----------	----------	----------	----------	----------	----------	----------
01_AE.TH.06.AA034a_wg2.JX44708	----------	----------	----------	----------	--TGGTAA-C	TAGAGATCCC	TCAGACCACT	--CTAGACTG	A-GTAAA---	AATCTCTAGC
01_AE.TH.06.AA038a_WG3.JX44713	----------	----------	----------	----------	----------	----------	----------	----------	----------	----------
01_AE.TH.06.AA055a_WG4.JX44731	----------	----------	----------	----------	----------	----------	----------	----------	----------	----------
01_AE.TH.06.AA056a_WG5.JX44731	----------	----------	----------	----------	----------	----------	----------	-----GACTG	A-GTAAA---	AATCTCTAGC
01_AE.TH.06.AA059a_WG5.JX44735	-------TGC	CCGTCTGTGT	TAGGA-----	---------C	TCTGGTAA-C	TAGAGATCCC	TCAGACCACT	--CTAGACTG	A-GTAAA---	AATCTCTAGC
01_AE.TH.06.AA063a_WG37.JX4474	----------	----------	----------	----------	----------	----------	----ACCACT	--CTAGACTG	A-GTAAA---	AATCTCTAGC
01_AE.TH.06.AA068a_14.JX447465	----------	----------	----------	----------	----------	----------	----------	----------	----------	----------
01_AE.TH.06.AA082a_WG9.JX44764	-------TGC	CCGTCTGTGT	TAGGA-----	---------C	TCTGGTAA-C	TAGAGATCCC	TCAGACCACT	--CTAGACTG	A-GTAAA---	AATCTCTAGC
01_AE.TH.06.AA085a_wg2.JX44768	----------	----------	----------	----------	--TGGTAA-C	TAGAGATCCC	TCAGACCACT	--CTAGACTG	A-GTAAA---	AATCTCTAGC
01_AE.TH.06.AA088a_wg14.JX4477	----------	----------	----------	----------	--TGGTAA-C	TAGAGATCCC	TCAGACCACT	--CTAGACTG	A-GTAAA---	AATCTCTAGC
01_AE.TH.06.AA099a_WG9.JX44789	----------	----------	----------	----------	--TGGTAA-C	TAGAGATCCC	TCAGACCACT	--CTAGACGG	A-GTAAA---	AATCTCTAGC
01_AE.TH.07.AA015a_WG4.JX44687	----------	----------	----------	----------	----------	----------	TCAGACCACT	--CTAGACTG	A-GTAAA---	AATCTCTAGC
01_AE.TH.07.AA019a_WG11.JX4469	----------	----------	----------	----------	--TGGTAA-C	TAGAGATCCC	TCAGACCACT	--CTAGACTG	A-GTAAA---	AATCTCTAGC
01_AE.TH.07.AA028a_wg3.JX44702	----------	----------	----------	----------	--TGGTAA-C	TAGAGATCCC	TCAGACCACT	--CTAGACTG	A-GTAAA---	AATCTCTAGC
01_AE.TH.07.AA050a_WG7.JX44728	----------	----------	----------	----------	----------	----------	----------	----------	----------	----------
01_AE.TH.08.AA007a_WG10.JX4467	----------	----------	----------	----------	----------	----------	----------	----------	----------	----------
01_AE.TH.08.AA037a_WG6.JX44712	---------C	CCGTCTGTGT	TAGGA-----	---------C	TCTGGTAA-C	TAGAGATCCC	TCAGACCACT	--CTAGACTG	A-GTAA----	AATCTCTAGC
01_AE.TH.08.AA060a_WG1.JX44735	----------	----------	----------	----------	----------	----------	----------	----------	----------	----------
01_AE.TH.08.AA067a_WG12.JX4474	----------	----------	----------	----------	--TGGTAA-C	TAGAGATCCC	TCAGACCACT	--CTAGACTG	A-GTAAA---	AATCTCTAGC
01_AE.TH.08.AA108a_WG6.JX44802	----------	----------	----------	----------	--TGGTAA-C	TAGAGATCCC	TCAGACCACT	--CTAGACTG	A-GTAAA---	AATCTCTAGC
01_AE.TH.09.AA090a_WG11.JX4477	----------	----------	----------	----------	--TGGTAA-C	TAGAGATCCC	TCAGACCACT	--CTAGACTG	A-GTAAA---	AATCTCTAGC
01_AE.TH.09.AA111a_WG11.JX4480	----------	----------	----------	----------	--TGGTAA-C	TAGAGATCCC	TCAGACCACT	--CTAGACTG	A-GTAAA---	AATCTCTAGC
01_AE.TH.90.CM240.U54771	TGGTGTGTGC	CCGTCTGTGT	TAGGA-----	---------C	TCTGGTAA-C	TAGAGATCCC	TCAGACCACT	--CTAGACTG	A-GTAAA---	AATCTCTACC
01_AE.TH.93.93TH253.U51189	TGGTGTGTGC	CCATCTGTGT	TAGGA-----	---------C	TCTGGTAA-C	TAGAGATCCC	TCAGATCACT	--CTAGACTG	A-GTAAA---	AATCTCTAGC
01_AE.TH.93.93TH9021.AF164485	TGGTGTGTGC	CCGTCTGTGT	TAGGA-----	---------C	TCTGGTAA-C	TAAAAATCCC	TCAGACCACT	--CTAGACTG	A-GTAAA---	AATCTCTAGC
01_AE.TH.95.95TNIH022.AB032740	TGGTGTGTGC	CCGTCTGTGT	TAAGA-----	---------C	TCTGGTAA-C	TAGAGATCCC	TCAGACCACT	--CTAGACTG	A-GTAAA---	AATCTCTAGC
01_AE.TH.95.95TNIH047.AB032741	TGGTGTGTGC	CCGTCTGTGT	TAGGA-----	---------C	TCTGGTAA-C	TAGAGATCCC	TCAGACCACT	--CTAGACTG	A-GTAAA---	AATCTCTAGC
01_AE.TH.x.NP03.AB485654	TGGTGTGTGC	CCGTCTGTGT	TTGGA-----	---------C	TCTGGTAA-C	TAGAGATCCC	TCAGACCACT	--CTAGACTG	A-GTAAA---	AATCTCTAGC
01_AE.VN.97.97VNAG204.FJ185247	----------	----------	----------	----------	----------	----------	----------	----------	----------	----------
01_AE.VN.97.97VNAG206.FJ185248	----------	----------	----------	----------	----------	----------	----------	----------	----------	----------
01_AE.VN.97.97VNAG207.FJ185249	----------	----------	----------	----------	----------	----------	----------	----------	----------	----------
01_AE.VN.97.97VNAG210.FJ185251	----------	----------	----------	----------	----------	----------	----------	----------	----------	----------
01_AE.VN.97.97VNAG212.FJ185252	----------	----------	----------	----------	----------	----------	----------	----------	----------	----------
01_AE.VN.97.97VNAG214.FJ185253	----------	----------	----------	----------	----------	----------	----------	----------	----------	----------
01_AE.VN.97.97VNAG218.FJ185255	----------	----------	----------	----------	----------	----------	----------	----------	----------	----------
01_AE.VN.97.97VNHCM314.FJ18524	----------	----------	----------	----------	----------	----------	----------	----------	----------	----------
01_AE.VN.97.97VNHCM319.FJ18524	----------	----------	----------	----------	----------	----------	----------	----------	----------	----------
01_AE.VN.97.97VNHCM343.FJ18524	----------	----------	----------	----------	----------	----------	----------	----------	----------	----------
01_AE.VN.97.97VNHCM345.FJ18524	----------	----------	----------	----------	----------	----------	----------	----------	----------	----------
01_AE.VN.98.98VNND15.FJ185235	----------	----------	----------	----------	----------	----------	----------	----------	----------	----------
02_AG.CM.08.DE00208CM001.JX140	----------	----------	----------	----------	----------	----------	----------	----------	----------	----------
02_AG.CM.08.DE00208CM004.JX140	----------	----------	----------	----------	----------	----------	----------	----------	----------	----------
02_AG.CM.99.pBD6_15.AY271690	TAGTGTGTGC	CCGTCTGTTG	TGTGA-----	---------C	TCTGGTAA-C	TAGAGATCCC	TCAGACCACT	--TTAGACTG	T-GTAAA---	AATCTCTAGC
02_AG.ES.06.P1261.EU786671	-------TGC	CCGTCTGTTG	TGTGA-----	---------C	TCTGGTAT-C	TAGAGATCCC	TCAGACCACT	--CTAGACTT	A-GTGAAA--	AATCTCTAGC
02_AG.ES.06.P1423.EU884501	------GTGC	CCGTCTGTTG	TGTGA-----	---------C	TCTGGTAA-C	TAGAGATCCC	TCAGACCACT	--CGAGACCG	T-GTAGAA--	AATCTCTAGC
02_AG.FR.91.DJ263.AF063223	----------	----------	----------	----------	----------	----------	----------	----------	----------	----------
02_AG.GH.03.03GH181AG.AB286855	TAGTGTGTGC	CCGTCTGTTG	TGTGA-----	---------C	TCTGGTAT-C	TAGAGATCCC	TCAGACCACT	--GTAGACTG	T-GTAAAA--	AATCTCTAGC
02_AG.GH.03.03GH182AG.AB286857	TAGTGTGTGC	CCGTCTGTTG	TGTGA-----	---------C	TCTGGTAA-C	TAGAGATCCC	TCAGACCACT	--TTACTCAG	T-GTAAA---	AATCTCTAGC
02_AG.GH.03.03GH189AG.AB286862	TAGTGTGTGC	CCGTCTGTTG	TGTGA-----	---------C	TCTGGTAA-C	TAGAGATCCC	TCAGACCACT	--TTCAGTTT	GTGTAAA---	AATCTCTAGC
02_AG.GH.03.03GH197AG.AB286863	TAGTGTGTGC	CCGTCTGTTG	TGTGA-----	---------C	TCTGGTAA-C	TAGAGATCCC	TCAGACTACT	--CTAGACGG	T-GTAA----	AATCTCTAGC
02_AG.GH.03.GHNJ185.AB231895	TAGTGTGTGC	CCGTCTGTTG	TGTGA-----	---------C	TCTGGTAA-C	TAGAGATCCC	TCAGACCACT	--CTAGACTG	T-GTAGAA--	AATCTCTAGC
02_AG.GH.03.GHNJ188.AB231896	TAGTGTGTGC	CCGTCTGTTG	TGTGA-----	---------C	TCTGGTAA-C	TAGAGATCCC	TCAGACTACT	--TTAGTCAG	T-GTAAA---	AATCTCTAGC
02_AG.GH.03.GHNJ196.AB231898	TAGTGTGTGC	CCGTCTGTTG	TGTGA-----	---------C	TCTGGTAA-C	TAGAGATCCC	TCAGACCAAT	--TTAGTCTT	G-GTAAA---	AATCTCTAGC
02_AG.GH.97.97GH_AG1.AB049811	TAGTGTGTGC	CCGTCTGTTG	TGTGA-----	---------C	TCTGGTAA-C	TAGAGATCCC	TCAGACTACT	--CTAGACTG	T-GTAAA---	AATCTCTAGC
02_AG.GH.x.I_2496.AB485633	TAGTGTGTGC	CCGTCTATTG	TGTGA-----	---------C	TCTGGTAT-C	TAGAGATCCC	TCAGACCACT	--CTAGACTG	T-GTAAGAA-	AATCTCTAGC
02_AG.GW.04.CC_0030.FJ694791	----------	----------	----------	----------	----------	----------	----------	----------	----------	----------
02_AG.GW.05.CC_0048.FJ694792	----------	----------	----------	----------	----------	----------	----------	----------	----------	----------
02_AG.KR.07.07MHI10.JQ316136	TAGTGTGTGC	CCGTCTGTTG	TGTGA-----	---------C	TCTGGTAA-C	TAGAGATCCC	TCAGACCATT	--TTAGCTTG	A-GTAGA---	AATCTCTAGC
02_AG.LR.x.POC44951.AB485636	TAGTGTGTGC	CCGTCTGTTG	TGTGA-----	---------C	TCTGGTAA-C	TAGAGATCCC	TCAGACCACT	--CTAGACTG	T-ATAAA---	AATCTCTAGC
02_AG.NG.x.IBNG.L39106	TAGTGTGTGC	CCGTCTGATT	GTGTGA----	---------C	TCTGGTAA-C	TAGAGATCCC	TCAGACCACT	--CTAGACTG	T-GTAAA---	AATCTCTAGC
02_AG.SE.94.SE7812.AF107770	----------	----------	----------	----------	----------	----------	----------	----------	----------	--TCTCTAGC
03_AB.BY.00.98BY10443.AF414006	TAGTGTGTGC	CCGTCTGTTG	TATGA-----	---------C	TCTGGTAA-C	TAGAGATCCC	TCAGACCACT	--CTAGACGG	T-GTAAAA--	AATCTCTAGC
03_AB.RU.98.RU98001_98RU001.AF	----------	----------	----------	----------	----------	----------	----------	----------	-------A--	AATCTCTAGC
04_cpx.CY.94.94CY032_3.AF04933	----------	----------	----------	----------	----------	----------	----------	----------	----------	----------
04_cpx.GR.00.DE00400GR002.JX14	----------	----------	----------	----------	----------	----------	----------	----------	----------	----------
04_cpx.GR.91.GR11_97PVCH.AF119	TAGTGTGTGC	CCGTCTGTTG	TGTGA-----	---------C	TCTGGTAA-C	TAGAGATCCC	TCAGACCACT	--CTAGACTG	T-ATAAAA--	AATCTCTAGT
04_cpx.GR.97.GR84_97PVMY.AF119	TAGTGTGTGC	CCGTCTGGTG	TTTGA-----	---------C	TCTGGTAA-C	TAGAGATCCC	TCAGACCACT	--CTAGACGG	T-ATAAA---	AATCTCTAGC
05_DF.BE.93.VI961.AF076998	----------	----------	----------	----------	----------	----------	----------	----------	----------	----------
05_DF.BE.x.VI1310.AF193253	----------	----------	----------	----------	----------	----------	----------	----------	----------	-ATCTCTAGC
05_DF.ES.99.X492.AY227107	----------	----------	----------	----------	----------	----------	----------	----------	----------	----------
06_cpx.AU.96.BFP90.AF064699	TAGTGTGTGC	CCGTCTGTTG	TGTGA-----	---------C	TCTGGTAA-C	TAGAGATCCC	TCAGACCACT	--CTAGACAG	T-GTAAA---	AATCTCTAGC
06_cpx.CD.x.BCF_Dioum.AB485660	TAGTGTGTGC	CCGTCTGTTG	TGTGA-----	---------C	TCTGGTAA-C	TAGAGATCCC	TCAGACCACT	--CTAGACAG	T-GAAA----	AATCTCTAGC
06_cpx.EE.01.EE0359.AY535659	TAGTGTGTGC	CCGTCTGTTG	TGTGA-----	---------C	TCTGGTAA-C	TAGAGATCCC	TCAGAACACT	--TTAGACAG	T-GTAAA---	AATCTCTAGC
06_cpx.GH.03.03GH173_06.AB2868	TAGTGTGTGC	CCGTCTGTTG	TGTGA-----	---------C	TCTGGTAA-C	TAGAGATCCC	TCAGACCACT	--CTAGAAGG	T-GTAAA---	AATCTCTAGC
06_cpx.ML.95.95ML127.AJ288982	TAGTGTGTGC	CCGTCTGTTG	TGTGA-----	---------C	TCTGGTAA-C	TAGAGATCCC	TCAGACCACG	--CTAGGCGG	T-GTAA----	AATCTCTAGC
06_cpx.ML.95.95ML84.AJ245481	TAGTGTGTGC	CCGTCTGTTG	TGTGA-----	---------C	TCTGGTAA-C	TAGAGATCCC	TCAGACCACT	--CTTGACAG	T-GTAAA---	AATCTCTAGC
06_cpx.SN.97.97SE1078.AJ288981	TAGTGTGTGC	CCGTCTGGTG	TTTGA-----	---------C	TCTGGTAA-C	TAGAGATCCC	TCAGACCACT	--CTAGAAGG	T-GTAAA---	AATCTCTAGC
07_BC.CN.05.XJDC6431_2.EF36837	----------	----------	----------	----------	----------	----------	----------	----------	----------	----------
07_BC.CN.05.XJDC6441.EF368370	----------	----------	----------	----------	----------	----------	----------	----------	----------	----------
07_BC.CN.06.Sichuan_2006_SC006	----------	----------	----------	----------	----------	----------	----------	----------	----------	----------
07_BC.CN.06.Sichuan_2006_SC008	----------	----------	----------	----------	----------	----------	----------	----------	----------	----------
07_BC.CN.06.Xinjiang_2006_709.	----------	----------	----------	----------	----------	----------	----------	----------	----------	----------
07_BC.CN.07.CNGZD.JQ423923	----------	----------	----------	----------	----------	----------	----------	----------	----------	----------
07_BC.CN.98.98CN009.AF286230	----------	----------	----------	----------	----------	----------	----------	----------	----------	----------
08_BC.CN.00.p00CH_HH090_08_BC3	CAGTGTGTGC	CCGTCTATTG	TGTGA-----	---------C	TCTGGTAA-C	TAGAGATCCC	TCAGACC-CT	--TGTGGTAG	T-GTGGAA--	AATCTCTAGC
08_BC.CN.00.p00CH_WS035_08_BC5	CAGTGTGTGC	CCGTCTATTG	TGTGA-----	---------C	TCTGGTAA-C	TAGAGATCCC	TCAGACC-CT	--TGTGGTAG	T-GTGGAA--	AATCTCTAGC
08_BC.CN.01.p01CH_DL001_08_BC0	CAGTGTGTGC	CCGTCTATTG	TGTGA-----	---------C	TCTGGTAA-C	TAGAGATCCC	TCAGACC-CT	--TGTGGTAG	T-GTGGAA--	AATCTCTAGC

```
08_BC.CN.06.nx2.HM067748         CAGTGTGTGC CCATCTGTTG TGTGA----- ---------C TCTGGTAA-C TAGAGATCCC TCAGACC-CT --TGTGGCAG T-GTGGAA-- AATCTCTAGC
08_BC.CN.98.98CN006.AF286229     ---------- ---------- ---------- ---------- ---------- ---------- ---------- ---------- ---------- ----------
09_cpx.CI.00.00IC_10092.AJ8665   TAGTGTGTGC CCGTCTGTTG TGTGA----- ---------C TCTGGTAA-C TAGAGATCCC TCAGACCACT --CTAGACGA A-GTAAA--- AATCTCTAGC
10_CD.TZ.96.96TZ_BF061.AF28954   ---------- ---------- ---------- ---------- ---------- ---------- ---------- ---------- ---------- CCTCTCTAGC
10_CD.TZ.96.96TZ_BF071.AF28954   ---------- ---------- ---------- ---------- ---------- ---------- ---------- ---------- ---------- CCTCTCTAGC
10_CD.TZ.96.96TZ_BF110.AF28955   ---------- ---------- ---------- ---------- ---------- ---------- ---------- ---------- ---------- CCTCTCTAGC
11_cpx.CM.95.95CM_1816.AF49262   ---------- ---------- ---------- ---------- ---------- ---------- ---------- ---------- -----TA--- AATCTCTAGC
11_cpx.CM.96.96CM_4496.AF49262   ---------- ---------- ---------- ---------- ---------- ---------- ---------- ---------- ---------- ---------C
11_cpx.CM.97.MP818.AJ291718      AAGGGGGCCC CCGCCTGTGG GGGGA----- ---------C TTTGGAAC-C TAGAAATCCC TAAAACCTTT --TAGCCCGG --GGGGAA-- AATTTTTAGC
11_cpx.FR.99.MP1298.AJ291719     TAGTGTGTGC CCATCTGTTG TGTGA----- ---------C TCTGGTAA-C TAGAGATCCC TCAGACCTTT ---TAGTCCG T-GTGGAA-- AATCTCTAGC
11_cpx.FR.99.MP1307.AJ291720     TAGTGTGTGC CCGTCTGTTG TGTGA----- ---------C TCTGGTAA-C TAGAGATCCC TCAGACCTCT ---TATTACG T-GTGGAAAA TCTATATACA
11_cpx.GR.x.GR17.AF179368        ---------- ---------- ---------- ---------- ---------- ---------- ---------- ---------- ---------- ----------
12_BF.AR.97.A32879.AF408629      TAGTGTGTGC CCGTCTGTTG TGTGA----- ---------C TCTGGTAA-C TAGAGATCCC TCAGACCATT --TTAGTCAG G-GTGGAA-- AATCTCTAGC
12_BF.AR.97.A32989.AF408630      TAGTGTGTGC CCGTCTGTTG TGTGA----- ---------C TCTGGTAA-C TAGAGATCCC TCAGACCATT --TTAGTCAG T-GTGGAA-- AATCTCTAGC
12_BF.AR.99.ARMA159.AF385936     TAGTGTGTGC CCATTTGTTG TGTGA----- ---------C TCTGGTAA-C TAGAGATCCC TCAGACCATT --CTAGTCAG T-GTGGAA-- AATCTCTAGC
12_BF.UY.99.URTR23.AF385934      TAGTGTGTGC CCGTCTGTTG TGTGA----- ---------C TCTGGTAA-C TAGAGATCCC TCAGACTATT --TTAGTCAG T-GTGAAA-- AATCTCTAGC
12_BF.UY.99.URTR35.AF385935      TAGTGTGTGC CCGTCTGTTG TGTGA----- ---------C TCTGGTAA-C TAGAGATCCC TCAGACCATT --TTAGTCAG T-GTGGAA-- AATCTCTAGC
13_cpx.CM.02.02CM_A1394.DQ8453   TGGTGTGTGC CCATCTGTTG TGTGA----- ---------C CCTGGTAT-C TAGAGATCCC TCAGACATTT A-GTCCGTGC G-GTACGGAA AATCTCTAGC
13_cpx.CM.04.04CM_173_9.DQ8453   TAGTGTGTGC CCGTCTGTTG TGTGA----- ---------C TCTGGTAT-C TAGAGATCCC TCAGACCTT- --TTAGTCCG T-GTGGAA-- AATCTCTAGC
13_cpx.CM.04.04CM_632_28.DQ845   TAGTGTGTGC CCGTCTGTTG TGTGA----- ---------C TCTGGTAT-C TAGAGATCCC TCAGACTTT- --ATAGTCAG T-GTGGAA-- AATCTCTAGC
13_cpx.CM.96.96CM_1849.AF46097   ---------- ---------- ---------- ---------- ---------- ---------- ---------- ---------- --------A- AATCTCTAGC
13_cpx.CM.96.96CM_4164.AF46097   ---------- ---------- ---------- ---------- ---------- ---------- ---------- ---------- --------A- AATCTCTAGC
14_BG.ES.00.X605.AF450096        ---------- -------TTG TGTGA----- ---------C TCTGGTAA-C TAGAGATCCC TCAGACCACT --CTAAATAG T-GTAAA--- AATCTCTAGC
14_BG.ES.00.X623.AF450097        ---------- -------TTG TGTGA----- ---------C TCTGGTAA-C TAGAGATCCC TCAGACCACT --CTAGATAG T-GTA---A- AATCTCTAGC
14_BG.ES.05.X1870.FJ670522       TAA-GTGTGC CCGTCTGTTG TGTGA----- ---------C TCTGGTAA-C TAGAGATCCC TCAGACCACT --CTAGTTG- --TTGTAAA- AATCTCTAGC
14_BG.ES.05.X772_8.FJ670528      -TAAGGGTGC CCGTCTGTTG TGTGA----- ---------C TCTGGTAA-C TAGAGATCCC TCAGACCACT --CTAGATA- --GTGTAAA- AATCTCTAGC
15_01B.TH.99.99TH_MU2079.AF516   ---------- ---------- ---------- ---------- ---------- ---------- ---------- ----TAATTC G-CCCTTA-- AATCTCTAGC
15_01B.TH.99.99TH_R2399.AF5305   ---------- ---------- ---------- ---------- ---------- ---------- ---------- ---------- ---------- ----------
16_A2D.KR.97.97KR004.AF286239    ---------- ---------- ---------- ---------- ---------- ---------- ---------- ---------- ---------- ----------
18_cpx.CU.99.CU14.AY586541       ---------- ---------- ---------- ---------- ---------- ---------- ---------- ---------- ---------- ----------
18_cpx.CU.99.CU68.AY894993       ---------- ---------- ---------- ---------- ---------- ---------- ---------- ---------- ---------- ----------
18_cpx.CU.99.CU76.AY586540       ---------- ---------- ---------- ---------- ---------- ---------- ---------- ---------- ---------- ----------
19_cpx.CU.99.CU29.AY588971       TAGTGTGTGC CCGTCTGTTG TGTGA----- ---------C TCTGGTAA-C TAGAGATCCC TCAGACCACT --CTAAAAGG A-GTAA---- AATCTCTAGC
20_BG.CU.03.CB134.DQ020274       ---------- -------TTG TGTGA----- ---------C TCTGGTAA-C TAGAGATCCC TCAGACCACT --CTAGACAG T-GTAAA--- AATCTCTAGC
20_BG.CU.99.Cu103.AY586545       TAGTGTGTGC CCGTCTGTTG TGTGA----- ---------C TCTGGTAA-C TAGAGATCCC TCAGACCACT --CTAGACAG T-GTAAA--- AATCTCTAGC
20_BG.ES.99.R77.AY586544         TAGTGTGTGC CCGTCTGTTG TGTGA----- ---------C TCTGGTAA-C TAGAGATCCC TCAGACCACT --CTAGACAG T-GTAAA--- AATCTCTAGC
22_01A1.CM.02.02CAMLT72.EU7439   ---------- ---------- ---------- ---------- ---------- ---------- ---------- ---------- ---------- ----------
22_01A1.CM.06.LPH27MF.JN864049   ---------- ---------- ---------- ---------- ---------- ---------- ---------- ---------- ---------- ----------
22_01A1.CM.10.LB054.JN864059     ---------- ---------- ---------- ---------- ---------- ---------- ---------- ---------- ---------- ----------
23_BG.CU.03.CB118.AY900571       ---------- -------TTG TGTGA----- ---------C TCTGGTAA-C TAGAGATCCC TCAGACCACT --CTAGACAG T-GTAAA--- AATCTCTAGC
23_BG.CU.03.CB347.AY900572       ---------- -------TTG TGTGA----- ---------C TCTGGTAA-C TAGAGATCCC TCAGACCACT --CTAGACAG T-GTAAA--- AATCTCTAGC
24_BG.CU.03.CB378.AY900574       ---------- -------TTG TGTGA----- ---------C TCTGGTAA-C TAGAGATCCC TCAGACCACT --CTAGACRG T-GTAAA--- AATCTCTAGC
24_BG.CU.03.CB471.AY900575       ---------- -------TTG TGTGA----- ---------C TCTGGTAA-C TAGAGATCCC TCAGACAACT --CTAGATAG T-GTAAA--- AATCTCTAGC
24_BG.ES.08.X2456_2.FJ670526     ------GTGC CCGTCTGTTG TGTGA----- ---------C TCTGGTAA-C TAGAGATCCC TCAGACCACT --CTAGACAG T-GTAAA--- AATCTCTAGC
25_cpx.CM.06.06CM_BA_040.EU693   TAGTGTGTGC CCGTCTGTTG TGTGA----- ---------C TCTGGTAA-C TAGAGATCCC TCAGACCACT --GTAGATAT T-GTAA---- AATCTCTAGC
25_cpx.SA.03.J11233.EU697906     TAGTGTGTGC CCGTCTGTTG TGTGA----- ---------C TCTGGTAA-C TAGAGATCCC TCAGACCACT --GTAGCGAG T-GTAA---- AATCTCTAGC
25_cpx.SA.03.J11451.EU697908     TAGTGTGTGC CCGTCTGTTG TGTGA----- ---------C TCTGGTAA-C TAGAGATCCC TCAGACCACT --GTAG-GAG T-GTAA---- AATCTCTAGC
26_AU.CD.02.02CD_KS069.FM87778   TGGTGTGTGC CCGTCTGTTG ATTGA----- ---------C TCTGGTAA-C TAGAGATCCC TCAGACTACT --GAAGGTAG T-ATAAAA-- AATCTTTACC
26_AU.CD.02.02CD_LBTB084.FM877   TAGTGTGTGC CCGTCTGTTG TTTGA----- ---------C TCTGGTAA-C TAGAGATCCC TCAGATCACT -TTTAGGCAG A-GTAAA--- AATCTCTAGC
26_AU.CD.02.02CD_MBTB047.FM877   TGGTGTGTGC CCGTCTGTTG TGTGA----- ---------C TCTGGTAA-C TAGAGATCCC TCAGACCACT --CTAGGTAG T-GTAAA--- AATCTCTAGC
26_AU.CD.97.97CD_KTB119.FM8777   TAGTGTGTGC CCGTCTGTTG TGTGA----- ---------C TCTGGTAA-C TAGAGATCCC TCAGACCACT --CTAGGTAG T-GTAAA--- AATCTCTAGC
27_cpx.FR.04.04CD_FR_KZS.AM851   TAGTGTGTGC CCGTCTGTTG TGTGA----- ---------C TCTGGTAA-C TAGAGATCCC TCAGACCATT --TTAGTCAG T-GTGGAA-- AATCTCTAGC
28_BF.BR.05.0679SV.JF804812      ---------- ---------- ---------- ---------- ---------- ---------- ---------- ---------- ---------- ----------
28_BF.BR.99.BREPM12313.DQ08587   ---------- ---------- ---------- ---------- ---------- ---------T TCTGACGCTT --TTAGTCAG T-GTGGAA-- AATCTCTAGC
28_BF.BR.99.BREPM12609.DQ08587   ---------- ---------- ---------- ---------- ---------- ---------- ---------- ---------- ---------- ------TCGC
28_BF.BR.99.BREPM12817.DQ08587   ---------- ---------- ---------- ---------- --------AC TAGAGATCCC TCAGACACTT --TTAGTCAG T-GTGGAA-- AATCTCTAGC
29_BF.BR.01.BREPM16704.DQ08587   ---------- ---------- ---------- ---------- ---------- ---------- ---------- ---------- ---------- ------TAGC
29_BF.BR.05.0264RI.JF804807      ---------- ---------- ---------- ---------- ---------- ---------- ---------- ---------- ---------- ----------
29_BF.BR.99.BREPM11948.DQ08587   ---------- ---------- ---------- ---------- ---------- ---------- ------TCTT --TTAGTTCG T-GTGGAA-- AATCTCTAGC
31_BC.BR.02.110PA.EF091932       TAGTGTGTGC CCGTCTGTTG TGTGA----- ---------C TCTGGTAA-C TAGAGATCCC TCAGACACCT --TTWGTCAG T-GTGGAA-- AATCTCTAGC
31_BC.BR.04.04BR142.AY727527     TAGTGTGTGC CCGTCTGTTG TGTGA----- ---------C TCTGGTAG-C TAGAGATCCC TCAGATACCT --TTTGTCAG T-GTGGAA-- AATCTCTAGC
32_06A1.EE.01.EE0369.AY535660    TAGTGTGTGC CCGTCTGTTG TGTGA----- ---------C TCTGGTAA-C TAGAGATCCC TCAGAACACT --TTAGACAG T-GTAAA--- AATCTCTAGC
32_06A1.EE.02.EST2002_1169.DQ1   ---------- --GACTGTTG TGTGA----- ---------C TCTGGTAA-C TAGAGATCCC TCAGAAACAC T-TTAGACAG T-GTAAA--- AATCTCTAGC
33_01B.ID.07.JKT189_C.AB547463   ---------- ---------- ---------- ---------- ---------- ---------- ---------- ---------- ---------- ----------
33_01B.MY.05.05MYKL007_1.DQ366   ---------- ---------- ---------- ---------- ---------- ---------- ---------- ---------- ---------- ----------
33_01B.MY.05.05MYKL015_2.DQ366   ---------- ---------- ---------- ---------- ---------- ---------- ---------- ---------- ---------- ----------
33_01B.MY.05.05MYKL031_1.DQ366   ---------- ---------- ---------- ---------- ---------- ---------- ---------- ---------- ---------- ----------
33_01B.MY.05.05MYKL045_1.DQ366   ---------- ---------- ---------- ---------- ---------- ---------- ---------- ---------- ---------- ----------
38_BF1.UY.03.UY03_3389.FJ21378   ---------- --------TG TGTGA----- ---------C TCTGGTAA-C TAGAGATCCC TCAGACCACT --TTAGTCAG T-GTGGAA-- AATCTCTAGC
38_BF1.UY.04.UY04_3987.FJ21378   ---------- --------TG TGTGA----- ---------C TCTGGTAA-C TAGAGATCCC TCAGACCATT --TTAGTCAG T-GTGGAA-- AATCTCTAGC
38_BF1.UY.04.UY04_4022.FJ21378   ---------- --------TG TGTGA----- ---------C TCTGGTAC-T A-GAGATCCC TCAGACACTT --TTAGTCCG T-GTGGAA-- AATCTCTAGC
38_BF1.UY.05.UY05_4752.FJ21378   ---------- --------TG TGTGA----- ---------C TCTGGTAA-C TAGAGATCCC TCAGACCCTT --TTAGTCAG T-GTGGAA-- AATCTCTAGC
39_BF.BR.03.03BRRJ103.EU735534   TAGTGTGTGC CCGTCTGTTG TGTGA----- ---------C TCTGGTAA-C TAGAGATCCC TCAGACCTTT ---TAGTCAG T-GTGGAA-- AATCTCTAGC
39_BF.BR.03.03BRRJ327.EU735536   ---------- ---------- ---------- ---------- ---------- ---------- --------TT --TTAGTCAG T-GTGGAA-- AATCTCTAGC
39_BF.BR.04.04BRRJ179.EU735535   TAGTGTGTGC CCGTCTGTTG TGTGA----- ---------C TCTGGTAT-C TAGAGATCCC TCAGACCATT --TTAGTCAG T-GTGGAA-- AATCTCTAGC
40_BF.BR.04.04BRRJ115.EU735538   TAGTGTGTGC CCGTCTGTTG TGTGA----- ---------C TCTGGTAA-C TAGAGATCCC TCAGACCATT --TTAGTCAG T-GTGGAA-- AATCTCTAGC
40_BF.BR.04.04BRSQ46.EU735540    TAGTGTGTGC CCGTCTGTTG TGTGA----- ---------C TCTGGTAA-C TAGAGATCCC TCAGACCCTT --TTAGTCAG T-GTGGAA-- AATCTCTAGC
40_BF.BR.05.05BRRJ055.EU735537   TAGTGTGTGC CCGTCTGTTG TGTGA----- ---------C TCTGGTAA-C TAGAGATCCC TCAGACCATT --TTAGTCAG T-GTGGAA-- AATCTCTAGC
40_BF.BR.05.05BRRJ200.EU735539   TAGTGTGTGC CCGTCTGTTG TGTGA----- ---------C TCTGGTAA-C TAGAGATCCC TCAGACCCAT --TTAGTCAG T-GTGGAA-- AATCTCTAGC
42_BF.LU.03.luBF_01_03.EU17015   TAGTGTGTGC CCGTCTGTTG TGTGA----- ---------C TCTGGTAA-C TAGAGATCCC TCAGACCATT --TTAGTCCG T-GTGGAA-- AATCTCTAGC
42_BF.LU.03.luBF_02_03.EU17015   TAGTGTGTGC CCGTCTGTTG TGTGA----- ---------C TCTGGTAA-C TAGAGATCCC TCAGACCATT --TTAGTCCG T-GTGGAA-- AATCTCTAGC
42_BF.LU.03.luBF_04_03.EU17014   TAGTGTGTGC CCGTCTGTTG TGTGA----- ---------C TCTGGTAA-C TAGAGATCCC TCAGACCATT --TTAGTCCG T-GTGGAA-- AATCTCTAGC
42_BF.LU.03.luBF_05_03.EU17015   TAGTGTGTGC CCGTCTGTTG TGTGA----- ---------C TCTGGTAA-C TAGAGATCCC TCAGACCAYT --TTAGTCCG T-GTGGAA-- AATCTCTAGC
42_BF.LU.03.luBF_06_03.EU17014   TAGTGTGTGC CCGTCTGTTG TGTGA----- ---------C TCTGGTAA-C TAGAGATCCC TCAGACCCTT --TTAGTCCG T-GTGGAA-- AATCTCTAGC
42_BF.LU.03.luBF_07_03.EU17014   TAGTGTGTGC CCGTCTGTTG TGTGA----- ---------C TCTGGTAA-C TAGAGATCCC TCAGACCATT --TTAGTCCG T-GTGGAA-- AATCTCTAGC
42_BF.LU.03.luBF_08_03.EU17014   TAGTGTGTGC CCGTCTGTTG TGTGA----- ---------C TCTGGTAA-C TAGAGATCCC TCAGACCATT --TTAGTCCG A-GTGGAA-- AATCTCTAGC
42_BF.LU.03.luBF_09_03.EU17015   TAGTGTGTGC CCGTCTGTTG TGTGA----- ---------C TCTGGTAA-C TAGAG-WCCC TCAGACCWTT --TTAGTCCG K-GKGGAA-- AATCTCTAGC
42_BF.LU.04.luBF_11_04.EU17014   TAGTGTGTGC CCGTCTGTTG TGTGA----- ---------C TCTGGTAA-C TAGAGATCCC TCAGACCATT --TTAGTCCG T-GTGGAA-- AATCTCTAGC
42_BF.LU.04.luBF_12_04.EU17014   TGGTGTGTGC CCGTCTGTTG TGTGA----- ---------C TCTGGTAA-C TAGAGATCCC TCAGACCATT --TTAGTCCG T-GTGGAA-- AATCTCTAGC
42_BF.LU.05.luBF_13_05.EU17013   TAGTGTGTGC CCGTCTGTTG TGTGA----- ---------C TCTGGTAA-C TAGAGATCCC TCAGACCAAT --TTAGTCCG T-GTGGAA-- AATCTCTAGC
42_BF.LU.05.luBF_14_05.EU17014   TAGTGTGTGC CCGTCTGTTG TGTGA----- ---------C TCTGGTAA-C TAGAGATCCC TCAGACCAAT --TTAGTCCG T-GTGGAA-- AATCTCTAGC
42_BF.LU.05.luBF_16_05.EU17015   TAGTGTGTGC CCGTCTGTTG TGTGA----- ---------C TCTGGTAA-C TAGAGATCCC TCAGACCACT --TTAGTCCG T-GTGGAA-- AATCTCTAGC
42_BF.LU.05.luBF_17_05.EU17014   TAGTGTGTGC CCGTCTGTTG TGTGA----- ---------C TCTGGTAA-C TAGAGATCCC TCAGACCAAT --TTAGTCCG K-GTGGAA-- AATCTCTAGC
42_BF.LU.06.luBF_18_06.EU17013   TAGTGTGTGC CCGTCTGTTG TGTGA----- ---------C TCTGGTAA-C TAGAGATCCC TCAGACCACT --TTAGTCCG T-GTGGAA-- AATCTCTAGC
42_BF.LU.06.luBF_19_06.EU17013   TAGTGTGTGC CCGTCTGTTG TGTGA----- ---------C TCTGGTAA-C TAGAGATCCC TCAGACCATT --TTAGTCCG T-GTGGAA-- AATCTCTAGC
43_02G.SA.03.J11223.EU697904     TAGTGTGTGC CCATCTGTTG TGTGA----- ---------C TCTGGTAA-C TAGAGATCCC TCAGACCACT --CTAGACGG T-GTAAA--- AATCTCTAGC
43_02G.SA.03.J11232.EU697905     TAGTGTGTGC CCGTCTGTTG TGTGA----- ---------C TCTGGTAA-C TAGAGATCCC TCAGACCACT --CTAGACTG T-GTGAA--- AATCTCTAGC
43_02G.SA.03.J11243.EU697907     TAGTGTGTGC CCGTCTGTTG TGTGA----- ---------C TCTGGTAA-C TAGAGATCCC TCAGACCACT --CCAGACTG T-GTGAA--- AATCTCTGGC
43_02G.SA.03.J11456.EU697909     TAGTGTGTGC CCGTCTGTTG TGTGA----- ---------C TCTGGTAA-C TAGAGATCCC TCAGACCACT --CTAGACTG T-GTAAA--- AATCTCTAGC
44_BF.CL.00.CH80.FJ358521        ------GTGC CCGTCTGTTG TGTGA----- ---------C TCTGGTAG-C TAGAGATCCC TCAGACCATT --TTAGTCAG T-GTGGAA-- AATCTCTAGC
44_BF.CL.01.CH12.AY536235        TAGTGTGKGC CCGTCTGTTG TTTGA----- ---------C TCTGGTAG-C TAGAGATCCC TCAGACCATT --TTAGTCAG T-GTRGAA-- AATCTCTAGC
45_cpx.CD.97.97CD_MBFE185.FN39   CAGTGTGTGC CCGTCTGTTG TGTGA----- ---------C TYTGGTAA-C TAGAGATCCC TCAGACCACT --CTAGACAG T-GTAAA--- AATCTCTAGC
45_cpx.CD.97.97CD_MBS30.FN3928   CAGTGTGTGC CCGTCTGTTG TGTGA----- ---------C TCTGGTAA-C TAGAGATCCC TCAGACCACT --CTAGACGG T-ATAAA--- AATCTCTAGC
45_cpx.CM.97.97CM_MP814.FN3928   CAGTGTGTGC CCGTYTGTTG TGTGA----- ---------C TTTGGTAA-C TAGAGATCCC TCAGACCACT --YTAGRCGG T-GTAAA--- AATCTYTAGC
45_cpx.FR.04.04FR_AUK.EU448295   TAGTGTGTGC CCGTCTGTTG TGTGA----- ---------C TCTGGTAA-C TAGAGATCCC TCAGACCACT --CTAGGCGG T-GTAAA--- AATCTCTAGC
```

```
45_cpx.GA.97.97GA_TB45.FN39287    CAGTGTGTGC CCATYTATTG A--------- ---------Y TCTGGTAA-C TAGAGATC-- ---------- ---------- --ATATAAA- AATCTCTAGC
46_BF.BR.01.01BR087.DQ358801      TAGTGTGTGC CCGTCTGTTG TGTGA----- ---------C TCTGGTAA-C TAGAGATCCC TCAGACCATT --TTAGTCAG T-GTGGAA-- AATCTCTAGC
46_BF.BR.01.01BR125.DQ358802      TAGTGTGTGC CCGTCTGTTG TATGA----- ---------C TCTGGTAA-C TAAAGATCCC TCAGAACATT --TTAGTCTG T-GTGGAAA- AATCTCTAGC
46_BF.BR.07.07BR_FPS625.HM0264    TAGTGTGTGC CCGTCTGTTG TGTGA----- ---------C TCTGGTAA-C TAGAGATCCC TCAGAACATT --TTAGTCCG A-GTGGAA-- AATCTCTAGC
46_BF.BR.07.07BR_FPS742.HM0264    ---------- ---------- ---------- ---------- ---------A CTGAGATCCC TCAGACCATT --TTAGTCAG T-GTGGAA-- AATCTCTAGC
46_BF.BR.07.07BR_FPS783.HM0264    TTGTGTGTGC CCGTCTGTTG TGTGA----- ---------C TCTGGTAA-C TAGAGATCCC TCAGAACATT --TTAGTCCG G-GTGGAA-- AATCTCTAGC
46_BF.BR.07.07BR_FPS810.HM0264    TAGTGTGTGC CCGTCTGTTG TGTGA----- ---------C TCTGGTAA-C TAGAGATCCC TCAGAACATT --TTAGTCTG G-GTAGAA-- AATCTCTAGC
46_BF.BR.07.07BR_FPS812.HM0264    TAGTGTGTGC CCGTCTGTTG TGTGA----- ---------C TCTGGTAA-C TAGAGATCCC TCAGACATTT ---TAGTCAG T-GTGGAA-- AATCTCTAGC
47_BF.ES.08.P1942.GQ372987        --AGGGTTGC CCGTCTGTTG TGTGA----- ---------C TCTGGTAA-C TAGAGATCCC TCAGACCATT ---TAGTCAG T-GTGGAAA- AATCTCTAGC
47_BF.ES.08.X2457_2.FJ670529      -AAGGGTTGC CCGTCTGTTG TGTGA----- ---------C TCTGGTAA-C TAGAGATCCC TCAGACCATT ---TAGTCAG T-GTGGAA-- AATCTCTAGC
49_cpx.GM.02.N18380.HQ385477      ---------- --ATCTGTGG TGTGA----- ---------C TCTGGTAA-C TAGAGATCCC TCAGACCTCT --CTAGACG- --GTGTAAA- AATCTCTAGC
49_cpx.GM.03.N26677.HQ385479      ---------- --ATCTGTGG TGTGA----- ---------C TCTGGTAG-C TAGAGATCCC TCAGACCACT --CTAGACG- --GTGTAAA- AATCTCTAGC
49_cpx.GM.97.N28353.HQ385478      --GTGTGTGC CCATCTGTAG TGTGTGA--- ---------C TCTGGTAA-C TAGAGATCCC TCAGACCACT --CTAGAAG- --GTGTAAA- AATCTCTAGC
52_01B.MY.03.03MYKL018_1.DQ366    ---------- ---------- ---------- ---------- ---------- ---------- ---------- ---------- ---------- ----------
53_01B.MY.04.04MYKL016_1.DQ366    ---------- ---------- ---------- ---------- ---------- ---------- ---------- ---------- ---------- ----------
53_01B.MY.10.10MYKJ067.JX39061    ---------- ---------- ---------- ---------- ---------- ---------- ---------- ---------- ---------- ----------
53_01B.MY.10.10MYKJ079.JX39061    ---------- ---------- ---------- ---------- ---------- ---------- ---------- ---------- ---------- ----------
53_01B.MY.11.11FIR164.JX390610    ---------- ---------- ---------- ---------- ---------- ---------- ---------- ---------- ---------- ----------
54_01B.MY.07.07MYKLD49.EU03191    ---------- ---------- ---------- ---------- ---------- ---------- ---------- -----GCAAC C-TCAATA-- AATCTCTAGC
54_01B.MY.08.08MYKL044.JX39097    ---------- ---------- ---------- ---------- ---------- ---------- ---------- ---------- ---------- ----------
54_01B.MY.09.09MYSB023.JX39097    ---------- ---------- ---------- ---------- ---------- ---------- ---------- ---------- -------A-- AATCTCTAGC
55_01B.CN.10.HNCS102056.JX5746    ---------- ---------- ---------- ---------- ---------- ---------- ---------- ---------- ---------- ----------
55_01B.CN.11.GDDG318.JX574662     ---------- ---------- ---------- ---------- ---------- ---------- ---------- ---------- ---------- ----------
01A1.MM.99.mCSW105.AB097872       TGGTGTGTGC CCGTCTGTTG TGTGA----- ---------C TCTGGTAA-C TAGAGATCCC TCAGACCACT --CTAGAAGG T-GTAAA--- AATCTCTAGC
01B.CN.08.08CYM047.JF340054       ---------- ---------- ---------- ---------- ---------- ---------- ---------- ---------- ---------- ----------
01B.JP.x.pHIV_1_Y271B01AE64.AB    TAGTGTGTGC CCGTCTGTTG TGTGA----- ---------C TCTGGTAA-C TAGAGATCCC TCAGACCACT T-TCAGTTTG T-GTAAA--- AATCTCTAGC
01B.MM.00.mIDU502.AB097865        ---------- ---------- ---------- ---------- ---------- ---------- ---------- ---------- ---------- -ATCTCTAGC
01B.MM.99.mCSW104.AB097867        ---------- ---------- ---------- ---------- ---------- ---------- ---------- ---------- --------A- AATCTCTAGC
01B.MY.04.04MYKL019_1.DQ366665    ---------- ---------- ---------- ---------- ---------- ---------- ---------- ---------- ---------- ----------
01B.MY.05.05MYKL043_1.DQ366666    ---------- ---------- ---------- ---------- ---------- ---------- ---------- ---------- ---------- ----------
01B.MY.06.06MMYKLD46.EF495062     TGGTGTGTGC CCGTCTGTGT TAGGA----- ---------C TCTGGTAA-C TAGAGATCCC TCAGACCACT --CTAGACTG A-TTAAA--- AATCTCTAGC
01B.MY.07.07MYKLD47.EU031913      ---------- ---------- ---------- ---------- ---------- ---------- ---------- ---------- ---------- ----------
01B.MY.07.07MYKLD48.EU031914      TGGTGTGTGC CCGTCTGTGT TAGGA----- ---------C TCTGGTAA-C TAGAGATCCC TCAGACCACT --CTAGACTG A-TTAAA--- AATCTCTAGC
01B.TH.05.05TH140456.JN631793     ---------- ---------- ---------- ---------- -----TAA-C TAGAGATCCC TCAGACCACT --CTAGACTG A-GTAAA--- AATCTCTAGC
01B.TH.05.AA095a_WG21.JX447830    ---------- -CGTCTGTGT TAGGA----- ---------C TCTGGTAA-C TAGAGTTCCC TCAGACCACT --CTAGACGA A-GTAAA--- AATCTCTAGC
01B.TH.06.AA020a_wg2.JX446927     ---------- ---------- ---------- ---------- ---------- ---------- ---------- ---------- ---------- ----------
01B.TH.06.AA025a_WG13.JX447000    ---------- ---------- ---------- ---------- --TGGTAA-C TAGAGATCCC TCAGACCACC --CTAGACTG A-GTAAA--- AATCTCTAGC
01B.TH.06.AA084a_WG10.JX447668    ---------- ---------- ---------- ---------- --TGGTAA-C TAGAGATCCC TCAGACCACT --CTAGACTG A-GTAAA--- AATCTCTAGC
01B.TH.91.CM237.AY167123          TAGTGTGTGC CCGTCTGTTG TGTGA----- ---------C TCTGGTAG-C TAGAGATCCC TCAGACCTTG --TTAGTCAG T-GTGGAA-- AATCTCTAGC
01BC.MM.00.mCSW503.AB097866       ---------- ---------- ---------- ---------- ---------- ---------- ---------- ---------- ---------- ----------
01BC.MM.99.mIDU107.AB097868       CAGTGTGTGC CCGTCTATTG TGTGA----- ---------C TCTGGTAA-C TAGAGATCCC TCAGACCATT ---TTGGTAG T-GTGGAA-- AATCTCTAGC
01F2G.CM.02.LT31.JN864056         ---------- ---------- ---------- ---------- ---------- ---------- ---------- ---------- ---------- ----------
0206.DZ.10.DEURF10DZ001.JX1406    ---------- ---------- ---------- ---------- ---------- ---------- ---------- ---------- ---------- ----------
0206.GH.03.03GH195AG_06.AB2868    TAGTGTGTGC CCGTCTGTTG TGTGA----- ---------C TCTGGTAA-C TAGAGATCCC TCAGACTACT --CTAGACGG T-GTAA---- AATCTCTAGC
0206.NE.00.NE36.AJ508597          TAGTGTGTGC CCGTCTGTTG TGTGA----- ---------C TCTGGTAA-C TAGAGATCCC TCAGACCACT --CTAGACCG C-GTAAA--- AATCTCTAGC
0206.NE.00.NE95.AJ508596          TAGTGTGTGC CCGTCTGTTG TGTGA----- ---------C TCTGGTAA-C TAGAGATCCC TCAGACCACT --GTTGATAG T-GTAAA--- AATCTCTAGC
0206.NE.97.NE03.AJ508595          TAGTGTGTGC CCGTCTGTTG TGTGA----- ---------C TCTGGTAA-C TAGAGATCCC TCAGACCACT --CTAGGCGG T-GTAAA--- AATCTCTAGC
0209.CI.01.01IC_17395.AJ866554    TAGTGTGTGC CCGTCTGTTG TGTGA----- ---------C TCTGGTAA-C TAGAGATCCC TCAGACCACT --CTAGACTG T-GTAAAA-- AATCTCTAGC
0209.CI.01.01IC_PCI118.AJ86655    TAGTGTGTGC CCGTCTGGTG TTTGA----- ---------C TCTGGTAA-C TAGAGATCCC TCAGACCACT --CTAGACTG T-GTAAA--- AATCTCTAGC
0209.CI.01.01IC_PCI127.AJ86655    TAGTGTGTGC CCGTCTGTTG TGTGA----- ---------C TCTGGTAA-C TAGAGATCCC TCAGACCACT --CTAGACGG T-GTAAA--- AATCTCTAGC
0209.CI.97.97IC_PCI3.AJ866555     TAGTGTGTGC CCGTCTGTTG TGTGA----- ---------C TCTGGTAA-C TAGAGATCCC TCAGACCACT --CTAGACGA G-GTAAA--- AATCTCTAGC
0222.CM.02.02CAMLT04.EU743964     ---------- ---------- ---------- ---------- ---------- ---------- ---------- ---------- ---------- ----------
0222.CM.08.BDSH129.JN864052       ---------- ---------- ---------- ---------- ---------- ---------- ---------- ---------- ---------- ----------
0222.CM.10.LB045.JN864053         ---------- ---------- ---------- ---------- ---------- ---------- ---------- ---------- ---------- ----------
02A1.ES.05.X230_10.FJ670515       TTAAGGGTGC CCGTCTGTTG TGTGA----- ---------C TCTGGTAA-C TAGAGATCCC TCAGACCATT --CTAGACGG T-GTAAA--- AATCTCTAGC
02A1.ES.07.ES_P1751.GQ372986      -AAGGGGTGC CCGTCTGTTG TGTGA----- ---------C TCTGGTAA-C TAGAGATCCC TCAGACCACT --CTAGACGG T-GTAAA--- AATCTCTAGC
02A1.GH.03.GHNJ176.AB231894       TAGTGTGTGC CCGTCTGTTG TGTGA----- ---------C TCTGGTAA-C TAGAGATCCC TCAGACCACT --CTAGTCTG C-GTAAA--- AATCTCTAGC
02A1.GH.97.97GH_AG2.AB052867      TAGTGTGTGC CCGTCTGTTG TGTAA----- ---------C TCTGGTAA-C TAGAGATCCC TCAGACCACT --CTAGGTTG T-GTAAA--- AATCTCTAGC
02A1.RU.10.10RU6637.JN230353      TAGTGTGTGC CCGTCTGTTG TGTGA----- ---------C TCTGGTAA-C TAGAGATCCC TCAGACCACT --CTAGGCAG T-GTAAA--- AATCTCTAGC
02B.ES.99.99SP_11339.DQ926899     TAGTGTGTGC CCGTCTGTTG TGTGA----- ---------C TCTGGTAT-C TAGAGATCCC TCAGACCACT --CGAGATTG A-GTAGAA-- AATCTCTAGC
02B.FR.02.URF4.JN882652           ---------- ---------- ---------- ---------- ---------- ---------- ---------- ---------- ---------- ----------
02B.FR.09.URF2.JN882654           ---------- ---------- ---------- ---------- ---------- ---------- ---------- ---------- ---------- ----------
02BD.FR.08.URF1.JN882653          -----TGTGC CCGTCTGTTG TGCGA----- ---------C TCTGGTAA-C TAGAGATCCC TCAGACCACT --CTAGACTG T-GTAAA--- AATCTCTAGC
02BG.FR.10.URF5.JN882655          ---------- ---------- ---------- ---------- ---------- ---------- ---------- ---------- ---------- ----------
02C.BE.93.VI1035.AJ276595         ---------- ---------- ---------- ---------- ---------- ---------- ---------- ---------- ---------- ----------
02D.GH.03.GHNJ193.AB231897        TAGTGTGTGC CCGTCTGTTG TGTGA----- ---------C TCTGGTAA-C TAGAGATCCC TCAGACCACT --CTAGTCTG C-GTAAA--- AATCTCTAGC
02D.GH.03.p03GH194AG09.AB48004    TAGTGTGTGC CCGTCTGTTG TGTGA----- ---------C TCTGGTAA-C TAGAGATCCC TCAGACCTTT --GTAGTCAC T-GTAGAA-- AATCTCTAGC
02F2G.CM.02.LT66.JN864057         ---------- ---------- ---------- ---------- ---------- ---------- ---------- ---------- ---------- ----------
02GK.CI.01.01IC_PCI123.AJ86655    TAGTGTGTGC CCGTCTGTTG TGTGA----- ---------C TCTGGTTA-C TAGAGATCCC TCAGACCACT --CTAGACTG T-GTAAA--- AATCTCTAGC
06A1.BJ.x.B76.AJ293865            ---------- ---------- ---------- ---------- ---------- ---------- ---------- ---------- ---------- ----------
1819.CU.99.CU64.AY894995          TAGTGTGTGC CCGTCTGTTG TGTGA----- ---------C TCTGGTAA-C TAGAGATCCC TCAGACCACT --CTAGACGG --GTGTAAA- AATCTCTAGC
26C.CD.02.02CD_LBTB032.FM87777    TAGTGTGTGC CCGTCTGTTG TTTGA----- ---------C TCTGGTAA-C TAGAGATCCC TCAGACCATT --CTAGGCAG A-GTAAA--- AATCTCTAGC
26C.CD.97.97CD_KFE267.FM877778    TAGTGTGTGC CCGTCTGTTA TGTGA----- ---------C TCTGGTAA-C TAGAGATCCC TTAGACCACT --CTAGGCAG T-GTGAAA-- AATCTCTAGC
26C.CD.97.MBFE250.FM877783        TAGTGTGTGC CCGTCTGTTG TTTGA----- ---------C TCTGGTAA-C TAGAGATCCC TCAGATCACT --CTAGTTAG T-GTGAA--- AATCTCTAGC
A1B.EE.02.EST2002_394.DQ167216    ---------- ---------G TATGA----- ---------C TCTGGTAA-C TAGAGATCCT TCAGACCACT --CTAGGCGG T-GTAAA--- AATCTCTAGC
A1BD.GB.03.34567.JN417238         ---------- ---------- ---------- ---------- ---------- ---------- ---------- ---------- ---------- ---CTCTAGC
A1C.CA.04.04CA7750.EU220698       TAGTGTGTGC CCGTCTGTTG TTTGA----- ---------C TCTGGTAA-C TAGAGATCCC TCAGACCACT --CTAGACTG T-GTAAA--- AATCTCTAGC
A1C.IN.01.1579A.DQ083238          TAGTGTGTGC CCGTCTGTTG TGTGA----- ---------C TCTGGTAA-C TAGAGATCCC TCAGACCACT --CTAGGCGG T-GTAAA--- AATCTCTAGC
A1C.IN.95.95IN21301.AF067156      ---------- ---------- ---------- ---------- ---------- ---------- ---------- ---------- --------A- AATCTCTAGC
A1C.RW.92.92RW009_06.U88823       ---------- ---------- ---------- ---------- ---------- ---------- ---------- ---------- ---------- ----------
A1CD.KE.02.ML2000.EU110093        ---------- ---------- ---------- ---------- ---------- ---------- ---------- ---------- ---------- ----------
A1CD.SE.95.SE8603.AF075702        ---------- ---------- ---------- ---------- ---------- ---------- ---------- ---------- ---------- ----------
A1CDGKU.ZA.99.CM4_99ZACM4.AF41    TAGTGTGTGC CCGTCTGTTG TGTGA----- ---------C TCTGGTAA-C TAGAGATCCC TCAGACCCTT ---TTGGTAG T-GTGGAA-- AATCTCTAGC
A1CG.KE.02.ML1979PCR.EU110096     ---------- ---------- ---------- ---------- ---------- ---------- ---------- ---------- ---------- ----------
A1D.DK.96.FSA.DQ912822            ---------- ---------- ---------- ---------- ---------- ---------- ---------- ---------- ---------- ----------
A1D.DK.98.MA.DQ912823             ---------- ---------- ---------- ---------- ---------- ---------- ---------- ---------- ---------- ----------
A1D.GB.00.8179.JN417236           ---------- ---------- ---------- ---------- ---------- ---------- ---------- ---------- ---------- --TCTCTAGC
A1D.GB.03.33365.JN417239          ---------- ---------- ---------- ---------- ---------- ---------- ---------- ---------- ---------- ----TCTAGC
A1D.GB.10.11762.JN417241          ---------- ---------- ---------- ---------- ---------- ---------- ---------- ---------- -------A-- AATCTCTAGC
A1D.GB.10.12792.JN417240          ---------- ---------- ---------- ---------- ------AA-C TAGAGATCCC TCAGACCACT --CTAGACGG T-GTAAA--- AATCTCTAGC
A1D.KE.02.ML1974.EU110090         ---------- ---------- ---------- ---------- ---------- ---------- ---------- ---------- ---------- ----------
A1D.SE.93.SE6954.AF075701         ---------- ---------- ---------- ---------- ---------- ---------- ---------- ---------- ---------- ----------
A1D.TZ.96.TZBFL0011.AF442569      ---------- ---------- ---------- ---------- ---------- ---------- ---------- ---------- ---------- ----------
A1D.TZ.96.TZBFL0088.AF442570      ---------- ---------- ---------- ---------- ---------- ---------- ---------- ---------- ---------- ----------
A1D.TZ.97.TZBFL0086.AF442566      ---------- ---------- ---------- ---------- ---------- ---------- ---------- ---------- ---------- ----------
A1D.UG.07.p191947.JX236674        TAGTGTGTGC CCGTCTATTG TGTGA----- ---------C TCTGGTAA-C TAGAGATCCC TCAGACCACT --CTAGACTG A-GTAAA--- AATCTCTAGC
A1D.UG.08.p191982.JX236675        TAGTGTGTGC CCGTCTGTTG TGTGA----- ---------C TCTGGTAA-C TAGAGATCCC TCAGACCTCT --TTAGTCAG T-GTGGAA-- AATCTCTAGC
A1D.UG.90.UG266.AY352657          ---------- ---------- ---------- ---------- ---------- ---------- ---------- ---------- ---------- ----------
A1D.UG.92.UG035.AY352656          ---------- ---------- ---------- ---------- ---------- ---------- ---------- ---------- ---------- ----------
A1DHK.NO.97.97NOGIL3.AJ237565     TAGTGTGTGC CCGTCTGTTG TGTGA----- ---------C TCTGGTAA-C TAGAGATCCC TCAGACCATT --ATAGTCAG T-GTGGAA-- AATCTCTAGC
A1DK.CD.85.MAL_MALCG.X04415       CAGTGTGTGC CCATCTGTTG TGTGA----- ---------C TCTGGTAA-C TAGAGATCCC TCAGACCACT --CTAGACGG T-GTAAA--- AATCTCTAGC
A1G.BE.94.VI1197.AJ276596         ---------- ---------- ---------- ---------- ---------- ---------- ---------- ---------- ---------- ----------
A1G.NG.92.92NG003.U88825          ---------- ---------- ---------- ---------- ---------- ---------- ---------- ---------- ---------- ----------
A1G.RU.11.11RU6900.JX500697       TAGTGTGTGC CCGTCTGTTG TGTGA----- ---------C TCTGGTAA-C TAGAGATCCC TCAGACCAAT --CTAGAGTG T-GTAAA--- AATCTCTAGC
A1G.RU.11.11RU6939.JX500706       TAGTGTGTGC CCGTCTGTTG TGTGA----- ---------C TCTGGTAA-C TAGAGATCCC TCAGACCACT --TTAGATTG A-GTAAA--- AATCTCTAGC
```

```
A1GHU.GA.x.VI354.AF076474         ---------- ---------- ---------- ---------- ---------- ---------- ---------- ---------- ---------- ----------
A1GJ.BW.98.BW2117.AF192135        ---------- ---------- ---------- ---------- ---------- ---------- ---------- --AGGGGGCC A-AGTCGG-- CCTCTCTAGC
A2C.ZA.98.DU178_98ZADU178.AF41    TAGTGTGTGC CCGTCTGTTG TGTGA----- ---------C TCTGGTAA-C TAGAGATCCC TCAGACCTTT --TGTGGTAG T-GTGGAA-- AATCTCTAGC
A2C.ZM.90.ZAM184.U86780           ---------- ---------- ---------- ---------- ---------- ---------- ---------- ---------- --------A- AATCTCTAGC
A2CD.KE.01.ML1956.EU110089        ---------- ---------- ---------- ---------- ---------- ---------- ---------- ---------- ---------- ----------
AC.KE.97.ML672.AY322191           TAGTGTGTGC CCATCTGTTG TGTGA----- ---------C TCTGGTAA-C TAGAGATCCC TCAGACCACT --CTAGACGG T-GTAAA--- AATCTCTAGC
AC.ZA.04.04ZAPS204B1.DQ093606      ---------- ---TCTGTTG TGTGA----- ---------C TCTGGTAA-C TAGAGATCCC TCAGACCCTT --GTTGGTAG T-GTGGAA-- AATCTCTAGC
AGKU.CD.x.BCF_Kita.AB485665       TAGTGTGTGC CCGTCTGTTG TGTGA----- ---------C TCTGGTAA-C TAGAGATCCC TCAGACCACT --TTAGAGCA C-GTAAA--- AATCTCTAGC
BC.AR.01.ARE195FL.AY968312        ---------- ---------- ---------- ---------- ---------- ---------- ---------- ---------- ---------- ----------
BC.BR.01.01_BR_RGS45.GQ365651     TAGTGTGTGC CCGTCTGTTG TGTGA----- ---------C TCTGGTAA-C TAGAGATCCC TCAGACACTT --GTAGTCAG T-GTGGAA-- AATCTCTAGC
BC.BR.01.01_BR_RGS69.GQ365652     TAGTGTGTGC CCGTCTGTTG TGTGA----- ---------C TCTGGTAA-C TAGAGATCCC TCAGACACCT ---TTGTCAG T-GTGGAA-- AATCTCTAGC
BC.BR.02.02_BR_HF31.GQ365649      TAGTGTGTGC CCGTCTGTTG TGTGA----- ---------C TCTGGTAA-C TAGAGATCCC TCAGACACCT --TTTGTCAG T-GTGGAA-- AATCTCTAGC
BC.BR.05.05_BR_NSP24.GQ365650     TAGTGTGTGC CCGTCTGTTG TGTGA----- ---------C TCTGGTAA-C TAGAGATCCC TCAGACACCT --TGTGTTAG T-GTGGAA-- AATCTCTAGC
BC.BR.06.06BR1114.JN692478        TAGTGTGTGC CCGTCTGTTT TGTGA----- ---------C TCTGGTAA-C TAGAGATCCC TCAGACACCT --TTTGTCAG T-GTGGAA-- AATCTCTAGC
BC.BR.92.92BR023.HM100716         TAGTGTGTGC CCGTCTGTTG TGTGA----- ---------C TCTGGTAA-C TAGAGATCCC TCAGACACCT --TTTGTCAG T-GTGGAA-- AATCTCTAGC
BC.CN.07.309.HM776938             ---------- ---------- ---------- ---------- ---------- ---------- ---------- ---------- ---------- ---------G
BC.CN.07.341.HM776939             ---------- ---------- ---------- ---------- ---------- ---------- ---------- ---------- ---------- ----------
BC.IN.02.INDNARI_0218440.EU000    ---------- ---------- ---------- ---------- ---------- ---------- ---------- ---------- --------A- AATCTCTAGC
BC.IN.02.NARI7_3.EU000511         ---------- ---------- ---------- ---------- ---------- ---------- ---------- ---------- --------A- AATCTCTAGC
BC.IN.02.NARI9_3.EU000508         ---------- ---------- ---------- ---------- ---------- ---------- ---------- ---------- --------A- AATCTCTAGC
BC.IN.99.NARI10_2.EU000516        ---------- ---------- ---------- ---------- ---------- ---------- ---------- ---------- --------A- AATCTCTAGC
BC.MM.99.mIDU103.AB097873         ---------- ---------- ---------- ---------- ---------- ---------- ---------- ---------- ---------- --------TT
BCU.FR.06.06FR_CRN.EU448296       TAGTGTGTGC CCGTCTGTTG TGTGA----- ---------C TCTGGTAA-C TAGAGATCCC TCAGACACCT --TTTGTCAT T-GTGGAA-- AATCTCTAGC
BF.BR.03.BREPM1026.EF637055       ---------- ---------- ---------- ---------- ---------- ---------- ---------- ---------- ---------- ----------
BF.BR.03.BREPM1029.EF637052       ---------- ---------- ---------- ---------- ---------- ---------- ---------- ---------- ---------- ----------
BF1.BR.00.BREPM13853.DQ085875     ---------- ---------- ---------- ---------A TCTGGTAC-T A-GAGATCCC TCAGACCATT --TGAGTCAG T-GTGGAA-- AATCTCTAGC
BF1.BR.01.01BR042.DQ358799        ---------- ---------- ---------- ---------- -----AA--C TAGAGATCCC TCAGACCATT --TTAGTCAG T-GTGGAA-- AATCTCTAGC
BF1.BR.01.01BR047.DQ358800        ---------- ---TCTGTTG TGTGA----- ---------C TCTGGTAA-C TAGAGATCCC TCAGACCATT --GTAGTCAG T-GTGGAA-- AATCTCTAGC
BF1.BR.01.01BR226.DQ358803        TAGTGTGTGC CCGTCTGTTG TGTGA----- ---------C TCTGGTAA-C TAGAGATCCC TCAGACCTTT ---TAGTCAG T-GTGGAA-- AATCTCTAGC
BF1.BR.01.01BR323.DQ358804        TAGTGTGTGC CCGTCTGTTG TGTGA----- ---------C TCTGGTAA-C TAGAGATCCC TCAGACCATT --TTAGTCCG T-GTGGAA-- AATCTCTAGC
BF1.BR.02.02BR005.DQ358806        TAGTGTGTGC CCGTCTGTTG TGTGA----- ---------C TCTGGTAA-C TAGAGATCCC TCAGACCCTT --TTAGTCAG T-GTGGAA-- AATCTCTAGC
BF1.BR.02.02BR006.DQ358807        TAGTGTGTGC CCGTCTGTTG TGTGA----- ---------C TCTGGTAA-C TAGAGATCCC TCAGACCCTT --TTAGTCCG T-GTGGAA-- AATCTCTAGC
BF1.BR.02.02BR033.DQ358811        ---------- ---------- ---------- ---------- ---------- ---------- ---------- ---------- ---------- ----------
BF1.BR.02.02BR034.DQ358812        TAGTGTGTGC CCGTCTGTTG TGTGA----- ---------C TCTGGTAA-C TAGAGATCCC TCAGACCATT --TTAGTCAG T-GTGGAA-- AATCTCTAGC
BF1.BR.02.02BR2028.JN692437       ---------- ---------- ---------- ---------- ---------- -----CCCTC AGACCTTTTA -----GTCAG T-GTGGAA-- AATCTCTAGC
BF1.BR.03.03BR2018.JN692449       TAGTGTGTGC CCGTCTGTTG TGTG------ ---------C TCTGGTAA-C TATAGATCCC TCAGACAATT --CTAGTCAG T-GTGGAA-- AATCTATAGC
BF1.BR.03.03BR2019.JN692448       ---------- ---------- ---------- ---------- ---------- ---------- ---------- ---------- ---------- ----------
BF1.BR.04.04BR1067.JN692456       TAGTGTGTGC CCGTCTGTTG TATGA----- ---------C TCTGGTAA-C TAGAGATCCC TCAGACCAAT --TTAGTCGG A-GTAGAA-- AATCTCTAGC
BF1.BR.05.0008SP.JF804805         ---------- ---------- ---------- ---------- ---------- ---------- ---------- ---------- ---------- ----------
BF1.BR.05.0632SV.JF804810         ---------- ---------- ---------- ---------- ---------- ---------- ---------- ---------- ---------- ----------
BF1.BR.06.06BR_FPS561.HM026455    AAGTGTGTGC CCGTCTGTTG TGTGA----- ---------C TCTGGTAA-C TAGAGATCCC TCAGACCATT --TAGTCAGT G-TGGAA--- AATCTCTAGC
BF1.BR.99.BREPM107.AY771588       ---------- ---------- ---------- ---------- ---------- ---------- ---------- ---------- ---------- ----------
BF1.BR.99.BREPM108.AY771589       TAGTGTGTGC CCGTCTGTTG TGTGA----- ---------C TCTGGTAA-C TAGAGATCCC TCAGACCATC --TTAGTCAG T-GTGGAA-- AATCTCTAGC
BF1.BR.99.BREPM11931.DQ085869     ---------- ---------- ---------- ---------C TGGTACT--- --GAGATCCC TCTGACCTTT ---TAGTCCG T-GTGGAA-- AATCTCTAGC
BF1.ES.08.ES_X2524_2.GQ372989     TAGTGTGTGC CCGTCTGTTG TGTGA----- ---------C TCTGGTAA-C TAGAGATCCC TCAGACTATT --TTAGTCAG A-GTAGAA-- AATCTCTAGC
BF1.ES.08.X2432_2.FJ853621        -AGTGTGTGC CCGTCTGTTG TGTGACTCTG GTAACTAA-C TCTGGTGA-C TAGAGATCCC TCAGACCCTT --TTAGTCAG T-GTGGAA-- AATCTCTAGC
BF1.ES.09.DEMBF09ES003.JX14066    ---------- ---------- ---------- ---------- ---------- ---------- ---------- ---------- ---------- ----------
BF1.ES.09.DEMBF09ES006.JX14066    ---------- ---------- ---------- ---------- ---------- ---------- ---------- ---------- ---------- ----------
BF1.IT.01.53143.GU595149          ---------- ---------- ---------- ---------- ---------- ---------- ---------- ---------- ---------- ----------
BF1.IT.02.30638.GU595148          ---------- ---------- ---------- ---------- ---------- ---------- ---------- ---------- ---------- ----------
BF1.IT.02.57954.GU595150          TAGTGTGTGC CCGTCTGTTG TGTGA----- ---------C TCTGGTAA-A TAGAGATCCC TCAGACCATT --TTAGTCAG T-GTGGAA-- AATCTCTAGC
BF1.IT.02.58736.GU595160          TAGTGTGTGC CCGTCTGTTG TGTGA----- ---------C TCTGGTAA-C TAGAGATCCC TCAGACCATT --TAAGTCAG T-GTGGAA-- AATCTCTAGC
BF1.IT.02.59211.GU595151          TAGTGTGTGC CCGTCTGTTG TGTGA----- ---------C TCTGGTAA-C TAGAGATCCC TCAGACACTT --TTAGTCAG T-GTGGAA-- AATCTCTAGC
BF1.IT.05.83166.GU595152          TAGTGTGTGC CCATCTGTTG TTTGA----- ---------C TCCGGTTA-C TAGAGATCCC TCAGACCATT --TTAGTCCG T-GTGGAA-- AATCTCTAGC
BF1.IT.06.89072.GU595153          TAGTGTGTGC CCGTCTGTTG TGTGA----- ---------C TCTGGTAA-C TAGAGACCCC TCAGACCCTT --TTAGCCAG T-GTGGAA-- AATCTCTAGC
BF1.JP.04.DR6082.AB480298         TAGTGTGTGC CCATCTGTTG TGTGA----- ---------C TCTGGTAG-C TAGAGATCCC TCAGACCATT --TTAGTCAG T-GTGGAA-- AATCTCTAGC
BF1.JP.04.DR6190.AB480300         TAGTGTGTGC CCGTCTGTTG TGTGA----- ---------C TCTGGTAA-C TAGAGATCCC TCAGACCATT --TTAGTCAG T-GTGGAA-- AATCTCTAGC
BF1.JP.x.DR0769.AB253430          TAGTGTGTGC CCGTCTGTTG TGTGA----- ---------C TCTGGTAA-C TAGAGATCCC TCAGACCCTT --TTAGTCAG T-GTGGAA-- AATCTCTAGC
BFG.MO.05.MO108.GU207884          ---------- ---------- ---------- ---------- ---------- ---------- ---------- ---------- ---------- ----------
BG.CU.x.Cu100.AY586546            TAGTGTGTGC CCGTCTGTTG TGTGA----- ---------C TCTGGTAA-C TAGAGATCCC TCAGACCACT --CTAGACAG T-GTAAA--- AATCTCTAGC
BG.DE.01.9196_01.AY882421         TAGTGTGTGC CCGTCTGTTG TGTGA----- ---------C TCTGGTAA-C TAGAGATCCC TCAGACCACT --CTAAATAG T-GTAA---- AATCTCTAGC
CD.KE.01.ML1076.EU110086          ---------- ---------- ---------- ---------- ---------- ---------- ---------- ---------- ---------- ----------
CU.JP.04.DR5782.AB286849          CAGTGTGTGC CCGTCTGTTG TGTGA----- ---------C TCTGGTAA-C TAGAGATCCC TCAGACCTTT --TAGTCAGT --GTGGAA-- AATCTCTAGC
DF1G.ES.04.X963_4.FJ670527        -TAAGGGTGC CCGTCTGTTA TGTGA----- ---------C TCTGGTAA-C TAGAGATCCC TCAGACCACT --CTAGATAG T-GTAAA--- AATCTCTAGC
DO.FR.08.RBF208.GQ351296          ---------- TCATCTGTT- --CAA----- ---------C CCTGGTAT-C TAGAGATCCC TCAGATCACG --TAGACTGA G-GYAGAA—AATCTCTAGC
```

```
                                   ....|....| ....|....| ....|....| ....|....| ....|....| ....|....| ....|....| ....|....| ....|....| ....|....|
                                         105        115        125        135        145        155        165        175        185        195
B.FR.83.HXB2_LAI_IIIB_BRU.K034     AG-TGGCGCC CGAACAGG-- ---------- ---------- ---GACCTGA AA-GCGAAAG GGAAA----- ---------- ---------- ----------
A1.CD.02.02CD_KTB035.AM000055      AT-TGGCGCC CAACCAGG-- ---------- ---------- ---GACTCGA AA-GCGAAAG TT-------- ---------- ---------- ----------
A1.CD.97.97CD_KCC2.AM000053        AG-TGGCGCC CGAACAGGGA CTCG------ ---------- ---AAAGCGA AA-GTAAAAG TT-------- ---------- ---------- ----------
A1.ES.05.X1608_8.FJ670519          AG-TGGCGCC CGAACAGG-- ---------- ---------- ---GACTCGA AA-GCGAAAG TT-------- ---------- ---------- ----------
A1.IT.02.60000.EU861977            AG-TGGCGCC CGAACAGG-- ---------- ---------- ---GACTCGA AA-GCGAAAG TT-------- ---------- ---------- ----------
A1.KE.01.ML1901PCR2.EU110095       ---------- ---------- ---------- ---------- ---------- --------TG TT-------- ---------- ---------- ----------
A1.KE.01.ML1945.EU110088           ---------- ---------- ---------- ---------- ---------- ---------- -T-------- ---------- ---------- ----------
A1.KE.02.ML1990.EU110092           ---------- ---------- ---------- ---------- ---------- ---------- TT-------- ---------- ---------- ----------
A1.KE.02.ML2014.EU110094           ---------- ---------- ---------- ---------- ---------- ---------- -T-------- ---------- ---------- ----------
A1.KE.86.ML170_1986.AF539405       ---------- ---------- ---------- ---------- -------GGA AA-GCGAAAG TT-------- ---------- ---------- ----------
A1.KE.94.Q23_17.AF004885           AG-TGGCGCC CGAACAGG-- ---------- ---------- ---GACTCGA AA-GCGAAAG TT-------- ---------- ---------- ----------
A1.KE.97.ML013_2.AY322185          AG-TGGCGCC CGAACAGG-- ---------- ---------- ---GACTCGA AA-GCGAAAG TT-------- ---------- ---------- ----------
A1.KE.97.ML605_3.AY322190          AG-TGGCGCC CGAACAGGGA CTTGAAA-GC GAAAGTAAGC AGGGACTCGA AA-GCGAAAG TT-------- ---------- ---------- ----------
A1.KE.97.ML752.AY322193            AG-TGGCGCC CGAACAGG-- ---------- ---------- ---GACTTGA AA-GCGAAAG TT-------- ---------- ---------- ----------
A1.RU.00.RU00051.EF545108          ---------- ---------- ---------- ---------- ---------- ---------- ---------- ---------- ---------- ----------
A1.RU.05.RU_560_1125_JA.JQ2928     ---------- ---------- ---------- ---------- ---------- ---------- ---------- ---------- ---------- ----------
A1.RU.06.RU_915_1016.JQ292896      AG-TGGCGCC CGAACAGG-- ---------- ---------- ---GACTCGA AA-GCGAAAG TT-------- ---------- ---------- ----------
A1.RU.06.RU_915_1035.JQ292897      AG-TGGCGCC CGAACAGG-- ---------- ---------- ---GACTTGA AA-GCGAAAG TT-------- ---------- ---------- ----------
A1.RU.06.RU_915_1038.JQ292898      AG-TGGCGCC CGAACAGG-- ---------- ---------- ---GACTTGA AA-GCGAAAG TT-------- ---------- ---------- ----------
A1.RU.06.RU_915_1041.JQ292899      AG-TGGCGCC CGAACAGG-- ---------- ---------- ---GACTCGA AA-GCGAAAG TT-------- ---------- ---------- ----------
A1.RU.06.RU_SP_B_049.JQ292900      AG-TGGCGCC CGAACAGG-- ---------- ---------- ---GACTTGA AA-GCGAAAG TT-------- ---------- ---------- ----------
A1.RU.07.Irkutsk_5.JQ292891        ---------- ---------- ---------- ---------- ---------- ---------- ---------- ---------- ---------- ----------
A1.RU.08.PokA1Ru.FJ864679          AG-TGGCGCC CGAACAGGGA CTTGAAA-GC GAAAGTTAAC AGGGACTCGA AA-GCGAAAG TT-------- ---------- ---------- ----------
A1.RU.08.RUA001.JQ292893           AG-TGGCGCC CGAACAGG-- ---------- ---------- ---GACTCGA AA-GCGAAAG TT-------- ---------- ---------- ----------
A1.RU.08.RUA007.JQ292894           AG-TGGCGCC CGAACAGG-- ---------- ---------- ---GACTCGA AA-GCGAAAG TT-------- ---------- ---------- ----------
A1.RU.10.10RU6617.JX500696         AG-TGGCGCC CGAACAGG-- ---------- ---------- ---GACTTGA AA-GCGAAAG TG---TT--- ---------- ---------- ----------
A1.RU.10.10RU6792.JX500695         AG-TGGCGCC CGAACAGG-- ---------- ---------- ---GACTCGA AA-GCGAAAG TT-------- ---------- ---------- ----------
A1.RU.11.11RU6950.JX500694         AG-TGGCGCC CGAACAGG-- ---------- ---------- ---GACTTGA AA-GCGAAAG TT-------- ---------- ---------- ----------
A1.RW.07.pR463F.JX236677           AG-TGGCGCC CGAACAGGGA CTTGAAA-GT GAAAGTTAAT AGGGACTTGA AA-GCGAAAG TT-------- ---------- ---------- ----------
A1.RW.07.pR880F.JX236678           AG-TGGCGCC CGAACAGG-- ---------- ---------- ---GACTTGA AA-GCGAAAG TG---TT--- ---------- ---------- ----------
A1.RW.92.92RW008.AB253421          AG-TGGCGCC CGAACAGGGA CTTGAAA-AG CGAAAGTAAC AGGGACTCGA AA-GCGAAAG TT-------- ---------- ---------- ----------
A1.RW.92.92RW025A.AB287376         AG-TGGCGCC CGAACAGG-- ---------- ---------- ---GACTCGA AA-GCGAAAG TAAGA----- ---------- ---------- ----------
A1.RW.93.93RW037A.AB287379         AG-TGGCGCC CGAACAGG-- ---------- ---------- ---GACTCGA AA-GCGAAAG TT-------- ---------- ---------- ----------
A1.SE.95.UGSE8131.AF107771         AG-TGGCGCC CGAACAGGGA CCCGAAA-AG TGAAAGTAAT AGGGACTCGA AA-GCGAAAG TT-------- ---------- ---------- ----------
A1.TZ.97.97TZ03.AF361873           ---------- ------GGAC CCTAA---AG TGAAAGTAAT AGGGACTCGA AA-GCGAAAG TT-------- ---------- ---------- ----------
A1.UA.00.98UA0116.AF413987         AG-TGGCGCC CGAACAGG-- ---------- ---------- ---GACTCGA AA-GCGAAAG TT-------- ---------- ---------- ----------
A1.UG.07.p191084.JX236669          AG-TGGCGCC CGAACAGG-- ---------- ---------- ---GACTCGA AA-GCGAAAG TG---TT--- ---------- ---------- ----------
A1.UG.07.p191845.JX236671          AG-TGGCGCC CGAACAGGGA CTTTAAAAGC GAAAGT-AAC AGGGACTCGA AA-GCGGAAG TT-------- ---------- ---------- ----------
A1.UG.07.p9004SDM.JX236676         AG-TGGCGCC CGAACAGG-- ---------- ---------- ---GACTTGA AA-GCGAAAG TT-------- ---------- ---------- ----------
A1.UG.85.U455_U455A.M62320         AG-TGGCGCC CGAACAGG-- ---------- ---------- ---GACTCGA AA-GCGAAAG TT-------- ---------- ---------- ----------
A1.UG.92.92UG037.AB253429          AG-TGGCGCC CGAACAGGGA CTTGAAA-GC GAAAGT-AAT AGGGACTCGA AA-ACGAAAG TT-------- ---------- ---------- ----------
A1.UG.92.UG029.AB098333            AG-TGGCGCC CGAACAGG-- ---------- ---------- ---GACTCGA AA-GCGAAAG TT-------- ---------- ---------- ----------
A1.UG.x.UG031.AB098331             AG-TGGCGCC CGAACAGG-- ---------- ---------- ---GACTCGA AA-GCGAAAG TT-------- ---------- ---------- ----------
A1.UG.x.UG275.AB485632             AG-TGGCGCC CGAACAGG-- ---------- ---------- ---GACTCGA AA-GCGAAAG TT-------- ---------- ---------- ----------
A1.ZA.04.04ZASK162B1.DQ396400      AG-TGGCGCC CGAACAGG-- ---------- ---------- ---GACTCGA AA-GCGAAAG TT-------- ---------- ---------- ----------
A2.CD.97.97CDKTB48.AF286238        ---------- ---------- ---------- ---------- ---TTTTTCG AA-GCGAAG- TA-------- ---------- ---------- ----------
A2.CY.94.94CY017_41.AF286237       AA-AGCGAAA GTAACAGG-- ---------- ---------- ---GACTCGA AA-GCGAAAG TT-------- ---------- ---------- ----------
B.AU.86.MBC200.AF042100            AG-TGGCGCC CGAACAGG-- ---------- ---------- ---GACTTGA AA-GCGAAAG GAAAA----- ---------- ---------- ----------
B.AU.87.MBC925.AF042101            AG-TGGCGCC CGAACAGG-- ---------- ---------- ---GACTTGT AA-GCGAAAG AGAAA----- ---------- ---------- ----------
B.AU.95.C24.AF538304               AG-TGGCGCC CGAACAGG-- ---------- ---------- ---GACTTGA AA-GCGAAAG AGAAA----- ---------- ---------- ----------
B.AU.95.C42.AF538305               AG-TGGCGCC CGAACAGG-- ---------- ---------- ---GACCGGA AA-GCGAAAG AAAAA----- ---------- ---------- ----------
B.AU.95.C76.AF538306               AG-TGGCGCC CGAACAGG-- ---------- ---------- ---GTCTTGA AA-GCGAAAG AGAAA----- ---------- ---------- ----------
B.AU.95.C92.AF538307               AG-TGGCGCC CGAACAGG-- ---------- ---------- ---GACTTGA AA-GCGAAAG AGAAA----- ---------- ---------- ----------
B.AU.95.MBCC54.AF042103            --------CC CGAACAGG-- ---------- ---------- ---GACTTGA AA-GCGAAAG AGAAA----- ---------- ---------- ----------
B.AU.96.MBCC98.AF042104            ---------- ---------- ---------- ---------- ---------- ---------G GAAA------ ---------- ---------- ----------
B.AU.96.MBCD36.AF042105            ---------- ---ACAGG-- ---------- ---------- ---GACTTGA AA-GCGAAAG GGAAA----- ---------- ---------- ----------
B.AU.99.1181.AF538302              AG-TGGCGCC CGAACAGG-- ---------- ---------- ---GACTTGA AA-GCGAAAG AGAAA----- ---------- ---------- ----------
B.AU.x.2870718.AY857022            ---------- ---------- ---------- ---------- ---------- ---------- ---------- ---------- ---------- ----------
B.AU.x.9125091.AY857165            ---------- ---------- ---------- ---------- ---------- ---------- ---------- ---------- ---------- ----------
B.AU.x.VH_VHPCR.AF146728           ---------- ---------- ---------- ---------- ---------- ---------- ---------- ---------- ---------- ----------
B.BO.09.DEMB09BO001.JX140656       -G-TGGCGCC CGAACAGG-- ---------- ---------- ---GACTTGA AA-GCGAAAG TAGAA----- ---------- ---------- ----------
B.BR.02.02BR008.DQ358808           AG-TGGCGCC CGAACAGG-- ---------- ---------- ---GACCTGA AA-GTGAAAG TAGAA----- ---------- ---------- ----------
B.BR.02.02BR011.DQ358809           AG-TGGCGCC CGAACAGG-- ---------- ---------- ---GACTTGA AA-GCGAAAG TAAGA----- ---------- ---------- ----------
B.BR.02.02BR013.DQ358810           AG-TGGCGCG CGAACAGG-- ---------- ---------- ---GACTAGA AA-GCGAAAG TAGAC----- ---------- ---------- ----------
B.BR.02.02BR1013.JN692432          AG-TGGCGCC CGAACAGG-- ---------- ---------- ---GACCCGA AA-GCGAAAG TAGAA----- ---------- ---------- ----------
B.BR.02.02BR2025.JN692435          AG-TGGCGCC CGAACAGG-- ---------- ---------- ---GACCCGA AAAGCGAAAG TAGAA----- ---------- ---------- ----------
B.BR.02.02BR2032.JN692439          ---------- ---------- ---------- ---------- ---------- ---------- ----T----- ---------- ---------- ----------
B.BR.02.02BR2033.JN692440          AG-TGGCGCC CGAACAGG-- ---------- ---------- ---GACTTGA AAAGCGAAAG TAAAA----- ---------- ---------- ----------
B.BR.02.02BR2041.JN692443          AG-TGGCGCC CGAACAGG-- ---------- ---------- ---GACTTGA AA-ACGAAAG TAGAA----- ---------- ---------- ----------
B.BR.02.02BR2042.JN692444          AG-TGGCGCC CGAACAGG-- ---------- ---------- ---GACCCGA AA-GCGAAAG TAGAA----- ---------- ---------- ----------
B.BR.02.04BR1064.JN692433          AG-TGGCGCC CGAACAGG-- ---------- ---------- ---GACTAGA AA-GCGAAAG TAAAA----- ---------- ---------- ----------
B.BR.02.05BR1094.JN692470          AG-TGGCGCC CGAACAGG-- ---------- ---------- ---GACGCGA AA-GCGAAAG TAGAA----- ---------- ---------- ----------
B.BR.03.03BR1020.JN692445          AG-TGGCGCC CGAACAGG-- ---------- ---------- ---GACCTTA AAAGCGAAAG TAGAA----- ---------- ---------- ----------
B.BR.03.03BR1046.JN692447          AG-TGGCGCC CGAACAGG-- ---------- ---------- ---GACGTGA AA-GCGAAAG TAAGA----- ---------- ---------- ----------
B.BR.03.BREPM1023.EF637057         AG-TGGCGCC CGAACAGG-- ---------- ---------- ---GACCTGA AA-GCGAAAG GAGAA----- ---------- ---------- ----------
B.BR.03.BREPM1024.EF637056         AG-TGGCGCC CGAACAGG-- ---------- ---------- ---GACTTGA AA-GCGAAAG TAGAA----- ---------- ---------- ----------
B.BR.03.BREPM1027.EF637054         AG-TGGCGCC CGAACAGG-- ---------- ---------- ---GACTTGA AA-GCGAAAG TAAAA----- ---------- ---------- ----------
B.BR.03.BREPM1028.EF637053         ---------- ---------- ---------- ---------- -----CTTGA AA-GCGAAAG TAGAA----- ---------- ---------- ----------
B.BR.03.BREPM1032.EF637051         ---------- ---------- ---------- ---------- ---------- ---------- ---------- ---------- ---------- ----------
B.BR.03.BREPM1033.EF637050         AG-TGGCGCC CGAACAGG-- ---------- ---------- ---GACTCGA AA-GCGAAAG TAGAA----- ---------- ---------- ----------
B.BR.03.BREPM1035.EF637049         ---------- ---------- ---------- ---------- ---------- ---------- ---------- ---------- ---------- ----------
B.BR.03.BREPM1038.EF637048         AG-TGGCGCC CGAACAGG-- ---------- ---------- ---GACTTGA AA-GCGAAAG TAACG----- ---------- ---------- ----------
B.BR.03.BREPM1040.EF637047         AG-TGGCGCC CGAACAGG-- ---------- ---------- ---GACTAGA AA-ACGAAAG TAGAA----- ---------- ---------- ----------
B.BR.03.BREPM2012.EF637046         AG-TGGCGCC CGAACAGG-- ---------- ---------- ---GACTTGA AA-GCGAAAG TAAAG----- ---------- ---------- ----------
B.BR.04.04BR1047.JN692450          AG-TGGCGCC CGAACAGG-- ---------- ---------- ---GACTTGG AA-GCGAAAG TAGAA----- ---------- ---------- ----------
B.BR.04.04BR1049.JN692451          AG-TGGCGCC CGAACAGG-- ---------- ---------- ---GACGCGA AA-GCGAAAG TAAAG----- ---------- ---------- ----------
B.BR.04.04BR1051.JN692452          AG-TGGCGCC TGAACAGG-- ---------- ---------- ---GACCCGA AA-GCGAAAG TAGGA----- ---------- ---------- ----------
B.BR.04.04BR1054.JN692453          AG-TGGCGCC CGAACAGG-- ---------- ---------- ---GACTTGA AA-GCGAAAG TAAAG----- ---------- ---------- ----------
B.BR.04.04BR1055.JN692454          AG-TGGCGCC CGAACAGG-- ---------- ---------- ---GACGCGA AA-GCGAAAG TAGAA----- ---------- ---------- ----------
B.BR.04.04BR1057.JN692455          AG-TGGCGCC CGAACAGG-- ---------- ---------- ---GACTTGA AA-ACGAAAG TAAGA----- ---------- ---------- ----------
B.BR.04.04BR1068.JN692457          AG-TGGCGCC --GAAAGG-- ---------- ---------- ---GACCCGA AA-GCGAAAG TAGAA----- ---------- ---------- ----------
B.BR.04.BREPM1066.FJ195090         AG-TGGCGCC CGAACAGG-- ---------- ---------- ---GACTCGA AA-ACGAAAG TAGAA----- ---------- ---------- ----------
B.BR.04.BREPM1070.FJ195086         GG-TGGCGCC GGACCAGG-- ---------- ---------- ---GCCTTGA AA-AGGAAAT AAGAA----- ---------- ---------- ----------
B.BR.05.05BR1077.JN692460          ---------- ---------- ---------- ---------- ---------- ---------- ----T----- ---------- ---------- ----------
B.BR.05.05BR1078.JN692461          ---------- ---------- ---------- ---------- ---------- -----AACTC TTAAT----- ---------- ---------- ----------
B.BR.05.05BR1079.JN692462          AG-TGGCGCC CGAACAGG-- ---------- ---------- ---GACTTGA AA-GTGAAAG CGGAA----- ---------- ---------- ----------
B.BR.05.05BR1080.JN692463          ---------- ---------- ---------- ---------- ---------- ---------- ----T----- ---------- ---------- ----------
B.BR.05.05BR1082.JN692465          AG-TGGCGCC CGAACAGG-- ---------- ---------- ---GACCTGA AA-GCGAAAG TGAGA----- ---------- ---------- ----------
B.BR.05.05BR1089.JN692467          ---------- ---------- ---------- ---------- ---------- ---------- ----T----- ---------- ---------- ----------
B.BR.05.05BR1092.JN692468          AG-TGGCGCC CGAACAGG-- ---------- ---------- ---GACTTAA AA-GCGAAAG TAGAA----- ---------- ---------- ----------
B.BR.05.05BR1095.JN692471          AG-TGGCGCC CGAACAGG-- ---------- ---------- ---GACCTGA AA-GCGAAAG TGAGA----- ---------- ---------- ----------
B.BR.05.05BR1101.JN692473          AG-TGGCGCC CGAACAGG-- ---------- ---------- ---GACCGGA AA-ACGAAAG TAAAA----- ---------- ---------- ----------
B.BR.05.05BR1104.JN692474          AG-TGGCGCC CGAACAGG-- ---------- ---------- ---GACTTGA AA-GTGAAAG TAGAA----- ---------- ---------- ----------
```

```
B.BR.05.05BR1107.JN692475          AG-TGGCGCC CGAACAGG-- ---------- ---------- ---GACTCGA AA-GCGAAAG TAAAG----- ---------- ---------- ----------
B.BR.05.BREPM1081.FJ195091         AG-TGGCGCC CGAACAGG-- ---------- ---------- ---GACTTGA AA-ACGAAAG TAAAA----- ---------- ---------- ----------
B.BR.05.BREPM1084.FJ195088         AG-TGGCGCC GGAACAGG-- ---------- ---------- ---GACTTGA GA-GCGAAAG TAGAA----- ---------- ---------- ----------
B.BR.05.BREPM1093.FJ195089         AG-TGGCGCC CGAACAGG-- ---------- ---------- ---GACCTGA AA-GCGAAAG TAGAA----- ---------- ---------- ----------
B.BR.06.06BR1115.JN692479          AG-TGGCGCC CGAACAGG-- ---------- ---------- ---GACCCGA AA-GCGAAAG TAGAA----- ---------- ---------- ----------
B.BR.06.06BR1119.JN692480          AG-TGGCGCC CGAACAGG-- ---------- ---------- ---GACTTGA AA-GCGAAAG TAAAG----- ---------- ---------- ----------
B.CA.06.502_1027_wg01.JF320413     AG-TGGCGCC CGAACAGG-- ---------- ---------- ---GACTTGA AA-ACGAAAG AGAAA----- ---------- ---------- ----------
B.CA.06.502_1799_FL02.JF320427     AG-TGGCGCC CGAACAGG-- ---------- ---------- ---GACCGGA AA-GCGAAAG AGAAA----- ---------- ---------- ----------
B.CA.07.502_1191_03.JF320424       AG-TGGCGCC CGAACAGG-- ---------- ---------- ---GACCCGA AA-GCGAAAG TAGAA----- ---------- ---------- ----------
B.CA.96.WC10C_4.AY314056           AG-TGGCGCC CGAACAGG-- ---------- ---------- ---GACTTGA AA-GCGAAAG AGAAA----- ---------- ---------- ----------
B.CA.97.CANB3FULL.AY779553         ---------- ---------- ---------- ---------- ---------- ---------- ---------- ---------- ---------- ----------
B.CH.03.HIV_CH_BID_V4470_2003.     ---------- ---------- ---------- ---------- ---------- ---------- ---------- ---------- ---------- ----------
B.CN.01.CNHN24.AY180905            AG-TGGCGGC CGAACAGG-- ---------- ---------- ---GACTAGA AA-GCGAAAG TGAGA----- ---------- ---------- ----------
B.CN.02.02HNsc11.DQ007903          AG-TGGCGCC CGAACAGG-- ---------- ---------- ---GACCTGA AA-GCGAAAG TGAGA----- ---------- ---------- ----------
B.CN.03.SHXDC0081.JF932492         AG-TGGCGCC CGAACAGG-- ---------- ---------- ---GACCTGA AA-GCGAAAG TGAGA----- ---------- ---------- ----------
B.CN.05.05CNHB_hp3.DQ990880        AG-TGGCGCC CGAACAGG-- ---------- ---------- ---GACGCGA AA-GCGAAAG TGAGA----- ---------- ---------- ----------
B.CN.06.CC056.JF932482             AG-TGGCGCC CGAACAGG-- ---------- ---------- ---GACTTGA AA-ACGAAAG TGAGA----- ---------- ---------- ----------
B.CN.07.AH070011.JF932468          ---------- ---------- ---------- ---------- -GGGACTTGA AA-GCGAAAG TGAGA----- ---------- ---------- ----------
B.CN.07.AH070014.JF932469          AG-TGGCGCC CGAACAGG-- ---------- ---------- ---GACTTGA AA-ACGAAAG TGAGA----- ---------- ---------- ----------
B.CN.07.AH070017.JF932470          AG-TGGCGCC CGAACAGG-- ---------- ---------- ---GACTTGA AA-GCGAAAG TGAGA----- ---------- ---------- ----------
B.CN.07.AH070018.JF932471          AG-TGGCGCC CGAACAGG-- ---------- ---------- ---GACTTGA AA-ACGAAAG TAGAA----- ---------- ---------- ----------
B.CN.07.AH070057.JF932472          AG-TGGCGCC CGAACAGG-- ---------- ---------- ---GACCTGA AA-GCGAAAG TAAGA----- ---------- ---------- ----------
B.CN.07.BJ070030.JF932473          AG-TGGCGCC CGAACAGG-- ---------- ---------- ---GACTTGA AA-GCGAAAG TAAGA----- ---------- ---------- ----------
B.CN.07.CBJC261.JF932474           AG-TGGCGCC CGAACAGG-- ---------- ---------- ---GACGCGA AA-GCGAAAG TGAGA----- ---------- ---------- ----------
B.CN.07.CBJC392.JF932475           AG-TGGCGCC CGAACAGG-- ---------- ---------- ---GACCTGA AA-GCGAAAG TGAGA----- ---------- ---------- ----------
B.CN.07.CBJC394.JF932476           AG-TGGCGCC CGAACAGG-- ---------- ---------- ---GACTTGA AA-GCGAAAG TGAGA----- ---------- ---------- ----------
B.CN.07.CBJC396.JF932477           AG-TGGCGCC CGAACAGG-- ---------- ---------- ---GACCCGA AA-GCGAAAG TAAGA----- ---------- ---------- ----------
B.CN.07.GS070017.JF932484          ---------- ---ACAGG-- ---------- ---------- ---GACGCGA AA-ACGAAAG TGAGA----- ---------- ---------- ----------
B.CN.07.GZ070002.JF932485          ---------- ---ACAGG-- ---------- ---------- ---GACTTGA AA-GCGAAAG TGAGA----- ---------- ---------- ----------
B.CN.07.GZ070030.JF932486          AG-TGGCGCC CGAACAGG-- ---------- ---------- ---GACGCGA AA-GCGAAAG TAAGA----- ---------- ---------- ----------
B.CN.07.HB070006.JF932487          ---------- ----CAGG-- ---------- ---------- ---GACTTGA AA-GCGAAAG TGAGA----- ---------- ---------- ----------
B.CN.07.HB070022.JF932488          AG-TGGCGCC CGAACAGG-- ---------- ---------- ---GACTTGA AA-GCGAAAG TAAGA----- ---------- ---------- ----------
B.CN.07.HB070035.JF932489          AG-TGGCGCC CGAACAGG-- ---------- ---------- ---GACTTGA AA-GCGAAAG TGAGA----- ---------- ---------- ----------
B.CN.07.JL070038.JF932490          ---------- ---------- ---------- ---------- ---------- ---------- ---------- ---------- ---------- ----------
B.CN.07.SX070080.JF932493          ---------- ---------- ---------- ---------- AGGGACCTGA AA-GCGAAAG TGAGA----- ---------- ---------- ----------
B.CN.07.hb070025.JF932499          AG-TGGCGCC CGAACAGG-- ---------- ---------- ---GACTTGA AA-GCGAAAG TAAGA----- ---------- ---------- ----------
B.CN.07.hen1345.JF932500           AG-TGGCGCC CGAACAGG-- ---------- ---------- ---GACGCGA AA-GCGAAAG TGAGA----- ---------- ---------- ----------
B.CN.08.CBJC476.JF932478           AG-TGGCGCC CGAACAGG-- ---------- ---------- ---GACTTGA AA-GCGAAAG TGAGA----- ---------- ---------- ----------
B.CN.08.CBJC489.JF932479           AG-TGGCGCC CGAACAGG-- ---------- ---------- ---GACGCGA AA-GCGAAAG TGAGA----- ---------- ---------- ----------
B.CN.08.CBJC500.JF932480           AG-TGGCGCC CGAACAGG-- ---------- ---------- ---GACTTGA AA-GCGAAAG TGAGA----- ---------- ---------- ----------
B.CN.08.CBJC503.JF932481           AG-TGGCGCC CGAACAGG-- ---------- ---------- ---GACTTGA AA-GCGAAAG TGAGA----- ---------- ---------- ----------
B.CN.08.cbjc468.JF932498           AG-TGGCGCC CGAACAGG-- ---------- ---------- ---GACTTGA AA-GTGAAAG TGAGA----- ---------- ---------- ----------
B.CN.09.YN09P0014.JF932494         AG-TGGCGCC CGAACAGG-- ---------- ---------- ---GACTTGA AA-GCGAAAG TGAGA----- ---------- ---------- ----------
B.CN.09.ZK042.JF932497             AG-TGGCGCC CGAACAGG-- ---------- ---------- ---GACTTGA AA-GCGAAAG TGAGA----- ---------- ---------- ----------
B.CN.10.DEMB10CN002.JX140658       AG-TGGCGCC CGAACAGG-- ---------- ---------- ---GACCTGA AA-GCGAAAG TAGAA----- ---------- ---------- ----------
B.CN.98.YN9802.JF932495            AG-TGGCGCC CGAACAGG-- ---------- ---------- ---GACGCGA AA-GCGAAAG TGAGA----- ---------- ---------- ----------
B.CN.98.YN9838.JF932496            ---TGGCGCC CGAACAGG-- ---------- ---------- ---GACTTGA AA-ACGAAAG TGAGA----- ---------- ---------- ----------
B.CN.99.plwj.GU177863              AG-TGGCGCC CGAACAGG-- ---------- ---------- ---GACGCGA AA-GCGAAAG TGAGA----- ---------- ---------- ----------
B.CN.x.RL42.U71182                 AG-TGGCGCC CGAACAGG-- ---------- ---------- ---GACTTGA AA-GCGAAAG TGAGA----- ---------- ---------- ----------
B.CU.99.Cu19.AY586542              AG-TGGCGCC CGAACAGG-- ---------- ---------- ---------- ---------- ---------- ---------- ---------- ----------
B.CU.99.Cu43.AY586543              AG-TGGCGCC CGAACAGG-- ---------- ---------- ---GACTTGA AA-GTGAAAG TAGAA----- ---------- ---------- ----------
B.DE.86.D31.U43096                 AG-TGGCGCC CGAACAGG-- ---------- ---------- ---GACTTGA AA-GTGAAAG TAAGA----- ---------- ---------- ----------
B.DE.86.HAN.U43141                 AG-TGGCGCC CGAACAGG-- ---------- ---------- ---GACTTGA AA-GCGAAAG TAGAA----- ---------- ---------- ----------
B.DK.01.CTL_016.EF514704           ---------- ---AC-AG-- ---------- ---------- ---GACCGGA AA-GCGAAAG AGAAA----- ---------- ---------- ----------
B.DK.01.CTL_017.EF514705           AG-TGGCGCC CCGAACAG-- ---------- ---------- --GGACTTGA AA-GCGAAAG AGAAA----- ---------- ---------- ----------
B.DK.01.CTL_018.EF514706           AG-TGGCGCC CCGGACAG-- ---------- ---------- --GGACTTGA AA-GCGAAAG AGAAA----- ---------- ---------- ----------
B.DK.01.CTL_023.EF514707           ---------- ---------- ---------- ---------- ---------- ---------- ---------- ---------- ---------- ----------
B.DK.01.CTL_030.EF514708           AG-TGGCGCC CGACAGGG-- ---------- ---------- ---AC-GCGA AA-GCGAAAG TAGAA----- ---------- ---------- ----------
B.DK.01.CTL_033.EF514709           AG-TGGCGCC CGAAACAG-- ---------- ---------- --GGACYTAA AA-GCGAAAG AGAAA----- ---------- ---------- ----------
B.DK.01.CTL_035.EF514710           ---------- ---------- ---------- ---------- ---------- ---------- ---------- ---------- ---------- ----------
B.DK.01.CTL_041.EF514711           ---------- ---------- ---------- ---------- ---------- ---------- ---------- ---------- ---------- ----------
B.DK.01.CTL_043.EF514712           ---------- ---------- ---------- ---------- ---------- ---------- ---------- ---------- ---------- ----------
B.DK.04.PMVL_012.EF514699          GT-KGCGCCC CGAACAGG-- ---------- ---------- ---GACTTGA AA-GCGAAAG AGAAA----- ---------- ---------- ----------
B.DK.04.PMVL_013.EF514700          -T-GCGGCCC CGAACAGG-- ---------- ---------- --GACCTTGA AA-GCGAAAG AGAAA----- ---------- ---------- ----------
B.DK.04.PMVL_018.EF514697          ---KGCGCCC CGAACAGG-- ---------- ---------- ---GACTTGA AA-GCGAAAG AGAAA----- ---------- ---------- ----------
B.DK.04.PMVL_025.EF514702          GT-KGCGCCC CGAACAGG-- ---------- ---------- ---GACTTGA AA-GCGAAAG AGAAA----- ---------- ---------- ----------
B.DK.04.PMVL_027.EF514698          ---GSSGCCC CGAACAGG-- ---------- ---------- ---GACCTGA AA-GCGAAAG AGAAA----- ---------- ---------- ----------
B.DK.04.PMVL_039.EF514703          --------CC CGAACAGG-- ---------- ---------- ---GACTTGA AA-GTGAAAG AGAAA----- ---------- ---------- ----------
B.DK.04.PMVL_049.EF514701          ---------- -GAACAGG-- ---------- ---------- ---GACTTGA AA-GCGAAAG TAGAA----- ---------- ---------- ----------
B.DK.07.PMVL_011.FJ694790          ---------C CGAACAGG-- ---------- ---------- ---GACTTGA AA-GTGAAAG AGAAA----- ---------- ---------- ----------
B.ES.05.X1890.EU786672             AG-TGGCGCC CGAACAGG-- ---------- ---------- ---GACTTGA AA-ACGAAAG AGAAA----- ---------- ---------- ----------
B.ES.06.X1959.EU786675             AG-TGGCGCC CGAACASG-- ---------- ---------- ---GACCCGA AA-GCGAAAG TAGAA----- ---------- ---------- ----------
B.ES.06.X2102.EU786677             AG-TGGCGCC CGAACAGG-- ---------- ---------- ---GACCCGA AA-GTGAAAG TTGAA----- ---------- ---------- ----------
B.ES.07.X2149.EU786678             AG-TGGCGCC CGAACAGG-- ---------- ---------- ---GACCGGA AA-GCGAAAG AGAAA----- ---------- ---------- ----------
B.ES.07.X2210_3.EU786679           AG-TGGCGCC CGAACAGG-- ---------- ---------- ---GACTTGA AA-GCGAAAG GGAAA----- ---------- ---------- ----------
B.ES.07.X2231.EU786680             AG-TGGCGCC CGAACAGG-- ---------- ---------- ---GACCTGA AA-GTGAAAG AGGAA----- ---------- ---------- ----------
B.ES.08.ES_X2515_3.GQ372988        AG-TGGCGCC CGAACAGG-- ---------- ---------- ---GACTTGA AAAGCGAAAG TAGAA----- ---------- ---------- ----------
B.ES.08.ES_X2556_3.GQ372990        AG-TGGCGCC CGAACAGG-- ---------- ---------- ---GACTTGA AA-GCGAAAG AGAAA----- ---------- ---------- ----------
B.ES.08.P2008.FJ670531             AG-TGGCGCC CGAACAGG-- ---------- ---------- ---GACTTGA AA-GCGAAAG GAAAA----- ---------- ---------- ----------
B.ES.08.X2425_2.FJ670525           AG-TGGCGCC CGAACAGG-- ---------- ---------- ---GACTTGA AA-GCGAAAG TAGAA----- ---------- ---------- ----------
B.ES.08.X2510_2.FJ853622           AG-TGGCGCC CGAACAGG-- ---------- ---------- ---GACTTGA AA-GTGAAAG TAAGA----- ---------- ---------- ----------
B.ES.08.X2555_2.GU362883           AG-TGGCGCC CGAACAGG-- ---------- ---------- ---GACTCGA AA-GCGAAAG AGAAA----- ---------- ---------- ----------
B.ES.08.X2574_2.GU362886           AG-TGGCGCC CGAACAGG-- ---------- ---------- ---GACTTGA AA-GCGAAAG TAGAA----- ---------- ---------- ----------
B.ES.09.P2149_3.GU362881           AG-TGGCGCC CGAACAGG-- ---------- ---------- ---GACTTGA AA-GCGAAAG TAAAA----- ---------- ---------- ----------
B.ES.09.X2689_2.GU362885           AG-TGGCGCC CGAACAGG-- ---------- ---------- ---GACGCGA AA-GCGAAAG TAGAA----- ---------- ---------- ----------
B.ES.89.U61.DQ854716               AG-TGGCGCC CGAACAGG-- ---------- ---------- ---GACTTGA AA-GCGAAAG AGAAA----- ---------- ---------- ----------
B.FR.05.DEMB05FR001.JX140652       ---------- -GAACAGG-- ---------- ---------- --GACCCTGA AA-GCGAAAG TAGAA----- ---------- ---------- ----------
B.FR.08.DEMB08FR002.JX140654       ---------- ----CAGG-- ---------- ---------- ---GACTTGA AA-GTGAAAG TTGAA----- ---------- ---------- ----------
B.GA.88.OYI_397.M26727             AG-TGGCGCC CGAACAGG-- ---------- ---------- ---GACCTTA AA-GTGAAAG TGGAA----- ---------- ---------- ----------
B.GB.05.MM45d213_GN1.HM586212      AG-TGGCGCC CGAACAGG-- ---------- ---------- ---GACTTGA AAAGTGAAAG AGAAA----- ---------- ---------- ----------
B.GB.83.CAM1.D10112                AG-TGGCGCC CGAACAGG-- ---------- ---------- ---GACTTGA AA-ACGAAAG TAGAA----- ---------- ---------- ----------
B.GB.86.GB8_46R.AJ271445           AG-TGGCGCC CGAACAGG-- ---------- ---------- ---GACTTGA AA-GTGAAAG TTGAA----- ---------- ---------- ----------
B.GB.x.MANC.U23487                 AG-TGGCGCC CGAACAGG-- ---------- ---------- ---GACCTGA AA-GCGAAAG TAGAA----- ---------- ---------- ----------
B.HK.06.HK003.FJ460500             ---------C CGAACAGG-- ---------- ---------- ---GACCTGA AA-GCGAAAG AGAA------ ---------- ---------- ----------
B.HK.06.HK004.FJ460501             ---------C CGAACAGG-- ---------- ---------- ---GACCTGA AA-GCGAAAG AGAAA----- ---------- ---------- ----------
B.IN.x.11807.EF694037              ---------- ---------- ---------- ---------- ---GACTTGA AA-GTGAAAG GAAAA----- ---------- ---------- ----------
B.IT.05.SG1.DQ672623               AG-TGGCGCC CGAACAGG-- ---------- ---------- ---GACCTGA AA-GCGAAAG AGAAA----- ---------- ---------- ----------
B.JP.00.DR2508.AB289588            AG-TGGCGCC CGAACAGG-- ---------- ---------- ---GACCTGA AA-GCGAAAG TAGAA----- ---------- ---------- ----------
B.JP.01.DR388.AB289590             AG-TGGCGCC CGAACAGG-- ---------- ---------- ---GACTTGA AA-GCGAAAG TAGAA----- ---------- ---------- ----------
B.JP.04.04JPDR6075B.AB221125       AG-TGGCGCC CGAACAGG-- ---------- ---------- ---GACGCGA AA-GCGAAAG TAGAA----- ---------- ---------- ----------
B.JP.04.DR5913.AB480696            AG-TGGCGCC CGAACAGG-- ---------- ---------- ---GACTTGA AA-GCGAAAG AGAAA----- ---------- ---------- ----------
B.JP.04.DR6089.AB286955            AG-TGGCGCC CGAACAGG-- ---------- ---------- ---GACTTGA AA-GCGAAAG AGAAA----- ---------- ---------- ----------
B.JP.04.DR6174.AB480692            AG-TGGCGCC CGAACAGG-- ---------- ---------- ---GACTTGA AA-GTGAAAG TAAGA----- ---------- ---------- ----------
B.JP.04.DR6175.AB480694            AG-TGGCGCC CGAACAGG-- ---------- ---------- ---GACTTGA AA-GCGAAAG AGAAA----- ---------- ---------- ----------
B.JP.05.DR6538.AB287363            AG-TGGCGCC CGAACAGG-- ---------- ---------- ---GACTTGA AA-GTGAAAG TAGAA----- ---------- ---------- ----------
```

```
B.JP.05.DR6737.AB287364          AG-TGGCGCC TGAACAGG-- ---------- ---------- ---GACCTGA AA-ACGAAAG CGGAA----- ---------- ---------- ----------
B.JP.05.DR7060.AB287366          AG-TGGCGCC CGAACAGG-- ---------- ---------- ---GACTTGA AA-ACGAAAG TAGAA----- ---------- ---------- ----------
B.JP.05.DR7065.AB287368          AG-TGGCGCC CGAACAGG-- ---------- ---------- --GACCTTGA AA-GCGAAAG TGGAA----- ---------- ---------- ----------
B.JP.98.DR1120.AB480698          AG-TGGCGCC CGAACAGG-- ---------- ---------- ---GACCCGA AA-TCGAAAG TAGAA----- ---------- ---------- ----------
B.JP.99.DR1348.AB287370          AG-TGGCGCC CGAACAGG-- ---------- ---------- ---GACTTGA AA-GCGAAAG TAAGA----- ---------- ---------- ----------
B.JP.x.DR1673.AB564745           AG-TGGCGCC CGAACAGG-- ---------- ---------- ---GACTTGA AA-GCGAAAG AGAAG----- ---------- ---------- ----------
B.JP.x.DR1712.AB604946           AG-TGGCGCC CGAACAGG-- ---------- ---------- ---GACCCGA AA-GCGAAAG CGGAA----- ---------- ---------- ----------
B.JP.x.DR1777.AB604948           AG-TGGCGCC CGAACAGG-- ---------- ---------- ---GACTTGA AA-GCGAAAG TAGAA----- ---------- ---------- ----------
B.JP.x.JRC03B.AB565496           AG-TGGCGCC CGAACAGG-- ---------- ---------- ---GACTTGA AA-ACGAAAG TAAGA----- ---------- ---------- ----------
B.JP.x.JRC05B.AB565497           AG-TGGCGCC CGAACAGG-- ---------- ---------- ---GACTTGA AA-GCGAAAG CAAAA----- ---------- ---------- ----------
B.JP.x.JRC65B.AB565502           AG-TGGCGCC CGAACAGG-- ---------- ---------- ---GACGCGA AA-ACGAAAG TGAGA----- ---------- ---------- ----------
B.JP.x.pJPDR0796B02.AB565478     AG-TGGCGCC CGAACAGG-- ---------- ---------- ---GACCCGA AA-GCGAAAG TAGAA----- ---------- ---------- ----------
B.JP.x.pJRC57B09.AB641836        AG-TGGCGCC CGAACAGG-- ---------- ---------- ---GACCCGA AA-ACGAAAG TAGAA----- ---------- ---------- ----------
B.KR.02.02OSG1.JQ429433          AG-TGGCGCC CGAACAGG-- ---------- ---------- ---GACTTGA AA-GCGACAG TAGAA----- ---------- ---------- ----------
B.KR.03.03HJY8.JQ316131          AG-TGGCGCC CGAACAGG-- ---------- ---------- ---GGCCTGA AA-GCGAAAG TAGAA----- ---------- ---------- ----------
B.KR.03.03KDE11.JQ316128         AG-TGGCGCC CGAACAGG-- ---------- ---------- ---GACGCGA AA-GCGAAAG TAGAA----- ---------- ---------- ----------
B.KR.03.03KGS5.JQ316132          AG-TGGCGCC CGAACAGG-- ---------- ---------- ---GACCAGA AA-GCGAAAG TAGAA----- ---------- ---------- ----------
B.KR.03.03LSH1.JQ316127          AG-TGGCGCC CGAACAGG-- ---------- ---------- ---GACCCGA AA-GCGAAAG TAGAA----- ---------- ---------- ----------
B.KR.03.03YGS3.JQ316135          AG-TGGCGCC CGAACAGG-- ---------- ---------- ---GACTTGA AA-GTGAAAG TAGAA----- ---------- ---------- ----------
B.KR.04.04CWS5.JQ316133          AG-TGGCGCC CGAACAGG-- ---------- ---------- ---GACCCGA AA-ACGAAAG TAGAA----- ---------- ---------- ----------
B.KR.04.04KJS8.JQ316130          AG-TGGCGCC CGAACAGG-- ---------- ---------- ---GACGCGA AA-GCGAAAG TAGAA----- ---------- ---------- ----------
B.KR.04.04KMH5.DQ295193          AG-TGGCGCC CGAACAGG-- ---------- ---------- ---GACTTGA AA-GCGAAAG TAGAA----- ---------- ---------- ----------
B.KR.04.04KMK5.JQ316126          AG-TGGCGCC CGAACAGG-- ---------- ---------- ---GACTTGA AA-GCGAAAG TAGAA----- ---------- ---------- ----------
B.KR.04.04LHS6.AY839827          AG-TGGCGCC CGAACAGG-- ---------- ---------- ---GACTTGA AA-GCGAAAG TGGTA----- ---------- ---------- ----------
B.KR.04.04LSK7.DQ295192          AG-TGGCGCC CGAACAGG-- ---------- ---------- ---GACTTGA AA-GCGAAAG TAGAA----- ---------- ---------- ----------
B.KR.04.04WK7_HIV_1_wk.DQ29519   AG-TGGCGCC CGAACAGG-- ---------- ---------- ---GACTTGA AA-ACGAAAG TAGAA----- ---------- ---------- ----------
B.KR.05.05CSR3.DQ837381          AG-TGGCGCC CGAACAGG-- ---------- ---------- ---GACTTGA AA-GCGAAAG GAAAA----- ---------- ---------- ----------
B.KR.05.05YJN2.JQ316134          AG-TGGCGCC CGAACAGG-- ---------- ---------- ---GACTTGA AA-ACGAAAG TAGAA----- ---------- ---------- ----------
B.KR.07.07KYY4.JQ341411          AG-TGGCGCC CGAACAGG-- ---------- ---------- ---GACTTGA AA-GCGAAAG TAGTA----- ---------- ---------- ----------
B.KR.99.99HYH2.JQ316129          AG-TGGCGCC CGAACAGG-- ---------- ---------- --GACCGAGA AA-GCGAAAG TAGAA----- ---------- ---------- ----------
B.MM.99.mSTD101.AB097870         AG-TGGCGCC CGAACAGG-- ---------- ---------- ---GACGCGA AA-GCGAAAG TAAGA----- ---------- ---------- ----------
B.NL.00.671_00T36.AY423387       AG-TGGCGCC CGAACAGG-- ---------- ---------- ---GACTTGA AG----AAAG TAAAA----- ---------- ---------- ----------
B.NL.86.3202A21_ACH3202A21.U34   AG-TGGCGCC CGAACAGG-- ---------- ---------- ---GACGCGA AA-GCGAAAG AGAAA----- ---------- ---------- ----------
B.NL.96.H434_42_A1.AY970948      AA-TGGCGCC CGAACAGG-- ---------- ---------- ---GACGCGA AA-GCGAAAG TAGAA----- ---------- ---------- ----------
B.PE.06.502_0491_wg5.JF320183    AG-TGGCGCC CGAACAGG-- ---------- ---------- ---GACCTGA AA-GCGAAAG GGAAA----- ---------- ---------- ----------
B.PE.06.502_0524_FL04.JF320008   ---------- ---------- ---------- ---------- ---------A AA-GCGAAAG CAAAA----- ---------- ---------- ----------
B.PE.06.502_0648_FL02.JF320215   AG-TGGCGCC CGAACAGG-- ---------- ---------- ---GACCGGA AA-GCGAAAG AGAAA----- ---------- ---------- ----------
B.PE.06.502_0841_FL04.JF320208   AG-TGGCGCC CGAACAGG-- ---------- ---------- ---GACCTGA AA-GCGAAAG AGAAA----- ---------- ---------- ----------
B.PE.06.502_2794_FL05.JF320244   AG-TGGCGCC CGAACAGG-- ---------- ---------- ---GACTTGA AA-GCGAAAG TAAA------ ---------- ---------- ----------
B.PE.07.502_0525_wg5.JF320191    AG-TGGCGCC CGAACAGG-- ---------- ---------- ---GACTTGA AA-GTGAAAG TAAAA----- ---------- ---------- ----------
B.PE.07.502_1047_wg5.JF320226    AG-TGGCGCC CGAACAGG-- ---------- ---------- ---GACTTGA AA-GCGAAAG TAGTA----- ---------- ---------- ----------
B.PE.07.502_1399_wg4.JF320013    AG-TGGCGCC CGAACAGG-- ---------- ---------- ---GACCCGA AA-GCGAAAG TAGAA----- ---------- ---------- ----------
B.PE.07.502_2254_FL6.JF320018    AG-TGGCGCC CGAACAGG-- ---------- ---------- --GACCTTGA AA-GCGAAAG AGAAA----- ---------- ---------- ----------
B.PE.07.502_2349_wg2.JF320028    AG-TGGCGCC CGAACAGG-- ---------- ---------- ---GACTTGA AA-GCGAAAG TTGAA----- ---------- ---------- ----------
B.PE.07.502_2622_wg1.JF320189    AG-TGGCGCC CGAACAGG-- ---------- ---------- ---GACTTGA AA-ACGAAAG CAGAA----- ---------- ---------- ----------
B.PE.07.502_2649_wg8.JF320019    AG-TGGCGCC CGAACAGG-- ---------- ---------- ---GACTTGA AA-GCGAAAG TAGAA----- ---------- ---------- ----------
B.RU.04.04RU128005.AY682547      AG-TGGCGCC CGAACAGG-- ---------- ---------- ---GACYYGA AA-GCGAAAG AGAAA----- ---------- ---------- ----------
B.RU.04.04RU129005.AY751406      AG-TGGCGCC CGAACAGG-- ---------- ---------- ---GACTTGA AA-GTGAAAG GGAAA----- ---------- ---------- ----------
B.RU.04.04RU139089.AY751407      AG-TGGCGCC CGAACAGG-- ---------- ---------- ---GACTTGA AA-GCGAAAG TAGAA----- ---------- ---------- ----------
B.RU.04.04RU139095.AY819715      AG-TGGCGCC CGAACAGG-- ---------- ---------- ---GACTTGA AA-GCGAAAG TAGAA----- ---------- ---------- ----------
B.RU.09.09RU4457.JX500709        AG-TGGCGCC CGAACAGG-- ---------- ---------- ---GACCCGA AAAGTGAAAG TAAAA----- ---------- ---------- ----------
B.RU.10.10RU6629.JX500707        AG-TGGCGCC CGAACAGG-- ---------- ---------- ---GACCTGA AA-GCGAAAG AGAAA----- ---------- ---------- ----------
B.RU.11.11RU21n.JX500708         AG-TGGCGCC CGAACAGG-- ---------- ---------- ---GACTTGA AA-GTGAAAG TAAA------ ---------- ---------- ----------
B.TH.06.AA010a_WG3.JX446800      AG-TGGCGCC CGAACAGG-- ---------- ---------- ---GACTTGA AA-GCGAAAG TAAA------ ---------- ---------- ----------
B.TH.07.AA040a_WG11.JX447156     ---------- ---------- ---------- ---------- --GGACTTGA AA-ACGAAAG TGAGA----- ---------- ---------- ----------
B.TH.90.BK132.AY173951           ---------- ---------- ---------- ---------- --GGACCTGA AA-GCGAAAG AGAAA----- ---------- ---------- ----------
B.TW.94.TWCYS_LM49.AF086817      AG-TGGCGCC CGAACAGG-- ---------- ---------- ---GACCTGA AA-GCAAAAG AGAAA----- ---------- ---------- ----------
B.US.00.14294_1.DQ853436         AG-TGGCGCC CGAACAGG-- ---------- ---------- ---GACGCGA AA-GCGAAAG TAGAA----- ---------- ---------- ----------
B.US.00.ES1_20.EF363123          AG-TGGCGCC CGAACAGG-- ---------- ---------- ---GACTTGA AA-GCGAAAG AGAAA----- ---------- ---------- ----------
B.US.00.RHPA_TF1.JN944917        AG-TGGCGCC CGAACAGG-- ---------- ---------- ---GACTTGA AA-GCGAAAG AGAAA----- ---------- ---------- ----------
B.US.00.THRO_TF1.JN944930        AG-TGGCGCC CGAACAGG-- ---------- ---------- ---GACCTAA AA-GCGAAAG TAAAA----- ---------- ---------- ----------
B.US.00.WITO_flB3.FJ496169       AG-TGGCGCC CGAACAGG-- ---------- ---------- ---GACCCGA AA-GTGAAAG TGAAT----- ---------- ---------- ----------
B.US.01.REJO_TF1.JN944911        AG-TGGCGCC CGAACAGG-- ---------- ---------- ---GACTTGA AA-GCGAAAG GGAAA----- ---------- ---------- ----------
B.US.01.TRJO_flA1.FJ496151       AG-TGGCGCC CGAACAGG-- ---------- ---------- ---GACCCGA AA-GCGAAAG AGAAA----- ---------- ---------- ----------
B.US.02.04013226_2_flH11.FJ496   AG-TGGCGCC CGAACAGG-- ---------- ---------- ---GACTTGA AA-GCGAAAG AGAAA----- ---------- ---------- ----------
B.US.04.ES10_53.EF363127         AG-TGGCGCC CGAACAGG-- ---------- ---------- ---GACACGA AA-GCGAAAG AGAAA----- ---------- ---------- ----------
B.US.04.ES4_24.EF363124          AG-TGGCGCC CGAACAGG-- ---------- ---------- ---GACTTGA AA-GCGAAAG GGAAA----- ---------- ---------- ----------
B.US.04.ES8_43.EF363126          AG-TGGCGCC CGAACAGG-- ---------- ---------- ---GACCGGA AA-GCGAAAG AGAAA----- ---------- ---------- ----------
B.US.04.SAMI_WGA1.EU547186       AG-TGGCGCC CGAACAGG-- ---------- ---------- ---GACTTGA AA-GCGAAAG TAGAA----- ---------- ---------- ----------
B.US.04.USPI71101EI7y04051pcWG   AG-TGGCGCC CGAACAGG-- ---------- ---------- ---GACTTGA AA-GCGAAAG AGAAA----- ---------- ---------- ----------
B.US.04.USPI83747EI6y04121pcWG   AG-TGGCGCC CGAACAGG-- ---------- ---------- ---GACTTGA AA-GCGAAAG AGAAA----- ---------- ---------- ----------
B.US.05.04013396_0_flE6.FJ4960   AG-TGGCGCC CGAACAGG-- ---------- ---------- ---GACTTGA AA-GCGAAAG AGAAA----- ---------- ---------- ----------
B.US.05.502_0223_wg1.JF320059    AG-TGGCGCC CGAACAGG-- ---------- ---------- ---GACCGGA AA-GCGAAAG AGAAA----- ---------- ---------- ----------
B.US.05.502_1400_FL02.JF320043   AG-TGGCGCC TGAACAGG-- ---------- ---------- ---GACTTGA AA-GCGAAAG AGAAA----- ---------- ---------- ----------
B.US.05.502_2136_FL02.JF320185   AG-TGGCGCC CGAACAGG-- ---------- ---------- ---GACTTGA AA-GCGAAAG AGAAA----- ---------- ---------- ----------
B.US.05.502_2495_wg02.JF320054   AG-TGGCGCC CGAACAGG-- ---------- ---------- ---GACCTGA AA-GCGAAAG AGAAA----- ---------- ---------- ----------
B.US.05.CR0307R.FJ469717         AG-TGGCGCC CGAACAGG-- ---------- ---------- ---GACGCGA AA-GCGAAAG TAGAA----- ---------- ---------- ----------
B.US.05.USPI38417EI33y05051pcW   AG-TGGCGCC CGAACAGG-- ---------- ---------- ---GACTTGA AAAGCGAAAG TAAAA----- ---------- ---------- ----------
B.US.05.USPI88403EI14y05121pcW   AG-TGGCGCC CGAACAGG-- ---------- ---------- ---GACTTGA AA-GCGAAAG AGAAA----- ---------- ---------- ----------
B.US.06.502_0341_FL05.JF320003   AG-TGGCGCC CGAACAGG-- ---------- ---------- ---GACTTGA AA-GCGAAAG AGAAA----- ---------- ---------- ----------
B.US.06.502_1046_FL04.JF320564   AG-TGGCGCC CGAACAGG-- ---------- ---------- ---GACTTGA AA-GCGAAAG TAAGA----- ---------- ---------- ----------
B.US.06.502_1174_FL09.JF320053   AG-TGGCGCC CGAACAGG-- ---------- ---------- ---GACTTGA AA-GCGAAAG TAGAA----- ---------- ---------- ----------
B.US.06.502_1211_FL01.JF320151   AG-TGGCGCC CGAACAGG-- ---------- ---------- ---GACGCGA AA-GCGAAAG TAGAA----- ---------- ---------- ----------
B.US.06.502_1619_FL06.JF320126   AG-TGGCGCC CGAACAGG-- ---------- ---------- ---GACCCGA AA-GTGAAAG AAGAA----- ---------- ---------- ----------
B.US.06.502_2667_FL03.JF320145   AG-TGGCGCC CGAACAGG-- ---------- ---------- ---GACTTGA AA-GCGAAAG AGAAA----- ---------- ---------- ----------
B.US.06.CH106_TF1.JN944897       AG-TGGCGCC CGAACAGG-- ---------- ---------- ---GACTTGA AA-ACGAAAG TAGAA----- ---------- ---------- ----------
B.US.06.CH40E_flA1.FJ495818      AG-TGGCGCC CGAACAGG-- ---------- ---------- ---GACTTGA AA-GTGAAAG AGAAA----- ---------- ---------- ----------
B.US.06.CH58E_flA9.FJ495941      AG-TGGCGCC CGAACAGG-- ---------- ---------- ---GACCTGA AA-GCGAAAG TAGAA----- ---------- ---------- ----------
B.US.06.CH77E_flA1.FJ496000      AG-TGGCGCC CGAACAGG-- ---------- ---------- ---GACGCGA AA-GCGAAAG AGAAA----- ---------- ---------- ----------
B.US.07.502_0364_wg2.JF320563    AG-TGGCGCC CGAACAGG-- ---------- ---------- ---GACTTGA AA-GCGAAAG AGAAA----- ---------- ---------- ----------
B.US.07.502_1115_wg1.JF320045    AG-TGGCGCC CGAACAGG-- ---------- ---------- ---GACTTGA AA-GTGAAAG AGAAA----- ---------- ---------- ----------
B.US.07.502_1478_wg4.JF320150    AG-TGGCGCC CGAACAGG-- ---------- ---------- ---GACGCGA AA-GCGAAAG TAGGA----- ---------- ---------- ----------
B.US.07.502_1897_wg6.JF320182    AG-TGGCGCC CGAACAGG-- ---------- ---------- ---GACCGGA AA-GCGAAAG TAAAA----- ---------- ---------- ----------
B.US.07.502_2289_05.JF320197     AG-TGGCGCC CGAACAGG-- ---------- ---------- ---GACTTGA AA-GCGAAAG TAGAA----- ---------- ---------- ----------
B.US.09.C1P.GU733713             AG-TGGCGCC CGAACAGG-- ---------- ---------- ---GACCAGA AA-GCGAAAG TAGAA----- ---------- ---------- ----------
B.US.09.DEMB09US002.JX140657     -----GCGCC CGAACAGG-- ---------- ---------- ---GACCTGA AA-GCGAAAG AGAAA----- ---------- ---------- ----------
B.US.10.CP1.JN397365             AG-TGGCGCC CGAACAGG-- ---------- ---------- ---GACTTGA AA-GCGAAAG AGAAA----- ---------- ---------- ----------
B.US.10.VC1.JN397364             AG-TGGCGCC CGAACAGG-- ---------- ---------- ---GACCGGA AA-GCGAAAG AGAAA----- ---------- ---------- ----------
B.US.11.ES38.JN397362            AG-TGGCGCC CGAACAGG-- ---------- ---------- ---GACCTTA AAAGCGAAAG AGAAA----- ---------- ---------- ----------
B.US.83.5018_83.AY835777         AG-TGGCGCC CGAACAGG-- ---------- ---------- ---GACTTGA AA-GCGAAAG TAAAG----- ---------- ---------- ----------
B.US.83.5157_83.AY835781         AG-TGGCGCC CGAACAGG-- ---------- ---------- ---GACTTGA AA-GCGAAAG TAAAG----- ---------- ---------- ----------
B.US.83.RF_HAT3.M17451           AG-TGGCGCC CGAACAGG-- ---------- ---------- ---GACCAGA AA-GCGAAAG TAGAA----- ---------- ---------- ----------
B.US.83.SF2_LAV2_ARV2.K02007     AG-TGGCGCC CGAACAGG-- ---------- ---------- ---GACGCGA AA-GCGAAAG TAGAA----- ---------- ---------- ----------
B.US.84.5019_84.AY835779         AG-TGGCGCC CGAACAGG-- ---------- ---------- ---GACTTGA AA-GCGAAAG TAAAG----- ---------- ---------- ----------
B.US.84.MNCG_MN.M17449           AG-TGGCGCC CGAACAGG-- ---------- ---------- ---GACTTGA AA-GCGAAAG AAAAA----- ---------- ---------- ----------
```

```
B.US.84.NY5CG.M38431            AG-TGGCGCC CGAACAGG-- ---------- ---------- ---GACTTGA GA-GCGAAAG TAAAG----- ---------- ---------- ----------
B.US.84.SF33.AY352275           AG-TGGCGCC CGAACAGG-- ---------- ---------- ---GACTTGA AA-GTGAAAG TTAA------ ---------- ---------- ----------
B.US.85.5077_85.AY835769        AG-TGGCGCC CGAACAGG-- ---------- ---------- ---GACTTGA AA-GCGAAAG TAAAG----- ---------- ---------- ----------
B.US.85.Ba_L.AB221005           AG-TGGCGCC CGAACAGG-- ---------- ---------- ---GACTTGA AA-GCGAAAG AGAAA----- ---------- ---------- ----------
B.US.86.5084_86.AY835775        AG-TGGCGCC CGAACAGG-- ---------- ---------- ---GACTTGA AA-GCGAAAG TAAAG----- ---------- ---------- ----------
B.US.86.5096_86.AY835749        AG-TGGCGCC CGAACAGG-- ---------- ---------- ---GACTTGA AA-GCGAAAG GGAAA----- ---------- ---------- ----------
B.US.86.5127_86.AY835774        AG-TGGCGCC CGAACAGG-- ---------- ---------- ---GACTTGA AA-GCGAAAG TAAAG----- ---------- ---------- ----------
B.US.86.AD87_ADA.AF004394       AG-TGGCGCC CGAACAGG-- ---------- ---------- ---GACTTGA AA-GTGAAAG TAGAA----- ---------- ---------- ----------
B.US.86.JRFL_JR_FL.U63632       ---------- ---------- ---------- ---------- ---------  ---------- ---------- ---------- ---------- ----------
B.US.86.YU_2.M93258             AG-TGGCGCC CGAACAGG-- ---------- ---------- ---GACTTGA AA-GCGAAAG GAAAA----- ---------- ---------- ----------
B.US.87.5113_87.AY835758        AG-TGGCGCC CGAACAGG-- ---------- ---------- ---GACTTGA AA-GCGAAAG TAAAG----- ---------- ---------- ----------
B.US.87.BC_BCSG3.L02317         AG-TGGCGCC CGAACAGG-- ---------- ---------- ---GACCGGA AA-GCGAAAG AGAAA----- ---------- ---------- ----------
B.US.88.5160_88.AY835763        AG-TGGCGCC CGAACAGG-- ---------- ---------- ---GACTTGA AA-GCGAAAG TAAAG----- ---------- ---------- ----------
B.US.88.WR27.AF286365           AG-TGGCGCC CGAACAGG-- ---------- ---------- ---GACCTAA AA-GCGAAAG TAGAA----- ---------- ---------- ----------
B.US.89.P896_89_6.U39362        AG-TGGCGCC CGAACAGG-- ---------- ---------- ---GACCGGA AA-GCGAAAG AGAAA----- ---------- ---------- ----------
B.US.90.US1.AY173952            ---------- ---------- ---------- ---------- --GGACCTGA AA-GCGAAAG AGAAA----- ---------- ---------- ----------
B.US.90.US2.AY173953            ---------- ---------- ---------- ---------- --GGACTTGA AA-GCGAAAG GGAAA----- ---------- ---------- ----------
B.US.90.US3.AY173954            ---------- ---------- ---------- ---------- --GGACTAGA AA-GCGAAAG TAAAA----- ---------- ---------- ----------
B.US.90.US4.AY173955            ---------- ---------- ---------- ---------- --GGACCCGA AA-GCGAAAG TAGAA----- ---------- ---------- ----------
B.US.90.WCIPR.U69591            AG-TGGCGCC CGAACAGG-- ---------- ---------- ---GACTTGA AA-GCGAAAG GGAAA----- ---------- ---------- ----------
B.US.90.WEAU160_GHOSH.U21135    AG-TGGCGCC CGAACAGG-- ---------- ---------- ---GACCCGA AA-GCGAAAG GGAAA----- ---------- ---------- ----------
B.US.91.5048_91.AY835761        AG-TGGCGCC CGAACAGG-- ---------- ---------- ---GACTTGA AA-GCGAAAG TAAAG----- ---------- ---------- ----------
B.US.91.DH12_3.AF069140         AG-TGGCGCC CGAACAGG-- ---------- ---------- ---GACCGGA AA-GCGAAAG AGAAA----- ---------- ---------- ----------
B.US.91.SUMA_flC5.FJ496145      AG-TGGCGCC CGAACAGG-- ---------- ---------- ---GACCGGA AA-GCGAAAG AGAAA----- ---------- ---------- ----------
B.US.93.WCD32P0793.DQ487188     AG-TGGCGCC CGAACAGG-- ---------- ---------- --GACGCTGA AA-GTGAAAG AGAAA----- ---------- ---------- ----------
B.US.93.WCM32P0793.DQ487190     AG-TGGCGCC CGAACAGG-- ---------- ---------- ---GACTTGA AA-GCGAAAG GAAAA----- ---------- ---------- ----------
B.US.94.5082_94.AY835773        AG-TGGCGCC CGAACAGG-- ---------- ---------- ---GACTTGA AA-GCGAAAG TAAAG----- ---------- ---------- ----------
B.US.95.5073_95.AY835768        AG-TGGCGCC CGAACAGG-- ---------- ---------- ---GACTTGA AA-GCGAAAG TAAAG----- ---------- ---------- ----------
B.US.95.USPI90770EI72y95091pcW  AG-TGGCGCC CGAACAGG-- ---------- ---------- ---GACTTGA AA-ACGAAAG AGAAA----- ---------- ---------- ----------
B.US.96.5155_96.AY835753        AG-TGGCGCC CGAACAGG-- ---------- ---------- ---GACTTGA AA-GCGAAAG TAAAG----- ---------- ---------- ----------
B.US.96.USPI55751EI32y96071pcW  AG-TGGCGCC CGAACAGG-- ---------- ---------- ---GACCCGA AA-GCGAAAG TAGAA----- ---------- ---------- ----------
B.US.97.ARES2.AB078005          AG-TGGCGCC CGAACAGG-- ---------- ---------- ---GACTTGA AA-GCGAAAG AGAAA----- ---------- ---------- ----------
B.US.98.15384_1.DQ853463        AG-TGGCGCC CGAACAGG-- ---------- ---------- ---GACGCGA AA-GCGAAAG TAAAA----- ---------- ---------- ----------
B.US.98.98USHVTN1925c1.AY56010  AG-TGGCGCC CGAACAGG-- ---------- ---------- ---GACCTGA AA-GCGAAAG AGAAA----- ---------- ---------- ----------
B.US.98.98USHVTN3605c9.AY56010  AG-TGGCGCC CGAACAGG-- ---------- ---------- ---GACTTGA AA-GCGAAAG AGAAA----- ---------- ---------- ----------
B.US.98.98USHVTN8229c6.AY56010  AG-TGGCGCC CGAACAGG-- ---------- ---------- ---GACCCGA AA-GTGAAAG TAGGTGAA-- ---------- ---------- ----------
B.US.98.98USHVTN941c1.AY560110  AG-TGGCGCC CGAACAGG-- ---------- ---------- ---GACCAGA AA-GCGAAAG TAGAA----- ---------- ---------- ----------
B.US.98.WC3_0498_4.EF175212     AG-TGGCGCC CGAACAGG-- ---------- ---------- ---GACTCGA AA-GCGAAAG TAGAA----- ---------- ---------- ----------
B.US.x.CR0059T.FJ469694         AG-TGGCGCC CGAACAGG-- ---------- ---------- ---GACTTGA AA-GCGAAAG AGAAA----- ---------- ---------- ----------
B.US.x.sample_C_BID_D617.JX503  TT-------- -GAACAGG-- ---------- ---------- ---GACCTGA AA-GTGAAAG TAGA------ ---------- ---------- ----------
B.VE.10.DEMB10VE001.JX140659    ---------- ------GG-- ---------- ---------- ---GACGCGA AA-GCGAAAG TAGAA----- ---------- ---------- ----------
B.ZA.03.03ZAPS045MB2.DQ396398   AG-TGGCGCC CGAACAGG-- ---------- ---------- ---GACCTGA AA-GCGAAAG AGAAA----- ---------- ---------- ----------
C.BR.02.02BR2022.JN692434       AG-TGGCGCC CGAACAGG-- ---------- ---------- ---GACTTGA AA-GCGAAAG TAAGA----- ---------- ---------- ----------
C.BR.04.04BR013.AY727522        AG-TGGCGCC CGAACAGG-- ---------- ---------- ---GACTTGA AA-GCGAAAG TAGAA----- ---------- ---------- ----------
C.BR.04.04BR021.AY727523        AG-TGGCGCC CGAACAGG-- ---------- ---------- ---GACTTGA AA-GCGAAAG TAAGA----- ---------- ---------- ----------
C.BR.04.04BR038.AY727524        AG-TGGCGCC CGAACAGG-- ---------- ---------- ---GACTTGA AA-GCGAAAG TAGAA----- ---------- ---------- ----------
C.BR.04.04BR073.AY727525        AG-TGGCGCC CGAACAGG-- ---------- ---------- ---GACTTGA AA-GCGAAAG TAAGA----- ---------- ---------- ----------
C.BR.07.DEMC07BR003.JX140663    --------CC CGAACAGG-- ---------- ---------- ---GACTCGA AA-GCGAAAG TAAGA----- ---------- ---------- ----------
C.BR.92.BR025_d.U52953          ---------- ---------- ---------- ---------- ---GACTTGA AA-GCGAAAG TAAGA----- ---------- ---------- ----------
C.BR.98.98BR004.AF286228        ---------- ---------- ---------- ---------- ------TTGA AA-GTGAAAG TAAGA----- ---------- ---------- ----------
C.BW.00.00BW07621.AF443088      AG-TGGCGCC CGAACAGG-- ---------- ---------- ---GACTTGA AA-GTGAAAG TAAGA----- ---------- ---------- ----------
C.BW.00.00BW076820.AF443089     AG-TGGCGCC CGAACAGG-- ---------- ---------- ---GACTTGA AA-GCGGAAG TAAGA----- ---------- ---------- ----------
C.BW.00.00BW087421.AF443090     AG-TGGCGCC CGACCAGG-- ---------- ---------- ---GACTTGA AA-GCGAAAG TAAGA----- ---------- ---------- ----------
C.BW.00.00BW147127.AF443091     AG-TGGCGCC CGAACAGG-- ---------- ---------- ---GACGCGA AA-GCGAAAG TAGGA----- ---------- ---------- ----------
C.BW.00.00BW16162.AF443092      AG-TGGCGCC CGAACAGG-- ---------- ---------- ---GACCTGA AA-GCGAAAG TAAGA----- ---------- ---------- ----------
C.BW.00.00BW1686.AF443093       AG-TGGCGCC CGAACAGG-- ---------- ---------- ---GACGCGA AA-GCGAAAG TGAGA----- ---------- ---------- ----------
C.BW.00.00BW17593.AF443094      AG-TGGCGCC CGAACAGG-- ---------- ---------- ---GACTTGA AA-GCGAAAG TAAGA----- ---------- ---------- ----------
C.BW.00.00BW17732.AF443095      ---TGGCGCC CGAACAGG-- ---------- ---------- ---GACGCGA AA-GCGAAAG TAGAA----- ---------- ---------- ----------
C.BW.00.00BW17835.AF443096      AG-TGGCGCC CGAACAGG-- ---------- ---------- ---GACCCGA AA-GCGAAAG TAAGA----- ---------- ---------- ----------
C.BW.00.00BW17956.AF443097      AG-TGGCGCC CGAACAGG-- ---------- ---------- ---GACTTGA AA-GCGAAAG TAGAA----- ---------- ---------- ----------
C.BW.00.00BW18113.AF443098      AG-TGGCGCC CGAACGGG-- ---------- ---------- ---GACCTGA AA-GTGAAAG TAAGA----- ---------- ---------- ----------
C.BW.00.00BW18595.AF443099      AG-TGGCGCC CGAACAGG-- ---------- ---------- ---GGCTTGA AA-GCGAAAG TAAGA----- ---------- ---------- ----------
C.BW.00.00BW18802.AF443100      AG-TGGCGCC CGAACAGG-- ---------- ---------- ---GACTTGA AA-GCGAAAG TAAGA----- ---------- ---------- ----------
C.BW.00.00BW192113.AF443101     AG-TGGCGCC CGAACAGG-- ---------- ---------- ---GACTTGA AA-AGCGAAA GTAAA----- ---------- ---------- -----A----
C.BW.00.00BW20361.AF443102      AG-TGGCGCC CGAACAGG-- ---------- ---------- ---GACTTGA AA-GCGAAAG TAGAA----- ---------- ---------- ----------
C.BW.00.00BW20636.AF443103      AG-TGGCGCC CGAACAGG-- ---------- ---------- ---GACCCGA AG-GCGAAAG TAAGA----- ---------- ---------- ----------
C.BW.00.00BW20872.AF443104      AG-TGGCGCC CGAACAGG-- ---------- ---------- ---GACTTGA AA-GCGAAAG TAAGA----- ---------- ---------- ----------
C.BW.00.00BW2127214.AF443105    AG-TGGCGCC CGACCAGG-- ---------- ---------- ---GACCCGA AA-ACGAAAG TGAGA----- ---------- ---------- ----------
C.BW.00.00BW22767.AF443107      AG-TGGCGCC CGAACAGG-- ---------- ---------- ---GACTTGA AA-GCGAAAG CAAGA----- ---------- ---------- ----------
C.BW.00.00BW38193.AF443108      AG-TGGCGCC CGAACAGG-- ---------- ---------- ---GC-CTGA AA-GCGAAAG TAAGA----- ---------- ---------- ----------
C.BW.00.00BW38428.AF443109      AG-TGGCGCC CGAACAGG-- ---------- ---------- ---GACCTGA AA-GCAAAAG TGAAG----- ---------- ---------- ----------
C.BW.00.00BW38713.AF443110      AG-TGGCGCC CGAACAGG-- ---------- ---------- ---GACCTGA AA-GCGAAAG TAAGA----- ---------- ---------- ----------
C.BW.00.00BW3876_9.AF443111     AG-TGGCGCC CGAACAGG-- ---------- ---------- ---GACCTGA AA-GCGAAAG TAAGA----- ---------- ---------- ----------
C.BW.00.00BW3886_8.AF443112     AG-TGGCGCC CGAACAGG-- ---------- ---------- ---GACTTGA AA-GTGAAAG TAAGA----- ---------- ---------- ----------
C.BW.00.00BW3891_6.AF443113     AG-TGGCGCC CGAACAGG-- ---------- ---------- ---GACTTGA AA-GCGAAAG TAAGA----- ---------- ---------- ----------
C.BW.00.00BW3970_2.AF443114     AG-TGGCGCC CGAACAGG-- ---------- ---------- ---GACGCGA AA-GCGAAAG TAAGA----- ---------- ---------- ----------
C.BW.00.00BW5031_1.AF443115     G--TGGCGCC CGAACAGG-- ---------- ---------- ---GACTTGA AA-GCGAAAG TAAGA----- ---------- ---------- ----------
C.BW.96.96BW01B03.AF110959      AG-TGGCGCC CGAACAGG-- ---------- ---------- ---GACCTGA AA-GCGAAAG TAAGA----- ---------- ---------- ----------
C.BW.96.96BW0402.AF110962       AG-TGGCGCC CGAACAGG-- ---------- ---------- ---GACGCGA AA-GTGAAAG TAAGA----- ---------- ---------- ----------
C.BW.96.96BW0502.AF110967       AG-TGGCGCC CGAACAGG-- ---------- ---------- ---GACCAGA AA-GCGAAAG TAAAA----- ---------- ---------- ----------
C.BW.96.96BW06.AF290028         AG-TGGCGCC CGAACAGG-- ---------- ---------- ---GACTTGA AA-GCGAAAG TAAGA----- ---------- ---------- ----------
C.BW.96.96BW1104.AF110969       AG-TGGCGCC CGAACAGG-- ---------- ---------- ---GACCTGA AA-GCGAAAG TGAGA----- ---------- ---------- ----------
C.BW.96.96BW1210.AF110972       AG-TGGCGCC CGAACAGG-- ---------- ---------- ---GACCAGA AA-GCGAAAG TGAGA----- ---------- ---------- ----------
C.BW.96.96BW15C02.AF110974      AG-TGGCGCC CGAACAGG-- ---------- ---------- ---GACCTGA AA-GCGAAAG TAAGG----- ---------- ---------- ----------
C.BW.96.96BW16B01.AF110976      AG-TGGCGCC CGAACAGG-- ---------- ---------- ---GACGCGA AA-GCGAAAG TAGAA----- ---------- ---------- ----------
C.BW.96.96BW17.AF110980         AG-TGGCGCC CGAACAGG-- ---------- ---------- ---GACGCGA AA-GCGAAAG TAGGA----- ---------- ---------- ----------
C.BW.96.96BWM032.AF443075       AG-TGGCGCC CGAACAGG-- ---------- ---------- ---GACCTGA AA-ACGAAAG TGAGA----- ---------- ---------- ----------
C.BW.96.96BWMO1_5.AF443074      AG-TGGCGCC CGAACAGG-- ---------- ---------- ---GACCTGA AA-GCGAAAG TAGAA----- ---------- ---------- ----------
C.BW.98.98BWMC122.AF443076      AG-TGGCGCC CGAACAGG-- ---------- ---------- ---GACTTGA AA-GCGAAAG TAAGA----- ---------- ---------- ----------
C.BW.98.98BWMC134.AF443077      AG-TGGCGCC CGAACAGG-- ---------- ---------- ---GACCCGA AA-GCGAAAG TAGGA----- ---------- ---------- ----------
C.BW.98.98BWMC14A3.AF443078     AG-TGGCGCC CGAACAGG-- ---------- ---------- ---GACTTGA AG-GCGAAAG TAAGA----- ---------- ---------- ----------
C.BW.98.98BWMO1410.AF443079     AG-TGGCGCC CGAAAGGG-- ---------- ---------- ---GACTCGA AA-GCGAAAG TAAGA----- ---------- ---------- ----------
C.BW.98.98BWMO18D5.AF443080     AG-TGGCGCC CGAACAGG-- ---------- ---------- ---GACCTGA AA-GCGAAAG TAAGA----- ---------- ---------- ----------
C.BW.98.98BWMO36A5.AF443081     AG-TGGCGCC CGAACAGG-- ---------- ---------- ---GACTTGA AA-GCGAAAG TGAGA----- ---------- ---------- ----------
C.BW.98.98BWMO37D5.AF443082     AG-TGGCGCC CGAACAGG-- ---------- ---------- ---GACCTGA AA-GCGAAAG TAAGA----- ---------- ---------- ----------
C.BW.99.99BW393212.AF443083     AG-TGGCGCC CGAACAGG-- ---------- ---------- ---GACTTGA AA-ACGAAAG TAAGA----- ---------- ---------- ----------
C.BW.99.99BW46424.AF443084      AG-TGGCGCC CGAACAGG-- ---------- ---------- ---GACTTGA AA-GCGAAAG TAGAA----- ---------- ---------- ----------
C.BW.99.99BW4745.AF443085       AG-TGGCGCC CGAACAGG-- ---------- ---------- ---GACGCGA AA-GCGAAAG TAAGA----- ---------- ---------- ----------
C.BW.99.99BW47547.AF443086      AG-TGGCGCC CGAACAGG-- ---------- ---------- ---GACCCGA AA-GCGAAAG TAGGA----- ---------- ---------- ----------
C.BW.99.99BWMC168.AF443087      AG-TGGCGCC CGAACAGG-- ---------- ---------- ---GACCTGA AA-GCGAAAG TAAGG----- ---------- ---------- ----------
C.DJ.x.DJ259.AB485643           AG-TGGCGCC CGAACAGG-- ---------- ---------- ---GACCTGA AA-GCGAAAG TGAGA----- ---------- ---------- ----------
C.DK.01.CTL_015.EF514713        AG-TGGCGCC CGAACAGG-- ---------- ---------- ---GACTTGA AA-RCGAAAG TAAGA----- ---------- ---------- ----------
C.ES.06.X1936.EU786673          AG-TGGCGCC CGAACAGG-- ---------- ---------- ---GACTTGA AA-GCGAAAG TAAGA----- ---------- ---------- ----------
C.ES.07.X2118_2.EU884500        AG-TGGCGCC CGAACAGG-- ---------- ---------- ---GACTTGA AA-GCGAAAG TAAGA----- ---------- ---------- ----------
C.ES.08.X2363_2.EU786681        AG-TGGCGCC CGAACAGG-- ---------- ---------- ---GACGCGA AA-GCGAAAG TAGAA----- ---------- ---------- ----------
```

```
C.ET.86.ETH2220.U46016          AG-TGGCGCC CGAACAGG-- ---------- ---------- --GGACCTGA AA-GCGAAAG TGAGA----- ---------- ---------- ----------
C.IL.98.98IS002.AF286233        ---------- ---------- ---------- ---------- --------GA AA-ACGAAAG TGAGA----- ---------- ---------- ----------
C.IN.03.D24.EF469243            AG-TGGCGCC CGAACAGG-- ---------- ---------- ---GACGCGA AA-ACGAAAG TGAGA----- ---------- ---------- ----------
C.IN.93.93IN101.AB023804        AG-TGGCGCC CGAACAGG-- ---------- ---------- ---GACTTGA AA-GCGAAAG TAAGA----- ---------- ---------- ----------
C.IN.93.93IN904.AF067157        AG-TGGCGCC CGAACAGG-- ---------- ---------- ---GACTTGA AA-GCGAAAG TAAGA----- ---------- ---------- ----------
C.IN.93.93IN999.AF067154        AG-TGGCGCC CGAACAGG-- ---------- ---------- ---GACCTGA AA-GAGAAAG TAAGA----- ---------- ---------- ----------
C.IN.94.94IN11246.AF067159      AG-TGGCGCC CGAACAGG-- ---------- ---------- ---GACTTGA AA-GCGAAAG TAAGA----- ---------- ---------- ----------
C.IN.94.94IN476.AF286223        ---------- ---------- ---------- ---------- ------TTGA AA-GCGAAAG TAAGA----- ---------- ---------- ----------
C.IN.95.95IN21068.AF067155      AG-TGGCGCC CGAACAGG-- ---------- ---------- ---GACCTGA AA-GCGAAAG TAAGA----- ---------- ---------- ----------
C.IN.98.98IN012.AF286231        ---------- ---------- ---------- ---------- ------CTGA AA-GCGAAAG TAAGA----- ---------- ---------- ----------
C.IN.98.98IN022.AF286232        ---------- ---------- ---------- ---------- ------GCGA AA-GCGAAAG TAAGA----- ---------- ---------- ----------
C.IN.99.01IN565_10.AY049708     AG-TGGCGCC CGAACAGG-- ---------- ---------- ---GACCCGA AA-GCGAAAG TAAGA----- ---------- ---------- ----------
C.IN.x.VB39.EF694033            AG-TGGCGCC CGAACAGG-- ---------- ---------- ---GACTCGA AA-GCGAAAG TAAGA----- ---------- ---------- ----------
C.IN.x.VB49.EF694036            AG-TGGCGCC CGAACAGG-- ---------- ---------- ---GACCCGA AA-GCGAAAG TAAGA----- ---------- ---------- ----------
C.MM.99.mIDU101_3.AB097871      AG-TGGCGCC CGAACAGG-- ---------- ---------- ---GACCTGA AA-GCGAAAG TAAGA----- ---------- ---------- ----------
C.TZ.98.98TZ013.AF286234        ---------- ---------- ---------- ---------- -------CGA AA-GCGAAAG TAAGA----- ---------- ---------- ----------
C.TZ.98.98TZ017.AF286235        ---------- ---------- ---------- ---------- ------TTGA AA-GCGAAAG TAAGA----- ---------- ---------- ----------
C.ZA.00.1069MB.AY838567         AG-TGGCGCC CGAACAGG-- ---------- ---------- ---GACTTGA AA-GCGAAAG TAGGA----- ---------- ---------- ----------
C.ZA.00.1119MB.AY463229         AG-TGGCGCC CGAACAGG-- ---------- ---------- ---GACCCGA AA-GCGAAAG TGAGA----- ---------- ---------- ----------
C.ZA.00.1134MB.AY463217         AG-TGGCGCC CGAACAGG-- ---------- ---------- ---GACTTGA AA-GCGAAAG TAAGA----- ---------- ---------- ----------
C.ZA.00.1157M3M.AY585266        AG-TGGCGCC CGAACAGG-- ---------- ---------- ---GACCTGA AA-GCGAAAG TAAGA----- ---------- ---------- ----------
C.ZA.00.1162MB.AY463224         AG-TGGCGCC CGAACAGG-- ---------- ---------- ---GACCCGA AA-GTGAAAG TAAGA----- ---------- ---------- ----------
C.ZA.00.1165MB.AY463230         AG-TGGCGCC CGAACAGG-- ---------- ---------- ---GACGCGA AA-GCGAAAG TAAGA----- ---------- ---------- ----------
C.ZA.00.1168MB.AY463231         AG-TGGCGCC CGAACAGG-- ---------- ---------- ---GACTTGA AA-GCGAAAG TAAGA----- ---------- ---------- ----------
C.ZA.00.1170MB.AY463225         AG-TGGCGCC CGAACAGG-- ---------- ---------- ---GACTTGA AA-GCGAAAG TAAGA----- ---------- ---------- ----------
C.ZA.00.1171MB.AY463232         AG-TGGCGCC CGAACAGG-- ---------- ---------- ---GACTTGA AA-GCGAAAG TAAAA----- ---------- ---------- ----------
C.ZA.00.1176MB.AY463218         AG-TGGCGCC CGAACAGG-- ---------- ---------- ---GACGTGA AA-GCGAAAG TAAGA----- ---------- ---------- ----------
C.ZA.00.1178MB.AY463233         AG-TGGCGCC CGAACAGG-- ---------- ---------- ---GACGCGA AA-GTGAAAG TAAGA----- ---------- ---------- ----------
C.ZA.00.1184MB.AY838566         AG-TGGCGCC CGAACAGG-- ---------- ---------- ---GACTTGA AA-GCGAAAG TAAGA----- ---------- ---------- ----------
C.ZA.00.1189MB.AY838565         AG-TGGCGCC CGAACAGG-- ---------- ---------- ---GACTTGA AA-GCGAAAG TAAGA----- ---------- ---------- ----------
C.ZA.00.1192M3M.AY463219        AG-TGGCGCC CGAACAGG-- ---------- ---------- ---GACCTGA AA-GCGAAAG TAAGA----- ---------- ---------- ----------
C.ZA.00.1195MB.AY463220         AG-TGGCGCC CGAACAGG-- ---------- ---------- ---GACCTGA AA-GCGAAAG TAAGA----- ---------- ---------- ----------
C.ZA.00.1197MB.AY463234         AG-TGGCGCC CGAACAGG-- ---------- ---------- ---GACTTGA AA-ACGAAGG TAAGA----- ---------- ---------- ----------
C.ZA.00.1210MB.AY463221         AG-TGGCGCC CGAACAGG-- ---------- ---------- ---GACCTGA AA-GCGAAAG TAAGA----- ---------- ---------- ----------
C.ZA.00.1214MB.AY463236         AG-TGGCGCC CGAACAGG-- ---------- ---------- ---GACTTGA AA-GTGAAAG TAAGA----- ---------- ---------- ----------
C.ZA.00.1217MB.AY463226         AG-TGGCGCC CGAACAGG-- ---------- ---------- ---GACTTGA AA-GCGAAAG TAAGA----- ---------- ---------- ----------
C.ZA.00.1225MB.AY463227         AG-TGGCGCC CGAACAGG-- ---------- ---------- ---GACTTGA AA-ACGAAAG TAGAA----- ---------- ---------- ----------
C.ZA.00.1228MB.AY463222         AG-TGGCGCC CGAACAGG-- ---------- ---------- ---GACTTGA AA-GCGAAAG TAAGA----- ---------- ---------- ----------
C.ZA.00.J112MA.AY838568         AG-TGGCGCC CGAACAGG-- ---------- ---------- ---GACTTGA AA-GCGAAAG TGAGA----- ---------- ---------- ----------
C.ZA.00.J38MA.AY463228          AG-TGGCGCC CGAACAGG-- ---------- ---------- ---GACTTGA AA-GCGAAAG TAAGA----- ---------- ---------- ----------
C.ZA.01.01ZATM45.AY228557       AG-TGGCGCC CGAACAGG-- ---------- ---------- ---GACTTGA AA-GCGAAAG TAAGA----- ---------- ---------- ----------
C.ZA.01.2134MB.AY463237         AG-TGGCGCC CGAACAGG-- ---------- ---------- ---GACTTAA AA-GCGAAAG TAAGA----- ---------- ---------- ----------
C.ZA.01.J54Ma.AY463223          AG-TGGCGCC CGAACAGG-- ---------- ---------- ---GACCTGA AA-GCGAAAG TAAGA----- ---------- ---------- ----------
C.ZA.02.02ZAPS001MB1.DQ275648   AG-TGGCGCC CGAACAGG-- ---------- ---------- ---GACTTGA AA-GCGAAAG TAAGG----- ---------- ---------- ----------
C.ZA.02.02ZAPS005MB1.DQ351235   AG-TGGCGCC CGAACAGG-- ---------- ---------- ---GACTTGA AA-GCGAAAG CAGAA----- ---------- ---------- ----------
C.ZA.02.02ZAPS006MB1.DQ351220   AG-TGGCGCC CGAACAGG-- ---------- ---------- ---GACTTGA AA-GTGAAAG TAAGA----- ---------- ---------- ----------
C.ZA.02.02ZAPS008MB1.DQ275647   AG-TGGCGCC CGAACAGG-- ---------- ---------- ---GACCTGA AA-GCGAAAG TAAGA----- ---------- ---------- ----------
C.ZA.02.02ZAPS013MB1.DQ351222   AG-TGGCGCC CGAACAGG-- ---------- ---------- ---GACTTGA AA-GCGAAAG TAAGA----- ---------- ---------- ----------
C.ZA.02.02ZAPS014MB1.DQ351218   AG-TGGCGCC CGAACAGG-- ---------- ---------- ---GACTTGA AA-GCAAAAG TAAGA----- ---------- ---------- ----------
C.ZA.02.02ZAPS015MB1.DQ369995   AG-TGGCGCC CGAACAGG-- ---------- ---------- ---GACTTGA AA-GCGAAAG TAAGA----- ---------- ---------- ----------
C.ZA.03.03ZAPS017MB1.DQ351224   AG-TGGCGCC CGAACAGG-- ---------- ---------- ---GACCAGA AA-GTGAAAG TAAGA----- ---------- ---------- ----------
C.ZA.03.03ZAPS020MB1.DQ275653   AG-TGGCGCC CGAACAGG-- ---------- ---------- ---GACGCGA AA-GCGAAAG TGAGA----- ---------- ---------- ----------
C.ZA.03.03ZAPS021MB1.DQ369978   AG-TGGCGCC CGAACAGG-- ---------- ---------- ---GACCTGA AA-GCGAAAG TAAGA----- ---------- ---------- ----------
C.ZA.03.03ZAPS023MB1.DQ351225   AG-TGGCGCC CGAACAGG-- ---------- ---------- ---GACCCGA AA-GCGAAAG TAAGA----- ---------- ---------- ----------
C.ZA.03.03ZAPS024MB1.DQ396367   AG-TGGCGCC CGAACAGG-- ---------- ---------- ---GACCTGA AA-GCGAAAG TAAGA----- ---------- ---------- ----------
C.ZA.03.03ZAPS025MB1.DQ351226   AG-TGGCGCC CGAACAGG-- ---------- ---------- ---GACCTGA AA-GCGAAAG TAAGA----- ---------- ---------- ----------
C.ZA.03.03ZAPS026MB1.DQ369985   AG-TGGCGCC CGAACAGG-- ---------- ---------- ---GACCCGA AA-GCGAAAG TAAGA----- ---------- ---------- ----------
C.ZA.03.03ZAPS027MB1.DQ351223   AG-TGGCGCC CGAACAGG-- ---------- ---------- ---GACTTGA AA-ACGAAAG TAAGA----- ---------- ---------- ----------
C.ZA.03.03ZAPS030MB1.DQ369996   AG-TGGCGCC CGAACAGG-- ---------- ---------- ---GACTTGA AA-GCGAAAG TAGGA----- ---------- ---------- ----------
C.ZA.03.03ZAPS032MB1.DQ445633   AG-TGGCGCC CGAACAGG-- ---------- ---------- ---GACTTGA AA-GCGAAAG TGAGA----- ---------- ---------- ----------
C.ZA.03.03ZAPS034MB1.DQ369979   AG-TGGCGCC CGAACAGG-- ---------- ---------- ---GACCTGA AA-GCGAAAG TAAGA----- ---------- ---------- ----------
C.ZA.03.03ZAPS042MB1.DQ369977   AG-TGGCGCC CGAACAGG-- ---------- ---------- ---GACTTGA AA-GCGAAAG TAAGA----- ---------- ---------- ----------
C.ZA.03.03ZAPS043MB1.DQ351227   AG-TGGCGCC CGAACAGG-- ---------- ---------- ---GACTTGA AA-GCGAAAG TAAGA----- ---------- ---------- ----------
C.ZA.03.03ZAPS044MB1.DQ396384   AG-TGGCGCC CGAACAGG-- ---------- ---------- ---GACGCGA AA-GCGAAAG TAAGA----- ---------- ---------- ----------
C.ZA.03.03ZAPS046MB1.DQ369984   AG-TGGCGCC CGAACAGG-- ---------- ---------- ---GACCAGA AA-GCGAAAG TAAGA----- ---------- ---------- ----------
C.ZA.03.03ZAPS048MB1.DQ396364   AG-TGGCGCC CGAACAGG-- ---------- ---------- ---GACTTGA AA-GCGAAAG TAGAA----- ---------- ---------- ----------
C.ZA.03.03ZAPS049MB1.DQ369986   AG-TGGCGCC CGAACAGG-- ---------- ---------- ---GACTTGA AA-GCGAAAG TAAGA----- ---------- ---------- ----------
C.ZA.03.03ZAPS050MB1.DQ369980   AG-TGGCGCC CGAACAGG-- ---------- ---------- ---GACCCGA AA-GCGAAAG TAGGA----- ---------- ---------- ----------
C.ZA.03.03ZAPS051MB1.DQ396385   AG-TGGCGCC CGAACAGG-- ---------- ---------- ---GACTTGA AA-GCGAAAG TAAGA----- ---------- ---------- ----------
C.ZA.03.03ZAPS052MB1.DQ369987   AG-TGGCGCC CGAACAGG-- ---------- ---------- ---GACTTGA AA-GCGAAAG TAAGA----- ---------- ---------- ----------
C.ZA.03.03ZAPS054MB2.DQ369988   AG-TGGCGCC CGAACAGG-- ---------- ---------- ---GACTTGA AA-GCGAAAG TAAGA----- ---------- ---------- ----------
C.ZA.03.03ZAPS055MB1.DQ396373   AG-TGGCGCC CGAACAGG-- ---------- ---------- ---GACTTGA AA-GCGAAAG TAAGA----- ---------- ---------- ----------
C.ZA.03.03ZAPS056MB1.DQ396374   AG-TGGCGCC CGAACAGG-- ---------- ---------- ---GACTTGA AA-GCGAAAG TAAGA----- ---------- ---------- ----------
C.ZA.03.03ZAPS057MB2.DQ369989   AG-TGGCGCC CGAACAGG-- ---------- ---------- ---GACTTGA AA-GCGAAAG TAAGA----- ---------- ---------- ----------
C.ZA.03.03ZAPS059MB2.DQ445634   AG-TGGCGCC CGAACAGG-- ---------- ---------- ---GACTTGA AA-GCGAAAG TAAGA----- ---------- ---------- ----------
C.ZA.03.03ZAPS063MB1.DQ396388   AG-TGGCGCC CGAACAGG-- ---------- ---------- ---GACCCGA AA-GCGAAAG TAAGA----- ---------- ---------- ----------
C.ZA.03.03ZAPS066MB2.DQ396375   AG-TGGCGCC CGAACAGG-- ---------- ---------- ---GACTTGA AA-GCGAAAG TAAGA----- ---------- ---------- ----------
C.ZA.03.03ZAPS067MB2.DQ396389   AG-TGGCGCC CGAACAGG-- ---------- ---------- ---GACCCGA AA-GCGAAAG TACGA----- ---------- ---------- ----------
C.ZA.03.03ZAPS071MB1.DQ396376   AG-TGGCGCC CGAACAGG-- ---------- ---------- ---GACTTGA AA-GCGAAAG TAAGA----- ---------- ---------- ----------
C.ZA.03.03ZAPS073MB1.DQ275649   AG-TGGCGCC CGAACAGG-- ---------- ---------- ---GACCTGA AA-GCGAAAG TAGGA----- ---------- ---------- ----------
C.ZA.03.03ZAPS074MB2.DQ351228   AG-TGGCGCC CGAACAGG-- ---------- ---------- ---GACTTGA AA-GCGAAAG TAAGA----- ---------- ---------- ----------
C.ZA.03.03ZAPS077B1.DQ093591    AG-TGGCGCC CGAACAGG-- ---------- ---------- ---GACTTGA AA-GCGAAAG TAAGA----- ---------- ---------- ----------
C.ZA.03.03ZAPS079B1.DQ093592    AG-TGGCGCC CGAACAGG-- ---------- ---------- ---GACCCGA AA-GCGAAAG TAGGA----- ---------- ---------- ----------
C.ZA.03.03ZAPS081MB1.DQ351219   AG-TGGCGCC CGAACAGG-- ---------- ---------- ---GACTTGA AA-GCGAAAG TAAGA----- ---------- ---------- ----------
C.ZA.03.03ZAPS083MB1.DQ351229   AG-TGGCGCC CGAACAGG-- ---------- ---------- ---GACTTGA AA-GCGAAAG TAAGA----- ---------- ---------- ----------
C.ZA.03.03ZAPS086MB1.DQ275654   AG-TGGCGCC CGAACAGG-- ---------- ---------- ---GACCTGA AA-GCGAAAG TAAGA----- ---------- ---------- ----------
C.ZA.03.03ZAPS088MB1.DQ275651   AG-TGGCGCC CGAACAGG-- ---------- ---------- ---GACCCGA AA-GCGAAAG TAAGA----- ---------- ---------- ----------
C.ZA.03.03ZAPS089MB1.DQ351216   AG-TGGCGCC CGAACAGG-- ---------- ---------- ---GACTTGA AA-GCGAAAG TAGAA----- ---------- ---------- ----------
C.ZA.03.03ZAPS091MB1.DQ275645   AG-TGGCGCC CGAACAGG-- ---------- ---------- ---GACTTGA AA-ACGAAAG TAGAA----- ---------- ---------- ----------
C.ZA.03.03ZAPS094MB1.DQ396377   AG-TGGCGCC CGAACAGG-- ---------- ---------- ---GACCCGA AA-GCGAAAG TAAGA----- ---------- ---------- ----------
C.ZA.03.03ZAPS095MB1.DQ275652   AG-TGGCGCC CGAACAGG-- ---------- ---------- ---GACTTGA AA-GCGAAAG TAAGA----- ---------- ---------- ----------
C.ZA.03.03ZAPS097MB1.DQ351230   AG-TGGCGCC CGAACAGG-- ---------- ---------- ---GACCTAA AA-GCGAAAG TAAGA----- ---------- ---------- ----------
C.ZA.03.03ZAPS099MB1.DQ275655   AG-TGGCGCC CGAACAGG-- ---------- ---------- ---GACTTGA AA-GCGAAAG TAAGA----- ---------- ---------- ----------
C.ZA.03.03ZAPS103MB2.DQ275656   AG-TGGCGCC CGAACAGG-- ---------- ---------- ---GACCCGA AA-GCGAAAG TAAGA----- ---------- ---------- ----------
C.ZA.03.03ZAPS104MB1.DQ369990   AG-TGGCGCC CGAACAGG-- ---------- ---------- ---GACTTGA AA-GCGAAAG TAAGA----- ---------- ---------- ----------
C.ZA.03.03ZAPS105MB2.DQ445632   AG-TGGCGCC CGAACAGG-- ---------- ---------- ---GACTTGA AA-GCGAAAG TAAGA----- ---------- ---------- ----------
C.ZA.03.03ZAPS108MB1.DQ396378   AG-TGGCGCC CGAACAGG-- ---------- ---------- ---GACTTGA AA-GCGAAAG TAAAA----- ---------- ---------- ----------
C.ZA.03.03ZAPS112MB2.DQ396386   AG-TGGCGCC CGAACAGG-- ---------- ---------- ---GACCTGA AA-GCGAAAG TAGAA----- ---------- ---------- ----------
C.ZA.03.03ZAPS113MB2.DQ396365   AG-TGGCGCC CGAACAGG-- ---------- ---------- ---GACTTGA AA-GCGAAAG TAAGA----- ---------- ---------- ----------
C.ZA.03.03ZAPS116MB1.DQ445635   AG-TGGCGCC CGAACAGG-- ---------- ---------- ---GACTTGA AA-GCGAAAG TAAGA----- ---------- ---------- ----------
C.ZA.03.03ZAPS118MB1.DQ396368   AG-TGGCGCC CGAACAGG-- ---------- ---------- ---GACTTGA AA-GCGAAAG TAAGA----- ---------- ---------- ----------
C.ZA.03.03ZAPS122MB1.DQ396370   AG-TGGCGCC CGAACAGG-- ---------- ---------- ---GACTTGA AA-GCGAAAG TAAGG----- ---------- ---------- ----------
C.ZA.03.03ZAPS123MB1.DQ396369   AG-TGGCACC CGAACAGG-- ---------- ---------- ---GACCCGA AA-GCGAAAG TAAGA----- ---------- ---------- ----------
C.ZA.03.03ZAPS124MB1.DQ369976   AG-TGGCGCC CGAACAGG-- ---------- ---------- ---GACCTGA AA-GCGAAAG TAAGA----- ---------- ---------- ----------
C.ZA.03.03ZAPS125MB1.DQ396390   AG-TGGCGCC CGAACAGG-- ---------- ---------- ---GACTTGA AA-GCGAAAG TGAGA----- ---------- ---------- ----------
```

```
C.ZA.03.03ZAPS126MB1.DQ275657    AG-TGGCGCC CGAACAGG-- ---------- ---------- ---GACCTGA AA-GCGAAAG TAAGA----- ---------- ---------- ----------
C.ZA.03.03ZAPS128MB1.DQ275643    AG-TGGCGCC CGAACAGG-- ---------- ---------- ---GACTTGA AA-GCGAAAG TAAGA----- ---------- ---------- ----------
C.ZA.03.03ZAPS130MB1.DQ275658    AG-TGGCGCC CGAACAGG-- ---------- ---------- ---GACCCGA AA-GCGAAAG TAAGA----- ---------- ---------- ----------
C.ZA.03.03ZAPS131MB1.DQ396380    AG-TGGCGCC CGAACAGG-- ---------- ---------- ---GACCTGA AA-GCGAAAG TAAGA----- ---------- ---------- ----------
C.ZA.03.03ZAPS133MB1.DQ275646    AG-TGGCGCC CGAACAGG-- ---------- ---------- ---GGCTTGA AA-GCGAAAG TAAGA----- ---------- ---------- ----------
C.ZA.03.03ZAPS136MB1.DQ351231    AG-TGGCGCC CGAACAGG-- ---------- ---------- ---GACTTGA AA-GCGAAAG TAAGA----- ---------- ---------- ----------
C.ZA.03.03ZAPS140MB1.DQ369981    AG-TGGCGCC CGAACAGG-- ---------- ---------- ---GACGCGA AA-GCGAAAG TAAGA----- ---------- ---------- ----------
C.ZA.03.03ZAPS143MB1.DQ396391    AG-TGGCGCC CGAACAGG-- ---------- ---------- ---GACCTGA AA-ACGAAAG TAAGA----- ---------- ---------- ----------
C.ZA.03.03ZAPS151MB1.DQ396392    AG-TGGCGCC CGAACAGG-- ---------- ---------- ---GACTTGA AA-GCGAAAG TAAGA----- ---------- ---------- ----------
C.ZA.03.03ZAPS152MB1.DQ396399    AG-TGGCGCC TGAACAGG-- ---------- ---------- ---GACCCGA AA-GCGAAAG TAAGA----- ---------- ---------- ----------
C.ZA.03.03ZAPS155MB1.DQ396371    AG-TGGCGCC CGAACAGG-- ---------- ---------- ---GACTTGA AA-GCGAAAG TAAGA----- ---------- ---------- ----------
C.ZA.03.03ZASK005B2.DQ011175     AG-TGGCGCC CGAACAGG-- ---------- ---------- ---GACCAGA AA-GCGAAAG TAAGA----- ---------- ---------- ----------
C.ZA.03.03ZASK006B2.AY878056     AG-TGGCGCC CGAACAGG-- ---------- ---------- ---GACTTGA AA-GCGAAAG TAAGA----- ---------- ---------- ----------
C.ZA.03.03ZASK010B2.DQ164104     AG-TGGCGCC CGAACAGG-- ---------- ---------- ---GACGTGA AA-GCGAAAG TAAGA----- ---------- ---------- ----------
C.ZA.03.03ZASK011B2.AY901965     AG-TGGCGCC CGAACAGG-- ---------- ---------- ---GACTTGA AA-GCGAAAG TAAGA----- ---------- ---------- ----------
C.ZA.03.03ZASK013B2.DQ275660     AG-TGGCGCC CGAACAGG-- ---------- ---------- ---GACCTGA AA-GCGAAAG TAAGA----- ---------- ---------- ----------
C.ZA.03.03ZASK016MB2.DQ351233    AG-TGGCGCC CGAGCAGG-- ---------- ---------- ---GACTTGA AA-ACGAAAG TAAGA----- ---------- ---------- ----------
C.ZA.03.03ZASK019B2.AY878063     AG-TGGCGCC CGAACAGG-- ---------- ---------- ---GACACGA AA-GCGAAAG TAAGA----- ---------- ---------- ----------
C.ZA.03.03ZASK020B2.AY878064     AG-TGGCGCC CGAACAGG-- ---------- ---------- ---GACCTGA AA-GCGAAAG TAAGA----- ---------- ---------- ----------
C.ZA.03.03ZASK026B2.DQ011165     AG-TGGCGCC CGAACAGG-- ---------- ---------- ---GACCTGA AA-GCGAAAG TAAGA----- ---------- ---------- ----------
C.ZA.03.03ZASK034B1.AY878065     AG-TGGCGCC CGAACAGG-- ---------- ---------- ---GACCCGA AA-GCGAAAG TAAGG----- ---------- ---------- ----------
C.ZA.03.03ZASK036B1.AY901966     AG-TGGCGCC CGAACAGG-- ---------- ---------- ---GACTTGA AA-GCGAAAG TAAGA----- ---------- ---------- ----------
C.ZA.03.03ZASK039B2.AY878068     AG-TGGCGCC CGAACAGG-- ---------- ---------- ---GACTTGA AA-GCGAAAG TGAGA----- ---------- ---------- ----------
C.ZA.03.03ZASK058B2.AY901967     AG-TGGCGCC CGAACAGG-- ---------- ---------- ---GACGCGA AA-GCGAAAG TAAGA----- ---------- ---------- ----------
C.ZA.03.03ZASK061B1.AY901968     AG-TGGCGCC CGAACAGG-- ---------- ---------- ---GACTCGA AA-GCGAAAG TAGGA----- ---------- ---------- ----------
C.ZA.03.03ZASK062B1.DQ164113     AG-TGGCGCC CGAACAGG-- ---------- ---------- ---GACGCGA AA-GCGAAAG TAAGA----- ---------- ---------- ----------
C.ZA.03.03ZASK066B1.AY901969     AG-TGGCGCC CGAACAGG-- ---------- ---------- ---GACGCGA AA-GCGAAAG TAAGA----- ---------- ---------- ----------
C.ZA.03.03ZASK067B1.DQ275642     AG-TGGCGCC CGAACAGG-- ---------- ---------- ---GACCCGA AA-GCGAAAG TAAGA----- ---------- ---------- ----------
C.ZA.03.03ZASK072B1.DQ093593     AG-TGGCGCC CGAACAGG-- ---------- ---------- ---GACTTGA AA-GTGAAGG TAAGA----- ---------- ---------- ----------
C.ZA.03.03ZASK073B1.AY901970     AG-TGGCGCC CGAACAGG-- ---------- ---------- ---GACTTGA AA-GCGAAAG TAAGA----- ---------- ---------- ----------
C.ZA.03.03ZASK076B1.AY901975     AG-TGGCGCC CGAACAGG-- ---------- ---------- ---GACCCGA AA-GCGAAAG TAGAA----- ---------- ---------- ----------
C.ZA.03.03ZASK078B1.AY901971     AG-TGGCGCC CGAACAGG-- ---------- ---------- ---GACCCGA AA-GCGAAAG TAAGA----- ---------- ---------- ----------
C.ZA.03.03ZASK084B1.AY901981     AG-TGGCGCC CGAACAGG-- ---------- ---------- ---GACCCGA AA-GCGAAAG TAAGA----- ---------- ---------- ----------
C.ZA.03.03ZASK092B1.AY878057     AG-TGGCGCC CGAACAGG-- ---------- ---------- ---GACTTGA AA-GCGAAAG TAAGA----- ---------- ---------- ----------
C.ZA.03.03ZASK094B1.AY878070     AG-TGGCGCC CGAACAGG-- ---------- ---------- ---GACTTGA AA-GCGAAAG TAAGA----- ---------- ---------- ----------
C.ZA.03.03ZASK097B1.AY878060     AG-TGGCGCC CGAACAGG-- ---------- ---------- ---GACCCGA AA-GCGAAAG TAAGA----- ---------- ---------- ----------
C.ZA.03.03ZASK098B1.AY878061     AG-TGGCGCC CGAACAGG-- ---------- ---------- ---GACTTGA AA-GTGAAAG TAGGA----- ---------- ---------- ----------
C.ZA.03.03ZASK103B1.DQ164106     AG-TGGCGCC CGAACAGG-- ---------- ---------- ---GACTTGA AA-GCGAAAG TAAGA----- ---------- ---------- ----------
C.ZA.03.03ZASK104B1.DQ396395     AG-TGGCGCC CGAACAGG-- ---------- ---------- ---GACCTGA AA-GCGAAAG TAAGA----- ---------- ---------- ----------
C.ZA.03.03ZASK107B1.DQ056410     AG-TGGCGCC CGAACAGG-- ---------- ---------- ---GACGCGA AA-GCGAAAG TAAGA----- ---------- ---------- ----------
C.ZA.03.03ZASK110B1.DQ056411     AG-TGGCGCC CGAACAGG-- ---------- ---------- ---GACTTGA AA-GCGAAAG TAGGA----- ---------- ---------- ----------
C.ZA.03.03ZASK111B1.DQ056404     AG-TGGCGCC CGAACAGG-- ---------- ---------- ---GACTTGA AA-GCGAAAG TAAGA----- ---------- ---------- ----------
C.ZA.03.03ZASK113B1.DQ351237     AG-TGGCGCC CGAACAGG-- ---------- ---------- ---GACTTGA AA-GCGAAAG TAGAA----- ---------- ---------- ----------
C.ZA.03.03ZASK117B1.DQ056408     AG-TGGCGCC CGAACAGG-- ---------- ---------- ---GACCTGA AA-GCGAAAG TAAGA----- ---------- ---------- ----------
C.ZA.03.03ZASK118B1.DQ011169     AG-TGGCGCC CGAACAGG-- ---------- ---------- ---GACCCGA AA-GCGAAAG TGAGA----- ---------- ---------- ----------
C.ZA.03.03ZASK120B1.DQ011176     AG-TGGCGCC CGAACAGG-- ---------- ---------- ---GACTCGA AA-GCGAAAG TAAGA----- ---------- ---------- ----------
C.ZA.03.03ZASK211B1.DQ093601     AG-TGGCGCC CGAACAGG-- ---------- ---------- ---GACTTGA AA-ACGAAAG TAGAA----- ---------- ---------- ----------
C.ZA.03.03ZASK212B.DQ978981      AG-TGGCGCC CGAACAGG-- ---------- ---------- ---GACCCGA AA-GCGAAAG TAAGA----- ---------- ---------- ----------
C.ZA.03.03ZASK213B1.DQ093607     AG-TGGCGCC CGAACAGG-- ---------- ---------- ---GACCTGA AA-GCGAAAG TGAGA----- ---------- ---------- ----------
C.ZA.03.03ZASK215M6W.DQ275661    AG-TGGCGCC CGAACAGG-- ---------- ---------- ---GACCCGA AA-GCGAAAG TAAGA----- ---------- ---------- ----------
C.ZA.03.03ZASK223B1.DQ093597     AG-TGGCGCC CGAACAGG-- ---------- ---------- ---GACCCGA AA-GCGAAAG TAAGA----- ---------- ---------- ----------
C.ZA.03.03ZASK224MB1.DQ275664    AG-TGGCGCC CGAACAGG-- ---------- ---------- ---GACCAGA AA-GCGAAAG CAAGA----- ---------- ---------- ----------
C.ZA.03.03ZASK226B1.DQ164108     AG-TGGCGCC CGAACAGG-- ---------- ---------- ---GACTTGA AA-GTGAAAG TAAGA----- ---------- ---------- ----------
C.ZA.03.03ZASK232B1.DQ093589     AG-TGGCGCC CGAACAGG-- ---------- ---------- ---GACTCGA AA-GCGAAAG TAAGA----- ---------- ---------- ----------
C.ZA.03.03ZASK233B1.DQ351234     AG-TGGCGCC CGAACAGG-- ---------- ---------- ---GACGCGA AA-GCGAAAG TAAGA----- ---------- ---------- ----------
C.ZA.03.04ZAPS194MB1.DQ275650    AG-TGGCGCC CGAACAGG-- ---------- ---------- ---GACCTGA AA-GTGAAAG TAGGA----- ---------- ---------- ----------
C.ZA.03.04ZASK082B1.AY901972     AG-TGGCGCC CGAACAGG-- ---------- ---------- ---GACGCGA AA-GCGAAGG TAAAA----- ---------- ---------- ----------
C.ZA.03.SK023B2.AY772690         AG-TGGCGCC CGAACAGG-- ---------- ---------- ---GGCTTGA AA-GCGAAAG TAAGA----- ---------- ---------- ----------
C.ZA.03.SK029B2.AY772691         AG-TGGCGCC CGAACAGG-- ---------- ---------- ---GACCCGA AA-GCGAAAG TAAGA----- ---------- ---------- ----------
C.ZA.03.SK033B2.AY772692         AG-TGGCGCC CGAACAGG-- ---------- ---------- ---GACCTGA AA-GCGAAAG TGAGA----- ---------- ---------- ----------
C.ZA.03.SK040B1.AY703908         AG-TGGCGCC CGAACAGG-- ---------- ---------- ---GACCCGA AA-GCGAAAG TAGGA----- ---------- ---------- ----------
C.ZA.03.SK041B1.AY772693         AG-TGGCGCC CGAACAGG-- ---------- ---------- ---GACTTGA AA-GCGAAAG TAAGA----- ---------- ---------- ----------
C.ZA.03.SK043B1.AY772700         AG-TGGCGCC CGAACAGG-- ---------- ---------- ---GACTCGA AA-GCGAAAG TAAGA----- ---------- ---------- ----------
C.ZA.03.SK065B1.AY772694         AG-TGGCGCC CGAACAGG-- ---------- ---------- ---GACCCGA AA-GCGAAAG TAGGA----- ---------- ---------- ----------
C.ZA.03.SK112B1.AY772695         AG-TGGCGCC CGAACAGG-- ---------- ---------- ---GACGCGA AA-GCGAAAG TAAGA----- ---------- ---------- ----------
C.ZA.03.SK116B1.AY772696         AG-TGGCGCC CGAACAGG-- ---------- ---------- ---GACCTGA AA-GCGAAAG TAAGA----- ---------- ---------- ----------
C.ZA.04.04ZAPS157MB1.DQ351232    AG-TGGTGCC CGAACAGG-- ---------- ---------- ---GACTTGA AA-GCGAAAG TAAAA----- ---------- ---------- ----------
C.ZA.04.04ZAPS160B1.DQ164107     AG-TGGCGCC CGAACAGG-- ---------- ---------- ---GACTTGA AA-GTGAAAG TAAGA----- ---------- ---------- ----------
C.ZA.04.04ZAPS161B1.DQ164115     AG-TGGCGCC CGAACAGG-- ---------- ---------- ---GACGCGA AA-GCGAAAG TAAGA----- ---------- ---------- ----------
C.ZA.04.04ZAPS165MB1.DQ351221    AG-TGGCGCC CGAACAGG-- ---------- ---------- ---GACTTGA AA-GCGAAAG TAAGA----- ---------- ---------- ----------
C.ZA.04.04ZAPS168B1.DQ164121     AG-TGGCGCC CGAACAGG-- ---------- ---------- ---GACTTGA AA-ACGAAAG TGAGA----- ---------- ---------- ----------
C.ZA.04.04ZAPS169MB1.DQ396393    AG-TGGCGCC CGAACAGG-- ---------- ---------- ---GACCTGA AA-GCGAAAG TGAGA----- ---------- ---------- ----------
C.ZA.04.04ZAPS172MB1.DQ275659    AG-TGGCGCC CGAACAGG-- ---------- ---------- ---GACCCGA AA-GCGAAAG TAAGA----- ---------- ---------- ----------
C.ZA.04.04ZAPS177MB1.DQ396394    AG-TGGCGCC CGAACAGG-- ---------- ---------- ---GACCTGA AA-GCGAAAG TAAGA----- ---------- ---------- ----------
C.ZA.04.04ZAPS188B1.DQ164122     AG-TGGCGCC CGAACAGG-- ---------- ---------- ---GACCTGA AA-GCGAAAG TAAGA----- ---------- ---------- ----------
C.ZA.04.04ZAPS189B1.DQ164117     AG-TGGCGCC CGAACAGG-- ---------- ---------- ---GACGCGA AA-GCGAAAG TAAGA----- ---------- ---------- ----------
C.ZA.04.04ZAPS190B1.DQ093602     AG-TGGCGCC CGAACAGG-- ---------- ---------- ---GACTTGA AA-GCGAAAG TAAGA----- ---------- ---------- ----------
C.ZA.04.04ZAPS195B1.DQ164118     AG-TGGCGCC CGAACAGG-- ---------- ---------- ---GACTTGA AA-GCGAAAG TAAGA----- ---------- ---------- ----------
C.ZA.04.04ZAPS197MB1.DQ369997    AG-TGGCGCC CGAACAGG-- ---------- ---------- ---GACTTGA AA-GCGAAAG TAAGA----- ---------- ---------- ----------
C.ZA.04.04ZAPS198MB1.DQ445637    AG-TGGCGCC CGAACAGG-- ---------- ---------- ---GACGCGA AA-GCGAAAG TAAGA----- ---------- ---------- ----------
C.ZA.04.04ZAPS199B1.DQ164110     AG-TGGCGCC CGAACAGG-- ---------- ---------- ---GACTTGA AA-GCGAAAG TAAGA----- ---------- ---------- ----------
C.ZA.04.04ZAPS202B1.DQ093598     AG-TGGCGCC CGAACAGG-- ---------- ---------- ---GACTTGA AA-GCGAAAG TAAGA----- ---------- ---------- ----------
C.ZA.04.04ZAPS205B1.DQ093599     AG-TGGCGCC CGAACAGG-- ---------- ---------- ---GACTTGA AA-TCGAAAG TAAGA----- ---------- ---------- ----------
C.ZA.04.04ZAPS206B1.DQ164126     AG-TGGCGCC CGAACAGG-- ---------- ---------- ---GACCAGA AA-GCGAAAG TAAGA----- ---------- ---------- ----------
C.ZA.04.04ZAPS208B1.DQ093603     AG-TGGCGCC CGAACAGG-- ---------- ---------- ---GACTTGA AA-GCGAAAG TAAGA----- ---------- ---------- ----------
C.ZA.04.04ZAPS214B1.DQ093588     AG-TGGCGCC CGAACAGG-- ---------- ---------- ---GACCCGA AA-GCGAAAG TAAGA----- ---------- ---------- ----------
C.ZA.04.04ZAPS216B1.DQ093600     AG-TGGCGCC CGAACAGG-- ---------- ---------- ---GACTTGA AA-GCGAAAG TAAGA----- ---------- ---------- ----------
C.ZA.04.04ZAPS217B1.DQ164119     AG-TGGCGCC CGAACAGG-- ---------- ---------- ---GACCTGA AA-GCGAAAG TGAGA----- ---------- ---------- ----------
C.ZA.04.04ZASK031B2.AY901974     AG-TGGCGCC CGAACAGG-- ---------- ---------- ---GACCTGA AA-GCGAAAG TAGAA----- ---------- ---------- ----------
C.ZA.04.04ZASK083B2.DQ093585     AG-TGGCGCC CGAACAGG-- ---------- ---------- ---GACTTGA AA-GCGAAAG TGAGA----- ---------- ---------- ----------
C.ZA.04.04ZASK127B1.DQ011172     AG-TGGCGCC CGAACAGG-- ---------- ---------- ---GACTTGA AA-GCGAAAG TAAGA----- ---------- ---------- ----------
C.ZA.04.04ZASK128B1.DQ093586     AG-TGGCGCC CGAACAGG-- ---------- ---------- ---GACCCGA AA-GCGAAAG TAAGA----- ---------- ---------- ----------
C.ZA.04.04ZASK131B1.DQ056412     AG-TGGCGCC CGAACAGG-- ---------- ---------- ---GACGCGA AA-GCGAAAG TAAGG----- ---------- ---------- ----------
C.ZA.04.04ZASK132B1.DQ093594     AG-TGGCGCC CGAACAGG-- ---------- ---------- ---GACCCGA AA-GCGAAAG TAAGA----- ---------- ---------- ----------
C.ZA.04.04ZASK135B1.DQ011166     AG-TGGCGCC CGAACAGG-- ---------- ---------- ---GACCCGA AA-GCGAAAG TAAGA----- ---------- ---------- ----------
C.ZA.04.04ZASK136B1.DQ011177     AG-TGGCGCC CGAACAGG-- ---------- ---------- ---GACCCGA AA-GCGAAAG TAGGA----- ---------- ---------- ----------
C.ZA.04.04ZASK139B1.AY878072     AG-TGGCGCC CGAACAGG-- ---------- ---------- ---GACCTGA AA-GCGAAAG TAAGA----- ---------- ---------- ----------
C.ZA.04.04ZASK142B1.DQ011167     AG-TGGCGCC CGAACAGG-- ---------- ---------- ---GACTAGA AA-GCGAAAG TCAGA----- ---------- ---------- ----------
C.ZA.04.04ZASK145B1.AY901976     AG-TGGCGCC CGAACAGG-- ---------- ---------- ---GACCTGA AA-GCGAAAG CAAGA----- ---------- ---------- ----------
C.ZA.04.04ZASK146.AY772699       AG-TGGCGCC CGAACAGG-- ---------- ---------- ---GACCGGA AA-GCGAAAG TAAAA----- ---------- ---------- ----------
C.ZA.04.04ZASK147B1.DQ164114     AG-TGGCGCC CGAACAGG-- ---------- ---------- ---GACTTGA AA-GCGAAAG TAAGA----- ---------- ---------- ----------
C.ZA.04.04ZASK148B1.DQ093590     AG-TGGCGCC CGAACAGG-- ---------- ---------- ---GACTTGA AA-GCGAAAG TAAGA----- ---------- ---------- ----------
C.ZA.04.04ZASK150B1.DQ011178     AG-TGGCGCC CGAACAGG-- ---------- ---------- ---GACCTGA AA-GCGAAAG TAAGA----- ---------- ---------- ----------
C.ZA.04.04ZASK151B1.AY901977     AG-TGGCGCC CGAACAGG-- ---------- ---------- ---GACTTGA AA-ACGAAAG TAAGA----- ---------- ---------- ----------
C.ZA.04.04ZASK154B1.AY878071     AG-TGGCGCC CGAACAGG-- ---------- ---------- ---GACCTGA AA-GCGAAAG TGAGA----- ---------- ---------- ----------
```

```
C.ZA.04.04ZASK155B1.AY901978     AG-TGGCGCC CGAACAGG-- ---------- ---------- ---GACTTGA AA-GCGAAAG TAAGA----- ---------- ---------- ----------
C.ZA.04.04ZASK156B1.DQ011171     AG-TGGCGCC CGAACAGG-- ---------- ---------- ---GACCTGA AA-GCGAAAG TAAGA----- ---------- ---------- ----------
C.ZA.04.04ZASK159B1.DQ011179     AG-TGGCGCC CGAACAGG-- ---------- ---------- ---GACCCGA AA-GCGAAAG TAAGA----- ---------- ---------- ----------
C.ZA.04.04ZASK160B1.DQ011173     AG-TGGCGCC CGAACAGG-- ---------- ---------- ---GACCCGA AA-GCGAAAG TAAAA----- ---------- ---------- ----------
C.ZA.04.04ZASK161B1.DQ011170     AG-TGGCGCC CGAACAGG-- ---------- ---------- ---GACTTGA AA-GCGAAAG TAAGA----- ---------- ---------- ----------
C.ZA.04.04ZASK163B1.AY901979     AG-TGGCGCC CGAACAGG-- ---------- ---------- ---GACTTGA AA-GCGAAAG TAAGA----- ---------- ---------- ----------
C.ZA.04.04ZASK164B1.DQ056405     AG-TGGCGCC CGAACAGG-- ---------- ---------- ---GACGCGA AA-GCGAAAG TGAGA----- ---------- ---------- ----------
C.ZA.04.04ZASK165B1.DQ396387     AG-TGGCGCC CGAACAGG-- ---------- ---------- ---GACTTGA AA-GCGAAAG TAGGA----- ---------- ---------- ----------
C.ZA.04.04ZASK167B1.DQ164127     AG-TGGCGCC CGAACAGG-- ---------- ---------- ---GACCCGA AA-GCGAAAG TAAGA----- ---------- ---------- ----------
C.ZA.04.04ZASK168B1.AY878058     AG-TGGCGCC CGAACAGG-- ---------- ---------- ---GACTTGA AA-GCGAAAG TAAAA----- ---------- ---------- ----------
C.ZA.04.04ZASK169B1.DQ396381     AG-TGGCGCC CGAACAGG-- ---------- ---------- ---GACTTGA AA-GCGAAAG TAAGA----- ---------- ---------- ----------
C.ZA.04.04ZASK170B1.DQ093595     AG-TGGCGCC CGAACAGG-- ---------- ---------- ---GACGCGA AA-GCGAAAG TAAGA----- ---------- ---------- ----------
C.ZA.04.04ZASK171B1.DQ351217     AG-TGGCGCC CGAACAGG-- ---------- ---------- ---GACTTGA AA-GCGAAAG TAAGA----- ---------- ---------- ----------
C.ZA.04.04ZASK172B1.DQ369998     AG-TGGCGCC CGAACAGG-- ---------- ---------- ---GACCCGA AA-GCGAAAG TAGGA----- ---------- ---------- ----------
C.ZA.04.04ZASK173B1.DQ093604     AG-TGGCGCC CGAACAGG-- ---------- ---------- ---GACCTGA AA-GCGAAAG TAAGA----- ---------- ---------- ----------
C.ZA.04.04ZASK174B1.AY901980     AG-TGGCGCC CGAACAGG-- ---------- ---------- ---GACCTGA AA-GCGAAAG TAGAA----- ---------- ---------- ----------
C.ZA.04.04ZASK175B1.DQ164129     AG-TGGAAAA TCTCTAGCAG TGGCGCCCGA AC-------- AGGGACTTGA AA-GCGAAAG TAAGA----- ---------- ---------- ----------
C.ZA.04.04ZASK176B1.DQ056416     AG-TGGCGCC CGACCAGG-- ---------- ---------- ---GACTTGA AA-ACGAAAG TAAGA----- ---------- ---------- ----------
C.ZA.04.04ZASK178B1.DQ093587     AG-TGGCGCC CGAACAGG-- ---------- ---------- ---GACCTGA AA-GCGAAAG TAAGA----- ---------- ---------- ----------
C.ZA.04.04ZASK180B1.AY878059     AG-TGGCGCC CGAACAGG-- ---------- ---------- ---GACTTGA AA-GCGAAAG TAAGA----- ---------- ---------- ----------
C.ZA.04.04ZASK181B1.AY878062     AG-TGGCGCC CGAACAGG-- ---------- ---------- ---GACTTGA AA-GCGAAAG TAAGA----- ---------- ---------- ----------
C.ZA.04.04ZASK182B1.AY878054     AG-TGGCGCC CGAACAGG-- ---------- ---------- ---GACTTGA AA-GCGAAAG TAAGA----- ---------- ---------- ----------
C.ZA.04.04ZASK183B1.AY878055     AG-TGGCGCC CGAACAGG-- ---------- ---------- ---GACTTGA AA-GTGAAAG TAAGA----- ---------- ---------- ----------
C.ZA.04.04ZASK184B1.DQ056418     AG-TGGCGCC CGAACAGG-- ---------- ---------- ---GACGCGA AA-GCGAAAG TAAGA----- ---------- ---------- ----------
C.ZA.04.04ZASK185B1.DQ011174     AG-TGGCGCC CGAACAGG-- ---------- ---------- ---GACCTGA AA-GCGAAAG TAAGA----- ---------- ---------- ----------
C.ZA.04.04ZASK190B1.DQ056409     AG-TGGCGCC CGAACAGG-- ---------- ---------- ---GACTTGA AA-GCGAAAG TAAGA----- ---------- ---------- ----------
C.ZA.04.04ZASK191B1.DQ369993     AG-TGGCGCC CGAACAGG-- ---------- ---------- ---GACGCGA AA-GCGAAAG TAAGA----- ---------- ---------- ----------
C.ZA.04.04ZASK192B1.DQ396382     AG-TGGCGCC CGAACAGG-- ---------- ---------- ---GACCCGA AA-GCGAAAG TGAGA----- ---------- ---------- ----------
C.ZA.04.04ZASK193B1.DQ396396     AG-TGGCGCC CGAACAGG-- ---------- ---------- ---GACCTGA AA-ACGAAAG TAAGA----- ---------- ---------- ----------
C.ZA.04.04ZASK196B1.DQ056413     AG-TGGCGCC CGAACAGG-- ---------- ---------- ---GACTTGA AA-GCGAAAG TAAGA----- ---------- ---------- ----------
C.ZA.04.04ZASK200B1.DQ396383     AG-TGGCGCC CGAACAGG-- ---------- ---------- ---GACTTGA AA-GCGAAAG TAAGA----- ---------- ---------- ----------
C.ZA.04.04ZASK201B1.DQ396397     AG-TGGCGCC CGAACAGG-- ---------- ---------- ---GACCCGA AA-GCGAAAG TAAGA----- ---------- ---------- ----------
C.ZA.04.04ZASK202B1.DQ011180     AG-TGGCGCC CGAACAGG-- ---------- ---------- ---GACCTGA AA-GTGAAAG TAAGA----- ---------- ---------- ----------
C.ZA.04.04ZASK204B1.DQ056414     AG-TGGCGCC CGAACAGG-- ---------- ---------- ---GACTTGA AA-GCGAAAG TAAGA----- ---------- ---------- ----------
C.ZA.04.04ZASK206B1.DQ056415     AG-TGGCGCC CGAACAGG-- ---------- ---------- ---GACTTGA AA-GCGAAAG TAAAA----- ---------- ---------- ----------
C.ZA.04.04ZASK208B1.DQ056406     AG-TGGCGCC CGAACAGG-- ---------- ---------- ---GACTTGA AA-GCGAAAG TGAGA----- ---------- ---------- ----------
C.ZA.04.04ZASK217B1.DQ056417     AG-TGGCGCC CGAACAGG-- ---------- ---------- ---GACGCGA AA-GCGAAAG CAAGA----- ---------- ---------- ----------
C.ZA.04.04ZASK234B1.DQ093605     AG-TGGCGCC CGAACAGG-- ---------- ---------- ---GACCCGA AA-GCGAAAG TAGGA----- ---------- ---------- ----------
C.ZA.04.CAP30_5w_F4.GQ999973     AG-TGGCGCC CGAACAGG-- ---------- ---------- ---GACTTGA AA-GCGAAAG TAAGA----- ---------- ---------- ----------
C.ZA.04.CAP61_8w_F3.GQ999975     AG-TGGCGCC CGAACAGG-- ---------- ---------- ---GACTTGA AA-GCGAAAG TAAGA----- ---------- ---------- ----------
C.ZA.04.SK133B1.AY772698         AG-TGGCGCC CGAACAGG-- ---------- ---------- ---GACTTGA AA-GCGAAAG TAAGA----- ---------- ---------- ----------
C.ZA.04.SK134B1.AY703909         AG-TGGCGCC CGAACAGG-- ---------- ---------- ---GACGCGA AA-GCGAAAG TAAGA----- ---------- ---------- ----------
C.ZA.04.SK140B1.AY901973         AG-TGGCGCC CGAACAGG-- ---------- ---------- ---GACGCGA AA-ACGAAAG TGAGA----- ---------- ---------- ----------
C.ZA.04.SK143B1.AY703910         AG-TGGCGCC CGAACAGG-- ---------- ---------- ---GACCTGA AA-GCGAAAG TAAGA----- ---------- ---------- ----------
C.ZA.04.SK144B1.AY703911         AG-TGGCGCC CGAACAGG-- ---------- ---------- ---GACTTGA AA-GCGAAAG TAAGG----- ---------- ---------- ----------
C.ZA.05.05ZAPSK240B1.DQ369991    AG-TGGCGCC CGAACAGG-- ---------- ---------- ---GACCCGA AA-GCGAAAG TAAGA----- ---------- ---------- ----------
C.ZA.05.05ZASK243B1.DQ396372     AG-TGGCGCC CGAACAGG-- ---------- ---------- ---GACTTGA AA-GCGAAAG TAAGA----- ---------- ---------- ----------
C.ZA.05.05ZASK244B1.DQ369992     AG-TGGCGCC CGAACAGG-- ---------- ---------- ---GACTTGA AA-GCGAAAG TAAGA----- ---------- ---------- ----------
C.ZA.05.05ZASK245B1.DQ369982     AG-TGGCGCC CGAACAGG-- ---------- ---------- ---GACTTGA AA-GCGAAAG TAAGA----- ---------- ---------- ----------
C.ZA.05.05ZASK246B1.DQ369983     AG-TGGCGCC CGAACAGG-- ---------- ---------- ---GACTTGA AA-GCGAAAG TCAGA----- ---------- ---------- ----------
C.ZA.05.05ZASK247B1.DQ369994     AG-TGGCGCC TGAACAGG-- ---------- ---------- ---GACTCGA AA-GCGAAAG TAGAA----- ---------- ---------- ----------
C.ZA.05.CAP174_4w.GQ999981       AG-TGGCGCC CGAACAGG-- ---------- ---------- ---GACCCGA AA-GCGAAAG TAAGA----- ---------- ---------- ----------
C.ZA.05.CAP206_8w_F1.GQ999982    AG-TGGCGCC CGAACAGA-- ---------- ---------- ---GACCTGA AA-GCGAAAG TAAGA----- ---------- ---------- ----------
C.ZA.05.CAP210_5w.GQ999983       AG-TGGCGCC CGAACAGG-- ---------- ---------- ---GACTTGA AA-GCGAAAG TAAGA----- ---------- ---------- ----------
C.ZA.05.CAP228_8w_F2.GQ999984    AG-TGGCGCC CGAACAGG-- ---------- ---------- ---GACCCGA AA-GCGAAAG TAAGA----- ---------- ---------- ----------
C.ZA.05.CAP229_7w.GQ999985       AG-TGGCGCC CGAACAGG-- ---------- ---------- ---GACTTGA AA-GCGAAAG TAAGA----- ---------- ---------- ----------
C.ZA.05.CAP239_5w_F1.GQ999991    AG-CGGCGCC CGAACAGG-- ---------- ---------- ---GACTTGA AA-GCGAAAG TAAGA----- ---------- ---------- ----------
C.ZA.05.CAP244_8w_F1.GQ999986    AG-TGGAACC CGAACAGG-- ---------- ---------- ---GACTTGA AA-GCGAAAG TAAGA----- ---------- ---------- ----------
C.ZA.05.CAP248_9w.GQ999987       AG-TGGCGCC CGAACAGG-- ---------- ---------- ---GACCAGA AA-GCGAAAG TAAGA----- ---------- ---------- ----------
C.ZA.05.CAP255_8w_F1.GQ999988    AG-TGGCGCC CGAACAGG-- ---------- ---------- ---GACCTGA AA-GCGAAAG CAAGA----- ---------- ---------- ----------
C.ZA.05.CAP256_6w.GQ999989       AG-TGGCGCC CGAACAGG-- ---------- ---------- ---GACCTGA AA-GCGAAAG TAAGA----- ---------- ---------- ----------
C.ZA.05.CAP257_7w_F1.GQ999990    AG-TGGCGCC CGAACAGG-- ---------- ---------- ---GACTTGA AA-GCGAAAG TAAGA----- ---------- ---------- ----------
C.ZA.05.CAP45_5w_F1.GQ999974     AG-TGGCGCC CGAACAGG-- ---------- ---------- ---GACCTGA AA-GCGAAAG TAAGA----- ---------- ---------- ----------
C.ZA.05.CAP63_5w_F4.GQ999976     AG-TGGCGCC CGAGCAGG-- ---------- ---------- ---GACGCGA AA-GCGAAAG TAGGA----- ---------- ---------- ----------
C.ZA.05.CAP65_6w.GQ999977        AG-TGGCGCC CGAACAGG-- ---------- ---------- ---GACCCGA AA-GCGAAAG TAGAA----- ---------- ---------- ----------
C.ZA.05.CAP84_3w_F2.GQ999978     AG-TGGCGCC CGAACAGG-- ---------- ---------- ---GACTTGA AA-GCGAAAG TAAGA----- ---------- ---------- ----------
C.ZA.05.CAP85_5w_F1.GQ999979     AG-TGGCGCC CGAACAGG-- ---------- ---------- ---GACTTGA AA-ACGAAAG TAAGA----- ---------- ---------- ----------
C.ZA.05.CAP88_5w_F2.GQ999980     AG-TGGCGCC CGAACAGG-- ---------- ---------- ---GACTTGA AA-GCGAAAG TAAGA----- ---------- ---------- ----------
C.ZA.05.CAP8_3w_F2.GQ999972      AG-TGGCGCC CGAACAGG-- ---------- ---------- ---GACTTGA AA-GCGAAAG TAAAA----- ---------- ---------- ----------
C.ZA.07.DEMC07ZA011.JX140664     AG-TGGCGCC CGAACAGG-- ---------- ---------- ---GACTTGA AA-GCGAAAG TAAGA----- ---------- ---------- ----------
C.ZA.08.DEMC08ZA011.JX140666     -G-TGGCGCC CGAACAGG-- ---------- ---------- ---GACCCGA AA-GCGAAAG TAGGA----- ---------- ---------- ----------
C.ZA.09.DEMC09ZA008.JX140667     -G-TGGCGCC CGAACAGG-- ---------- ---------- ---GACTTGA AA-GCGAAAG TAAGA----- ---------- ---------- ----------
C.ZA.09.DEMC09ZA009.JX140668     -G-TGGCGCC CGAACAGG-- ---------- ---------- ---GACCAGA AA-GCGAAAG TAAGA----- ---------- ---------- ----------
C.ZA.10.DEMC10ZA001.JX140669     ------CGCC CGAACAGG-- ---------- ---------- ---GACTTGA AA-GCGAAAG TAAGA----- ---------- ---------- ----------
C.ZA.90.pZAC_R3714.JN188292      AG-TGGCGCC CGAACAGG-- ---------- ---------- ---GACTAGA AA-ACGAAAG TAAGG----- ---------- ---------- ----------
C.ZA.97.97ZA003.AY118165         ---------- ---------- ---------- ---------- ------TTGA AA-GCGAAAG TAAAA----- ---------- ---------- ----------
C.ZA.97.97ZA009.AY118166         ---------- ---------- ---------- ---------- ------CCGA AA-GCGAAAG TGAGA----- ---------- ---------- ----------
C.ZA.97.97ZA012.AF286227         ---------- ---------- ---------- ---------- ------TTGA AA-ACGAAAG TAAGA----- ---------- ---------- ----------
C.ZA.98.98ZA445.AY158533         ---------- ---------- ---------- ---------- -------TGA AA-GCGAAAG TGAGA----- ---------- ---------- ----------
C.ZA.98.98ZA502.AY158534         ---------- ---------- ---------- ---------- ------TTGA AA-GCGAAAG TAGAA----- ---------- ---------- ----------
C.ZA.98.98ZA528.AY158535         ---------- ---------- ---------- ---------- -------AGA AA-GCGAAAG TAAGA----- ---------- ---------- ----------
C.ZA.98.TV001.AY162223           AG-TGGCGCC CGAACAGG-- ---------- ---------- ---GACCAGA AA-GTGAAAG TGAGA----- ---------- ---------- ----------
C.ZA.98.TV002.AY162224           AG-TGGCGCC CGAACAGG-- ---------- ---------- ---GACTTGA AA-GCGAAAG TGAGA----- ---------- ---------- ----------
C.ZA.98.TV012.AY162225           AG-TGGCGCC TGAACAGG-- ---------- ---------- ---GACTTGA AA-GCGAAAG TAAGA----- ---------- ---------- ----------
C.ZA.99.99ZACM9.AF411967         AG-TGGCGCC CGAACAGG-- ---------- ---------- ---GACTTGA AA-GCGAAAG TAAGA----- ---------- ---------- ----------
C.ZA.99.99ZALT21.EU293446        AG-TGGCGCC CGAACAGG-- ---------- ---------- ---GACGCGA AA-GCGAAAG TAAGA----- ---------- ---------- ----------
C.ZA.99.99ZALT39.EU293447        AG-TGGCGCC CGAACAGG-- ---------- ---------- ---GACCCGA AA-GCGAAAG TAAGA----- ---------- ---------- ----------
C.ZA.99.99ZALT42.EU293448        AG-TGGCGCC CGAACAGG-- ---------- ---------- ---GACGCGA AA-GCGAAAG TAGAA----- ---------- ---------- ----------
C.ZA.99.99ZALT45.EU293449        AG-TGGCGCC CGAACAGG-- ---------- ---------- ---GACCCGA AA-GCGAAAG TAAGA----- ---------- ---------- ----------
C.ZA.99.99ZALT46.EU293450        AG-TGGCGCC CGAACAGG-- ---------- ---------- ---GACGCGA AA-GCGAAAG TAAGA----- ---------- ---------- ----------
C.ZA.99.99ZALT4.EU293444         AG-TGGCGCC CGAACAGG-- ---------- ---------- ---GACTTGA AA-GCGAAAG TAAGA----- ---------- ---------- ----------
C.ZA.99.99ZALT5.EU293445         AG-TGGCGCC CGAACAGG-- ---------- ---------- ---GACCCGA AA-GCGAAAG TAGGA----- ---------- ---------- ----------
C.ZA.99.99ZATM10.AY228556        AG-TGGCGCC CGAACAGG-- ---------- ---------- ---GACTTGA AA-GCGAAAG TAAGA----- ---------- ---------- ----------
C.ZA.99.ZASW7.AF411966           AG-TGGCGCC CGAACAGG-- ---------- ---------- ---GACCCGA AA-GCGAAAG TAAGA----- ---------- ---------- ----------
C.ZM.02.02ZM108.AB254141         AG-TGGCGCC CGAACAGG-- ---------- ---------- ---GACGCGA AA-GCGAAAG TAGAA----- ---------- ---------- ----------
C.ZM.02.02ZM110.AB254142         AG-TGGCGCC CGAACAGG-- ---------- ---------- ---GACGCGA AA-GCGAAAG TACGA----- ---------- ---------- ----------
C.ZM.02.02ZM114.AB254146         AG-TGGCGCC CGAACAGG-- ---------- ---------- ---GACTTGA AA-GCGAAAG TAAAA----- ---------- ---------- ----------
C.ZM.02.02ZM115.AB254148         AG-TGGCGCC CGAACAGG-- ---------- ---------- ---GACTTGA AA-GCGAAAG TAAGA----- ---------- ---------- ----------
C.ZM.02.02ZMBC.AB254149          AG-TGGCGCC CGAACAGG-- ---------- ---------- ---GACGCGA AA-GTGAAAG TAAGA----- ---------- ---------- ----------
C.ZM.02.02ZMDB.AB254153          AG-TGGCGCC CGAACAGG-- ---------- ---------- ---GACTTGA AA-GCGAAAG TAAAA----- ---------- ---------- ----------
C.ZM.02.02ZMJC.AB254155          AG-TGGCGCC CGAACAGG-- ---------- ---------- ---GACGCGA AA-GCGAAAG TAAGA----- ---------- ---------- ----------
C.ZM.03.ZM246F_flA10.FJ496186    AG-TGGCGCC CGAACAGG-- ---------- ---------- ---GACTTGA AA-GCGAAAG TAAAA----- ---------- ---------- ----------
C.ZM.03.ZM247F_flA1.FJ496195     AG-TGGCGCC CGAACAGG-- ---------- ---------- ---GACTTGA AA-GCGAAAG TAAGA----- ---------- ---------- ----------
C.ZM.03.ZM249M_flC1.FJ496209     AG-TGGCGCC CGAACAGG-- ---------- ---------- ---GACCCGA AA-GCGAAAG TAGAA----- ---------- ---------- ----------
C.ZM.89.ZAM18.AB485645           AG-TGGCGCC CGAACAGG-- ---------- ---------- ---GACCTGA AA-GCGAAAG TAAGA----- ---------- ---------- ----------
```

C.ZM.96.96ZM651.AF286224	----------	----------	----------	----------	------CTTG	AA-GCGAAAG	TAGA------	----------	----------	----------
C.ZM.96.96ZM751.AF286225	----------	----------	----------	----------	------CCGA	AA-GCGAAAG	TAAGA-----	----------	----------	----------
C.ZM.x.HIV1084i.AY805330	AG-TGGCGCC	CGAACAGG--	----------	----------	---GACTTGA	AA-GCGAAAG	TAAGA-----	----------	----------	----------
D.CD.83.ELI.K03454	AG-TGGCGCC	CGAACAGG--	----------	----------	---GACCTGA	AA-GCGAAAG	TAGAA-----	----------	----------	----------
D.CD.83.NDK.M27323	AG-TGGCGCC	CGAACAGG--	----------	----------	---GACCTGA	AA-GCGAAAG	TAGAA-----	----------	----------	----------
D.CD.84.84ZR085.U88822	AG-TGGCGCC	CGAACAGG--	----------	----------	---GACCTGA	AA-GCGAAAG	TAGAA-----	----------	----------	----------
D.CD.85.Z2Z6_Z2_CDC_Z34.M22639	AG-TGGCGCC	CGAACAGG--	----------	----------	---GACCTGA	AA-GCGAAAG	TAGAA-----	----------	----------	----------
D.CM.10.DEMD10CM009.JX140670	-G-TGGCGCC	CGAACAGG--	----------	----------	---GACTTGA	AA-GCGAAAG	TAGAA-----	----------	----------	----------
D.KE.93.MB2059.AF133821	AG-TGGCGCC	CGAACAGG--	----------	----------	---GACTTGA	AA-GCGAAAG	TAGAA-----	----------	----------	----------
D.KE.97.ML415_2.AY322189	AG-TGGCGCC	CGAACAGG--	----------	----------	---GACTTGA	AA-GCGAAAG	TAGAA-----	----------	----------	----------
D.KR.04.04KBH8.DQ054367	AG-TGGCGCC	CGAACAGG--	----------	----------	---GACTTGA	AA-GCGAAAG	TAGAA-----	----------	----------	----------
D.SN.90.SE365.AB485648	AG-TGGCGCC	CGAACAGG--	----------	----------	---GACCCGA	AA-GCGAAAG	TAGAA-----	----------	----------	----------
D.UG.05.p190049.JX236668	AG-TGGCGCC	CGAACAGG--	----------	----------	---GACTTGA	AA-GTGAAAG	TAGAA-----	----------	----------	----------
D.UG.07.p191647.JX236670	AG-TGGCGCC	CGAACAGG--	----------	----------	---GACTTGA	AA-GCGAAAG	TAGAA-----	----------	----------	----------
D.UG.07.p191882.JX236673	AG-TGGCGCC	CGAACAGG--	----------	----------	---GACCTGA	AA-GCGAAAG	TAGAA-----	----------	----------	----------
D.UG.07.pSC191727.JX236679	AG-TGGCGCC	CGAACAGG--	----------	----------	---GACTTGA	AA-GCGAAAG	TAGAA-----	----------	----------	----------
D.UG.08.p191859.JX236672	AG-TGGCGCC	CGAACAGG--	----------	----------	---GACGCGA	AA-GCGAAAG	TAGAA-----	----------	----------	----------
D.UG.91.UG270.AB485650	AG-TGGCGCC	CGAACAGG--	----------	----------	---GACTTGA	AA-GCGAAAG	TAGAA-----	----------	----------	----------
D.UG.92.92UG001.AJ320484	AG-TGGCGCC	CGAACAGGGA	CC--------	----------	------TGAA	AA-GCGAAAG	TAAAA-----	----------	----------	----------
D.UG.94.94UG114.U88824	----------	----------	----------	----------	------CTGA	AA-GCGAAAG	TAGAA-----	----------	----------	----------
D.ZA.84.R2.AY773338	----------	----------	----------	----------	----------	----------	----------	----------	----------	----------
D.ZA.85.R286.AY773340	----------	----------	----------	----------	----------	----------	----------	----------	----------	----------
D.ZA.86.R482.AY773341	----------	----------	----------	----------	----------	----------	----------	----------	----------	----------
D.ZA.90.R1.EF633445	----------	----------	----------	----------	--------TC	GG-CTGGAAC	GAGTT-----	----------	----------	----------
F1.AR.02.ARE933.DQ189088	----------	----------	----------	----------	----------	----------	----------	----------	----------	----------
F1.BE.93.VI850.AF077336	----------	----------	----------	----------	---GGACTTG	AA-GCGAAAG	TAGAA-----	----------	----------	----------
F1.BR.02.02BR082.FJ771006	AG-TGGCGCC	CGAACAGG--	----------	----------	---GACTTGA	AA-GTGAAAG	TAGAA-----	----------	----------	----------
F1.BR.02.02BR170.FJ771007	AG-TGGCGCC	CGAACAGG--	----------	----------	---GACTTGA	AA-GCGAAAG	TAGAA-----	----------	----------	----------
F1.BR.06.06BR564.FJ771008	AG-TGGCGCC	CGAACAGG--	----------	----------	---GACCTGA	AA-GCGAAAG	TAGAA-----	----------	----------	----------
F1.BR.06.06BR579.FJ771009	AG-TGGCGCC	CGAACAGG--	----------	----------	---GACGCGA	AA-GCGAAAG	TAGAA-----	----------	----------	----------
F1.BR.07.07BR844.FJ771010	AG-TGGCGCC	CGAACAGG--	----------	----------	---GACGCGA	AA-ACGAAAG	TAGAA-----	----------	----------	----------
F1.BR.89.BZ126.AY173957	AG-TGGCGCC	CGAACAGG--	----------	----------	---GACGCGA	AA-GCGAAAG	TAGAA-----	----------	----------	----------
F1.BR.93.93BR020_1.AF005494	----------	----------	----------	----------	------CTGA	AA-GCGAAAG	TAAA------	----------	----------	----------
F1.DE.x.MVP_30846.EU446022	----------	----------	----------	----------	----------	--AAGCGAAA	GCGAA-----	----------	----------	----------
F1.ES.02.ES_X845_4.FJ670516	AG-TGGCGCC	CGAACAGG--	----------	----------	--GACGCGAA	AA-GCGAAAA	TAAAA-----	----------	----------	----------
F1.ES.11.DEMF110ES001.JX140671	----------	----------	----------	----------	--GGACCTGA	AA-ACGAAAG	TAGAA-----	----------	----------	----------
F1.ES.x.P1146.DQ979023	----------	----------	----------	----------	----------	----------	----------	----------	----------	----------
F1.ES.x.X1093_2.DQ979025	AG-TGGCGCC	CGAACAGG--	----------	----------	---GACGCGA	AA-GCGAAAG	TAGAA-----	----------	----------	----------
F1.ES.x.X1670.DQ979024	AG-TGGCGCC	CGAACAGG--	----------	----------	---GACYYGA	AA-GCGAAAG	TAGAA-----	----------	----------	----------
F1.FI.93.FIN9363.AF075703	----------	----------	----------	----------	---------A	AA-GCGAAAG	TAGAA-----	----------	----------	----------
F1.RO.96.BCI_R07.AB485658	AG-TGGCGCC	CGAACAGG--	----------	----------	---GACTTGA	AA-GCGAAAG	TAGAA-----	----------	----------	----------
F1.RU.08.D88_845.GQ290462	AG-TGGCGCC	CGAACAGG--	----------	----------	---GACGCGA	AA-GCGAAAG	TAGAA-----	----------	----------	----------
F2.CM.10.DEMF210CM007.JX140673	-G-TGGCGCC	CGAACAGG--	----------	----------	---GACCTGA	AA-GCGAAAG	TAGAA-----	----------	----------	----------
G.BE.96.DRCBL.AF084936	AG-TGGCGCC	CGAACAGGGA	CTTGAAA-AC	GAAAGTTAAT	AGGGACTCGA	AA-GCGAAAG	TT--------	----------	----------	----------
G.CM.01.A1786.FJ389367	AG-TGGCGCC	CGAACAGGGA	CTTGAAA-GC	GAAAGTTAAT	AGGGACTCGA	AA-GCGAAAG	TT--------	----------	----------	----------
G.CM.04.178_15.FJ389363	AG-TGGCGCC	CGAACAGGGA	CTTGAAA-AC	GAAAGTTAAC	AGGGACTCGA	AA-GCGGAAG	TT--------	----------	----------	----------
G.CM.04.314_40.FJ389364	AG-TGGCGCC	CGAACAGGGA	CTTGAAA-AC	GAAAGTTAAT	AGGGACTCGA	AA-GCGAAAG	TT--------	----------	----------	----------
G.CM.04.515_28.FJ389365	AG-TGGCGCC	CGAACAGGGA	CKYGAAA-GC	GAAAGTTAAM	AGGGACTCGA	AA-GCGRAAG	TT--------	----------	----------	----------
G.CM.04.944_5.FJ389366	AG-TGGCGCC	CGAACAGGGA	CTTGAAA-AC	GAAAGTTAAT	AGGGACTCGA	AA-GCGAAAG	TT--------	----------	----------	----------
G.CM.10.DEMG10CM008.JX140676	---TGGCGCC	CGAACAGGGA	CTTGAAA-GT	GAAAGTTAAT	AGGGACTCGA	AA-GCGAAAG	TT--------	----------	----------	----------
G.CM.96.96CMABB55.AY772535	AG-TGGCGCC	CGAACAGGGA	CTTGAAA-GC	GAAAGTTAAA	AGGGACTCGA	AA-GCGAAAG	TT--------	----------	----------	----------
G.CN.06.sh52.HM067749	AG-TGGCGCC	CGAACAGG--	----------	----------	---GACTTGA	AA-GCGAAAG	TTAATATGGG	AATGGAAAGG	GAAAGTT---	----------
G.CU.99.Cu74.AY586547	AG-TGGCGCC	CGAACAGGGA	CTTGAAA-GC	GAAAGTTAAC	AGGGACTCGA	AA-GCGAAAG	TT--------	----------	----------	----------
G.CU.99.Cu85.AY586548	AG-TGGCGCC	CGAACAGGGA	CTTGAAA-GY	GAAAGTTAAT	AGGGACTCGA	AA-GCGAAAG	TT--------	----------	----------	----------
G.CU.99.Cu87.AY586549	AG-TGGCGCC	CGAACAGGGA	CTTGAAA-GC	GAAAGTTAAT	AGGGACTCGA	AA-GCGAAAG	TT--------	----------	----------	----------
G.ES.05.P962.EU786670	AG-TGGCGCC	CGAACAGGGA	CTTGAAA-AC	GAAAGTTAAC	AGGGACTTGT	AA-GCGGAAG	TTAACGGGGA	CTCGAAAGCG	AAAGTT----	----------
G.ES.08.P1981_2.FJ670530	AG-TGGCGCC	CGAACAGG--	----------	----------	---GACTTGA	AA-GAGAAAG	TTAAAAGGGA	CTCGAAAGCG	AAAGTT----	----------
G.ES.09.X2634_2.GU362882	AG-TGGCGCC	CGAACAGG--	----------	----------	---GACTTGA	AA-GCGAAAG	TTAACAGGGA	CTCGAAAGCG	AAAGTT----	----------
G.ES.99.X138.AF450098	AG-TGGCGCC	CGAACAGGGA	CTTGAAA-GC	GAAAGTTMAC	AGGSACTCGA	AA-GCGAAAG	TT--------	----------	----------	----------
G.GH.03.03GH175G.AB287004	AG-TGGCGCC	CGAACAGGGA	CCCGAAA-GT	GAAAGTTAAT	AGGGACTCGA	AA-GCGAAAG	TT--------	----------	----------	----------
G.KE.93.HH8793_12_1.AF061641	AG-TGGCGCC	CGAACAGGGA	CTTGAAA-G-	TGAAGTTAAC	AGGGACTCGA	AA-GCGAAAG	TT--------	----------	----------	----------
G.NG.92.92NG083_JV10832.U88826	----------	----------	-ATGAAA-GC	GAAAGTTAAT	AGGGACTCGA	AA-ACGAAAG	TT--------	----------	----------	----------
G.PT.x.PT2695.AY612637	AG-TGGCGCC	CGAACAGGGA	CTTGAAA-GC	GAAAGTTAAA	CGGGACTCGA	AA-GCGAAAG	TT--------	----------	----------	----------
G.PT.x.PT3037.FR846408	AG-TGGCGCC	CGAACAGGGA	CCTGAAA-GC	GAAAGTTAAT	AGGGACTCGA	AA-GCGAAAG	TT--------	----------	----------	----------
G.PT.x.PT3306.FR846409	AG-TGGCGCC	CGAACAGGGA	CTTGGAA-GC	GAAAGTTAAC	AGGGACTCGA	AA-GCGAAAG	TT--------	----------	----------	----------
G.PT.x.PT988.FR846410	AG-TGGCGCC	CGAACAGG--	----------	----------	---GACTCGA	AA-GCGAAAG	TT--------	----------	----------	----------
G.SE.93.SE6165_G6165.AF061642	AG-TGGCGCC	CGAACAGGGA	CTTGAAA-GC	GAAAGTTAAC	AGGGACTCGA	AA-GCGAAAG	TT--------	----------	----------	----------
H.BE.93.VI991.AF190127	AG-TGGCGCC	CGAACAGG--	----------	----------	---GACTTGA	AA-GCGAAAG	CAAAA-----	----------	----------	----------
H.BE.93.VI997.AF190128	----------	----------	----------	----------	----------	----------	----------	----------	----------	----------
H.CF.90.056.AF005496	----------	----------	----------	----------	------TTGA	TA-GTGAAAG	TAAAA-----	----------	----------	----------
H.GB.00.00GBAC4001.FJ711703	AG-TGGCGCC	CGAACAGG--	----------	----------	---GACTTGA	AA-GCGAAAG	TAAAA-----	----------	----------	----------
J.CM.04.04CMU11421.GU237072	AG-TGGCGCC	CGAACAGG--	----------	----------	--GACCTTGA	AA-GTGAAAG	TAGGA-----	----------	----------	----------
J.SE.93.SE9280_7887.AF082394	----------	----------	----------	----------	----------	----------	----------	----------	----------	----------
J.SE.94.SE9173_7022.AF082395	----------	----------	----------	----------	----------	----------	----------	----------	----------	----------
01_AE.CF.90.90CF11697.AF197340	AG-TGGCGCC	CGAACAGGGA	CTTGAAA-GC	GAAAGTTAAT	AGGGACTCGA	AA-GCGAAAG	TT--------	----------	----------	----------
01_AE.CF.90.90CF4071.AF197341	AG-TGGCGCC	CGAACAGGGA	CTTGAAA-GC	GAAAGTTAAC	AGGGACTCGA	AA-GCGAAAG	TT--------	----------	----------	----------
01_AE.CF.90.90CR402_CAR_E_4002	AG-TGGCGCC	CGAACAGGGA	CTTGAAA-GC	GAAAGTTAAT	AGGGACTCGA	AA-GCGAAAG	TT--------	----------	----------	----------
01_AE.CN.05.FJ051.DQ859178	AG-TGGCGCC	CGAACAGGGA	CTTGAAA-GT	GAAAGTTAAT	AGGGACTCGA	AA-GCGGAAG	TT--------	----------	----------	----------
01_AE.CN.05.FJ053.DQ859179	AG-TGGCGCC	CGAACAGGGA	CTTGAAA-GC	GAAAGTTAAT	AGGGACTCGA	AA-GCGAAAG	TT--------	----------	----------	----------
01_AE.CN.05.Fj052.EF036528	AG-TGGCGCC	CGAACAGGGA	CTTGAAA-GC	GAAAGTTAAT	AGGGACTCGA	AA-GCGAAAG	TT--------	----------	----------	----------
01_AE.CN.05.Fj055.EF036527	AGGTGGCGCC	CGAACAGGGA	CTTGAAA-GC	GAAAGTTAAT	AGGGACTCGA	AA-GCGAAAG	TT--------	----------	----------	----------
01_AE.CN.05.Fj056.EF036529	AG-TGGCGCC	CGAACAGGGA	CTTGAAA-GC	GAAAGTTAAT	AGGGACTCGA	AA-GCGAAAG	TT--------	----------	----------	----------
01_AE.CN.05.Fj057.EF036530	AG-TGGCGCC	CGAACAGGGA	CTTGAAA-GC	GAAAGTTAAT	AGGGACTCGA	AA-GCGAAAG	TT--------	----------	----------	----------
01_AE.CN.05.Fj065.EF036534	AG--GGCGCC	CGAACAGGGA	CCTTGAA-GC	GGAAGTTAAT	AGGGACTCGA	AA-GCGAAAG	TT--------	----------	----------	----------
01_AE.CN.05.Fj066.EF036535	AG-TGGCGCC	CGAACAGGGA	CTTGA---GT	GACAGTTAAT	AGGGACTCGA	AA-GCGAAAG	TT--------	----------	----------	----------
01_AE.CN.06.FJ054.DQ859180	AG-TGGCGCC	CGAACAGGGA	CTTGAAA-GC	GAAAGTTAAT	AGGGACTCGA	AA-GCGGAAG	TT--------	----------	----------	----------
01_AE.CN.06.Fj062.EF036531	AG-TGGTGCC	CGAACAGGGA	CTTGAAA-GC	GAAAGTTAAT	AGGGACTCGA	AA-GCGAAAG	TT--------	----------	----------	----------
01_AE.CN.06.Fj063.EF036532	AG-TGGCGCC	CGAACAGGGA	CTCGAAA-GC	GAAAGTTAAT	AGGGACTCGA	AA-GCGAAAG	TT--------	----------	----------	----------
01_AE.CN.06.Fj064.EF036533	AG-TGGCGCC	CGAACAGGGA	CTTGAAA-GC	GAAAGTTAAT	AGGGACTCGA	AA-GCGAAAG	TT--------	----------	----------	----------
01_AE.HK.04.HK001.DQ234790	----------	CGAACAGGGA	CTTGAAA-GC	GAAAGTTAAT	AGGGACTCGA	AA-GCGAAAG	TT--------	----------	----------	----------
01_AE.JP.x.DR0492.AB253423	AG-TGGCGCC	CGAACAGGGA	CTTGAAA-GT	GAAAGTTAAT	AGGGACTCGA	AA-GCGAAAG	TT--------	----------	----------	----------
01_AE.JP.x.DR2594.AB253660	AG-TGGCGCC	CGAACAGGGA	CTTGAAA-GC	GAAAGTTAAT	AGGGACTCGA	AA-GCGAAAG	TT--------	----------	----------	----------
01_AE.JP.x.DR6824.AB253426	AG-TGGCGCC	CGAACAGGGA	CTTGAAA-GT	GAAAGTTAAT	AGGGACTCGA	AA-GCGAAAG	TT--------	----------	----------	----------
01_AE.JP.x.JRC77AE.AB565504	AG-TGGCGCC	CGAACAGGGA	CTTGAAA-AC	GAAAGTTAAT	AGGGACTCGA	AA-GCGAAAG	TT--------	----------	----------	----------
01_AE.TH.04.AA027a_wg4.JX44702	----------	-------GGA	CTTGAAA-GC	GAAAGTTAAT	AGGGACTCGA	AA-GCGAAAG	TT--------	----------	----------	----------
01_AE.TH.04.AA075a_WG7.JX44754	AG-TGGCGCC	CGAACAGGGA	CTTGAAA-GC	GAAAGTTAAT	AGGGACTCGA	AA-GCGAAAG	TT--------	----------	----------	----------
01_AE.TH.04.BKM.DQ314732	AG-TGGCGCC	CGAACAGGGA	CTTGAAA-GC	GAAAGTTAAT	AGGGACTCGA	AA-GCGAAAG	TT--------	----------	----------	----------
01_AE.TH.05.AA004a_wg4a.JX4467	AG-TGGCGCC	CGAACAGGGA	CTTGAAA-GC	GAAAGTTAAT	AGGGACTCGA	AA-GCGAAAG	TT--------	----------	----------	----------
01_AE.TH.05.AA033a_wg6a.JX4470	AG-TGGCGCC	CGAACAGGGA	CTTGAAA-GC	GAAAGTTAAT	AGGGACTCGA	AA-GCGAAAG	TT--------	----------	----------	----------
01_AE.TH.05.AA049a_WG13.JX4472	----------	----------	----------	----------	----------	-----GAAAG	TT--------	----------	----------	----------
01_AE.TH.05.AA064a_WG2.JX44741	----------	----------	----------	----------	----------	----CGAAAG	TT--------	----------	----------	----------
01_AE.TH.05.AA079a_WG4.JX44759	---TGGCGCC	CGAACAGGGA	CTTGAAA-GT	GAAAGTTAAT	AGGGACTCGA	AA-GCGGAAG	TT--------	----------	----------	----------
01_AE.TH.05.AA101a_WG1.JX44793	-G-TGGCGCC	CGAACAGGGA	CTTGAAA-GC	GAAAGTTAAT	AGGGACTCGA	AA-GCGAAAG	TT--------	----------	----------	----------
01_AE.TH.05.AA107a_wg4.JX44802	----------	--------GA	CTTGAAA-GC	GAAAGTTAAT	AGGGACTCGA	AA-GCGAAAG	TT--------	----------	----------	----------
01_AE.TH.06.AA002a_WG1.JX44666	AG-TGGCGCC	CGAACAGGGA	CTTGAAA-GC	GAAAGTAAA-	AGGGACTCGA	AA-GCGAAAG	GTT-------	----------	----------	----------

```
01_AE.TH.06.AA017a_wg1.JX44689     ---------C CGAACAGGGA CTTGAAA-GT GAAAGTTAAT AGGGACTCGA AA-GCGAAAG TT-------- ---------- ---------- ----------
01_AE.TH.06.AA034a_wg2.JX44708     AG-TGGCGCC CGAACAGGGA CTTGAAA-GT GAAAGTTAAT AGGGACTCGA AA-GCGAAAG GTT------- ---------- ---------- ----------
01_AE.TH.06.AA038a_WG3.JX44713     ---------- -GAACAGGGA CTTGAAA-GC GAAAGTTAAT AGGGACTCGA AA-GCGAAAG TT-------- ---------- ---------- ----------
01_AE.TH.06.AA055a_WG4.JX44731     ---------- --------GA CTTGAAA-GT GAAAGATAAT AGGGACTCGA AA-GCGAAAG TT-------- ---------- ---------- ----------
01_AE.TH.06.AA056a_WG5.JX44731     AG-TGGCGCC CGAACAGGGA CTTGAAA-GC GAAAGTTAAT AGGGACTCGA AA-GCGAAAG TT-------- ---------- ---------- ----------
01_AE.TH.06.AA059a_WG5.JX44735     AG-TGGCGCC CGAACAGGGA CCTGAAA-AC GAAAGTTAAT AGGGACTCGA AA-GCGAAAG TT-------- ---------- ---------- ----------
01_AE.TH.06.AA063a_WG37.JX4474     AG-TGGCGCC CGAACAGGGA CTTGAAA-GT GAAAGTTAAT AGGGACTCGA AA-GCGAAAG TT-------- ---------- ---------- ----------
01_AE.TH.06.AA068a_14.JX447465     ---------- ---------- -TTGAAA-GC GAAAGTTAAT AGGGACTCGA AA-GCGAAAG TT-------- ---------- ---------- ----------
01_AE.TH.06.AA082a_WG9.JX44764     AG-TGGCGCC CGAACAGGGA CTTGAAA-GC GAAAGTTAAT AGGGACTCGA AA-GCGAAAG TT-------- ---------- ---------- ----------
01_AE.TH.06.AA085a_wg2.JX44768     AG-TGGCGCC CGAACAGGGA CTTTAAA-GT GAAAGTAAA- AGGGACTCGA AA-GCGGAAG TT-------- ---------- ---------- ----------
01_AE.TH.06.AA088a_wg14.JX4477     AG-TGGCGCC CGAACAGGGA CTTGAAA-GC GAAAGTTAAT AGGGACTCGA AA-GCGAAAG TT-------- ---------- ---------- ----------
01_AE.TH.06.AA099a_WG9.JX44789     AG-TGGCGCC CGAACAGGGA CTTGAAA-GT GAAAGTTAAT AGGGACTCGA AA-GCGCAAG TT-------- ---------- ---------- ----------
01_AE.TH.07.AA015a_WG4.JX44687     AG-TGGCGCC CGAACAGGGA CTTGAAA-GC GAAAGTTAAT AGGGACTCGA AA-GCGAAAG TT-------- ---------- ---------- ----------
01_AE.TH.07.AA019a_WG11.JX4469     AG-TGGCGCC CGAACAGGGA CTTGAAA-GT GAAAGTTAAT AGGGACTCGA AA-ACGAAAG GTT------- ---------- ---------- ----------
01_AE.TH.07.AA028a_wg3.JX44702     AG-TGGCGCC CGAACAGGGA CTTGAAA-AC GAAAGTTAAT AGGGACTCGA AA-GCGAAAG TT-------- ---------- ---------- ----------
01_AE.TH.07.AA050a_WG7.JX44728     ---------- --------GA CTTGAAA-AC GAAAGTTAAT AGGGACTCGA AA-GCGAAAG TT-------- ---------- ---------- ----------
01_AE.TH.08.AA007a_WG10.JX4467     ---------- --------GA CTTGAAA-GC GAAAGTTAAT AGGGACTCGA AA-GCGGAAG TT-------- ---------- ---------- ----------
01_AE.TH.08.AA037a_WG6.JX44712     AG-TGGCGCC CGAACAGGGA CTTGAAA-GT GAAAGTTAAT AGGGACTCGA AA-GCGGAAG TT-------- ---------- ---------- ----------
01_AE.TH.08.AA060a_WG1.JX44735     ---------- ---------- ---------- ---------- --------GA AA-GCGAAAG TT-------- ---------- ---------- ----------
01_AE.TH.08.AA067a_WG12.JX4474     AG-TGGCGCC CGAACAGGGA CTTGAAA-GC GAAAGTTAAT AGGGACTCGA AA-GCGAAAG TT-------- ---------- ---------- ----------
01_AE.TH.08.AA108a_WG6.JX44802     AG-TGGCGCC CGAACAGGGA CTTGAAA-GC GAAAGTTAAT AGGGACTCGA AA-GCGAAAG TT-------- ---------- ---------- ----------
01_AE.TH.09.AA090a_WG11.JX4477     AG-TGGCGCC CGAACAGGGA CTTGAAA-GC GAAAGTTAAT AGGGACTCGA AA-GCGAAAG TT-------- ---------- ---------- ----------
01_AE.TH.09.AA111a_WG11.JX4480     AG-TGGCGCC CGAACAGGGA CTTGAAA-GC GAAAGTTAAT AGGGACTCGA AA-GCGAAAG TT-------- ---------- ---------- ----------
01_AE.TH.90.CM240.U54771           AG-TGGCGCC CGAACAGGGC ACTCGAAAGC GAAAGTTAAT AGGGACTCGA AA-GCGAAAG TT-------- ---------- ---------- ----------
01_AE.TH.93.93TH253.U51189         AG-TGGCGCC CGAACAGGGA CTTGAAA-GC GAAAGTTAAT AGGGACTCGA AA-GCGAAAG TT-------- ---------- ---------- ----------
01_AE.TH.93.93TH9021.AF164485      AG-TGGCGCC CGAACAGGGA CTTGAAA-GC GAAAGCTAAT AGGGACTCGA AA-GCGAAAG TT-------- ---------- ---------- ----------
01_AE.TH.95.95TNIH022.AB032740     AG-TGGCGCC CGAACAGGGA CTTGAAA-GC GAAAGTTAAT AGGGACTCGA AA-GCGGAAG TT-------- ---------- ---------- ----------
01_AE.TH.95.95TNIH047.AB032741     AG-TGGCGCC CGAACAGGGA CTTGAAA-GT GAAAGTTAAT AGGGACTCGA AA-GCGGAAG TT-------- ---------- ---------- ----------
01_AE.TH.x.NP03.AB485654           AG-TGGCGCC CGAACAGGGA CTTGAAA-GT GAAAGTTAAT AGGGACTCGA AA-GCGAAAG TT-------- ---------- ---------- ----------
01_AE.VN.97.97VNAG204.FJ185247     ---------- ---------- ---------- ---------- ---------- ---------- ---------- ---------- ---------- ----------
01_AE.VN.97.97VNAG206.FJ185248     ---------- ---------- ---------- ---------- ---------- ---------- ---------- ---------- ---------- ----------
01_AE.VN.97.97VNAG207.FJ185249     ---------- ---------- ---------- ---------- ---------- ---------- ---------- ---------- ---------- ----------
01_AE.VN.97.97VNAG210.FJ185251     ---------- ---------- ---------- ---------- ---------- ---------- ---------- ---------- ---------- ----------
01_AE.VN.97.97VNAG212.FJ185252     ---------- ---------- ---------- ---------- ---------- ---------- ---------- ---------- ---------- ----------
01_AE.VN.97.97VNAG214.FJ185253     ---------- ---------- ---------- ---------- ---------- ---------- ---------- ---------- ---------- ----------
01_AE.VN.97.97VNAG218.FJ185255     ---------- ---------- ---------- ---------- ---------- ---------- ---------- ---------- ---------- ----------
01_AE.VN.97.97VNHCM314.FJ18524     ---------- ---------- ---------- ---------- ---------- ---------- ---------- ---------- ---------- ----------
01_AE.VN.97.97VNHCM319.FJ18524     ---------- ---------- ---------- ---------- ---------- ---------- ---------- ---------- ---------- ----------
01_AE.VN.97.97VNHCM343.FJ18524     ---------- ---------- ---------- ---------- ---------- ---------- ---------- ---------- ---------- ----------
01_AE.VN.97.97VNHCM345.FJ18524     ---------- ---------- ---------- ---------- ---------- ---------- ---------- ---------- ---------- ----------
01_AE.VN.98.98VNND15.FJ185235      ---------- ---------- ---------- ---------- ---------- ---------- ---------- ---------- ---------- ----------
02_AG.CM.08.DE00208CM001.JX140     AG-TGGCGCC CGAACAGGGA CCGG------ --AAGTTAAT AGGGACTCGA AA-GCGAAAG TT-------- ---------- ---------- ----------
02_AG.CM.08.DE00208CM004.JX140     -------GCC CGAACAGGGA CTTG------ --AAGTTAAT AGGGACTCGA AA-GCGAAAG TT-------- ---------- ---------- ----------
02_AG.CM.99.pBD6_15.AY271690       AG-TGGCGCC CGAACAGGGA CTTG------ --AAGTTAAT AGGGACTCGA AA-GCGAAAG TT-------- ---------- ---------- ----------
02_AG.ES.06.P1261.EU786671         AG-TGGCGCC CGAACAGGGA CCGG------ --AAGTTAAT AGGGACTCGA AA-GCGAAAG TT-------- ---------- ---------- ----------
02_AG.ES.06.P1423.EU884501         AG-TGGCGCC CGAACAGGGA CCGG------ --AAGTTAAT AGGGACTCGA AA-GCGAAAG TT-------- ---------- ---------- ----------
02_AG.FR.91.DJ263.AF063223         ---------- -----GGACC GGAA------ ----GCTAAT AGGGACTCGA AA-GCGAAAG TT-------- ---------- ---------- ----------
02_AG.GH.03.03GH181AG.AB286855     AG-TGGCGCC CGAACAGGGA CCTG------ --AAATTAAT AGGGACTCGA AA-GCGAAAG TT-------- ---------- ---------- ----------
02_AG.GH.03.03GH182AG.AB286857     AG-TGGCGCC CGAACAGGGA CCGG------ --AAGTTAAT AGGGACTCGA AA-GCGAAAG TT-------- ---------- ---------- ----------
02_AG.GH.03.03GH189AG.AB286862     AG-TGGCGCC CGAACAGGGA CCGG------ --AAGTTAAT AGGGACTCGA AA-GCGAAAG TT-------- ---------- ---------- ----------
02_AG.GH.03.03GH197AG.AB286863     AG-TGGCGCC CGAACAGGGA CTTG------ --GAGTTAAT AGGGACTCGA AA-GCGAAAG TG-------- ---------- ---------- ----------
02_AG.GH.03.GHNJ185.AB231895       AG-TGGCGCC CGAACAGGGA CTTGAAG--- -----TTAAT AGGGACTCGG AAAGCGAAAG TACCAGAGAA GTCTCTCCGA CGCAGTCGAA AGCGAAAGTT
02_AG.GH.03.GHNJ188.AB231896       AG-TGGCGCC CGAACAGGGA CCGG------ --AAGTTAAT AGGGACTCGA AA-GCGAAAG TT-------- ---------- ---------- ----------
02_AG.GH.03.GHNJ196.AB231898       AG-TGGCGCC CGAACAGGGA CCGG------ --AAGTTAAT AGGGACGCGA AA-GCGAAAG TT-------- ---------- ---------- ----------
02_AG.GH.97.97GH_AG1.AB049811      AG-TGGCGCC CGAACAGGGA CTTG------ --AAGTTAAT AGGGACTCGA AA-GCGAAAG TT-------- ---------- ---------- ----------
02_AG.GH.x.I_2496.AB485633         AG-TGGCGCC CGAACAGGGA CCGG------ --AAATTAAT AGGGACTCGA AA-GCGAAAG TT-------- ---------- ---------- ----------
02_AG.GW.04.CC_0030.FJ694791       ---------- --AACAGGGA C--------C GGAAGTTAAT AGGGACTCGA AA-GCGAAAG TT-------- ---------- ---------- ----------
02_AG.GW.05.CC_0048.FJ694792       ---------- ---------- ---------- -GAAGTTAAT AGGGACTCGA AA-GCGAAAG TT-------- ---------- ---------- ----------
02_AG.KR.07.07MHI10.JQ316136       AG-TGGCGCC CGAACAGGGA CTTG------ --AAGTTAAT AGGGACTCGA AA-GCGAAAG TT-------- ---------- ---------- ----------
02_AG.LR.x.POC44951.AB485636       AG-TGGCGCC CGAACAGGGA CTTG------ --AAGATAAT AGGGACTCGA AA-GCGAAAG TT-------- ---------- ---------- ----------
02_AG.NG.x.IBNG.L39106             AG-TGGCGCC CGAACAGGAC TTGAC----- ----GGTAAT AGGGACTCGA AA-GCGAAAG TT-------- ---------- ---------- ----------
02_AG.SE.94.SE7812.AF107770        AG-TGGCGCC CGAACAGGGA CTTG------ --AAGTTAAT AGGGACTCGA AA-GCGAAAG TT-------- ---------- ---------- ----------
03_AB.BY.00.98BY10443.AF414006     AG-TGGCGCC CGAACAGGGA CTTGAAA--- ---------- ---------- ---GCGAAAG TT-------- ---------- ---------- ----------
03_AB.RU.98.RU98001_98RU001.AF     AG-TGGCGCC CGAACAGGGA CTTGAAA--- ---------- ---------- ---GCGAAAG TT-------- ---------- ---------- ----------
04_cpx.CY.94.94CY032_3.AF04933     ---------- -----TTGAA AGTG------ -AAAGTTAAT AG-GACTCGA AA-GCGAAAG TT-------- ---------- ---------- ----------
04_cpx.GR.00.DE00400GR002.JX14     ---------- -GAACAGGGA CTTGAAA-GC GAAAGTTAAT AGGGACTCGA AA-GCGAAAG TT-------- ---------- ---------- ----------
04_cpx.GR.91.GR11_97PVCH.AF119     AG-TGCCGCC CGAACAGGGA CTTGAAA-GT GAAAGTTAAT AGGGACTCGA AA-GCGGAAG TT-------- ---------- ---------- ----------
04_cpx.GR.97.GR84_97PVMY.AF119     AG-TGGCGCC CGAACAGGGA CCCGAAA-GT GAAAGTTAAT AGGGACTCGA AA-GCGGAAG TT-------- ---------- ---------- ----------
05_DF.BE.93.VI961.AF076998         ---------- ---------- ---------- ---------- -GGACCTTGA AA-ACGAAAG TAGAA----- ---------- ---------- ----------
05_DF.BE.x.VI1310.AF193253         AG-TGGCGCC CGAACAGG-- ---------- ---------- ---GACTTGA AA-ACGAAAG TAGAA----- ---------- ---------- ----------
05_DF.ES.99.X492.AY227107          ---------- ---------- ---------- ---------- -GGGACTTGA AA-ACGAAAG TAAA------ ---------- ---------- ----------
06_cpx.AU.96.BFP90.AF064699        AG-TGGCGCC CGAACAGGGA CTTGAAA-GC GAAAGTTAAT AGGGACTCGA AA-GCGAAAG TT-------- ---------- ---------- ----------
06_cpx.CD.x.BCF_Dioum.AB485660     AG-TGGCGCC CGAACAGGGA CTTTAAA-GC GAAAGTTAAT AGGGACTCGA AA-GCGAAAG TT-------- ---------- ---------- ----------
06_cpx.EE.01.EE0359.AY535659       AG-TGGCGCC TGAACAGGGA CCAGAAA-AC GAAAGTTAAT AGGGACTCGA AA-GCGAAAG TT-------- ---------- ---------- ----------
06_cpx.GH.03.03GH173_06.AB2868     AG-TGGCGCC CGAACAGGGA CCCGAAA-GT GAAAGTTAAT AGGGACTCGA AA-GCGGAAG TT-------- ---------- ---------- ----------
06_cpx.ML.95.95ML127.AJ288982      AG-TGGCGCC CGAACAGGGA CTTGAAA-GC GAAAGTTAAT AGGGACTCGA AA-GCGAAAG TT-------- ---------- ---------- ----------
06_cpx.ML.95.95ML84.AJ245481       AG-TGGCGCC CGAACAGGGA CCTGAAA-GC GAAAGTTAAT AGGGACTCGA AA-GCGAAAG TT-------- ---------- ---------- ----------
06_cpx.SN.97.97SE1078.AJ288981     AG-TGGCGCC CGAACAGGGA CCCGAAA-GC GAAAGTTAAT AGGGACTCGA AA-GCGAAAG TT-------- ---------- ---------- ----------
07_BC.CN.05.XJDC6431_2.EF36837     ---------- ---------- ---------- ---------- ------GCGA AA-GCGAAAG TAGAG----- ---------- ---------- ----------
07_BC.CN.05.XJDC6441.EF368370      ---------- ---------- ---------- ---------- ------TTGA AA-GCGAAAG TAAGA----- ---------- ---------- ----------
07_BC.CN.06.Sichuan_2006_SC006     ---------- ---------- ---------- ---------- ---------- ---------G TAAGA----- ---------- ---------- ----------
07_BC.CN.06.Sichuan_2006_SC008     ---------- ---------- ---------- ---------- ---------- ---------G TAAGA----- ---------- ---------- ----------
07_BC.CN.06.Xinjiang_2006_709.     ---------- ---------- ---------- ---------- ---------- -----GAAAG TAAAG----- ---------- ---------- ----------
07_BC.CN.07.CNGZD.JQ423923         ---------- ---------- ---------- ---------- ------TTGA AA-GCGAAAG TAAGA----- ---------- ---------- ----------
07_BC.CN.98.98CN009.AF286230       ---------- ---------- ---------- ---------- ------TTGA AA-GCGAAAG TAAGA----- ---------- ---------- ----------
08_BC.CN.00.p00CH_HH090_08_BC3     AG-TGGCGCC CGAACAGGGA CTTGAAA-GC GAAAGT-AAG A--------- ---------- ---------- ---------- ---------- ----------
08_BC.CN.00.p00CH_WS035_08_BC5     AG-TGGCGCC CGAACAGGGA CCTGAAA-GC GAAAGT-GAG A--------- ---------- ---------- ---------- ---------- ----------
08_BC.CN.01.p01CH_DL001_08_BC0     AG-TGGCGCC CGAACAGGGA CTTGAAA-GC GAAAGT-AAG A--------- ---------- ---------- ---------- ---------- ----------
08_BC.CN.06.nx2.HM067748           AG-TGGCGCC CGAACAGGGG CAAGAAA-AG GAAAAT-GAG A--------- ---------- ---------- ---------- ---------- ----------
08_BC.CN.98.98CN006.AF286229       ---------- ---------- -TTGAAA-GC GAAAGT-AAG A--------- ---------- ---------- ---------- ---------- ----------
09_cpx.CI.00.00IC_10092.AJ8665     AG-TGGCGCC CGAACAGGGA CTTTAAA-GT GAAAGT-AAT AGGGACTCGA AA-GCGGAAG TT-------- ---------- ---------- ----------
10_CD.TZ.96.96TZ_BF061.AF28954     AG-TGGCGCC CGAACAGG-- ---------- ---------- --GTACCTGA AA-GCGAAAG TAGAA----- ---------- ---------- ----------
10_CD.TZ.96.96TZ_BF071.AF28954     AG-TGGCGCC CGAACAGG-- ---------- ---------- ---GACTTGA AA-GCGAAAG TAGAA----- ---------- ---------- ----------
10_CD.TZ.96.96TZ_BF110.AF28955     AG-TGGCGCC CGAACAGG-- ---------- ---------- ---GACTTGA AA-GCGAAAG TAGAA----- ---------- ---------- ----------
11_cpx.CM.95.95CM_1816.AF49262     AG-TGGCGCC CGAACAGG-- ---------- ---------- ---GACCCGA AA-GCGAAAG TAAGA----- ---------- ---------- ----------
11_cpx.CM.96.96CM_4496.AF49262     AG-TGGCGCC CGAACAGG-- ---------- ---------- ---GACTTGA AA-GCGAAAG TAAGA----- ---------- ---------- ----------
11_cpx.CM.97.MP818.AJ291718        AG-GGGGGCC CCAACAGG-- ---------- ---------- ---GACCCGA AA-GCCAAAG TTAGA----- ---------- ---------- ----------
11_cpx.FR.99.MP1298.AJ291719       AG-TGGCGCC CGAACAGG-- ---------- ---------- ---GACTTGA AA-GCGAAAG TAAGA----- ---------- ---------- ----------
11_cpx.FR.99.MP1307.AJ291720       GAAGGGCGCC CGAACCTG-- ---------- ---------- --GGACCCCG AA-GCGAAAG TAAGA----- ---------- ---------- ----------
11_cpx.GR.x.GR17.AF179368          ---------- ---------- ---------- ---------- ---------- ---------- ---------- ---------- ---------- ----------
12_BF.AR.97.A32879.AF408629        AG-TGGCGCC CGAACAGG-- ---------- ---------- ---GACCTGA AA-GCGAAAG TAGAA----- ---------- ---------- ----------
12_BF.AR.97.A32989.AF408630        AG-TGGCGCC CGAACAGG-- ---------- ---------- ---GACTTGA AA-GCGAAAG TAGAA----- ---------- ---------- ----------
12_BF.AR.99.ARMA159.AF385936       AG-TGGCGCC CGAACAGG-- ---------- ---------- ---GACTTGA AA-GCGAAAG TAGAA----- ---------- ---------- ----------
12_BF.UY.99.URTR23.AF385934        AG-TGGCGCC CGAACAGG-- ---------- ---------- ---GACTAGA AA-GCGAAAG TAGAA----- ---------- ---------- ----------
12_BF.UY.99.URTR35.AF385935        AG-TGGCGCC CGAACAGG-- ---------- ---------- ---GACCTGA AA-GCGAAAG TAAAA----- ---------- ---------- ----------
```

```
13_cpx.CM.02.02CM_A1394.DQ8453   AG-TGGCGCC CGAACAGG-- ---------- ---------- ---GACCAGG GA-AGCGAAA GTGAA----- ---------- ---------- ----------
13_cpx.CM.04.04CM_173_9.DQ8453   AG-TGGCGCC CGAACAGG-- ---------- ---------- ---GACTTGA AA-GTGAAAG CTAA------ ---------- ---------- ----------
13_cpx.CM.04.04CM_632_28.DQ845   AG-TGGCGCC CGAACAGG-- ---------- ---------- ---GACTTGA AG-CGAAAGC GAAAG----- ---------- ---------- ----------
13_cpx.CM.96.96CM_1849.AF46097   AG-TGGCGCC CGAACAGG-- ---------- ---------- ---GACGTGA AA-GCGAAAG CTAA------ ---------- ---------- ----------
13_cpx.CM.96.96CM_4164.AF46097   AG-TGGCGCC CGAACAGG-- ---------- ---------- ---GACTTAA AA-GCGAAAG CTAA------ ---------- ---------- ----------
14_BG.ES.00.X605.AF450096        AG-TGGCGCC CGAACAGGGA CTTGAAA-GC GAAAGTTAAC AGGGACTCGA AA-GCGAAAG TT-------- ---------- ---------- ----------
14_BG.ES.00.X623.AF450097        AG-TGGCGCC CGAACAGGGA CTTGAAA-GC GAAAGTTAAT AGGGACTCGA AA-GCGAAAG TT-------- ---------- ---------- ----------
14_BG.ES.05.X1870.FJ670522       AG-TGGCGCC CGAACAGG-- ---------- ---------- ---GACTTGA AA-GCGAAAG TTAACAGGGA CTCGAAAGCG AAAGTT---- ----------
14_BG.ES.05.X772_8.FJ670528      AG-TGGCGCC CGAACAGG-- ---------- ---------- ---GACTTGA AA-GCGAAAG TTAACAGGGA CTCGAAAGCG AAAGTT---- ----------
15_01B.TH.99.99TH_MU2079.AF516   AG-TGGCGCC CGAACAGGGA CCTTAAA-GT GAAAGTTAAT AGGGACTCGA AA-GCGAAAG TT-------- ---------- ---------- ----------
15_01B.TH.99.99TH_R2399.AF5305   ---------- ---------A CTTGAAA-GC GAAAGTTAAT AGGGACTCGA AA-GCGGAAG TT-------- ---------- ---------- ----------
16_A2D.KR.97.97KR004.AF286239    ---------- -----CGTAA AGTG------ -AAAG-TAAT AGGGACTTGA AA-GCGAAAG TT-------- ---------- ---------- ----------
18_cpx.CU.99.CU14.AY586541       ---------- ---------- ---------- ---------- ---------- ---------- ---------- ---------- ---------- ----------
18_cpx.CU.99.CU68.AY894993       ---------- ---------- ---------- ---------- ---------- ---------- ---------- ---------- ---------- ----------
18_cpx.CU.99.CU76.AY586540       ---------- ---------- ---------- ---------- ---------- ---------- ---------- ---------- ---------- ----------
19_cpx.CU.99.CU29.AY588971       AG-TGGCGCC CGAACAGGGA CTTAAAA-GC GAAAGTTAAT AGGGACTCGA AA-GCGAAAG TT-------- ---------- ---------- ----------
20_BG.CU.03.CB134.DQ020274       AG-TGGCGCC CGAACAGGGA CTTGAAA-GT GAAAGTTAAA AGGGACTCGA AA-GCGAAAG TT-------- ---------- ---------- ----------
20_BG.CU.99.Cu103.AY586545       AG-TGGCGCC CGAACAGGGA CTTGAAA-GT GAAAGTTAAA AGGGACTCGA AA-GCGAAAG TT-------- ---------- ---------- ----------
20_BG.ES.99.R77.AY586544         AG-TGGCGCC CGAACAGGGA CTTGAAA-GC GAAAGTTAAA AGGGACTCGA AA-GCGAAAG TT-------- ---------- ---------- ----------
22_01A1.CM.02.02CAMLT72.EU7439   ---------- ---------- ---------- ---------- ---------- ---------- ---------- ---------- ---------- ----------
22_01A1.CM.06.LPH27MF.JN864049   ---------- ---------- ---------- ---------- ---------- ---------- ---------- ---------- ---------- ----------
22_01A1.CM.10.LB054.JN864059     ---------- ---------- ---------- ---------- ---------- ---------- ---------- ---------- ---------- ----------
23_BG.CU.03.CB118.AY900571       AG-TGGCGCC CGAACAGGGA CTTGAAA-GC GAAAGTTAAA AGGGACTCGA AA-GCGGAAG TT-------- ---------- ---------- ----------
23_BG.CU.03.CB347.AY900572       AG-TGGCGCC CGAACAGGGA CTTGAAA-GT GAAAGTTAAA AGGGACTCGA AA-GCGGAAG TT-------- ---------- ---------- ----------
24_BG.CU.03.CB378.AY900574       AG-TGGCGCC CGAACAGGGA CTTGAAA-GT GAAAGTTAAT AGGGACTCGA AA-GCGAAAG TT-------- ---------- ---------- ----------
24_BG.CU.03.CB471.AY900575       AG-TGGCGCC CGAACAGGGA CCTGAAA-GT GAAAGTTAAT AGGGACTCGA AA-GCGAAAG TT-------- ---------- ---------- ----------
24_BG.ES.08.X2456_2.FJ670526     AG-TGGCGCC CGAACAGG-- ---------- ---------- ---GACTTGA AA-GCGAAAG TTAATAGGGA CTCGAAAGCG AAAGTT---- ----------
25_cpx.CM.06.06CM_BA_040.EU693   AG-TGGCGCC CGAACAGGGA CCTGAAA-AA GAAAGTTAAT AGGGACTCGA AA-GCGAAAG TT-------- ---------- ---------- ----------
25_cpx.SA.03.J11233.EU697906     AG-TGGCGCC CGAACAGGGA CCCGAAA-GY GAAAGTTAAT AGGGACTCGA AA-GCGGAAG TT-------- ---------- ---------- ----------
25_cpx.SA.03.J11451.EU697908     AG-TGGCGCC CGAACAGGGA CTTGAAA-GT GAAAGTTAAT AGGGACTCGA AA-GCGAAAG TT-------- ---------- ---------- ----------
26_AU.CD.02.02CD_KS069.FM87778   AG-GGGCGCC CAACCAGGGC CTTTAAA-GC GAAAGT-AAA AGGGACTCGA AA-GCGGAAG TT-------- ---------- ---------- ----------
26_AU.CD.02.02CD_LBTB084.FM877   AG-TGGCGCT CGAACAGGGA CCTTAAA-GC GAAAGT-AAA AGGGACTCGA AA-GCGGAAG TT-------- ---------- ---------- ----------
26_AU.CD.02.02CD_MBTB047.FM877   AG-TGGCGCC CGAACAGGGA CCTTAAA-GC GAAAGT-AAA AGGGACTCGA AA-GCGGAAG TT-------- ---------- ---------- ----------
26_AU.CD.97.97CD_KTB119.FM8777   AG-TGGCGCC CGAACAGGGA CTTGAAA-GC GAAAG-TAAA AGGGACTCGA AA-GCGGAAG TT-------- ---------- ---------- ----------
27_cpx.FR.04.04CD_FR_KZS.AM851   AG-TGGCGCC CGAACAGG-- ---------- ---------- ---GA-CTTG AA-AGCGAAA GTAA------ ---------- ---------- ----------
28_BF.BR.05.0679SV.JF804812      ---------- ---------- ---------- ---------- -------AGA AA-GCGAAAG TAAAA----- ---------- ---------- ----------
28_BF.BR.99.BREPM12313.DQ08587   AG-TGGCGCC CGAACAGG-- ---------- ---------- ---GACTTGA AA-GCGAAAG TAGAA----- ---------- ---------- ----------
28_BF.BR.99.BREPM12609.DQ08587   AG-TGGCGCC CGAACAGG-- ---------- ---------- ---GACTTGA AA-GCGAAAG TAGAA----- ---------- ---------- ----------
28_BF.BR.99.BREPM12817.DQ08587   AG-TGGCGCC CGAACAGG-- ---------- ---------- ---GACC-GA AA-GCGAAAG TAGAA----- ---------- ---------- ----------
29_BF.BR.01.BREPM16704.DQ08587   AG-TGGCGCC CGAACAGG-- ---------- ---------- ---GACTTGT GA-GCGAAAG TTGAA----- ---------- ---------- ----------
29_BF.BR.05.0264RI.JF804807      ---------- ---------- ---------- ---------- ---------- ---------- ---------- ---------- ---------- ----------
29_BF.BR.99.BREPM11948.DQ08587   AG-TGGCGCC CGAACAGG-- ---------- ---------- ---GACGCGA AA-GCGAAAG TAGAA----- ---------- ---------- ----------
31_BC.BR.02.110PA.EF091932       AG-TGGCGCC CGAACAGG-- ---------- ---------- ---GACTCGA AA-GCGAAAG TAAGA----- ---------- ---------- ----------
31_BC.BR.04.04BR142.AY727527     AG-TGGCGCC CGAACAGG-- ---------- ---------- ---GACGCGA AA-GCGAAAG TGAGA----- ---------- ---------- ----------
32_06A1.EE.01.EE0369.AY535660    AG-TGGCGCC CGAACAGGGA CCTGAAA-AC GAAAGTTAAT AGGGACTCGA AA-GCGAAAG TT-------- ---------- ---------- ----------
32_06A1.EE.02.EST2002_1169.DQ1   AG-TGGCGCC CGAACAGGGA CTTGAAA-AC GAAAGTTAAT AGGGACTCGA AA-GCGAAAG TT-------- ---------- ---------- ----------
33_01B.ID.07.JKT189_C.AB547463   ---------- ---------- ---------- ---------- ---------- ---------- ---------- ---------- ---------- ----------
33_01B.MY.05.05MYKL007_1.DQ366   AG-TGGCGCC CGAACAGGGA CTTGAAA-AC GAAAGTTAAT AGGGACTCGA AA-GCGAAAG TT-------- ---------- ---------- ----------
33_01B.MY.05.05MYKL015_2.DQ366   AG-TGGCGCC CGAACAGGGA CTTGAAA-GC GAAAGTTAAT AGGGACTCGA AA-GCGAAAG TT-------- ---------- ---------- ----------
33_01B.MY.05.05MYKL031_1.DQ366   AG-TGGCGCC CGAACAGGGA CCTTAAA-GA GGAAGTTAAT AGGGACTCGA AA-GCGAAAG TT-------- ---------- ---------- ----------
33_01B.MY.05.05MYKL045_1.DQ366   AG-TGGCGCC CGAACAGGGA CTTGAAA-GC GAAAGTTAAT AGGGACTCGA AA-GCGAAAG TT-------- ---------- ---------- ----------
38_BF1.UY.03.UY03_3389.FJ21378   AG-TGGCGCC CGAACAGG-- ---------- ---------- ---GACCTGA AA-GCGAAAG TAGAA----- ---------- ---------- ----------
38_BF1.UY.04.UY04_3987.FJ21378   AG-TGGCGCC CGAACAGG-- ---------- ---------- ---GACGCGA AA-GCGAAAG TAGAA----- ---------- ---------- ----------
38_BF1.UY.04.UY04_4022.FJ21378   AG-TGGCGCC CGAACAGG-- ---------- ---------- ---GACCTGA AA-GCGAAAG TAGAA----- ---------- ---------- ----------
38_BF1.UY.05.UY05_4752.FJ21378   AG-TGGCGCC CGAACAGG-- ---------- ---------- ---GACTTGA AA-GCGAAAG TAGAA----- ---------- ---------- ----------
39_BF.BR.03.03BRRJ103.EU735534   AG-TGGCGCC CGAACAGG-- ---------- ---------- ---GACCCGA AA-GCGAAAG TAGAA----- ---------- ---------- ----------
39_BF.BR.03.03BRRJ327.EU735536   AG-TGGCGCC CGAACAGG-- ---------- ---------- ---GACCTGA AA-GTGAAAG TAGAA----- ---------- ---------- ----------
39_BF.BR.04.04BRRJ179.EU735535   AG-TGGCGCC CGAACAGG-- ---------- ---------- ---GACCTGA AA-GCGAAAG TAGAA----- ---------- ---------- ----------
40_BF.BR.04.04BRRJ115.EU735538   AG-TGGCGCC CGAACAGG-- ---------- ---------- ---GACGCGA AA-GCGAAAG TAGAA----- ---------- ---------- ----------
40_BF.BR.04.04BRSQ46.EU735540    AG-TGGCGCC CGAACAGG-- ---------- ---------- ---GACTTGA AA-GCGAAAG TAGAA----- ---------- ---------- ----------
40_BF.BR.05.05BRRJ055.EU735537   AG-TGGCGCC CGAACAGG-- ---------- ---------- ---GACCTGA AA-GCGAAAG TAGAA----- ---------- ---------- ----------
40_BF.BR.05.05BRRJ200.EU735539   AG-TGGCGCC CGAACAGG-- ---------- ---------- ---GACCTGA AA-GCGAAAG TAGAA----- ---------- ---------- ----------
42_BF.LU.03.luBF_01_03.EU17015   AG-TGGCGCC CGAACAGG-- ---------- ---------- ---GACYTGA AA-GCGAAAG TAGAA----- ---------- ---------- ----------
42_BF.LU.03.luBF_02_03.EU17015   AG-TGGCGCC CGAACAGG-- ---------- ---------- ---GACCTGA AA-GCGAAAG TAGAA----- ---------- ---------- ----------
42_BF.LU.03.luBF_04_03.EU17014   AG-TGGCGCC CGAACAGG-- ---------- ---------- ---GACCTGA AA-GCGAAAG TAGAA----- ---------- ---------- ----------
42_BF.LU.03.luBF_05_03.EU17015   AG-TGGCGCC CGAACAGG-- ---------- ---------- ---GACYTGA AA-GCGAAAG TAGAA----- ---------- ---------- ----------
42_BF.LU.03.luBF_06_03.EU17014   AG-TGGCGCC CGAACAGG-- ---------- ---------- ---GACTTGA AA-GCGAAAG TAGAA----- ---------- ---------- ----------
42_BF.LU.03.luBF_07_03.EU17014   AG-TGGCGCC CGAACAGG-- ---------- ---------- ---GACCYGA AA-GCGAAAG TAGAA----- ---------- ---------- ----------
42_BF.LU.03.luBF_08_03.EU17014   AG-TGGCGCC CGAACAGG-- ---------- ---------- ---GACCTGA AA-GCGAAAG TAGAA----- ---------- ---------- ----------
42_BF.LU.03.luBF_09_03.EU17015   AG-TGGCGCC CGAACAGG-- ---------- ---------- ---GACYTGA AA-GCGAAAG TAGAA----- ---------- ---------- ----------
42_BF.LU.04.luBF_11_04.EU17014   AG-TGGCGCC CGAACAGG-- ---------- ---------- ---GACYTGA AA-GCGAAAG TAGAA----- ---------- ---------- ----------
42_BF.LU.04.luBF_12_04.EU17014   AG-TGGCGCC CGAACAGG-- ---------- ---------- ---GACCCGA AA-GCGAAAG TAGAA----- ---------- ---------- ----------
42_BF.LU.05.luBF_13_05.EU17013   AG-TGGCGCC CGAACAGG-- ---------- ---------- ---GACYTGA AA-GCGAAAG TAGAA----- ---------- ---------- ----------
42_BF.LU.05.luBF_14_05.EU17014   AG-TGGCGCC CGAACAGG-- ---------- ---------- ---GACTTGA AA-GCGAAAG TAGAA----- ---------- ---------- ----------
42_BF.LU.05.luBF_16_05.EU17015   AG-TGGCGCC CGAACAGG-- ---------- ---------- ---GACTTGA AA-GCGAAAG TAGAA----- ---------- ---------- ----------
42_BF.LU.05.luBF_17_05.EU17014   AG-TGGCGCC CGAACAGG-- ---------- ---------- ---GACYTGA AA-GCGAAAG TAGAA----- ---------- ---------- ----------
42_BF.LU.06.luBF_18_06.EU17013   AG-TGGCGCC CGAACAGG-- ---------- ---------- ---GACCCGA AA-GCGAAAG TAGAA----- ---------- ---------- ----------
42_BF.LU.06.luBF_19_06.EU17013   AG-TGGCGCC CGAACAGG-- ---------- ---------- ---GACCCGA AA-GCGAAAG TAGAA----- ---------- ---------- ----------
43_02G.SA.03.J11223.EU697904     AG-TGGCGCC CGAACAGGGA CTTGAAA-GT GAAAGTTAAT AGGGACTCGA AA-GCGRAAG TT-------- ---------- ---------- ----------
43_02G.SA.03.J11232.EU697905     AG-TGGCGCC CGAACAGGGA CTTGAAA-AG CGAAAGTAAT AGGGACTCGA AA-GCGGAAG TT-------- ---------- ---------- ----------
43_02G.SA.03.J11243.EU697907     AG-TGGCGCC CGAACAGGGA CTTGAAA-GT GAAAGTTAAT AGGGACTCGA AA-GCGCAAG TT-------- ---------- ---------- ----------
43_02G.SA.03.J11456.EU697909     AG-TGGCGCC CGAACAGGGA CTTGAAA-GT GAAAGTTAAT AGGGACTCGA AA-GCGAAAG TT-------- ---------- ---------- ----------
44_BF.CL.00.CH80.FJ358521        AG-TGGCGCC CGAACAGG-- ---------- ---------- ---GACTTGA AA-GCGAAAG TAGAA----- ---------- ---------- ----------
44_BF.CL.01.CH12.AY536235        AG-TGGCGCC CGAACAGG-- ---------- ---------- ---GACTTGA AA-RCGAAAG TAAAA----- ---------- ---------- ----------
45_cpx.CD.97.97CD_MBFE185.FN39   AG-TGGCGCC CGAACAGG-- ---------- ---------- ---GACTCGA AA-GCGAAAG TT-------- ---------- ---------- ----------
45_cpx.CD.97.97CD_MBS30.FN3928   AG-TGGCGCC CGAACAGGGA CTTGAAA-AC GAAAG----- ---------- ------TAAG TT-------- ---------- ---------- ----------
45_cpx.CM.97.97CM_MP814.FN3928   AG-TGGCGCC CGAACAGGGA CTTG------ ---------- ---------A AA-GCGAAAG TAAAAGTT-- ---------- ---------- ----------
45_cpx.FR.04.04FR_AUK.EU448295   AG-TGGCGCC CGAACAGG-- ---------- ---------- ---GACTCGA AA-GCGAAAG TT-------- ---------- ---------- ----------
45_cpx.GA.97.97GA_TB45.FN39287   AG-TGGCGCC CGAACAGG-- ---------- ---------- ---GACCTGA AA-GCGAAAG TA-------- ---------- ---------- ----------
46_BF.BR.01.01BR087.DQ358801     AG-TGGCGCC CGAACAGG-- ---------- ---------- ---GACTTGA AA-GCGAAAG TAGAA----- ---------- ---------- ----------
46_BF.BR.01.01BR125.DQ358802     AG-TGGCGCC CGAACAGG-- ---------- ---------- ---GACCTGA AA-GCGAAAG TAGAA----- ---------- ---------- ----------
46_BF.BR.07.07BR_FPS625.HM0264   AG-TGGCGCC CGAACAGG-- ---------- ---------- ---GACTTGA AA-GCGAAAG TAGAA----- ---------- ---------- ----------
46_BF.BR.07.07BR_FPS742.HM0264   AG-TGGCGCC CGAACAGG-- ---------- ---------- ---GACTTGA AA-GCGAAAG TAGAA----- ---------- ---------- ----------
46_BF.BR.07.07BR_FPS783.HM0264   AG-TGGCGCC CGAACAGG-- ---------- ---------- ---GACGCGA AA-GCGAAAG TAGAA----- ---------- ---------- ----------
46_BF.BR.07.07BR_FPS810.HM0264   AG-TGGCGCC CGAACAGG-- ---------- ---------- ---GACCCGA AA-GCGAAAG TAGAA----- ---------- ---------- ----------
46_BF.BR.07.07BR_FPS812.HM0264   AG-TGGCGCC CGAACAGG-- ---------- ---------- ---GACGCGA AA-GCGAAAG TAGAA----- ---------- ---------- ----------
47_BF.ES.08.P1942.GQ372987       AG-TGGCGCC CGAACAGG-- ---------- ---------- ---GACCCGA AA-GCGAAAG TAGAA----- ---------- ---------- ----------
47_BF.ES.08.X2457_2.FJ670529     AG-TGGCGCC CGAACAGG-- ---------- ---------- ---GACCCGA AA-GCGAAAG TAGAA----- ---------- ---------- ----------
49_cpx.GM.02.N18380.HQ385477     AG-TGGCGCC CGAACAGG-- ---------- ---------- ---GACTTGA AA-GCGAAAG TAAGA----- ---------- ---------- ----------
49_cpx.GM.03.N26677.HQ385479     AG-TGGCGCC CGAACAGG-- ---------- ---------- ---GACTCGA AA-ACGAAAG TAAGG----- ---------- ---------- ----------
49_cpx.GM.97.N28353.HQ385478     AG-TGGCGCC CGAACAGG-- ---------- ---------- ---GACTTGA AA-GCGAAAG TAAGG----- ---------- ---------- ----------
52_01B.MY.03.03MYKL018_1.DQ366   ---------- ---------- ---------- ---------- ---------- ---------- ---------- ---------- ---------- ----------
53_01B.MY.04.04MYKL016_1.DQ366   ---------- ---------- ---------- ---------- ---------- ---------- ---------- ---------- ---------- ----------
53_01B.MY.10.10MYKJ067.JX39061   ---------- ---------- ---------- --------AT AGGGACTCGA AA-GCGAAAG TT-------- ---------- ---------- ----------
53_01B.MY.10.10MYKJ079.JX39061   ---------- ---------- ---------- --------AT AGGGACTCGA AA-GCGAAAG TT-------- ---------- ---------- ----------
```

```
53_01B.MY.11.11FIR164.JX390610    ---------- ---------- ---------- ---------- -GGGACTCGA AA-GCGRAAG TT-------- ---------- ---------- ----------
54_01B.MY.07.07MYKLD49.EU03191    AG-TGGCGCC CGAACAGGGA CTTGAAA-GT GAAAGTTAAT AGGGACTCGA AA-GCGAAAG TT-------- ---------- ---------- ----------
54_01B.MY.08.08MYKL044.JX39097    ---------- ---------- ---------- ---------- ----ACTCGA AA-GCGGAAG TT-------- ---------- ---------- ----------
54_01B.MY.09.09MYSB023.JX39097    AG-TGGCGCC CGAACAGGGA CTTGAAA-AT AAAAGTTATT AGGGACTCGA AA-GCGGAAG TT-------- ---------- ---------- ----------
55_01B.CN.10.HNCS102056.JX5746    ---------- ---------- ---------- ---------- ---------- ---------- ---------- ---------- ---------- ----------
55_01B.CN.11.GDDG318.JX574662     ---------- ---------- ---------- ---------- ---------- ---------- ---------- ---------- ---------- ----------
01A1.MM.99.mCSW105.AB097872       AG-TGGCGCC CGAACAGGGA CTCGAAA-GC GGAAGTTAAT AGGGACTCGA AA-GCG-AAG TT-------- ---------- ---------- ----------
01B.CN.08.08CYM047.JF340054       ---------A GTTATAGG-- ---------- ---------- ---GACTCG- ---AAAGCGA AAGTT----- ---------- ---------- ----------
01B.JP.x.pHIV_1_Y271B01AE64.AB    AG-TGGCGCC CGAACAGG-- ---------- ---------- ---GACTGGA AAAGCGAAAG TAAAA----- ---------- ---------- ----------
01B.MM.00.mIDU502.AB097865        AG-TGGCGCC CGAACAGG-- ---------- ---------- --GCACGCGA AA-ACGAAAG TGAGA----- ---------- ---------- ----------
01B.MM.99.mCSW104.AB097867        AG-TGGCGCC CGAACAGGGA CTTGAAA-GC GAAAGTTAAT AGGGACTCGA AA-GCGAAAG TT-------- ---------- ---------- ----------
01B.MY.04.04MYKL019_1.DQ366665    ---------- ---------- ---------- ---------- ---------- ---------- ---------- ---------- ---------- ----------
01B.MY.05.05MYKL043_1.DQ366666    AG-TGGCGCC CGAACAGGGA CTTGAAG-GC GGAAGTTAAT AGGGACTCGA AA-GCGAAAG TT-------- ---------- ---------- ----------
01B.MY.06.06MMYKLD46.EF495062     AG-TGGCGCC CGAACAGGGA CTTGAAA-GC GAAAGTTAAT AGGGACTCGA AA-GCGAAAG TT-------- ---------- ---------- ----------
01B.MY.07.07MYKLD47.EU031913      ---------- ---------- ---------- ---------- ---------- ---------- ---------- ---------- ---------- ----------
01B.MY.07.07MYKLD48.EU031914      AG-TGGCGCC CGAACAGGGA CTTGAAA-GC GAAAGTTAAT AGGGACTCGA AA-GCGGAAG TT-------- ---------- ---------- ----------
01B.TH.05.05TH140456.JN631793     AG-TGGCGCC CGAACAGGGA CTTGAAA-GC GAAAGTTAAT AGGGACTCGA AA-GCGAAAG TT-------- ---------- ---------- ----------
01B.TH.05.AA095a_WG21.JX447830    AG-TGGCGCC CGAACAGGGA CTTGAAA-GC GAAAGTTAAT AGGGACTCGA AA-GCGAAAG TT-------- ---------- ---------- ----------
01B.TH.06.AA020a_wg2.JX446927     ---------- --------GA CTTTAAA-GT GAAAGTTAAT AGGGACTCGA AA-GCGAAAG TT-------- ---------- ---------- ----------
01B.TH.06.AA025a_WG13.JX447000    AG-TGGCGCC CGAACAGGGA CTTGAAA-GC GAAAGTTAAT AGGGACTCGA AA-GCGAAAG TT-------- ---------- ---------- ----------
01B.TH.06.AA084a_WG10.JX447668    AG-TGGCGCC CGAACAGGGA CTTGAAA-GC GAAAGTTAAA AGGGACTCGA AA-GCGGAAG TT-------- ---------- ---------- ----------
01B.TH.91.CM237.AY167123          AG-TGGCGCC CGAACAGG-- ---------- ---------- ---GACTTGA AA-GCGAAAG TAGAA----- ---------- ---------- ----------
01BC.MM.00.mCSW503.AB097866       ---------- ---------- ---------- ---------- ---------- ---------- ---------- ---------- ---------- ----------
01BC.MM.99.mIDU107.AB097868       AG-TGGCGCC CGAACAGGGA CTTGAAA-GC GAAAGT-AAT AGGGACTCGA AA-GCGAAAG TT-------- ---------- ---------- ----------
01F2G.CM.02.LT31.JN864056         ---------- ---------- ---------- ---------- ---------- ---------- ---------- ---------- ---------- ----------
0206.DZ.10.DEURF10DZ001.JX1406    ---------- ---------- ---------- ---------- ---------- ---------- ---------- ---------- ---------- ----------
0206.GH.03.03GH195AG_06.AB2868    AG-TGGCGCC CGAACAGGGA CTTGGAG--- -----TTAAT AGGGACTCGA AA-GCGAAAG TG-------- ---------- ---------- ----------
0206.NE.00.NE36.AJ508597          AG-TGGCGCC CGAACAGGGA CTTG------ -AAAGTTAAT AGGGACTCGA AA-GCGAAAG TT-------- ---------- ---------- ----------
0206.NE.00.NE95.AJ508596          AG-TGGCGCC CGAACAGGGA CTCGAAA-GC GAAAGTTAAT AGGGACTCGA AA-ACGAAAG TT-------- ---------- ---------- ----------
0206.NE.97.NE03.AJ508595          AG-TGGCGCC CGAACAGGGA CTTGAAA-GC GAAAGTTAAT AGGGACTCGA AA-GCGAAAG TT-------- ---------- ---------- ----------
0209.CI.01.01IC_17395.AJ866554    AG-TGGCGCC CGAACAGGGA CTTGAAA-GC GAAAGTTAAT AGGGACTCGA AA-GCGAAAG TT-------- ---------- ---------- ----------
0209.CI.01.01IC_PCI118.AJ86655    AG-TGGCGCC CGAACAGGGA CTTGAAA-GC GAAAGTTAAT AGGGACTCGA AA-GCGAAAG TT-------- ---------- ---------- ----------
0209.CI.01.01IC_PCI127.AJ86655    AG-TGGCGCC CGAACAGGGA CTTGAAA-GC GAAAGTTAAT AGGGACTCGA AA-GCGAAAG TT-------- ---------- ---------- ----------
0209.CI.97.97IC_PCI3.AJ866555     AG-TGGCGCC CGAACAGGGA CTTGAAA-GC GAAAGTTAAT AGGGACTCGA AA-GCGAAAG TT-------- ---------- ---------- ----------
0222.CM.02.02CAMLT04.EU743964     ---------- ---------- ---------- ---------- ---------- ---------- ---------- ---------- ---------- ----------
0222.CM.08.BDSH129.JN864052       ---------- ---------- ---------- ---------- ---------- ---------- ---------- ---------- ---------- ----------
0222.CM.10.LB045.JN864053         -------CCG CAACCTCA-- ---------- ---------- ---ACCTCGA AA-GCGAAAG TT-------- ---------- ---------- ----------
02A1.ES.05.X230_10.FJ670515       AG-TGGCGCC CGAACAGGGA C--------C GGAAGTTAAT AGGGACTCGA AA-ACGAAAG TT-------- ---------- ---------- ----------
02A1.ES.07.ES_P1751.GQ372986      AG-TGGCGCC CGAACAGG-- ---------- ---------- ---GACTTGA AA-GCGAAAG TT-------- ---------- ---------- ----------
02A1.GH.03.GHNJ176.AB231894       AG-TGGCGCC CGAACAGGGA CCGG------ --AAGTTAAT AGGGACTCGA AA-GCGAAAG TT-------- ---------- ---------- ----------
02A1.GH.97.97GH_AG2.AB052867      AG-TGGCGCC CGAACAGGGA CCGG------ --AAGTTAAT AGGGGCTCGA AA-GCGAAAG TT-------- ---------- ---------- ----------
02A1.RU.10.10RU6637.JN230353      AG-TGGCGCC CGAACAGGGA CCGG------ --AAGTTAAT AGGGACTCGA AA-GCGAAAG TT-------- ---------- ---------- ----------
02B.ES.99.99SP_11339.DQ926899     AG-TGGCGCC CGAACAGGGA CCGGAAA-TA TAGG------ ---GACTCGA AA-GCGAAAG TT-------- ---------- ---------- ----------
02B.FR.02.URF4.JN882652           ---------- ---------- ---------- ---------- -GGGACTCGA AA-GCGAAAG TT-------- ---------- ---------- ----------
02B.FR.09.URF2.JN882654           ---------- ---------- ---------- ---------- ---------- -------AAG AGAAA----- ---------- ---------- ----------
02BD.FR.08.URF1.JN882653          AG-TGGCGCC CGAACAGGGA CTTGAAA-GC GAAAGTTAAT AGGGACTCGA AA-GCGAAAG TT-------- ---------- ---------- ----------
02BG.FR.10.URF5.JN882655          ---------- ---------- ---------- ------TAAA AGGGACTCGA AA-GCGAAAG TT-------- ---------- ---------- ----------
02C.BE.93.VI1035.AJ276595         ---------- ---------- ---------- ---------- ---------- ---------- ---------- ---------- ---------- ----------
02D.GH.03.GHNJ193.AB231897        AG-TGGCGCC CGAACAGGGA CTTG------ --AAGTTAAT AGGGACTCGA AA-GCGAAAG TT-------- ---------- ---------- ----------
02D.GH.03.p03GH194AG09.AB48004    AG-TGGCGCC CGAACAGGGA CTTG------ -AAAGT-AAT AGGGACTCGA AA-GCGAAAG TT-------- ---------- ---------- ----------
02F2G.CM.02.LT66.JN864057         ---------- ---------- ---------- ---------- ---------- ---------- ---------- ---------- ---------- ----------
02GK.CI.01.01IC_PCI123.AJ86655    AG-TGGCGCC CGAACAGGGA CTCGAAA-GC GAAAGTTAAT AGGGACTCGA AA-GCGGAAG TT-------- ---------- ---------- ----------
06A1.BJ.x.B76.AJ293865            ---------- ---------- ---------- --------AT AGGGACTCGA AA-GCGAAAG TT-------- ---------- ---------- ----------
1819.CU.99.CU64.AY894995          AG-TGGCGCC CGAACAGG-- ---------- ---------- ---GACTTGA AA-GCGAAAG TAGTT----- ---------- ---------- ----------
26C.CD.02.02CD_LBTB032.FM87777    AG-TGGCGCC CGAACAGGGA CCTGAAA-AG CGAAAGTAAA AGGGACTCGA AA-GCGGCGG TT-------- ---------- ---------- ----------
26C.CD.97.97CD_KFE267.FM877778    AG-TGGCGCC CGAACAGGGA CTTGAAA-GC GAAAGTAAA- AGGGACTCGA AA-GCGGAAG TT-------- ---------- ---------- ----------
26C.CD.97.MBFE250.FM877783        AG-TGGCGCC CGAACAGGGA CTTGAAAAGC GAAAGT-AAA AGGGACTCGA AA-GCGGAAG TT-------- ---------- ---------- ----------
A1B.EE.02.EST2002_394.DQ167216    AG-TGGCGCC CGAACAGG-- ---------- ---------- ---GACTTGA AA-GCGAAAG TT-------- ---------- ---------- ----------
A1BD.GB.03.34567.JN417238         AG-TGGCGCC CGAACAGG-- ---------- ---------- ---GACTCGA AA-GCGAAAG TAGAA----- ---------- ---------- ----------
A1C.CA.04.04CA7750.EU220698       AG-TGGCGCC CGAACAGG-- ---------- ---------- ---GACTCGA AA-GCGAAAG TGTT------ ---------- ---------- ----------
A1C.IN.01.1579A.DQ083238          AG-TGGCGCC CGAACAGGGA CTTGAAAAGC GAAAG-TAAT AGGGACTCGA AA-GCGAAAG TT-------- ---------- ---------- ----------
A1C.IN.95.95IN21301.AF067156      AG-TGGCGCC CGAACAGG-- ---------- ---------- ---GACTTGA AA-GCGAAAG TAAGA----- ---------- ---------- ----------
A1C.RW.92.92RW009_06.U88823       ---------- ---------- ---------- ---------- ------ATGA AA-GCGAAAG TAAGA----- ---------- ---------- ----------
A1CD.KE.02.ML2000.EU110093        ---------- ---------- ---------- ---------- ---------- ---------- -T-------- ---------- ---------- ----------
A1CD.SE.95.SE8603.AF075702        ---------- ---------- ---------- ---------- ---------- ---------- ---------- ---------- ---------- ----------
A1CDGKU.ZA.99.CM4_99ZACM4.AF41    AG-TGGCGCC CGAACAGG-- ---------- ---------- ---GACTTGA AA-ACGAAAG TAAGA----- ---------- ---------- ----------
A1CG.KE.02.ML1979PCR.EU110096     ---------- ---------- ---------- ---------- ---------- ---------- ---------- ---------- ---------- ----------
A1D.DK.96.FSA.DQ912822            ---------- ---------- ---------- ---------- --GGACTTGA AA-GCGAAAG TAGAA----- ---------- ---------- ----------
A1D.DK.98.MA.DQ912823             AG-TGGCGCC CGAACAGG-- ---------- ---------- ---GACTTGA AA-GCGAAAG TAGAA----- ---------- ---------- ----------
A1D.GB.00.8179.JN417236           AG-TGGCGCC CGAACAGG-- ---------- ---------- ---GACTTGA AA-GCGAAAG TAGAA----- ---------- ---------- ----------
A1D.GB.03.33365.JN417239          AG-GGGCGCC CGAACAGG-- ---------- ---------- ---GACCTGA AA-GCGAAAG TAGAA----- ---------- ---------- ----------
A1D.GB.10.11762.JN417241          AG-TGGCGCC CGAACAGG-- ---------- ---------- ---GACTCGA AA-GCGAAAG TAGAA----- ---------- ---------- ----------
A1D.GB.10.12792.JN417240          AG-TGGCGCC CGAACAGG-- ---------- ---------- ---GACTCGA AA-GCGAAAG TAGAA----- ---------- ---------- ----------
A1D.KE.02.ML1974.EU110090         ---------- ---------- ---------- ---------- ---------- ---------- -A-------- ---------- ---------- ----------
A1D.SE.93.SE6954.AF075701         ---------- ---------- ---------- ---------- ---------- ---------- ---------- ---------- ---------- ----------
A1D.TZ.96.TZBFL0011.AF442569      -G-TGGCGCC CGAACAGG-- ---------- ---------- ---GACTTGA GA-GCGAAAG TAGAA----- ---------- ---------- ----------
A1D.TZ.96.TZBFL0088.AF442570      -G-TGGCGCC CGAACAGG-- ---------- ---------- ---GACTTGA AA-GCGAAAG TT-------- ---------- ---------- ----------
A1D.TZ.97.TZBFL0086.AF442566      -G-TGGCGCC CGAACAGG-- ---------- ---------- ---GACTTGA AA-GCGAAAG TAGAA----- ---------- ---------- ----------
A1D.UG.07.p191947.JX236674        AG-TGGCGCC CGAACAGGGA CCTGAAA-GC GAAAGTTAAC AGGGACTCGA AA-ACGAAAG TT-------- ---------- ---------- ----------
A1D.UG.08.p191982.JX236675        AG-TGGCGCC CGAACAGG-- ---------- ---------- ---GACTTGA AA-GCGAAAG TAAAA----- ---------- ---------- ----------
A1D.UG.90.UG266.AY352657          -G-TGGCGCC CGAACAGG-- ---------- ---------- ---GACTCGA AA-GCGAAAG TT-------- ---------- ---------- ----------
A1D.UG.92.UG035.AY352656          -G-TGGCGCC CGAACAGG-- ---------- ---------- ---GACTCGA AA-GCGAAAG TT-------- ---------- ---------- ----------
A1DHK.NO.97.97NOGIL3.AJ237565     AG-TGGCGCC CGAACAGG-- ---------- ---------- ---GACCTGA AA-GCGAAAG TGGAA----- ---------- ---------- ----------
A1DK.CD.85.MAL_MALCG.X04415       AG-TGGCGCC CGAACAGGGA CTTTAAA-GT GAAAGT-AAC AGGGACTCGA AA-GCGGAAG TT-------- ---------- ---------- ----------
A1G.BE.94.VI1197.AJ276596         ---------- ---------- ---------- ---------- ---------- ---------- ---------- ---------- ---------- ----------
A1G.NG.92.92NG003.U88825          ---------- -----TTGAA AGCG------ -AAAGTTAAC AGGGACTCGA AA-GCGAAAG TT-------- ---------- ---------- ----------
A1G.RU.11.11RU6900.JX500697       AG-TGGCGCC CGAACAGGGA CCGG------ --AAGTTAAT AGGGACTCGA AA-GCGAAAG TT-------- ---------- ---------- ----------
A1G.RU.11.11RU6939.JX500706       AG-TGGCGCC CGAACAGGGA CCTG------ --AAGTTAAT AGGGACTCGA AA-GCGAAAG TT-------- ---------- ---------- ----------
A1GHU.GA.x.VI354.AF076474         ---------- -------GGA CTTGAAA-GC GAAAG-TAAC AGGGACTCGA AA-GCGAAAG TT-------- ---------- ---------- ----------
A1GJ.BW.98.BW2117.AF192135        AG-TGGCGCC CGAACAGG-- ---------- ---------- ---GACTCGA AA-GCGAAAG TAAGA----- ---------- ---------- ----------
A2C.ZA.98.DU178_98ZADU178.AF41    AG-TGGCGCC CGAACAGG-- ---------- ---------- ---GACCTGA AA-GCGAAAG TAAGA----- ---------- ---------- ----------
A2C.ZM.90.ZAM184.U86780           AG-TGGCGCC CGAACAGG-- ---------- ---------- ---GACTTGA AA-GCGAAAG TAAGA----- ---------- ---------- ----------
A2CD.KE.01.ML1956.EU110089        ---------- ---------- ---------- ---------- ---------- ---------- -A-------- ---------- ---------- ----------
AC.KE.97.ML672.AY322191           AG-TGGCGCC CGAACAGGGA CTTGAAA-GC GAAAGTAAAT AGGGACTTGA AA-GCGAAAG TT-------- ---------- ---------- ----------
AC.ZA.04.04ZAPS204B1.DQ093606     AG-TGGCGCC CGAACAGG-- ---------- ---------- ---GACCCGA AA-GCGAAAG TAAGA----- ---------- ---------- ----------
AGKU.CD.x.BCF_Kita.AB485665       AG-TGGCGCC CGAACAGG-- ---------- ---------- ---GACTCGA AA-GCGAAAG TAA------- ---------- ---------- ----------
BC.AR.01.ARE195FL.AY968312        ---------- ---------- ---------- ---------- ---------- ---------- ---------- ---------- ---------- ----------
BC.BR.01.01_BR_RGS45.GQ365651     AG-TGGCGCC CGAACAGG-- ---------- ---------- ---GACCTGA AA-GCGAAAG TGAGA----- ---------- ---------- ----------
BC.BR.01.01_BR_RGS69.GQ365652     AG-TGGCGCC CGAACAGG-- ---------- ---------- ---GACTTGA AA-ACGAAAG TAAGA----- ---------- ---------- ----------
BC.BR.02.02_BR_HF31.GQ365649      AG-TGGCGCC CGAACAGG-- ---------- ---------- ---GACTTGA AA-GCGAAAG TAAGA----- ---------- ---------- ----------
BC.BR.05.05_BR_NSP24.GQ365650     AG-TGGCGCC CGAACAGG-- ---------- ---------- ---GACCAGA AA-GCGAAAG TAGAA----- ---------- ---------- ----------
BC.BR.06.06BR1114.JN692478        AG-TGGCGCC CGAACAGG-- ---------- ---------- ---GACTTGA AA-GCGAAAG TAGAA----- ---------- ---------- ----------
BC.BR.92.92BR023.HM100716         AG-TGGCGCC CGAACAGG-- ---------- ---------- ---GACTTGA AA-GCGAAAG TAAGA----- ---------- ---------- ----------
BC.CN.07.309.HM776938             AG-TGGCGCC CGAACAGG-- ---------- ---------- ---GACTTGA AA-GCGAAAG TGAGA----- ---------- ---------- ----------
BC.CN.07.341.HM776939             -G-TGGCGCC CGAACAGG-- ---------- ---------- ---GACTTGA AA-GTGAAAG TAAGA----- ---------- ---------- ----------
```

```
BC.IN.02.INDNARI_0218440.EU000   AG-TGGCGCC CGAACAGG-- ---------- ---------- --GACCCGAA AA-GTGAAAG TAAGA----- ---------- ---------- ----------
BC.IN.02.NARI7_3.EU000511        AG-TGGCGCC CGAACAGG-- ---------- ---------- ---GACCCGA AA-GCGAAAG TAAGA----- ---------- ---------- ----------
BC.IN.02.NARI9_3.EU000508        AG-TGGCGCC CGAACAGG-- ---------- ---------- ---GACTTGA AA-GCGAAAG TAAGA----- ---------- ---------- ----------
BC.IN.99.NARI10_2.EU000516       AG-TGGCGCC CGAACAGG-- ---------- ---------- ---GACGCGA AA-GCGAAAG TGAGA----- ---------- ---------- ----------
BC.MM.99.mIDU103.AB097873        AG-TGGCGCC CGAACAGG-- ---------- ---------- ---GACCTGA AA-GCGAAAG TGAGA----- ---------- ---------- ----------
BCU.FR.06.06FR_CRN.EU448296      AG-TGGCGCC CGAACAGG-- ---------- ---------- ---GACCTGA AA-GCGAAAG TAAGA----- ---------- ---------- ----------
BF.BR.03.BREPM1026.EF637055      ---------- ---------- ---------- ---------- -GGAACTTGA AA-GCGAAAG TGAGA----- ---------- ---------- ----------
BF.BR.03.BREPM1029.EF637052      ---------- ---------- ---------- ---------- ---------- ---------- ---------- ---------- ---------- ----------
BF1.BR.00.BREPM13853.DQ085875    AG-TGGCGCC CGAACAGG-- ---------- ---------- ---GACTTGA AA-ACGAAAG TAAAA----- ---------- ---------- ----------
BF1.BR.01.01BR042.DQ358799       AG-TGGCGCC CGAACAGG-- ---------- ---------- ---GACTTGA AA-GCGAAAG TAGAA----- ---------- ---------- ----------
BF1.BR.01.01BR047.DQ358800       AG-TGGCGCC CGAACAGG-- ---------- ---------- ---GACCTGA AA-GCGAAAG TAGAA----- ---------- ---------- ----------
BF1.BR.01.01BR226.DQ358803       AG-TGGCGCC CGAACAGG-- ---------- ---------- ---GACCTGA AA-GCGAAAG TAGAA----- ---------- ---------- ----------
BF1.BR.01.01BR323.DQ358804       AG-TGGCGCC CGAACAGG-- ---------- ---------- ---GACTTGA AA-GTGAAAG TAGAA----- ---------- ---------- ----------
BF1.BR.02.02BR005.DQ358806       AG-TGGCGCC CGAACAGG-- ---------- ---------- ---GACCCGA AA-GCGAAAG TAGAA----- ---------- ---------- ----------
BF1.BR.02.02BR006.DQ358807       AG-TGGCGCC CGAACAGG-- ---------- ---------- ---GACCCGA AA-GCGAAAG TAGAA----- ---------- ---------- ----------
BF1.BR.02.02BR033.DQ358811       ---------- ---------- ---------- ---------- -------TGA AA-GCGAAAG TAGAA----- ---------- ---------- ----------
BF1.BR.02.02BR034.DQ358812       AG-TGGCGCC CGAACAGG-- ---------- ---------- ---GACGTGA AA-GCGAAAG TAGAA----- ---------- ---------- ----------
BF1.BR.02.02BR2028.JN692437      AG-TGGCGCC CGAA-AGG-- ---------- ---------- ---GACTTGA AA-GCGAAAG TAGAG----- ---------- ---------- ----------
BF1.BR.03.03BR2018.JN692449      AG-TGGCGCC CGAACAGG-- ---------- ---------- ---GACCAGA AA-GCGAAAG TAGAA----- ---------- ---------- ----------
BF1.BR.03.03BR2019.JN692448      ---------- ---------- ---------- ---------- ---------- ---------- ----T----- ---------- ---------- ----------
BF1.BR.04.04BR1067.JN692456      AG-TGGCGCC CGAACAGG-- ---------- ---------- ---GACCCGA AAAGCGAAAG TAGAA----- ---------- ---------- ----------
BF1.BR.05.0008SP.JF804805        -----GCGCC CGAACAGG-- ---------- ---------- ---GAC-TGA AA-GCGAAAG TAGAA----- ---------- ---------- ----------
BF1.BR.05.0632SV.JF804810        ---------- ---------- ---------- ---------- ---------- ---------- ---------- ---------- ---------- ----------
BF1.BR.06.06BR_FPS561.HM026455   AG-TGGCGCC CGAACAGG-- ---------- ---------- ---GACTTGA AA-GCGAAAG CAGAA----- ---------- ---------- ----------
BF1.BR.99.BREPM107.AY771588      ---------- ---------- ---------- ---------- ---------- ---------- ---------- ---------- ---------- ----------
BF1.BR.99.BREPM108.AY771589      AG-TGGCGCC CGAACAGG-- ---------- ---------- ---GACTTGA AA-ACGAAAG TAAGA----- ---------- ---------- ----------
BF1.BR.99.BREPM11931.DQ085869    AG-TGGCGCC CGAACAGG-- ---------- ---------- ---GACCTGA AA-GCGAAAG TAAGA----- ---------- ---------- ----------
BF1.ES.08.ES_X2524_2.GQ372989    AG-TGGCGCC CGAACAGG-- ---------- ---------- ---GACTTGA AA-GCGAAAG TAGAA----- ---------- ---------- ----------
BF1.ES.08.X2432_2.FJ853621       AG-TGGCGCC CGAACAGG-- ---------- ---------- ---GACCTGA AA-GCGAAAG TAGAA----- ---------- ---------- ----------
BF1.ES.09.DEMBF09ES003.JX14066   AG-TGGCGCC CGAACAGG-- ---------- ---------- ---GACGTGA AA-ACGAAAG TAGAA----- ---------- ---------- ----------
BF1.ES.09.DEMBF09ES006.JX14066   ---------- ---ACAGG-- ---------- ---------- ---GACTTGA AA-GCGAAAG TAGAA----- ---------- ---------- ----------
BF1.IT.01.53143.GU595149         ---------- ---------- ---------- ---------- ---------- ---------- ---------- ---------- ---------- ----------
BF1.IT.02.30638.GU595148         ---------- ---------- ---------- ---------- ---------- ---------- ---------- ---------- ---------- ----------
BF1.IT.02.57954.GU595150         AG-TGGCGCC CGAACAGG-- ---------- ---------- ---GACTCGA AA-GCGAAAG TAGAA----- ---------- ---------- ----------
BF1.IT.02.58736.GU595160         AG-TGGCGCC CGAACAGG-- ---------- ---------- ---GACTTGA AA-GCGAAAG TAGAA----- ---------- ---------- ----------
BF1.IT.02.59211.GU595151         AG-TGGCGCC CGAACAGG-- ---------- ---------- ---GACTTGA AA-GCGAAAG TAAGA----- ---------- ---------- ----------
BF1.IT.05.83166.GU595152         AG-TGGCGCC CGAACAGG-- ---------- ---------- ---GACTTGA AA-GCGAAAG TAAAA----- ---------- ---------- ----------
BF1.IT.06.89072.GU595153         AG-TGGCGCC CGAACAGG-- ---------- ---------- ---GACTAGA AA-GCGAAAG TAGAA----- ---------- ---------- ----------
BF1.JP.04.DR6082.AB480298        AG-TGGCGCC CGAACAGG-- ---------- ---------- ---GACGCGA AA-GCGAAAG TAGAA----- ---------- ---------- ----------
BF1.JP.04.DR6190.AB480300        AG-TGGCGCC CGAACAGG-- ---------- ---------- --GACGCTGA AA-GCGAAAG TAGAA----- ---------- ---------- ----------
BF1.JP.x.DR0769.AB253430         AG-TGGCGCC CGAACAGG-- ---------- ---------- ---GACGCGA AA-GCGAAAG TAGAA----- ---------- ---------- ----------
BFG.MO.05.MO108.GU207884         ---------C CGAACAGGGA CTTGAAA-GC GAAAGTTAAC AGGGACTCGA AA-GCGAAAG TT-------- ---------- ---------- ----------
BG.CU.x.Cu100.AY586546           AG-TGGCGCC CGAACAGGGA CTTGAAA-GC GAAAGTTAAA AGGGACTCGA AA-GCGAAAG TT-------- ---------- ---------- ----------
BG.DE.01.9196_01.AY882421        AG-TGGCGCC CGAACAGGGA CTTGAAA-GC GAAAGT-AAC AGGGACTCGA AA-GCGAAAG TT-------- ---------- ---------- ----------
CD.KE.01.ML1076.EU110086         ---------- ---------- ---------- ---------- ---------- ---------- --AGA----- ---------- ---------- ----------
CU.JP.04.DR5782.AB286849         AG-TGGCGCC CGAACAGG-- ---------- ---------- ---GACGCGA AA-GCGAAAG TAAAA----- ---------- ---------- ----------
DF1G.ES.04.X963_4.FJ670527       AG-TGGCGCC CGAACAGG-- ---------- ---------- ---GACTTGA AA-GTGAAAG TTAATAGGGA CTCGAAAGCG GAAGTT---- -----C----
DO.FR.08.RBF208.GQ351296         AG-TGGCGCC CGAACAGG-- ---------- ---------- ---GACCGGA AA-GAGAAAG TGAAA----- ---------- ---------- ----------
```

	205	215	225	235	245	255	265	275	285	295
		---SD/3WJ----							---------SD-----------	
										------SDa----
B.FR.83.HXB2_LAI_IIIB_BRU.K034	CCAGAGGAG-	--CTCTCTCG	ACGCAGG--A	CTCGGCTTGC	TGAA------	GCGCGCACGG	CAAGAGGCGA	GGGG----CG	GCGA---CTG	--GTGAGTAC
A1.CD.02.02CD_KTB035.AM000055	ACAGAGAAG-	--TTCTCTCG	ACGCAGG--A	CTCGGCTTGC	TGAG------	GTGCACACAG	CAAGAGGCGA	GAG-----CG	GCGA---CTG	--GTGAGTAC
A1.CD.97.97CD_KCC2.AM000053	CCAGAGAAG-	--TTCTCTCG	ACGCAGG--A	CTCGGCTTGC	TGAG------	GTGCACACAG	CAAGAGGCGA	GAG-----CG	GCGA---CTG	--GTGAGTAC
A1.ES.05.X1608_8.FJ670519	CCAGAGAAG-	--TTCTCTCG	ACGCAGG--A	CTCGGCTTGC	TGAG------	GTGCACACAG	CAAGAGGCGA	GAG-----CG	GCGA---CTG	--GTGAGTAC
A1.IT.02.60000.EU861977	CCAGAGAAG-	--WTCTCTCG	ACGCAGG--A	CTCGGCTTGC	TGAG------	GTGCACACAG	CAAGAGGCGA	GAGG----CG	GCGA---CTG	--GTGAGTAC
A1.KE.01.ML1901PCR2.EU110095	CCAGAGAAG-	--ATCTCTCG	ACGCAGG--A	CTCGGCTTGC	TGACTGAA--	GTGCACACAG	CAAGAGGCGA	GAG-----CG	GCGA---CTG	--GTGAGTAC
A1.KE.01.ML1945.EU110088	CCAGAGAAG-	--TTCTCTCG	ACGCAGG--A	CTCGGCTTGC	TGAG------	GTGCACACAG	CAAGAGGCGA	GAG-----CG	GCGA---CTG	--GTGAGTAC
A1.KE.02.ML1990.EU110092	CCAGAGAAG-	--TTCTCTCG	ACGCAGG--A	CTCGGCTTGC	TGAG------	-TGCACACAG	CAAGAGGCGA	GAG-----CG	GCGA---CTG	--GTGAGTAC
A1.KE.02.ML2014.EU110094	CCAGAGAAG-	--TTCTCTCG	ACGCAGG--A	CTCGGCTTGC	TGAG------	GTGCACACAG	CAAGAGGCGA	GAG-----CG	GCGA---CTG	--GTGAGTAC
A1.KE.86.ML170_1986.AF539405	CCAGAGAAG-	--TTTTCTCG	ACGCAGG--A	CTCGGCTTGC	TGAG------	GTGCACACAG	CAAGAGGCGA	GAG-----CG	GCGA---CTG	--GTGAGTAC
A1.KE.94.Q23_17.AF004885	CCAGAGAAG-	--TTCTCTCG	ACGCAGG--A	CTCGGCTTGC	TGAG------	GTGCACACAG	CAAGAGGCGA	GAG-----CG	GCGA---CTG	--GTGAGTAC
A1.KE.97.ML013_2.AY322185	CCAGAGAAG-	--TTCTCTCG	ACGCAGG--A	CTCGGCTTGC	TGAG------	GTGCACACAG	CAAGAAGCGA	GAG-----CG	GCGA---CTG	--GTGAGTAC
A1.KE.97.ML605_3.AY322190	CCAGAGAAG-	--TTCTCTCG	ACGCAGG--A	CTCGGCTTGC	TGAG------	GTGCACACAG	CAAGAGGCGA	GAG-----CG	GCGA---CTG	--GTGAGTAC
A1.KE.97.ML752.AY322193	CCAGAGAGG-	--TTCTCTCG	ACGCAGG--A	CTCGGCTTGC	TGAG------	GTGCACACAG	CAAGAGGCGA	GAG-----CG	GCGA---CTG	--GTGAGTAC
A1.RU.00.RU00051.EF545108	----------	----------	----------	CTCGGCTTGC	TGAG------	GTGC-CACAG	CAAGAGGCGA	GAG-----CG	GCGA---CYG	--GTGAGTAC
A1.RU.05.RU_560_1125_JA.JQ2928	----------	----------	----------	----------	----------	----------	----------	GAG-----CG	GGGA---CTG	--GGGAGTAC
A1.RU.06.RU_915_1016.JQ292896	CCAGAGAAG-	--TTCTCTCG	ACGCAGG--A	CTCGGCTTGC	TGAG------	GTGCACACAG	CAAGAGGCGA	GAG-----CG	GCGA---CTG	--GTGAGTAC
A1.RU.06.RU_915_1035.JQ292897	CCAGAGAAG-	--TTCTCTCG	ACGCAGG--G	CTCGGCTTGC	TGAG------	GTGCACACAG	CAAGAGGCGA	GAG-----CG	GCGA---CTG	--GTGAGTAC
A1.RU.06.RU_915_1038.JQ292898	CCAGAGAAG-	--TTCTCTCG	ACGCAGG--A	CTCGGCTTGC	TGAG------	GTGCACACAG	CAAGAGGCGA	GAG-----CG	GCGA---CTG	--GTGAGTAC
A1.RU.06.RU_915_1041.JQ292899	CCAGAGAAG-	--TTCTCTCG	ACGCAGG--A	CTCGGCTTGC	TGAG------	GTGCACACAG	CAAGAGGCGA	GAG-----CG	GCGA---CTG	--GTGAGTAC
A1.RU.06.RU_SP_B_049.JQ292900	CCAGAGAAG-	--TTCTCTCG	ACGCAGG--G	CTCGGCTTGC	TGAG------	GTGCACACAG	CAAGAGGCGA	GAG-----CG	GCGA---CTG	--GTGAGTAC
A1.RU.07.Irkutsk_5.JQ292891	----------	----------	----------	----------	----------	----------	----------	---G-----CG	GGGA---CTG	--GGGAGTAC
A1.RU.08.PokA1Ru.FJ864679	CCAGAGAAG-	--TTCTCTCG	ACGCAGG--A	CTCGGCTTGC	TGAG------	GTGCACACAG	CAAGAGGCGA	GAG-----CG	GCGA---CTG	--GTGAGTAC
A1.RU.08.RUA001.JQ292893	CCAGAGAAG-	--ATCTCTCG	ACGCAGG--A	CTCGGCTTGC	TGAG------	GTGCACACAG	CAAGAGGCGA	GAACGAG-CG	GCGA---CTG	--GTGAGTAC
A1.RU.08.RUA007.JQ292894	CCAGAGAAG-	--TTCTCTCG	ACGCAGG--A	CTCGGCTTGC	TGAG------	GTGCACACAG	CAAGAGGCGA	GAG-----CG	GCGA---CTG	--GTGAGTAC
A1.RU.10.10RU6617.JX500696	CCAGAGTAG-	--ATCTCTCG	ACGCAAGT-A	CTCGGCTTGC	TGAG------	GTGCACACAG	CAAGAGGCGA	GAG-----CG	GCGA---CTG	--GTGAGTAC
A1.RU.10.10RU6792.JX500695	CCAGAGAAG-	--TTCTCTCG	ACGCAGG--A	CTCGGCTTGC	TGAG------	GTGCACACAG	CAAGAGGCGA	GAG-----CG	GCGA---CTG	--GTGAGTAC
A1.RU.11.11RU6950.JX500694	CCAGAGAAG-	--TTCTCTCG	ACGCAGG--A	CTCGGCTTGC	TGAG------	GTGCACACAG	CAAGAGGCGA	GAG-----CG	GCGA---CTG	--GTGAGTAC
A1.RW.07.pR463F.JX236677	CCAGAGAAG-	--TTCTCTCG	ACGCAGG--A	CTCGGCTTGC	TGA-------	GTGCACACAG	CAAGAGGCGA	GAG-----CG	GCGA---CTG	--GTGAGTAC
A1.RW.07.pR880F.JX236678	CCAGAGAAG-	--TTCTCTTG	ACGCAAG--A	CTCGGCTTGT	TGAG------	GTGCACACAG	CAAGCGGCGA	GAG-----CG	GCGA---CTG	--GTGAGTAC
A1.RW.92.92RW008.AB253421	CCAGAGAAG-	--TTCTCTCG	ACGCAGG--A	CTCGGCTTGC	TGAG------	GTGCACACAG	CAAGAGGCGA	GAG-----CG	GCGA---CTG	--GTGAGTAC
A1.RW.92.92RW025A.AB287376	CCAGAGAAG-	--TTCTCTCG	ACGCAGG--A	CTCGGCTTGC	TGAG------	GTGCACACAG	CAAGAGGCGA	GAG-----CG	GCGA---CTG	--GTGAGTAC
A1.RW.93.93RW037A.AB287379	CCAGAGAAG-	--TTCTCTCG	ACGCAGG--A	CTCGGCTTGC	TGAG------	GTGCACACAG	CAAGAGGCGA	GAG-----CG	GCGA---CTG	--GTGAGTAC
A1.SE.95.UGSE8131.AF107771	CCAGAGAAT-	---TCTCTCG	ACGCAGG--A	CTCGGCTTGC	TGAA-----G	GTGCACACAG	CAAGAGGCGA	GAG-----CG	GCGA---CTG	--GTGAGTAC
A1.TZ.97.97TZ03.AF361873	CCAGAGAAG-	--TTCTCTCG	ACGCAGG--A	CTCGGCTTGC	TGAG------	GTGCACACAG	CAAGAGGCGA	GAG-----CG	GCGA---CTG	--GTGAGTAC
A1.UA.00.98UA0116.AF413987	CCAGAGAAG-	--TTCTCTCG	ACGCAGG--A	CTCGGCTTGC	TGAG------	GTGCACACAG	CAAGAGGCGA	GAG-----CG	GCGA---CTG	--GTGAGTAC
A1.UG.07.p191084.JX236669	CCAGAGAAG-	--CTCTCTCG	ACGCGAGG-A	CTCGGCTTGC	TGAG------	GTGCACACAG	CAAGGGGCGA	GAG-----CG	GCGA---CTG	--GTGAGTAC
A1.UG.07.p191845.JX236671	CCAGAGAAG-	--ATCTCTCG	ACGCAGG--A	CTCGGCTTGC	TGAG------	GTGCACACAG	CAAGAGGCGA	GAG-----CG	GCGA---CTG	--GTGAGTAC
A1.UG.07.p9004SDM.JX236676	CCGGAGAAG-	--TTCTCTCG	ACGCAGG--A	CTCGGCTTGC	TGAG------	GTGCACACAG	CAAGAGGCGA	GAG-----CG	GCGA---CTG	--GTGAGTAC
A1.UG.85.U455_U455A.M62320	CCAGAGAAG-	--TTCTCTCG	ACGCAGG--A	CTCGGCTTGC	TGAG------	GTGCACACAG	CAAGAGGCGA	GAG-----CG	GCGA---CTG	--GTGAGTAC
A1.UG.92.92UG037.AB253429	CCAGAGAAGA	--AGCTCTCT	TGACGCAGGA	CTCGGCTTGC	TGAG------	GTGCACACAG	CAAGAGGCGA	GAG-----CG	GCGA---CTG	--GTGAGTAC
A1.UG.92.UG029.AB098333	CCAGAGGAG-	--TTCTCTCG	ACGCAGG--A	CTCGGCTTGC	TGAG------	GTGCACACAG	CAAGAGGCGA	GAG-----CG	GCGA---CTG	--GTGAGTAC
A1.UG.x.UG031.AB098331	CCAGAGAAG-	--TTCTCTCG	ACGCAGG--A	CTCGGCTTGC	TGAG------	GTGCACACAG	CAAGAGGCGA	GAG-----CG	GCGA---CTG	--GTGAGTAC
A1.UG.x.UG275.AB485632	CCAGAGAAG-	--TTCTCTCG	ACGCAGG--A	CTCGGCTTGC	TGAG------	GTGCACACAG	CAAGAGGCGA	GAG-----CG	GCGA---CTG	--GTGAGTAC
A1.ZA.04.04ZASK162B1.DQ396400	CCAGAGAAG-	--TTCTCTCG	ACGCAGG--A	CTCGGCTTGC	TGAG------	GTGCACACAG	CAAGAGGCGA	GAG-----CG	GCGA---CTG	--GTGAGTAC
A2.CD.97.97CDKTB48.AF286238	CCAGGGAAG-	--TTCTCTCG	ACGCAAG--A	CTCGGCTTGC	TGAG------	GTGCACACGG	CAAGAGGCGA	GAAG----CG	ACGG---CTG	--GTGAGTAC
A2.CY.94.94CY017_41.AF286237	CCAGAGGAG-	--TTCTCTCG	ACGCAGG--A	CTCGGCTTGC	TGAG------	GTGCACACGG	CAAGAGGCGA	GGGG----CG	GCGA---CTG	--GTGAGTAC
B.AU.86.MBC200.AF042100	CCAGAGGAG-	--CTCTCTCG	ACGCAGG--A	CTCGGCTTGC	TGAA------	GCGCGCACGG	CAAGAGGCGA	GGGG----CG	GCGA---CTG	--GTGAGTAC
B.AU.87.MBC925.AF042101	CCAGAGGAG-	--CTCTCTCG	ACGCAGG--A	CTCGGCTTGC	TGTA------	GCGCGCACGG	CAAGAGGCGA	GGGG----CG	GCGA---CTG	--GTGAGTAC
B.AU.95.C24.AF538304	CCAGAGGAG-	--CTCTCTCG	ACGCAGG--A	CTCGGCTTGC	TGAA------	ACGCGCGCGG	CAAGAGGCGA	GGGG----CG	GCGA---CCG	--GTGAGTAC
B.AU.95.C42.AF538305	CCAGAGGAG-	--CTCTCTCG	ACGCAAG--A	CTCGGCTTGC	TGAA------	GCGCGCATAG	CAAGAGGCGA	GGGG----CG	GCGA---CTG	--GTGAGTAC
B.AU.95.C76.AF538306	CCAGAGGAG-	--CTCTCTCG	ACGCAGG--A	CTCTGCTTGC	TGAA------	GCGCGCACAG	CAAGAGGCGA	GGGG----CG	GCGA---CTG	--GTGAGTAC
B.AU.95.C92.AF538307	CCAGAGGAG-	--CTCTCTCG	ACGCAGG--A	CTCGGCTTGC	TGAA------	ACGCGCGCGG	CAAGAGGCGA	GGGG----CG	GCGA---CCG	--GTGAGTAC
B.AU.95.MBCC54.AF042103	CCAGAGGAG-	--CTCTCTCG	ACGCAGG--A	CTCGGCTTGC	TGAA------	GCGCGCACGG	CAAGAGGCGA	GGGG----CG	GCGA---CTG	--GTGAGTAC
B.AU.96.MBCC98.AF042104	CCAGAGGAG-	--CTCTCTCG	ACGCAGG--A	CTCGGCTTGC	TGAA------	GCGCGCACGG	CAAGAGGCGA	GGGG----TG	GCGA---CTG	--GTGAGTAC
B.AU.96.MBCD36.AF042105	CCAGAGAAG-	--ATCTCTCG	ACGCAGG--A	CTCGGCTTGC	TGAA------	GCGCGCACAG	CAAGAGGCGA	GGGG----CG	GCGA---CTG	--GTGAGTAC
B.AU.99.1181.AF538302	CCAGAGGAG-	--CTCTCTCG	ACGCAGG--A	CTCGGCTTGC	TGAA------	GCGCGCACGG	CAAGAGGCGA	GGGG----CG	GCGA---CTG	--GTGAGTAC
B.AU.x.2870718.AY857022	CCAGAGGAG-	--ATCTCTCG	ACGCAGG--A	CTCGGCTTGC	TGAA------	GCGCGCACAG	CAAGAGGCGA	GGGG----CG	GCGA---CTG	--GTGAGTAC
B.AU.x.9125091.AY857165	----------	----------	----------	----------	--------TG	AAGCGCACGG	CAAGAGGCGA	GGGG----CG	GCGA---CTG	--GTGAGTAC
B.AU.x.VH_VHPCR.AF146728	---GAGGAG-	--CTCTCTCG	ACGCAGG--A	CTCGGCTTGC	TGAA------	GCGCGCACGG	CAAGAGGCGA	GGGG----CG	GCGA---ACG	--GTGAGTAC
B.BO.09.DEMB09BO001.JX140656	CCAGAGGAG-	--ATCTCTCG	ACGCAGG--A	CTCGGCTTGC	TGAA------	GCGCGCACGG	CAAGAGGCGA	GGGG----AG	GCGA---CTG	--GTGAGTAC
B.BR.02.02BR008.DQ358808	CCAGAGGAG-	--CTCTCTCG	ACGCAGG--A	CTCGGCTTGC	TGAA------	GCGCGCACAG	CAAGAGGCGA	GGGG----CG	GCGA---CTG	--GTGAGTAC
B.BR.02.02BR011.DQ358809	CCAGAGGAG-	--ATCTCTCG	ACGCAGG--A	CTCGGCTTGC	TGAA-----A	GCGCGCTCGG	CAAGAGGCGA	GGGG----CG	GCGA---CTG	--GTGAGTAC
B.BR.02.02BR013.DQ358810	CCAGAGGAG-	--CTCTCTCG	GACGCAGG-A	CTCGGCTTGC	TGAA------	GCGCGCACGG	CAAGAGGCGA	GGGG----CG	GCGA---CTG	--GTGAGTAC
B.BR.02.02BR1013.JN692432	CCAGAGGAG-	--ATCTCTCG	ACGCAGG--A	CTCGGCTTGC	TGAA------	GCGCGCACGG	CAAGAGGCGA	GGGG----CG	GCGA---CTG	--GTGAGTAC
B.BR.02.02BR2025.JN692435	CCAGAGGAG-	--TTCTCTCG	ACGCAGG--A	CTCGGCTTGC	TGAA------	GCGCGCGCGG	CAAGAGGCGA	GGGG----CG	GCGA---CCG	--GTGAGTAC
B.BR.02.02BR2032.JN692439	CCAGAGGAG-	--ATCTCTCG	ACGCAGG--A	CTCGGCTTGC	TGAA------	GCGCGCACGG	CAAGAGGCGA	GGGG----CG	GCGA---CTG	--GTGAGTAC
B.BR.02.02BR2033.JN692440	CCAGAGGAG-	----CTCTCG	ACGCAGG--A	CTCGGCTTGC	TGAA------	GCGCGCACAG	CAAGAGGCGA	GGGG----CG	GCGA---CTG	--GTGAGTAC
B.BR.02.02BR2041.JN692443	CCGGAGGAG-	--CTCTCTCG	ACGCAGG--A	CTCGGCTTGC	TGAA------	GCGCGCGCGG	CAAGAGGCGA	GGGG----CG	GCGA---CTG	--GTGAGTAC
B.BR.02.02BR2042.JN692444	CCAGAGGAG-	--CTCTCTCG	ACGCAGG--A	CTCGGCTTGC	TGAA------	GCGCGCGCGG	CAAGAGGCGA	GGGG----CG	GCGA---CTG	--GTGAGTAC
B.BR.02.04BR1064.JN692433	CCAGAGGAG-	--ATCTCTCG	ACGCAGG--A	CTCGGCTTGC	TGAA------	GCGCGCACGG	CAAGAGGCGA	GGGG----CG	GCGA---CTG	--GTGAGTAC
B.BR.02.05BR1094.JN692470	CCAGAGGAG-	--CTCTCTCG	ACGCAGG--A	CTCGGCTTGC	TGAA------	GCGCGCGCGG	CAAGAGGCGA	GGGG----CG	GCGA---CTG	--GTGAGTAC
B.BR.03.03BR1020.JN692445	CCAGAGGAG-	--ATCTCTCG	ACGCAGG--A	CTCGGCTTGC	TGAA------	GCGCGCTCGG	CAAGAGGCGA	GGGG----CG	GCGA---CTG	--GTGAGTAC
B.BR.03.03BR1046.JN692447	CCAGAGAAG-	--ATCTCTCG	ACGCAGG--A	CTCGGCTTGC	TGAA------	GCGCGCACAG	CAAGAGGCGA	GGGG----CG	GCGA---CTG	--GTGAGTAC
B.BR.03.BREPM1023.EF637057	CCAGAGGAG-	--ATCTCTCG	ACGCAGG--A	CTCGGCTTGC	TGAA------	GCGCGCACAG	CAAGAGGTGA	GGGG----CG	GCGA---ACG	--GTGAGTAC
B.BR.03.BREPM1024.EF637056	CCAGAGAAG-	--ATCTCTCG	ACGCAGG--A	CTCGGCTTGC	TGAA------	GCGCGCACAG	CAAGAGGCGA	GGGG----CG	GCGA---CTG	--GTGAGTAC
B.BR.03.BREPM1027.EF637054	CCAGAGAAG-	--ATCTCTCG	ACGCAGG--A	CTCGGCTTGC	TGAA------	GCGCGCACGG	CAAGAGGCGA	GGGG----CG	GCGA---ACG	--GTGAGTAC
B.BR.03.BREPM1028.EF637053	CCAGAGGAG-	--CTCTCTCG	GCGCAGG--A	CTCGGCTTGC	TGAA------	GCGCGCACGG	CAAGAGGCGA	GGGG----CG	GCGA---CTG	--GTGAGTAC
B.BR.03.BREPM1032.EF637051	----------	G-ATCTCTCG	ACGCAGG--A	CTCGGCTTGC	TGAA------	GCGCGCACGG	CAAGAGGCGA	GGGG----CG	GCGA---ACG	--GTGAGTAC
B.BR.03.BREPM1033.EF637050	CCAGAGGAG-	--ATCTCTCG	ACGCAGG--A	CTCGGCTTGC	TGAA------	GCGCGCACAG	CAAGAGGCGA	GGGG----CG	GCGA---CTG	--GTGAGTAC
B.BR.03.BREPM1035.EF637049	----------	---TCTCTCG	ACGCAGG--A	CTCGGCTTGC	TGAA------	GCGCGCGCGG	CAAGAGGCGA	GGGG----CG	GCGA---CTG	--GTGAGTAC
B.BR.03.BREPM1038.EF637048	CCAGAGGAG-	--ATCTCTTG	ACGCAGG--A	CTCGGCTTGC	TGAA------	GCGCGCACAG	CAAGAGGCGA	GGGG----CG	GCGA---CTG	--GTGAGTAC
B.BR.03.BREPM1040.EF637047	CCAGAGGAG-	--ATCTCTCG	ACGCAGG--A	CTCGGCTTGC	TGAA------	GCGCGCGCGG	CAAGAGGCGA	GGGG----CG	GCGA---CTG	--GTGAGTAC
B.BR.03.BREPM2012.EF637046	CCAGAGGAG-	--ATCTCTCG	ACGCAGG--A	CTCGGCTTGC	TGAA------	GCGCGCACAG	CAAGAGGCGA	GGGG----CG	GCGA---CTG	--GTGAGTAC
B.BR.04.04BR1047.JN692450	CCAGAGGAG-	--CTCTCTCG	ACGCAGG--A	CTCGGCTTGG	TGAA------	GCGCGCACGG	CAAGAGGCGA	GGGG----CG	GCGA---CTG	--GTGAGTAC
B.BR.04.04BR1049.JN692451	CCAGAGGAG-	--AACTCTCG	ACGCAGG--A	CTCGGCTTGC	TGAA------	GCGCGCACGG	CAAGAGGCGA	GGGG----CG	GCGA---CTG	--GTGAGTAC
B.BR.04.04BR1051.JN692452	CCAGAGGAG-	--ATCTCTCG	ACGCAGG--A	CTCGGCTTGC	TGAA------	GTGCGCACGG	CAAGAGGCGA	GGGGA---CG	GCGG---CTG	G-GTGAGTAC
B.BR.04.04BR1054.JN692453	CCAGAGAAG-	--ATCTCTCG	ACGCAGG--A	CTCGGCTTGC	TGAA------	GCGCGCACAG	C-AGAGGCGA	GGGG----CG	GCGA---CTG	--GTGAGTAC
B.BR.04.04BR1055.JN692454	CCAGAGACG-	--ATCTCTCG	ACGCAGG--A	CTCGGCTTGC	TGAA------	GTGCGCACGG	CAAGAGGCAA	GGGG----CG	GCGA---CTG	--GTGAGTAC
B.BR.04.04BR1057.JN692455	CCAGAGGAG-	--CTCTCTTG	ACGCAGG--A	CTCGGCTTGC	TGAA------	GCGCGCACAG	CAAGAGGCGA	GGGG----CG	GCGA---CTG	--GTGAGTAC
B.BR.04.04BR1068.JN692457	CCAGAGAAG-	--ATCTCC-G	ACGCAGG--A	CTCGGCTTGC	TGAA------	GCGCGCACAG	CAAGAGGCGA	GGGG----CG	GCGA---CTG	--GTGAGTAC
B.BR.04.BREPM1066.FJ195090	CCAGGAGAG-	--CTCTCTCG	ACGCAGG--A	CTCGGCTTGC	TGAA------	GCGCGCACAG	CAAGAGGCGA	GGGG----CG	GCGA---CTG	--GTGAGTAC
B.BR.04.BREPM1070.FJ195086	CCGGAGGAC-	--CTCTCTGG	CCCCAGG--A	CTGGGCTTCT	GGAA------	GCGCCCCCGC	CAAGAGGGAG	GGGG----GG	GGGA---TTG	--GTGAGTCC
B.BR.05.05BR1077.JN692460	CCAGAGGAG-	--ATCTCTCG	ACGCAAGG-A	CTCGGCTTGC	TGAA------	GTGCGCACAG	CAAGAGGCGA	GGGG----CG	GCGA---CTG	--GTGAGTAC
B.BR.05.05BR1078.JN692461	CCAGAGGAG-	--ATCTCTCG	ACGCAGG--A	CTCGGCTTGC	TGAA----AA	GCGCGCTCGG	CAAGAGGCGA	GGGG----CG	GCGA---CTG	--GTGAGTAC
B.BR.05.05BR1079.JN692462	CCAGAGAAG-	--ATCTCTCG	ACGCAGG--A	CTCGGCTTGC	TGAA------	GCGCGCACGG	CAAGAGGCGA	GGGG----CG	GCGA---CTG	--GTGAGTAC
B.BR.05.05BR1080.JN692463	CCAGAGGAG-	--ATCTCTCG	ACGCAGG--A	CTCGGCTTGC	TGAA------	GCGCGCACGG	CAAGAGGCGA	GGGG----CG	GCGA---CTG	--GTGAGTAC
B.BR.05.05BR1082.JN692465	CCAGAGAAG-	--CTCTCTTG	ACGCAGT--A	CTCGGCTTGC	TGAA------	GCGCGCACAG	CAAGAGGCGA	GTGG----CG	GCGA---CTG	--GTGAGTAC
B.BR.05.05BR1089.JN692467	CCAGAGGAG-	--ATCTCTCG	ACGCAGG--A	CTCGGCTTGC	TGAA------	GCGCGCACGG	CAAGAGGCGA	GGGG----CG	GCGA---CTG	--GTGAGTAC
B.BR.05.05BR1092.JN692468	CCAGAGGAG-	--ATCTCTCG	ACGCAGG--A	CTCGGCTTGC	TGAA------	GCGCGCACAG	CAAGAGGCGA	GGGG----CG	GCGA---CCG	--GTGAGTAC
B.BR.05.05BR1095.JN692471	CCAGAGGAG-	--CTCTCTTG	ACGCAGG--A	CTCGGCTTGC	TGAA------	GCGCGCACAG	CAAGAGGCGA	GGGG----AG	GCGG---CTG	--GTGAGTAC

B.BR.05.05BR1101.JN692473	CCAGAGGAG-	--ATCTCTCG	ACGCAGG--A	CTCGGCTTGC	TGAA------	GCGCGCACGG	CAAGAGGCGA	GGGG----CG	GCGG---CTG	--GTGAGTAC
B.BR.05.05BR1104.JN692474	CCAGAGAAG-	--ATCTCTCG	ACGCAGG--A	CTCGGCTTAC	TGAA------	GCGCGCGCGG	CAAGAGGCGA	GGGG----CG	GCGA---CCG	--GTGAGTAC
B.BR.05.05BR1107.JN692475	CCAGAGAAG-	--ATCTCTCG	ACGCAGG--A	CTCGGCTTGC	TGAA------	GCGCGCACAG	CAAGAGGCGA	GGGG----CG	GCGA---CTG	--GTGAGTAC
B.BR.05.BREPM1081.FJ195091	CCAGAGGAG-	--ATCTCTCG	ACGCAGG--A	CTCGGCTTGC	TGAA------	GCGCGCACAG	CAAGAGGCGA	GGGGA---CG	GCGA---CTG	--GTGAGTAC
B.BR.05.BREPM1084.FJ195088	CCAGAGGAG-	--ACCTCTCG	ACGCAGG--A	CTCGGCTTGC	TGAA------	GCGCGCTCAG	CAAGAGGCGA	GGGG----CG	GCAA---CCG	--GTAAGTAC
B.BR.05.BREPM1093.FJ195089	CCAGAGGAG-	--ATCTCTCG	ACGCAGG--A	CTCGGCTTGC	TGAA------	GCGCGCACAG	CAAGAGGCGA	GGGG----CG	GCGA---CTG	--GTGAGTAC
B.BR.06.06BR1115.JN692479	CCAGAGAAG-	--ATCTCTCG	ACGCAGG--A	CTCGGTTTGC	TGAA------	GCGCGCACAG	CAAGAGGCGA	GGGG----CG	GCGA---CTG	--GTGAGTAC
B.BR.06.06BR1119.JN692480	CCAGAGGAG-	--ATCTCTCG	ACGCAGG--A	CTCGGCTTGC	TGA-------	GCGCGCACAG	CAAGAGGCGA	GGGGA---CG	GCGA---CTG	--GTGAGTAC
B.CA.06.502_1027_wg01.JF320413	CCAGAGAAG-	--CTCTCTCG	ACGCAGG--A	CTCGGCTTGC	TGAA------	GCGCGCACGG	CAAGAGGCGA	GGGG----CG	GCGA---CCG	--GTGAGTAC
B.CA.06.502_1799_FL02.JF320427	CCAGAGGAG-	--CTCTCTCG	ACGCAGG--A	CTCGGCTTGC	TGAA------	GCGCGCACAG	CAAGAGGCGA	GGGG----CG	GCGA---CTG	--GTGAGTAC
B.CA.07.502_1191_03.JF320424	CCAGAGGAG-	--ATCTCTCG	ACGCAGG--A	CTCGGCTTGC	TGAA------	GCGCGCGCGG	CAAGAGGCGA	GGGG----CG	GCGA---CTG	--GTGAGTAC
B.CA.96.WC10C_4.AY314056	CCAGAGGAG-	--ATCTCTCG	ACGCAGG--A	CTCGGCTTGC	TGAA------	GCGCGCACGG	CAAGAGGCGA	GGGG----CG	GCGA---CTG	--GTGAGTAC
B.CA.97.CANB3FULL.AY779553	----------	----------	----------	----------	----------	----------	CAAGAGGCGA	GGGG----CG	GCGA---CTG	--GTGAGTAC
B.CH.03.HIV_CH_BID_V4470_2003.	----------	--GACAAGGA	ACTACGG--A	CTCGGCTTGC	TGAA------	GCGCGCACGG	CAAGAGGCGA	GGGG----CG	GCGG---CTG	--GTGAGTAC
B.CN.01.CNHN24.AY180905	CCAGAGGAG-	--CTCTCTCG	ACGCAGG--A	CTCGGCTTGC	TGAA------	GCGCGCACAG	CAAGAGGCGA	GAG-----CG	GCGA---CTG	--GTGAGTAC
B.CN.02.02HNsc11.DQ007903	CCAGAGGAG-	--CTCTCTCG	ACGCAGG--A	CTCGGCTTGC	TGAA------	GCGCGCACAG	CAAGAGGCGA	GAG-----CG	GCGA---CTG	--GTGAGTAC
B.CN.03.SHXDC0081.JF932492	CCAGAGAAG-	--CTCTCTCG	ACGCAGG--A	CTCGGCTTGC	TGAA------	GCGCGCACAG	CAAGAGGCGA	GAG-----CG	GCGA---CTG	--GTGAGTAC
B.CN.05.05CNHB_hp3.DQ990880	CCAGAGGAG-	--ATCTCTCG	ACGCAGG--A	CTCGGCTTGC	TGAA------	GCGCGCACAG	CAAGAGGCGA	GGGG----CG	GCGA---CTG	--GTGAGTAC
B.CN.06.CC056.JF932482	CCAGAGGAG-	--CTCTCTCG	ACGCAGG--A	CTCGGCTTGC	TGAA------	GCGCGCGCAG	CAAGAGGCGA	GAG-----CG	GCGA---CTG	--GTGAGTAC
B.CN.07.AH070011.JF932468	CCAGAGAAG-	--CTCTCTCG	ACGCAGG--A	CTCGGCTTGC	TGAA------	GCGCGCACAG	CAAGAGGCGA	GAG-----CG	GCGG---CTG	--GTGAGTAC
B.CN.07.AH070014.JF932469	CCAGAGGAG-	--CTCTCTCG	ACGCAGG--A	CTCGGCTTGC	TGAA------	GCGCGCACAG	CAAGAGGCGA	GAG-----CG	GCGA---CTG	--GTGAGTAC
B.CN.07.AH070017.JF932470	CCAGAGGAG-	--CTCTCTCG	ACGCAGG--A	CTCGGCTTGC	TGAA------	GCGCGCACAG	CAAGAGGCGA	GAG-----CG	GCGA---CTG	--GTAAGTAC
B.CN.07.AH070018.JF932471	CCAGAGAAG-	--ATCTCTCG	ACGCAGG--A	CTCGGCTTGC	TGAA------	GCGCGCGCAG	CAAGAGGCGA	GAG-----CG	GCGA---CTG	--GTGAGTAC
B.CN.07.AH070057.JF932472	CCAGAGCAG-	--CTCTCTCG	ACGCAGG--A	CTCGGCTTGC	TGAA------	GCGCGCACAG	CAAGAGGCGA	GAG-----CG	GCGA---CTG	--GTGAGTAC
B.CN.07.BJ070030.JF932473	CCAGAGGAG-	--CTCTCTCG	ACGCAGG--A	CTCGGCTTGC	TGAA------	GCGCGCACAG	CAAGAGGCGA	GAG-----CG	GCGA---CTG	--GTGAGTAC
B.CN.07.CBJC261.JF932474	CCAGAGAAG-	--CTCTCTCG	ACGCAGG--A	CTCGGCTTGC	TGAA------	GCGCGCGCAG	CAAGAGGCGA	GAG-----CG	GCGA---CTG	--GTGAGTAC
B.CN.07.CBJC392.JF932475	CCAGAGGAG-	--CTCTCTCG	ACGCAGG--A	CTCGGCTTGC	TGAA------	GCGCGCACAG	CAAGAGGCGA	GAG-----CG	GCGA---CTG	--GTGAGTAC
B.CN.07.CBJC394.JF932476	CCAGAGGAG-	--CTCTCTCG	ACGCAGG--A	CTCGGCTTGC	TGAA------	GCGCGCACAG	CAAGAGGCGA	GAG-----CG	GCGA---CTG	--GTGAGTAC
B.CN.07.CBJC396.JF932477	CCAGAGAAG-	--CTCTCTCG	ACGCAGG--A	CTCGGCTTGC	TGAA------	GCGCGCACAG	CAAGAGGCGA	GAG-----CG	GCGA---CTG	--GTGAGTAC
B.CN.07.GS070017.JF932484	CCAGAGAAG-	--CTCTCTCG	ACGCAGG--A	CTCGGCTTGC	TGAA------	GCGCGCACAG	CAAGAGGCGA	GGGG----CG	GCGA---CTG	--GTGAGTAC
B.CN.07.GZ070002.JF932485	CCAGAGGAG-	--CTCTCTCG	ACGCAGG--A	CTCGGCTTGC	TGAA------	GCGCGCGCAG	CAAGAGGCGA	GAG-----CG	GCGA---CTG	--GTGAGTAC
B.CN.07.GZ070030.JF932486	CCAGAGGAG-	--CTCTCTCG	ACGCAGG--A	CTCGGCTTGC	TGAA------	GCGCGCGCAG	CAAGAGGCGA	GAG-----CG	GCGA---CTG	--GTGAGTAC
B.CN.07.HB070006.JF932487	CCAGAGGAG-	--CTCTCTCG	ACGCAGG--A	CTCGGCTTGC	TGAA------	GCGCGCGCAG	CAAGAGGCGA	GAG-----CG	GCGG---CTG	--GTGAGTAC
B.CN.07.HB070022.JF932488	CCAGAGAAG-	--ATCTCTCG	ACGCAGG--A	CTCGGCTTGC	TGAA------	GCGCGCACAG	CAAGAGGCGA	GAG-----CG	GCGA---CTG	--GTGAGTAC
B.CN.07.HB070035.JF932489	CCAGAGGAG-	--CTCTCTCG	ACGCAGG--A	CTCGGCTTGC	TGAA------	GCGCGCACAG	CAAGAGGCGA	GGGG----CG	GCGA---CTG	--GTGAGTAC
B.CN.07.JL070038.JF932490	----------	----------	----------	----------	----------	----------	----------	--------CG	GCGA---CTG	--GTGAGTAC
B.CN.07.SX070080.JF932493	CCAGAGGAG-	--ATCTCTCG	ACGCAGG--A	CTCGGCTTGC	TGAA------	GCGCGCGCAG	CAAGAGGCGA	GAG-----GG	GCGA---CTG	--GTGAGTAC
B.CN.07.hb070025.JF932499	CCAGAGGAG-	--CTCTCTCG	ACGCAGG--A	CTCGGCTTGC	TGAA------	GCGCGCACAG	CAAGAGGCGA	GAG-----CG	GCGA---CTG	--GTGAGTAC
B.CN.07.hen1345.JF932500	CCAGAGGAG-	--CTCTCTCG	ACGCAGG--A	CTCGGCTTGC	TGAA------	GCGCGCACAG	CAAGAGGCGA	GAG-----GG	GCGA---CTG	--GTGAGTAC
B.CN.08.CBJC476.JF932478	CCAGAGGSG-	--CTCTCTCG	ACGCAGG--A	CTCGGCTTGC	TGAA------	GCGCGCACAG	CAAGAGGCGA	GAG-----CG	GCGA---CTG	--GTGAGTAC
B.CN.08.CBJC489.JF932479	CCAGAGGAG-	--CTCTCTCG	ACGCAGG--A	CTCGGCTTGC	TGAA------	GCGCGCACAG	CAAGAGGCGA	GAG-----CG	GCGA---CTG	--GTGAGTAC
B.CN.08.CBJC500.JF932480	CCAGAGGAG-	--CTCTCTCG	ACGCAGG--G	CTCGGCTTGC	TGAA------	GCGTGCGCAG	CAAGAGGCGA	GAG-----CG	GCGA---CTG	--GTGAGTAC
B.CN.08.CBJC503.JF932481	CCAGAGGAG-	--CTCTCTCG	ACGCAGG--A	CTCGGCTTGC	TGAA------	GCGCGCACAG	CAAGAGGCGA	GAG-----CG	GCGA---CTG	--GTGAGTAC
B.CN.08.cbjc468.JF932498	CCAGAGGAG-	--CTCTCTCG	ACGCAGG--A	CTCGGCTTGC	TGAA------	GCGCGCACAG	CAAGAGGCGA	GAG-----CG	GCGA---CTG	--GTGAGTAC
B.CN.09.YN09P0014.JF932494	CCAGAGGAG-	--CTCTCTCG	ACGCAGG--A	CTCGGCTTGC	TGAA------	GCGCGCACAG	CAAGAGGCGA	GAG-----CG	GCGA---CTG	--GTGAGTAC
B.CN.09.ZK042.JF932497	CCAGAGGAG-	--ATCTCTCG	ACGCAGG--A	CTCGGCTTGC	TGAA------	GCGCGCGCAG	CAAGAGGCGA	GAG-----CG	GCGG---CTG	--GTGAGTAC
B.CN.10.DEMB10CN002.JX140658	CCAGAGGAG-	--CTCTCTCG	ACGCAGG--A	CTCGGCTTGC	TGAA------	GCGCGCACAG	CAAGAGGCGA	GGGG----CG	GCGA---CCG	--GTGAGTAC
B.CN.98.YN9802.JF932495	CCAGAGGAG-	--CTCTCTCG	ACGCAGG--A	CTCGGCTTGC	TGAA------	GCGCGCACAG	CAAGAGGCGA	GAG-----CG	GCGA---CTG	--GTGAGTAC
B.CN.98.YN9838.JF932496	CCGGAGGAG-	--CTCTCTCG	ACGCAGG--A	CTCGGCTTGC	TGAA------	GCGCGCACAG	CAAGAGGCGA	GAG-----CG	GCGA---CTG	--GTGAGTAC
B.CN.99.plwj.GU177863	CCAGAGGAG-	--ATCTCTCG	ACGCAGG--A	CTCGGCTTGC	TGAA------	GCGCGCGCAG	CAAGAGGCGA	GGG-----GG	GCGA---CTG	--GTGAGTAC
B.CN.x.RL42.U71182	CCAGAGGAG-	--CTCTCTCG	ACGCAGG--A	CTCGGCTTGC	TGAA------	GCGCGCACAG	CAAGAGGCGA	GAG-----CG	GCGA---CTG	--GTGAGTAC
B.CU.99.Cu19.AY586542	-----GGAG-	--CTCTCTCG	ACGCAGG--A	CTCGGCTTGC	TGAA------	GCGCGCACAG	CAAGAGGCGA	GGGG----CG	GCGA---CTG	--GTGAGTAC
B.CU.99.Cu43.AY586543	CCAGAGGAG-	--CTCTCTCG	ACGCAGG--A	CTCGGCTTGC	TGAA------	GCGCGCACGG	CAAGAGGCGA	GGGG----CG	GCGA---CTG	--GTGAGTAC
B.DE.86.D31.U43096	CCAGAGGAG-	--CTCTCTCG	ACGCAGG--A	CTCGGCTTGC	TGAA------	GCGCGCACAG	CAAGAGGCGA	GGGG----CG	GCGA---CTG	--GTGAGTAC
B.DE.86.HAN.U43141	CCAGAGGAG-	--ATCTCTCG	ACGCAGG--A	CTCGGCTTGC	TGAA------	GCGCGCACGG	CAAGAGGCGA	GGGG----CG	GCGA---CTG	--GTGAGTAC
B.DK.01.CTL_016.EF514704	CCAGAGGAG-	--ATCTCTCG	ACGCAGG--A	CTCGGCTTGC	TGAA------	GCGCGCACAG	CAAGAGGCGA	GGGG----CG	GCGA---CTG	--GTGAGTAC
B.DK.01.CTL_017.EF514705	CCAGAGGAG-	--CTCTCTCG	ACGCAGG--A	CTCGGCTTGC	TGAA------	GCGCGCACAG	CAAGAGGCGA	GGGG----CG	GCGA---CTG	--GTGAGTAC
B.DK.01.CTL_018.EF514706	CCAGAGGAG-	--CTCTCTCG	GCGCAGG--A	CTCGGCTTGC	TGAA------	GCGCGCACGG	CAAGAGGCGA	GGGG----CG	GCGA---CTG	--GTGAGTAC
B.DK.01.CTL_023.EF514707	----------	---TCTCTCG	ACGCAGG--A	CTCGGCTTGC	TGAA------	GCGCGCACAG	CAAGAGGCGA	GGGG----CG	GCGA---CTG	--GTGAGTAC
B.DK.01.CTL_030.EF514708	CCAGAGAAG-	--ATCTCTCG	ACGCAGG--A	CTCGGCTTGC	TGAA------	GCGCGCACAG	CAAGAGGCGA	GGGG----CG	GCGA---CTG	--GTGAGTAC
B.DK.01.CTL_033.EF514709	CCAGAGGAG-	--CTCTCTCG	ACGCAGG--A	CTCGGCTTGC	TGAA------	GCGCGCACAG	CAAGAGGCGA	GGGG----CG	GCGA---CTG	--GTGAGTAC
B.DK.01.CTL_035.EF514710	----------	-----TCGCA	G------G-A	CTCGGCTTGC	TGAA-----G	CGCGCACAGC	AAGAGGCGAG	GGCGG--CG	ACGA---CTG	--GTGAGTAC
B.DK.01.CTL_041.EF514711	----------	----------	----------	----------	----------	----------	----------	----------	--GA---CTG	--GTGAGTAC
B.DK.01.CTL_043.EF514712	----------	---TCTCTCG	ACGCAGG--A	CTCGGCTTGC	TGAA------	GCGCGCGCGG	CAAGAGGCGA	GGGG----CG	GCGA---CTG	--GTGAGTAC
B.DK.04.PMVL_012.EF514699	CCAGAGGAG-	--CTCTCTCG	GCGCAGG--A	CTCGGCTTGC	TGAA------	GCGCGCACRG	CAAGAGGCGA	GGGG----CG	GCGA---CTG	--GTGAGTAC
B.DK.04.PMVL_013.EF514700	CCAGAGGAG-	--ATCTCTCG	GCGCAGG--A	CTCGGCTTGC	TGCA------	GCGCGCGTGG	CAAGAGGCGA	GGGG----CG	GCGA---ACG	--GTGAGTAC
B.DK.04.PMVL_018.EF514697	CCAGAGGAG-	--CTCTCTCG	ACGCAGG--A	CTCGGCTTGC	TGAA------	GCGCGCACAG	CAAGAGGCGA	GGGG----CG	GCGG---CTG	--GTGAGTAC
B.DK.04.PMVL_025.EF514702	CCAGAGGAG-	--ATCTCTCG	ACGCAGG--A	CTCGGCTTGC	TGAA------	GCGCGCACAG	CAAGAGGCGA	GGGG----CG	GCGA---CTG	--GTGAGTAC
B.DK.04.PMVL_027.EF514698	CCAGAGGGC-	-----TCTCG	ACGCAGG--A	CTCGGCTTGC	TGAA------	GCGCGCACGG	CAAGAGGCGA	GGGG----CG	GCGG---CTA	G-GTGAGTAC
B.DK.04.PMVL_039.EF514703	CATTTTAAA-	--AAGAAAGG	GGGGATG--G	GGGGGCTTGT	TGAA------	GGGCGAGCGG	CAAAGGGCGA	GGGG----CG	GCGG---AC-	--GTAGAGAC
B.DK.04.PMVL_049.EF514701	CCAGAGGAG-	--CTCTCTCG	ACGCAGG--A	CTCGGCTTGC	TGAA------	GCGCGCACGG	CAAGAGGCGA	GGGG----CG	GCGA---CTG	--GTGAGTAC
B.DK.07.PMVL_011.FJ694790	CCAGAGAAG-	--CTCTCTCG	ACGCAGG--A	CTCGGCTTGC	TGAA------	GCGCGCACGG	CAAGAGGCGA	GGGG----CG	GCGA---CTG	--GTGAGTAC
B.ES.05.X1890.EU786672	CCAGAGGAG-	--ATCTCTCG	ACGCAGG--A	CTCGGCTTGC	TGAA------	GCGCGCACGG	CAAGAGGCGA	GGGG----CG	GCGA---CTG	--GTGAGTAC
B.ES.06.X1959.EU786675	CCAGAGAAG-	--ATCTCTCG	ACGCAGG--A	CTCGGCTTGY	TGAA------	GMGCGCACAG	CAAGAGGCGA	GGGG----CG	GCGA---CCG	--GTGAGTAC
B.ES.06.X2102.EU786677	CCAGAGGAG-	--ATCTCTCG	ACGCAGG--A	CTCGGCTTGC	TTGA------	GCGCGCAAGG	CAAGAGGCGA	GGGG----CG	GCGA---CTG	--GTGAGTAC
B.ES.07.X2149.EU786678	CCAGAGGAG-	--CTCTCTCG	ACGCAGG--A	CTCGGCTTGC	TGAA------	GCGCGCACAG	CAAGAGGCGA	GGGG----CG	GCGA---CTG	--GTGAGTAC
B.ES.07.X2210_3.EU786679	CCAGAGAAG-	--CTCTCTCG	ACGCAGG--A	CTCGGCTTGC	TGAA------	GCGCGCACAG	CAAGAGGCGA	GGGG----CG	GCGA---CTG	--GTGAGTAC
B.ES.07.X2231.EU786680	CCAGAGGAG-	--CTCTCTCG	ACGCAGG--A	CTCGGCTTGC	TGAA------	GCGCGCACGG	CAAGAGGCGA	GGGG----CG	GCGA---CTG	--GTGAGTAC
B.ES.08.ES_X2515_3.GQ372988	CCAGAGGAG-	--ATCTCTCG	ACGCAGG--A	CTCGGCTTGC	TGAA------	GCGCGCACAG	CAAGAGGCGA	GGGG----CG	GCGA---ACG	--GTGAGTAC
B.ES.08.ES_X2556_3.GQ372990	CCGGAGAAG-	--ATCTCTCG	ACGCAGG--A	CTCGGCTTGC	TGAA------	GCGCGCACGG	CAAGAGGCGA	GGGG----CG	GCGA---CTG	--GTGAGTAC
B.ES.08.P2008.FJ670531	CCAGAGGAG-	--CTCTCTCG	ACGCAGG--A	CTCGGCTTGC	TGAA------	GCGCGCACAG	CAAGAGGCGA	GGGG----CG	GCGA---CTG	--GTGAGTAC
B.ES.08.X2425_2.FJ670525	CCAGAGGAG-	--ATCTCTCG	ACGCAGG--A	CTCGGCTTGC	TGAA------	GCGCGCACGG	CAAGAGGCGA	GGGG----CG	GCGA---CTG	--GTGAGTAC
B.ES.08.X2510_2.FJ853622	CCAGAGGAG-	--CTCTCTTG	ACGCAGG--A	CTCGGCTTGC	TGAA------	GCGCGCACGG	CAAGAGGCGA	GGGG----CG	GCGA---CTG	--GTGAGTAC
B.ES.08.X2555_2.GU362883	CCAGAGGAG-	--CTCTCTCG	ACGCAGG--A	CTCGGCTTGC	TGAA------	GCGCGCGCGG	CAAGAGGCGA	GGGG----CG	GCGA---CTG	--GTGAGTAC
B.ES.08.X2574_2.GU362886	CCAGAGGAG-	--CTCTCTCG	ACGCAGG--A	CTCGGCTTGC	TGAA------	GCGCGCACAG	CAAGAGGCGA	GGGG----CG	GCGA---CTG	--GTGAGTAC
B.ES.09.P2149_3.GU362881	CCAGAGGAG-	--AACTCTCG	ACGCAGG--A	CTCGGCTTGC	TG--------	AAGCGCACAG	CAAGAGGCGA	GGGG----CA	GCGA---CTG	--GTGAGTAC
B.ES.09.X2689_2.GU362885	CCAGAGAAG-	--CTCTCTCG	ACGCAGG--A	CTCGGCTTGC	TGAA------	GCGCGCACGG	CAAGAGGCGA	GGGG----CG	GCGA---CTG	--GTGAGTAC
B.ES.89.U61.DQ854716	CCAGAGAAG-	--CTCTCTCG	ACGCAGG--A	CTCGGCTTGC	TGAA------	GCGCGCACGG	CAAGAGGCGA	GGGGA---CG	GCGA---CTG	--GTGAGTAC
B.FR.05.DEMB05FR001.JX140652	CCAGAGGAG-	--CTCTCTCG	ACGCAGG--A	CTCGGCTTGC	TGAA------	GCGCGCACGG	CAAGAGGCGA	GGGG----CG	GCGA---CTG	--GTGAGTAC
B.FR.08.DEMB08FR002.JX140654	CCAGAGGAG-	--CTCTCTCG	ACGCAGG--A	CTCGGCTTGC	CGAA------	GCGCGCACGG	CAAGAGGCGA	GGGG----CG	GCGA---CTG	--GTGAGTAC
B.GA.88.OYI_397.M26727	CCAGAGGAG-	--CTCTCTCG	ACGCAGG--A	CTCGGCTTGC	TTAG------	-CGCGCACGG	CAAGAGGCGA	GGGG----CG	GCGA---CTG	--GTGAGTAC
B.GB.05.MM45d213_GN1.HM586212	CCAGAGAAG-	--CTCTCTCG	ACGCAGG--A	CTCGGCTTGC	TGAA------	GCGCGCACGG	CAAGAGGCGA	GGGG----CG	GCGA---CCG	--GTGAGTAC
B.GB.83.CAM1.D10112	CCGGAGGAG-	--CTCTCTCG	ACGCAGG--A	CTCGGCTTGC	TGAA------	GCGCGCACAG	CAAGAGGCGA	GGGG----CG	GCGA---CTG	--GTGAGTAC
B.GB.86.GB8_46R.AJ271445	CCAGAGGAG-	--CTCTCTCG	ACGCAAGG-A	CTCGGCTTGC	TGAA------	GCGCGCGCGG	CAAGAGGCGA	GGGG----CG	GCGA---CTG	--GTGAGTAC
B.GB.x.MANC.U23487	CCAGAGGAG-	--CTCTCTCG	ACGCAGG--A	CTCGGCTTGC	TGAA------	GCGCGCACGG	CAAGAGGCGA	GGGA----CG	GCGA---CTG	--GTGAGTAC
B.HK.06.HK003.FJ460500	CCAGAGGAG-	--CTCTCTCG	ACGCAGG--A	CTCGGCTTGC	TGAA------	GCGCGCGCGG	CAAGAGGCGA	GGGG----CG	GCGA---CCG	--GTGAGTAC
B.HK.06.HK004.FJ460501	CCAGAGGAG-	--CTCTCTCG	ACGCAGG--A	CTCGGCTTGC	TGAA------	GCGCGCGCGG	CAAGAGGCGA	GGGG----CG	GCGA---CTG	--GTGAGTAC
B.IN.x.11807.EF694037	CCAGAGGAG-	--CTCTCTCG	ACGCAGG--A	CTCGGCTTGC	TGAA------	GCGCGCACGG	CAAGAGGCGA	GGGG----CG	GCGA---CCG	--GTGAGTAC
B.IT.05.SG1.DQ672623	CCAGAGGAG-	--CTCTCTCG	ACGCAGG--A	CTCGGCTTGC	TGAA------	GCGCGCACAG	CAAGAGGCGA	GGGG----CG	GCGA---CTG	--GTGAGTAC
B.JP.00.DR2508.AB289588	CCAGAGGAG-	--CTCTCTCG	ACGCAGG--A	CTCGGCTTGC	TGAA------	GCGTGCACGG	CAAGAGGCGA	GGGG----CG	GCGA---ACG	--GTGAGTAC
B.JP.01.DR388.AB289590	CCAGAGGAG-	--CTCTCTCG	ACGCAGG--A	CTCGGCTTGC	TGAA------	GCGCGCACGG	CAAGAGGCGA	GGGG----CG	GCGA---CTG	--GTGAGTAC
B.JP.04.04JPDR6075B.AB221125	CCGGAGGAG-	--ATCTCTCG	ACGCAGG--A	CTCGGCTTGC	TGAA------	GCGCGCACAG	CAAGAGGCGA	GGGG----CG	GCGA---CTG	--GTGAGTAC
B.JP.04.DR5913.AB480696	CCAGAGGAG-	--CTCTCTCG	ACGCAGG--A	CTCGGCTTGC	TGAA------	GCGCGCACGG	CAAGAGGCGA	GGGG----CG	GCGA---CTG	--GTGAGTAC
B.JP.04.DR6089.AB286955	CCAGAGGAG-	--CTCTCTCG	ACGCAGG--A	CTCGGCTTGC	TGAA------	GCGCGCACAG	CAAGAGGCGA	GGGG----CG	GCGA---CTG	--GTGAGTAC
B.JP.04.DR6174.AB480692	CCAGAGGAG-	--CTCTCTCG	ACGCAGG--A	CTCGGCTTGC	TGAA------	GCGCGCACGG	CAAGAGGCGA	GGGG----CG	GCGA---CTG	--GTGAGTAC

B.JP.04.DR6175.AB480694	CCAGAGAAG-	--ATCTCTCG	ACGCAGG--A	CTCGGCTTGC	TGAA------	GCGCGCACGG	CAAGAGGCGA	GGGG----CG	GCGG---CTG	--GTGAGTAC
B.JP.05.DR6538.AB287363	CCAGAGGAG-	--CTCTCTCG	ACGCAGG--A	CTCGGCTTGC	TGAG------	-CGCGCACGG	CAAGAGGCGA	GGGG----CG	GCGA---CTG	--GTGAGTAC
B.JP.05.DR6737.AB287364	CCGGAGCAG-	--CTCTCTCG	ACGCAGG--A	CTCGGCTTGC	TGAA------	GCGCGCACAG	CAAGAGGCGA	GGGG----AG	GCGA---CTG	--GTGAGTAC
B.JP.05.DR7060.AB287366	CCAGAGGAG-	--ATCTCTCG	ACGCAGG--A	CTCGGCTTGC	TGAA------	GCGCGCACAG	CAAGAGGCGA	GGGG----AG	GCGA---CTG	--GTGAGTAC
B.JP.05.DR7065.AB287368	CCAGAGTAG-	--ATCTCTCG	ACGCAGG--A	CTCGGCTTGC	TGAG------	-CGCGCACAG	CAAGAGGCGA	GGGG----CG	GCGA---ACG	--GTGAGTAC
B.JP.98.DR1120.AB480698	CCAGAGGAG-	--ATCTCTCG	ACGCAGG--A	CTCGGCTTGC	TGAA------	GCGCGCGCGG	CAAGAGGCGA	GGGG----CG	GCGA---CTG	--GTGAGTAC
B.JP.99.DR1348.AB287370	CCAGAGGAG-	--CTCTCTCG	ACGCAGG--A	CTCGGCTTGC	TGAA------	GCGCGCACAG	CAAGAGGCGA	GGGG----CG	GCGA---CTG	--GTGAGTAC
B.JP.x.DR1673.AB564745	CCAGAGGAG-	--ATCTCTCG	ACGCAGG--A	CTCGGCTTGC	TGAA------	GCGCGCACAG	CAAGAGGCGA	GGGG----CG	GCGA---CTG	--GTGAGTAC
B.JP.x.DR1712.AB604946	CCAGAGGAG-	--ATCTCTCG	ACGCAGG--A	CTCGGCTTGC	TGAA------	GTGCACACGG	CAAGAGGCGA	GGGG----CG	GCGA---CTG	--GTGAGTAC
B.JP.x.DR1777.AB604948	CCAGAGGAG-	--ATCTCTCG	ACGCAGG--A	CTCGGCTTGC	TGAA------	GTGCACACGG	CAAGAGGCGA	GGGG----CG	GCGA---CTG	--GTGAGTAC
B.JP.x.JRC03B.AB565496	CCAGAGGAG-	--CTCTCTCG	ACGCAGG--A	CTCGGCTTGC	TGAA------	GCGCGCACGG	CAAGAGGCGA	GGGG----CG	GCGA---ACG	--GTGAGTAC
B.JP.x.JRC05B.AB565497	CCAGAGGAG-	--ATCTCTCG	ACGCAGG--A	CTCGGCTTGC	TGAA------	GCGCGCACAG	CAAGAGGCGA	GGGG----CG	GCGA---CTG	--GTGAGTAC
B.JP.x.JRC65B.AB565502	CCAGAGGAG-	--CTCTCTCG	ACGCAGG--A	CTCGGCTTGC	TGAA-----A	GCGCGCACAG	CAAGAGGCGA	GAG-----CG	GCGG---CTG	--GTGAGTAC
B.JP.x.pJPDR0796B02.AB565478	CCAGAGGAG-	--ATCTCTCG	ACGCAGG--A	CTCGGCTTGC	TGAA------	GCGCGCACAG	CAAGAGGCGA	GGGG----CG	GCGA---CTG	--GTGAGTAC
B.JP.x.pJRC57B09.AB641836	CCGGAGGAG-	--ATCTCTCG	ACGCAGG--A	CTCGGCTTGC	TGAA---AGC	GCG----CGG	CAAGAGGCGA	GGGG----CG	GCGA---ACG	--GTGAGTAC
B.KR.02.02OSG1.JQ429433	CCAGAGGAG-	--CTCTCTCG	ACACAGG--A	CTCGGCTTGC	TGAA------	GCGCGCACGG	CAAGAGGCGA	GGGG----CG	GCGA---CTG	--GTGAGTAC
B.KR.03.03HJY8.JQ316131	CCGGAGGAG-	--CTCTCTCG	ACGCAGG--A	CTCGGCTTGC	TGAA------	GCGCGCACGG	CAAGAGGCGA	GGGG----CG	GCGA---CTG	--GTGAGTAC
B.KR.03.03KDE11.JQ316128	CCGGAGGAG-	--CTCTCTTG	ACGCAGG--A	CTCGGCTTGC	TGAA------	GCGCGCACGG	CAAGAGGCGA	GGGG----CG	GCGA---CCG	--GTGAGTAC
B.KR.03.03KGS5.JQ316132	CCGGAGGAG-	--CTCTCTCG	ACGCAGG--A	CTCGGCTTGC	TGAA-----A	GCGCGCACCA	CAAGAGGCGA	GGGG----CG	GCGA---CTG	--GTGAGTAC
B.KR.03.03LSH1.JQ316127	CCGGAGGAG-	--CTCTCTCG	ACGCAGG--A	CTCGGCTTGC	TGAA------	CCGCGCACGG	CAAAAGGCGA	GGGG----CG	GCAA---CCG	--GTGAGTAC
B.KR.03.03YGS3.JQ316135	CCAGAGGAG-	--CTCTCTCG	ACGCAGG--A	CTCGGCTTGC	TGAA------	GCGCGCACGG	CAAGAGGCGA	GGGG----CG	GCGA---CCG	--GTGAGTAC
B.KR.04.04CWS5.JQ316133	CCAGAGGAG-	--CTCTCTCG	ACGCAGG--A	CTCGGCTTGC	TGAA------	GCGCGCACAG	CAAGAGGCGA	GAGG----CG	GCGA---CTG	--GTGAGTAC
B.KR.04.04KJS8.JQ316130	CCGGAGGAG-	--CTCTCTCG	ACGCAGG--A	CTCGGCTTGC	TGAA------	GCGCGCACGG	CAAGAGGCGA	GGGG----CG	GCGA---CTG	--GTGAGTAC
B.KR.04.04KMH5.DQ295193	CCGGAGGAG-	--CTCTCTTG	ACGCAGG--A	CTCGGCTTGC	TGAA------	GCGCGCACGG	CAAGAGGCGA	GGGG----CG	GCGA---CTG	--GTGAGTAC
B.KR.04.04KMK5.JQ316126	CCAGAGGAG-	--CTCTCTCG	ACGCAGG--A	CTCGGCTTGC	TGAA------	GCGCGCACGG	CAAGAGGCGA	GGGG----CG	GCGA---ACG	--GTGAGTAC
B.KR.04.04LHS6.AY839827	CCGGAGAAG-	--ATCTCTCG	ACGCAGG--A	CTCGGCTTGC	TGAA------	GCGCGCACGG	CAAGAGGCGA	GGGG----CG	GCGA---CTG	--GTGAGTAC
B.KR.04.04LSK7.DQ295192	CCAGAGGAG-	--CTCTCTCG	ACGCAGG--A	CTCGGCTTGC	TGAG------	-CGCGCACGG	CAAGAGGCGA	GGGG----CA	GCGA---CTG	--GTGAGTAC
B.KR.04.04WK7_HIV_1_wk.DQ29519	CCGGAGGAG-	--CTCTCTCG	ACGCAGG--A	CTCGGCTTGC	TGAA------	GCGCGCACGG	CAAGAGGCGA	GGGG----CG	GCGA---CTG	--GTGAGTAC
B.KR.05.05CSR3.DQ837381	CCAGAGGAG-	--CTCTCTCG	ACGCAGG--A	CTCGGCTTGC	TGAA------	GCGCGCACGG	CAAGAGGCGA	GGGG----CG	GCGA---CCG	--GTGAGTAC
B.KR.05.05YJN2.JQ316134	CCAGAGGAG-	--CTCTCTCG	ACGCAGG--A	CTCGGCTTGC	TGAA------	GCGCGCACAG	CAAGAGGCGA	GGGG----CG	GCGA---CCG	--GTGAGTAC
B.KR.07.07KYY4.JQ341411	CCGGAGGAG-	--CTCTCTCG	ACGCAGG--A	CTCGGCTTGC	TGAA------	GCGCGCACGG	CAAGAGGCGA	GGGG----CG	GCGA---CTG	--GTGAGTAC
B.KR.99.99HYH2.JQ316129	CCAGAGGAG-	--CTCTCTCG	ACGCACG--A	CTCGGCTTGC	TGA-------	GCGCGCACGG	CAAGAGGCGA	GGGG----CG	GCGA---CCG	--GTGAGTAC
B.MM.99.mSTD101.AB097870	CCAGAGAAG-	--ATCTCTCG	ACGCAGG--A	CTCGGCTTGC	TGAA-----A	GCGCGCGCAG	CAAGAGGCGA	GAG-----CG	GCGG---CTG	--GTGAGTAC
B.NL.00.671_00T36.AY423387	CCAGAGGAG-	--ATCTCTCG	ACGCAGG--A	CTCGGCTTGC	TGAAGAAGAA	GCGCGCGCGG	CAAGAGGCGA	GGGG----CG	GCGA---CTG	--GTGAGTAC
B.NL.86.3202A21_ACH3202A21.U34	CCAGAGGAG-	--CTCTCTCG	ACGCAGG--A	CTCGGCTTGC	TGAA------	GCGCGCACGC	CAAGAGGCGA	GGGG----CG	GCGG---CTG	--GTGAGTAC
B.NL.96.H434_42_A1.AY970948	CCAGAGGAG-	--CTCTCTTG	ACGCAGG--A	CTCGGCTTGC	TGAA------	GCGCGCGCGG	CAAGAGGCGA	GGGG----CG	GCGG---CTG	--GTGAGTAC
B.PE.06.502_0491_wg5.JF320183	CCAGAGGAG-	--CTCTCTCG	ACGCAGG--A	CTCGGCTTGC	TGAA------	GCGCGCACGG	CAAGCGGCGA	GGGG----CG	GCGAA--CTG	--GTGAGTAC
B.PE.06.502_0524_FL04.JF320008	CCAGAGAAG-	--CTCTCTTG	ACGCAGG--A	CTCGGCTTGC	TGAA------	GTGCGCACGG	CAAGAGGCGA	GGGG----CG	GCGA---CTG	--GTGAGTAC
B.PE.06.502_0648_FL02.JF320215	CCAGAGGAG-	--CTCTCTCG	ACGCAGG--A	CTCGGCTTGC	TGAA------	GCGCGCACAG	CAAGAGGCGA	GGGG----CG	GCGA---CTG	--GTGAGTAC
B.PE.06.502_0841_FL04.JF320208	CCAGAGGAA-	--ATCTCTCG	ACGCAGG--A	CTCGGCTTGC	TGAA------	GCGCGCGCGG	CAAGAGGCGA	GGGG----CG	GCGA---CTG	--GTGAGTAC
B.PE.06.502_2794_FL05.JF320244	CCGGAGGAG-	--CTCTCTCG	ACGCAAGG-A	CTCGGCTTGC	TGAA------	GCGCGCGCGG	CAAGAGGCGA	GGGG----CG	GCGA---CTG	--GTGAGTAC
B.PE.07.502_0525_wg5.JF320191	CCAGAGAAG-	--CTCTCTTG	ACGCAGG--A	CTCGGCTTGC	TGAA------	GCGCGCACGG	CAAGAGGCGA	GAGG----CG	GCGG---CTG	--GTGAGTAC
B.PE.07.502_1047_wg5.JF320226	CCAGAGAGA-	--ATCTCTCG	ACGCAGG--A	CTCGGCTTGC	TGAA------	GCGCGCACGG	CAAGAGGCGA	GAG-----CG	GCGA---ACG	--GTGAGTAC
B.PE.07.502_1399_wg4.JF320013	CCAGAGGAG-	--CTCTCTCG	ACGCAGG--A	CTCGGCTTGC	TGAA------	GCGCGCACAG	CAAGAGGCGA	GGGG----CG	GCGA---CTG	--GTGAGTAC
B.PE.07.502_2254_FL6.JF320018	CCAGAGGAG-	--AACTCTCG	ACGCAGG--A	CTCGGCTTGC	TGAA------	GCGCGCACAG	CAAGAGGCGA	GGGG----CG	GCGA---CTG	--GTGAGTAC
B.PE.07.502_2349_wg2.JF320028	CCAGAGGAG-	--CTCTCTCG	ACGCAGG--A	CTCGGCTTGC	TGCA------	GCGCGCACAG	CAAGAGGCGA	GGGG----CG	GCGA---CCG	--GTGAGTAC
B.PE.07.502_2622_wg1.JF320189	CCAGAGGAG-	--CTCTCTTG	ACGCAGG--A	CTCGGCTTGC	TGAA------	GCGCGCACAG	CAAGAGGCGA	GGGG----CG	GCGA---CTG	--GTGAGTAC
B.PE.07.502_2649_wg8.JF320019	CCAGAGGAG-	--CTCTCTCG	ACGCAGG--A	CTCGGCTTGC	TGA-------	GCGCGCACGG	CAAGAGGCGA	GAGG----CG	GCGG---CTG	--GTGAGTAC
B.RU.04.04RU128005.AY682547	CCAGAGGAG-	--CTCTCTCG	ACGCAGG--A	CTCGGCTTGC	TGAA------	GTGCGCACAG	CAAGAGGCGA	GGGG----CG	GCGA---CTG	--GTGAGTAC
B.RU.04.04RU129005.AY751406	CCAGAGGAG-	--CTCTCTCG	ACGCAGG--A	CTCGGCTTGC	TGAA------	GCGCGCACGG	CAAGAGGCGA	GGGG----CG	GCGA---CTG	--GTGAGTAC
B.RU.04.04RU139089.AY751407	CCAGAGAAG-	--CTCTCTCG	ACGCAGG--A	CTCGGCTTGC	TGAA------	GCGCGCACAG	CAAGAGGCGA	GGGG----CG	GCGA---CTG	--GTGAGTAC
B.RU.04.04RU139095.AY819715	CCAGAGAAG-	--CTCTCTCG	ACGCAGG--A	CTCGGCTTGC	TGAA------	GCGCGCACGG	CAAGAGGCGA	GGGG----CG	GCGA---CTG	--GTGAGTAC
B.RU.09.09RU4457.JX500709	CCAGAGAAG-	----CTCTCG	ACGCAGG--A	CTCGGCTTGC	TGAA------	GTGCGCACGG	CAAGAGGCGA	GGGG----CG	GCGA---CTG	--GTGAGTAC
B.RU.10.10RU6629.JX500707	CCAGAGGAG-	--CTCTCTCG	ACGCAGG--A	CTCGGCTTGC	TGAA------	GTGCGCACGG	CAAGAGGCGA	GGGG----CG	GCGA---CTG	--GTGAGTAC
B.RU.11.11RU21n.JX500708	CCAGAGGAG-	----CTCTTG	ACGCAGG--A	CTCGGCTTGC	TGAA------	GTGCGCACGG	CAAGAGGCGA	GGGG----CG	GCGA---CTG	--GTGAGTAC
B.TH.06.AA010a_WG3.JX446800	CCAGAGAAG-	----CTCTCG	ACGCAGG--A	CTCGGCTTGC	TGAA------	GTGCGCACGG	CAAGAGGCGA	GGGG----CG	GCGA---CCG	--GTGAGTAC
B.TH.07.AA040a_WG11.JX447156	CCAGAGGAG-	--CTCTCTCG	ACGCAGG--A	CTCGGCTTGC	TGAA------	GCGCGCACAG	CAAGAGGCGA	GAG-----CG	GCGA---CTG	--GTGAGTAC
B.TH.90.BK132.AY173951	CCAGAGGAG-	--CTCTCTCG	ACGCAGG--A	CTCGGCTTGC	TGAA------	GCGCGCACGG	CAAGAGGCGA	GGGG----CG	GCGA---CTG	--GTGAGTAC
B.TW.94.TWCYS_LM49.AF086817	CCAGAGGAG-	--ATCTCTCG	ACGCAGG--A	CTCGGCTTGC	TGAA------	GTGCGCACGG	CAAGAGGCGA	GGGG----CG	GCGG---CTG	--GTGAGTAC
B.US.00.14294_1.DQ853436	CCAGAGAAG-	--ATCTCTCG	ACGCAGG--A	CTCGGCTTGC	TGAA------	GCGCGCACGG	CAAGAGGCGA	GGGG----CG	GCGA---CTG	--GTGAGTAC
B.US.00.ES1_20.EF363123	CCAGAGGAG-	--CTCTCTCG	ACGCAGG--A	CTCGGCTTGC	TGAA------	GCGCGCACAG	CAAGAGGCGA	GGGG----CG	GCGA---CTG	--GTGAGTAC
B.US.00.RHPA_TF1.JN944917	CCAGAGGAG-	--CTCTCTCG	ACGCAGG--A	CTCGGCTTGC	TGAA------	GCGCGCACGG	CAAGAGGCGA	GGGG----CG	GCGA---CTG	--GTGAGTAC
B.US.00.THRO_TF1.JN944930	CCAGAGGAG-	--ATCTCTCG	ACGCAGG--A	CTCGGCTTGC	TGAA------	GCGCGCACGG	CAAGAGGCGA	GGGG----CG	GCGA---CCG	--GTGAGTAC
B.US.00.WITO_flB3.FJ496169	CCAGAGGAG-	--ATCTCTCG	ACGCAGG--A	CTCGGCTTGC	TGAA------	GCGCGCACAG	CAAGAGGCGA	GGGG----CA	GCGA---CTG	--GTGAGTAC
B.US.01.REJO_TF1.JN944911	CCAGAGGAG-	--CTCTCTCG	ACGCAGG--A	CTCGGCTTGC	TGAA------	GCGCGCACGG	CAAGAGGCGA	GGGG----CG	GCGA---CTG	--GTGAGTAC
B.US.01.TRJO_flA1.FJ496151	CCAGAGGAG-	--CTCTCTCG	ACGCAGG--A	CTCGGCTTGC	TGAA------	GCGCGCGCGG	CAAGAGGCGA	GGGG----CG	GCGA---CTG	--GTGAGTAC
B.US.02.04013226_2_flH11.FJ496	CCAGAGGAG-	--ATCTCTCG	ACGCAGG--A	CTCGGCTTGC	TGAA------	ACGCGCGCGG	CAAGAGGCGA	GGGGTA--CG	GCGA---CTG	--GTGAGTAC
B.US.04.ES10_53.EF363127	CCAGAGAAG-	--ATCTCTCG	ACGCAGG--A	CTCGGCTTGC	TGAA------	GCGCGCACGG	CAAGAGGCGA	GGGG----CG	GCGA---CCG	--GTGAGTAC
B.US.04.ES4_24.EF363124	CCAGAGGAG-	--CTCTCTCG	ACGCAGG--A	CTCGGCTTGC	TGAA------	GCGCGCACGG	CAAGAGGCGA	GGGG----CG	GCGA---CTG	--GTGAGTAC
B.US.04.ES8_43.EF363126	CCAGAGGAG-	--CTCTCTCG	ACGCAGG--A	CTCGGCTTGC	TGAA------	GCGCGCACGG	CAAGAGGCGA	GAGG----CG	GCGA---CTG	--GTGAGTAC
B.US.04.SAMI_WGA1.EU547186	CCAGAGGAG-	--ATCTCTCG	ACGCAGG--A	CTCGGCTTGC	TGAA------	GCGCGCACGG	CAAGAGGCGA	GGGG----CG	GCGA---CCG	--GTGAGTAC
B.US.04.USPI71101EI7y04051pcWG	CCAGAGGAG-	--CTCTCTCG	ACGCAGG--A	CTCGGCTTGC	TGAA------	GCGCGCACGG	CAAGAGGCGA	GGGG----CG	GCGA---CTG	--GTGAGTAC
B.US.04.USPI83747EI6y04121pcWG	CCAGAGGAG-	--CTCTCTCG	ACGCAGG--A	CTCGGCTTGC	TGAA------	GCGCGCACAG	CAAGAGGCGA	GGGG----CG	GCGA---CTG	--GTGAGTAC
B.US.05.04013396_0_flE6.FJ4960	CCAGAGAAG-	--TTCTCTCG	ACGCAGG--A	CTCGGCTTGC	TGAA------	GCGCGCTCGG	CAAGAGGCGA	GGGG-T--CG	GCGA---CTG	--GTGAGTAC
B.US.05.502_0223_wg1.JF320059	CCAGAGGAG-	--ATCTCTCG	ACGCAGG--A	CTCGGCTTGC	TGAA------	GCGCGCACAG	CAAGAGGCGA	GGGG----CG	GCGA---ACG	--GTGAGTAC
B.US.05.502_1400_FL02.JF320043	CCAGAGAAG-	--CTCTCTCG	ACGCAGG--A	CTCGGCTTGC	TGAA------	GCGCGCACAG	CAAGAGGCGA	GGGG----CG	GCGA---CTG	--GTGAGTAC
B.US.05.502_2136_FL02.JF320185	CCAGAGGAG-	--ATCTCTCG	ACGCAGG--A	CTCGGCTTGC	TGAA------	GCGCGCACAG	CAAGAGGCGA	GGGG----CG	GCGA---CCG	--GTGAGTAC
B.US.05.502_2495_wg02.JF320054	CCAGAGAAG-	--CTCTCTCG	ACGCAGG--A	CTCGGCTTGC	TGAA------	GTGCGCACGG	CAAGAGGCGA	GGGG----CG	GCGA---CTG	--GTGAGTAC
B.US.05.CR0307R.FJ469717	CCAGAGGAG-	--ATCTCTCG	ACGCAGG--A	CTCGGCTTGC	TGAA------	GCGCGCGCGG	CAAGAGGCGA	GGGG----TG	GCGA---CTG	--GTGAGTAC
B.US.05.USPI38417EI33y05051pcW	CCAGAGGAG-	--CTCTCTCG	ACGCAGG--A	CTCGGCTTGC	TGAA------	GTGCGCACGG	CAAGAGGCGA	GGGG----CG	GCGA---CCG	--GTGAGTAC
B.US.05.USPI88403EI14y05121pcW	CCAGAGGAG-	--CTCTCTCG	ATGCAGG--A	CTCGGCTTGC	TGAA------	GCGCGCACAG	CAAGAGGCGA	GGGG----CG	GCGA---CTG	--GTGAGTAC
B.US.06.502_0341_FL05.JF320003	CCAGAGGAG-	--ATCTCTCG	ACGCAGG--A	CTCGGCTTGC	TGAA------	GCGCGCACGG	CAAGAGGCGA	GGGG----CG	GCGA---CCG	--GTGAGTAC
B.US.06.502_1046_FL04.JF320564	CCAGAGAAG-	--ATCTCTCG	ACGCAGG--A	CTCGGCTTGC	TGAA------	GCGCGCACAG	CAAGAGGCGA	GGGG----CG	GCGA---CTG	--GTGAGTAC
B.US.06.502_1174_FL09.JF320053	CCAGAGAAG-	--ATCTCTCG	ACGCAGG--A	CTCGGCTTGC	TGAA------	GCGCGCACAG	CAAGAGGCGA	GGGG----CG	GCGA---CTG	--GTGAGTAC
B.US.06.502_1211_FL01.JF320151	CCAGAGGAG-	--ATCTCTCG	ACGCAGG--A	CTCGGCTTGC	TGAA------	GCGCGCTCGG	CAAGAGGCGA	GGGG----CG	GCGA---CCG	--GTGAGTAC
B.US.06.502_1619_FL06.JF320126	CCAGAGCAG-	--CTCTCTCG	ACGCAGG--A	CTCGGCTTGC	TGAA------	GCGCGCACAG	CAAGAGGCGA	GGGG----CG	GCGA---ACG	--GTGAGTAC
B.US.06.502_2667_FL03.JF320145	CCAGAAGAG-	--CTCTCTCG	ACGCAGG--A	CTCGGCTTGC	TGAA------	GCGCGCACGG	CAAGAGGCGA	GGGG----CG	GCGA---CTG	--GTGAGTAC
B.US.06.CH106_TF1.JN944897	CCAGAGGAG-	--ATCTCTCG	ACGCAGG--A	CTCGGCTTGC	TGAA------	GCGCGCACGG	CAAGAGGCGA	GGGG----CG	GCGA---CTG	--GTGAGTAC
B.US.06.CH40E_flA1.FJ495818	CCAGAGGAG-	--CTCTCTCG	ACGCAGG--A	CTCGGCTTGC	TGAA------	GCGCGCACGG	CAAGAGGCGA	GGGG----CG	GCGA---CTG	--GTGAGTAC
B.US.06.CH58E_flA9.FJ495941	CCGGAGGAG-	--CTCTCTCG	ACGCAGG--A	CTCGGCTTGC	TGAA------	GCGCGCACGG	CAAGAGGCGA	GGGG----CG	GCGA---CTG	--GTGAGTAC
B.US.06.CH77E_flA1.FJ496000	CCAGAGAAG-	--TTCTCTCG	ACGCAGG--A	CTCGGCTTGC	TGAA------	GCGCGCACAG	CAAGAGGCGA	GGGG----AG	GCGA---CTG	--GTGAGTAC
B.US.07.502_0364_wg2.JF320563	CCAGAGGAG-	--ATCTCTCG	ACGCAGG--A	CTCGGCTTGC	TGAA------	GCGCGCACAG	CAAGAGGCGA	GGGG----CG	GCGG---CTG	--GTGAGTAC
B.US.07.502_1115_wg1.JF320045	CCAGAGGAG-	--CTCTCTCG	ACGCAGG--A	CTCGGCTTGC	TGAA------	GCGCGCACAG	CAAGAGGCGA	GAGG----CG	GCGA---CTG	--GTGAGTAC
B.US.07.502_1478_wg4.JF320150	CCGGAGGAG-	--ATCTCTCG	ACGCAGG--A	CTCGGCTTGC	TGAA------	GCGCGCACAG	CAAGAGGCGA	GGGG----CG	GCGA---CTG	--GTGAGTAC
B.US.07.502_1897_wg6.JF320182	CCAGAGGAG-	--ATCTCTCG	ACGCAGG--A	CTCGGCTTGC	TGAA------	GTGCGCACAG	CAAGAGGCGA	GGGG----CG	GCGA---CCG	--GTGAGTAC
B.US.07.502_2289_05.JF320197	CCAGAGAAG-	--CTCTCTCG	ACGCAGG--A	CTCGGCTTGC	TGAA------	GCGCGCACGG	CAAGAGGCGA	GGGG----CG	GCGA---CTG	--GTGAGTAC
B.US.09.C1P.GU733713	CCAGAGGAG-	--ATCTCTCG	ACGCAGG--A	CTCGGCTTGC	TGAA------	GCGCGCACAG	CAAGAGGCGA	GGGG----CG	GCGA---CTG	--GTGAGTAC
B.US.09.DEMB09US002.JX140657	CCGGAGGAG-	--ATCTCTCG	ACGCAGG--A	CTCGGCTTGC	TGAA------	GCGCGCACAG	CAAGAGGCGA	GGGG----CG	GCGA---CTG	--GTGAGTAC
B.US.10.CP1.JN397365	CCAGAGGAG-	--CTCTCTCG	ACGCAGG--A	CTCGGCTTGC	TGA-------	GCGCGCACAG	CAAGAGGCGA	GGGG----CG	GCGA---CTG	--GTGAGTAC
B.US.10.VC1.JN397364	CCAGAGGAG-	--ATCTCTCG	ACGCAGG--A	CTCGGCTTGC	TGA-------	GCGCGCACAG	CAAGAGGCGA	GGGG----CG	GCGA---CTG	--GTGAGTAC
B.US.11.ES38.JN397362	CCAGAGGAG-	--CTCTCTCG	ACGCAGG--A	CTCGGCTTGC	TGAA-----A	GCGCGCACGG	CAAGAGGCGA	GGAG----CG	GCGA---CTG	--GTGAGTAC
B.US.83.5018_83.AY835777	CCAGAGGAG-	--ATCTCTCG	ACGCAGG--A	CTCGGCTTGC	TGAA------	GCGCGCACGG	CAAGAGGCGA	GGGG----CG	GCGA---CTG	--GTGAGTAC
B.US.83.5157_83.AY835781	CCAGAGGAG-	--ATCTCTCG	ACGCAGG--A	CTCGGCTTGC	TGAA------	GCGCGCACGG	CAAGAGGCGA	GGGG----CG	GCGA---CTG	--GTGAGTAC
B.US.83.RF_HAT3.M17451	CCAGAGGAG-	--ATCTCTCG	ACGCAGG--A	CTCGGCTTGC	TGAA------	GCGCGCGCGG	CAAGAGGCGA	GGGG----CG	GCGA---ACG	--GTGAGTAC
B.US.83.SF2_LAV2_ARV2.K02007	CCAGAGGAG-	--CTCTCTCG	ACGCAGG--A	CTCGGCTTGC	TGAA------	GCGCGCACAG	CAAGAGGCGA	GGGG----CG	GCGA---CTG	--GTGAGTAC

B.US.84.5019_84.AY835779	CCAGAGGAG-	--ATCTCTCG	ACGCAGG--A	CTCGGCTTGC	TGAA------	GCGCGCACGG	CAAGAGGCGA	GGGG----CG	GCGA---CTG	--GTGAGTAC
B.US.84.MNCG_MN.M17449	CCAGAG----	--CTCTCTCG	ACGCAGG--A	CTCGGCTTGC	TGAA------	GCGCGCACGG	CAAGAGGCGA	GGGG----CG	GCGA---CTG	--GTGAGTAC
B.US.84.NY5CG.M38431	CCAGAGGAG-	--ATCTCTCG	ACGCAGG--A	CTCGGCTTGC	TGAA------	GCGCGCACGG	CAAGAGGCGA	GGGG----CG	GCGA---CTG	--GTGAGTAC
B.US.84.SF33.AY352275	CCAGAGGAG-	--CTCTCTCG	ACGCAGG--A	CTCGGCTTGC	TGAA-----A	GCGCGCACGG	CAAGAGGCGA	GGGG----CG	GCGA---CTG	--GTGAGTAC
B.US.85.5077_85.AY835769	CCAGAGGAG-	--ATCTCTCG	ACGCAGG--A	CTCGGCTTGC	TGAA------	GCGCGCACGG	CAAGAGGCGA	GGGG----CG	GCGA---CTG	--GTGAGTAC
B.US.85.Ba_L.AB221005	CCAGAGGAG-	--CTCTCTCG	ACGCAGG--A	CTCGGCTTGC	TGAA------	--GCGCACGG	CAAGAGGCGA	GGGG----CG	GCGA---CTG	--GTGAGTAC
B.US.86.5084_86.AY835775	CCAGAGGAG-	--ATCTCTCG	ACGCAGG--A	CTCGGCTTGC	TGAA------	GCGCGCACGG	CAAGAGGCGA	GGGG----CG	GCGA---CTG	--GTGAGTAC
B.US.86.5096_86.AY835749	CCAGAGGAG-	--CTCTCTCG	ACGCAGG--A	CTCGGCTTGC	TGAA------	GCGCGCGCAG	CAAGAGGCGA	GGGG----CG	GCGA---CTG	--GTGAGTAC
B.US.86.5127_86.AY835774	CCAGAGGAG-	--ATCTCTCG	ACGCAGG--A	CTCGGCTTGC	TGAA------	GCGCGCACGG	CAAGAGGCGA	GGGG----CG	GCGA---CTG	--GTGAGTAC
B.US.86.AD87_ADA.AF004394	CCAGAGAAG-	--CTCTCTCG	ACGCAGG--A	CTCGGCTTGC	TGAA------	GCGCGCACGG	CAAGAGGCGA	GGGG----CG	GCGA---CTG	--GTGAGTAC
B.US.86.JRFL_JR_FL.U63632	------GAG-	--CTCTCTCG	ACGCAGG--A	CTCGGCTTGC	TGAA------	GCGCGCACGG	CAAGAGGCGA	GGGG----CG	GCGA---CTG	--GTGAGTAC
B.US.86.YU_2.M93258	CCAGAGGAG-	--CTCTCTCG	ACGCAGG--A	CTCGGCTTGC	TGAA------	GCGCGCACGG	CAAGAGGCGA	GGGG----CG	GCGA---CTG	--GTGAGTAC
B.US.87.5113_87.AY835758	CCAGAGGAG-	--ATCTCTCG	ACGCAGG--A	CTCGGCTTGC	TGAA------	GCGCGCACGG	CAAGAGGCGA	GGGG----CG	GCGA---CTG	--GTGAGTAC
B.US.87.BC_BCSG3.L02317	CCAGAGGAG-	--CTCTCTCG	ACGCAGG--A	CTCGGCTTGC	TGAA------	GCGCGCACGG	CAAGAGGCGA	GGGG----CG	GCGA---CTG	--GTGAGTAC
B.US.88.5160_88.AY835763	CCAGAGGAG-	--ATCTCTCG	ACGCAGG--A	CTCGGCTTGC	TGAA------	GCGCGCACGG	CAAGAGGCGA	GGGG----CG	GCGA---CTG	--GTGAGTAC
B.US.88.WR27.AF286365	CCAGAGGAG-	--ATCTCTCG	ACGCAGG--A	CTCGGCTTGC	TGAA------	GCGCGCGCGG	CAAGAGGCGA	GGGG----CG	GCGA---CTG	--GTGAGTAC
B.US.89.P896_89_6.U39362	CCAGAGGAG-	--ATCTCTCG	ACGCAGG--A	CTCGGCTTGC	TGAA------	GCGCGCACAG	CAAGAGGCGA	GGGG----CG	GCGA---CTG	--GTGAGTAC
B.US.90.US1.AY173952	CCAGAGGAG-	--ATCTCTCG	ACGCAGG--A	CTCGGCTTGC	TGAA------	GCGCGCG--G	CAAGAGGCGA	GGGG----CG	GCGA---CTG	--GTGAGTAC
B.US.90.US2.AY173953	CCAGAGGAG-	--CTCTCTCG	ACGCAGG--A	CTCGGCTTGC	TGAA------	GCGCGCACGG	CAAGAGGCGA	GGGG----CG	GCGA---CCG	--GTGAGTAC
B.US.90.US3.AY173954	CCGGAGGAG-	--CTCTCTCG	ACGCAGG--A	CTCGGCTTGC	TGAA------	GCGCGCACAG	CAAGAGGCGA	GGGG----CG	GCGA---CTG	--GTGAGTAC
B.US.90.US4.AY173955	CCAGAGAAG-	--TTCTCTCG	ACGCAGG--A	CTCGGCTTGC	TGAA------	GTGCGCACGG	CAAGAGGCGA	GGGG----CG	GCGA---CCG	--GTGAGTAC
B.US.90.WCIPR.U69591	CCAGAGGAG-	--CTCTCTCG	ACGCAGG--A	CTCGGCTTGC	TGAA------	GCGCGCACGG	CAAGAGGCGA	GGGG----CG	GCGG---CTG	--GTGAGTAC
B.US.90.WEAU160_GHOSH.U21135	CCAGAGGAG-	--CTCTCTCG	ACGCAGG--A	CTCGGCTTGC	TGAA------	GCGCGCACGG	CAAGAGGCGA	GGGG----CG	GCGA---CTG	--GTGAGTAC
B.US.91.5048_91.AY835761	CCAGAGGAG-	--ATCTCTCG	ACGCAGG--A	CTCGGCTTGC	TGAA------	GCGCGCACGG	CAAGAGGCGA	GGGG----CG	GCGA---CTG	--GTGAGTAC
B.US.91.DH12_3.AF069140	CCAGAGAAG-	--CTCTCTCG	ACGCAGG--A	CTCGGCTTGC	TGAA------	GCGCGCACGG	CAAGAGGCGA	GGGG----CG	GCGA---ACG	--GTGAGTAC
B.US.91.SUMA_flC5.FJ496145	CCAGAGGAG-	--CTCTCTCG	ACGCAGG--A	CTCGGCTTGC	TGAA------	GCGCGCACGG	CAAGAGGCGA	GGGG----CG	GCGA---CTG	--GTGAGTAC
B.US.93.WCD32P0793.DQ487188	CCAGAGGAG-	--CTCTCTCG	ACGCAGG--A	CTCGGCTTGC	TGCA------	GCGCGCACGG	CAAGAGGCGA	GGGG----CG	GCGA---CTG	--GTGAGTAC
B.US.93.WCM32P0793.DQ487190	CCAGAGGAG-	--CTCTCTCG	ACGCAGG--A	CTCGGCTTGC	TGAA------	GCGCGCACAG	CAAGAGGCGA	GGGG----CG	GCGA---CTG	--GTGAGTAC
B.US.94.5082_94.AY835773	CCAGAGGAG-	--ATCTCTCG	ACGCAGG--A	CTCGGCTTGC	TGAA------	GCGCGCACGG	CAAGAGGCGA	GGGG----CG	GCGA---CTG	--GTGAGTAC
B.US.95.5073_95.AY835768	CCAGAGGAG-	--ATCTCTCG	ACGCAGG--A	CTCGGCTTGC	TGAA------	GCGCGCACGG	CAAGAGGCGA	GGGG----CG	GCGA---CTG	--GTGAGTAC
B.US.95.USPI90770EI72y95091pcW	CCAGAGAAG-	--CTCTCTTG	ACGCAGG--A	CTCGGCTTGC	TGAA------	GCGCGCACGG	CAAGAGGCGA	GGGG----CG	GCGA---CTG	--GTGAGTAC
B.US.96.5155_96.AY835753	CCAGAGGAG-	--ATCTCTCG	ACGCAGG--A	CTCGGCTTGC	TGAA------	GCGCGCACGG	CAAGAGGCGA	GGGG----CG	GCGA---CTG	--GTGAGTAC
B.US.96.USPI55751EI32y96071pcW	CCAGAGGAG-	--CTCTCTCG	ACGCAGG--A	CTCGGCTTGC	TGAA------	GCGCGCACAG	CAAGAGGCGA	GGGG----CG	GCGA---CTG	--GTGAGTAC
B.US.97.ARES2.AB078005	CCAGAGGAG-	--ATCTCTCG	ACGCAGG--A	CTCGGCTTGS	TGAA------	GCGCGCACGG	CAAGAGGCGA	GGGG----CG	GCGA---CTG	--GTGAGTAC
B.US.98.15384_1.DQ853463	CCAGAGAAG-	--ATCTCTCG	ACGCAGG--A	CTCGGCTTGC	TGAA------	GCGCGCACGG	CAAGAGGCGA	GGGG----CG	GCGA---CTG	--GTGAGTAC
B.US.98.98USHVTN1925c1.AY56010	CCAGAGGAG-	--CTCTCTCG	ACGCAGG--A	CTCGGCTTGC	TGAA------	GCGCGCACAG	CAAGAGGCGA	GGGG----CG	GCGA---ACG	--GTGAGTAC
B.US.98.98USHVTN3605c9.AY56010	CCAGAGGAG-	--CTCTCTCG	ACGCAGG--A	CTCGGCTTGC	TGAG------	-CGCGCACGG	CAAGAGGCGA	GAGG----CG	GCGG---CTG	--GTGAGTAC
B.US.98.98USHVTN8229c6.AY56010	CCAGAGGAG-	--ATCTCTCG	ACGCAAGG-A	CTCGGCTTGC	TGCA------	GCGCGCACGG	CAAGAGGCGA	GGGG----CG	GCGA---CTG	--GTGAGTAC
B.US.98.98USHVTN941c1.AY560110	CCAGAGGAG-	--CTCTCTCG	ACGCAGG--A	CTCGGCTTGC	TGAA------	GTGCGCACGG	CAAGAGGCGA	GGGG----CG	GCGA---CTG	--GTGAGTAC
B.US.98.WC3_0498_4.EF175212	CCAGAGGAG-	--CTCTCTCG	ACGCAGG--A	CTCGGCTTGC	TGAA------	GCGCGCACAG	CAAGAGGCGA	GGGG----CG	GCGA---CTG	--GTGAGTAC
B.US.x.CR0059T.FJ469694	CCAGAGGAG-	--CTCTCTCG	ACGCAGG--A	CTCGGCTTGC	TGAA------	GCGCGCACAG	CAAGAGGCGA	GGGG----CG	GCGA---CTG	--GTGAGTAC
B.US.x.sample_C_BID_D617.JX503	CCAGAGAAG-	----CTCTCG	ACGCGAGG-A	CTCGGCTTGC	TGAATT----	GCGCGCACAG	CAAGAGGCGA	GGGG----CG	GCGA---ACG	--GTGAGTAC
B.VE.10.DEMB10VE001.JX140659	CCAGAGGAG-	--CTCTCTCG	ACGCAGG--A	CTCGGCTTGC	TGAA------	GCGCGCACAG	CAAGAGGCGA	GGGG----CG	GCGA---CTG	--GTGAGTAC
B.ZA.03.03ZAPS045MB2.DQ396398	CCAGAGGAG-	--CTCTCTCG	ACGCAGG--A	CTCGGCTTGC	TGAA------	GCGCGCACAG	CAAGAGGCGA	GGGG----CG	GCGG---CTG	--GTGAGTAC
C.BR.02.02BR2022.JN692434	CCAGAGAAG-	--ATCTCTCG	ACGCAGG--A	CTCGGCTTGC	TGAA-----A	GTGCACTCGG	CAAGAGGCGA	GAG-----CG	GCGG---CTG	--GTGAGTAC
C.BR.04.04BR013.AY727522	CCAGAGGAG-	--ATCTCTCG	ACGCAGG--A	CTCGGCTTGC	TGAA------	GTGCACTCGG	CAAGAGGCGA	GAG-----CG	GCGG---CTG	--GTGAGTAC
C.BR.04.04BR021.AY727523	CCAGAGGAG-	--ATCTCTCG	ACGCAGG--A	CTCGGCTTGC	TGAA------	GTGCACTCGG	CAAGAGGCGA	GAG-----CG	GCGA---CTG	--GTGAGTAC
C.BR.04.04BR038.AY727524	CCAGAGGAG-	--AACTCTCG	ACGCAGG--A	CTCGGCTTGC	TGAA------	GTGCACTCAG	CAGGAGGCGA	GAG-----CG	GCGG---CTG	--GTGAGTAC
C.BR.04.04BR073.AY727525	CCAGAGGAG-	--ATCTCTCG	ACGCAGG--A	CTCGGCTTGC	TGAA------	GTGCACTCGG	CAAGAGGCGA	GAG-----CG	GCGG---CTG	--GTGAGTAC
C.BR.07.DEMC07BR003.JX140663	CCAGAGAAG-	--ATCTCTCG	ACGCAGG--A	CTCGGCTTGC	TGAA------	GTGCACACGG	CAAGAGGCGA	GAG-----CG	GCGG---CTG	--GTGAGTAC
C.BR.92.BR025_d.U52953	CCAGAGGAG-	--ATCTCTCG	ACGCAGG--A	CTCGGCTTGC	TGAA------	GTGCACTCGG	CAAGAGGCGA	GAG-----CG	GCGG---CTG	--GTGAGTAC
C.BR.98.98BR004.AF286228	CCAGAGGAG-	--ATCTCTCG	ACGCAGG--A	CTCGGCTTGC	TGAA------	GTGCACTCGG	CAAGAGGCGA	GAG-----CG	GCGA---CTG	--GTGAGTAC
C.BW.00.00BW07621.AF443088	CCAGAGGAG-	--ATCTCTCG	ACGCAGG--A	CTCGGCTTGC	TGAA------	GTGCACTCGG	CAAGAGGCGA	GAG-----CG	GCGA---CTG	--GTGAGTAC
C.BW.00.00BW076820.AF443089	CCAGAGGAG-	--ATCTCTCG	ACGCAGG--A	CTCGGCTTGC	TGAA------	GTGCACTTGG	CAAGAGGCGA	GAG-----CG	GCGG---CTG	--GTGAGTAC
C.BW.00.00BW087421.AF443090	CCAGAGGAG-	--ATCTCTCG	ACGCAGG--A	CTCGGCTTGC	TGAA------	GTGCACTCGG	CAAGAGGCGA	GAG-----GG	GCGG---CTG	--GTGAGTAC
C.BW.00.00BW147127.AF443091	CCAGAGAAG-	--ATCTCTCG	ACGCAGG--A	CTCGGCTTGC	TGAA------	GTGCACTCGG	CAAGAGGCGA	GAG-----CG	GCGG---CTG	--GTGAGTAC
C.BW.00.00BW16162.AF443092	CCAGAGGAG-	--ATCTCTCG	ACGCAGG--A	CTCGGCTTGC	TGAA-----G	TGCACTCCGG	CAAGAGGCGA	GAG-----CG	GCGA---CTG	--GTGAGTAC
C.BW.00.00BW1686.AF443093	CCAGAGAAG-	--ATCTCTCG	ACGCAGG--A	CTCGGCTTGC	TGAA------	GTGCACTCGG	CAAGAGGCGA	GAGGAG--CG	GCGG---CTG	--GTGAGTAC
C.BW.00.00BW17593.AF443094	CCAGAGGAG-	--ATCTCTCG	ACGCGGG--A	CTCGGCTTGC	TGAA------	GTGCACTCGG	CAAGAGGCGA	GAG-----CG	GCGGA--CTG	--GTGAGTAC
C.BW.00.00BW17732.AF443095	CCAGAGGAG-	--TTCTCTCG	ACGCAGG--A	CTCGGCTTGC	TGAA------	GTGCACTCGG	CAAGAGGCGA	GAG-----CG	GCGG---CTG	--GTGAGTAC
C.BW.00.00BW17835.AF443096	CCAGAGGAG-	--ATCTCTCG	ACGCAGG--A	CTCGGCTTGC	TGAA------	GTGCACTCGG	CAAGAGGCGA	GAG-----CG	GCGA---CTG	--GTGAGTAC
C.BW.00.00BW17956.AF443097	CCAGAGGAG-	--ATCTCTCG	ACGCAGG--A	CTCGGCTTGC	TGAA------	GTGCACACGG	CAAGAGGCGA	GAG-----CG	GCGG---CTG	--GTGAGTAC
C.BW.00.00BW18113.AF443098	CCAGAGAAG-	--ATCTCTCG	ACGCAGG--G	CTCGGCTTGC	TGAA------	GTGCACTCGG	CAAGAGGCGA	GAG-----GG	GCGA---CTG	--GTGAGTAC
C.BW.00.00BW18595.AF443099	CCAGAGGAG-	--ATCTCTCG	ACGCAGG--A	CTCGGCTTGC	TGAA------	GTGCACTCGG	CAAGAGGCGA	GAG-----CG	GCGA---CTG	--GTGAGTAC
C.BW.00.00BW18802.AF443100	CCAGAGGAG-	--ATCTCTCG	ACGCAGG--A	CTCGGCTTGC	TGAA------	GTGCACTCGG	CAAGAGGCGA	GAG-----CG	GCGG---CTG	--GTGAGTAC
C.BW.00.00BW192113.AF443101	CCAGAGAAG-	--TTCTCTCG	ACGCAGG--A	CTCGGCTTGC	TGAA------	GTGCACTCGG	CAAGAGGCGA	GGGGAG--CG	GCGG---CTG	--GTGAGTAC
C.BW.00.00BW20361.AF443102	CCAGAGGAG-	--AACTCTCG	ACGCAGG--A	CTCGGCTTGC	TGAA------	GTGCACTCGG	CAGGAGGCGA	GAG-----CG	GCGG---CTG	--GTGAGTAC
C.BW.00.00BW20636.AF443103	CCAGAGGAG-	--ATCTCTCG	ACGCAGG--A	CTCGGCTTGC	TGAA------	GTGCACTCGG	CAAGAGGCGA	GAG-----AG	GCGA---CTG	--GTGAGTAC
C.BW.00.00BW20872.AF443104	CCAGAGGAG-	--ATCTCTCG	ACGCAGG--A	CTCGGCTTGC	TGAA------	GTGCACTCGG	CAAGAGGCGA	GAG-----CG	GCGG---CTG	--GTGAGTAC
C.BW.00.00BW2127214.AF443105	CCAGAGGAG-	--ATCTCTCG	ACGCAGG--A	CTCGGCTTGC	TGAA------	GTGCACTCGG	CAAGAGGCGA	GAG-----CG	GCGG---CTG	--GTGAGTAC
C.BW.00.00BW22767.AF443107	CCAGAGGAG-	--ATCTCTCG	ACGCAGG--A	CTCGGCTTGC	TGAA------	GTGCACTCGG	CAAGAGGCGA	AAG-----CG	GCGG---CTG	--GTGAGTAC
C.BW.00.00BW38193.AF443108	CCAGAGGAG-	--ATCTCTCG	ACGCAGG--A	CTCGGCTTGC	TGAA------	GTGCACTCGG	CAAGAGGCGA	GAG-----CG	GCGA---CTG	--GTGAGTAC
C.BW.00.00BW38428.AF443109	CCAGAGAAG-	--ATCTCTCG	ACGCAGG--A	CTCGGCTTGC	TGAA------	GTGCACTCGG	CAAGAGGCGA	GAG-----CG	GCGA---CTG	--GTGAGTAC
C.BW.00.00BW38713.AF443110	CCAGAGGAG-	--ATCTCTCG	ACGCAGG--A	CTCGGCTTGC	TGAA------	GTGCACTCGG	CAAGAGGCGA	GAG-----CG	GCGA---CTG	--GTGAGTAC
C.BW.00.00BW3876_9.AF443111	CCAGAGAAG-	--ATCTCTCG	ACGCAGG--G	CTCGGCTTGC	TGAA------	GTGCACTCGG	CAAGAGGCGA	GA------GG	GCGA---CTG	--GCGAGTAC
C.BW.00.00BW3886_8.AF443112	CCAGAGGAG-	--ATCTCTCG	ACGCAGG--A	CTCGGCTTGC	TGAA------	GTGCACTCGG	CAAGAGGCGA	GAG-----CG	GCGA---CTG	--GTGAGTAC
C.BW.00.00BW3891_6.AF443113	CCAGAGGAG-	--ATCTCTCG	ACGCAGG--A	CTCGGCTTGC	TGAA------	GTGTACTCGG	CAAGAGGCGA	GAG-----CG	GCGA---CTG	--GTGAGTAC
C.BW.00.00BW3970_2.AF443114	CCAGAGAAG-	--ATCTCTCG	ACGCAGG--A	CTCGGCCTGC	TGAA------	GTGCACTCGG	CAGGAGGCGA	GAG-----CG	GCGG---CTG	--GTGAGTAC
C.BW.00.00BW5031_1.AF443115	CCAGAGGAG-	--ATCTCTCG	ACGCAGG--A	CTCGGCTTGC	TGAA------	GTGCACTCGG	CAAGAGGCGA	GAG-----CG	GCGG---CTG	--GTGAGTAC
C.BW.96.96BW01B03.AF110959	CCGGAGGAG-	--ATCTCTCG	ACGCAGG--A	CTCGGCTTGC	TGAA------	GTGCACTCGG	CAAGAGGCGA	AAG-----GG	GCGG---CTG	--GTGAGTAC
C.BW.96.96BW0402.AF110962	CCAGAGGAG-	--ATCTCTCG	ACGCAGG--A	CTCGGCTTGC	TGAA------	GTGCACTCGG	CAAGAGGCGA	GAG-----CG	GCGG---CTG	--GTGAGTAC
C.BW.96.96BW0502.AF110967	CCAGAGAAG-	--TTCTCTCG	ACGCAGG--A	CTCGGCTTGC	TGAA------	GTGCACTCGG	CAAGAGGCGA	GAG-----GG	GCGG---CTG	--GTGAGTAC
C.BW.96.96BW06.AF290028	CCAGAGGAG-	--GGCTCTCG	ACGCAGG--A	CTCGGCTTGC	TGAA------	GTGCACTCGG	CAAGAGGCGA	GGGG----CG	GCGA---CTG	--GTGAGTAC
C.BW.96.96BW1104.AF110969	CCAGAGGAG-	--ATCTCTCG	ACGCAGG--A	CTCGGCTTGC	TGAA------	GTGCACTCGG	CAAGAGGCGA	GAG-----GG	GCGG---CTG	--GTGAGTAC
C.BW.96.96BW1210.AF110972	CCAGAGGAG-	--ATCTCTCG	ACGCAGG--A	CTCGGCTTGC	TGAA------	GTGCACTCGG	CAAGAGGCGA	GGG-----AG	GCGA---CTG	--GTGAGTAC
C.BW.96.96BW15C02.AF110974	CCAGAGGAG-	--ATCTCTCG	ACGCAGG--A	CTCGGCTTGC	TGAA------	TTGCACTCGG	CAAGAGGCGA	AAG-----CG	GCGG---CTG	--GTGAGTAC
C.BW.96.96BW16B01.AF110976	CCAGAGGAG-	--ATCTCTCG	ACGCAGG--A	CTCGGCTTGC	TGAA------	GTGCACTCGG	CAAGAGGCGA	GGGG----CG	GCGG---CTG	--GTGAGTAC
C.BW.96.96BW17.AF110980	CCAGAGGAG-	--ATCTCTCG	ACGCAGG--A	CTCGGCTTGC	TGAA------	GTGCACTCGG	CAGGAGGCGA	GAG-----CG	GCGG---CTG	--GTGAGTAC
C.BW.96.96BWM032.AF443075	CCGGAGCAG-	--ATCTCTCG	ACGCAGG--A	CTCGGCTTGC	TGAA------	GCGCGCACGG	CAAGAGGCGA	GGG-----GG	GCGA---CTG	--GTGAGTAC
C.BW.96.96BWMO1_5.AF443074	CCAGAGGAA-	--ATCTCTCG	ACGCAGG--A	CTCGGCTTGC	TGAA------	GTGCACTCGG	CAAGAGGCGA	GAG-----GG	GCGG---CTG	--GTGAGTAC
C.BW.98.98BWMC122.AF443076	CCAGAGGAG-	--ATCTCTCG	ACGCAGG--A	CTCGGCTTGC	TGAA------	GTGCACTCGG	CAAGGGGCGA	GAG-----GG	GCGG---CTG	--GTGAGTAC
C.BW.98.98BWMC134.AF443077	CCAGAGGAG-	--ATCTCTCG	ACGCAGG--A	CTCGGCTTGC	TGAA------	GTGCACTCGG	CAAGAGGCGA	GAG-----AG	GCGG---CTG	--GTGAGTAC
C.BW.98.98BWMC14A3.AF443078	CCAGAGGAG-	--ATCTCTCG	ACGCAGG--A	CTCGGCTTGC	TGAA-----A	GTGCACTCGG	CAAGAGGCGA	GGGGGC--GG	CGGA---CTG	--GTGAGTAC
C.BW.98.98BWMO1410.AF443079	CCAGAGGAG-	--ATCTCTCG	ACGCAGG--A	CTCGGCTTGC	TGAA------	GTGCACTCGG	CAAGAGGCGA	GAG-----CG	GCGG---CTG	--GTGAGTAC
C.BW.98.98BWMO18D5.AF443080	CCAGAGGAG-	--ATCTCTCG	ACGCAGG--A	CTCGGCTTGC	TGAA-----A	GTGCACTCGG	CAAGAGGCGA	GAGG----CG	GCGG---CTG	--GTGAGTAC
C.BW.98.98BWMO36A5.AF443081	CCAGAGGAG-	--ATCTCTCG	ACGCAGG--A	CTCGGCTTGC	TGGA-----A	GTGCACTCGG	CAAGAGGCGA	GGG-----GG	GCGG---CTG	--GTGAGTAC
C.BW.98.98BWMO37D5.AF443082	CCAGAGGAG-	--ATCTCTCG	ACGCAGG--A	CTCGGCTTGC	TGAA------	GTGCACTCGG	CAAGAGGCGA	GAG-----GG	GCGA---CTG	--GTGAGTAC
C.BW.99.99BW393212.AF443083	CCAGAGAAG-	--ATCTCTCG	ACGCAGG--A	CCCGGCTTGC	TGAA------	GTGCACTCGG	CAAGAGGCGA	GAG-----GG	GCGG---CTG	--GTGAGTAC
C.BW.99.99BW46424.AF443084	CCAGAGGAG-	--ATCTCTCG	ACGCAGG--G	ATCGGCTTGC	TGAA-----A	GTGCACTCGG	CAAGAGGCGA	GAG-----CG	GCGG---CTG	--GTGAGTAC
C.BW.99.99BW4745.AF443085	CCAGAGGAG-	--ATCTCTCG	ACGCAGG--A	CTCGGCTTGC	TGAA------	GTGCACTCGG	CGAGAGGCGA	GAG-----CG	GCGG---CTG	--GTGAGTAC
C.BW.99.99BW47547.AF443086	CCAGAGGAG-	--ATCTCTCG	ACGCAGG--A	CTCGGCTTGC	TGAA-----A	GTGCACTCGG	CAAGAGGCGA	GAG-----CG	GCGG---CCG	--GTGAGTAC
C.BW.99.99BWMC168.AF443087	CCAGAGGAG-	--ATCTCTCG	ACGCAGG--A	CTCGGCTTGC	TGAA------	GTGCACACGG	CAAGAGGCGA	GAG-----CG	GCGG---CTG	--GTGAGTAC
C.DJ.x.DJ259.AB485643	CCAGAGGAG-	--ATCTCTCG	ACGCAGG--G	CTCGGCTTGC	TGAA------	GTGCACTCGG	CAAGAGGCGA	GAG-----GG	GCGG---CTG	--GTGAGTAC
C.DK.01.CTL_015.EF514713	CCAGAGGAG-	--ATCTCTCG	ACGCAGG--A	CTCGGCTTGC	TGAA-----A	GTGCACTCGG	CAAGAGGCGA	GAG-----CG	GCGG---CTG	--GTGAGTAC
C.ES.06.X1936.EU786673	CCAGAGAAG-	--ATCTCTCG	ACGCAGG--A	CTCGGCTTGC	TGAA------	GTGCACTCGG	CAAGAGGCGA	GAG-----GG	GCGG---CTG	--GTGAGTAC

C.ES.07.X2118_2.EU884500	CCAGAGGAG- --ATCTCTCG ACGCAGG--A CTCGGCTTGC TGAA------ GTGCACTCGG CAAGAGGCGA GAG-----CG GCGG---CTG --GTGAGTAC
C.ES.08.X2363_2.EU786681	CCAGAGAAG- --ATCTCTCG ACGCAGG--A CTCGGCTTGC TGAG------ GTGCACACGG CAAGAGGCGA GAG-----GG GCGA---CTG --GTGAGTA-
C.ET.86.ETH2220.U46016	CCAGAGGAG- --ATCTCTCG ACGCAGG--A CTCGGCTTGC TGAA------ GTGCACTCGG CAAGAGGCGA GAG-----CG GCGA---CTG --GTGAGTAC
C.IL.98.98IS002.AF286233	CCAGAGGAG- --ATCTCTCG ACGCAGG--A CTCGGCTTGC TAAA------ GTGCACTTGG CAAGAGGCGA GAG-----CG GCGA---CTG --GTGAGTAC
C.IN.03.D24.EF469243	CCAGAGAAG- --ATCTCTCG ACGCAGG--A CTCGGCTTGC TGAA------ GTGCACTCGG CAAGAGGCGA GAG-----GG GCGG---CTG --GTGAGTAC
C.IN.93.93IN101.AB023804	CCAGAGAAG- -ATCCTCTAG ACGCAGG--A CTCGGCTTGC TGAA------ GTGCACTCGG CAAGAGGCGA GAG-----CG GCGA---CTG --GTGAGTAC
C.IN.93.93IN904.AF067157	CCAGAGGAG- --ATCTCTCG ACGCAGG--A CTCGGCTTGC TGAA------ GTGCACTCGG CAAGAGGCGA GAG-----TG GCGA---CTG --GTGAGTAC
C.IN.93.93IN999.AF067154	CCAGAGGAG- --ATCTCTCG ACGCAGG--A CTCGGCTTGC TGAA------ GTGCACTCGG CAAGAGGCGA GAG-----CG GCGA---CTG --GTGAGTAC
C.IN.94.94IN11246.AF067159	CCAGAGGAG- --ATCTCTCG ACGCAGG--A CTCGGCTTGC TGAA------ GTGCACTCGG CAAGAGGCGA GAG-----CG GCGA---CTG --GTGAGTAC
C.IN.94.94IN476.AF286223	CCAGAGGAG- --ATCTCTCG ACGCAGG--A CTCGGCTTGC TGAA------ GTGCACTCAG CAAGAGGCGA GGG-----GG GCGA---CTG --GTGAGTAC
C.IN.95.95IN21068.AF067155	CCAGAGAAG- --ATCTCTCG ACGCAGG--A CTCGGCTTGC TGAA------ GTGCACTCGG CAAGAGGCGA GAG-----CG GCGA---CTG --GTGAGTAC
C.IN.98.98IN012.AF286231	CCAGAGGAG- --ATCTCTCG ACGCAGG--A CTCGGCTTGC TGAA------ GTGCACTCGG CAAGAGGCGA GGGG----TG GCGG---CTG --GTGAGTAC
C.IN.98.98IN022.AF286232	CCAGAGGAG- --AACTCTCG ACGCAGG--A CTCGGCTTGC TGAA------ GTGCACTCGG CAAGAGGCGA GGGG----CG GCGA---CTG --GTGAGTAC
C.IN.99.01IN565_10.AY049708	CCAGAGAAG- --ATCTCTCG ACGCAGG--A CTCGGCTTGC TGAA------ GTGCACTCGG CAAGAGGCGA GAG-----GG GCGA---CTG --GTGAGTAC
C.IN.x.VB39.EF694033	CCAGAGGAG- --ATCTCTCG ACGCAGG--A CTCGGCTTGC TGAA------ GTGCACTCAG CAAGAGGCGA GGG-----CG GCGA---CTG --GTGAGTAC
C.IN.x.VB49.EF694036	CCAGAGGAG- --ATCTCTCG ACGCAGG--A CTCGGCTTGC TGAA------ GTGCACTCGG CAAGAGGCGA GAG-----AG GCGA---CTG --GTGAGTAC
C.MM.99.mIDU101_3.AB097871	CCAGAGGAG- --ATCTCTCG ACGCAGG--A CTCGGCTTGC TGAA------ GTGCACTCGG CAAGAGGCGA GAG-----GG GCGA---CTG --GTGAGTAC
C.TZ.98.98TZ013.AF286234	CCAGAGGAG- --ATCTCTCG ACGCAGG--A CTCGGCTTGC TGAA------ GTGCACTCGG CAAGAGGCGA GGG-----GG GCGA---CTG --GTGAGTAC
C.TZ.98.98TZ017.AF286235	CCAGAGCAG- --ATCTCTCG ACGCAGG--A CTCGGCTTGC TGAA------ GTGCACTCGG CAAGAGGCGA GAG-----CG GCGG---CTG --GTGAGTAC
C.ZA.00.1069MB.AY838567	CCAGAGGAG- --ATCTCTCG ACGCAGG--A CTCGGCTTGC TGAA------ GTGCACTCGG CAAGAGGCGA GAG-----CG GCGG---CTG --GTGAGTAC
C.ZA.00.1119MB.AY463229	CCAGAGAAG- --ATCTCTCG ACGCAGG--A CTCGGCTTGC TGAA------ GTGCACTCGG CAAGAGGCGA GAG-----GG GCGG---CTG --GTGAGTAC
C.ZA.00.1134MB.AY463217	CCAGAGGAG- --ATCTCTCG ACGCAAG--A CTCGGCTTGC TGAA------ GTGCACTCGG CAAGAGGCGA GAG-----CG GCGA---CTG --GTGAGTAC
C.ZA.00.1157M3M.AY585266	CCAGAGGAG- --GTCTCTCG ACGCAGG--A CTCGGCTTGC TGAA------ GTGCACTCGG CAAGAGGCGA GAG-----CG GCGA---CTG --GTGAGTAC
C.ZA.00.1162MB.AY463224	CCAGAGGAG- --GTCTCTCG ACGCAGG--A CTCGGCTTGC TGAA------ GTGCACTCGG CAAGAGGCGA GAG-----CG GCGA---CTG --GTGAGTAC
C.ZA.00.1165MB.AY463230	CCAGAGGAG- --ATCTCTCG ACGCAGG--A CTCGGCTTGC TGAA------ GTGCACTCGG CAAGAGGCGA GAG-----CG GCGG---CTG --GTGAGTAC
C.ZA.00.1168MB.AY463231	CCAGAGGAG- --ATCTCTCG ACGCAGG--A CTCGGCTTGC TGAA------ GTGCACTCGG CAAGAGGCGA GAG-----CG GCGG---CTG --GTGAGTAC
C.ZA.00.1170MB.AY463225	CCAGAGAAG- --AACTCTCG ACGCAGG--A CTCGGCTTGC TGAA------ GTGCACTCGG CAAGAGGCGA GAG-----CG GCGG---CTG --GTGAGTAC
C.ZA.00.1171MB.AY463232	CCAGAGGAG- --ATCTCTCG ACGCAGG--A CTCGGCTTGC TGAA------ GTGCACTCGG CGAGAGGCGA GAG-----CG GCGG---CTG --GTGAGTAC
C.ZA.00.1176MB.AY463218	CCAGAGGAG- --ATCTCTCG ACGCAAG--A CTCGGCTTGC TGAA------ GTGCACTCGG CAAGAGGCGA GAG-----CG GCGA---CTG --GTGAGTAC
C.ZA.00.1178MB.AY463233	CCAGAGGAG- --ATCTCTCG ACGCAGG--A CTCGGCTTGC TGAA------ GTGCACTCGG CAAGAGGCGA GGGG----AG GCG------- --GTGAGTAC
C.ZA.00.1184MB.AY838566	CCAGAGGAG- --ATCTCTCG ACGCAGG--A CTCGGCTTGC TGAA------ GTGCACTCGG CAAGAGGCGA GAG-----CG GCGG---CTG --GTGAGTAC
C.ZA.00.1189MB.AY838565	CCAGAGAAG- --ATCTCTCG ACGCAGG--A CTCGGCTTGC TGAA------ GTGCACTCGG CAAGAGGCGA GGG-----CG GCGA---CTG --GTGAGTAC
C.ZA.00.1192M3M.AY463219	CCAGAGGAG- --ATCTCTCG ACGCAGG--A CTCGGCTTGC TGAA------ GTGCACTCGG CAAGAGGCGA GAG-----CG GCGA---CTG --GTGAGTAC
C.ZA.00.1195MB.AY463220	CCAGAGGAG- --ATCTCTCG ACGCAGG--G CTCGGCTTGC TGAA------ GTGCACTCGG CAAGAGGCGA GAG-----CG GCGA---CTG --GTGAGTAC
C.ZA.00.1197MB.AY463234	CCAGAGGAG- --ATCTCTCG ACGCAGG--A CTCGGCTTGC TGAA-----C GTGCACTCGG CAAGAGGCGA GAG-----CG GCGA---CTG --GTGAGTAC
C.ZA.00.1210MB.AY463221	CCAGAGGAG- --ATCTCTCG ACGCAGG--A CTCGGCTTGC TGAA------ GTGCACTCGG CAAGAGGCGA GAG-----CG GCGA---CTG --GTGAGTAC
C.ZA.00.1214MB.AY463236	CCAGAGGAA- --ATCTCTCG ACGCAGG--A CTCGGCTTGC TGAA------ GTGCACTCGG CAAGAGGCGA GAG-----CG GCGG---CTG --GTGAGTAC
C.ZA.00.1217MB.AY463226	CCAGAGAAG- --ATCTCTCG ACGCAGG--A CTCGGCTTGC TGAA------ GTGCACTCGG CAAGAGGCGA GAG-----CG GCGA---CTG --GTGAGTAC
C.ZA.00.1225MB.AY463227	CCAGAGGAG- --ATCTCCCG ACGCAGG--A CTCGGCTTGC TGAA------ GTGCACTCGG CAAGAGGCGA GAG-----CG GCGA---CTG --GTGAGTAC
C.ZA.00.1228MB.AY463222	CCAGAGGAG- --ATCTCTCG ACGCAAG--A CTCGGCTTGC TGAA------ GCGCACTCGG CAAGAGGCGA GAG-----CG GCGA---CTG --GTGAGTAC
C.ZA.00.J112MA.AY838568	CCAGAGAAG- --ATCTCTCG ACGCAGG--A CTCGGCTTGC TGAA------ GTGCACTCGG CAAGAGGCGA GGGG----CG GCGG---CTG --GTGAGTAC
C.ZA.00.J38MA.AY463228	CCAGAGGAG- --ATCTCTCG ACGCAGG--A CTCGGCTTGC TGAA------ GTGCACTCGG CAAGAGGCGA GAG-----CG GCGA---CTG --GTGAGTAC
C.ZA.01.01ZATM45.AY228557	CCAGAGAAG- --ATCTCTCG ACGCAGG--A CTCGGCTTGC TTAA------ GTGCACTCGG CAAGAGGCGA GAG-----GG GCGG---CTG --GTGAGTAC
C.ZA.01.2134MB.AY463237	CCAGAGGAG- --ATCTCTCG ACGCAGG--A CTCGGCTTGC TGAA------ GTGCACTCGG CAAGAGGCGA GAG-----GG GCGG---CTG --GTGAGTAC
C.ZA.01.J54Ma.AY463223	CCAGAGGAG- --ATCTCTCG ACGCGAG--A CTCGGCTTGC TGAA------ GTGCACTCGG CAAGAGGCGA GAG-----CG GCGG---CTG --GTGAGTAC
C.ZA.02.02ZAPS001MB1.DQ275648	CCAGAGGAG- --ATCTCTCG ACGCAGG--A CTCGGCTTGC TGAA------ GTGCACTCGG CAGGAGGCGA GAG-----CG GCGG---CTG --GTGAGTAC
C.ZA.02.02ZAPS005MB1.DQ351235	CCAGAGGAG- --ATCTCTCG ACGCAGG--A CTCGGCTTGC TGAA------ GTGCACACGG CAAGAGGCGA GAG-----CG GCGA---CTG --GTGAGTAC
C.ZA.02.02ZAPS006MB1.DQ351220	CCAGAGGAG- --ATCTCTCG ACGCAGG--A CTCGGCTTGC TGAA------ GTGCACTCGG CAAGAGGCGA GAG-----CG GCGA---CTG --GTGAGTAC
C.ZA.02.02ZAPS008MB1.DQ275647	CCAGAGGAG- --ATCTCTCG ACGCAGG--G CTCGGCTTGC TGAA-----A GTGCACTCGG CAAGAGGCGA GAG-----CG GCGG---CTG --GTGAGTAC
C.ZA.02.02ZAPS013MB1.DQ351222	CCAGAGGAG- --ATCTCTCG ACGCAAG--A CTCGGCTTGC TGAA------ GTGCACTCGG CAAGAGGCGA GAG-----CG GCGA---CTG --GTGAGTAC
C.ZA.02.02ZAPS014MB1.DQ351218	CCAGAGAAG- --ATCTCTCG ACGCAGG--A CTCGGCTTGC TGAA------ GTGCACTCGG CAAGAGGCGA GAG-----CG GCGG---CTG --GTGAGTAC
C.ZA.02.02ZAPS015MB1.DQ369995	CCAGAGAAG- --ATCTCTCG ACGCAGG--G CTCGGCTTGC TGAA------ GTGCACTCGG CAAGAGGCGA GAG-----GG GCGA---CTG --GTGAGTAC
C.ZA.03.03ZAPS017MB1.DQ351224	CCAGAGGAG- --ATCTCTCG ACGCAGG--A CTCGGCTTGC TGAA------ GTGCACTCGG CAAGAGGCGA GAG-----CG GCGA---CTG --GTGAGTAC
C.ZA.03.03ZAPS020MB1.DQ275653	CCAGAGGAG- --ATCTCTCG ACGCAGG--G CTCGGCTTGC TGAA------ GTGCACTCGG CAAGAGGCGA GAG-----CG GCGA---CTG --GTGAGTAC
C.ZA.03.03ZAPS021MB1.DQ369978	CCAGAGGAG- --ATCTCTCG ACGCAGG--A CTCGGCTTGC TGAA------ GTGCACTCGG CAAGAGGCGA GAG-----CG GCGG---CCG --GTGAGTAC
C.ZA.03.03ZAPS023MB1.DQ351225	CCAGAGGAG- --ATCTCTCG ACGCAGG--A CTCGGCTTGC TGAA------ GTGCACTCGG CAAGAGGCGA GAG-----CG GCGA---CTG --GCGAGTAC
C.ZA.03.03ZAPS024MB1.DQ396367	CCAGAGGAG- --ATCTCTCG ACGCAAG--A CTCGGCTTGC TGAA------ GTGCACTCGG CAAGAGGCGA GAG-----CG GCGA---CTG --GTGAGTAC
C.ZA.03.03ZAPS025MB1.DQ351226	CCAGAGAAG- --ATCTCTCG ACGCAGG--A CTCGGCTTGC TGAA------ GTGCACTCGG CAAGAGGCGA GAG-----GG GCGG---CTG --GTGAGTAC
C.ZA.03.03ZAPS026MB1.DQ369985	CCAGAGGAG- --ATCTCTCG ACGCAGG--A CTCGGCTTGC TGAA------ GTGCACTCGG CAAGAGGCGA GAG-----GG GCGG---CTG --GTGAGTAC
C.ZA.03.03ZAPS027MB1.DQ351223	CCAGAGGAG- --ACCTCTCG ATGCAAG--A CTCGGCTTGC TGAA------ GTGCACTCGG CAAGAGGCGA GAG-----CG GCGA---CTG --GTGAGTAC
C.ZA.03.03ZAPS030MB1.DQ369996	CCAGAGGAG- --ATCTCTCG ACGCAGG--A CTCGGCTTGC TGAA------ GTGCACTCGG CAAGAGGCGA GAG-----CG GCGG---CTG --GTGAGTAC
C.ZA.03.03ZAPS032MB1.DQ445633	CCAGAGGAG- --AACTCTCG ACGCAGG--A CTCGGCTTGC TGAA------ GTGCACTCGG CAAGAGGCGA GAG-----CG GCGA---CTG --GTGAGTAC
C.ZA.03.03ZAPS034MB1.DQ369979	CCAGAGGAG- --ATCTCTCG ACGCAGG--A CTCGGCTTGC TGAA------ GTGCACTCGG CAAGAGGCGA GAG-----CG GCGG---CTG --GTGAGTAC
C.ZA.03.03ZAPS042MB1.DQ369977	CCAGAGGAG- --ATCTCTCG ACGCAGG--A CTCGGCTTGC TGAA------ GTGCACTCGG CAAGAGGCGA GAG-----CG GCGA---CTG --GTGAGTAC
C.ZA.03.03ZAPS043MB1.DQ351227	CCAGAGGAG- --ATCTCTCG ACGCAGG--A CTCGGCTTGC TGAA------ GTGCACTCGG CAAGAGGCGA GAG-----AG GCGG---CTG --GTGAGTAC
C.ZA.03.03ZAPS044MB1.DQ396384	CCAGAGGAG- --ATCTCTCG ACGCAGG--A CTCGGCTTGC TGAA------ GTGCACTCGG CAGGAGGCGA GAG-----CG GCGA---CTG --GTGAGTAC
C.ZA.03.03ZAPS046MB1.DQ369984	CCAGAGAAG- --ATCTCTCG ACGCAGG--A CTCGGCTTGC TGAA------ GTGCACTCGG CAAGAGGCGA GAG-----CG GCGA---CTG --GTGAGTAC
C.ZA.03.03ZAPS048MB1.DQ396364	CCAGAGAAG- --ATCTCTCG ACGCAGG--A CTCGGCTTGC TGAA------ GTGCACTCGG CAAGAGGCGA GAG-----CG GCGA---CTG --GTGAGTAC
C.ZA.03.03ZAPS049MB1.DQ369986	CCAGAGGAG- --ATCTCTCG GCGCAGG--A CTCGGCTTGC TGAA------ GTGCACTCGG CAAGAGGCGA GGGGG---CG GCGA---CTG --GTGAGTAC
C.ZA.03.03ZAPS050MB1.DQ369980	CCAGAGAAG- --AACTCTCG ACGCAGG--A CTCGGCTTGC TGAA------ GTGCACTCGG CAAGAGGCGA GAG-----CG GCGG---CTG --GTGAGTAC
C.ZA.03.03ZAPS051MB1.DQ396385	CCAGAGAAG- --AGCTCTCG ACGCAGG--A CTCGGCTTGC TGAA------ GTGCACTCGG CAGGAGGCGA GGGG----CG GCGG---CCG --GTGAGTAC
C.ZA.03.03ZAPS052MB1.DQ369987	CCAGAGGAG- --ATCTCTCG ACGCAGG--A CTCGGCTTGC TGAA------ GTGCACTCGG CAAGAGGCGA GAG-----CG GCGA---CTG --GTGAGTAC
C.ZA.03.03ZAPS054MB2.DQ369988	CCAGAGAAG- --ATCTCTTG ACGCAGG--G CTCGGCTTGC TGAA------ GTGCACTCGG CAAGAGGCGA GAG-----GG GCGA---CTG --GTGAGTAC
C.ZA.03.03ZAPS055MB1.DQ396373	CCAGAGGAG- --ATCTCTCG ACGCAGG--A CTCGGCTTGC TGAA------ GTGCACTCGG CAAGAGGCGA GAG-----CG GCGA---CTG --GTGAGTAC
C.ZA.03.03ZAPS056MB1.DQ396374	CCAGAGGAG- --ATCTCTCG ACGCAGG--A CTCGGCTTGC TGAA------ GTGCACTCGG CAAGAGGCGA GAG-----CG GCGG---CTG --GTGAGTAC
C.ZA.03.03ZAPS057MB2.DQ369989	CCAGAGAAG- --ATCTCTCG ACGCAGG--G CTCGGCTTGC TGAA------ GTGCACTCGG CAAGAGGCGA GAG-----GG GCGA---CTG --GTGAGTAC
C.ZA.03.03ZAPS059MB2.DQ445634	CCAGAGAAG- --ATCTCTCG ACGCAGG--A CTCGGCTTGC TGAA------ GTGCACTCGG CAAGAGGCGA GAG-----CG GCGA---CTG --GTGAGTAC
C.ZA.03.03ZAPS063MB1.DQ396388	CCAGAGGAG- --ATCTCTCG ACGCAGG--A CTCGGCTTGC TGAA------ GTGCACTCGG CAAGAGGCGA GAG-----CG GCGG---CTG --GTGAGTAC
C.ZA.03.03ZAPS066MB2.DQ396375	CCAGAGGAG- --ATCTCTCG ACGCAGG--A CTCGGCTTGC TGAA------ GTGCACTCGG CAAGAGGCGA GAG-----CG GCGG---CTG --GTGAGTAC
C.ZA.03.03ZAPS067MB2.DQ396389	CCAGAGTAG- --AACTCTCG ACGCAGG--A CTCGGCTTGC TGAA------ GTGCACTCGG CAAGAGGCGA GGG-----GG GCGA---CTG --GTGAGTAC
C.ZA.03.03ZAPS071MB1.DQ396376	CCAGAGGAG- --ATCTCTCG ACGCAGG--A CTCGGCTTGC TGAA------ GTGCACTCGG CAAGAGGCGA GAG-----CG GCGG---CTG --GTGAGTAC
C.ZA.03.03ZAPS073MB1.DQ275649	CCAGAGGAG- --ATCTCTCG ACGCAGG--A CTCGGCTTGC TGAA------ GTGCACTCGG CAAGAGGCGA GAG-----CG GCGA---CTG --GTGAGTAC
C.ZA.03.03ZAPS074MB2.DQ351228	CCAGAGAAG- --ATCTCTCG ACGCAGG--A CTCGGCTTGC TGAA------ GTGCACTCGG CAAGAGGCGA GAG-----CG GCGA---CTG --GTGAGTAC
C.ZA.03.03ZAPS077B1.DQ093591	CCAGAGGAG- --ATCTCTCG ACGCAGG--A CTCGGCTTGC TGAA------ GTGCACTCGG CAAGAGGCGA GAG-----CG GCGA---CTG --GTGAGTAC
C.ZA.03.03ZAPS079B1.DQ093592	CCAGAGAAG- --ATCTCTCG ACGCAGG--A CTCGGCTTGC TGAA------ GTGCACTCGG CAAGAGGCGA GGG-----GG GCGA---CTG --GTGAGTAC
C.ZA.03.03ZAPS081MB1.DQ351219	CCAGAGGAG- --ATCTCTCG ACGCAGG--G CTCGGCTTGC TGAA------ GTGCACTCGG CAAGAGGCGA GAG-----CG GCGG---CTG --GTGAGTAC
C.ZA.03.03ZAPS083MB1.DQ351229	CCAGAGGAG- --ATCTCTCG ACGCAGG--A CTCGGCTTGC TGAA------ GTGCACTCGG CAAGAGGCGA GAG-----CG GCGA---CTG --GTGAGTAC
C.ZA.03.03ZAPS086MB1.DQ275654	CCAGAGGAG- --ATCTCTCG ACGCAGG--A CTCGGCTTGC TGAA------ GTACACTCGG CAAGAGGCGA GAG-----CG GCGG---CTG --GTGAGTAC
C.ZA.03.03ZAPS088MB1.DQ275651	CCAGAGGAG- --ATCTCTCG ACGCAGG--A CTCGGCTTGC TGAA------ GTGCACTCGG CAAGAGGCGA GAG-----CG GCGG---CTG --GTGAGTAC
C.ZA.03.03ZAPS089MB1.DQ351216	CCAGAGGAG- --TTCTCTCG ACGCAGG--A CTCGGCTTGC TGAA------ GTGCACTCGG CAAGAGGCGA GAG-----CG GCGG---CTG --GTGAGTAC
C.ZA.03.03ZAPS091MB1.DQ275645	CCAGAGGAG- --ATCTCTCG ACGCAAG--A CTCGGCTTGC TGAA------ GTGCACTCGG CAAGAGGCGA GAG-----CG GCGG---CTG --GTGAGTAC
C.ZA.03.03ZAPS094MB1.DQ396377	CCAGAGAAG- --ATCTCTCG ACGCAGG--A CTCGGCTTGC TGAA------ GTGCACTCGG CAAGAGGCGA GAG-----CG GCGG---CTG --GTGAGTAC
C.ZA.03.03ZAPS095MB1.DQ275652	CCAGAGGAG- --ATCTCTCG ACGCAGG--A CTCGGCTTGC TGAA------ GTGCACTTGG CAAGAGGCGA GAG-----GG GCGG---CTG --GTGAGTAC
C.ZA.03.03ZAPS097MB1.DQ351230	CCAGAGGAG- --ATCTCTCG ACGCAGG--A CTCGGCTTGC TGAA------ GTGCACTCGG CAAGAGGCGA GAGG----CG GCGA---CTG --GTGAGTAC
C.ZA.03.03ZAPS099MB1.DQ275655	CCAGAGGAG- --ATCTCTCG ACGCAGG--A CTCGGCTTGC TGAA------ GTGCACTCGG CAAGAGGCGA GAG-----CG GCGG---CTG --GTGAGTAC
C.ZA.03.03ZAPS103MB2.DQ275656	CCAGAGAAG- --ACCTCTCG ACGCAGG--A CTCGGCTTGC TGAA------ GTGCACTCGG CAAGAGGCGA GGG-----GG GCGG---CTG --GTGAGTAC
C.ZA.03.03ZAPS104MB1.DQ369990	CCGGAGGAG- --ATCTCTCG ACGCAGG--A CTCGGCTTGC TGAA------ GTGCACTCGG CAAGAGGCGA GAG-----CG GCGA---CTG --GTGAGTAC
C.ZA.03.03ZAPS105MB2.DQ445632	CCAGAGAAG- --ATCTCTCG ACGCAGG--A CTCGGCTTGC TGAA------ GTGCACTCGG CAAGAGGCGA GAG-----CG GCGG---CTG --GTGAGTAC
C.ZA.03.03ZAPS108MB1.DQ396378	CCAGAGGAG- --ATCTCTCG ACGCAGG--A CTCGGCTTGC TGAA------ GTGCACTCGG CAAGAGGCGA GAG-----CG GCGA---CTG --GTGAGTAC
C.ZA.03.03ZAPS112MB2.DQ396386	CCAGAGGAG- --ATCTCTCG ACGCAGG--A CTCGGCTTGC TGAA------ GTGCACTTGG CAAGAGGCGA GGGG----CG GCGA---CTG --GTGAGTAC
C.ZA.03.03ZAPS113MB2.DQ396365	CCAGAGGAG- --ATCTCTCG ACGCAGG--A CTCGGCTTGC TGAA------ GTGCACTCGG CAAGAGGCGA GAG-----CG GCGG---CTG --GTGAGTAC
C.ZA.03.03ZAPS116MB1.DQ445635	CCAGAGGAG- --ATCTCTCG ACGCAGG--A CTCGGCTTGC TGAA------ GTGCACTCGG CAAGAGGCGA GAG-----CG GCGG---CTG --GTGAGTAC
C.ZA.03.03ZAPS118MB1.DQ396368	CCAGGGGAG- --ATCTCTCG ACGCAGG--A CTCGGCTTGC TGAA------ GTGCACTCGG CAAGAGGCGA GAG-----CG GCGA---CTG --GTGAGTAC
C.ZA.03.03ZAPS122MB1.DQ396370	CCAGAGGAG- --ATCTCTCG ACGCAAG--A CTCGGCTTGC TGAA------ GTGCACTCGG CAAGAGGCGA GAG-----CG GCGA---CTG --GTGAGTAC
C.ZA.03.03ZAPS123MB1.DQ396369	CCAGAGGAG- --ATCTCTCG ACGCAAG--A CTCGGCTTGC TGAA------ GTGCACTCGG CAAGAGGCGA GAG-----CG GCGA---CTG --GTGAGTAC

```
C.ZA.03.03ZAPS124MB1.DQ369976    CCAGAGGAG- --ATCTCTCG ACGCAGG--A CTCGGCTTGC TGAA------ GTGCACTCGG CAAGAGGCGA GAG-----CG GCGA---CTG --GTGAGTAC
C.ZA.03.03ZAPS125MB1.DQ396390    CCAGAGAAG- --ATCTCTCG ACGCAGG--A CTCGGCTTGC TGAA------ GTGCACTCGG CAAGAGGCGA GAG-----GG GCGG---CTG --GTGAGTAC
C.ZA.03.03ZAPS126MB1.DQ275657    CCAGAGAAG- --ATCTCTCG ACGCAGG--A CTCGGCTTGC TGAA------ GTGCACTCGG CAAGAGGCGA GAG-----CG GCGG---CTG --GTGAGTAC
C.ZA.03.03ZAPS128MB1.DQ275643    CCAGAGGAG- --ATCTCTCG ACGCAGG--A CTCGGCTTGC TGAA------ GTGCACTCGG CAAGAGGCGA GAG-----CG GCGG---CTG --GTGAGTAC
C.ZA.03.03ZAPS130MB1.DQ275658    CCAGAGAAG- --ATCTCTCG ACGCAAG--A CTCGGCTTGC TGAA------ GTGCACTCGG CAAGAGGCGA GAG-----CG GCGG---CTG --GTGAGTAC
C.ZA.03.03ZAPS131MB1.DQ396380    CCAGAGGAG- --ATCTCTCG ACGCAGG--A CTCGGCTTGC TGAA------ GTGCACTCGG CAAGAGGCGA GAG-----AG GCGA---CTG --GTGAGTAC
C.ZA.03.03ZAPS133MB1.DQ275646    CCAGAGAAG- --ATCTCTCG ACGCAAG--A CTCGGCTTGC TGAA------ GTGCACTCGG CAAGAGGCGA GAG-----CG GCGA---CTG --GTGAGTAC
C.ZA.03.03ZAPS136MB1.DQ351231    CCAGAGGAG- --ATCTCTCG ACGCAGG--A CTCGGCTTGC TGAA------ GTGCACTCGG CAAGAGGCGA GAG-----CG GCGG---CTG --GTGAGTAC
C.ZA.03.03ZAPS140MB1.DQ369981    CCAGAGAAG- --ATCTCTCG ACGCAGG--A CTCGGCTTGC TGAA------ GTGCACTCGG CAAGAGGCGA GAG-----CG GCGA---CTG --GTGAGTAC
C.ZA.03.03ZAPS143MB1.DQ396391    CCAGAGGAG- --ATCTCTCG ACGCAGG--A CTCGGCTTGC TGAA-----A GTGCACTCGG CAAGAGGCGA GAG-----CG GCGA---CTG --GTGAGTAC
C.ZA.03.03ZAPS151MB1.DQ396392    CCAGAGGAG- --ATCTCTCG ACGCAGG--A CTCGGCTTGC TGAA-----G GTGCACTCGG CAAGAGGCGA GAG-----CG GCGA---CTG --GTGAGTAC
C.ZA.03.03ZAPS152MB1.DQ396399    CCAGAGGAG- --ATCTCTCG ACGCAGG--A CTCGGCTTGC TGAA------ GTGCACTCGG CAAGAGGCGA GAG-----CG GCGG---CTG --GTGAGTAC
C.ZA.03.03ZAPS155MB1.DQ396371    CCAGAGGAG- --ATCTCTCG ACGCAGG--G CTCGGCTTGC TGAA------ GTGCACTCGG CAAGAGGCGA GAG-----CG GCGG---CTA --GTGAGTAC
C.ZA.03.03ZASK005B2.DQ011175     CCAGAGAAG- --AACTCTCG ACGCAGG--A CTCGGCTTGC TGAA------ GTGCACTCGG CAAGAGGCGA GGGG----CG GCGA---CTG --GTGAGTAC
C.ZA.03.03ZASK006B2.AY878056     CCAGAGAAG- --ATCTCTCG ACGCAGG--A CTCGGCTTGC TGAA------ GTGCACTCGG CAAGAGGCGA GAG-----CG GCGG---CTG --GTGAGTAC
C.ZA.03.03ZASK010B2.DQ164104     CCAGAGAAG- --ATCTCTCG ACGCAGG--G CTCGGCTTGC TGAA------ GTGCACTCGG CAAGAGGCGA GAG-----GG GCGG---CTG --GTGAGTAC
C.ZA.03.03ZASK011B2.AY901965     CCAGAGGAG- --ATCTCTCG ACGCAGG--A CTCGGCTTGC TGAA------ GTGCACTCGG CAAGAGGCGA GAG-----CG GCGA---CTG --GTGAGTAC
C.ZA.03.03ZASK013B2.DQ275660     CCAGAGAAG- --ATCTCTCG ACGCAGG--A CTCGGCTTGC TGAA------ GTGCACTCGG CAAGAGGCGA GAG-----CG GCGA---CTG --GTGAGTAC
C.ZA.03.03ZASK016MB2.DQ351233    CCAGAGAAG- --ATCTCTCG ACGCAGG--A CTCGGCTTGC TGAA------ GTGCACTCGG CAAGAGGCGA GAA-----CG GCGG---CTG --GTGAGTAC
C.ZA.03.03ZASK019B2.AY878063     CCAGAGAAG- --ATCTCTCG ACGCAGG--A CTCGGCTTGC TGAA------ GTGCACGCGG CAAGAGGCGA GGG-----GG GCGG---CTG --GTGAGTAC
C.ZA.03.03ZASK020B2.AY878064     CCAGAGGAG- --ATCTCTCG ACGCAGG--A CTCGGCTTGC TGAA------ GTGCACTCGG CAAGAGGCGA GAG-----CG GCGG---CTG --GTGAGTAC
C.ZA.03.03ZASK026B2.DQ011165     CCAGAGGAG- --ATCTCTCG ACGCAGG--A CTCGGCTTGC TGAA------ GTGCACTCGG CAAGAGGCGA GAG-----CG GCGG---CTG --GTGAGTAC
C.ZA.03.03ZASK034B1.AY878065     CCAGAGAAG- --AACTCTCG ACGCAGG--A CTCGGCTTGC TGAA------ GTGCACTCGG CAAGAGGCGA GAG-----CG GCGG---CTG --GTGAGTAC
C.ZA.03.03ZASK036B1.AY901966     CCAGAGGAG- --ATCTCTCG ACGCAGG--A CTCGGCTTGC TGAA------ GTGCACTCGG CAAGAGGCGA GAG-----CG GCGG---CTG --GTGAGTAC
C.ZA.03.03ZASK039B2.AY878068     CCAGAGAAG- --ATCTCCCG ACGCAGG--A CTCGGCTTGC TGAA------ GTGCACTCGG CAAGAGGCGA GAG-----CG GCGG---CTG --GTGAGTAC
C.ZA.03.03ZASK058B2.AY901967     CCAGAGAAG- --ATCTCTCG ACGCAGG--A CTCGGCTTGC TGAA------ GTGCACTCGG CAGGAGGCGA GGG-----GG GCGA---CTG --GTGAGTAC
C.ZA.03.03ZASK061B1.AY901968     CCAGAGGAG- --ATCTCTCG ACGCAGG--A CTCGGCTTGC TGAA------ GTGCACTCGG CAAGAGGCGA GAG-----CG GCGG---CTG --GTGAGTAC
C.ZA.03.03ZASK062B1.DQ164113     CCAGAGGAG- --ATCTCTCG ACGCAGG--A CTCGGCTTGC TGAA------ GTGCACTCGG CAAGAGGCGA GAG-----CG GCGG---CTG --GTGAGTAC
C.ZA.03.03ZASK066B1.AY901969     CCAGAGAAG- --ATCTCTCG ACGCAGG--G CTCGGCTTGC TGAA------ GTGCACTCGG CAAGAGGCGA GAGG----CG GCGG---CTG --GTGAGTAC
C.ZA.03.03ZASK067B1.DQ275642     CCAGAGGAA- --ATCTCTCG ACGCAGG--A CTCGGCTTGC TGAA-----A GTGCACTCGG CAAGAGGCGA GAG-----CG GCGG---CTG --GTGAGTAC
C.ZA.03.03ZASK072B1.DQ093593     CCAGAGGAG- --ATCTCTCG ACGCAGG--A CTCGGCTTGC TGAA------ GTGCACTCGG CAAGAGGCGA GAG-----GG GCGA---CTG --GTGAGTAC
C.ZA.03.03ZASK073B1.AY901970     CCAGAGAAG- --ATCTCTCG ACGCAGG--A CTCGGCTTGC TGAA-----A GTGCACTCGG CAAGAGGCGA GAG-----CG GCGG---CTG --GTGAGTAC
C.ZA.03.03ZASK076B1.AY901975     CCAGAGGAG- --ATCTCTCG ACGCAGG--A CTCGGCTTGC TGAA------ GTGCACTCGG CAAGAGGCGA GAG-----CG GCGA---CTG --GTGAGTAC
C.ZA.03.03ZASK078B1.AY901971     CCAGAGGAG- --ATCTCTCG ACGCAGG--A CTCGGCTTGC TGAA------ GTGCACTCGG CAAGAGGCGA GAG-----CG GCGA---CTG --GTGAGTAC
C.ZA.03.03ZASK084B1.AY901981     CCAGAGAAG- --ATCTCTCG ACGCAGG--A CTCGGCTTGC TGAA-----A GTGCACTCGG CAAGAGGCGA GAG-----CG GCGA---CTG --GTGAGTAC
C.ZA.03.03ZASK092B1.AY878057     CCAGAGGAG- --ATCTCTCG ACGCAGG--A CTCGGCTTGC TGAA------ GTGCACACGG CAAGAGGCGA GAG-----CG GCGA---CTG --GTGAGTAC
C.ZA.03.03ZASK094B1.AY878070     CCAGAGGAG- --ATCTCTCG ACGCAGG--A CTCGGCTTGC TGAA------ GTGCACTCGG CAAGAGGCGA GAG-----CG GCGA---CTG --GTGAGTAC
C.ZA.03.03ZASK097B1.AY878060     CCAGAGAAG- --ATCTCTCG ACGCAGG--A CTCGGCTTGC TGAA------ GTGCACTCGG CAAGAGGCGA GAG-----CG GCGG---CTG --GTGAGTAC
C.ZA.03.03ZASK098B1.AY878061     CCAGAGGAG- --ATCTCTCG ACGCAGG--A CTCGGCTTGC TGAA------ GTGCACTCGG CAAGAGGCGA GAG-----CG GCGG---CTG --GTGAGTAC
C.ZA.03.03ZASK103B1.DQ164106     CCAGAGGAG- --ATCTCTCG ACGCAGG--A CTCGGCTTGC TGAA------ GTGCACTCGG CAAGAGGCGA GAG-----CG GCGA---CTG --GTGAGTAC
C.ZA.03.03ZASK104B1.DQ396395     CCAGAGGAG- --ATCTCTCG ACGCAGG--A CTCGGCTTGC TGAA------ GTGCACTCGG CAAGAGGCGA GAG-----GG GCGA---CTG --GTGAGTAC
C.ZA.03.03ZASK107B1.DQ056410     CCAGAGAAG- --ATCTCTCG ACGCAGG--A CTCGGCTTGC TGAA------ GTGCACTCGG CAAGAGGCGA GAG-----CG GCGA---CTG --GTGAGTAC
C.ZA.03.03ZASK110B1.DQ056411     CCAGAGAAG- --ATCTCTCG ACGCAGG--A CTCGGCTTGC TGAA------ GTGCACTCGG CAAGAGGCGA GAG-----CG GCGA---CTG --GTGAGTAC
C.ZA.03.03ZASK111B1.DQ056404     CCAGAGAAG- --ATCTCTCG ACGCAGG--G CTCGGCTTGC TGAA------ GTGCACTCGG CAGGAGGCGA GAG-----GG GCGA---CTG --GTGAGTAC
C.ZA.03.03ZASK113B1.DQ351237     CCAGAGGAG- --ATCTCTCG ACGCAGG--G CTCGGCTTGC TGAA------ GTGCACTCGG CAAGAGGCGA GAG-----CG GCGG---CTG --GTGAGTAC
C.ZA.03.03ZASK117B1.DQ056408     CCAGAGGAG- --ATCTCTCG ACGCAGG--A CTCGGCTTGC TGAA------ GTGCACTCGG CAAGAGGCGA GAG-----CG GCGA---CTG --GTGAGTAC
C.ZA.03.03ZASK118B1.DQ011169     CCAGAGGAG- --ATCTCTCG ACGCAGG--A CTCGGCTTGC TGAA------ GTGCACTCGG CAAGAGGCGA GAG-----CG GCGA---CTG --GTGAGTAC
C.ZA.03.03ZASK120B1.DQ011176     CCAGAGGAG- --ATCTCTCG ACGCAGG--A CTCGGCTTGC TGAA------ GTGCACTCGG CAAGAGGCGA GAG-----CG GCGG---CTG --GTGAGTAC
C.ZA.03.03ZASK211B1.DQ093601     CCAGAGAAG- --ATCTCTCG ACGCAAG--A CTCGGCTTGC TGAA------ GTGCACTCGG CAAGAGGCGA GAG-----CG GCGA---CTG --GTGAGTAC
C.ZA.03.03ZASK212B.DQ978981      CCAGAGGAG- --ATCTCTCG ACGCAGG--A CTCGGCTTGC TGAA------ GTGCACTCGG CAAGAGGCGA GAG-----CG GCGA---CTG --GTGAGTAC
C.ZA.03.03ZASK213B1.DQ093607     CCAGAGGAG- --ATCTCTCG ACGCAGG--A CTCGGCTTGC TGAA------ GTGCACTCGG CAAGAGGCGA GAGG----CG GCGG---CGG --GTGAGTAC
C.ZA.03.03ZASK215M6W.DQ275661    CCAGAGGAG- --ATCTCTCG ACGCAGG--A CTCGGCTTGC TGAA------ GTGCACTCGG CAAGAGGCGA GAG-----CG GCGG---CTG --GTGAGTAC
C.ZA.03.03ZASK223B1.DQ093597     CCAGAGGAG- --ATCTCTCG ACGCAGG--A CTCGGCTTGC TGAA------ GTGCACTCGG CAAGAGGCGA GAG-----CG GCGA---CTG --GTGAGTAC
C.ZA.03.03ZASK224MB1.DQ275664    CCAGAGGAG- --ATCTCTCG ACGCAGG--A CTCGGCTTGC TGAA------ GTGCACTCGG CAAGAGGCGA GAG-----CG GCGG---CTG --GTGAGTAC
C.ZA.03.03ZASK226B1.DQ164108     CCAGAGAAGA --AGAACTCT CGACGCAGGG CTCGGCTTGC TGAA------ GTGCACTCGG CAAGAGGCGA GAG-----GG GCGA---CTG --GTGAGTAC
C.ZA.03.03ZASK232B1.DQ093589     CCAGAGCAG- --ATCTCTCG ACGCAGG--A CTCGGCTTGC TGAA------ GTGCACTCGG CAAGAGGCGA GGGGAG--CG GCGG---CTG --GTGAGTAC
C.ZA.03.03ZASK233B1.DQ351234     CCAGAGGAG- --ATCTCTCG ACGCAGG--A CTCGGCTTGC TGAA------ GTGCACTCGG CAAGAGGCGA GAG-----GG GCGG---CTG --GTGAGTAC
C.ZA.03.04ZAPS194MB1.DQ275650    CCAGAGGAG- --ATCTCTCG ACGCAGG--G CTCGGCTTGC TGAA------ GTGCACTCGG CAAGAGGCGA GAG-----CG GCGA---CTG --GTGAGTAC
C.ZA.03.04ZASK082B1.AY901972     CCAGAGAAG- --TTCTCTCG ACGCAGG--A CTCGGCTTGC TGAA------ GTGCACTCGG CAAGAGGCGA GG------CG GCGGCGGCTG --GTGAGTAC
C.ZA.03.SK023B2.AY772690         CCAGAGGAG- --ATCTCTCG ACGCAGG--A CTCGGCTTGC TGAA------ GTGCACTCGG CAAGAGGCGA GAG-----CG GCGA---CTG --GTGAGTAC
C.ZA.03.SK029B2.AY772691         CCAGAGGAG- --ATCTCTCG ACGCAGG--A CTCGGCTTGC TGAA------ GTGCACTCAG TAAGAGGCGA GAG-----CG GCGA---CTG --GTGAGTAC
C.ZA.03.SK033B2.AY772692         CCAGAGGAG- --ATCTCTCG ACGCAGG--A CTCGGCTTGC TGAA------ GTGCACTCGG CAAGAGGCGA GAG-----CG GCGG---CTG --GTGAGTAC
C.ZA.03.SK040B1.AY703908         CCAGAGCAG- --ATCTCTCG ACGCAGG--A CTCGGCTTGC TGAA------ GTGCACTCGG CAAGAGGCGA GAG-----CG GCGA---CTG --GTGAGTAC
C.ZA.03.SK041B1.AY772693         CCAGAGGAG- --GTCTCTCG ACGCAGG--A CTCGGCTTGC TGAA------ GTGCACTCGG CAAGAGGCGA GAG-----CG GCGG---CTG --GTGAGTAC
C.ZA.03.SK043B1.AY772700         CCAGAGGAG- --ATCTCTCG ACGCAGG--A CTCGGCTTGC TGAA------ GTGCACTCGG CAAGAGGCGA GAG-----CG GCGA---CTG --GTGAGTAC
C.ZA.03.SK065B1.AY772694         CCAGAGGAG- --ATCTCTCG ACGCAGG--A CTCGGCTTGC TGAA------ GTGCACTCGG CAGGAGGCGA GGG-----GG GCGA---CTG --GTGAGTAC
C.ZA.03.SK112B1.AY772695         CCAGAGGAG- --ATCTCTCG ACGCAGG--A CTCGGCTTGC TGAA------ GTGCACACGG CAAGAGGCGA GAG-----CG GCGG---CTG --GTGAGTAC
C.ZA.03.SK116B1.AY772696         CCAGAGAAG- --ATCTCTCG ACGCAGG--A CTCGGCTTGC TGAA------ GTGCACTCGG CAAGAGGCGA GAG-----CG GCGG---CTG --GTGAGTAC
C.ZA.04.04ZAPS157MB1.DQ351232    CCAGAGAAG- --ATCTCTCG ACGCAGG--A CTCGGCTTGC TGAA-----A GTGCACTCGG CAAGAGGCGA GAG-----GG GCGA---CTG --GTGAGTAC
C.ZA.04.04ZAPS160B1.DQ164107     CCAGAGTAG- --ATCTCTCG ACGCAGG--A CTCGGCTTGC TGAA------ GTGCACTCGG CAAGAGGCGA GAG-----CG GCGA---CTG --GTGAGTAC
C.ZA.04.04ZAPS161B1.DQ164115     CCAGAGGAG- --ATCTCTCG ACGCAGG--A CTCGGCTTGC TGAA------ GTGCACTCGG CAAGAGGCGA GAG-----GG GCGA---CTG --GTGAGTAC
C.ZA.04.04ZAPS165MB1.DQ351221    CCAGAGGAG- --ATCTCTCG ACGCAGG--A CTCGGCTTGC TGAA------ GTGCACTCGG CAAGAGGCGA GAG-----GG GCGA---CTG --GTGAGTAC
C.ZA.04.04ZAPS168B1.DQ164121     CCAGAGGAG- --ATCTCTCG ACGCAGG--A CTCGGCTTGC TGAA------ GTGCACTCAG CAAGAGGCGA GAG-----CG GCGG---CTG --GTGAGTAC
C.ZA.04.04ZAPS169MB1.DQ396393    CCAGAGGAG- --ATCTCTCG ACGCAGG--A CTCGGCTTGC TGAA------ GTGCACTTGG CAAGAGGCGA GAG-----CG GCGG---CTG --GTGAGTAC
C.ZA.04.04ZAPS172MB1.DQ275659    CCAGAGGAG- --ATCTCTCG ACGCAGG--A CTCGGCTTGC TGAA------ GTGCACTCGG CAAGAGGCGA GAG-----CG GCGG---CTG --GTGAGTAC
C.ZA.04.04ZAPS177MB1.DQ396394    CCAGAGGAG- --ATCTCTCG ACGCAGG--A CTCGGCTTGC TGAA------ GTGCACTCGG CAAGAGGCGA GAG-----CG GCGG---CTG --GTGAGTAC
C.ZA.04.04ZAPS188B1.DQ164122     CCAGAGGAG- --ATCTCTCG ACGCAGG--A CTCGGCTTGC TGAA------ GTGCACTCGG CAAGAGGCGA GAG-----CG GCGG---CTG --GTGAGTAC
C.ZA.04.04ZAPS189B1.DQ164117     CCAGAGGAG- --ATCTCTCG ACGCAGG--A CTCGGCTTGC TGAA------ GTGCACTCGG CAAGAGGCGA GAG-----CG GCGG---CTG --GTGAGTAC
C.ZA.04.04ZAPS190B1.DQ093602     CCAGAGAAG- --ATCTCTCG ACGCAGG--A CTCGGCTTGC TGAA------ GTGCACTCGG CAAGAGGCGA GAG-----CG GCGG---CTG --GTGAGTAC
C.ZA.04.04ZAPS195B1.DQ164118     CCAGAGGAG- --ATCTCTCG ACGTAGG--A CTCGGCTTGC TGAA------ GTGCACTCGG CAAGAGGCGA GAG-----CG GCGA---CTG --GTGAGTAC
C.ZA.04.04ZAPS197MB1.DQ369997    CCAGAGAAG- --ATCTCTCG ACGCAGG--A CTCGGCTTGC TGAA------ GTGCACTCGG CAAGAGGCGA GAG-----CG GCGG---CTG --GTGAGTAC
C.ZA.04.04ZAPS198MB1.DQ445637    CCAGAGGAG- --ATCTCTCG ACGCAGG--A CTCGGCTTGC TGAA------ GTGCACTCGG CAAGAGGCGA GAG-----CG GCGA---CTG --GTGAGTAC
C.ZA.04.04ZAPS199B1.DQ164110     CCAGAGAAG- --ATCTCTCG ACGCAGG--A CTCGGCTTGC TGAA------ GTGCACTCGG CAAGAGGCGA GAG-----GG GCGA---CTG --GTGAGTAC
C.ZA.04.04ZAPS202B1.DQ093598     CCAGAGGAG- --ATCTCTCG ACGCAGG--A CTCGGCTTGC TGAA------ GTGCACTTGG CAAGAGGCGA GAG-----CG GCGG---CTG --GTGAGTAC
C.ZA.04.04ZAPS205B1.DQ093599     CCAGAGAAG- --AACTCTCG ACGCAGG--G CTCGGCTTGC TGAA------ GTGCACTCGG CAAGAGGCGA GAG-----GG GCGA---CTG --GTGAGTAC
C.ZA.04.04ZAPS206B1.DQ164126     CCAGCGGAG- --ATCTCTCG ACGCAGG--A CTCGGCTTGC TGAA------ GTGCACACGG CAAGAGGCGA GAG-----CG GCGG---CTG --GTGAGTAC
C.ZA.04.04ZAPS208B1.DQ093603     CCAGAGAAG- --ATCTCTCG ACGCAGG--A CTCGGCTTGC TGAA------ GTGCACTCGG CAAGAGGCGA GAG-----CG GCGG---CTG --GTGAGTAC
C.ZA.04.04ZAPS214B1.DQ093588     CCAGAGAAG- --ATCTCTCG ACGCAGG--A CTCGGCTTGC TGAA-----A GTGCACTCGG CAAGAGGCGA GAG-----CG GCGA---CTG --GTGAGTAC
C.ZA.04.04ZAPS216B1.DQ093600     CCAGAGAAG- --ATCTCTCG ACGCAGG--A CTCGGCTTGC TGAC------ GTGCACTCGG CAAGAGGCGA GAG-----CG GCGA---CTG --GTGAGTAC
C.ZA.04.04ZAPS217B1.DQ164119     CCAGAGAAG- --ATCTCTCG ACGCAGG--A CTCGGCTTGC TGAA------ GTGCACTCGG CAAGAGGCGA GAGG----CG GCGG---CTG --GTGAGTAC
C.ZA.04.04ZASK031B2.AY901974     CCAGAGAAG- --ATCTCTCG ACGCAGG--A CTCGGCTTGC TGAA------ GTGCACTCGG CAGGAGGCGA GAG-----CG GCGA---CTG --GTGAGTAC
C.ZA.04.04ZASK083B2.DQ093585     CCAGAGGAG- --ATCTCTCG ACGCAGG--A CTCGGCTTGC TGAA-----A GTGCACTCGG CAAGAGGCGA GGG-----GG GCGA---CTG --GTGAGTAC
C.ZA.04.04ZASK127B1.DQ011172     CCAGAGGAG- --ATCTCTCG ACGCAGG--A CTCGGCTTGC TGAA------ GTGCACTCGG CAAGAGGCGA GAG-----CG GCGA---CTG --GTGAGTAC
C.ZA.04.04ZASK128B1.DQ093586     CCAGAGGAG- --ATCTCTCG ACGCAGG--A CTCGGCTTGC TGAA------ GTGCACTCGG CAAGAGGCGA GAG-----CG GCGG---CTG --GTGAGTAC
C.ZA.04.04ZASK131B1.DQ056412     CCAGAGAAG- --ATCTCTCG ACGCAGG--A CTCGGCTTGC TGAA------ GTGCACTCGG CAAGAGGCGA GAG-----CG GCGG---CTG --GTGAGTAC
C.ZA.04.04ZASK132B1.DQ093594     CCAGAGAAG- --TTCGCTCG ACGCAGG--A CTCGGCTTGC TGAA------ GTGCACTCGG CAAGAGGCGA GAG-----CG GCGA---CTG --GTGAGTAC
C.ZA.04.04ZASK135B1.DQ011166     CCAGAGAAG- --ATCTCTCG ACGCAGG--A CTCGGCTTGC TGAA------ GTGCACTCGG CAAGAGGCGA GAG-----CG GCGG---CTG --GTGAGTAC
C.ZA.04.04ZASK136B1.DQ011177     CCAGAGGAG- --ATCTCTCG ACGCAGG--G CTCGGCTTGC TGAA------ GTGCACTCGG CAAGAGGCGA GAGG----CG GCGA---CTG --GTGAGTAC
C.ZA.04.04ZASK139B1.AY878072     CCAGAGGAG- --ATCTCTCG ACGCAAG--A CTCGGCTTGC TGAA------ GTGCACTCGG CAAGAGGCGA GAG-----CG GCGA---CTG --GTGAGTAC
C.ZA.04.04ZASK142B1.DQ011167     CCAGAGGAG- --ATCTCTCG ACGCAGG--A CTCGGCTTGC TGAA------ GTGCACTCAG CAGGAGGCGA GGGG----CG GCGG---CTG --GTGAGTAC
C.ZA.04.04ZASK145B1.AY901976     CCAGAGGAG- --ATCTCTCG ACGCAGG--A CTCGGCTTGC TGAA------ GTGCACTCGG CAAGAGGCGA GAG-----CG GCGG---CTG --GTGAGTAC
C.ZA.04.04ZASK146.AY772699       CCAGAGAAG- --ATCTCTCG ACGCAGG--A CTCGGCTTGC TGAA------ GTGCACTCGG CAAGAGGCGA GAG-----CG GCGA---CTG --GTGAGTAC
C.ZA.04.04ZASK147B1.DQ164114     CCAGAGGAG- --ATCTCTCG ACGCAGG--A CTCGGCTTGC TGAA------ GTGCACTCGG CAAGAGGCGA GAG-----CG GCGG---CTG --GTGAGTAC
C.ZA.04.04ZASK148B1.DQ093590     CCAGAGAAG- --ATCTCTCG ACGCAGG--A CTCGGCTTGC TGAA-----A GTGCACTCGG CAAGAGGCGA GGG-----CG GCGG---CTG --GTGAGTAC
C.ZA.04.04ZASK150B1.DQ011178     CCAGAGAAG- --ATCTCTCG ACGCAGG--A CTCGGCTTGC TGAA-----A GTGCACTCGG CAAGAGGCGA GGGGA---AG GCGG---CTG --GTGAGTAC
```

```
C.ZA.04.04ZASK151B1.AY901977     CCAGAGGAG- --ATCTCTCG ACGCAGG--A CTCGGCTTGC TGAA------ GTGCACTCGG CAAGAGGCGA GAG-----CG GCGA---CTG --GTGAGTAC
C.ZA.04.04ZASK154B1.AY878071     CCAGAGAAG- --ATCTCTCG ACGCAGG--A CTCGGCTTGC TGAA------ GTGCACTCGG CAAGAGGCGA GAGG----CG GCGG---CTG --GTGAGTAC
C.ZA.04.04ZASK155B1.AY901978     CCAGAGGAG- --ATCTCTCG ACGCAGG--A CTCGGCTTGC TGAA------ GTGCACACGG CAAGAGGCGA GAG-----GG GCGG---CTG --GTGAGTAC
C.ZA.04.04ZASK156B1.DQ011171     CCAGAGAAG- --ATCTCTCG ACGCAGG--A CTCGGCTTGC TGAA------ GTGCACTCGG CAAGAGGCGA GAG-----CG GCGA---CTG --GTGAGTAC
C.ZA.04.04ZASK159B1.DQ011179     CCAGAGGAG- --ATCTCTCG ACACAGG--A CTCGGCTTGC TGAG------ GTGCACACAG CAAGAGGCGA GGG-----GG GCGA---CTG --GTGAGTAC
C.ZA.04.04ZASK160B1.DQ011173     CCAGAGAAG- --ATCTCTCG ACGCAGG--A CTCGGCTTGC TGAA------ GTGCACTCGG CAAGAGGCGA GAG-----CG GCGA---CTG --GTGAGTAC
C.ZA.04.04ZASK161B1.DQ011170     CCAGAGGAG- --ATCTCTCG ACGCAGG--A CTCGGCTTGC TGAA------ GTGCACTCGG CAAGAGGCGA GAG-----AG GCGA---CTG --GTGAGTAC
C.ZA.04.04ZASK163B1.AY901979     CCAGAGGAG- --ATCTCTCG ACGCAGG--A CTCGGCTTGC TGAA------ GTGCACTCGG CAAGAGGCGA GAG-----CG GCGA---CTG --GTGAGTAC
C.ZA.04.04ZASK164B1.DQ056405     CCAGAGAAG- --ATCTCTCG ACGCAGG--A CTCGGCTTGC TGAA------ GTGCACTCGG CAAGAGGCGA GAG-----CG GCGG---CTG --GTGAGTAC
C.ZA.04.04ZASK165B1.DQ396387     CCAGAGGAG- --ATCTCTCG ACGCAGG--A CTCGGCTTGC TGAA------ GTGCACTCGG CAAGAGGCGA GAG-----GG GCGG---CTG --GTGAGTAC
C.ZA.04.04ZASK167B1.DQ164127     CCAGAGGAG- --ATCTCTCG ACGCAGG--A CTCGGCTTGC TGAA------ GTGCACTCGG CAAGAGGCGA GAG-----CG GCGGA--CTG --GTGAGTAC
C.ZA.04.04ZASK168B1.AY878058     CCAGAGGAG- --ATCTCTCG ACGCAGG--A CTCGGCTTGC TGAA------ GTGCACTCGG CAAGAGGCGA GAG-----CG GCGA---CTG --GTGAGTAC
C.ZA.04.04ZASK169B1.DQ396381     CCAGAGGAG- --ATCTCTCG ACGCAGG--A CTCGGCTTGC TGAA------ GTGCACTCGG CAAGAGGCGA GAG-----GG GCGG---CTG --GTGAGTAC
C.ZA.04.04ZASK170B1.DQ093595     CCAGAGGAG- --ATCTCTCG ACGCAGG--A CTCGGCTTGC TGAA------ GTGCACTCGG CAAGAGGCGA GAG-----CG GCGG---CTG --GTGAGTAC
C.ZA.04.04ZASK171B1.DQ351217     CCAGAGGAG- --ATCTCTCG ACGCAGG--A CTCGGCTTGC TGAA------ GTGCACTCGG CAAGAGGCGA GAG-----CG GCGG---CTG --GTGAGTAC
C.ZA.04.04ZASK172B1.DQ369998     CCAGAGAAG- --ATCTCTCG ACGCAGG--A CTCGGCTTGC TGAA------ GTGCACTCGG CAAGAGGCGA GAG-----GG GCGA---CTG --GTGAGTAC
C.ZA.04.04ZASK173B1.DQ093604     CCAGAGGAG- --ATCTCTCG ACGCAGG--A CTCGGCTTGC TGAA------ GTGCACTCGG CAAGAGGCGA GAG-----CG GCGG---CTG --GTGAGTAC
C.ZA.04.04ZASK174B1.AY901980     CCAGAGGAG- --ATCTCTCG ACGCAGG--A CTCGGCTTGC TGAA------ GTGCACTCGG CAAGAGGCGA GGGG----CG GCGG---CTG --GTGAGTAC
C.ZA.04.04ZASK175B1.DQ164129     CCAGAGGAG- --ATCTCTCG ACGCAGG--A CTCGGCTTGC TGAA------ GTGCACTCGG CAAGGGGCGA GAG-----CG GCGA---CTG --GTGAGTAC
C.ZA.04.04ZASK176B1.DQ056416     CCAGAGGAG- --ATCTCTCG ACGCAGG--A CTCGGCTTGC TGAA------ GTGCACTCGG CAAGAGGCGA GAG-----CG GCGA---CTG --GTGAGTAC
C.ZA.04.04ZASK178B1.DQ093587     CCAGAGAAG- --ATCTCTCG ACGCAAG--A CTCGGCTTGC TGAA------ GTGCACTCGG CAAGAGGCGA GAG-----CG GCGA---CTG --GTGAGTAC
C.ZA.04.04ZASK180B1.AY878059     CCAGAGAAG- --ATCTCTCG ACGCAGG--A CTCGGCTTGC TGAA------ GTGCACTCGG CAAGAGGCGA GAG-----CG GCGA---CTG --GTGAGTAC
C.ZA.04.04ZASK181B1.AY878062     CCAGAGGAG- --ATCTCTCG ACGCAGG--A CTCGGCTTGC TGAA------ GTGCACTCGG CAAGAGGCGA GGGG----CG GCGG---CTG --GTGAGTAC
C.ZA.04.04ZASK182B1.AY878054     CCAGAGGAG- --ATCTCTCG ACGCAGG--A CTCGGCTTGC TGAA------ GTGCACTCGG CAAGAGGCGA GAG-----CG GCGA---CTG --GTGAGTAC
C.ZA.04.04ZASK183B1.AY878055     CCAGAGGAG- --ATCTCTCG ACGCAGG--A CTCGGCTTGC TGAA------ GTGCACTCGG CAAGAGGCGA GAG-----CG GCGG---CTG --GTGAGTAC
C.ZA.04.04ZASK184B1.DQ056418     ACAGAGGAG- --ATCTCTCG ACGCAGG--A CTTGGCTTGC TGAA------ GTGCACGCGG CAAGAGGCGA GGG-----GG GCGA---CTG --GTGAGTAC
C.ZA.04.04ZASK185B1.DQ011174     CCAGAGAAG- --TTCTCTCG ACGCAGG--A CTCGGCTTGC TGAA------ GTGCACTCGG CAAGAGGCGA GAG-----CG GCGA---CTG --GTGAGTAC
C.ZA.04.04ZASK190B1.DQ056409     CCAGAGGAG- --CTCTCTCG ACGCAGG--A CTCGGCTTGC TGAA------ GTGCACTCGG CAAGAGGCGA GAG-----CG GCGA---CTG --GTGAGTAC
C.ZA.04.04ZASK191B1.DQ369993     CCAGAGGAG- --ATCTCTCG ACGCAGG--A CTCGGCTTGC TGAA------ GTGCACTCGG CAAGAGGCGA GAG-----GG GCGG---CTG --GTGAGTAC
C.ZA.04.04ZASK192B1.DQ396382     CCAGAGAAG- --ATCTCTCG ACGCAGG--A CTCGGCTTGC TGAA------ GTGCACTCGG CAAGAGGCGA GAG-----CG GCGG---CTG --GTGAGTAC
C.ZA.04.04ZASK193B1.DQ396396     CCAGAGGAG- --ATCTCTCG ACGCAGG--G CTCGGCTTGC TGAA------ GTGCACTCGG CAAGAGGCGA GAG-----CG GCGG---CTG --GTGAGTAC
C.ZA.04.04ZASK196B1.DQ056413     CCAGAGGAG- --ATCTCTCG ACGCAGG--A CTCGGCTTGC TGAA------ GTGCACTCGG CAAGAGGCGA GAG-----CG GCGG---CTG --GTGAGTAC
C.ZA.04.04ZASK200B1.DQ396383     CCAGAGGAG- --ATCTCTCG ACGCAAG--A CTCGGCTTGC TGAA------ GTGCACTCGG CAAGAGGCGA GAG-----CG GCGG---CTG --GTGAGTAC
C.ZA.04.04ZASK201B1.DQ396397     CCAGAGGAG- --ATCTCTCG ACGCAGG--A CTCGGCTTGC TGAA------ GTGCACTCGG CAAGAGGCGA GAG-----CG GCGA---CTG --GTGAGTAC
C.ZA.04.04ZASK202B1.DQ011180     CCAGAGAAG- --ATCTCTCG ACGCAGG--A CTCGGCTTGC TGAA------ GTGCACTCGG CAAGAGGCGA GAG-----CG GCGG---CTG --GTGAGTAC
C.ZA.04.04ZASK204B1.DQ056414     CCAGAGGAG- --ATCTCTCG ACGCAAG--A CTCGGCTTGC TGAA------ GTGCACTCGG CAAGAGGCGA GAG-----CG GCGG---CTG --GTGAGTAC
C.ZA.04.04ZASK206B1.DQ056415     CCAGAGAAG- --ATCTCTCG ACGCAGG--A CTCGGCTTGC TGAA------ GTGCACGCGG CAAGAGGCGA GAG-----CG GCGG---CTG --GTGAGTAC
C.ZA.04.04ZASK208B1.DQ056406     CCAGAGGAG- --AACTCTCG ACGCAGG--A CTCGGCTTGC TGAA------ GTGCACTCGG CAAGAGGCGA GAG-----CG GCGG---CTG --GTGAGTAC
C.ZA.04.04ZASK217B1.DQ056417     CCAGAGAAG- --ATCTCTCG ACGCAGG--A CTCGGCTTGC TGAA------ GTGCACTCGG CAAGAGGCGA GAG-----GG GCGG---CTG --GTGAGTAC
C.ZA.04.04ZASK234B1.DQ093605     CCAGAGGAG- --ATCTCTCG ACGCAGG--A CTCGGCTTGC TGAA------ GTGCACTCGG CAAGAGGCGA GAG-----CG GCGA---CTG --GTGAGTAC
C.ZA.04.CAP30_5w_F4.GQ999973     CCAGAGGAG- --ATCTCTCG ACGCAGG--A CTCGGCTTGC TGAA------ GTGCACTCGG CAAGAGGCGA GAG-----CG GCGA---CTG --GTGAGTAC
C.ZA.04.CAP61_8w_F3.GQ999975     CCAGAGAAG- --ATCTCTCG ACGCAGG--G CTCGGCTTGC TGAA------ GTGCACTCGG CAGGAGGCGA GAG-----GG GCGG---CTG --GTGAGTAC
C.ZA.04.SK133B1.AY772698         CCAGAGAAG- --ATCTCTCG ACGCAGG--A CTCGGCTTGC TGAA------ GTGCACTCGG CAAGAGGCGA GAG-----CG GCGG---CTG --GTGAGTAC
C.ZA.04.SK134B1.AY703909         CCAGAGGAG- --ATCTCTCG ACGCAGG--A CTCGGCTTGC TGAA------ GTGCACACGG CAAGAGGCGA GAG-----CG GCGG---CTG --GTGAGTAC
C.ZA.04.SK140B1.AY901973         CCAGAGAAG- --ATCTCTCG ACGCAGG--A CTCGGCTTGC TGAA-----A GTGCACTCGG CAAGAGGCGA GAG-----CG GCGA---CTG --GTGAGTAC
C.ZA.04.SK143B1.AY703910         CCAGAGGAG- --ATCTCTCG ACGCAGG--A CTCGGCTTGC TGAA------ GTGCACTCGG CGAGAGGCGA GAG-----CG GCGG---CTG --GTGAGTAC
C.ZA.04.SK144B1.AY703911         CCAGAGAAG- --ATCTCTCG ACGCAGG--A CTCGGCTTGC TGAA------ GTGCACTCGG CAAGAGGCGA GAG-----CG GCGG---CTG --GTGAGTAC
C.ZA.05.05ZAPSK240B1.DQ369991    CCAGAGGAG- --ATCTCTCG ACGCAAG--A CTCGGCTTGC TGAA------ GTGCACTCGG CAAGAGGCGA GAG-----CG GCGA---CTG --GTGAGTAC
C.ZA.05.05ZASK243B1.DQ396372     CCAGAGGAG- --ATCTCTCG ACGCAGG--G CTCGGCTTGC TGAA------ GTGCACTCGG CAAGAGGCGA GAG-----CG GCGG---CTG --GTGAGTAC
C.ZA.05.05ZASK244B1.DQ369992     CCAGAGAAG- --ATCTCTCG ACGCAAG--A CTCGGCTTGC TGAA------ GTGCACTCGG CAAGAGGCGA GAG-----CG GCGG---CTG --GTGAGTAC
C.ZA.05.05ZASK245B1.DQ369982     CCAGAGGAG- --ATCTCTCG ACGCAGG--A CTCGGCTTGC TGAA------ GTGCACTCGG CAAGAGGCGA GAG-----CG GCGG---CTG --GTGAGTAC
C.ZA.05.05ZASK246B1.DQ369983     CCAGAGGAG- --ATCTCTCG ACGCAAG--A CTCGGCTTGC TGAA------ GTGCACTCGG CAAGAGGCGA GAG-----CG GCGG---CTG --GTGAGTAC
C.ZA.05.05ZASK247B1.DQ369994     CCAGAGGAG- --ATCTCTCG ACGCAGG--A CTCGGCTTGC TGAA------ GTGCACTCGG CAGGAGGCGA GAG-----CG GCGG---CTG --GTGAGTAC
C.ZA.05.CAP174_4w.GQ999981       CCAGAGGAG- --GTCTCTCG ACGCAAG--A CTCGGCTTGC TGAA------ GTGCACTCGG CAAGAGGCGA GAG-----CG GCGG---CTG --GTGAGTAC
C.ZA.05.CAP206_8w_F1.GQ999982    CCAGAGGAG- --ATCTCTCG ACGCAGG--A CTCGGCTTGC TGAA------ GTGCACTCGG CAAGAGGCGA GAG-----CG GCGA---CTG --GTGAGTAC
C.ZA.05.CAP210_5w.GQ999983       CCAGAGGAG- --ATCTCTCG ACGCAGG--A CTCGGCTTGC TGAA------ GTGCACACGG CAAGAGGCGA GAG-----CG GCGG---CTG --GTGAGTAC
C.ZA.05.CAP228_8w_F2.GQ999984    CCAGAGAAG- --ATCTCTCG ACGCAGG--A CTCGGCTTGC TGAA------ GTGCACTCGG CAAGAGGCGA GAG-----CG GCGA---CTG --GTGAGTAC
C.ZA.05.CAP229_7w.GQ999985       CCAGAGAAG- --ATCTCTCG ACGCAGG--A CTCGGCTTGC TGAA------ GTGCACTCGG CAAGAGGCGA GAG-----CG GCGG---CTG --GTGAGTAC
C.ZA.05.CAP239_5w_F1.GQ999991    CCAGAGGAG- --ATCTCTCG ACGCAGG--A CTCGGCTTGC TGAA------ GTGCACTCGG CAAGAGGCGA GAG-----CG GCGA---CTG --GTGAGTAC
C.ZA.05.CAP244_8w_F1.GQ999986    CCAGAGAAG- --ATCTCTCG ACGCAGG--A CTCGGCTTGC TGAA-----A GTGCACGCGG CAAGAGGCGA GGG-----GG GCGA---CTG --GTGAGTAC
C.ZA.05.CAP248_9w.GQ999987       CCAGAGGAG- --ATCTCTCG ACGCAGG--A CTCGGCTTGC TGAA------ GTGCACTCGG CAAGAGGCGA GAG-----CG GCGG---CTG --GTGAGTAC
C.ZA.05.CAP255_8w_F1.GQ999988    CCAGAGAAG- --ATCTCTCG ACGCAGG--A CTCGGCTTGC TAAA------ GTGCACTTAG CAAGAGGCGA GAG-----CG GCGA---CTG --GTGAGTAC
C.ZA.05.CAP256_6w.GQ999989       CCAGAGAAG- --ATCTCTCG ACGCAGG--A CTCGGCTTGC TGAA------ GTGCACTCGG CAAGAGGCGA GAG-----GG GCGG---CTG --GTGAGTAC
C.ZA.05.CAP257_7w_F1.GQ999990    CCAGAGGAG- --ATCTCTCG ACGCAGG--A CTCGGCTTGC TGAA------ GTGCACTCGG CAAGAGGCGA GAG-----CG GCGA---CTG --GTGAGTAC
C.ZA.05.CAP45_5w_F1.GQ999974     CCAGAGGAG- --ATCTCTCG ACGCAGG--A CTCGGCTTGC TGAA------ GTGCACTCGG CAAGAGGCGA GAG-----CG GCGA---CTG --GTGAGTAC
C.ZA.05.CAP63_5w_F4.GQ999976     CCAGAGAAG- --TTCTCTCG ACGCAGG--A CTCGGCTTGC TGAA------ GTGCACTCGG CAAGAGGCGA GAG-----CG GCGG---CTG --GTGAGTAC
C.ZA.05.CAP65_6w.GQ999977        CCAGAGAAG- --ATCTCTCG ACGCAGG--A CTCGGCTTGC TGAA------ GTGCACTCGG CAAGAGGCGA GAG-----CG GCGG---CTG --GTGAGTAC
C.ZA.05.CAP84_3w_F2.GQ999978     CCAGAGGAG- --ATCTCTCG ACGCAGG--A CTCGGCTTGC TGAA------ GTGCACTCGG CAAGAGGCGA GAG-----CG GCGG---CTG --GTGAGTAC
C.ZA.05.CAP85_5w_F1.GQ999979     CCAGAGAAG- --AACTCTCG ACGCAGG--A CTCGGCTTGC TGAA------ GTGCACTCGG CAAGAGGCGA GGGG----AG GCGA---CTG --GTGAGTAC
C.ZA.05.CAP88_5w_F2.GQ999980     CCAGAGGAG- --ATCTCTCG ACGCAAG--A CTCGGCTTGC TGAA------ GTGCACTCGG CAAGAGGCGA GAG-----CG GCGA---CTG --GTGAGTAC
C.ZA.05.CAP8_3w_F2.GQ999972      CCAGAGAAG- --ATCTCTCG ACGCAGG--A CTCGGCTTGC TGAA------ GTGCACACGG CAAGAGGCGA GAG-----CG GCGG---CTG --GTGAGTAC
C.ZA.07.DEMC07ZA011.JX140664     CCAGAGGAG- --ATCTCTCG ACGCAGG--A CTCGGCTTGC TGAA------ GTGCACTCGG CAAGAGGCGA GAG-----CG GCGG---CTG --GTGAGTAC
C.ZA.08.DEMC08ZA011.JX140666     CCAGAGGAG- --ATCTCTCG ACGCAGG--A CTCGGCTTGC TGAA------ GTGCACCCGG CAGGAGGCGA GAG-----CG GCGG---CTG --GTGAGTAC
C.ZA.09.DEMC09ZA008.JX140667     CCAGAGAAG- --ATCTCTCG ACGCAAGG-A CTCGGCTTGC TGAA------ GTGCACTCGG CAAGAGGCGA GAG-----CG GCGA---CTG --GTGAGTAC
C.ZA.09.DEMC09ZA009.JX140668     CCAGAGGAG- --ATCTCTCG ACGCAGG--A CTCGGCTTGC TGAA------ GTGCACTCGG CAAGAGGCGA GAG-----CG GCGA---CTG --GTGAGTAC
C.ZA.10.DEMC10ZA001.JX140669     CCAGAGGAG- --ATCTCTCG ACGCAGG--A CTCGGCTTGC TGAA------ GTGCACTCGG CAAGAGGCGA GGGG----CG GCGA---CTG --GTGAGTAC
C.ZA.90.pZAC_R3714.JN188292      CCAGAGGAG- --ATCTCTCG ACGCAGG--A CTCGGCTTGC TGAA------ GTGCACACGG CAAGAGGCGA GAG-----CG GCGG---CTG --GAGAGTAC
C.ZA.97.97ZA003.AY118165         CCAGAGGAG- --ATCTCTCG ACGCAGG--A CTCGGCTTGC TGAA-----A GTGCACTCGG CAAGAGGCGA GAG-----CG GCGG---CTG --GTGAGTAC
C.ZA.97.97ZA009.AY118166         CCAGAGAAG- --AGCTCTCG ACGCAGG--A CTCGGCTTGC TGAA------ GTGCACTCGG CAAGAGGCGA GAG-----AG GCGG---CTG --GTGAGTAC
C.ZA.97.97ZA012.AF286227         CCAGAGAAG- --ATCTCTCG ACGCAGG--A CTCGGCTTGC TGAA------ GTGCACTCGG CAAGAGGCGA GGGG----CG GCGA---CTG --GTGAGTAC
C.ZA.98.98ZA445.AY158533         CCAGAGGAG- --ATCTCTCG ACGCAGG--A CTCGGCTTGC TGAA------ GTGCACTCGG CAAGAGGCGA GAG-----CG GCGA---CTG --GTGAGTAC
C.ZA.98.98ZA502.AY158534         CCAGAGAAG- --ATCTCTCG ACGCAGG--A CTCGGCTTGC TGAA-----A GTGCACTCGG CAAGAGGCGA GAG-----CG GCGG---CTG --GTGAGTAC
C.ZA.98.98ZA528.AY158535         CCAGAGAAG- --ATCTCTCG ACGCAGG--A CTCGGCTTGC TGAA------ GTGCACTCGG CAAGAGGCGA GAG-----CG GCGG---CTG --GTGAGTAC
C.ZA.98.TV001.AY162223           CCAGAGGAG- --ATCTCTCG ACGCAGG--A CTCGGCTTGC TGAA------ GTGCACACGG CAAGAGGCGA GAG-----GG GCGG---CTG --GTGAGTAC
C.ZA.98.TV002.AY162224           CCAGAGAAG- --ATCTCTCG ACGCAGG--A CTCGGCTTGC TGAA------ GTGCACTCGG CAAGAGGCGA GGG-----GG GCGA---CTG --GTGAGTAC
C.ZA.98.TV012.AY162225           CCAGAGGAG- --ATCTCTCG ACGCAGG--A CTCGGCTTGC TGAA------ GTGCACTCGG CAAGAGGCGA GAG-----AG GCGG---CTG --GTGAGTAC
C.ZA.99.99ZACM9.AF411967         CCAGAGCAG- --ATCTCTCG ACGCAGG--A CTCGGCTTGC TGAA------ GTGCACTCGG CAAGAGGCGA GAG-----CG GCGA---CTG --GTGAGTAC
C.ZA.99.99ZALT21.EU293446        CCAGAGGAG- --ATCTCTCG ACGCAGG--A CTCGGCTTGC TGAA------ GTGCACTCGG CAAGAGGCGA GAG-----GG GCGG---CTG --GTGAGTAC
C.ZA.99.99ZALT39.EU293447        CCAGAGGAG- --ATCTCTCG ACGCAGG--A CTCGGCTTGC TGAA------ GTGCACTCGG CAAGAGGCGA GAG-----CG GCGG---CTG --GTGAGTAC
C.ZA.99.99ZALT42.EU293448        CCAGAGGAG- --ATCTCTCG ACGCAGG--A CTCGGCTTGC TGAA------ GTGCACTCGG CAAGAGGCGA GAG-----CG GCGG---CTG --GTGAGTAC
C.ZA.99.99ZALT45.EU293449        CCAGAGGAG- --ATCTCTCG ACGCAGG--A CTCGGCTTGC TGAA------ GTGCACTCGG CAAGAGGCGA GAG-----GG GCGG---CTG --GTGAGTAC
C.ZA.99.99ZALT46.EU293450        CCAGAGAAG- --ATCTCTCG ACGCAGG--A CTCGGCTTGC TGAA------ GTGCACTCGG CAAGAGGCGA GGG-----GG GCGA---CTG --GTGAGTAC
C.ZA.99.99ZALT4.EU293444         CCAGAGAAG- --ATCTCTCG ACGCAGG--A CTCGGCTTGC TGAA------ GTGCACTCGG CAAGAGGCGA GAG-----CG GCGG---CTG --GTGAGTAC
C.ZA.99.99ZALT5.EU293445         CCAGAGGAG- --ATCTCTCG ACGCAGG--A CTCGGCTTGC TGAA------ GTGCACTCGG CAAGAGGCGA GAG-----CG GCGA---CTG --GTGAGTAC
C.ZA.99.99ZATM10.AY228556        CCAGAGGAG- --ATCTCTCG ACGCAGG--A CTCGGCTTGC TGAA------ GTGCACTCGG CAAGAGGCGA GAG-----CG GCGG---CTG --GTGAGTAC
C.ZA.99.ZASW7.AF411966           CCAGAGAAG- --ATCTCTCG ACGCAGG--A CTCGGCTTGC TGAA------ GTGCACTCGG CAAGAGGCGA GGGG----CG GCGG---CTG --GTGAGTAC
C.ZM.02.02ZM108.AB254141         CCAGAGGAG- --AACTCTCG ACGCAGG--A CTCGGCTTGC TGAA------ GTGCACTCGG CAAGAGGCGA GAG-----CG GCGG---CTG --GTGAGTAC
C.ZM.02.02ZM110.AB254142         CCAGAGAAG- --ATCTCTCG ACGCAGG--A CTCGGCTTGC TGAA-----A GTGCACTCGG CAAGAGGCGA GGG-----GG GCGA---CTG --GTGAGTAC
C.ZM.02.02ZM114.AB254146         CCAGAGGAG- --ATCTCTCG ACGCAGG--A CTCGGCTTGC TGAA------ GTGCACTCGG CAAGAGGCGA GAG-----CG GCGG---CTG --GTGAGTAC
C.ZM.02.02ZM115.AB254148         CCAGAGAAG- --ATCTCTCG ACGCAGG--A CTCGGCTTGC TAAA------ GTGCACTTGG CAAGAGGCGA GAGG----CG GCGG---CTG --GTGAGTAC
C.ZM.02.02ZMBC.AB254149          CCAGAGGAG- --ATCTCTCG ACGCAGG--A CTCGGCTTGC TGAA------ GTGCACACGG CAAGAGGCGA GAG-----CG GCGG---CTG --GTGAGTAC
C.ZM.02.02ZMDB.AB254153          CCAGAGGAG- --ATCTCTCG ACGCAGG--A CTCGGCTTGC TGAA------ GTGCACTCGG CAAGAGGCGA GAG-----CG GCGG---CTG --GTGAGTAC
C.ZM.02.02ZMJC.AB254155          CCAGAGAAG- --ATCTCTCG GCGCAGG--A CTCGGCTTGC TGAA------ GTGCACTCGG CAAGAGGCGA GAG-----GG GCGG---CTG --GTGAGTAC
C.ZM.03.ZM246F_flA10.FJ496186    CCAGAGGAG- --ATCTCTCG ACGCAGG--A CTCGGCTTGC TGAA-----A GTGCACTCGG CAAGAGGCGA GAG-----CG GCGG---CTG --GTGAGTAC
C.ZM.03.ZM247F_flA1.FJ496195     CCAGAGGAG- --ATCTCTCG ACGCAGG--A CTCGGCTTGC TGAA------ GTGCACACGG CAAGAGGCGA GAG-----CG GCGA---CTG --GTGAGTAC
```

```
C.ZM.03.ZM249M_flC1.FJ496209     CCAGAGAAG- --ATCTCTCG ACGCAGG--A CTCGGCTTGC TGAA------ GTGCACTCGG CAAGAGGCGA GAG-----CG GCGG---CTG --GTGAGTAC
C.ZM.89.ZAM18.AB485645           CCAGAGGAG- --ATCTCTCG ACGCAGG--A CTCGGCTTGC TGAA------ GTGCACTCGG CAAGAGGCGA GAG-----CG GCGG---CTG --GTGAGTAC
C.ZM.96.96ZM651.AF286224         CCAGAGGAG- --ATCTCTCG ACGCAGG--A CTCGGCTTGC TGAA------ GTGCACTCGG CAAGAGGCGA GAG-----CG GCGG---CTG --GTGAGTAC
C.ZM.96.96ZM751.AF286225         CCAGAGGAG- --ATCTCTCG ACGCAGG--A CTCGGCTTGC TGAA------ GTGCACTCGG CAAGAGGCGA GAG-----CG GCGG---CTG --GTGAGTAC
C.ZM.x.HIV1084i.AY805330         CCAGAGGAG- --ATCTCTCG ACGCAGG--A CTCGGCTTGC TGAA------ GTGCACTCGG CAAGAGGCGA GAG-----CG GCGG---CTG --GTGAGTAC
D.CD.83.ELI.K03454               CCAGAGGAG- --CTCTCTCG ACGCAGG--A CTCGGCTTGC TGAA------ GCGCGCACGG CAAGAGGCGA GGGG----CA GCGA---CTG --GTGAGTAC
D.CD.83.NDK.M27323               CCAGAGAAG- --ATCTCTCG ACGCAGG--A CTCGGCTTGC TGAA------ GCGCGCACAG CAAGAGGCGA GGGG----CA GCGA---CTG --GTGAGTAC
D.CD.84.84ZR085.U88822           CCAGAGGAG- --ATCTCTCG ACGCAGG--A CTCGGCTTGC TGAA-----A GCGCGCTCGG CAAGAGGCGA GGGG----CA GCGA---CTG --GTGAGTAC
D.CD.85.Z2Z6_Z2_CDC_Z34.M22639   CCAGAGAAG- --CTCTCTCG ACGCAGG--A CTCGGCTTGC TGAA------ GCGCGCACGG CAAGAGGCGA GGGG----CA GCGA---CCG --GTGAGTAC
D.CM.10.DEMD10CM009.JX140670     CCAGAGGAG- --CTCTCTCG ACGCAGG--A CTCGGCTTGC TGA------- GCGCGCACGG CAAGAGGCGA GGGG----TA GCGA---CTG --GTGAGTAC
D.KE.93.MB2059.AF133821          CCAGAGGAG- --CTCTCTCG ACGCAGG--A CTCGGCTTGC TGAA------ GCGCGCACAG CAAGAGGCGA GGGG----CA GCGA---ACG --GTGAGTAC
D.KE.97.ML415_2.AY322189         CCAGAGGAG- --CTCTCTCG ACGCAGG--A CTCGGCTTGC TGAA------ GCGCGCACAG CAAGAGGCGA GGG-----CA GCGA---ACG --GTGAGTAC
D.KR.04.04KBH8.DQ054367          CCAGAGGAG- --CTCTCCCG ACGCAGG--A CTTGGCTTGC TGAA------ GCGCGCACAG CAAGAGGCGA GGGG----TA GCGA---CTG --GTGAGTAC
D.SN.90.SE365.AB485648           CCAGAGGAG- --CTCTCTCG ACGCAGG--A CTCGGCTTGC TGAA------ GCGCGCACGG CAAGAGGCGA GGGG----CA GCGA---CTG --GTGAGTAC
D.UG.05.p190049.JX236668         CCAGAGGAG- --CTCTCTCG ACGCAGG--A CTCGGCTTGC TGAA------ GCGCGCACGG CAAGAGGCGA GGGG----CA GCGA---ACG --GTGAGTAC
D.UG.07.p191647.JX236670         CCAGAGGAG- --CTCTCTCG ACGCAGG--A CTCGGCTTGC TGA------- GCGCGCACAG CAAGAGGCGA GGGG----CA GCGA---ACG --GTGAGTAC
D.UG.07.p191882.JX236673         CCAGAGAAG- --TTCTCTCG ACGCAGG--A CTCGGCTTGC TGA------- GCGCGCACAG CAAGAGGCGA GGGG----CA GCGA---CTG --GTGAGTAC
D.UG.07.pSC191727.JX236679       CCAGAGGAG- --CTCTCTCG ACGCAGG--A CTCGGCTTGC TGAA------ GCGCGCACAG CAAGAGGCGA GGGG----CA GCGA---ACG --GTGAGTAC
D.UG.08.p191859.JX236672         CCGGAGAAG- --ATCTCTCG ACGCAGG--A CTCGGCTTGC TGAA------ GCGCGCACAG CAAGAGGCGA GGGG----CA GCGA---ACC --GTGAGTAC
D.UG.91.UG270.AB485650           CCAGAGGAG- --CTCTCTCG ACGCAGG--A CTCGGCTTGC TGAA------ GCGCGCACGG CAAGAGGCGA GGGG----CA GCGA---ACG --GTGAGTAC
D.UG.92.92UG001.AJ320484         CCAGAGAAG- --CTCTCTCG ACGCAGG--A CTCGGCTTGC TGAG------ -CGCGCACGG CAAGAGGCGA GGGG----CA GCGA---CTG --GTGAGTAC
D.UG.94.94UG114.U88824           CCAGAGGAG- --ATCTCTCG ACGCAGG--A CTCGGCTTGC TGAA------ GCGCGCACGG CAAGAGGCGA GGGG----CA GCGG---AAG --GTGAGTAC
D.ZA.84.R2.AY773338              ---------- ---------- ---------- ---------- ---------- ---------- ---------- ---------- --GA---CTG --GTGAGTAC
D.ZA.85.R286.AY773340            ---------- ---------- ---------- ---------- ---------- ---------- ---------- ---------A GCGA---CTG --GTGAGTAC
D.ZA.86.R482.AY773341            ---------- ---------- ---------- ---------- ---------- ---------- ---------- ---------- ---A---CTG --GTGAGTAC
D.ZA.90.R1.EF633445              AAGTGAGGA- --GTCTCTCG CGCA---GGA CTCGGCTTGC TGAA------ GCGCGCGCGG CAAGAGGCGA GGGG----CA GCGA---CTG --GTGAGTAC
F1.AR.02.ARE933.DQ189088         ---------- --------TT GCAGG----A CTCGGCTTGC TGAA------ GTGCACACGG CAAGAGGCGA GAG-----CG GCGA---CTG --GTGAGTAC
F1.BE.93.VI850.AF077336          CCAGAGAAG- --ATCTCTCG ACGCGGGG-A CTCGGCTTGC TGAA------ GTGCAACACG GCAAGAGGCG AGAG----CG GCGA---CTG --GTGAGTAC
F1.BR.02.02BR082.FJ771006        CCAGAGGAG- --ATCTCTCG ACGCAGG--A CTCGGCTTGC TGAA------ GTGCACACGG CAAGAGGCGA GAG-----CG GCGA---CTG --GTGAGTAC
F1.BR.02.02BR170.FJ771007        CCAGAGAAG- --ATCTCTCG ACGCAGG--A CTCGGCTTGC TGAA------ GTGCACACGG CAAGAGGCGA GAG-----CG GCGA---CTG --GTGAGTAC
F1.BR.06.06BR564.FJ771008        CCAGAGCAG- --ATCTCTCG ACGCAGG--A CTCGGCTTGC TGAA------ GTGCACACGG CAAGAGGCGA GAG-----CG GCGA---CTG --GTGAGTAC
F1.BR.06.06BR579.FJ771009        CCAGAGAAG- --ATCTCTCG ACGCAGG--A CTCGGCTTGC TGAA------ GTGCACACGG CAAGAGGCGA GAG-----CG GCGG---CTG --GTGAGTAC
F1.BR.07.07BR844.FJ771010        CCAGAGAAG- --ATCTCTCG ACGCAGG--A CTCGGCTTGC TGAA------ GTGCACACAG CAAGAGGCGA GAG-----CG GCGA---CTG --GTGAGTAC
F1.BR.89.BZ126.AY173957          CCAGAGAAG- --ATCTCTCG ACGCAGG--A CTCGGCTTGC TGAA------ GTGCACACGG CAAGAGGCGA GAG-----GG GCGA---CTG --GTGAGTAC
F1.BR.93.93BR020_1.AF005494      CCAGAGAAG- --AACTCTCG ACGCAGG--A CTCGGCTTGC TGAA------ GTGCACACGG CAAGAGGCGA GAG-----CG GCGA---CTG --GTGAGTAC
F1.DE.x.MVP_30846.EU446022       CCGAGAATT- ---TCTCTCG ACGCAGG--A CTCGGCTTGC TGTA---AGT GCC---ACGG CAAGAGGCGA GAG-----CG GCGG---CTG --GTGAGTAC
F1.ES.02.ES_X845_4.FJ670516      CCAGAGAAG- --ATCTCTCG ACGCAGG--A CTCGGCTTGC TGAA------ GTGCACACGG CAAGAGGCGA GGGGAG--CG GCGA---CTG --GTGAGTAC
F1.ES.11.DEMF110ES001.JX140671   CCAGAGAAG- --ATCTCTCG ACGCAGG--A CTCGGCTTGC TGAA------ GTGCACACGG CAAGAGGCGA GGGG----CG GCGG---CTG --GTGAGTAC
F1.ES.x.P1146.DQ979023           ---------- ---------- ---------- CTCGGCTTGC TGAGTGC--- -----CACGG CAGAGGCGAG AG------CG GCGA---CTG --GTGAGTAC
F1.ES.x.X1093_2.DQ979025         CCAGAGAAG- --ATCTCTCG ACGCAGG--A CTCGGCTTGC TGAA------ GTGCGCACGG CAAGAGGCGA GGGGAG--CG GCGA---CTG --GTGAGTAC
F1.ES.x.X1670.DQ979024           CCAGAGAAG- --ATCTCTCG ACGCAGG--A CTCGGCTTGC TGAA------ GCGCGCACAG CAAGAGGCGA GAG-----CG GCGA---CTG --GTGAGTAC
F1.FI.93.FIN9363.AF075703        CCAGAGAAG- --ATCTCTCG ACGCAGG--A CTCGGCTTGC TGAA------ GTGCACACGG CAAGAGGCGA GAG-----CG GCGA---CTG --GTGAGTAC
F1.RO.96.BCI_R07.AB485658        CCAGAGAAG- --ATCTCTCG ACGCAGG--A CTCGGCTTGC TGAA------ GTGCACACGG CAAGAGGCGA GAG-----CG GCGA---CTG --GTGAGTAC
F1.RU.08.D88_845.GQ290462        CCAGAGGAG- --ATCTCTCG ACGCAGG--A CTCGGCTTGC TGAG------ GTGCACACAG CAAGAGGCGA GAG-----CG GCGG---CTG --GTGAGTAC
F2.CM.10.DEMF210CM007.JX140673   CCAGAGGAG- --ATCTCTCG ACGCAGG--A CTCGGCTTGC TGA------- GTGCACGCAG CAAGAGGCGA GAAG----CG GCGA---CTG --GTGAGTAC
G.BE.96.DRCBL.AF084936           CCAGAGAAG- --TTCTCTCG ACGCAGG--A CTCGGCTTGC TGAG------ GTGCACACAG CAAGAGGCGA GAG-----CG GCGA---CTG --GTGAGTAC
G.CM.01.A1786.FJ389367           CCAGAGAAG- --ATCTCTCG ACGCAGG--A CTCGGCTTGC TGAA------ GTGCACTCGG CAAGAGGCGA GAG-----CG GCGA---CTG --GTGAGTAC
G.CM.04.178_15.FJ389363          CCAGAGAAG- --TTCTCTCG ACGCAGG--A CTCGGCTTGC TGAG------ GTGCACACAG CAAGAGGCGA GAG-----CG GCGA---CTG --GTGAGTAC
G.CM.04.314_40.FJ389364          CCAGAGAAG- --ATCTCTCG ACGCAGG--A CTCGGCTTGC TGAG------ -TGCACACAG CAAGAGGCGA GGCGAGAGCG GCGA---CTG --GTGAGTAC
G.CM.04.515_28.FJ389365          CCAGAGAAG- --TTCTCTCG RCGCAGG--A CTCGGCTTGC TGAG------ GTGCACRCAG CAAGAGGCGA GAG-----CG GCGA---CTG --GTGAGTAC
G.CM.04.944_5.FJ389366           CCAGAGAAG- --TTCTCTCG ACGCAGG--A CTCGGCTTGC TGAA------ GTGCACACAG CAAGAGGCGA GAG-----CG GCGA---CTG --GTGAGTAC
G.CM.10.DEMG10CM008.JX140676     CCAGAGAAG- --TTCTCTCG ACGCAGG--A CTCGGCTTGC TAAA------ GTGCACACAG CAAGAGGCGA GAG-----CG GCGA---CTG --GTGAGTAC
G.CM.96.96CMABB55.AY772535       CCAGAGAAG- --TTCTCTCG ACGCAGG--A CTCGGCTTGC TGAG------ GTGCACACAG CAAGAGGCGA GAG-----CG GCGA---CTG --GTGAGTAC
G.CN.06.sh52.HM067749            CCCGAGAAG- --TTCTCTCG AGCCAGG--A CTCGGCTTGC TGAA------ GTGCACCAGC AAGAGGCGAG AG------CG GCGA---CTG --GTGAGTAC
G.CU.99.Cu74.AY586547            CCAGAGAAG- --TTCTCTCG ACGCAGG--A CTCGGCTTGC TGAG------ GTGCACACAG CAAGAGGCGA GAG-----CG GCGA---CTG --GTGAGTAC
G.CU.99.Cu85.AY586548            CCAGAGAAG- --TTCTCTCG ACGCAGG--A CTCGGCTTGC TGAG------ GTGCACACAG CAAGAGGCGA GAG-----CG GCGA---CTG --GTGAGTAC
G.CU.99.Cu87.AY586549            CCAGAGAAG- --TTCTCTCG ACGCAGG--A CTCGGCTTGC TGAG------ GTGCACACGG CAAGAGGCGA GAG-----CG GCGA---CTG --GTGAGTAC
G.ES.05.P962.EU786670            CCAGAGAAG- --TTCTCTCG ACGCAGG--A CTCGGCTTGC TGAA------ GTGCACACAG CAAGAGGCGA GAG-----CG GCGA---CTG --GTGAGTAC
G.ES.08.P1981_2.FJ670530         CCAGAGAAG- --TTCTCTCG ACGCAGG--A CTCGGCTTGC TGAA-----G GTGCACACAG CAAGAGGCGA GAG-----CG GCGG---CTG --GTGAGTAC
G.ES.09.X2634_2.GU362882         CCAGAGAAG- --TTCTCTCG ACGCAGG--A CTCGGCTTGC TGAA-----G GTGCACGCAG CAAGAGGCGA GAG-----CG GCGG---CTG --GTGAGTAC
G.ES.99.X138.AF450098            CCAGAGAAG- --TTCTCTCG ACGCAGG--A CTCGGCTTGC TGAA-----G GTGCRCACAG CAAGAGGCGA GAG-----CG GCGA---CTG --GTGAGTAC
G.GH.03.03GH175G.AB287004        CCAGAGAAG- --TTCTCTCG ACGCAGG--A CTCGGCTTGC TGAA------ GTGCACACAG CAAGAGGCGA GAG-----CG GCGA---CTG --GTGAGTAC
G.KE.93.HH8793_12_1.AF061641     CCAGAGAAG- --ATCTCTCG ACGCAGG--A CTCGGCTTGC TGAG------ GTGCACGCAG CAAGAGGCGA GAG-----CG GCGA---CTG --GTGAGTAC
G.NG.92.92NG083_JV10832.U88826   CCAGAGAAG- --TTCTCTCG ACGCAGG--A CTCGGCTTGC TGAA------ GTGCACACAG CAAGAGGCGA GAG-----CG GCGA---CTG --GTGAGTAC
G.PT.x.PT2695.AY612637           CCAGAGAAG- --TTCTCTCG ACGCAGG--A CTCGGCTTGC TGAA-----G GTGCACACAG CAAGAGGCGA GAG-----CG GCGG---CTG --GTGAGTAC
G.PT.x.PT3037.FR846408           CCAGAGAAG- --TTCTCTCG ACGCAGG--A CTCGGCTCGC TGAA-----G GTGCACTCGG CGAGAGGCGA GAG-----CG GCGA---CTG --GTGAGTAC
G.PT.x.PT3306.FR846409           CCAGAGAAG- --TTCTCTCG ACGCAGG--A CTCGGCTTGC TGAA-----G GTGCACACGG CAAGAGGCGA GAG-----CG GCGA---CTG --GTGAGTAC
G.PT.x.PT988.FR846410            CCAGAGAAG- --TTCTCTCG ACGCAGG--A CTCGGCTTGC TGAG------ GTGCACACAG CAAGAGGCGA GAG-----CG GCGG---CTG --GTGAGTAC
G.SE.93.SE6165_G6165.AF061642    CCAGAGAAG- --TTCTCTCG ACGCAGG--A CTCGGCTTGC TGAG------ GTGCACACAG CAAGAGGCGA GAG-----CG GCGA---CTG --GTGAGTAC
H.BE.93.VI991.AF190127           CCAGAGAAG- --CTCTCTCG ACGCAGG--A CTCGGCTTGC TGAA------ GTGCACACAG CAAGAGGCGA GGGG----CG GCGTGA-CTG --GTGAGTAC
H.BE.93.VI997.AF190128           ---------- ---TCTCTCG ACGCAGG--A CTCGGCTTGC TGAA------ GTGCACACGG CAAGAGGCGA GGGG----CG GCGCGA-CTG --GTGAGTAC
H.CF.90.056.AF005496             CCAGAGAAG- --ATCTCTCG ACGCAGG--A CTCGGCTTGC TGAA------ GTGCACACAG CAAGAGGCGA GAG-----CG GCGA---CTG --GTGAGTAC
H.GB.00.00GBAC4001.FJ711703      CCAGAGAAG- --TTCTCTCG ACGCAGG--A CTCGGCTTGC TGAG------ -TGCACACGG CAAGAGGCGA GGGG----CG GCGCGA-CTG --GTGAGTAC
J.CM.04.04CMU11421.GU237072      CCAGAGAAG- --TTCTCTCG ACGCAGG--A CTCGGCTTGC TGAA------ GTGCACACAG CAAGAGGCGA GAG-----CG GCGA---CTG --GTGAGTAC
J.SE.93.SE9280_7887.AF082394     ---------- --TTCTCTCG ACGCAGG--A CTCGGCTTGC TGAA------ GTGCACACGG CAAGAGGCGA GGGG----CG GCGA---CTG --GTGAGTAC
J.SE.94.SE9173_7022.AF082395     ---------- --TTCTCTCG ACGCAGG--A CTCGGCTTGC TGAA------ GTGCACACGG CAAGAGGCGA AGGG----CG GCGA---CTG --GTGAGTAC
01_AE.CF.90.90CF11697.AF197340   CCAGAGAAG- --TTCTCTCG ACGCAGG--A CTCGGCTTGC TGAG------ GTGCTCACAG CAAGAGGCGA GAG-----CG GCGA---CTG --GTGAGTAC
01_AE.CF.90.90CF4071.AF197341    CCAGAGAAG- --TTCCCTCG ACGCAGG--A CTCGGCTTGC TGAG------ GTGCACGCAG CAAGAGGCGA AGAG----CG GCGA---CTG --GTGAGTAC
01_AE.CF.90.90CR402_CAR_E_4002   CCAGAGAAG- --TTCTCTCG ACGCAGG--A CTCGGCTTGC TGAG------ GTGCACACAG CAAGAGGCGA GAG-----CG GCGA---CTG --GTGAGTAC
01_AE.CN.05.FJ051.DQ859178       CCAGAGAAG- --ATCTCTCG ACGCAGG--A CTCGGCTTGC TGAG------ -TGCACACAG CAAGAGGCGA GAG-----CG GCGA---CTG --GTGAGTAC
01_AE.CN.05.FJ053.DQ859179       CCAGAGAAG- --TTCTCTCG ACGCAGG--A CTCGGCTTGC TGAG------ GTGCACACAG CAAGAGGCGA GAG-----CG GCGA---CTG --GTGAGTAC
01_AE.CN.05.Fj052.EF036528       CCAGAGAAG- --TTCTCTCG ACGCAGG--A CTCGGCTTGC TGAG------ GTGCACACAG CAAGAGGCGA GAG-----CG GCGG---CTG --GTGAGTAC
01_AE.CN.05.Fj055.EF036527       CCAGAGAAG- --TTCTCTCG ACGCAGG--A CTCGGCTTGC TGAG------ GTGCACACAG CAAGAGGCGA GAG-----CG GCTA---CTG --GTGAGTAC
01_AE.CN.05.Fj056.EF036529       CCAGAGAAG- --TTCTCTCG ACGCAGG--A CTCGGCTTGC TGAG-----G TGCGCAACAG CAAGAGGCGA GAG-----CG GCTA---CTG --GTGAGTAC
01_AE.CN.05.Fj057.EF036530       CCAGAGAAG- --TTCTCTCG ACGCAGG--A CTCGGCTTGC TGAG-----G TGCGCAACAG CAAGAGGCGA GAG-----CA GCGG---CTG --GTGAGTAC
01_AE.CN.05.Fj065.EF036534       CCAGAGAAG- --TTCTCTCG ACGCAGG--A CTCGGCTTGC TGAG------ GTGCACACAG CAAGAGGCGA GAG-----CG GCGA---CTG --GTGAGTAC
01_AE.CN.05.Fj066.EF036535       CCAGAGAAG- --TTCTCTCG ACGCAGG--A CTCGGCTTGC TGAG------ GTGCACACAG CAAGAGGCGA GAG-----CG GCGG---AAG --GTGAGTAC
01_AE.CN.06.FJ054.DQ859180       CCAGAGAAG- --TTCTCTCG ACGCAGG--A CTCGGCTTGC TGAG------ GTGCACACAG CAAGAGGCGA GAG-----CG GCGA---CTG --GTGAGTAC
01_AE.CN.06.Fj062.EF036531       CCAGAGAAG- --TTCCCTCG ACGCAAG--A CTCGGCTTGC TGAG------ GTGCACACAG CAAGAGGCGA GAG-----CG GCGA---CTG --GTGAGTAC
01_AE.CN.06.Fj063.EF036532       CCAGAGAAG- --TTCTCTCG ACGCAGG--A CTCGGCTTGC TGAG------ GTGCACACAG CAAGAGGCGA GAG-----CG GCGA---CTG --GTGAGTAC
01_AE.CN.06.Fj064.EF036533       CCAGAGAAG- --TTCTCTCG ACGCTGG--A CTCGGCTTGC TGAG------ GTACACACAG CAAGAGGCGA GAG-----CG GCGAA--CTG --GTAAGTAC
01_AE.HK.04.HK001.DQ234790       CCAGAGAAG- --TTCTCTCG AGCGCAGG-A CTCGGCTTGC TGAG------ GTGCACACAG CAAGAGGCGA GAG-----CG GCGA---CTG --GTGAGTAC
01_AE.JP.x.DR0492.AB253423       CCAGAGAAG- --TTCTCTCG ACGCAGG--A CTCGGCTTGC TGAG------ GTGCACACAG CAAGAGGCGA GAG-----CG GCGA---CTG --GTGAGTAC
01_AE.JP.x.DR2594.AB253660       CCAGAGAAG- --TTCTCTCG ACGCAGG--A CTCGGCTTGC TGAG------ GTGCACACAG CAAGAGGCGA GAG-----CG GCGA---CTG --GTGAGTAC
01_AE.JP.x.DR6824.AB253426       CCAGAGAAG- --TTCTCTCG ACGCAGG--A CTCGGCTTGC TGAG------ GTGCACACAG CAAGAGGCGA GAG-----CG GCGA---CTG --GTGAGTAC
01_AE.JP.x.JRC77AE.AB565504      CCAGAGAAG- --TTCTCTCG ACGCAGG--A CTCGGCTTGC TGAG------ GTGCACACAG CAAGAGGCGA GAG-----CG GCGA---CTG --GTGAGTAC
01_AE.TH.04.AA027a_wg4.JX44702   CCAGAGAAG- --TTCTCTCG ACGCAGG--A CTCGGCTTGC TGAG------ GTGCACACAG CAAGAGGCGA GAG-----CG GCGA---CTG --GTGAGTAC
01_AE.TH.04.AA075a_WG7.JX44754   CCAGAGAAG- --TTCTCTTG ACGCAGG--A CTCGGCTTGC TGAG------ GTGCACACAG CAAGAGGCGA GAG-----CG GCGA---CTG --GTGAGTAC
01_AE.TH.04.BKM.DQ314732         CCAGAGAAG- --TTCTCTCG ACGCAGG--A CTCGGCTTGC TGCG------ GTGCACACAG CAAGCGGCGA GAG-----CG GCGA---CTG --GTGAGTAC
01_AE.TH.05.AA004a_wg4a.JX4467   CCAGAGAAG- --TTCTCTCG ACGCAGG--A CTCGGCTTGC TGAG------ GTGCACACAG CAAGAGGCGA GAG-----CG GCGA---CTG --GTGAGTAC
01_AE.TH.05.AA033a_wg6a.JX4470   CCAGAGAAG- --TTCTCTCG ACGCAGG--A CTCGGCTTGC TGAA-----G GTGCACGCAG CAAGAGGCGA GAAG----CG GCGA---CTG --GTGAGTAC
01_AE.TH.05.AA049a_WG13.JX4472   CCAGAGAAG- --TTCTCTCG ACGCAGG--A CTCGGCTTGC TGAG------ GTGCACACAG CAAGAGGCGA GAG-----CG GCGA---CTG --GTGAGTAC
01_AE.TH.05.AA064a_WG2.JX44741   CCAGAGAAG- --TTCTCTCG ACGCAGG--A CTCGGCTTGC TGAG------ GTGCACACAG CAAGAGGCGA GAG-----CG GCGA---CTG --GTGAGTAC
01_AE.TH.05.AA079a_WG4.JX44759   CCAGAGAAG- --ATCTCTCG ACGCAGG--A CTCGGCTTGC TGAG------ GTGCACACAG CAAGAGGCGA GAG-----CG GCGA---CTG --GTGAGTAC
01_AE.TH.05.AA101a_WG1.JX44793   CCAGAGAAG- --TTCTCTCG ACGCAGG--A CTCGGCTTGC TGAG------ GTGCACACAG CAAGAGGCGA GAG-----CG GCGA---ACG --GTGAGTAC
```

```
01_AE.TH.05.AA107a_wg4.JX44802    CCAGAGAAG- --TTCTCTCG ACGCAGG--A CTCGGCTTGC TGAG------ GTGCACACAG CAAGAGGCGA GAG-----CG GCGA---CTG --GTGAGTAC
01_AE.TH.06.AA002a_WG1.JX44666    CCAGAGAAG- --TTCTCTCG ACGCAGG--A CTCGGCTTGC TGAG------ GTGCACACAG CAAGAGGCGA GAG-----AG GCGA---CTG --GTGAGTAC
01_AE.TH.06.AA017a_wg1.JX44689    CCAGAGAAG- --TTCTCTCG ACGCAGG--A CTCGGCTTGC TGAG------ GTGCACACAG CAAGAGGCGA GAG-----CG GCGA---CCG --GTGAGTAC
01_AE.TH.06.AA034a_wg2.JX44708    CCAGAGAAG- --TTCTCTCG ACGCAGG--A CTCGGCTTGC TGAG------ GTGCACACAG CAAGAGGCGA GAG-----CG GCGA---CTG --GTGAGTAC
01_AE.TH.06.AA038a_WG3.JX44713    CCAGAGAAG- --TTCTCTCG ACGCAGG--A CTCGGCTTGC TGAG------ GTGCACACAG CAAGAGGCGA GAG-----CG GCGA---CTG --GTGAGTAC
01_AE.TH.06.AA055a_WG4.JX44731    CCAGAGAAG- --TTCTCTCG ACGCAGG--A CTCGGCTTGC TGG------- GTGCACACGG CAAGAGGCGA GAG-----CG GCGA---CTG --GTGAGTAC
01_AE.TH.06.AA056a_WG5.JX44731    CCAGAGAAG- --TTCTCTCG ACGCAGG--A CTCGGCTTGC TGAG------ GTGCACACAG CAAGAGGCGA GAG-----CG GCGA---CTG --GTGAGTAC
01_AE.TH.06.AA059a_WG5.JX44735    CCAGAGAAG- --TTCTCTCG ACGCAGG--A CTCGGCTTGC TGAG------ GTGCACACAG CAAGAGGCGA GAG-----CG GCGAA--CTG --GTGAGTAC
01_AE.TH.06.AA063a_WG37.JX4474    CCAGAGAAG- --TTCTCTCG ACGCAGG--A CTCGGCTTGC TGAG------ GTGCACACAG CAAGAGGCGA GAG-----CG GCGA---CTG --GTGAGTAC
01_AE.TH.06.AA068a_14.JX447465    CCAGAGAAG- --TTCTCTCG ACGCAGG--A CTCGGCTTGC TGAG------ GTGCACACAG CAAGAGGCGA GAG-----CG GCGA---CTG --GTGAGTAC
01_AE.TH.06.AA082a_WG9.JX44764    CCAGAGAAG- --TTCTCTCG ACGCAGG--A CTCGGCTTGC TGCG------ GTGCACACAG CAAGAGGCGA GAG-----CG GCGA---CTG --GTGAGTAC
01_AE.TH.06.AA085a_wg2.JX44768    CCAGAGAAG- --TTCTCTCG ACGCAGG--A CTCGGCTTGC TGAG------ GTGCACACAG CAAGAGGCGA GCG-----CG GCGA---CTG --GTGAGTAC
01_AE.TH.06.AA088a_wg14.JX4477    CCAGAGAAG- --ATCTCTCG ACGCAGG--A CTCGGCTTGC TGAG------ GTGCACACAG CAAGAGGCGA GAG-----CG GCGA---CTG --GTGAGTAC
01_AE.TH.06.AA099a_WG9.JX44789    CCAGAGAAG- --TTCTCTCG ACGCAAGG-A CTCGGCTTGC TGAG------ GTGCACACAG CAAGAGGCGA GAG-----CG GCGA---CTA -GGTGAGTAC
01_AE.TH.07.AA015a_WG4.JX44687    CCAGAGAAG- --TTCTCTCG ACGCAGG--A CTCGGCTTGC TGAG------ GTGCACACAG CAAGAGGCGA GAG-----CG GCGA---CTG --GTGAGTAC
01_AE.TH.07.AA019a_WG11.JX4469    CCAGAGAAG- --TTCTCTCG ACGCAGG--A CTCGGCTTGC TGAG------ GTGCACACAG CAAGAGGCGA GAG-----CG GCGA---CTG --GTGAGTAC
01_AE.TH.07.AA028a_wg3.JX44702    CCAGAGAAG- --TTCTCTCG ACGCAGG--A CTCGGCTTGC TGAG------ GTGCACACAG CAAGAGGCGA GAG-----CG GCGA---CTG --GTGAGTAC
01_AE.TH.07.AA050a_WG7.JX44728    CCAGAGAAG- --TTCTCTCG ACGCAGG--A CTCGGCTTGC TGAG------ GTGCACACAG CAAGAGGCGA GAG-----CG GCGA---CTG --GTGAGTAC
01_AE.TH.08.AA007a_WG10.JX4467    CCAGAGAAG- --TTCTCTCG ACGCAGG--A CTCGGCTTGC TGAG------ GTGCACACAG CAAGAGGCGA GAG-----CG GCGA---CTG --GTGAGTAC
01_AE.TH.08.AA037a_WG6.JX44712    CCAGAGAAG- --TTCTCTCG ACGCAGG--A CTCGGCTTGC TGAG------ GTGCACACAG CAAGAGGCGA GAG-----CG GCGA---CTG --GTGAGTAC
01_AE.TH.08.AA060a_WG1.JX44735    CCAGAGAAG- --TTCTCTCG ACGCAGG--A CTCGGCTTGC TGAA------ GTGCACACAG CAAGAGGCGA GAG-----CG GCGA---CTG --GTGAGTAC
01_AE.TH.08.AA067a_WG12.JX4474    CCAGAGAAG- --TTCTCTCG ACGCAGG--A CTCGGCTTGC TGAG------ GTGCACACAG CAAGAGGCGA GAG-----CG GCGA---CTG --GTGAGTAC
01_AE.TH.08.AA108a_WG6.JX44802    CCAGAGAAG- --TTCTCTCG ACGCAGG--A CTCGGCTTGC TGAG------ GTGCACACAG CAAGAGGCGA GAG-----CG GCGA---CTG --GTGAGTAC
01_AE.TH.09.AA090a_WG11.JX4477    CCAGAGAAG- --TTCTCTCG ACGCAGG--A CTCGGCTTGC TGAG------ GTGCACACAG CAAGAGGCGA GAG-----CG GCGA---CTG --GTGAGTAC
01_AE.TH.09.AA111a_WG11.JX4480    CCAGAGAAG- --TTCTCTCG ACGCAGG--A CTCGGCTTGC TGAG------ GTGCACACAG CAAGAGGCGA GAG-----CG GCGA---CTG --GTGAGTAC
01_AE.TH.90.CM240.U54771          CCAGAGAAG- --TTCTCTCG ACGCAGG--A CTCGGCTTGC TGAG------ GTGCACACAG CAAGAGGCGA GAG-----CG GCGA---CTG --GTGAGTAC
01_AE.TH.93.93TH253.U51189        CCAGAGAAG- --TTCTCTCG ACGCAGG--A CTCGGCTTGC TGAG------ GTGCACACAG CAAGAGGCGA GAG-----CG GCGA---CTG --GTGAGTAC
01_AE.TH.93.93TH9021.AF164485     CCAGAGAAG- --TTCTCTCG ACGCAGG--A CTCGGCTTGC TGCG------ GTGCACACAG CAAGAGGCGA GAC-----GG GCGA---CTG --GTGAGTAC
01_AE.TH.95.95TNIH022.AB032740    CCAGAGAAG- --TTCTCTCG ACGCAGG--A CTCGGCTTGC TGAG------ GTGCACACAG CAAGAGGCGA GAG-----CG GCGA---CTG --GTGAGTAC
01_AE.TH.95.95TNIH047.AB032741    CCAGAGAAG- --TTCTCTCG ACGCAGG--A CTCGGCTTGC TGAG------ GTGCACACAG CAAGAGGCGA GAG-----CG GCGA---CTG --GTGAGTAC
01_AE.TH.x.NP03.AB485654          CCAGAGAAG- --TTCTCTCG ACGCAGG--A CTCGGCTTGC TGAG------ GTGCACACAG CAAGAGGCGA GGAG----CG GCGA---CTG --GTGAGTAC
01_AE.VN.97.97VNAG204.FJ185247    ---------- ---------- ---------- ---------- ---------- ---------- ---------- --------CG GCGA---CTG --GTGAGTAC
01_AE.VN.97.97VNAG206.FJ185248    ---------- ---------- ---------- ---------- ---------- ---------- ---------- --------CG GCGA---CTG --GTGAGTAC
01_AE.VN.97.97VNAG207.FJ185249    ---------- ---------- ---------- ---------- ---------- ---------- ---------- --------CG GCGA---CTG --GTGAGTAC
01_AE.VN.97.97VNAG210.FJ185251    ---------- ---------- ---------- ---------- ---------- ---------- ---------- --------CG GCGA---CTG --GTGAGTAC
01_AE.VN.97.97VNAG212.FJ185252    ---------- ---------- ---------- ---------- ---------- ---------- ---------- --------CG GCGA---CTG --GTGAGTAC
01_AE.VN.97.97VNAG214.FJ185253    ---------- ---------- ---------- ---------- ---------- ---------- ---------- --------CG GCGA---CTG --GTGAGTAC
01_AE.VN.97.97VNAG218.FJ185255    ---------- ---------- ---------- ---------- ---------- ---------- ---------- --------CG GCGA---CTG --GTGAGTAC
01_AE.VN.97.97VNHCM314.FJ18524    ---------- ---------- ---------- ---------- ---------- ---------- ---------- --------CG GCGA---CTG --GTGAGTAC
01_AE.VN.97.97VNHCM319.FJ18524    ---------- ---------- ---------- ---------- ---------- ---------- ---------- --------CG GCGA---CTG --GTGAGTAC
01_AE.VN.97.97VNHCM343.FJ18524    ---------- ---------- ---------- ---------- ---------- ---------- ---------- --------CG GCGA---CTG --GTGAGTAC
01_AE.VN.97.97VNHCM345.FJ18524    ---------- ---------- ---------- ---------- ---------- ---------- ---------- --------CG GCGA---CTG --GTGAGTAC
01_AE.VN.98.98VNND15.FJ185235     ---------- ---------- ---------- ---------- ---------- ---------- ---------- --------CG GCGA---CTG --GTGAGTAC
02_AG.CM.08.DE00208CM001.JX140    CCAGAGAAG- --ATCTCTCG ACGCAGGG-A CTCGGCTTGC TGAG------ GTGCACACGG CAAGAGGCGA GAG-----CG GCGA---CTG --GTGAGTAC
02_AG.CM.08.DE00208CM004.JX140    CCAGAGAAG- --TTCTCTCG ACGCAAGG-A CTCGGCTTGC TGAG------ GTGCACACAG CAAGAGGCGA GAG-----CG GCGA---CTG --GTGAGTAC
02_AG.CM.99.pBD6_15.AY271690      CCAGAGAAG- --ATCTCTCG ACGCACGG-A CTCGGCTTGC TGAG------ GTGCACACAG CAAGAGGCGA GAG-----CG GCGA---CTG --GTGAGTAC
02_AG.ES.06.P1261.EU786671        CCAGAGAAG- --CTCTCTCG ACGCAGGG-A CTCGGCTTGC TGAG------ GTGCACACAG CAAGAGGCGA GAG-----CG GCGA---CTG --GTGAGTAC
02_AG.ES.06.P1423.EU884501        CCAGAGAAG- --ATCTCTCG ACGCAAGG-G CTCGGCTTGC TGAG------ GTGCACACAG CAAGAGGCGA GAG-----CG GCGA---CTG --GTGAGTAC
02_AG.FR.91.DJ263.AF063223        CCAGAGAAG- --ATCTCTCG ACGCAGGG-A CTCGGCTTGC TGAG------ GTGCACGCAG CAAGAGGCGA GAG-----CG GCGA---CTG --GTGAGTAC
02_AG.GH.03.03GH181AG.AB286855    CCAGAGAAG- --TTCTCTCG ACGCAAGG-A CTCGGCTTGC TGAG------ GTGCACGCAG CAAGAGGCGA GAG-----CG GCGA---CTG --GTGAGTAC
02_AG.GH.03.03GH182AG.AB286857    CCAGAGAAG- --TTCTCTCG ACGCACGG-A CTCGGCTTGC TGAG------ GTGCACACAG CAAGAGGCGA GAG-----CG GCGA---CTG --GTGAGTAC
02_AG.GH.03.03GH189AG.AB286862    CCAGAGAAG- --CTCTCTCG ACGCAGGG-A CTCGGCTTGC TGAG------ GTGCACACAG CAAGAGGCGA GAG-----CG GCGA---CTG --GTGAGTAC
02_AG.GH.03.03GH197AG.AB286863    CCAGAGAAG- --ATCTCTCG ACGCAGGG-A CTCGGCTTGC TGAA------ GTGCACACAG CAAGAGGCGA GAG-----CG GCGA---CTG --GTGAGTAC
02_AG.GH.03.GHNJ185.AB231895      CCAGAGAAG- --CTCTCTCG ACGCAGGG-A CTCGGCTTGC TGAG------ GTGCACACGG CAAGAGGCGA GAG-----CG GCGA---CTG --GTGAGTAC
02_AG.GH.03.GHNJ188.AB231896      CCAGAGAAG- --ATCTCTCG ACGCACGG-A CTCGGCTTGC TGAG------ GTGCACACAG CAAGAGGCGA GAG-----CG GCGA---CTG --GTGAGTAC
02_AG.GH.03.GHNJ196.AB231898      CCAGAGAAG- ---TATCTCG ACGCACGG-A CTCGGCTTGC TGAG------ GTGCACACAG CAAGAGGCGA GAG-----CG GCGA---CTG --GTGAGTAC
02_AG.GH.97.97GH_AG1.AB049811     CCAGAGAAG- --TTCTCTCG ACGCAAGG-A CTCGGCTTGC TGAG------ GTGCACACAG CAAGAGGCGA GAG-----CG GCGA---CTG --GTGAGTAC
02_AG.GH.x.I_2496.AB485633        CCAGAGAAG- --CTCTCTCG ACGCAGGG-A CTCGGCTTGC TGAG------ GTGCACACAG CAAGAGGCGA GAG-----CG GCGA---CTG --GTGAGTAC
02_AG.GW.04.CC_0030.FJ694791      CCAGAGAAG- --CTCTCTCG ACGCAAGG-W CTCGGCTTGC TGA------- GTGCACACAS CAAGAGGCGG GAG-----CG GCGA---CTG --GTGAGTAC
02_AG.GW.05.CC_0048.FJ694792      CCAGAGAAG- --WTCTCTCG ACGCACGG-A CTYGGCTTGC TGAG------ GTGCACACAG CAAGAGGCGA GAG-----CG GCGA---CTG --GTGAGTAC
02_AG.KR.07.07MHI10.JQ316136      CCAGAGAAG- --CTCTCTTG ACGCAGAG-A CTCGGCTTGC TGAG------ GTGCACACAG CAAGAGGCGA GAG-----CG GCGA---CTG --GTGAGTAC
02_AG.LR.x.POC44951.AB485636      CCAGGGA-G- --CTCTCTCG ACGCAGAG-A CTCGGCTTGC TGAG------ GTGCACGCAG CAAGAGGCGA GAG-----CG GCGA---CTG --GTGAGTAC
02_AG.NG.x.IBNG.L39106            CCAGAGAAG- --ATCTCTCG ACGCAAGG-A CTCGGCTTGC TGAG------ GTGCGCACAG CAAGAGGCGA GAG-----CG GCGA---CTG --GTGAGTAC
02_AG.SE.94.SE7812.AF107770       CCAGAGAAG- --ATCTCTCG ACGCAGGG-A CTCGGCTTGC TGAG------ GTGCACACAG CAAGAGGCGA GAG-----CG GCGA---CTG --GTGAGTAC
03_AB.BY.00.98BY10443.AF414006    CCAGAGAAG- --TTCTCTCG ACGCAGG--A CTCGGCTTGC TGAG------ GTGCACACAG CAAGAGGCGA GAG-----CG GTGA---CTG --GTGAGTAC
03_AB.RU.98.RU98001_98RU001.AF    CCAGAGAAG- --ATCTCTCG ACGCAGG--A CTCGGCTTGC TGAG------ GTGCACACAG CAAGAGGCGA GAG-----CG GCGA---CTG --GTGAGTAC
04_cpx.CY.94.94CY032_3.AF04933    CCAGAGAAG- --TTCTCTCG ACGCAGG--A CTCGGCTTGC TGAG------ GTGCACACAG CAAGAGGCGA GAG-----CG GCGA---CTG --GTGAGTAC
04_cpx.GR.00.DE00400GR002.JX14    CCAGAGAAG- --TTCTCTCG ACGCAGG--A CTCGGCTTGC TGAA------ GTGCGCACAG CAAGAGGCGA GAG-----CG GCGA---CTG --GTGAGTRC
04_cpx.GR.91.GR11_97PVCH.AF119    CCAGAGAAG- --TTCTCTCG ACGCAGG--A CTCGGCTTGC TGAG------ GTGCACACAG CAAGAGGCGA GAG-----CG GCGA---CTG --GTGAGTAC
04_cpx.GR.97.GR84_97PVMY.AF119    CCAGAGAAG- --TTCTCTCG ACGCAGG--A CTCGGCTTGC TGAG------ GTGCACACAG CAAGAGGCGA GAG-----CG GCGA---CTG --GTGAGTAC
05_DF.BE.93.VI961.AF076998        CCAGAGAAG- --AACTCTCG ACGCAGG--A CTCGGCTTGC TGAA------ GCGCGCACGG CAAGAGGCGA GGGG----TA GCGA---CTG --GTGAGTAC
05_DF.BE.x.VI1310.AF193253        CCAGAGAAG- --AACTCTCG ACGCAGG--A CTCGGCTTGC TGAA------ GCGCGCAAGG CAAGAGGCGA GGGG----TA GCGA---CTG --GTGAGTAC
05_DF.ES.99.X492.AY227107         CCAGAGAAG- ---ACTCTCG ACGCAGG--A CTCGGCTTGC TGAA------ GCGCGCACGG CAAGAGGCGA GGGG----TA GCGA---CTG --GTGAGTAC
06_cpx.AU.96.BFP90.AF064699       CCAGAGAAG- --TTCTCTCG ACGCAGG--A CTCGGCTTGC TGAG------ GTGCACACAG CAAGAGGCGA GAG-----CG GCGA---CTG --GTGAGTAC
06_cpx.CD.x.BCF_Dioum.AB485660    CCAGAGAAG- --TTCTCTCG ACGCAGG--A CTCGGCTTGC TGAA------ GTGCACGCAG CAAGAGGCGA GAG-----CG GCGA---CTG --GTGAGTAC
06_cpx.EE.01.EE0359.AY535659      CCAGAGAAG- --TTCTCTCG ACGCAGG--A CTCGGCTTGC TGAA------ GTGCACACGG CAAGAGGCGA GAG-----CG GCAA---CTG --GTGAGTAC
06_cpx.GH.03.03GH173_06.AB2868    CCAGAGAAG- --TTCTCTCG ACGCAGG--A CTCGGCTTGC TGAG------ GTGCACACAG CAAGAGGCGA GAG-----CG GCGA---CTG --GTGAGTAC
06_cpx.ML.95.95ML127.AJ288982     CCAGAGAAG- --TTCTCTCG ACGCAGG--A CTCGGCTTGC TGAG------ GTGCACACAG CAAGAGGCGA GAG-----CG GCGA---CTG --GTGAGTAC
06_cpx.ML.95.95ML84.AJ245481      CCAGAGAAG- --TTCTCTCG ACGCAGG--A CTCGGCTTGC TGAG------ GTGCACACAG CAAGAGGCGA GAG-----CG GCGA---CTG --GTGAGTAC
06_cpx.SN.97.97SE1078.AJ288981    CCAGAGAAG- --TTCTCTCG ACGCAGG--A CTCGGCTTGC TGAG------ GTGCACACGG CAAGAGGCGA GGGG----CG GCGA---CCG --GTGAGTAC
07_BC.CN.05.XJDC6431_2.EF36837    CCAGAGGAG- --ATCTCTCG ACGCAGG--A CTCGGCTTGC TGAA------ GTGCACTCGG CAAGAGGCGA GAG-----CG GCGA---CTG --GTGAGTAC
07_BC.CN.05.XJDC6441.EF368370     CCAGAGGAG- --ATCTCTCA ACGCAGG--A CTCGGCTTGC TGAA------ GTGCACTCGG CAAGAGGCGA GAG-----CG GCGA---CTG --GTGAGTAC
07_BC.CN.06.Sichuan_2006_SC006    CCAGAGGAG- --ATCTCTCG ACGCAGG--A CTCGGCTTGC TGAA------ GTGCACTCGG CAAGAGGCGA GAG-----CG GCGA---CTG --GTGAGTAC
07_BC.CN.06.Sichuan_2006_SC008    CCAGAGAAG- --ATCTCTCG ACGCAGG--A CTCGGCTTGC TGAA------ GTGCACTCGG CAAGAGGCGA GAG-----CG GCGA---CTG --GTGAGTAC
07_BC.CN.06.Xinjiang_2006_709.    CCAGAGGAG- --ATCTCTCG ACGCAGG--A CTCGGCTTGC TGAA------ GCGCGCTCGG C-AGAGGCGA GAG-----CG GCGG---CTG --GTGAGTAC
07_BC.CN.07.CNGZD.JQ423923        CCAGAGGAG- --ATCTCTCG ACGCAGG--A CTCGGCTTGC TGAA------ GTGYACTCGG CAAGAGGCGA GAG-----CG GCGG---CTG --GTGAGTAC
07_BC.CN.98.98CN009.AF286230      CCAGAGGAG- --ATCTCTCG ACGCAGG--A CTCGGCTTGC TGAA------ GTGCACTCGG CAAGAGGCGA GAG-----CG GCGA---CTG --GTGAGTAC
08_BC.CN.00.p00CH_HH090_08_BC3    CCAGAGGAG- --ATCTCTCG ACGCAGG--A CTCGGCTTGC TGAA------ GTGCACTCGG CAAGAGGCGA GAG-----CG GCGA---CTG --GTGAGTAC
08_BC.CN.00.p00CH_WS035_08_BC5    CCAGAGGAG- --ATCTCTCG ACGCAGG--A CTCGGCTTGC TGAA------ GTGCACTCAG CAAGAGGCGA GAG-----CG GCGA---CTG --GTGAGTAC
08_BC.CN.01.p01CH_DL001_08_BC0    CCAGAGGAG- --ATCTCTCG ACGCAGG--A CTCGGCTTGC TGAA------ GTGCACTCGG CAAGAGGCGA GAG-----GG GCGA---CTG --GTGAGTAC
08_BC.CN.06.nx2.HM067748          CCCGAGGGG- --ATTTCTTG ACGCAGG--A CTCGGCTTGC TGAA------ GTGCACTCGG CAAGAGGCGA GAG-----GG GCGA---CTG --GTGAGTAC
08_BC.CN.98.98CN006.AF286229      CCAGAGGAG- --ATCTCTCG ACGCAGG--A CTCGGCTTGC TGAA------ GTGCACTCGG CAAGAGGCGA GAG-----CG GCGA---CTG --GTGAGTAC
09_cpx.CI.00.00IC_10092.AJ8665    CCAGAGAAG- --TTCTCTCG ACGCAGG--A CTCGGCTTGC TGAG------ GTGCACGCAG CAAGAGGCGA GAG-----CG GCGA---CTG --GTGAGTAC
10_CD.TZ.96.96TZ_BF061.AF28954    CCAGAGGAG- --ATCTCTCG ACGCAGG--A CTCGGCTTGC TGAA------ GCGCGCACAG CAAGAGGCGA GGGG----CA GCGA---ACG --GTGAGTAC
10_CD.TZ.96.96TZ_BF071.AF28954    CCAGAGGAG- --ATCTCTCG ACGCAGG--A CTCGGCTTGC TGAA------ GCGCGCACAG CAAGAGGCGA GGGG----CA GCGA---ACG --GTGAGTAC
10_CD.TZ.96.96TZ_BF110.AF28955    CCAGAGGAG- --CTCTCTCG ACGCAGG--A CTCGGCTTGC TGAA------ GCGCGCACAG CAAGAGGCGA GGGG----CA GCGA---ACG --GTGAGTAC
11_cpx.CM.95.95CM_1816.AF49262    CCAGAGAAG- --TTCTCTCG ACGCAGG--A CTCGGCTTGC TGAG------ GTGCACGCAG CAAGAGGCGA GAG-----CG GCGA---CTG --GTGAGTAC
11_cpx.CM.96.96CM_4496.AF49262    CCAGAGAAG- --TTCTCTNG ACGCAGG--A CTCGGCTTGT TGAG------ GTGCACACAG CAAGAGGCGA GAG-----CG GCGA---TTG --GTGAGTAC
11_cpx.CM.97.MP818.AJ291718       ACCCGAGAAG --TTCTCTCG ACGCAGGA-A CTCGGCTTGC TGAG------ GTGCACACAG CAAGAGGCGA AAG-----CG GCGAA--CTG --GTGAGTAC
11_cpx.FR.99.MP1298.AJ291719      CCAGAGAAAG --TTCTCTCG ACGCAGG--A CTCGGCTTGC TGAG------ GTGCACACAG CAAGAGGCGA GAG-----CG GCGG---CTG --GTGAGTAC
11_cpx.FR.99.MP1307.AJ291720      CCAGAGAAG- --TTCTCTCG ACGCAGG--A CTCGGCTTGC TGAA-----A GTGCACGCAG CAAGAGGCGA GAG-----CG GCGA---CTG --GTGAGTAC
11_cpx.GR.x.GR17.AF179368         ---------- ------CTTG ACGCAGG--A CTCGGCTTGC TGAG------ GTGCACGCAG CAAGAGGCGA GAG-----CG GCGG---CTG --GTGAGTAC
12_BF.AR.97.A32879.AF408629       CCAGAGAAG- --ATCTCTCG ACGCAGG--A CTCGGCTTGC TGAA------ GCGCGCACGG CAAGAGGCGA GGGG----CG GCGA---CTG --GTGAGTAC
12_BF.AR.97.A32989.AF408630       CCAGAGAAG- --ATCTCTCG ACGCAGG--A CTCGGCTTGC TGCA------ GCGCGCACGG CAAGAGGCGA GGGG----CG GCGA---CCG --GTGAGTAC
12_BF.AR.99.ARMA159.AF385936      CCAGAGGAG- --ATCTCTCG ACGCAGG--A CTCGGCTTGC TGAA------ GCGCGCACGG CAAGAGGCGA GAGG----CG GCGA---CTG --GTGAGTAC
```

```
12_BF.UY.99.URTR23.AF385934      CCAGAGAAG- --ATCTCTCG ACGCAGG--A CTCGGCTTGC TGAA------ --GCGCACGG CAAGAGGCGA GGGG----CG GCGA---CTG --GTGAGTAC
12_BF.UY.99.URTR35.AF385935      CCAGAGAAG- --ATCTCTCG ACGCAGG--A CTCGGCTTGC TGAG------ --GCGCACGG CAAGAGGCGA GGAG----CG GCGA---CTG --GTGAGTAC
13_cpx.CM.02.02CM_A1394.DQ8453   AGCGACGCAG A--------- -----GG--A CTCGGCTTGC TGAG------ -CGCGCACAG CAAGAGGCGA GGGG----CG GCGA---CTG --GTGAGTAC
13_cpx.CM.04.04CM_173_9.DQ8453   CCAGAGAAG- --TTCTCTCG ACGCAGG--A CTCGGCTTGC TGAG------ -CGCGCACAG CAAGAGGCGA GGGG----CG GCGG---CTG --GTGAGTAC
13_cpx.CM.04.04CM_632_28.DQ845   CCAGAGAAG- --TTCTCTCG ACGCAGG--A CTCGGCTTGC TGAG------ -CGCGCACAG CAAGAGGCGA GGGG----CG GCGG---CTG --GTGAGTAC
13_cpx.CM.96.96CM_1849.AF46097   CCAGAGAAG- --TTCTCTCG ACGCAGG--A CTCGGCTTGC TGAG------ -CGCGCACAG CAAGAGGCGA GGGG----CG GCGG---CTG --GTGAGTAC
13_cpx.CM.96.96CM_4164.AF46097   CCAGAGAAG- --TTCTCTCG ACGCAGG--A CTCGGCTTGC TGAG------ -CGCGCACAG CAAGAGGCGA GGGG----CG GCGG---CTG --GTGAGTAC
14_BG.ES.00.X605.AF450096        CCAGAGAAG- --TTCTCTCG ACGCAGG--A CTCGGCTTGC TGAA-----G GTGCACACAG CAAGAGGCGA GAG-----CG G------CTG --GTGAGTAC
14_BG.ES.00.X623.AF450097        CCAGAGAAG- --TTCTCTCG ACGCAGG--A CTCGGCTTGC TGAA-----G GTGCACACAG CAAGAGGCGA GAG-----CG GCGA---CTG --GTGAGTAC
14_BG.ES.05.X1870.FJ670522       CCAGAGAAG- --TTCTCTCG ACGCAGG--A CTCGGCTTGC TGAAAG---- GTGCACACGG CAAGAGGCGA GAG-----CG G------CGG --GTAAGTAC
14_BG.ES.05.X772_8.FJ670528      CCAGAGAAG- --TTCTCTCG ACGCAGG--A CTCGGCTTGC TGAA-----G GTGCACACAG CAAGAGGCGA GAG-----CG GCGG---CTG --GTGAGTAC
15_01B.TH.99.99TH_MU2079.AF516   CCAGAGAAG- --TTCTCTCG ACGCAGG--A CTCGGCTTGC TGAG------ GTGCACACAG CAAGAGGCGA GAG-----CG GCGA---CTG --GTGAGTAC
15_01B.TH.99.99TH_R2399.AF5305   CCAGAGAAG- --TTCTCTCG ACGCAGG--A CTCGGCTTGC TGAG------ GTGCACACAG CAAGAGGCGA GAG-----CG GCGG---CTG --GTGAGTAC
16_A2D.KR.97.97KR004.AF286239    CCAGAGAAG- --ATCTCTCG ACGCAGG--A CTCGGCTTGC TGAA------ GCGCACG--G CAAGAGGCGA GGGG----CG GCGA---CTG --GTGAGTAC
18_cpx.CU.99.CU14.AY586541       ---------- ----CTCTCG ACGCAGG--A CTCGGCTTGC TGAG------ GTGCACACAG CAAGAGGCGA GAG-----CG GCGA---CTG --GTGAGTAC
18_cpx.CU.99.CU68.AY894993       ---------- ----CTCTCG ACGCAGG--A CTCGGCTTGC TGAG------ GTGCACGCAG CAAGAGGCGA GAG-----CG GCGA---CTG --GTGAGTAC
18_cpx.CU.99.CU76.AY586540       ---------- ----CTCTCG ACGCAGG--A CTCGGCTTGC TGAG------ GTGCACACAG CAAGAGGCGA GAG-----CG GCGA---CTG --GTGAGTAC
19_cpx.CU.99.CU29.AY588971       CCAGAAAAG- --TTCTCTCG ACGCAGG--A CTCGGCTTGC TGAG------ GTGCACACAG CAAGAGGCGA GAG-----CG GCGA---CTG --GTGAGTAC
20_BG.CU.03.CB134.DQ020274       CCAGAGAAG- --TTCTCTCG ACGCAGG--A CTCGGCTTGC TGAG------ GTGCACACAG CAAGAGGCGA GAG-----CG GCGA---CTG --GTGAGTAC
20_BG.CU.99.Cu103.AY586545       CCAGAGAAG- --TTCTCTCG ACGCAGG--A CTCGGCTTGC TGAG------ GTGCACACAG CAAGAGGCGA GAG-----CG GCGA---CTG --GTGAGTAC
20_BG.ES.99.R77.AY586544         CCAGAGAAG- --TTCTCTCG ACGCAGG--A CTCGGCTTGC TGAG------ GTGCACACAG CAAGAGGCGA GAG-----CG GCGA---CTG --GTGAGTAC
22_01A1.CM.02.02CAMLT72.EU7439   ---------- ---------- ---------- ------TTGC TGAA------ GCGCGCACGG CAAGAGGCGA GAG-----CG GCGA---CTG --GTGAGTAC
22_01A1.CM.06.LPH27MF.JN864049   ---------- ---------- ---------- ---------- ---A------ GCGCGCACGG CAAGAGGCGA GAG-----CG GCGA---CTG --GTGAGTAC
22_01A1.CM.10.LB054.JN864059     ---------- ---------- ---------- ---------- ---------- ---------- ---------- --------CG GCAC---AGG --TTGA-T-C
23_BG.CU.03.CB118.AY900571       CCAGAGAAG- --TTCTCTCG ACGCAGG--A CTCGGCTTGC TGAG------ GTGCACACAG CAAGAGGCGA GAG-----CG GCGA---CTG --GTGAGTAC
23_BG.CU.03.CB347.AY900572       CCAGAGAAG- --TTCTCTCG ACGCAGG--A CTCGGCTTGC TGAG------ GTGCACACAG CAAGAGGCGA GAG-----CG GCGA---CTG --GTGAGTAC
24_BG.CU.03.CB378.AY900574       CCAGAGAAG- --TTCTCTCG ACGCAGG--A CTCGGCTTGC TGAG------ GTGCACACAG CAAGAGGCGA GAG-----CG GCGA---CTG --GTGAGTAC
24_BG.CU.03.CB471.AY900575       CCAGAGAAG- --TTCTCTCG ACGCAGG--A CTCGGCTTGC TGAG------ GTGCACACAG CAAGAGGCGA GAG-----CG GCGA---CTG --GTGAGTAC
24_BG.ES.08.X2456_2.FJ670526     CCAGAGAAG- --TTCTCTCG ACGCAGG--A CTCGGCTTGC TGAG------ GTGCACACAG CAAGAGGCGA GGAG----CG GCGA---CTG --GTGAGTAC
25_cpx.CM.06.06CM_BA_040.EU693   CCAGAGAAG- --TTCTCTCG ACGCAGG--A CTCGGCTTAC TGAA------ GTGCACACAG TAAGAGGCGA GAG-----CG GCGA---CTG --GTGAGTAC
25_cpx.SA.03.J11233.EU697906     CCAGAGAAG- --ATCTCTCG ACGCAGG--A CTCGGCTTGC TGAG------ GTGCACACAG CAAGAGGCGA GAG-----CG GCGG---CTG --GTGAGTAC
25_cpx.SA.03.J11451.EU697908     CCAGAGAAG- --TTCTCTCG ACGCAGG--A CTCGGCTTGC TGAG------ GTGCACACAG CAAGAGGCGA GAG-----CG GCGG---CTG --GTGAGTAC
26_AU.CD.02.02CD_KS069.FM87778   CCAGAGAAG- --TTCTCTCG ACGCAGG--A CTCGGCTTGC TGAG------ -TGCACACAG CAAGAGGCGA GGAG----CG GCGA---CTG --GTGAGTAC
26_AU.CD.02.02CD_LBTB084.FM877   CCAGAGAAG- --ATCTCTCG ACGCAGG--A CTCGGCTTGC TGAG------ -TGCACACAG CAAGAGGCGA GGGG----CG GCGA---CTG --GTGAGTAC
26_AU.CD.02.02CD_MBTB047.FM877   CCAGAGAAG- ---ACTCTCG ACGCAGG--A CTCGGCTTGC TGAG------ -TGCACACAG CAAGAGGCGA GGAG----CG GCGA---CTG --GTGAGTAC
26_AU.CD.97.97CD_KTB119.FM8777   CCAGAGAAG- --TTCTCTTG ACGCAGG--A CTCGGCTTGC TGAG------ -TGCACACAG CAAGAGGCGA GGAG----CG GCGA---CTG --GTGAGTAC
27_cpx.FR.04.04CD_FR_KZS.AM851   CCAGAGAAG- --TTCTCTCG ACGCAGG--A CTCGGCTTGC TGAA------ GTGCACACGG CAAGAGGCGA GGAG----CG GCGG---CTG --GTGAGTAC
28_BF.BR.05.0679SV.JF804812      CCAGAGGAG- --ATCTCTCG ACGCAGG--A CTCGGCTTGC TGAA------ GCGCGCACAG CAAGAGGCGA GGGG----CG GCGA---CTG --GTGAGTAC
28_BF.BR.99.BREPM12313.DQ08587   CCAGAGAAG- --CTCTCTCG ACGCAGG--A CTCGGCTTGC TGAA------ GCGCGCGCGG CAAGAGGCGA GGGG----CG GCGA---CTG --GTGAGTAC
28_BF.BR.99.BREPM12609.DQ08587   CCAGAGGAG- --CTCTCTCG ACGCAGG--A CTCGGCTTGC TGAA------ GCGCGCACGG CAAGAGGCGA GGGG----CG GCGA---CTG --GTGAGTAC
28_BF.BR.99.BREPM12817.DQ08587   CCAGAGGAG- --ATCTCTCG ACGCAGG--A CTCGGCTTGC TGAA------ GTGCGCACGG CAAGAGGCGA GGGG----CG GCGA---CTG --GTGAGTAC
29_BF.BR.01.BREPM16704.DQ08587   CCTGAGGAG- --CTCTCTCG ACGCAGG--A CTCGGCTTGC TGAA------ GCGCGCACAG CAAGAGGCCA GGGG----CC GCCA---CCG --GTGAGTAC
29_BF.BR.05.0264RI.JF804807      ---------- ---------- ---------- ---------- ---------- ---------G CAAGAGGCGA GGGG----CG GCGA---CTG --GTGAGTAC
29_BF.BR.99.BREPM11948.DQ08587   CCAGAGGAG- --CTCTCTCG ACGCAGG--A CTCGGCTTGC TGAA------ GCGCGCACAG CAAGAGGCGA GGGG----CG GCGA---CTG --GTGAGTAC
31_BC.BR.02.110PA.EF091932       CCAGAGGAG- --ATCTCTCG ACGCAGG--A CTCGGCTTGC TGAA------ GTGCACTCGG CAAGAGGCGA GAG-----CG GCGG---CTG --GTGAGTAC
31_BC.BR.04.04BR142.AY727527     CCAGAGGAG- --ATCTCTCG ACGCAGG--A CTCGGCTTGC TGAA------ GTGCACTCGG CAAGAGGCGA GAG-----CG GCGG---CTG --GTGAGTAC
32_06A1.EE.01.EE0369.AY535660    CCAGAGAAG- --TTCTCTCG ACGCAGG--A CTCGGCTTGC TGAA------ GTGCACACGG CAAGAGGCGA GAG-----CG GCGA---CTG --GTGAGTAC
32_06A1.EE.02.EST2002_1169.DQ1   CCAGAGAAG- --TTCTCTCG ACGCAGG--A CTCGGCTTGC TGAA------ GTGCACACGG CAAGAGGCGA GAGC----GG CGCM---CTG --GTGAGTAC
33_01B.ID.07.JKT189_C.AB547463   ---------- ------CTCG ACGCAGG--A CTCGGCTTGC TGAG------ GTGCACACAG CAAGAGGCGA GAGG----CG GCGA---CTG --GTGAGTAC
33_01B.MY.05.05MYKL007_1.DQ366   CCAGAGAAG- --TTCTCTCG ACGCAGG--A CTCGGCTTGC TGAA------ GTGCACACAG CAAGAGGCGA GAG-----CG GCGA---CTG --GTGAGTAC
33_01B.MY.05.05MYKL015_2.DQ366   CCAGAGAAG- --TTCTCTCG ACGCAGG--A CTCGGCTTGC TGAG------ -TGCACACAG CAAGAGGCGA GAG-----CG GCGG---CTG --GTGAGTAC
33_01B.MY.05.05MYKL031_1.DQ366   CCAGAGAAG- --TTCTCTCG ACGCAGG--A CTCGGCTTGC TGAG------ GTGCACACAG CAAGAGGCGA GAG-----CG GCGA---CTG --GTGAGTAC
33_01B.MY.05.05MYKL045_1.DQ366   CCAGAGAAG- --TTCTCTCG ACGCAGG--A CTCGGCTTGC TGAG------ GTGCACACAG CAAGAGGCGA GAG-----CG GCGA---CTG --GTGAGTAC
38_BF1.UY.03.UY03_3389.FJ21378   CCAGAGAAG- --ATCTCTCG ACGCAGG--A CTCGGCTTGC TGAA------ GCGCGCGCAG CAAGAGGCGA GAG-----CG GCGA---CTG --GTGAGTAC
38_BF1.UY.04.UY04_3987.FJ21378   CCAGAGAAG- --ATCTCTCG ACGCAGG--A CTCGGCTTGC TGAA------ GTGCGCACGG CAAGAGGCGA GAG-----CG GCGG---CTG --GTGAGTAC
38_BF1.UY.04.UY04_4022.FJ21378   CCAGAGGAG- --ATCTCTCG ACGCAGG--A CTCGGCTTGC TGAA------ GCGCGCACAG CAAGAGGCGA GAG-----CG GCGA---CTG --GTGAGTAC
38_BF1.UY.05.UY05_4752.FJ21378   CCAGAGAAG- --ATCTCTCG ACGCAGG--A CTCGGCTTGC TGAA------ GCGCGCGCAG CAAGAGGCGA GAG-----CG GCGA---CTG --GTGAGTAC
39_BF.BR.03.03BRRJ103.EU735534   CCAGAGGAG- --ATCTCTCG ACGCAGG--A CTCGGCTTGC TGAA------ GTGCGCACGG CAAGAGGCGA GGGG----CG GCGA---CTG --GTGAGTAC
39_BF.BR.03.03BRRJ327.EU735536   CCAGAGAAG- --ATCTCTCG ACGCAGG--A CTCGGCTTGC TGAA------ GCGCGCACGG CAAGAGGCGA GGGG----CG GCGA---CTG --GTGAGTAC
39_BF.BR.04.04BRRJ179.EU735535   CCAGAGGAG- --ATCTCTCG ACGCAGG--A CTCGGCTTGC TGAA------ GCGCGCACGG CAAGAGGCGA GGGG----CG GCGA---CTG --GTGAGTAC
40_BF.BR.04.04BRRJ115.EU735538   CCAGAGGAG- --ATCTCTCG ACGCAGG--A CTCGGCTTGC TGAA-----A GCGCGCTCGG CAAGAGGCGA GGGGA---CG GCGA---CTG --GTGAGTAC
40_BF.BR.04.04BRSQ46.EU735540    CCAGAGGAG- --CTCTCTCG ACGCAGG--A CTCGGCTTGC TGAA-----A GCGCGCTCGG CAAGAGGCGA GGGG----CG GCGA---CTG --GTGAGTAC
40_BF.BR.05.05BRRJ055.EU735537   CCAGAGGAG- --CTCTCTCG ACGCAGG--A CTCGGCTTGC TGAA-----A GCGCGCGCAG CAAGAGGCGA GGGG----CG GCGA---CTG --GTGAGTAC
40_BF.BR.05.05BRRJ200.EU735539   CCAGAGAAG- --ATCTCTCG ACGCAGG--A CTCGGCTTGC TGAA-----A GCGCGCTCGG CAAGAGGCGA GGGG----CG GCGGA--CTG --GTGAGTAC
42_BF.LU.03.luBF_01_03.EU17015   CCAGAGGAG- --CTCTCTCG ACGCAGG--A CTCGGCTTGC TGAA------ GTGCGCACGG CAAGAGGCGA GGGG----CG GCGA---CTG --GTGAGTAC
42_BF.LU.03.luBF_02_03.EU17015   CCAGAGGAG- --CTCTCTCG ACGCAGG--A CTCGGCTTGC TGAA------ GTGCGCACGG CAAGAGGCGA GGGG----CG GCGA---CTG --GTGAGTAC
42_BF.LU.03.luBF_04_03.EU17014   CCAGAGGAG- --CTCTCTCG ACGCAGG--A CTCGGCTTGC TGAA------ GTGCGCACGG CAAGAGGCGA GGGG----CG GCGA---CTG --GTGAGTAC
42_BF.LU.03.luBF_05_03.EU17015   CCAGAGGAG- --CTCTCTCG ACGCAGG--A CTCGGCTTGC TGAA------ GTGCGCACGG CAAGAGGCGA GGGG----CG GCGA---CTG --GTGAGTAC
42_BF.LU.03.luBF_06_03.EU17014   CCAGAGGAG- --CTCTCTCG ACGCAGG--A CTCGGCTTGC TGAA------ GTGCGCACGG CAAGAGGCGA GGGG----CG GCGA---CTG --GTGAGTAC
42_BF.LU.03.luBF_07_03.EU17014   CCAGAGGAG- --CTCTCTCG ACGCAGG--A CTCGGCTTGC TGAA------ GTGCGCACGG CAAGAGGCGA GGGG----CG GCGA---CTG --GTGAGTAC
42_BF.LU.03.luBF_08_03.EU17014   CCAGAGGAG- --CTCTCTCG ACGCAGG--A CTCGGCTTGC TGAA------ GTGCGCACGG CAAGAGGCGA GGGG----CG GCGA---CTG --GTGAGTAC
42_BF.LU.03.luBF_09_03.EU17015   CCAGAGGAG- --CTCTCTCG ACGCAGG--A CTCGGCTTGC TGAA------ GTGCGCACGG CAAGAGGCGA GGGG----CG GCGA---CTG --GTGAGTAC
42_BF.LU.04.luBF_11_04.EU17014   CCAGAGGAG- --CTCTCTCG ACGCAGG--A CTCGGCTTGC TGAA------ GTGCGCACGG CAAGAGGCGA GGGG----CG GCGA---CTG --GTGAGTAC
42_BF.LU.04.luBF_12_04.EU17014   CCAGAGGAG- --CTCTCTCG ACGCAGG--A CTCGGCTTGC TGAA------ GTGCGCACGG CAAGAGGCGA GGGG----CG GCGA---CTG --GTGAGTAC
42_BF.LU.05.luBF_13_05.EU17013   CCAGAGGAG- --CTCTCTCG ACGCAGG--A CTCGGCTTGC TGAA------ GTGCGCACGG CAAGAGGCGA GGGG----CG GCGA---CTG --GTGAGTAC
42_BF.LU.05.luBF_14_05.EU17014   CCAGAGGAG- --CTCTCTCG ACGCAGG--A CTCGGCTTGC TGAA------ GTGCGCACGG CAAGAGGCGA GGGG----CG GCGA---CTG --GTGAGTAC
42_BF.LU.05.luBF_16_05.EU17015   CCAGAGGAG- --CTCTCTYG ACGCAGG--A CTCGGCTTGC TGAA------ GTGCGCACGG CAAGAGGCGA GGGG----CG GCGA---CTG --GTGAGTAC
42_BF.LU.05.luBF_17_05.EU17014   CCAGAGGAG- --CTCTCTCG ACGCAGG--A CTCGGCTTGC TGAA------ GTGCGCACGG CAAGAGGCGA GGGG----CG GCGA---CTG --GTGAGTAC
42_BF.LU.06.luBF_18_06.EU17013   CCAGAGGAG- --CTCTCTCG ACGCAGG--A CTCGGCTTGC TGAA------ GTGCGCACGG CAAGAGGCGA GGGG----CG GCGA---CTG --GTGAGTAC
42_BF.LU.06.luBF_19_06.EU17013   CCAGAGGAG- --CTCTCTCG ACGCAGG--A CTCGGCTTGC TGAA------ GTGCGCACGG CAAGAGGCGA GGGG----CG GCGA---CTG --GTGAGTAC
43_02G.SA.03.J11223.EU697904     CCAGAGAAG- --TTCTCTCG ACGCAGG--A CTCGGCTTGC TGAG------ GTGCACACAG CAAGAGGCGA GAG-----CG GCGA---CTG --GTGAGTAC
43_02G.SA.03.J11232.EU697905     CCAGAGAAG- --TTCTCTCG ACGCAGG--A CTCGGCTTGC TGAG------ GTGCACAAAG CAAGAGGCGA GAG-----CG GCGA---CTG --GTGAGTAC
43_02G.SA.03.J11243.EU697907     CCAGAGAAG- --TTCTCTCG ACGCAGG--A CTCGGCTTGC TGGG------ -TGCACACGG CAAGAGGCGA GAG-----CG GCGA---CTG --GTGAGTAC
43_02G.SA.03.J11456.EU697909     CCAGAGAAG- --TTCTCTCG ACGCAGG--A CTCGGCTTGC TGAG------ GTGCACACAG CAAGAGGCGA GAG-----CG GCGA---CTG --GTGAGTAC
44_BF.CL.00.CH80.FJ358521        CCAGAGGAG- --ATCTCTCG ACGCAGG--A CTCGGCTTGC TGAA------ GTGCACACGG CAAGAGGCGA GAG-----GG GCGA---CTG --GTGAGTAC
44_BF.CL.01.CH12.AY536235        CCAGAGGAG- --ATCTCTCG ACGCAGG--A SYMGGCTTGC TGAA------ GTGCACAAGG CAAGMGGCGA GRGGAG--CG GCGA---CTG --GTGAGTAC
45_cpx.CD.97.97CD_MBFE185.FN39   CCAGAGAAG- --ATCTCTCG ACGCAGG--A CTCGGCTTGC TGAG------ GTGCACACAG CAAGAGGCGA GAG-----CG GCGA---CTG --GTGAGTAC
45_cpx.CD.97.97CD_MBS30.FN3928   CCAGAGAAG- --TTCTCTCG ACGCAGG--A CTCGGCTTGC TGAG------ GTGCACACAG CAAGAGGCGA GAG-----CG GCGA---CTG --GTGAGTAC
45_cpx.CM.97.97CM_MP814.FN3928   CCAGAGAAG- --ATCTCTCG ACGCAGG--A CTCGGCTTGS TGAG------ GTGCACACAG CAAGAGGCGA GAG-----CG GCGA---CTG --GTGAGTAC
45_cpx.FR.04.04FR_AUK.EU448295   CCAGAGAAG- --TTCTCTCG ACGCAGG--A CTCGGCTTGC TGAG------ GTGCACACAG CAAGAGGCGA GAG-----CG GCGA---CTG --GTGAGTAC
45_cpx.GA.97.97GA_TB45.FN39287   CCAGAGAAG- --TTCTCTCG ACGCAGG--A CTCGGCTTGC TGAG------ GTGCACACAG CAAGAGGCGA GAG-----CG GCGA---CTG --GTGAGTAC
46_BF.BR.01.01BR087.DQ358801     CCAGAGAAG- --ATCTCTCG ACGCAGG--A CTCGGCTTGC TGAA------ GTGCACACGG CAAGAGGCGA GAG-----CG GCGA---CTG --GTGAGTAC
46_BF.BR.01.01BR125.DQ358802     CCAGAGAAG- --ATCTCTCG ACGCAGG--A CTCGGCTTGC TGAA------ GTGCACACGG CAAGAGGCGA GAG-----CG GCGG---CTG --GTGAGTAC
46_BF.BR.07.07BR_FPS625.HM0264   CCAGAGAAG- --ATCTCTCG ACGCAGG--A CTCGGCTTGC TGAA------ GTGCACACGG CAAGAGGCGA GAG-----CG GCGA---CTG --GTGAGTAC
46_BF.BR.07.07BR_FPS742.HM0264   CCAGAGGAG- --ATCTCTCG ACGCAGG--A CTCGGCTTGC TGAA------ GTGCACACGG CAAGAGGCGA GAG-----CG GCGA---CTG --GTGAGTAC
46_BF.BR.07.07BR_FPS783.HM0264   CCAGAGAAG- --ATCTCTCG ACGCAGG--A CTCGGCTTGC TGAA------ GTGCACACGG CAAGAGGCGA GAG-----CG GCGA---CTG --GTGAGTAC
46_BF.BR.07.07BR_FPS810.HM0264   CCAGAGAAG- --ATCTCTCG ACGCAGG--A CTCGGCTTGC TGAA------ GTGCGCACGG CAAGAGGCGA GAG-----CG GCGA---CTG --GTGAGTAC
46_BF.BR.07.07BR_FPS812.HM0264   CCAGAGAAG- --TTCTCTCG ACGCAGG--A CTCGGCTTGC TGAA------ GTGCACACGG CAAGAGGCGA GAG-----CG GCGA---CTG --GTGAGTAC
47_BF.ES.08.P1942.GQ372987       CCAGAGAAG- --CTCTCTCG ACGCAGG--A CTCGGCTTGC TGAA------ GCGCGCTCGG CAAGAGGCGA GGGG----CG GCGA---CTG --GTGAGTAC
47_BF.ES.08.X2457_2.FJ670529     CCAGAGAAG- --CTCTCTCG ACGCAGG--A CTCGGCTTGC TGAA------ GCGCGCTCGG CAAGAGGCGA GGGG----CG GCGA---CTG --GTGAGTAC
49_cpx.GM.02.N18380.HQ385477     CCAGAGAAG- --CTCTCTCG ACGCAGG--A CTCGGCTTGC TGAA------ GTGCACACGG CAAGAGGCGA GAG-----CG GCGA---CTG --GTGAGTAC
49_cpx.GM.03.N26677.HQ385479     CCAGAAAAG- --ATCTCTCG ACGCAGG--A CTCGGCTTGC TGAA------ GTGCACACGG CAAGAGGCGA GAG-----CG GCGA---CTG --GTGAGTAC
49_cpx.GM.97.N28353.HQ385478     CCAGAGAAG- --CTCTCTCG ACGCAGG--A CTCGGCTTGC TGAA------ GTGCACACGG CAAGAGGCGA GAG-----CG GCGA---CTG --GTGAGTAC
52_01B.MY.03.03MYKL018_1.DQ366   ---------- ---TCTCTCG ACGCAGG--A CTCGGCYTGC TGAG------ GTGCACACAG CAAGAGGCGA RAG-----CG GCGA---CTG --GTGAGTAC
53_01B.MY.04.04MYKL016_1.DQ366   ---------- ---------G ACGCAGG--A CTCGGCTTGC TGAG------ GTGCACACAG CAAGAGGCGA GAG-----CG GCGA---CTG --GTGAGTAC
```

```
53_01B.MY.10.10MYKJ067.JX39061   CCAGAGAAG- --TTCTCTCG ACGCAGG--A CTCGGCTTGC TGA------- GTGCACACAG CAAGAGGCGA GGAG----CG GCGA---CTG --GTGAGTAC
53_01B.MY.10.10MYKJ079.JX39061   CCAGAGAAG- --TTCTCTCG ACGCAGG--A CTCGGCTTGC TGAG------ GTGCACGCAG CAAGAGGCGA GAG-----CG GCGA---CTG --GTGAGTAC
53_01B.MY.11.11FIR164.JX390610   CCAGAGGAG- --TTCTCTCG ACGCAGG--A CTCGGCTTGC TGAG------ GTGCACACAG CAAGAGGCGA GAG-----CG GCGA---CTG --GTGAGTAC
54_01B.MY.07.07MYKLD49.EU03191   CCAGAGAAG- --TTCTCTCG ACGCAAGG-A CTCGGCTTGC TGCA------ GCGCGCACAG CAAGAGGCGA GAG-----CG GCGA---CTG --GTGAGTAC
54_01B.MY.08.08MYKL044.JX39097   CCAGAGAAG- --TTCTCTCG ACGCAGG--A CTCGGCTTGC TGAAGT---- GCGCGCACAG CAAGAGGCGA GAG-----CG GCGA---CTG --GTGAGTAC
54_01B.MY.09.09MYSB023.JX39097   GGAGAGAAG- --TTCTCTCG ACGCAGG--A CTCGGCTTGC TGAAGT---- GCGCGCACAG CAAGAGGCGA GAG-----CG GCGA---CTG --GTGAGTAC
55_01B.CN.10.HNCS102056.JX5746   ---------- ---TCTCTCG ACGCAGG--A CTCGGCTTGC TGAG------ GTGCACACAG CAAGAGGCGA GAG-----CG GCGA---CTG --GTGAGTAC
55_01B.CN.11.GDDG318.JX574662    ---------- ---TCTCTCG ACGCAGG--A CTCGGCTTGC TGAG------ GTGCACACAG CAAGAGGCGA GAG-----CG GCGA---CTG --GTGAGTAC
01A1.MM.99.mCSW105.AB097872      CCAGAGAAG- --TTCTCTCG ACGCAGG--A CTCGGCTTGC TGAG------ GTGCACACAG CAAGAGGCGA GAG-----CG GCGA---CTG --GTGAGTAC
01B.CN.08.08CYM047.JF340054      CCAGAGAAG- --TTCTCTCG ACGCAGG--A CTCGGCTTGC TGAG------ GTGCACACAG CAAGAGGCGA GAG-----CG GCGA---CTG --GTGAGTAC
01B.JP.x.pHIV_1_Y271B01AE64.AB   CCAGAGGAG- --ATCTCTCG GCGCAGG--A CTCGGCTTGC TGCA------ GCGCGCGCGG CAAGAGGCGA GGGG----CG GCGA---CTG --GTGAGTAC
01B.MM.00.mIDU502.AB097865       CCAGAGGAG- --CTCTCTCG ACGCAGG--A CTCGGCTTGC TGAA------ GCGCGCGCAG CAAGAGGCGA GAG-----GG GCGA---CTG --GTGAGTAC
01B.MM.99.mCSW104.AB097867       CCAGAGAAG- --TTCTCTCG ACGCAGG--A CTCGGCTTGC TGAG------ GTGCACACAG CAAGAGGCGA GAG-----CG GCGA---CTG --GTGAGTAC
01B.MY.04.04MYKL019_1.DQ366665   ---------- ---TCTCTTG ACGCAGG--A CTCGGCTTGC TGAG------ GTGCACACAG CAAGAGGCGA GAG-----CG GCGA---CTG --GTGAGTAC
01B.MY.05.05MYKL043_1.DQ366666   CCAGAGAAG- --TTCTCTCG ACGCAGG--A CTCGGCTTGC TGAG------ GTGCACACAG CAAGAGGCGA GAG-----CG GCGA---CTG --GTGAGTAC
01B.MY.06.06MMYKLD46.EF495062    CCAGAGAAG- --TTCTCTCG ACGCAGG--A CTCGGCTTGC TGAG------ GTGCACACAG CAAGAGGCGA GAG-----CG GCGA---CTG --GTGAGTAC
01B.MY.07.07MYKLD47.EU031913     ---------- ---------- ---------- ---------- ---------- GTGCACACAG CA-GAGGCGA GAG-----CG GCGA---CTG --GTGAGTAC
01B.MY.07.07MYKLD48.EU031914     CCAGAGAAG- --TTCTCTCG ACGCAGG--A CTCGGCTTGC TGAG------ GTGCACACAG CAAGAGGCGA GAG-----CG GCGA---CTG --GTGAGTAC
01B.TH.05.05TH140456.JN631793    CCAGAGAAG- --TTCTCTCG ACGCAGG--A CTCGGCTTGC TGAG------ GTGCACACAG CAAGAGGCGA GAG-----CG GCGA---CTG --GTGAGTAC
01B.TH.05.AA095a_WG21.JX447830   CCAGAGAAG- --TTCTCTCG ACGCAGG--A CTCGGCTTGC TGAG------ GTGCACACAG CAAGAGGCGA GAG-----CG GCGG---CTG --GTGAGTAC
01B.TH.06.AA020a_wg2.JX446927    CCAGAGAAG- --TTCTCTCG ACGCAGG--A CTCGGCTTGC TGAG------ GTGCACACAG CAAGAGGCGA GAG-----CG GCGA---CTG --GTGAGTAC
01B.TH.06.AA025a_WG13.JX447000   CCAGAGAAG- --TTCTCTCG ACGCAGG--A CTCGGCTTGC TGAG------ GTGCACACAG CAAGAGGCGA GAG-----CG GCGA---CCG --GTGAGTAC
01B.TH.06.AA084a_WG10.JX447668   CCAGAGAAG- --TTCTCTCG ACGCAGG--A CTCGGCTTGC TGAG------ GYGCACACAG CAAGAGGCGA GAG-----CG GCGA---CAG -GGTGAGTAC
01B.TH.91.CM237.AY167123         CCAGAGGAG- --ATCTCTCG ACGCAGG--A CTCGGCTTGC TGAA------ GCGCGCACAG CAAGAGGCGA GGGG----CG GCGA---ACG --GTGAGTAC
01BC.MM.00.mCSW503.AB097866      ---------- ---C-TCTCG ACGCAGG--A CTCGGCTTGC TGAG------ GTGCACACAG CAAGAGGCGA GAG-----CG GCGA---CTG --GTGAGTAC
01BC.MM.99.mIDU107.AB097868      CCAGAGAAG- --TTCTCTCG ACGCAGG--A CTCGGCTTGC TGAA------ GTGCACTCGG C-AGAGGCGA GAG-----CG GCGA---CTG --GTGAGTAC
01F2G.CM.02.LT31.JN864056        ---------- ---------- ---------- ---------- ---------- ---------- --------GA AAG-----CG GCGA---CTG --GTGAGTAC
0206.DZ.10.DEURF10DZ001.JX1406   ---------- ---------- ---------- ---------- TGAG------ GTGCACACAG CAAGAGGCGA GAG-----CG GCGG---CTG --GTGAGTAC
0206.GH.03.03GH195AG_06.AB2868   CCAGAGAAG- --ATCTCTCG ACGCAGGG-A CTCGGCTTGC TGAA------ GTGCACACAG CAAGAGGCGA GAG-----CG GCGA---CTG --GTGAGTAC
0206.NE.00.NE36.AJ508597         CCAGAGAAG- --ATCTCTCG ACGCACGG-A CTCGGCTTGC TGAG------ GTGCACACAG CAAGAGGCGA GAG-----CG GCGA---CTG --GTGAGTAC
0206.NE.00.NE95.AJ508596         CCAGAGAAG- --TTCTCTCG ACGCAGG--A CTCGGCTTGC TGAA-----G GTGCACACAG CAAGAGGCGA GAG-----CG GCGA---CTG --GTGAGTAC
0206.NE.97.NE03.AJ508595         CCAGAGAAG- --TTCTCTCG ACGCAGG--A CTCGGCTTGC TGAG------ GTGCACACAG CAAGAGGCGA GAG-----CG GCGA---CTG --GTGAGTAC
0209.CI.01.01IC_17395.AJ866554   CCAGAGAAG- --TTCTCTCG ACGCAGG--A CTCGGCTTGC TGAG------ GTGCACACAG CAAGAGGCGA GAG-----CG GCGA---CTG --GTGAGTAC
0209.CI.01.01IC_PCI118.AJ86655   CCAGAGAAG- --TTCTCTCG ACGCGAGG-A CTCGGCTTGC TGAG------ GTGCACACAG CAAGAGGCGA GAG-----CG GCGA---CTG --GTGAGTAC
0209.CI.01.01IC_PCI127.AJ86655   CCAGAGAAG- --TTCTCTCG ACGCAGG--A CTCGGCTTGC TGAG------ GTGCACACAG CAAGAGGCGA GAG-----AG GCGA---CTG --GTGAGTAC
0209.CI.97.97IC_PCI3.AJ866555    CCAGAGAAG- --TTCTCTCG ACGCAGG--A CTCGGCTTGC TGAG------ GTGCACACAG CAAGAGGCGA GAG-----CG GCGA---CTG --GTGAGTAC
0222.CM.02.02CAMLT04.EU743964    ---------- ---------- ---------- ------TTGT GTGAGGG--- GCGCCCCCCG CAAGGGGCGA GAG-----CG GCGA---CTG --GTGAGTAC
0222.CM.08.BDSH129.JN864052      ---------- ---------- ---------- ---------- ---------- --GCGCACGG CAAGAGGCGA GAG-----CG GCGA---CTG --GTGAGTAC
0222.CM.10.LB045.JN864053        CCAGAGAAG- --ATCTCTCG ACGCAAGG-A CTCGGCTTGC TGAG------ GTGCACACGG CAAGAGGAGA GAG-----CG GCGA---CGG --GTGAGTAC
02A1.ES.05.X230_10.FJ670515      CCAGAGAAG- --ATCTCTCG ACGCACGG-A CTCGGCTTGC TGAG------ GTGCACACAG CAAGAGGCGA GAG-----CG GCGA---CTG --GTGAGTAC
02A1.ES.07.ES_P1751.GQ372986     CCAGAGAAG- --TTCTCTCG ACGCAGG--A CTCGGCTTGC TGAA------ GTGCACGCAG CAAGAGGCGA GAG-----CG GCGA---CTG --GTGAGTAC
02A1.GH.03.GHNJ176.AB231894      CCAGAGAAG- --ATCTCTCG ACGCAGGG-A CTCGGCTTGC TGAG------ GTGCACACAG CAAGAGGCGA GAG-----CG GCGA---CTG --GTGAGTAC
02A1.GH.97.97GH_AG2.AB052867     CCAGAGAAG- --ATCTCTCG ACGCACGG-A CTCGGCTTGC TGAG------ GTGCACACAG CAAGAGGCGA GAG-----CG GCGA---CTG --GTGAGTAC
02A1.RU.10.10RU6637.JN230353     CCAGAGAAG- --TTCTCTCG ACGCAGGG-G CTCGGCTTGC TGAG------ GTGCACACAG CAAGAGGCGA GAG-----CG GCGA---CTG --GTGAGTAC
02B.ES.99.99SP_11339.DQ926899    CCAGAGAAG- --ATCTCTCG ACGCAGG--A CTCGGCTTGC TGAG------ GTGCACGCAG CAAGAGGCGA GAG-----CG GCGA---CTG --GTGAGTAC
02B.FR.02.URF4.JN882652          CCAGAGAAG- --CTCTCTCG ACGCAAGG-A CTCGGCTTGC TGAG------ GTGCACACAG CAAGAGGCGA GAG-----CG GCGA---CTG --GTGAGTAC
02B.FR.09.URF2.JN882654          CCAGAGAAG- --TTCTCTCG ACGCAGG--A CTCGGCTTGC TGAA------ GCGCGCACAG CAAGAGGCGA GAG-----CG GCGA---CTG --GTGAGTAC
02BD.FR.08.URF1.JN882653         CCAGAGAAG- --TTCTCTCG ACGCAGG--A CTCGGCTTGC TGAA------ GTGCACACAG CAAGAGGCGA GAG-----CG GCGA---CTG --GTGAGTAC
02BG.FR.10.URF5.JN882655         CCAGAGAAG- --TTCTCTCG ACGCAGG--A CTCGGCTTGC TGAG------ GTGCACACAG CAAGAGGCGA GAG-----CG GCGA---CTG --GTGAGTAC
02C.BE.93.VI1035.AJ276595        ---------- --TTCTCTCG ACGCAGG--A CTCGGCTTGC TGAG------ GTGCACACAG CAAGAGGCGA GAG-----CG GCGA---CTG --GTGAGTAC
02D.GH.03.GHNJ193.AB231897       CCAGAGAAG- --ATCTCTCG ACGCAGGG-A CTCGGCTTGC TGAG------ GTGCACACAG CAAGAGGCGA GAG-----CG GCGA---CTG --GTGAGTAC
02D.GH.03.p03GH194AG09.AB48004   CCAGAGAAG- --TTCTCTCG ACGCAGGG-A CTCGGCTTGC TGAG------ -TGCACACGG CAAGAGGCGA GCG-----CG GCGA---CTG --GTGAGTAC
02F2G.CM.02.LT66.JN864057        ---------- ---------- ---------- ------TTGC TGAA------ GCGCGCACGG CAAGAGGCGA GGGG----CG GGGA---GTG --GGGAGTAC
02GK.CI.01.01IC_PCI123.AJ86655   CCAGAGAAG- --TTCTCTCG ACGCAGG--A CTCGGCTTGC TGAG------ GTGCACACAG CAAGAGGCGA GAG-----CG GCGA---CTG --GTGAGTAC
06A1.BJ.x.B76.AJ293865           CCAGAGAAG- --TTCTCTCG ACGCAGG--A CTCGGCTTGC TGAG------ -TGCACGCAG CAAGAGGCGA GGCGGC--CG GCGA---CTG --GTGAGTAC
1819.CU.99.CU64.AY894995         CCAGAGAAG- --ATCTCTCG ACGCAGG--A CTCGGCTTGC TGAG------ -TGCACGCAG CAAGAGGCGA GAGCGG---- --GA---CTG --GTGAGTAC
26C.CD.02.02CD_LBTB032.FM87777   CCAGAGAAG- --TTCTCTCG ACGCAAG--A CTCGGCTTGC TGAG------ -TGCACACGG CAAGAGGCGA GAAG----CG GCGA---CTG --GTGAGTAC
26C.CD.97.97CD_KFE267.FM877778   CCAGAGAAG- -GTTCTCTCG ACGCAGG--A CTCGGCTTGC TGAG------ -TGCACACAG CAAGAGGCGA GGAG----CA GCGA---CTG --GTGAGTAC
26C.CD.97.MBFE250.FM877783       CCAGAGAAG- --TTCTCTCG ACGCAGG--A CTCGGCTTGC TGAG------ -TGCACACAG CAAGAGGCGA GGAG----CG GCGA---CTG --GTGAGTAC
A1B.EE.02.EST2002_394.DQ167216   CCAGAGAAG- --TTCTCTCG ACGCAGG--G CTCGGCTT-G TGAG------ GTGCACACAG CAAGAGGCGA GAG-----CG GCGA---CTG --GTGAGTAC
A1BD.GB.03.34567.JN417238        CCAGAGGAG- --ATCTCTCG ACGCAGG--A CTCGGCTTGC TGAA------ GCGCGCACGG CAAGAGGCGA GGGG----AG GCGA---CTG --GTGAGTAC
A1C.CA.04.04CA7750.EU220698      CCAGAGAAG- --TTCTCTCG ACGCAGG--A CTCGGCTTGC TGAG------ GTGCACACAG CAAGAGGCGA GAG-----CG GCGA---CTG --GTGAGTAC
A1C.IN.01.1579A.DQ083238         CCAGAGAAG- --TTCTCTCG ACGCAGG--A CTCGGCTTGC TGAG------ GTGCACACAG CAAGAGGCGA GAG-----CG GCGA---CTG --GTGAGTAC
A1C.IN.95.95IN21301.AF067156     CCAGAGGAG- --ATCTCTCG ACGCAGG--A CTCGGCTTGC TGAA------ GTGCACTCGG CAAGAGGCGA GAG-----CG GCGA---CTG --GTGAGTAC
A1C.RW.92.92RW009_06.U88823      CCAGAGGAG- --ATCTCTCG ACGCGGG--A CTCGGCTTGC TGAA-----A GTGCACTCGG CAAGAGGCGA GAG-----CG GCGG---CTG --GTGAGTAC
A1CD.KE.02.ML2000.EU110093       CCAGAGAAG- --CTCTCTCG ACGCAGG--A CTCGGCTTGC TGAG------ GTGCACACAG CAAGAGGCGA GAG-----CG GCGA---CTG --GTGAGTAC
A1CD.SE.95.SE8603.AF075702       ---------- --TTCTCTCG ACGCAGG--A CTCGGCTTGC TGAA------ GCGCGCACGG CAAGAGGCGA GGGG----CA GCGA---ACG --GTGAGTAC
A1CDGKU.ZA.99.CM4_99ZACM4.AF41   CCAGAGGAG- --ATCTCTCG ACGCAGG--A CTCGGCTTGC TGAG------ -CGCGCACGG CAAGAGGCGA GGGG----CA GCGA---CTG --GTGAGTAC
A1CG.KE.02.ML1979PCR.EU110096    ---------- -----TCTCG ACGCAGG--A CTCGGCTTGC TGAG------ GTGCACACAG CAAGAGGCGA GAG-----CG GCGA---CTG --GTGAGTAC
A1D.DK.96.FSA.DQ912822           CCAGAGAAG- --ATCTCTCG ACGCAGG--A CTCGGCTTGC TGAA------ GTGCGCACGG CAAGCGGCGA GGGA----CA GCGA---ACA --GTGAGTAC
A1D.DK.98.MA.DQ912823            CCAGAGAAG- --ATCTCTCG ACGCAGG--A CTCGGCTTGC TGAA------ GTGCGCACGG CAAGAGGCGA GGGA----CA GCGA---ACA --GTGAGTAC
A1D.GB.00.8179.JN417236          CCAGAGGAG- --ATCTCTCG ACGCAGG--A CTCGGCTTGC TGAA------ GTGCACACGG CAAGAGGCGA GGGG----CA GCGA---ACG --GTGAGTAC
A1D.GB.03.33365.JN417239         CCAGAGGAG- --ATCTCTCG ACGCAGG--A CTCGGCTTGC TGAA------ GTGCGCACAG CAAGAGGCGA GGGG----CA GCGA---ACG --GTGAGTAC
A1D.GB.10.11762.JN417241         CCAGAGGAG- --ATCTCTCG ACGCAGG--A CTCGGCTTGC TGAA------ GCGCGCACAG CAAGAGGCGA GGGG----CA GCGA---ACG --GTGAGTAC
A1D.GB.10.12792.JN417240         CCAGAGGAG- --ATCTCTCG ACGCAGG--A CTCGGCTTGC TGA------- GTGCACACGG CAAGAGGCGA GGGG----CA GCGA---ACG --GTGAGTAC
A1D.KE.02.ML1974.EU110090        CCAGAGGAG- --ATCTCTCG ACGCAGG--A CTCGGCTTGC TGAA------ GTGCGCACGG CAAGAGGCGA GGGG----CA GCGA---ACG --GTGAGTAC
A1D.SE.93.SE6954.AF075701        ---------- --TTCTCTCG ACGCAGG--A CTCGGCTTGC TGAA------ GCGCGCACGG CAAGAGGCGA GGGG----CA GCGA---ACG --GCGAGTAC
A1D.TZ.96.TZBFL0011.AF442569     CCAGAGGAG- --CTCTCTCG ACGCAGG--A CTCGGCTTGC TGAA------ GCGCGCACGG CAAGAGGCGA GGGG----CA GCGA---ACG --GTGAGTAC
A1D.TZ.96.TZBFL0088.AF442570     CCAGAGGAG- --CTCTCTCG ACGCAGG--A CTCGGCTTGC TGAA------ GCGCGCGCAG CAAGAGGCGA GGGG----CA GCGA---ACG --GTGAGTAC
A1D.TZ.97.TZBFL0086.AF442566     CCAGAGAAG- --ATCTCTCG ACGCAGG--A CTCGGCTTGC TGAA------ GTGCGCACGG CAAGAGGCGA GGGG----AA GCGA---ACG --GTGAGTAC
A1D.UG.07.p191947.JX236674       CCAGAGAAG- --TTCTCTCG ACGCAGG--A CTCGGCTTGC TGAG------ GTGCACACAG CAAGAGGCGA GAGC----GG CTGA---CTG --GTGAGTAC
A1D.UG.08.p191982.JX236675       CCAGAGAAG- --ATCTCTCG ACGCAGG--A CTCGGCTTGC TGAA------ GCGCGCACAG CAAGAGGCGA GGGG----CA GCGA---ACG --GTGAGTAC
A1D.UG.90.UG266.AY352657         CCAGAGAAG- --TTCTCTCG ACGCAGG--A CTCGGCTTGC TGAG------ GTGCACACAG CAAGAGGCGA GAGN----CG GCGA---CTG --GTGAGTAC
A1D.UG.92.UG035.AY352656         CCAGAGAAG- --TTCTCTCG ACGCAGG--A CTCGGCTTGC TGAG------ GTGCACACAG CAAGAGGCGA GAG-----CG GCGA---CTG --GTGAGTAC
A1DHK.NO.97.97NOGIL3.AJ237565    CCAGAGAAG- --CTCTCTCG ACGCAGG--A CTCGGCTTGC TGAA------ GTGCACACGG CAAGAGGCGA GGG-----CG GCGTGA-CTG --GTGAGTAC
A1DK.CD.85.MAL_MALCG.X04415      CCAGAGAAG- --TTCTCTCG ACGCAGG--A CTCGGCTTGC TGAG------ GTGCACACAG CAAGAGGCGA GAG-----CG GCGA---CTG --GTGAGTAC
A1G.BE.94.VI1197.AJ276596        ---------- --ATCTCTCG ACGCAGG--A CTCGGCTTGC TGAA-----G GTGCACACAG CAAGAGGCGA GAG-----CG GCGCA--CTG --GTGAGTAC
A1G.NG.92.92NG003.U88825         CCAGAGAAG- --TTCTCTCG ACGCAGG--A CTCGGCTTGC TGAG------ GTGCACACAG CAAGAGGCGA GAG-----CG GCGA---CTG --GTGAGTAC
A1G.RU.11.11RU6900.JX500697      CCAGAGAAG- --ATCTCTCG ACGCAGGG-A CTCGGCTTGC TGAG------ GTGCACGCAG CAAGAGGCGA GAG-----CG GCGA---CTG --GTGAGTAC
A1G.RU.11.11RU6939.JX500706      CCAGAGAAG- --ATCTCTCG ACGCGAGG-A CTCGGCTTGC TGAG------ GTGCACACAG CAAGAGGCGA GAG-----CG GCGA---CTG --GTGAGTAC
A1GHU.GA.x.VI354.AF076474        CCAGAGAAGA --TTCTCTCG ACGCAGG--A CTCGGCTTGC TGAG------ GTGCACACAG CAAGAGGCGA GAG-----CG GCGA---CTG --GTGAGTAC
A1GJ.BW.98.BW2117.AF192135       CCAGAGAAG- --TTCTCTCG ACGCAGG--A CTCGGCTTGC TGAA------ GTGCACACGG CAAGAGGCGA GAG-----CG GCGA---CTG --GTGAGTAC
A2C.ZA.98.DU178_98ZADU178.AF41   CCAGAGGAG- --ATCTCTCG ACGCAGG--A CTCGGCTTGC TGAA------ GTGCACTCGG CAAGAGGCGA GAG-----GG GCGA---CTG --GTGAGTAC
A2C.ZM.90.ZAM184.U86780          CCAGAGGAG- --ATCTCTCG ACGCAGG--A CTCGGCTTGC TGAA------ GTGCACCCGG CAAGAGGCGA GAG-----CG GCGG---CTG --GTGAGTAC
A2CD.KE.01.ML1956.EU110089       CCAGAGGAG- --ATCTCTCG ACGCAGG--A CTCGGCTTGC TGAA------ GCGCGCACGG CAAGCGGCGA GGGG----CA GCGA---CTG --GTGAGTAC
AC.KE.97.ML672.AY322191          CCAGAGAAG- --TTCTCTTG ACGCAGG--A CTCGGCTTGC TGCG------ GTGCACACAG CAAGAGGCGA GAG-----CG GCGA---CTG --GTGAGTAC
AC.ZA.04.04ZAPS204B1.DQ093606    CCAGAGCAG- --ATCTCTCG ACGCAGG--A CTCGGCTTGC TGAA------ GTGCACTCGG CAAGAGGCGA GAG-----CG GCGA---CTG --GTGAGTAC
AGKU.CD.x.BCF_Kita.AB485665      CCAGAGAAG- --ATCTCTCG ACGCAGG--A CTCGGCTTGC TGAG------ -TGCACACAG CAAGAGGCGA GGGG----CG GCGA---ACG --GTGAGTAC
BC.AR.01.ARE195FL.AY968312       ---------- --------GC A------GAA CTCGGCTTGC TGAA------ GTGCACTCGG CAAGAGGCGA GAG-----CG GCGG---CTG --GTGAGTAC
BC.BR.01.01_BR_RGS45.GQ365651    CCAGAGGAG- --ATCTCTCG ACGCAGG--A CTCGGCTTGC TGAA------ GTGCACTCGG CAAGAGGCGA GAG-----CG GCGG---CTG --GTGAGTAC
BC.BR.01.01_BR_RGS69.GQ365652    CCAGAGGAG- --ATCTCTCG ACGCAGG--A CTCGGCTTGC TGAA------ GTGCACTCGG CAAGAGGCGA GAG-----CG GCGA---CTG --GTGAGTAC
BC.BR.02.02_BR_HF31.GQ365649     CCAGAGGAG- --AACTCTCG ACGCAGG--A CTCGGCTTGC TGAA------ GTGCACTCGG CAAGAGGCGA GAG-----AG GCGG---CTG G-TGGAGTAC
BC.BR.05.05_BR_NSP24.GQ365650    CCAGAGGAG- --ATCTCTCG ACGCAGG--A CTCGGCTTGT TGAA------ GTGCACTCGA CAAGAGGCGA GAG-----CG GCGG---CTG --GTGAGTAC
BC.BR.06.06BR1114.JN692478       CCAGAGAAG- --ATCTCTCG ACGCAGG--G CTCGGCTTGC TGAA------ GTGCACTCGG CAAGAGGCGA GAG-----CG GCGA---CTG --GTGAGTAC
BC.BR.92.92BR023.HM100716        CCAGAGGAG- --ATCTCTCG ACGCAGG--A CTCGGCTTGC TGAA------ GTGCACTCGG CAAGAGGCGA GAG-----CG GCGA---CTG --GTGAGTAC
```

```
BC.CN.07.309.HM776938            CCAGAGGAG- --ATCTCTCG ACGCAGG--A CTCGGCTTGC TGAC------ GTGCACTCGG CAAGAGGCGA GAG-----CG GCGG---CTG --GTGAGTAC
BC.CN.07.341.HM776939            CCAGAGGAG- --ATCTCTCG ACGCAGG--A CTCGGCTTGC TGAA------ GTGCACGCAG CAAGAGGCGA GAG-----CG GCGA---CTG --GTGAGTAC
BC.IN.02.INDNARI_0218440.EU000   CCAGAGAAG- --ATCTCTCG ACGCAGG--G CTCGGCTTGC TGAA------ GTGCACTCGG CAAGAGGCGA GAG-----GG GCGG---CTG --GTGAGTAC
BC.IN.02.NARI7_3.EU000511        CCAGAGAAG- --ATCTCTCG ACGCAGG--A CTCGGCTTGC TGAA-----A GTGCACTCGG CAAGAGGCGA GAG-----GG GCGA---CTG --GTGAGTAC
BC.IN.02.NARI9_3.EU000508        CCGGAGAAG- --ATCTCTCG ACGCAGG--A CTCGGCTTGC TGAA------ GTGCACTCGG CAAGAGGCGA GGGG----GG GCGA---CTG --GTGAGTAC
BC.IN.99.NARI10_2.EU000516       CCAGAGAAG- --CTCTCTCG ACGCAGG--A CTCGGCTTGC TGAA------ GCGCGCACAG CAAGAGGCGA GAG-----CG GCGA---CTG --GCGAGTAC
BC.MM.99.mIDU103.AB097873        CCAGAGGAG- --CTCTCTCG ACGCAGG--A CTCGGCTTGC TGAA------ GCGCGCACAG CAAGAGGCGA GAG-----CG GCGA---CTG --GTGAGTAC
BCU.FR.06.06FR_CRN.EU448296      CCAGAGGAG- --ATCTCTCG ACGCAGG--A CTCGGCTTGC TGAA------ GTGCACTCGG CAAGAGGCGA GAG-----CG GCGA---CTG --GTGAGTAC
BF.BR.03.BREPM1026.EF637055      CCAGAGAAG- --CTCTCTCG ACGCAGG--A CTCGGCTTGC TGAA------ GCGCGCGCGG CAAGAGGCGA GGGG----CG GCGA---CTG --GTGAGTAC
BF.BR.03.BREPM1029.EF637052      ---------- ---------- ---------- ---------- -TAA------ GCGCGCACGG CAAAAGGCGA GGGG----CG GCGA---CTG --GTGAGTAC
BF1.BR.00.BREPM13853.DQ085875    CCAGAGGAG- --ATCTCTTG ACGCAGG--A CTCGGCTTGC TGAA------ GTGCGCACGG CAAGAGGCGA GGGG----CA GCGA---ACG --GTGAGTAC
BF1.BR.01.01BR042.DQ358799       CCAGAGAAG- --ATCTCTCG ACGCAGG--A CTCGGCTTGC TGAA------ GTGCGCACGG CAAGAGGCGA GAGGAG--CG GCGA---CTG --GTGAGTAC
BF1.BR.01.01BR047.DQ358800       CCAGAGGAG- --CTCTCTCG ACGCAGG--A CTCGGCTTGC TGAA------ GCGCGCACGG CAAGAGGCGA GGGG----CG GCGA---CTG --GTGAGTAC
BF1.BR.01.01BR226.DQ358803       CCAGAGAAG- --ATCTCTCG ACGCAGG--A CTCGGCTTGC TGAA------ GCGCGCACGG CAAGAGGCGA GGGG----CG GCGA---CTG --GTGAGTAC
BF1.BR.01.01BR323.DQ358804       CCAGAGGAG- --CTCTCTCG ACGCAGG--A CTCGGCTTGC TGAA------ GCGCGCGCGG CAAGAGGCGA GGGG----CG GCGA---CTG --GTGAGTAC
BF1.BR.02.02BR005.DQ358806       CCAGGGGAG- --CTCTCTCG ACGCAGG--A CTCGGCTTGC TGAA------ ACGCGCGCGG CAAGAGGCGA GGGG----CG GCGA---CTG --GTGAGTAC
BF1.BR.02.02BR006.DQ358807       CCAGAGGAG- --CTCTCTCG ACGCAGG--A CTCGGCTTGC TGAA------ GCGCGCGCGG CAAGAGGCGA GGGG----CG GCGA---CTG --GTGAGTAC
BF1.BR.02.02BR033.DQ358811       CCAGAGGAG- --CTCTCTCG ACGCAGG--A CTCGGCTTGC TGAA------ GCGCGCATAG CAAGAGGCGA GGGG----CG GCGA---CTG --GTGAGTAC
BF1.BR.02.02BR034.DQ358812       CCAGAGAAG- --CTCTCTCG ACGCGAGG-A CTCGGCTTGC TGAA------ GCGCGCACAG CAAGAGGCGA GGGG----CG GCGA---CTG --GTGAGTAC
BF1.BR.02.02BR2028.JN692437      CCAGAGAAG- --CTCTCTCG ACGCAGG--A CTCGGCTTGC TGAA------ GCGCGCGCGG CAAGAGGCGA GGGG----CG GCGA---CTG --GTGAGTAC
BF1.BR.03.03BR2018.JN692449      CCAGAGGAG- --CTCTCTCG ACGCAGG--A CTCGGCTTGC TGAA------ GCGCGCACGG CAAGAGGCGA GGGG----CG GCGA---CTG --GTGAGTAC
BF1.BR.03.03BR2019.JN692448      CCAGAGGAG- --ATCTCTCG ACGCAGG--A CTCGGCTTGC TGAA------ GCGCGCACAG CAAGAGGCGA GGGG----CG GCGA---ACG --GTGAGTAC
BF1.BR.04.04BR1067.JN692456      CCAGAGGAG- --ATCTCTCG ACGCAGG--A CTCGGCTTGC TGTA-----A GCGCGCTCGG CAAGAGGCGA GGGG----CG GCGA---CTG --GTGAGTAC
BF1.BR.05.0008SP.JF804805        CCAGAGGAG- --CTCTCTCG ACGCAGG--A CTCGGCTTGC TGAA------ GCGCGCACGG CAAGAGGCGA GGGG----CG GCGA---CTG --GTGAGTAC
BF1.BR.05.0632SV.JF804810        ---------- ---------- ---------- ---------- -GAA------ GCGCGCGCGG CAAGAGGCGA GGGG----CG GCGG---CTG --GTGAGTAC
BF1.BR.06.06BR_FPS561.HM026455   CCAGAGGAG- --ATCTCTCG ACGCAGG--A CTCGGCTTGC TGAA-----A GTGCACGCGG CAAGAGGCGA GGG-----GG GCGA---CTG --GTGAGTAC
BF1.BR.99.BREPM107.AY771588      ------AAG- --ATCTCTCG ACGCAGG--A CTCGGCTTGC TGAA------ GCGCGCACGG CAAGAGGCGA GGGG----CG GCGA---CTG --GTGAGTAC
BF1.BR.99.BREPM108.AY771589      CCAGAGGAG- --ATCTCTCG ACGCAGG--A CTCGGCTTGC TGAAGAA--- GTGCGCACGG CAAGAGGCGA GGGG----CG GCGA---CTG --GTGAGTAC
BF1.BR.99.BREPM11931.DQ085869    CCAGAGGAG- --CTCTCTCG ACGCAGG--A CTCGGCTTGC TGAA------ GCGCGCACAG CAAGAGGCGA GGGG----CG GCGA---CTG --GTGAGTAC
BF1.ES.08.ES_X2524_2.GQ372989    CCAGAGAAG- --ATCTCTCG ACGCAGG--A CTCGGCTTGC TGAA------ GCGCGCACGG CAAGAGGCGA GGGG----CG GCGA---CCG --GTGAGTAC
BF1.ES.08.X2432_2.FJ853621       CCAGAGGAG- --ATCTCTCG ACGCAGG--A CTCGGCTTGC TGAA------ GCGCGCACAG CAAGAGGCGA GGGG----CG GCGA---CTG --GTGAGTAC
BF1.ES.09.DEMBF09ES003.JX14066   CCAGAGGAG- --CTCTCTTG ACGCAGG--A CTCGGCTTGC TGAA------ GCGCGCACAG CAAGAGGCGA GGGG----CG GCGA---CCG --GTGAGTAC
BF1.ES.09.DEMBF09ES006.JX14066   CCAGAGAAG- --ATCTCTTG ACGCAGG--A CTCGGCTTGC AGCA------ GCGCGCGCGG CAAGAGGCGA GGGG----CG GCGA---CTG --GTGAGTAC
BF1.IT.01.53143.GU595149         ---------- ---------- ---------- ---------- ---------- CCCCGCACCG CAAAAAGCCA AGGG----CC GCCA---CCG --GTGAGTAC
BF1.IT.02.30638.GU595148         ---------- ---------- ---------- ---------- ---------- ---------- ---CAGGAGC CGAG----GG GAGC---CGA --CTGAGTAC
BF1.IT.02.57954.GU595150         CCAGAGAAG- --ATCTCTCG ACGCAGG--A CTCGGCTTGC TGAA------ GCGCGCGCGT CAAGAGGCGA GGGT----CG GCGA---CTG --GTGAGTAC
BF1.IT.02.58736.GU595160         CCAGAGGAG- --ATCTCTCG ACGCAGG--A CTCGGCTTGC TGAA------ GCGCGCACAG CAAGAGGCGA GGGG----CG GCGA---CTG --GTGAGTAC
BF1.IT.02.59211.GU595151         CCAGAGGAG- --CTCTCTCG ACGCAGG--A CTCGGCTTGC TGAA------ GCGCGCACAG CAAGAGGCGA GGGG----CG GCGA---CTG --GTGAGTAC
BF1.IT.05.83166.GU595152         CCGGAGGAG- --CTCTCTCG ACGCAGG--A CTCGGCTTGC TGAA------ GCGCGCTCGG CAAGAGGCGA GGGG----CG GCGA---CTG --GTGAGTAC
BF1.IT.06.89072.GU595153         CCAGAGGAG- --CTCTCTCG ACGCAGG--A CTCGGCTTGC TGAA------ GCGCGCACGG CAAGAGGCGA GGGG----CG GCGA---CCG --GTGAGTAC
BF1.JP.04.DR6082.AB480298        CCAGAGGAG- --ATCTCTCG ACGCAGG--A CTCGGCTTGC TGAA------ GTGCACTCGG CAGGAGGCGA GAG-----CG GCGG---CTG --GTGAGTAC
BF1.JP.04.DR6190.AB480300        CCAGAGGAG- --ATCTCTCG ACGCAGG--A CTCGGCTTGC TGAA------ GTGCACACGG CAAGAGGCGA GAG-----CG GCGG---CTG --GTGAGTAC
BF1.JP.x.DR0769.AB253430         CCAGAGAAG- --CTCTCTCG ACGCAGG--A CTCGGCTTGC TGAA------ GCGCGCACAG CAAGAGGCGA GGGG----CG GCGA---CTG --GTGAGTAC
BFG.MO.05.MO108.GU207884         CCAGAGAAG- --ATCTCTCG ACGCAGG--A CTCGGCTTGC TGAA------ GTGCACACGG CAAGAGGCGA GAG-----CG GCGA---CTG --GTGAGTAC
BG.CU.x.Cu100.AY586546           CCAGAGAAG- --TTCTCTCG ACGCAGG--A CTCGGCTTGC TGAG------ GTGCACACAG CAAGAGGCGA GAG-----CG GCGA---CTG --GTGAGTAC
BG.DE.01.9196_01.AY882421        CCAGAGAAG- --TTCTCTCG ACGCAGG--A CTCGGCTTGC TGGG------ -TGCACACAG CAAGAGGCGA GAG-----CG GCGA---CTG --GTGAGTAC
CD.KE.01.ML1076.EU110086         CCAGAGGAG- --ATCTCTCG ACGCAGG--A CTCGGCTTGC TGAA------ GTGCACTCGG CAAGAGGCGA GAG-----CG GCGG---CTG --GTGAGTAC
CU.JP.04.DR5782.AB286849         CCAGAGGAG- --CTCTCTCG ACGCAGG--A CTCGGCTTGC TGAA-----A GTGCACTCGG CAAGAGGCGA GAG-----CG GCGA---CTG --GTGAGTAC
DF1G.ES.04.X963_4.FJ670527       CCAGAGGAG- --ATCTCTCG ACGCAGG--A CTCGGCTTGC TGCA------ GCGCGCACAG CAAGAGGCGA GGGG----CA GCAG---CTG --GTGAGTAC
DO.FR.08.RBF208.GQ351296         CCAGGGAAGA A-AACCTCCG ACGCAACGGG CTCGGCTTAG CGGA------ GTGCACCCGC TAAGAGGCGA GAGG-----A CTCACAGAAG GGGTGAGTAA
```

```
                                  SD-
                                  SDa-                                                               -----U5/AUG-----
                                  ....|....| ....|....| ....|....| ....|....| ....|....| ....|....| ....|....| ....|....| ..
                                       305        315        325        335        345        355        365        375
B.FR.83.HXB2_LAI_IIIB_BRU.K034    GCCAAAA--- ---------- ---------- ---ATTTTGA CTAGCGGAGG CTAGAA-GGA GAGAG--ATG GGTGCGAGAG CG
A1.CD.02.02CD_KTB035.AM000055     GCCTAAA--- ---------- ---------- --ATTTTTGA CTAGCAGAGG CTAGAA-GGA GAGAG--ATG GGTGCGAGAG CG
A1.CD.97.97CD_KCC2.AM000053       GCCTAAA--- ---------- ---------- ---ATTCTGA CTAGCGGAGG CTAGAA-GGA GAGAG--ATG GGTGCGAGAG CG
A1.ES.05.X1608_8.FJ670519         GCCA------ ---------- ---------- ---TTTTTGA CTAGCGGAGG CTAGAA-GGA GAGAG--ATG GGTGCGAGAG CG
A1.IT.02.60000.EU861977           GCCTAAA--- ---------- ---------- ---GATTTGA CTAGCGGAGG CTAGAA-GGA GAGAG--ATG GGTGCGAGAG CG
A1.KE.01.ML1901PCR2.EU110095      GCCA------ ---------- ---------- --TTTTTTGA CTAGCGGAGG CTAGAA-GGA GAGAG--ATG GGTGCGAGAG CG
A1.KE.01.ML1945.EU110088          GCCAA----- ---------- ---------- ---TTTTTGA CTAGCGGAGG CTAGAA-GGA GAGAG--ATG GGTGCGAGAG CG
A1.KE.02.ML1990.EU110092          GCCAAAA--- ---------- ---------- ---TTTTTGA CTAGCGGAGG CTAGAA-GGA GAGAG--ATG GGTGCGAGAG CG
A1.KE.02.ML2014.EU110094          GCCAA----- ---------- ---------- ---TTTTTGA CTAGCGGAGG CTAGAA-GGA GAGAG--ATG GGTGCGAGAG CG
A1.KE.86.ML170_1986.AF539405      GCCTA----- ---------- ---------- ---TTTTTGA CTAGCGGAGG CTAGAA-GGA GAGAG--ATG GGTGCGAGAG CG
A1.KE.94.Q23_17.AF004885          GCCAAT---- ---------- ---------- ---TTTTTGA CTAGCGGAGG CTAGAA-GGA GAGAG--ATG GGTGCGAGAG CG
A1.KE.97.ML013_2.AY322185         GCCAAAA--- ---------- ---------- -ATTTTTTGA CTAGCGGAGG CTAGAA-GGA GAGAG--ATG GGTGCGAGAG CG
A1.KE.97.ML605_3.AY322190         GCCA------ ---------- ---------- --TTTTTTGA CTAGCGGAGG CTAAAA-GGA GAAAG--ATG GGTCCGAGAG CG
A1.KE.97.ML752.AY322193           GCCA------ ---------- ---------- --TTTTTTGA CTAGCGGAGG CTAGAA-GGA GAGAG--ATG GGTGCGAGAG CG
A1.RU.00.RU00051.EF545108         GCCTAAA--- ---------- ---------- -GATTTTTGA CTAGCGGAGG CTAGAA-GGA GAGAG--ATG GGTGCGAGAG CG
A1.RU.05.RU_560_1125_JA.JQ2928    CCCTAAG--- ---------- ---------- --ATTTTTGA CTAGCGGAGG CTAGAA-GGA GAGAG--ATG GGTGCGAGAG CG
A1.RU.06.RU_915_1016.JQ292896     GCCTAAA--- ---------- ---------- -GATTTTTGA CTAGCGGAGG CTAGAA-GGA GAGAG--ATG GGTGCGAGAG CG
A1.RU.06.RU_915_1035.JQ292897     GCCTAAA--- ---------- ---------- -GATTTTTGA CTAGCGGAGG CTAGAA-GGA GAGAG--ATG GGTGCGAGAG CG
A1.RU.06.RU_915_1038.JQ292898     GCCTAAA--- ---------- ---------- -GATTATTGA CTAGCGGAGG CTAGAA-GGA GAGAG--ATG GGTGCGAGAG CG
A1.RU.06.RU_915_1041.JQ292899     GCCTAAA--- ---------- ---------- -GATTTTTGA CTAGCGGAGG CTAGAA-GGA GAGAG--ATG GGTGCGAGAG CG
A1.RU.06.RU_SP_B_049.JQ292900     GCCTAAA--- ---------- ---------- -GATTTTTGA CTAGCGGAGG CTAGAA-GGA GAGAG--ATG GGTGCGAGAG CG
A1.RU.07.Irkutsk_5.JQ292891       CCCTAAA--- ---------- ---------- --ATTTTTGA CTAGCGGAGG CTAGAA-GGA GAGAG--ATG GGTGCGAGAG CG
A1.RU.08.PokA1Ru.FJ864679         GCCA------ ---------- ---------- --TTTTTTGA CTAGCGGAGG CTAGAA-GGA GAGAG--ATG GGTGCGAGAG CG
A1.RU.08.RUA001.JQ292893          GCCCAAA--- ---------- ---------- ---ATTTTGA CTAGCGGAGG CTAGAA-GGA GAGAG--ATG GGTGCGAGAG CG
A1.RU.08.RUA007.JQ292894          GCCTAAG--- ---------- ---------- -ATTTTTTGA CTAGCGGAGG CTAGAA-GGA GAGAG--ATG GGTGCGAGAG CG
A1.RU.10.10RU6617.JX500696        GCCTAAA--- ---------- ---------- --ATTTTTGA CTAGCGGAGG CTAGAA-GGA GAGAG--ATG GGTGCGAGAG CG
A1.RU.10.10RU6792.JX500695        GCCTAAG--- ---------- ---------- --ATTTTTGA CTAGCGGAGG CTAGAA-GGA GAGAG--ATG GGTGCGAGAG CG
A1.RU.11.11RU6950.JX500694        GCCTAAA--- ---------- ---------- -GATTTTTGA CTAGCGGAGG CTAGAA-GGA GAGAG--ATG GGTGCGAGAG CG
A1.RW.07.pR463F.JX236677          GCCTAAG--- ---------- ---------- AATTTTTTGA CTAGCGGAGG CTAGAA-GGA GAGAG--ATG GGTGCGAGAG CG
A1.RW.07.pR880F.JX236678          GCCC------ ---------- ---------- --TTTTTTGA CTAGCGGAGG CTAGAA-GGA GAGAAG-ATG GGTGCGAGAG CG
A1.RW.92.92RW008.AB253421         GCCA------ ---------- ---------- --TTTTTTGA CTAGCGGAGG CTAGAA-GGA GAGAG--ATG GGTGCGAGAG CG
A1.RW.92.92RW025A.AB287376        GCCAA----- ---------- ---------- ---TTTTTGA CTAGCGGAGG CTAGAA-GGA GAGAG--ATG GGTGCGAGAG CG
A1.RW.93.93RW037A.AB287379        GCCTAAG--- ---------- ---------- AATTTTTTGA CTAGCGGAGG CTAGAA-GGA GAGAG--ATG GGTGCGAGAG CG
A1.SE.95.UGSE8131.AF107771        GCCA------ ---------- ---------- --TTTTTTGA CTAGCGGAGG CTAGAA-GGA GAGAG--ATG GGTGCGAGAG CG
A1.TZ.97.97TZ03.AF361873          GCCA------ ---------- ---------- ---TTTTTGA CTAGCGGAGG CTAGAA-GGA GAGAG--ATG GGTACGAGAG CG
A1.UA.00.98UA0116.AF413987        GCCTAAG--- ---------- ---------- -GATTTTTGA CTAGCGGAGG CTAGAA-GGA GAGAG--ATG GGTGCGAGAG CG
A1.UG.07.p191084.JX236669         GCCAA----- ---------- ---------- --TTTTTTGA CTAGCGGAGG CTAGAA-GGA GAGAG--ATG GGTGCGAGAG CG
A1.UG.07.p191845.JX236671         GCCAA----- ---------- ---------- ---TTTTTAA CTAGCGGAGG CTAGAA-GGA GAGAG--ATG GGTGCGAGAG CG
A1.UG.07.p9004SDM.JX236676        GCCT------ ---------- ---------- ---TTTTTGA CTAGCGGAGG CTAGAA-GGA GAGAG--ATG GGTGCGAGAG CG
A1.UG.85.U455_U455A.M62320        GCCTAAA--- ---------- ---------- -ATTTTTTGA CTAGCGGAGG CTAGAA-GGA GAGAA--ATG GGTGCGAGAG CG
A1.UG.92.92UG037.AB253429         GCCA------ ---------- ---------- --TTTTTTGA CTAGCGGAGG CTAGAA-GGA GAGAG--ATG GGTGCGAGAG CG
A1.UG.92.UG029.AB098333           GCCAAAA--- ---------- ---------- AATTTTTTGA CTAGCGGAGG CTAGAA-GGA GAGAG--ATG GGTGCGAGAG CG
A1.UG.x.UG031.AB098331            GCCAA----- ---------- ---------- ---TTTTTGA CTAGCGGAGG CTAGAA-GGA GAGAG--ATG GGTGCGAGAG CG
A1.UG.x.UG275.AB485632            GCCAAGA--- ---------- ---------- ATTTTTTTGA CTAGCGGAGG CTAGAA-GGA GAGAG--ATG GGTGCGAGAG CG
A1.ZA.04.04ZASK162B1.DQ396400     GCCGGGA--- ---------- ---------- AATTTTTTGA CTAGCGGAGG CTAGAA-GGA GAGAG--ATG GGTGCGAGAG CG
A2.CD.97.97CDKTB48.AF286238       GCCTAAA--- ---------- ---------- --ATTTTTGA CTAGCGGAGG CTAGAA-GGA GAGAG--ATG GGTGCGAGAG CG
A2.CY.94.94CY017_41.AF286237      GCCTAAT--- ---------- ---------- --ATTTTTGA CTAGCAGAGG CTAGAA-GGA GAGAG--ATG GGTGCGAGAG CG
B.AU.86.MBC200.AF042100           GCCAAAA--- ---------- ---------- -AATTTTTGA CTAGCGGAGG CTAGAG-GAG GAGAG--ATG GGTGCGAGAG CG
B.AU.87.MBC925.AF042101           GCCAAAA--- ---------- ---------- --AATTTTGA CTAGCGGAGG CTAGAA-GGA GAGAG--ATG GGTGCGAGAG CG
B.AU.95.C24.AF538304              GCCAAAT--- ---------- ---------- ---ATTTTGA CTAGCGGAGG CTATAA-GGA GAGAG--ATG GGTGCGAGAG CG
B.AU.95.C42.AF538305              GCCAAAA--- ---------- ---------- ---ATTTTGA CTAGCGGAGG CTAGAA-GGA GAGAG--ATG GGTGCGAGAG CG
B.AU.95.C76.AF538306              ACCAAAA--- ---------- ---------- ---ATTTTGA CTAGCGGAGG CTAGAA-GGA GAGAG--ATG GGTGCGAGAG CG
B.AU.95.C92.AF538307              GCCAAAA--- ---------- ---------- ---ATTTTGA CTAGCGGAGG CTAGAA-GGA GAGAG--ATG GGTGCGAGAG CG
B.AU.95.MBCC54.AF042103           GCCAAAA--- ---------- ---------- ---TTTTTGA CTAGCGGAGG CTAGAA-GGA GAGAG--ATG GGTGCGAGAG CG
B.AU.96.MBCC98.AF042104           GCCAAAA--- ---------- ---------- ---ATTTTGA CTAGCGGAGG CTAGAA-GGA GAGAG--ATG GGTGCGAGAG CG
B.AU.96.MBCD36.AF042105           GCCAAAA--- ---------- ---------- ---ATTTTGA CTAGCGGAGG CTAGAA-GGA GAAAG--ATG GGTGCGAGAG CG
B.AU.99.1181.AF538302             GCCAAAA--- ---------- ---------- --TTTTTTGA CTAGCGGATG CTAGAA-GAA AAAAG--ATG GGTGCGAGAG CG
B.AU.x.2870718.AY857022           GCCGAAA--- ---------- ---------- ---ATTTTGA CTAGCGGAGG CTAGAA-GGA GAGAG--ATG GGTGCGAGAG CG
B.AU.x.9125091.AY857165           GCCAAAA--- ---------- ---------- ---TTTTTGA CTAGCGGAGG CTAGAA-GGA GAGAG--ATG GGTGCGAGAG CG
B.AU.x.VH_VHPCR.AF146728          GCCAAAA--- ---------- ---------- ---ATTTTGA CTAGCGGAGG CTAGAA-GGA GAGAG--ATG GGTGCGAGAG CG
B.BO.09.DEMB09BO001.JX140656      GCCAAAA--- ---------- ---------- AATTTTTTGA CTAGCGGAGG CTAGAA-GGA GAGAG--ATG GGTGCGAGAG CG
B.BR.02.02BR008.DQ358808          GCCA------ ---------- ---------- --TTTTTTGA CTAGCGGAGG CTAGAA-GGA GAGAG--ATG GGTGCGAGAG CG
B.BR.02.02BR011.DQ358809          GCCAAAC--- ---------- --------AT AAAATTTTGA CTAGCGGAGG CTAGAA-GGA GAGAG--ATG GGTGCGAGAG CG
B.BR.02.02BR013.DQ358810          GCCAAAA--- ---------- --------AT AAAATTTTGA CTAGCGGAGG CTAGAA-GGA GAGAG--ATG GGTGCGAGAG CG
B.BR.02.02BR1013.JN692432         GCCAAAA--- ---------- -------GAA AAATTTTTGA CTAGCGGAGG CTAGAA-GGA GAGAG--ATG GGTGCGAGAG CG
B.BR.02.02BR2025.JN692435         GCCAAT---- ---------- ---------- ---TTTTTGA CTAGCGGAGG CTAGAA-GGA GAGAG--ATG GGTGCGAGAG CG
B.BR.02.02BR2032.JN692439         GCCTAAA--- ---------- ---------- ---ATTTTGA CTAGCGGAGG CTAGAA-GGA GAGAG--ATG GGTGCGAGAG CG
B.BR.02.02BR2033.JN692440         GCCAAAA--- ---------- ---------- -TATTTTTGA CTAGCGGAGG CTAGAA-GGA GAGAG--ATG GGTGCGAGAG CG
B.BR.02.02BR2041.JN692443         GCCAAAA--- ---------- ---------- ---TTTTTGA CTAGCGGAGG CTAGAA-GGA GAGAG--ATG GGTGCGAGAG CG
B.BR.02.02BR2042.JN692444         GCCAAAA--- ---------- ---------- ---TTTTTGA CTAGCGGAGG CTAGAA-GGA GAGAG--ATG GGTGCGAGAG CG
B.BR.02.04BR1064.JN692433         GCCAAAA--- ---------- ---------- ---TTTTTGA CTAGCGGAGG CTAGAA-GGA GAGAG--ATG GGTGCGAGAG CG
B.BR.02.05BR1094.JN692470         GCCAAAA--- ---------- ---------- ---ATTTTGA CTAGCGGAGG CTAGAA-GGA GAGAG--ATG GGTGCGAGAG CG
B.BR.03.03BR1020.JN692445         GCCAAAA--- ---------- ---------- ---TTTTTGA CTAGCGGAGG CTAGAA-GGA GAGAG--ATG GGTGCGAGAG CG
B.BR.03.03BR1046.JN692447         GCCAAAT--- ---------- ---------- ---ATTTTGA CTAGCGGAGG CTAGAA-GGA GAGAG--ATG GGTGCGAGAG CG
B.BR.03.BREPM1023.EF637057        GCCAAAA--- ---------- ---------- ---TTTTTGA CTAGCGGAGG CTAGAA-GGA GAGAG--ATG GGTGCGAGAG CG
B.BR.03.BREPM1024.EF637056        GCCAAAA--- ---------- ---------- -ATTTTGACT TAACCGGAGG CTAGAA-GGA GAGAG--ATG GGTGCGAGAG CG
B.BR.03.BREPM1027.EF637054        GCCA------ ---------- ---------- ---ATTTTGA CTAGCGGAGG CTAGAA-GGA GAGAG--ATG GGTGCGAGAG CG
B.BR.03.BREPM1028.EF637053        GCCAA----- ---------- ---------- ---ATTTTGA CTAGCGGAGG CTAGAA-GGA GAGAG--ATG GGTGCGAGAG CG
B.BR.03.BREPM1032.EF637051        GCCAAAA--- ---------- ---------- -AATTTTTGA CTAGCGGAGG CTAGAA-GGA GAGAG--ATG GGTGCGAGAG CG
B.BR.03.BREPM1033.EF637050        GCCA------ ---------- ---------- --TTTTTTGA CTAGCGGAGG CTATAA-GGA GAGAG--ATG GGTGCGAGAG CG
B.BR.03.BREPM1035.EF637049        GCCA------ ---------- --------AT TTTATTTTGA CTAGCGGAGG CTAGAA-GGA GAGAG--ATG GGTGCGAGAG CG
B.BR.03.BREPM1038.EF637048        GCCAAA---- ---------- ---------- ---TTTTTGA CTAGCGGAGG CTAGAA-GGA GAGAG--ATG GGTGCGAGAG CG
B.BR.03.BREPM1040.EF637047        GCCAA----- ---------- ---------- ---TTTTTGA CTAGCGGAGG CTAGAA-GGA GAGAG--ATG GGTGCGAGAG CG
B.BR.03.BREPM2012.EF637046        GCCATAA--- ---------- ---------- --AATTTTGA CTAGCGGAGG CTAGAA-GGA GAGAG--ATG GGTGCGAGAG CG
B.BR.04.04BR1047.JN692450         GCCAA----- ---------- ---------- ---TTTTTGA CTAGCGGAGG ATAGAA-GGA GAGAG--ATG GGTGCGAGAG CG
B.BR.04.04BR1049.JN692451         GCCAAAA--- ---------- ---------- ---ATTTTGA CTAGCAGAGG CTAGAA-GGA GAGAG--ATG GGTGCGAGAG CG
B.BR.04.04BR1051.JN692452         GCCAAAA--- ---------- --------AT AAAATTTTGA CTAGCGGAGG CTAGAA-GGA GAGAG--ATG GGTGCGAGAG CG
B.BR.04.04BR1054.JN692453         GCCAAAA--- ---------- ------TTAA AATTTTTTGA CTAGCGGAGG CTAGAA-GGA GAGAG--ATG GGTGCGAGAG CG
B.BR.04.04BR1055.JN692454         GCCAAAA--- ---------- --------AT AAAGTTTTGA CTAGCGGAGG CTAGAA-GGA AAGAG--ATG GGTGCGAGAG CG
B.BR.04.04BR1057.JN692455         GCCA------ ---------- ---------- ---ATTTTGA CTAGCGGAGG CTAGAA-GGA GAGAG--ATG GGTGCGAGAG CG
B.BR.04.04BR1068.JN692457         GCCA------ ---------- ---------- --TATTTTGA CTAGCGGAGG CTAGAA-GGA GAGAG--ATG GGTGCGAGAG CG
B.BR.04.BREPM1066.FJ195090        GCCTAAA--- ---------- ---------- ---ATTTTGA CTAGCGGAGG CTAGAA-GGA GAGAG--ATG GGTGCGAGAG CG
B.BR.04.BREPM1070.FJ195086        CCCAAAA--- ---------- ---------- ---ATTTTGA CTAGCGGAGG CTAGAA-GGA GAGAG--ATG GGTGCGAGAG CG
B.BR.05.05BR1077.JN692460         GCCAAAA--- ---------- --------AT AAAATTTTGA CTAGCGGAGG CTAGAA-GGA GAGAG--ATG GGTGCGAGAG CG
B.BR.05.05BR1078.JN692461         GCCAAAA--- ---------- ---------- ATAATTTTGA CTAGCGGAGG CTAGAA-GGA GAGAG--ATG GGTGCGAGAG CG
B.BR.05.05BR1079.JN692462         GCCAAAA--- ---------- ---------- -AATTTTTGA CTAGCGGAGG CTAGAA-GGA GAGAG--ATG GGTGCGAGAG CG
B.BR.05.05BR1080.JN692463         GCCAAAA--- ---------- ---------- ---ATTTTGA CTAGCGGAGG CTAGGA-GGA GAGAG--ATG GGTGCGAGAG CG
B.BR.05.05BR1082.JN692465         GCCAAAA--- ---------- ---------- ---TTTTTGA CTAGCGGAGG CTAGGA-GGA GAGAG--ATG GGTGCGAGAG CG
B.BR.05.05BR1089.JN692467         GCCAA----- ---------- ---------- ---TTTTTGA CTAGCGGAGG CTAGAA-GGA GAGAG--ATG GGTGCGAGAG CG
B.BR.05.05BR1092.JN692468         GCCAAAA--- ---------- --------AT AAAATTTTGA CTAGCGGAGG CTAGAA-GGA GAGAG--ATG GGTGCGAGAG CG
B.BR.05.05BR1095.JN692471         GCCA------ ---------- ---------T TTTATTTTGA CTAGCGGAGG CTAGAA-GGA GAGAG--ATG GGTGCGAGAG CG
```

```
B.BR.05.05BR1101.JN692473        GCCAAAA--- ---------- ---------- ---TTTTTGA CTAGCGGAGG CTAGAA-GGA GAGAG--ATG GGTGCGAGAG CG
B.BR.05.05BR1104.JN692474        GCCAAAA--- ---------- ---------- ---ATTTTGG CTGGCGGAGG CTAGAA-GGA GAGAG--ATG GGTGCGAGAG CG
B.BR.05.05BR1107.JN692475        GCCAAAC--- ---------- ---------- ------TTGA CTAGCGGAGG CTAGAA-GGA GAGAG--ATG GGTGCGAGAG CG
B.BR.05.BREPM1081.FJ195091       GCCAAAA--- ---------- -----AATTA AAAATTTTGA CTAGCGGAGG CTAGAA-GGA GAGAG--ATG GGTGCGAGAG CG
B.BR.05.BREPM1084.FJ195088       GCCAAAA--- ---------- ------ATAA AATTTTTTGA CTAGCGGAGG CTAGAA-GGA GAGGG--ATG GGTGCGAGAG CG
B.BR.05.BREPM1093.FJ195089       GCCAAAA--- ---------- ---------- ---ATTTTGA CTAGCGGAGG CTAGAA-GGA GAGAG--ATG GGTGCGAGAG CG
B.BR.06.06BR1115.JN692479        GCCAAAC--- ---------- ---------- -----TTTGA CTAGCGGAGG CTAGAA-GGA GAGAG--ATG GGTGCGAGAG CG
B.BR.06.06BR1119.JN692480        GCCAAAA--- ---------- ------ATAA AGTTTTTGAA TAAGGGAAGG TTAAAA-GGA GAGAG--ATG GTTGCGAGAG CG
B.CA.06.502_1027_wg01.JF320413   GCCAAAA--- ---------- ---------- -AATTTTTGA CTAGCGGAGG CTAGAA-GGA GAGAG--ATG GGTGCGAGAG CG
B.CA.06.502_1799_FL02.JF320427   GCCGAAA--- ---------- ---------- ---ATTTTGA CTAGCGGAGG CTAGAA-GGA GAGAG--ATG GGTGCGAGAG CG
B.CA.07.502_1191_03.JF320424     GCCAAAA--- ---------- ---------- -TTTTTGACT AGCGGAGGCT AGAAGG-AGA GAGAG--ATG GGTGCGAGAG CG
B.CA.96.WC10C_4.AY314056         GCCAAA---- ---------- ---------- ---TTTTTGA CTAGCGGAGG CTAGAA-GGA GAGAG--ATG GGTGCGAGAG CG
B.CA.97.CANB3FULL.AY779553       GCCAAAA--- ---------- ---------- ---ATTTTGA CTAGCGGAGG CTAGAA-GGA GAGAG--ATG GGTGCGAGAG CG
B.CH.03.HIV_CH_BID_V4470_2003.   GCCAAAA--- ---------- ---------- ---TTTTTGA CTAGCGGAGG CTAGAA-GGA GAGAGAGATG GGTGCGAGAG CG
B.CN.01.CNHN24.AY180905          GCCAAAT--- ---------- ---------- ---TTTTTGA CTAGCGGAGG CTAGAA-GGA GAGAG--ATG GGTGCGAGAG CG
B.CN.02.02HNsc11.DQ007903        GCCAAAA--- ---------- ---------- AATTTTGTGA CTAGCGGAGG CTAGAA-GGA GAGAG--ATG GGTGCGAGAG CG
B.CN.03.SHXDC0081.JF932492       GCCGAAA--- ---------- ---------- --ATTTTTGA CTAGCGGAGG CTAGAA-GGA GAGAG--ATG GGTGCGAGAG CG
B.CN.05.05CNHB_hp3.DQ990880      GCCA------ ---------- ---------- --TTTTTTGA CTAGCGGAGG CTAGAA-GGA GAGAG--ATG GGTGCGAGAG CG
B.CN.06.CC056.JF932482           GCCAAAT--- ---------- ---------- ---TTTTTGA CTAGCGGAGG CTAGAA-GGA GAGAG--ATG GGTGCGAGAG CG
B.CN.07.AH070011.JF932468        GCCAAAA--- ---------- ---------- --CTTTTTGA CTAGCGGAGG CTAGAA-GGA GAGAG--ATG GGTGCGAGAG CG
B.CN.07.AH070014.JF932469        GCCGAAA--- ---------- ---------- ---ATTTTGA CTAGCGGAGG CTAGAA-GGA GAGAG--ATG GGTGCGAGAG CG
B.CN.07.AH070017.JF932470        GCCA------ ---------- ---------- --TTTTTTGA CTAGCGGAGG CTAGAA-GGA GAGAG--ATG GGTGCGAGAG CG
B.CN.07.AH070018.JF932471        GCCAAAA--- ---------- ---------- AATTTTTTGA CTAGCGGAGG CTAGAA-GGA GAGAG--ATG GGTGCGAGAG CG
B.CN.07.AH070057.JF932472        GCCAAAA--- ---------- ---------- AATTTTTTGA CTAGCGGAGG CTAGAA-GGA GAGAG--ATG GGTGCGAGAG CG
B.CN.07.BJ070030.JF932473        GCCAAAA--- ---------- ---------- -TATTTTTGA CTAGCGGAGG CTAGAA-GGA GAGAG--ATG GGTGCGAGAG CG
B.CN.07.CBJC261.JF932474         GCCGAAA--- ---------- ---------- --ATTTTTGA CTAGCGGAGG CTAGAA-GGA GAGAG--ATG GGTGCGAGAG CG
B.CN.07.CBJC392.JF932475         GCCGAAA--- ---------- ---------- ---TTTTTGA CTAGCGGAGG CTAGAA-GGA GAGAG--ATG GGTGCGAGAG CG
B.CN.07.CBJC394.JF932476         GCCAAAA--- ---------- ---------- ---TTTTTGA CTAGCGGAGG CTAGAA-GGA GAGAG--ATG GGTGCGAGAG CG
B.CN.07.CBJC396.JF932477         GCCAAAT--- ---------- ---------- ----TTTTGA CTAGCGGAGG CTAGAA-GGA GAGAG--ATG GGTGCGAGAG CG
B.CN.07.GS070017.JF932484        GCCA------ ---------- ---------- TTTTTTTTGA CTAGCGGAGG CTAGAA-GGA GAGAG--ATG GGTGCGAGAG CG
B.CN.07.GZ070002.JF932485        GCCAAAA--- ---------- ---------- ---TTTTTGA CTAGCGGAGG CTAGAA-GGA GAGAG--ATG GGTGCGAGAG CG
B.CN.07.GZ070030.JF932486        GCCGAAA--- ---------- ---------- --ATTTTTGA CTAGCGGAGG CTAGAA-GGA GAGAG--ATG GGTGCGAGAG CG
B.CN.07.HB070006.JF932487        GCCATAA--- ---------- ---------- ---ATTTTGA CTAGCGGAGG CTAGAA-GGA GAGAG--ATG GGTGCGAGAG CG
B.CN.07.HB070022.JF932488        GCCGAAA--- ---------- ---------- --ATTTTTGA CTAGCGGAGG CTAGAA-GGA GAGAG--ATG GGTGCGAGAG CG
B.CN.07.HB070035.JF932489        GCCAAAA--- ---------- ---------- AATTTTTTGA CTAGCGGAGG CTAGAA-GGA GAGAG--ATG GGTGCGAGAG CG
B.CN.07.JL070038.JF932490        GCCGAAA--- ---------- ---------- -ACTTTTTGA CTAGCGGAGG CTAGAA-GGA GAGAG--ATG GGTGCGAGAG CG
B.CN.07.SX070080.JF932493        GCCAAAA--- ---------- ---------- -ACTTTTTGA CTAGCGGAGG CTAGAA-GGA GAGAG--ATG GGTGCGAGAG CG
B.CN.07.hb070025.JF932499        GCCGA----- ---------- ---------- ---TTTTTGA CTAGCAGAGG CTAGAA-GGA GAGAG--ATG GGTGCGAGAG CG
B.CN.07.hen1345.JF932500         GCCAAAT--- ---------- ---------- ----TTTTGA CTAGCGGAGG CTAGAA-GGA GAGAG--ATG GGTGCGAGAG CG
B.CN.08.CBJC476.JF932478         GCCGAAA--- ---------- ---------- --ATTTTTGA CTAGCGGAGG CTAGAA-GGA GAGAG--ATG GGTGCGAGAG CG
B.CN.08.CBJC489.JF932479         GCCAAAA--- ---------- ---------- -AATTTTTGA CTAGCGGAGG CTAGAA-GGA GAGAG--ATG GGTGCGAGAG CG
B.CN.08.CBJC500.JF932480         GCCGAAA--- ---------- -------AAA TTTTTTTTGA CTAGCGGAGG CTAGAA-GGA GAGAG--ATG GGTGCGAGAG CG
B.CN.08.CBJC503.JF932481         GCCAAAA--- ---------- ---------- -AATTTTTGA CTAGCGGAGG CTAGAA-GGA GAGAG--ATG GGTGCGAGAG CG
B.CN.08.cbjc468.JF932498         GCCAAAA--- ---------- ---------- --AATTTTGA CTAGCGGAGG CTAGAA-GGA GAGAG--ATG GGTGCGAGAG CG
B.CN.09.YN09P0014.JF932494       GCCAAAA--- ---------- ---------- AATTTTTTGA CTAGCGGAGG CTAGAA-GGA GAGAG--ATG GGTGCGAGAG CG
B.CN.09.ZK042.JF932497           GCCAAAA--- ---------- ---------A ATTTTTTTGA CTAGCGGAGG CTAGAA-GGA GAGAG--ATG GGTGCGAGAG CG
B.CN.10.DEMB10CN002.JX140658     GCCAAAA--- ---------- ---------- ----CTTTGA CTAGCGGAGG CTAGAA-GGA GAGAG--ATG GGTGCGAGAG CG
B.CN.98.YN9802.JF932495          GCCAAAA--- ---------- ---------- ---ATTTTGA CTAGCGGAGG CTAGAA-GGA GAGAG--ATG GGTGCGAGAG CG
B.CN.98.YN9838.JF932496          GCCATAA--- ---------- ---------- ---ATTTTGA CTAGCGGAGG CTAGAA-GGA GAGAG--ATG GGTGCGAGAG CG
B.CN.99.plwj.GU177863            GCCAA----- ---------- ---------- ---ATTTTGA CTAGCGGAGG CTAGAA-GGA GAGAG--ATG GGTGCGAGAG CG
B.CN.x.RL42.U71182               GCCAAT---- ---------- ---------- ------TTGA CTAGCGGAGG CTAGAA-GGA GAGAG--ATG GGCGCGAGAG CG
B.CU.99.Cu19.AY586542            GCCAAAA--- ---------- ---------- ---TTTTTGA CTAGCGGAGG CTAGAA-GGA GAGAG--ATG GGTGCGAGAG CG
B.CU.99.Cu43.AY586543            GCCAAAA--- ---------- ---------- ---TTTTTGA CTAGCGGAGG CTAGAA-GGA GAGAG--ATG GGTGCGAGAG CG
B.DE.86.D31.U43096               GCCAAAA--- ---------- ---------- ---ATTTTGA CTAGCGGAGG CTAGAA-GGA GAGAG--ATG GGTGCGAGAG CG
B.DE.86.HAN.U43141               GCCAAAA--- ---------- ---------- ----TTTTGA CTAGCGGAGG CTAGAA-GGA GAGAG--ATG GGTGCGAGAG CG
B.DK.01.CTL_016.EF514704         GCCGAAT--- ---------- ---------- ---TTGTTGA CTAGCGGAGG CTAGAA-GGA GAGAG--ATG GGTGCGAGAG CG
B.DK.01.CTL_017.EF514705         GCCGATA--- ---------- ---------- ----TTTTGA CTAGCGGAGG CTAGAA-GGA GAGAG--ATG GGTGCGAGAG CG
B.DK.01.CTL_018.EF514706         GCCA------ ---------- ---------- ---ATTTTGA CTAGCGGAGG CTAGAA-GGA GAGAG--ATG GGTGCGAGAG CG
B.DK.01.CTL_023.EF514707         GCCAAAA--- ---------- ---------- ---ATTTTGA CTAGCGGAGG CTAGAA-GGA GAGAG--ATG GGTGCGAGAG CG
B.DK.01.CTL_030.EF514708         GCCAAAA--- ---------- ---------- ---ATTTTGA CTAGCGGAGG CTAGAA-GGA GAGAG--ATG GGTGCGAGAG CG
B.DK.01.CTL_033.EF514709         GCCAAGA--- ---------- ---------- ---TTTTTGA CTAGCGGAGG CTAGAA-GGA GAGAG--ATG GGTGCGAGAG CG
B.DK.01.CTL_035.EF514710         GCCAA----- ---------- ---------- ---TTTTTGA CTAGCGGAGG CTAGAA-GGA GAGAG--ATG GGTGCGAGAG CG
B.DK.01.CTL_041.EF514711         GCCAAAT--- ---------- ---------- -----TTTTG ACTAGCGGAG CTAGAA-GGA GAGAG--ATG GGTGCGAGAG CG
B.DK.01.CTL_043.EF514712         GCCAAAA--- ---------- ---------- ---TTTTTGA CTAGCGGAGG CTAGGA-GGA GAGAG--ATG GGTGCGAGAG CG
B.DK.04.PMVL_012.EF514699        GCCAATA--- ---------- ---------- ---ATTTTGA CTAGCGGAGG CTAGAA-GGA GAGAG--ATG GGTGCGAGAG CG
B.DK.04.PMVL_013.EF514700        GCCAA----- ---------- ---------- ---ACTTTGA CTAGCGGAGG CTAGAA-GGA GAGAG--ATG GGTGCGAGAG CG
B.DK.04.PMVL_018.EF514697        GCCAAAA--- ---------- ---------- ---TTTTTGA CTAGCGGAGG CTAGAA-GGA GAGAG--ATG GGTGCGAGAG CG
B.DK.04.PMVL_025.EF514702        GCCATAA--- ---------- ---------- ---ATTTTGA CTAGCGGAGG CTAGAA-GGA GAGAG--ATG GGTGCGAGAG CG
B.DK.04.PMVL_027.EF514698        GCCAAAA--- ---------- ---------- --ATTTTTGA CTAGCGGAGG CTAGAA-GGA GAGAG--ATG GGTGCGAGAG CG
B.DK.04.PMVL_039.EF514703        TACCAAA--- ---------- ---------A AATTTTTTGA CTAGCGGAGG CTAGAA-GGA GAGAG--ATG GGTGCGAGAG CG
B.DK.04.PMVL_049.EF514701        GCCAAAA--- ---------- ---------- ---TTTTTGA CTAGCGGAGG CTAGAA-GGA GAGAG--ATG GGTGCGAGAG CG
B.DK.07.PMVL_011.FJ694790        GCCAAGA--- ---------- ---------- ---ATTTTGA CTAGCGGAGG CTAGAA-GGA GAGAG--ATG GGTGCGAGAG CG
B.ES.05.X1890.EU786672           GCCAAAA--- ---------- ---------- ---ATTTTGA CTAGCGGAGG CTAGAA-GGA GAGAG--ATG GGTGCGAGAG CG
B.ES.06.X1959.EU786675           GCCATAA--- ---------- ---------- ----TTTTGA CTAGCAGAGG CTAGAA-GGA GAGAG--ATG GGTGCGAGAG CG
B.ES.06.X2102.EU786677           GCCAAAA--- ---------- ---------- ----TTTTGA CTAGCGGAGG CTAGAA-GGA GAGAG--ATG GGTGCGAGAG CG
B.ES.07.X2149.EU786678           GCCGAAA--- ---------- ---------- ---ATTTTGA CTAGCGGAGG CTAGAA-GGA GAGAG--ATG GGTGCGAGAG CG
B.ES.07.X2210_3.EU786679         GCCAAAA--- ---------- ---------- AATTTTTTGA CTAGCGGAGG CTAGAA-GGA GAGAG--ATG GGTGCGAGAG CG
B.ES.07.X2231.EU786680           GCCGAAA--- ---------- ---------- --ATTTTTGA CTAGCGGAGG CTAGAA-GGA GAGAG--ATG GGTGCGAGAG CG
B.ES.08.ES_X2515_3.GQ372988      GCCAAAA--- ---------- ---------- -TTTTTTTGA CTAGCGGAGG CTAGAA-GGA GAGAG--ATG GGTGCGAGAG CG
B.ES.08.ES_X2556_3.GQ372990      GCCAA----- ---------- ---------- ---TTTTTGA CTAGCGGAGG CTAGAA-GGA GAGAG--ATG GGTGCGAGAG CG
B.ES.08.P2008.FJ670531           GCCTAAA--- ---------- ---------- AATTTTTTGA CTAGCGGAGG CTAGAA-GGA GAGAG--ATG GGTGCGAGAG CG
B.ES.08.X2425_2.FJ670525         GCCA------ ---------- ---------- ---TTTTTGA CTAGCGGAGG CTAGAA-GGA GAGAG--ATG GGTGCGAGAG CG
B.ES.08.X2510_2.FJ853622         GCCAAAA--- ---------- ---------- ---TTTTTGA CTAGCGGAGG CTAGAA-GGA GAGAG--ATG GGTGCGAGAG CG
B.ES.08.X2555_2.GU362883         GCCAAAA--- ---------- ---------- ---TTTTTGA CTAGCGGAGG CTAGAA-GGA GAGAG--ATG GGTGCGAGAG CG
B.ES.08.X2574_2.GU362886         GCCAAAA--- ---------- ---------- ---TTTTTGA CTAGCGGAGG CTAGAA-GGA GAGAG--ATG GGTGCGAGAG CG
B.ES.09.P2149_3.GU362881         GCCAAAA--- ---------- ---------- -CTTTTTTGA CTAGCGGAGG CTAGAA-GGA GAGAG--ATG GGTGCGAGAG CG
B.ES.09.X2689_2.GU362885         GCCAAAA--- ---------- ---------- --TTTTTTGA CTAGCGGAGG CTAGAA-GGA GAGAG--ATG GGTGCGAGAG CG
B.ES.89.U61.DQ854716             GCCAAAA--- ---------- ---------- ----TTTTGA CTAGCGGAGG CTAGAA-GGA GAGAG--ATG GGTGCGAGAG CG
B.FR.05.DEMB05FR001.JX140652     GCCA------ ---------- ---------- ----ATTTGA CTAGCGGAGG CTAGAA-GGA GAGAG--ATG GGTGCGAGAG CG
B.FR.08.DEMB08FR002.JX140654     GCCAATT--- ---------- ---------- -TGTTTTTGA CTAGCGGAGG CTAGAA-GGA GAGAG--ATG GGTGCGAGAG CG
B.GA.88.OYI_397.M26727           GCCAAAA--- ---------- ---------- --ATTTTTGA CTAGCGGAGG CTAGAA-GGA GAGAG--ATG GGTGCGAGAG CG
B.GB.05.MM45d213_GN1.HM586212    GCCAAAA--- ---------- ---------- --ATTTTTGA CTAGCGGAGG CTAGAA-GGA GAGAG--ATG GGTGCGAGAG CG
B.GB.83.CAM1.D10112              GCCA------ ---------- ---------- TTTATTTTGA CTAGCGGAGG CTAGAA-GGA GAGAG--ATG GGTGCGAGAG CG
B.GB.86.GB8_46R.AJ271445         GCCAAAA--- ---------- ---------- ---ATTTTGA CTAGCGGAGG CTAGAA-GGA GAGAG--ATG GGTGCGAGAG CG
B.GB.x.MANC.U23487               GCCAAAA--- ---------- ---------- ---ATTTTGA CTAGCGGAGG CTAGAA-GGA GAGAG--ATG GGTGCGAGAG CG
B.HK.06.HK003.FJ460500           GCCAAAA--- ---------- ---------- ---TTTTTGA CTAGCGGAGG CTAGAA-GGA GAGAG--ATG GGTGCGAGAG CG
B.HK.06.HK004.FJ460501           GCCAAAA--- ---------- ---------- ---TTTTTGA CTAGCGGAGG CTAGAA-GGA GAGAG--ATG GGTGCGAGAG CG
B.IN.x.11807.EF694037            GCCAAAA--- ---------- ---------- ---ATTTTGA CTAGCGGAGG CTAGAA-GGA GAGAG--ATG GGTGCGAGAG CG
B.IT.05.SG1.DQ672623             GCCAAAT--- ---------- ---------- ---TTTTTGA CTAGCGGAGG CTAGAA-GGA GAGAG--ATG GGTGCGAGAG CG
B.JP.00.DR2508.AB289588          GCCAA----- ---------- ---------- ---TTTTTGA CTAGCGGAGG CTAGAA-GGA GAGAG--ATG GGTGCGAGAG CG
B.JP.01.DR388.AB289590           GCCAAA---- ---------- ---------- ---CTTTTGA CTAGCGGAGG CTAGAA-GGA GAGAG--ATG GGTGCGAGAG CG
B.JP.04.04JPDR6075B.AB221125     GCCAA----- ---------- ---------- ---TTTTTGA CTAGCGGAGG CTAGAA-GGA GAAAG--ATG GGTGCGAGAG CG
B.JP.04.DR5913.AB480696          GCCAAAT--- ---------- ---------- ---ATTTTGA CTAGCGGAGG CTAGAA-GGA GAGAG--ATG GGTGCGAGAG CG
B.JP.04.DR6089.AB286955          GCCGAAA--- ---------- ---------- ---TTTTTGA CTAGCGGAGG CTAGAA-GGA GAGAG--ATG GGTGCGAGAG CG
B.JP.04.DR6174.AB480692          GCCAAAA--- ---------- ---------- ----TTTTGA CTAGCGGAGG CTAGAA-GGA GAGAG--ATG GGTGCGAGAG CG
```

```
B.JP.04.DR6175.AB480694          GCCA------ ---------- ---------- ---TTTTTGA CTAGCGGAGG CTAGAA-GGA GAGAG--ATG GGTGCGAGAG CG
B.JP.05.DR6538.AB287363          GCCAAAA--- ---------- ---------- AATTTTTTGA CTAGCGGAGG CTAGAA-GGA GAGAG--ATG GGTGCGAGAG CG
B.JP.05.DR6737.AB287364          GCCAAAT--- ---------- ---------- ---TTTTTGA CTAGCGGAGG CTAGAA-GGA GAGAG--ATG GGTGCGAGAG CG
B.JP.05.DR7060.AB287366          GCCAAAA--- ---------- -------ATA AAATTTTTGA CTAGCGGAGG CTAGAA-GGA GAGAG--ATG GGTGCGAGAG CG
B.JP.05.DR7065.AB287368          GCCAAT---- ---------- ---------- ---TTTTTGA CTAGCGGAGG CTAGAA-GGA GAGAG--ATG GGTGCGAGAG CG
B.JP.98.DR1120.AB480698          GCCAAAA--- ---------- ---------- ---ATTTTGA CTAGCGGAGG CTAGGA-GGA GAGAG--ATG GGTGCGAGAG CG
B.JP.99.DR1348.AB287370          GCCAAAA--- ---------- ---------- ---CTTTTGA CTAGCGGAGG CTAGAA-GGA GAGAG--ATG GGTGCGAGAG CG
B.JP.x.DR1673.AB564745           GCCAAAA--- ---------- ---------- ---TTTTTGA CTAGCGGAGG CTAGAA-GGA GAGAG--ATG GGTGCGAGAG CG
B.JP.x.DR1712.AB604946           GCCGAAA--- ---------- ---------- -ATTTTTTGA CTAGCGGAGG CTAGAA-GGA GAGAG--ATG GGTGCGAGAG CG
B.JP.x.DR1777.AB604948           GCCA------ ---------- ---------- ---ATTTTGA CTAGCGGAGG CTAGAA-GGA GAGAG--ATG GGTGCGAGAG CG
B.JP.x.JRC03B.AB565496           GCCAA----- ---------- ---------- ---TTTTTGA CTAGCGGAGG CTAGAA-GGA GAGAG--ATG GGTGCGAGAG CG
B.JP.x.JRC05B.AB565497           GCCAAAA--- ---------- ---------- --TTTTTTGA CTAGCGGAGG CTAGAA-GGA GAGAG--ATG GGTGCGAGAG CG
B.JP.x.JRC65B.AB565502           GCCAA----- ---------- ---------- ---TTTTTGA CTAGCGGAGG CTAGAA-GGA GAGAG--ATG GGTGCGAGAG CG
B.JP.x.pJPDR0796B02.AB565478     GCCAAAT--- ---------- ---------- ----TTTTGA CTAGCGGAGG CTAGAA-GGA GAGAG--ATG GGTGCGAGAG CG
B.JP.x.pJRC57B09.AB641836        GCCGAAA--- ---------- ---------- -TATTTTTGA CTAGCGGAGG CTAGAA-GGA GAGAG--ATG GGTGCGAGAG CG
B.KR.02.02OSG1.JQ429433          GCCAA----- ---------- ---------- ---TTTTTGA CTAGCGGAGG CTAGAA-GGA GAGAG--ATG GGTGCGAGAG CG
B.KR.03.03HJY8.JQ316131          GCCAAAA--- ---------- ---------- -AATTTTTGA CTAGCGGAGG CTAGAA-GGA GAGAG--ATG GGTGCGAGAG CG
B.KR.03.03KDE11.JQ316128         GCCAA----- ---------- ---------- ---TTTTTGA CTAGCGGAGG CTAGAA-GGA GAGAG--ATG GGTGCGAGAG CG
B.KR.03.03KGS5.JQ316132          CCCAAAT--- ---------- ---------- ---TTTTTGA CTAGCGGAGG CTAGAA-GGA GAGAG--ATG GGTGCGAGAG CG
B.KR.03.03LSH1.JQ316127          GCCAAAA--- ---------- ---------- ---TTTTTGA CTAGCGAAGG CTAGAA-GGA GAGAG--ATG GGTGCGAGAG CG
B.KR.03.03YGS3.JQ316135          GCCAAAA--- ---------- ---------- -AATTTTTGA CTAGCGGAGG CTAGAA-GGA GAGAG--ATG GGTGCGAGAG CG
B.KR.04.04CWS5.JQ316133          GCCGAAA--- ---------- ---------- ---TTTTTGA CTAGCGGAGG CTAGAA-GGA GAGAG--ATG GGTGCGAGAG CG
B.KR.04.04KJS8.JQ316130          GCCAAAA--- ---------- ---------- ---CTTTTGA CTAGCGGAGG CTAGAA-GGA GAGAG--ATG GGTGCGAGAG CG
B.KR.04.04KMH5.DQ295193          GCCAAAA--- ---------- ---------- ---TTTTTGA CTAGCGGAGG CTAGAA-GGA GAGAG--ATG GGTGCGAGAG CG
B.KR.04.04KMK5.JQ316126          GCCAAAA--- ---------- ---------- ---TTTTTGA CTAGCGGAGG CTAGAA-GGA GAGAG--ATG GGTGCGAGAG CG
B.KR.04.04LHS6.AY839827          GCCA------ ---------- ---------- TTTTGTTTGA CTAGCGGAGG CTAGAA-GGA GAGAG--ATG GGTGCGAGAG CG
B.KR.04.04LSK7.DQ295192          GCTGAAT--- ---------- ---------- ---TTTTTGA CTAGCGGAGG CTAGAA-GGA GAGAG--ATG GGTGCGAGAG CG
B.KR.04.04WK7_HIV_1_wk.DQ29519   GCCAAAA--- ---------- ---------- -ATTTTTTGA CTAGCGGAGG CTAGAA-GGA GAGAG--ATG GGTGCGAGAG CG
B.KR.05.05CSR3.DQ837381          GCCAAAA--- ---------- ---------- ---TTTTTGA CTAGCGGAGG CTAGAA-GGA GAGAG--ATG GGTGCGAGAG CG
B.KR.05.05YJN2.JQ316134          GCCAAAA--- ---------- ---------- -TATTTTTGA CTAGCGGAGG CTAGAA-GGA GAGAG--ATG GGTGCGAGAG CG
B.KR.07.07KYY4.JQ341411          GCCAAAA--- ---------- ---------- -ATATTTTGA CTAGCAGACG CTAAAA-AGA GAGAG--ATG GGTGCGAGAG CG
B.KR.99.99HYH2.JQ316129          GCCAA----- ---------- ---------- --TTTTTTGA CTAGCGGAGG CTAGAA-GGA GAGAG--ATG GGTGCGAGAG CG
B.MM.99.mSTD101.AB097870         GCCAAAA--- ---------- ---------- ---ATCTTGA CTAGCGGAGG CTAGAA-GGA GAGAG--ATG GGTGCGAGAG CG
B.NL.00.671_00T36.AY423387       GCCTAAA--- ---------- ---------- --AATTTTGA CTAGCGGAGG CTAGAA-GGA GAGAG--ATG GGTGCGAGAG CG
B.NL.86.3202A21_ACH3202A21.U34   CCCAAAA--- ---------- ---------- --ATTTTTGA CTAGCGGAGG CTAGAA-GGA GAGAG--ATG GGTGCGAGAG CG
B.NL.96.H434_42_A1.AY970948      GCCNAAA--- ---------- ---------- --AATTTTGA CTAGCGGAGG CTAGAA-GGA GAGAG--ATG GGTGCGAGAG CG
B.PE.06.502_0491_wg5.JF320183    GCCAAAA--- ---------- ---------- ---ATTTTGA CTAGCGGAGG CTAGAA-GGA GAGAG--ATG GGTGCGAGAG CG
B.PE.06.502_0524_FL04.JF320008   GCCAAAA--- ---------- ---------- -ATTTTTTGA CTAGCGGAGG CTAGAA-GGA GAGAG--ATG GGTGCGAGAG CG
B.PE.06.502_0648_FL02.JF320215   GCCAAAA--- ---------- ---------- ---TTTTTGA CTAGCGGAGG CTAGAA-GGA GAGAG--ATG GGTGCGAGAG CG
B.PE.06.502_0841_FL04.JF320208   GCCAAAA--- ---------- ---------- ---ATTTTGA CTAGCGGAGG CTAGAA-GGA GAGAG--ATG GGTGCGAGAG CG
B.PE.06.502_2794_FL05.JF320244   GCCA------ ---------- ---------- ---ATTTTGA CTAGCGGAGG CTAGAA-GGA GAGAG--ATG GGTGCGAGAG CG
B.PE.07.502_0525_wg5.JF320191    GCCAAAA--- ---------- --------AA TTTTTTTTGA CTAGCGGAGG CTAGAA-GGA GAGAG--ATG GGTGCGAGAG CG
B.PE.07.502_1047_wg5.JF320226    GCCAA----- ---------- ---------- ---TTTTTGA CTAGCGGAGG CTAGAA-GGA GAGAG--ATG GGTGCGAGAG CG
B.PE.07.502_1399_wg4.JF320013    GCCAAAA--- ---------- ---------- ---TTTTTGA CTAGCAGAGG CTAGAA-GGA GAGAG--ATG GGTGCGAGAG CG
B.PE.07.502_2254_FL6.JF320018    GCCATTA--- ---------- ---------- ---TTTTTGA CTAGCGGAGG CTAGAA-GGA GAGAG--ATG GGTGCGAGAG CG
B.PE.07.502_2349_wg2.JF320028    GCCAAAA--- ---------- ---------- ----TTTTGA CTAGCGGAGG CTAGAA-GGA GAGAG--ATG GGTGCGAGAG CG
B.PE.07.502_2622_wg1.JF320189    GCCAAAA--- ---------- ---------- --ACTTTTGA CTAGCAGAGG CTAGAA-GGA GAGAG--ATG GGTGCGAGAG CG
B.PE.07.502_2649_wg8.JF320019    GCCAAAA--- ---------- ---------- ---ATTTTGA CTAGCGGAGG CTAGAA-GGA GAGAG--ATG GGTGCGAGAG CG
B.RU.04.04RU128005.AY682547      GCCAAAA--- ---------- --------AA TTTTTTTTGA CTAGCGGAGG CTAGAA-GGA GAGAG--ATR GGTGCGAGAG CG
B.RU.04.04RU129005.AY751406      GCCAAAA--- ---------- ---------- ---ATTTTGA CTAGCGGAGG CTAGAA-GGA GAGAG--ATG GGTGCGAGAG CG
B.RU.04.04RU139089.AY751407      GCCAAAA--- ---------- -------ATA AAATTTCTGA CTAGCGGAGG CTAGAA-GGA GAGAG--ATG GGTGCGAGAG CG
B.RU.04.04RU139095.AY819715      GCCAAAA--- ---------- ---------- ---TTTTTGA CTAGCGGAGG CTAGAA-GGA GAGAG--ATG GGTGCGAGAG CG
B.RU.09.09RU4457.JX500709        GCCAAAA--- ---------- ---------- -ACTTTTTGA CTAGCGGAGG CTAGAA-GGA GAGAG--ATG GGTGCGAGAG CG
B.RU.10.10RU6629.JX500707        GCCAAAA--- ---------- ---------- ----TTTTGA CTAGCGGAGG CTAGAA-GGA GAGAG--ATG GGTGCGAGAG CG
B.RU.11.11RU21n.JX500708         GCCAAAA--- ---------- ---------- -ATTTTTTGA CTAGCGGAGG CTAGAA-GGA GAGAG--ATG GGTGCGAGAG CG
B.TH.06.AA010a_WG3.JX446800      GCCAAAT--- ---------- ---------- --ATTTTTGA CTAGCGGAGG CTAGAA-GGA GAGCG--ATG GGTGCGAGAG CG
B.TH.07.AA040a_WG11.JX447156     GCCAAAA--- ---------- ---------- -ATTTTTTGA CTAGCGGAGG CTAGAA-GGA GAGAG--ATG GGTGCGAGAG CG
B.TH.90.BK132.AY173951           GCCAAAA--- ---------- ---------- ---ATTTTGA CTAGCGGAGG CTAGAA-GGA GAGAG--ATG GGTGCGAGAG CG
B.TW.94.TWCYS_LM49.AF086817      GCCAAAA--- ---------- ---------- ---TTTTTGA CTAGCGGAGG CTAGAA-GGA GAGAG--ATG GGTGCGAGAG CG
B.US.00.14294_1.DQ853436         GCCATAA--- ---------- ---------- ----TTTTGA CTAGCGGAGG CTAGAA-GGA GAGAG--ATG GGTGCGAGAG CG
B.US.00.ES1_20.EF363123          GCCAAAA--- ---------- ---------- ---TTTTTGA CTAGCGGAGG CTAGAA-GGA GAGAG--ATG GGTGCGAGAG CG
B.US.00.RHPA_TF1.JN944917        GCCAA----- ---------- ---------- ---TTTTTGA CTAGCGGAGG CTAGAA-GGA GAGAG--ATG GGTGCGAGAG CG
B.US.00.THRO_TF1.JN944930        GCCAA----- ---------- ---------- --TTTTTTGA CTAGCGGAGG CTAGAA-GGA GAGAG--ATG GGTGCGAGAG CG
B.US.00.WITO_flB3.FJ496169       GCTGAAA--- ---------- ---------- ----TTTTGA CTAGCGGAGG CTAGAA-GGA GAGAG--ATG GGTGCGAGAG CG
B.US.01.REJO_TF1.JN944911        GCCGAAA--- ---------- ---------- --ATTTTTGA CTAGCGGAGG CTAGAA-GGA GAGAG--ATG GGTGCGAGAG CG
B.US.01.TRJO_flA1.FJ496151       GCCAAAA--- ---------- ---------- -ATTTTTTGA CTAGCGGAGG CTAGAA-GGA GAGAG--ATG GGTGCGAGAG CG
B.US.02.04013226_2_flH11.FJ496   GCCAAAA--- ---------- ---------- ---TTTTTGA CTAGCGGAGG CTAGAA-GGA GAGAG--ATG GGTGCGAGAG CG
B.US.04.ES10_53.EF363127         GCCAAAA--- ---------- ---------- ---TTTTTGA CTAGCGGAGG CTAGAA-GGA GAGAG--ATG GGTGCGAGAG CG
B.US.04.ES4_24.EF363124          GCCAAAA--- ---------- ---------- ---ATTTTGA CTAGCGGAGG CTAGAA-GGA GAGAG--ATG GGTGCGAGAG CG
B.US.04.ES8_43.EF363126          GCCGAAA--- ---------- ---------- ---TTTTTGA CTAGCGGAGG CTAGAA-GGA GAGAG--ATG GGTGCGAGAG CG
B.US.04.SAMI_WGA1.EU547186       GCCAT----- ---------- ---------- ---TTTTTGA CTAGCGGAGG CTAGAA-GGA GAGAG--ATG GGTGCGAGAG CG
B.US.04.USPI71101EI7y04051pcWG   GCCAAAA--- ---------- ---------- ---CTTTTGA CTAGCGGAGG CTAGAA-GGA GAGAG--ATG GGTGCGAGAG CG
B.US.04.USPI83747EI6y04121pcWG   GCCAAAT--- ---------- ---------- --TTTTTTGA CTAGCGGAGG CTAGAA-GGA GAGAG--ATG GGTGCGAGAG CG
B.US.05.04013396_0_flE6.FJ4960   GCCATAT--- ---------- ---------- ---TTTTTGA CTAGCGGAGG CTAGAA-GGA GAGAG--ATG GGTGCGAGAG CG
B.US.05.502_0223_wg1.JF320059    GCCAAAA--- ---------- ---------- ---TTTTTGA CTAGCGGAGG CTAGAA-GGA GAGAG--ATG GGTGCGAGAG CG
B.US.05.502_1400_FL02.JF320043   GCCG------ ---------- ---------- ---ATTTTGA CTAGCGGAGG CTAGAA-GGA GAGAG--ATG GGTGCGAGAG CG
B.US.05.502_2136_FL02.JF320185   GCCAAT---- ---------- ---------- ---TTTTTGA CTAGCGGAGG CTAGAA-GGA GAGAG--ATG GGTGCGAGAG CG
B.US.05.502_2495_wg02.JF320054   GCCAAAA--- ---------- ---------- ---ATTTTGA CTAGCGGAGG CTAGAA-GGA GAGAG--ATG GGTGCGAGAG CG
B.US.05.CR0307R.FJ469717         GCCAATG--- ---------- ---------- ----TTTTTT TCAGCGGAGG CTAGAA-GGA GAGAG--ATG GGTGCGAGAG CG
B.US.05.USPI38417EI33y05051pcW   GCCAAAA--- ---------- ---------- ---ACTTTGA CTAGCGGAGG CTAGAA-GGA GAGAG--ATG GGCGCGAGAG CG
B.US.05.USPI88403EI14y05121pcW   GCCAAAA--- ---------- ---------- ---TTTTTGA CTAGCGGAGG CTAGAA-GGA GAGAG--ATG GGTGCGAGAG CG
B.US.06.502_0341_FL05.JF320003   GCCAAAA--- ---------- ---------- ---TTTTTGA CTAGCGGAGG CTAGAA-GGA GAGAG--ATG GGTGCGAGAG CG
B.US.06.502_1046_FL04.JF320564   GCCATAT--- ---------- ---------- ----TTTTGA CTAGCGGAGG CTAGAA-GGA GAGAG--ATG GGTGCGAGAG CG
B.US.06.502_1174_FL09.JF320053   GCCAAAA--- ---------- ---------- -ATTTTGACT AGCGGAGGCT AGAAGG-AGA GAGAG--ATG GGTGCGAGAG CG
B.US.06.502_1211_FL01.JF320151   GCCATAA--- ---------- ---------- ---TTTTTGA CTAGCGGAGG CTAGAA-GGA GAGAG--ATG GGTGCGAGAG CG
B.US.06.502_1619_FL06.JF320126   GCCGAAA--- ---------- ---------- --AATTTTGA CTAGCGGAGG CTAGAA-GGA GAGAG--ATG GGTGCGAGAG CG
B.US.06.502_2667_FL03.JF320145   GCCAAAA--- ---------- ---------- -AATTTTTGA CTAGCGGAGG CTAGAA-GGA GAGAG--ATG GGTGCGAGAG CG
B.US.06.CH106_TF1.JN944897       GCCAAAA--- ---------- ---------- ---ACTTTGA CTAGCGGAGG CTAGAA-GGA GAGAG--ATG GGTGCGAGAG CG
B.US.06.CH40E_flA1.FJ495818      GCCAAAA--- ---------- ---------- ----TATTGA CTAGCGGAGG CTAGAA-GGA GAGAG--ATG GGTGCGAGAG CG
B.US.06.CH58E_flA9.FJ495941      GCCAAAA--- ---------- ---------- ---TTTTTGA CTAGCGGAGG CTAGAA-GGA GAGAG--ATG GGTGCGAGAG CG
B.US.06.CH77E_flA1.FJ496000      GCCAATA--- ---------- ---------- ---ATTTTGA CTAGCGGAGG CTAGAA-GGA GAGAG--ATG GGTGCGAGAG CG
B.US.07.502_0364_wg2.JF320563    GCCAAAA--- ---------- ---------- -AATTTTTGA CTAGCAGAGG CTAGAA-GGA GAGAG--ATG GGTGCGAGAG CG
B.US.07.502_1115_wg1.JF320045    GCCGAAA--- ---------- ---------- ---ATTTTGA CTAGCGGAGG CTAGAA-GGA GAGAG--ATG GGTGCGAGAG CG
B.US.07.502_1478_wg4.JF320150    GCCT------ ---------- ---------- ---TTTTTGA CTAGCGGAGG CTAGAA-GGA GAGAG--ATG GGTGCGAGAG CG
B.US.07.502_1897_wg6.JF320182    GCCAAAA--- ---------- --------AT AAAGTTTTGA CTAGCGGAGG CTAGAA-GGA GAGAG--ATG GGTGCGAGAG CG
B.US.07.502_2289_05.JF320197     GCCAA----- ---------- ---------- ---TTTTTGA CTAGCGGAGG CTAGAA-GGA GAGAG--ATG GGTGCGAGAG CG
B.US.09.C1P.GU733713             GCCAAA---- ---------- ---------- ---TTTTTGA CTAGCGGAGG CTAGAA-GGA GAGAG--ATG GGTGCGAGAG CG
B.US.09.DEMB09US002.JX140657     GCCATAA--- ---------- ---------- ---TTTTTGA CTAGCGGAGG CTAGAA-GGA GAGAG--ATG GGTGCGAGAG CG
B.US.10.CP1.JN397365             GCCAAAG--- ---------- ---------- --ATTCTTTG ACTAACGGAG GCTAGA-AAG AGAGG--ATG GGTGCGAGAG CG
B.US.10.VC1.JN397364             GCCAAAA--- ---------- ---------- -GTTTTTTGA CTAGCGAAGG CTAGAA-GGA GAGAG--ATG GGTGCGAGAG CG
B.US.11.ES38.JN397362            GCCA------ ---------- ---------- --TATTTTGA CTAGCGGAGG CTAGAA-GGA GAGAG--ATG GGTGCGAGAG CG
B.US.83.5018_83.AY835777         GCCAAAA--- ---------- ---------- ---ATTTTGA CTAGCGGAGG CTAGAA-GGA GAGAG--ATG GGTGCGAGAG CG
B.US.83.5157_83.AY835781         GCCAAAA--- ---------- ---------- ---ATTTTGA CTAGCGGAGG CTAGAA-GGA GAGAG--ATG GGTGCGAGAG CG
B.US.83.RF_HAT3.M17451           GCCGAAA--- ---------- ---------- ---ATTTTGA CTAGCGGAGG CTAGAA-GGA GAGAG--ATG GGTGCGAGAG CG
B.US.83.SF2_LAV2_ARV2.K02007     GCCAA----- ---------- ---------- ---TTTTTGA CTAGCGGAGG CTAGAA-GGA GAGAGAGATG GGTGCGAGAG CG
```

```
B.US.84.5019_84.AY835779         GCCAAAA--- ---------- ---------- ---ATTTTGA CTAGCGGAGG CTAGAA-GGA GAGAG--ATG GGTGCGAGAG CG
B.US.84.MNCG_MN.M17449           GCCAAAA--- ---------- ---------- --ATTCTTGA CTAGCGGAGG CTAGAA-GGA GAGAG--ATG GGTGCGAGAG CG
B.US.84.NY5CG.M38431             GCCAAAA--- ---------- ---------- ---ATTTTGA CTAGCGGAGG CTAGAA-GGA GAGAG--ATG GGTGCGAGAG CG
B.US.84.SF33.AY352275            GCCAAAA--- ---------- ---------- --AATTTTGA CTAGCGGAGG CTAGAA-GGA GAGAG--ATG GGTGCGAGAG CG
B.US.85.5077_85.AY835769         GCCAAAA--- ---------- ---------- ---ATTTTGA CTAGCGGAGG CTAGAA-GGA GAGAG--ATG GGTGCGAGAG CG
B.US.85.Ba_L.AB221005            GCCAAAA--- ---------- ---------- ---TTTTTGA CTAGCGGAGG CTAGAA-GGA GAGAG--ATG GGTGCGAGAG CG
B.US.86.5084_86.AY835775         GCCAAAA--- ---------- ---------- ---ATTTTGA CTAGCGGAGG CTAGAA-GGA GAGAG--ATG GGTGCGAGAG CG
B.US.86.5096_86.AY835749         GCCAAAA--- ---------- ---------- ---TTTTTGA CTAGCGGAGG CTAGAA-GGA GAGAG--ATG GGTGCGAGAG CG
B.US.86.5127_86.AY835774         GCCAAAA--- ---------- ---------- ---ATTTTGA CTAGCGGAGG CTAGAA-GGA GAGAG--ATG GGTGCGAGAG CG
B.US.86.AD87_ADA.AF004394        GCCAAAA--- ---------- ---------- ---ATTTTGA CTAGCGGAGG CTAGAA-GGA GAGAG--ATG GGTGCGAGAG CG
B.US.86.JRFL_JR_FL.U63632        GCCAAAA--- ---------- ---------- ---ATTTTGA CTAGCGGAGG CTAGAA-GGA GAGAG--ATG GGTGCGAGAG CG
B.US.86.YU_2.M93258              GCCAAAA--- ---------- ---------- -AATTTTTGA CTAGCGGAGG CTAGAA-GGA GAGAG--ATG GGTGCGAGAG CG
B.US.87.5113_87.AY835758         GCCAAAA--- ---------- ---------- ---ATTTTGA CTAGCGGAGG CTAGAA-GGA GAGAG--ATG GGTGCGAGAG CG
B.US.87.BC_BCSG3.L02317          GCCAAAA--- ---------- ---------- ---ATTTTGA CTAGCGGAGG CTAGAA-GGA GAGAG--ATG GGTGCGAGAG CG
B.US.88.5160_88.AY835763         GCCAAAA--- ---------- ---------- ---ATTTTGA CTAGCGGAGG CTAGAA-GGA GAGAG--ATG GGTGCGAGAG CG
B.US.88.WR27.AF286365            GCCATAA--- ---------- ---------- ----TTTTGA CTAGCGGAGG CTAGAA-GGA GAGAG--ATG GGTGCGAGAG CG
B.US.89.P896_89_6.U39362         GCCAAAT--- ---------- ---------- ---TTTTTGA CTAGCGGAGG CTAGAA-GGA GAGAG--ATG GGTGCGAGAG CG
B.US.90.US1.AY173952             GCCAAAA--- ---------- ---------- ---ATTTTGA CTAGCGGAGG CTAGAA-GGA GAGAG--ATG GGTGCGAGAG CG
B.US.90.US2.AY173953             GCCAAAA--- ---------- ---------- --ATTTTTGA CTAGCGGAGG CTAGAA-GGA GAGAG--ATG GGTGCGAGAG CG
B.US.90.US3.AY173954             GCCAAAA--- ---------- ---------- ----TTTTGA CTAGCGGAGG CTAGAA-GGA GAGAG--ATG GGTGCGAGAG CG
B.US.90.US4.AY173955             GCCAAAA--- ---------- ---------- ---TTTTTGA CTAGCGGAGG CTAGAA-GGA GAGAG--ATG GGTGCGAGAG CG
B.US.90.WCIPR.U69591             GCCAAAA--- ---------- ---------- ---ATTTTGA CTAGCGGAGG CTAGAA-GGA GAGAG--ATG GGTGCGAGAG CG
B.US.90.WEAU160_GHOSH.U21135     GCCGAAA--- ---------- ---------- ----TTTTGA CTAGCGGAGG CTAGAA-GGA GAGAG--ATG GGTGCGAGAG CG
B.US.91.5048_91.AY835761         GCCAAAA--- ---------- ---------- ---ATTTTGA CTAGCGGAGG CTAGAA-GGA GAGAG--ATG GGTGCGAGAG CG
B.US.91.DH12_3.AF069140          GCCAAAA--- ---------- ---------- ----TTTTGA CTAGCGGAGG CTAGAA-GGA GAGAG--ATG GGTGCGAGAG CG
B.US.91.SUMA_flC5.FJ496145       GCCGAAA--- ---------- ---------- --ATTTTTGA CTAGCGGAGG CTAGAA-GGA GAGAG--ATG GGTGCGAGAG CG
B.US.93.WCD32P0793.DQ487188      GCCAAAA--- ---------- ---------- ---TTTTTGA CTAGCGGAGG CTAGAA-GGA GAGAG--ATG GGTGCGAGAG CG
B.US.93.WCM32P0793.DQ487190      GCCAAAA--- ---------- ---------- ----TTTTGA CTAGCGGAGG CTAGAA-GGA GAGAG--ATG GGTGCGAGAG CG
B.US.94.5082_94.AY835773         GCCAAAA--- ---------- ---------- ---ATTTTGA CTAGCGGAGG CTAGAA-GGA GAGAG--ATG GGTGCGAGAG CG
B.US.95.5073_95.AY835768         GCCAAAA--- ---------- ---------- ---ATTTTGA CTAGCGGAGG CTAGAA-GGA GAGAG--ATG GGTGCGAGAG CG
B.US.95.USPI90770EI72y95091pcW   GCCAAAA--- ---------- ---------- ---ATTTTGA CTAGCGGAGG CTAGAA-GGA GAGAG--ATG GGTGCGAGAG CG
B.US.96.5155_96.AY835753         GCCAAAA--- ---------- ---------- ---TTTTTGA CTAGCGGAGG CTAGAA-GGA GAGAG--ATG GGTGCGAGAG CG
B.US.96.USPI55751EI32y96071pcW   GCCAAAA--- ---------- ---------- ---ATTTTGA CTAGCGGAGG CTAGAA-GGA GAGAG--ATG GGTGCGAGAG CG
B.US.97.ARES2.AB078005           GCCAAAC--- ---------- ---------- ---ATTTTGA CTAGCGGAGG CTAGAA-GGA GAGAG--ATG GGTGCGAGAG CG
B.US.98.15384_1.DQ853463         GCCATAA--- ---------- ---------- ----TTTTGA CTAGCGGAGG CTAGAA-GGA GAGAG--ATG GGTGCGAGAG CG
B.US.98.98USHVTN1925c1.AY56010   GCCAAAA--- ---------- ---------- ---TTTTTGA CTAGCGGAGG CTAGAA-GGA GAGAG--ATG GGTGCGAGAG CG
B.US.98.98USHVTN3605c9.AY56010   GCCAAAA--- ---------- ---------- ---TTTTTGA CTAGCGGAGG CTAGAA-GGA GAGAG--ATG GGTGCGAGAG CG
B.US.98.98USHVTN8229c6.AY56010   GCCAAAA--- ---------- ---------- -ATTTTTTGA CTAGCGGAGG CTAGAA-GGA GAGAG--ATG GGTGCGAGAG CG
B.US.98.98USHVTN941c1.AY560110   GCCAAAA--- ---------- ---------- ---TTTTTGA CTAGCGGAGG CTAGAA-GGA GAGAG--ATG GGTGCGAGAG CG
B.US.98.WC3_0498_4.EF175212      GCTGAAA--- ---------- ---------- ---ATTTTGA CTAGCGGAGG CTAGAA-GGA GAGAG--ATG GGTGCGAGAG CG
B.US.x.CR0059T.FJ469694          GCCAAAA--- ---------- ---------- -CTTTTTTGA CTGGCGGAGG CTAGAA-GGA GAGAG--ATG GGTGCGAGAG CG
B.US.x.sample_C_BID_D617.JX503   GCCA------ ---------- ---------- --TTTTTTGA CTAGCGGAGG CTAGAA-GGA GAGAG--ATG GGTGCGAGAG CG
B.VE.10.DEMB10VE001.JX140659     GCCAAAA--- ---------- ---------- ---TTTTTGA CTAGCGGAGG CTAGAA-GGA GAGAG--ATG GGTGCGAGAG CG
B.ZA.03.03ZAPS045MB2.DQ396398    GCCAAA---- ---------- ---------- ---TTTTTGA CTAGCGGAGG CTAGAA-GGA GAGAG--ATG GGTGCGAGAG CG
C.BR.02.02BR2022.JN692434        GCCA------ ---------- ---------T TTTCATTTGA CTAGCGGAGG CTAGAA-GGA GAGAG--ATG GGTGCGAGAG CG
C.BR.04.04BR013.AY727522         GCCA------ ---------- ---------T TTTATTTTGA CTAGCGGAGG CTAGAA-GGA GAGAG--ATG GGTGCGAGAG CG
C.BR.04.04BR021.AY727523         GCCA------ ---------- ---------T TTTTATTTGA CTAGCGGAGG CTAGAA-GGA GAGAG--ATG GGTGCGAGAG CG
C.BR.04.04BR038.AY727524         GCCA------ ---------- ---------T TTTTATTTGA CTAGCGGAGG CTAGAA-GGA GAGAG--ATG GGTGCGAGAG CG
C.BR.04.04BR073.AY727525         GCCA------ ---------- ---------T TTTTATTTGA CTAGCGGAGG CTAGAA-GGA GAGAG--ATG GGTGCGAGAG CG
C.BR.07.DEMC07BR003.JX140663     GCCAATT--- ---------- ---------- TTTTATTTGA CTAGCGGAGG CTAGAA-GGA GAGAG--ATG GGTGCGAGAG CG
C.BR.92.BR025_d.U52953           GCCA------ ---------- ---------T TTTTATTTGA CTAGCGGAGG CTAGAA-GGA GAGAG--ATG GGTGCGAGAG CG
C.BR.98.98BR004.AF286228         GCCA------ ---------- ---------T TTTTATTTGA CTAGCGGAGG CTAGAA-GGA GAGAG--ATG GGTGCGAGAG CG
C.BW.00.00BW07621.AF443088       GCCAAAA--- ---------- ---------- TTTTATTTGA CTAGCGGAGG CTAGAA-GGA GAGAG--ATG GGTGCGAGAG CG
C.BW.00.00BW076820.AF443089      GCCAAAT--- ---------- ---------- -TTTATTTGA CTAGCAGAGG CTAGAA-GGA GAGAG--ATG GGTGCGAGAG CG
C.BW.00.00BW087421.AF443090      GCCA------ ---------- --------AT TTTATTTTGA CTAGCGGAGG CTAGAA-GGA GAGAG--ATG GGTGCGAGAG CG
C.BW.00.00BW147127.AF443091      GCCAAAT--- ---------- ---------- -TTTATTTGA CTAGCGGAGG CTAGAA-GGA GAGAG--ATG GGTGCGAGAG CG
C.BW.00.00BW16162.AF443092       GCCAAAT--- ---------- ---------- -TTTATTTGA CTAGCGGAGG CTAGAA-GGA GAGAG--ATG GGTGCGAGAG CG
C.BW.00.00BW1686.AF443093        GCCAAAC--- ---------- ---------- TTTAATTTGA CTAGCGGAGG CTAGAA-GGA GAGAG--ATG GGTGCGAGAG CG
C.BW.00.00BW17593.AF443094       GCCA------ ---------- --------AT TTTTATTTGA CTAGCGGAGG CTAGAA-GGA GAGAG--ATG GGTGCGAGAG CG
C.BW.00.00BW17732.AF443095       GCCAAAT--- ---------- ---------- TTTATTTTGA CTAGCGGAGG CTAGAA-GGA GAGAG--ATG GGTGCGAGCG CG
C.BW.00.00BW17835.AF443096       GCCAAAA--- ---------- ---------- TTTTATTTGA CTAGCGGAGG CTAGAA-GGA GAGAG--ATG GGTGCGAGAG CG
C.BW.00.00BW17956.AF443097       GCCAAAT--- ---------- ---------- -TTTATTTGA CTAGCGGAGG CTAGAA-GGA GAGAG--ATG GGTGCGAGAG CG
C.BW.00.00BW18113.AF443098       GCCA------ ---------- --------AT TTTTATTTGA CTAGCGGAGG CTAGAA-GGA GAGAG--ATG GGTGCGAGAG CG
C.BW.00.00BW18595.AF443099       GCCAAAA--- ---------- ---------- TTTTATTTGA CTAGCGGAGG CTAGAA-GGA GAGAG--ATG GGTGCGAGAG CG
C.BW.00.00BW18802.AF443100       GCCAAAT--- ---------- ---------- -TTTATTTGA CTAGCGGAGG CTAGAA-GGA GAGAG--ATG GGTGCGAGAG CG
C.BW.00.00BW192113.AF443101      GCCAAAT--- ---------- ---------- TTTATTTTGA CTAGCGGAGG CTAGAA-GGA GAGAG--ATG GGTGCGAGAG CG
C.BW.00.00BW20361.AF443102       GCCAAAT--- ---------- ---------- -TTTATTTGA CTAGCGGAGG CTAGAA-GGA GAGAG--ATG GGTGCGAGAG CG
C.BW.00.00BW20636.AF443103       GCCA------ ---------- --------AT TTTTATTTGA CTAGCGGAGG CTAGAA-GGA GAGAG--ATG GGTGCGAGAG CG
C.BW.00.00BW20872.AF443104       GCCAATT--- ---------- ---------- --TTATTTGA CTAGCGGAGG CTAGAA-GGA GAGAG--ATG GGTGCGAGAG CG
C.BW.00.00BW2127214.AF443105     GCCA------ ---------- --------AT TTTTATTTGA CTAGCGGAGG CTAGAA-GGA GAGAG--ATG GGTGCGAGAG CG
C.BW.00.00BW22767.AF443107       GCCAAAT--- ---------- ---------- TTTTATTTGA CTAGCGGAGG CTAGAA-GGA GAGAG--ATG GGTGCGAGAG CG
C.BW.00.00BW38193.AF443108       GCCGATT--- ---------- ---------- --TTATTTGA CTAGCGGAGG CTAGAA-GGA GAGAG--ATG GGTGCGAGAG CG
C.BW.00.00BW38428.AF443109       GCCGAAT--- ---------- ---------- -TTTATTTGA CTAGCGGAGG CTAGAA-GGA GAGAG--ATG GGTGCGAGAG CG
C.BW.00.00BW38713.AF443110       GCCAAAT--- ---------- ---------- -TTTATTTGA CTAGCGGAGG CTAGAA-GGA GAGAG--ATG GGTGCGAGAG CG
C.BW.00.00BW3876_9.AF443111      GCCAAAA--- ---------- --------AA ATTTTTTTGA CTAGCGGAGG CTAGAA-GGA GAGAG--ATG GGTGCGAGAG CG
C.BW.00.00BW3886_8.AF443112      GCCAAAT--- ---------- ---------- -TTTATTTGA CTAGCGGAGG CTAGAA-GGA GAGAG--ATG GGTGCGAGAG CG
C.BW.00.00BW3891_6.AF443113      GCCAAAT--- ---------- ---------- -TTTATTTGA CTAGCGGAGG CTAGAA-GGA GAGAG--ATG GGTGCGAGAG CG
C.BW.00.00BW3970_2.AF443114      GCCA------ ---------- --------AT TTTTATTTGA CTAGCGGAGG CTAGAA-GGA GAGAG--ATG GGTGCGAGAG CG
C.BW.00.00BW5031_1.AF443115      GCCA------ ---------- --------AT TTTTATTTGA CTAGCGGAGG CTAGAA-GGA GAGAG--ATG GGTGCGAGAG CG
C.BW.96.96BW01B03.AF110959       GCCAAAT--- ---------- ---------- TTTTATTTGA CTAGCGGAGG CTAAAA-GGA GAGAG--ATG GGTGCGAGAG CG
C.BW.96.96BW0402.AF110962        GCCAAAT--- ---------- ---------- -TTTATTTGA CTAGCGGAGG CTAGAA-GGA GAGAG--ATG GGTGCGAGAG CG
C.BW.96.96BW0502.AF110967        GCCA------ ---------- ---------T TTTTATTTGA CTAGCGGAGG CTAGAA-GGA GAGAG--ATG GGTGCGAGAG CG
C.BW.96.96BW06.AF290028          GCCAAAT--- ---------- ---------- -TTTATTTGA CTAGCGGAGG CTAGAA-GGA GAGAG--ATG GGTGCGAGAG CG
C.BW.96.96BW1104.AF110969        GCCAAAT--- ---------- ---------- -TTTATTTGA CTAGCGGAGG CTAGAA-GGA GAGAG--ATG GGTGCGAGAG CG
C.BW.96.96BW1210.AF110972        GCCA------ ---------- --------AT TTTATTTTGA CTAGCGGAGG CTAGAA-GGA GAGAG--ATG GGTGCGAGAG CG
C.BW.96.96BW15C02.AF110974       GCCAAAT--- ---------- ---------- -TTTATTTGA CTAGCGGAGG CTAGAA-GGA GAGAG--ATG GGTGCGAGAG CG
C.BW.96.96BW16B01.AF110976       GCCA------ ---------- --------AT TTTTATTTGA CTAGCGGAGG CTGGAA-GGA GAGAG--ATG GGTGCGAGAG CG
C.BW.96.96BW17.AF110980          GCCAAAT--- ---------- ---------- -TTTATTTGA CTAGCGGAGG CTAGAA-GGA GAGAG--ATG GGTGCGAGAG CG
C.BW.96.96BWM032.AF443075        GCCA------ ---------- --------AT TTTTATTTGA CTAGCGGAGG CTAGAA-GGA GAGAG--ATG GGTGCGAGAG CG
C.BW.96.96BWMO1_5.AF443074       GCCA------ ---------- ---------- TTTTATTTGA CTAGCGGAGG CTAGAA-GGA GAGAG--ATG GGTGCGAGAG CG
C.BW.98.98BWMC122.AF443076       GCCA------ ---------- --------AT TTTTATTTGA CTAGCGGAGG CTAGAA-GGA GAGAG--ATG GGTGCGAGAG CG
C.BW.98.98BWMC134.AF443077       GCCAAAT--- ---------- ---------- -TTTATTTGA CTAGCGGAGG CTAGAA-GGA GAGAG--ATG GGTGCGAGAG CG
C.BW.98.98BWMC14A3.AF443078      GCCGAAT--- ---------- ---------- -TTTATTTGA CTAGCGGAGG CTAGAA-GGA GAGAG--ATG GGTGCGAGAG CG
C.BW.98.98BWMO1410.AF443079      GCCAATT--- ---------- ---------T TTTTATTTGA CTAGCGGAGG CTAGAA-GGA GAGAG--ATG GGTGCGAGAG CG
C.BW.98.98BWMO18D5.AF443080      GCCAAAT--- ---------- ---------- -TTTATTTGA CTAGCGGAGG CTAGAA-GGA GAGAG--ATG GGTGCGAGAG CG
C.BW.98.98BWMO36A5.AF443081      GCCAATT--- ---------- ---------- --TTATTTGA CTAGCGGTGG CTAGAA-GGA GAGAG--ATG GGTGCGAGAG CG
C.BW.98.98BWMO37D5.AF443082      GCCAAAA--- ---------- ---------- -TTTATTTGA CTAGCGGAGG CTAGAA-GGA GAGAG--ATG GGTGCGAGAG CG
C.BW.99.99BW393212.AF443083      GCCA------ ---------- --------AT TTTTATTTGA CTAGCGGAGG CTAGAA-GGA GAGAG--ATG GGTGCGAGAG CG
C.BW.99.99BW46424.AF443084       GCCAAAA--- ---------- ---------- TTTTATTTGA CTAGCGGAGG CTAGAA-GGA GAGAG--ATG GGTGCGAGAG CG
C.BW.99.99BW4745.AF443085        GCCTATT--- ---------- ---------- --TTATTTGA CTAGCGGAGG CTAGAA-GGA GAGAG--ATG GGTGCGAGAG CG
C.BW.99.99BW47547.AF443086       GCCAAAT--- ---------- ---------- -TTTATTTGA CTAGCGGAGG CTAGAA-GGA GAGAG--ATG GGTGCGAGAG CG
C.BW.99.99BWMC168.AF443087       GCCA------ ---------- --------AT TTTTATTTGA CTAGCGGAGG CTAGAA-GGA GAGAG--ATG GGTGCGAGAG CG
C.DJ.x.DJ259.AB485643            GCCATAT--- ---------- ---------- -TTTATTTGA CTAGCAAGGG CTAGAA-GGA GAGAG--ATG GGTGCGAGAG CG
C.DK.01.CTL_015.EF514713         GCCAATT--- ---------- ---------- --TTATTTGA CTAGCGGAGG CTAGAA-GGA GAGAG--ATG GGTGCGAGAG CG
C.ES.06.X1936.EU786673           GCCA------ ---------- --------AT TTTTATTTGA CTAGCGGAGG CTAGAA-GGA GAGAG--ATG GGTGCGAGAG CG
```

```
C.ES.07.X2118_2.EU884500          GCCAAAA--- ---------- ---------- TTTTATTTGA CTAGCGGAGG CTAGAA-GGA GAGAG--ATG GGTGCGAGAG CG
C.ES.08.X2363_2.EU786681          ---------- ---------- ---CGCCAAA TTTTAATTGA CTAGCGGAGG CTAGAA-GGA GAGAG--ATG GGTGCGAGAG CG
C.ET.86.ETH2220.U46016            GCCA------ ---------- --------AT TTTTATTTGA CTAGCGGAGG CTAGAA-GGA GAGAG--ATG GGTGCGAGAG CG
C.IL.98.98IS002.AF286233          GCCA------ ---------- --------AT TTTTATTTGA CTAGCGGAGG CTAGAA-GGA GAGAG--ATG GGTGCGAGAG CG
C.IN.03.D24.EF469243              GCCAATT--- ---------- ---------- --TTATTTGA CTAGCGGAGG CTAGAA-GGA GAGAG--ATG GGTGCGAGAG CG
C.IN.93.93IN101.AB023804          GCCAATT--- ---------- ---------- --TTATTTGA CTAGCGGAGG CTAGAA-GGA GAGAG--ATG GGTGCGAGAG CG
C.IN.93.93IN904.AF067157          GCCAATT--- ---------- ---------- --TTATTTGA CTAGCGGAGG CTAGAA-GGA GAGAG--ATG GGTGCGAGAG CG
C.IN.93.93IN999.AF067154          GCCAAAT--- ---------- ---------- -TTTATTTGA CTAGCGGAGG CTAGAA-GGA GAGAG--ATG GGTGCGAGAG CG
C.IN.94.94IN11246.AF067159        GCCAATT--- ---------- ---------- --TTATTTGA CTAGCGGAGG CTAGAA-GGA GAGAG--ATG GGTGCGAGAG CG
C.IN.94.94IN476.AF286223          GCCA------ ---------- --------AT TTTTATTTGA CTAGCGGAGG CTAGAA-GGA GAGAG--ATG GGTGCGAGAG CG
C.IN.95.95IN21068.AF067155        GCCAATT--- ---------- ---------- --TTATTTGA CTAGCGGAGG CTAGAA-GGA GAGAG--ATG GGTGCGAGAG CG
C.IN.98.98IN012.AF286231          GCCAAAT--- ---------- ---------- -TTTATTTGA CTAGCGGAGG CTAGAA-GGA GAGAG--ATG GGTGCGAGAG CG
C.IN.98.98IN022.AF286232          GCCGATT--- ---------- ---------- --TTATTTGA CTAGCGGAGG CTAGAA-GGA GAGAG--ATG GGTGCGAGAG CG
C.IN.99.01IN565_10.AY049708       GCCA------ ---------- --------AT TTTTATTTGA CTAGCGGAGG CTAGAA-GGA GAGAG--ATG GGTGCGAGAG CG
C.IN.x.VB39.EF694033              GCCA------ ---------- --------AT TTTTATTTGA CTAGCGGAGG CTAGAA-GGA GAGAG--ATG GGTGCGAGAG CG
C.IN.x.VB49.EF694036              GCCTAAT--- ---------- ---------- -TTTATTTGA CTAGCGGAGG CTAGAA-GGA GAGAG--ATG GGTGCGAGCG CG
C.MM.99.mIDU101_3.AB097871        GCCA------ ---------- --------AT TTTTATTTGA CTAGCGGAGG CTAGAA-GGA GAGAG--ATG GGTGCGAGAG CG
C.TZ.98.98TZ013.AF286234          GCCAATT--- ---------- ---------- --TTATTTGA CTGGCGGAGG CTAGAA-GGA GAGAG--ATG GGTGCGAGAG CG
C.TZ.98.98TZ017.AF286235          GCCA------ ---------- --------TT TTTTATTTGA CTAGCGGAGG CTAGAA-GGA GAGAG--ATG GGTGCGAGAG CG
C.ZA.00.1069MB.AY838567           GCCAAAT--- ---------- ---------- -TTTATTTGA CTAGCGGAGG CTAGAA-GGA GAGAG--ATG GGTGCGAGAG CG
C.ZA.00.1119MB.AY463229           GCCAAAT--- ---------- ---------- -TTTATTTGA CTAGCGGAGG CTAGAA-GGA GAGAG--ATG GGTGCGAGAG CG
C.ZA.00.1134MB.AY463217           GCCGAAT--- ---------- ---------- -TTTACTTGA CTAGCGGAGG CTAGAA-GGA GAGAG--ATG GGTGCGAGAG CG
C.ZA.00.1157M3M.AY585266          GCCAATT--- ---------- ---------- --TTATTTGA CTAGCGGAGG CTAGAA-GGA GAGAG--ATG GGTGCGAGAG CG
C.ZA.00.1162MB.AY463224           GCCAAAT--- ---------- ---------- -TTTATTTGA CTAGCGGAGG CTAGAA-GGA GAGAG--ATG GGTGCGAGAG CG
C.ZA.00.1165MB.AY463230           GCCA------ ---------- --------AT TTTATTTTGA CTAGCGGAGG CTAGAA-GGA GAGAG--ATG GGTGCGAGAG CG
C.ZA.00.1168MB.AY463231           GCCAAAT--- ---------- ---------- -TTTATTTGA CTAGCGGAGG CTAGAA-GGA GAGAG--ATG GGTGCGAGAG CG
C.ZA.00.1170MB.AY463225           GCCAAAT--- ---------- ---------- -TTTATTTGA CTAGCGGAGG CTAGGA-GGA GAGAG--GTG GGTGCGAGAG CG
C.ZA.00.1171MB.AY463232           GCCAAAT--- ---------- ---------- TTTATTTTGA CTAGCGGAGG CTAGAA-GGA GAGAG--ATG GGTGCGAGAG CG
C.ZA.00.1176MB.AY463218           GCCGAAT--- ---------- ---------- -TTTATTTGA CTAGCGGTGG CTAGAA-GGA GAGAGAGATG GGTGCGAGAG CG
C.ZA.00.1178MB.AY463233           GCCAAAA--- ---------- ---------- TTTTATTTGA CTAGCGGAGG CTAGAA-GGA GAGAG--ATG GGTGCGAGAG CG
C.ZA.00.1184MB.AY838566           GCCA------ ---------- --------AT TTTTATTTGA CTAGCGGAGG CTAGAA-GGA GAGAG--ATG GGTGCGAGAG CG
C.ZA.00.1189MB.AY838565           GCCAAAT--- ---------- ---------- -TTTATTTGA CTAGCGGAGG CTAGAA-GGA GAGAG--ATG GGTGCGAGAG CG
C.ZA.00.1192M3M.AY463219          GCCGAAT--- ---------- ---------- -TTTATTTGA CTAGCGGAGG CTAGAA-AGA GAGAG--ATG GGTGCGAGAG CG
C.ZA.00.1195MB.AY463220           GCCA------ ---------- --------AT TTTTATTTGA CTAGCGGAGG CTAGAA-GGA GAGAG--ATG GGTGCGAGAG CG
C.ZA.00.1197MB.AY463234           CCCA------ ---------- --------AT TTTTATTTGA CTAGCGGAGG CTAGAA-GGA GAGAG--ATG GGTGCGAGAG CG
C.ZA.00.1210MB.AY463221           GCCGAAT--- ---------- ---------- -TTTATTGGA CTAGCGGAGG CTAGAA-GGA GAGAG--ATG GGTGCGAGTG CG
C.ZA.00.1214MB.AY463236           GCCAAAA--- ---------- ---------- -TTTATTTGA CTAGCGGAGG CTAGAA-GGA GAGAG--ATG GGTGCGAGAG CG
C.ZA.00.1217MB.AY463226           GCCAAAT--- ---------- ---------- TTTATTTTGA CTAGCGGAGG CTAGAA-GGA GAGAG--ATG GGTGCGAGAG CG
C.ZA.00.1225MB.AY463227           GCCAAAT--- ---------- ---------- -TTTATTTGA CTAGCGGAGG CTAGAA-GGA GAGAG--ATG GGTGCGAGAG CG
C.ZA.00.1228MB.AY463222           GCCAAAT--- ---------- ---------- -TTTATTTGA CTAGCGGAGG CTAGAA-GGA GAGAG--ATG GGTGCGAGAG CG
C.ZA.00.J112MA.AY838568           GCCA------ ---------- ---------- TTTTATTTGA CTAGCGGAGG CTAGAA-GGA GAGAG--ATG GGTGCGAGAG CG
C.ZA.00.J38MA.AY463228            GCCGAAT--- ---------- ---------- -TTTATTTGA CTAGCGGAGG CTAGAA-GGA GAGAG--ATG GGTGCGAGAG CG
C.ZA.01.01ZATM45.AY228557         GCCA------ ---------- --------AT TTTTATTTGA CTAGCGGAGG CTAGAA-GGA GAGAG--ATG GGTGCGAGAG CG
C.ZA.01.2134MB.AY463237           GCCA------ ---------- --------TT TTTTATTTGA CTAGCGGAGG CTAGAA-GGA GAGAG--ATG GGTGCGAGAG CG
C.ZA.01.J54Ma.AY463223            GCCAATT--- ---------- ---------- --TTATTTGA CTAGCGGAGG CTAGAA-GGA GAGAG--ATG GGTGCGAGAG CG
C.ZA.02.02ZAPS001MB1.DQ275648     GCCAAAA--- ---------- ---------- TTTTATTTGA CTAGCGGAGG CTAGGA-GGA GAGAG--ATG GGTGCGAGAG CG
C.ZA.02.02ZAPS005MB1.DQ351235     GCCAAAT--- ---------- ---------- -TTTATTTGA CTAGCGGAGG CTAGAA-GGA GAGAG--ATG GGTGCGAGAG CG
C.ZA.02.02ZAPS006MB1.DQ351220     GCCAATT--- ---------- ---------- --TTATTTGA CTAGCGGAGG CTAGAA-GGA GAGAG--ATG GGTGCGAGAG CG
C.ZA.02.02ZAPS008MB1.DQ275647     GCCT------ ---------- ---------- -TTTATTTGA CTAGCGGAGG CTAGAA-GGA GAGGG--ATG GGTGCGAGAG CG
C.ZA.02.02ZAPS013MB1.DQ351222     GCCGAAT--- ---------- ---------- -TTTATTTGA CTAGCGGAGG TTAGAA-GGA GAGAG--ATG GGTGCGAGAG CG
C.ZA.02.02ZAPS014MB1.DQ351218     GCCAAAT--- ---------- ---------- -TTTATTTGA CTAGCGGAGG CTAGAA-GGA GAGAG--ATG GGTGCGAGAG CG
C.ZA.02.02ZAPS015MB1.DQ369995     GCCA------ ---------- --------AT TTTTATTTGA CTAGCGGAGG CTAGGA-GGA GAGAG--ATG GGTGCGAGAG CG
C.ZA.03.03ZAPS017MB1.DQ351224     GCCA------ ---------- --------AT TTTTATTTGA CTAGCGGAGG CTAGAA-GGA GAGAG--ATG GGTGCGAGAG CG
C.ZA.03.03ZAPS020MB1.DQ275653     GCCAATT--- ---------- ---------- --TTATTTGA CTAGCGGAGG CTAGAA-GGA GAGAG--ATG GGTGCGAGAG CG
C.ZA.03.03ZAPS021MB1.DQ369978     GCCGTTT--- ---------- ---------- --TTATTTGA CTAGCGGAGG CTAGAA-GGA GAGAG--ATG GGTGCGAGAG CG
C.ZA.03.03ZAPS023MB1.DQ351225     GCCTTTT--- ---------- ---------- ---ATTTTGA CTAGCGGAGG CTAGAA-GGA GAGAG--ATG GGTGCGAGAG CG
C.ZA.03.03ZAPS024MB1.DQ396367     GCCGAAT--- ---------- ---------- -TTTATTTGA CTAGCGGAGG CTAGAA-GGA GAGAG--ATG GGTGCGAGAG CG
C.ZA.03.03ZAPS025MB1.DQ351226     GCCAAAT--- ---------- ---------- -TTTATTTGA CTAGCGGAGG CTAGAA-GGA GAGAG--ATG GGTGCGAGAG CG
C.ZA.03.03ZAPS026MB1.DQ369985     GCCA------ ---------- --------AT TTTTATTTGA CTAGCGGAGG CTAGAA-GGA GAGAG--ATG GGTGCGAGTG CG
C.ZA.03.03ZAPS027MB1.DQ351223     GCCAAAT--- ---------- ---------- -TTTATTTGA CTAGCGGAGG CTAGAA-GGA GAGAG--ATG GGTGCGAGAG CG
C.ZA.03.03ZAPS030MB1.DQ369996     GCCA------ ---------- --------AT TTTTATTTGA CTAGCGGAGG CTAGAA-GGA GAGAG--ATG GGTGCGAGAG CG
C.ZA.03.03ZAPS032MB1.DQ445633     GCCGAAT--- ---------- ---------- TTTATTTTGA CTAGCGGAGG CTAGAA-GGA GAGAG--ATG GGTGCGAGAG CG
C.ZA.03.03ZAPS034MB1.DQ369979     GCCGAAT--- ---------- ---------- -TTTATTTGA CTAGCGGAGG CTAGAA-GGA GAGAG--ATG GGTGCGAGAG CG
C.ZA.03.03ZAPS042MB1.DQ369977     GCCGAAT--- ---------- ---------- -TTTATTTGA CTAGCGGAGG CTAGAA-GGA GAGAG--ATG GGTGCGAGAG CG
C.ZA.03.03ZAPS043MB1.DQ351227     GCCAAAT--- ---------- ---------- -TTTATTTGA CTAGCGGAGG CTAGAA-GGA GAGAG--ATG GGTGCGAGAG CG
C.ZA.03.03ZAPS044MB1.DQ396384     GCCGAAA--- ---------- ---------- TTTTATTTGA CTAGCGGAGG CTAGAA-GGA GAGAG--ATG GGTGCGAGAG CG
C.ZA.03.03ZAPS046MB1.DQ369984     GCCGAAT--- ---------- ---------- TTTTATTTGA CTAGCGGAGG CTAGAA-GGA GAGAG--ATG GGTGCGAGAG CG
C.ZA.03.03ZAPS048MB1.DQ396364     GCCAAGT--- ---------- ---------- -TTATTTTGA CTAGCGGAGG CTAGAA-GGA GAGAG--ATG GGTGCGAGAG CG
C.ZA.03.03ZAPS049MB1.DQ369986     GCCA------ ---------- --------AT TTTTATTTGA CTAGCAGAGG CTAGAA-GGA GAGAG--ATG GGTGCGAGAG CG
C.ZA.03.03ZAPS050MB1.DQ369980     GCCAAAT--- ---------- ---------- -TTTATTTGA CTAGCGGAGG CTAGAA-GGA GAGAG--ATG GGTGCGAGAG CG
C.ZA.03.03ZAPS051MB1.DQ396385     GCCAAAT--- ---------- ---------- -TTTATTTGA CTAGCGGAGG CTAGAA-GGA GAGAG--ATG GGTGCGAGAG CG
C.ZA.03.03ZAPS052MB1.DQ369987     GCCATTT--- ---------- ---------- --TTATTTGA CTAGCGGAGG CTAGAA-GGA GAGAG--ATG GGTGCGAGAG CG
C.ZA.03.03ZAPS054MB2.DQ369988     GCCAAAA--- ---------- ---------- TTTTATTTGA CTAGCGGAGG CTAGGA-GGA GAGAG--ATG GGTGCGAGAG CG
C.ZA.03.03ZAPS055MB1.DQ396373     GCCAAAT--- ---------- ---------- -TTTCTTTGA CTAGCGGAGG CTAGAA-GGA GAGAG--ATG GGTGCGAGAG CG
C.ZA.03.03ZAPS056MB1.DQ396374     GCCAATT--- ---------- ---------- --TTATTTGA CTAGCGGAGG CTAGAA-GGA GAGAG--ATG GGTGCGAGAG CG
C.ZA.03.03ZAPS057MB2.DQ369989     GCCA------ ---------- --------AT TTTTATTTGA CTAGCGGAGG CTAGAA-GGA GAGAG--ATG GGTGCGAGAG CG
C.ZA.03.03ZAPS059MB2.DQ445634     GCCAAAT--- ---------- ---------- -TTTATTTGA CTAGCGGAGG CTAGAA-GGA GAGAG--ATG GGTGCGAGAG CG
C.ZA.03.03ZAPS063MB1.DQ396388     GCCA------ ---------- --------AT TTTTATTTGA CTAGCAGAGG CTAGAA-GGA GAGAG--ATG GGTGCGAGAG CG
C.ZA.03.03ZAPS066MB2.DQ396375     GCCAATT--- ---------- ---------- --TTATTTGA CTAGCGGAGG CTAGAA-GGA GAGAG--ATG GGTGCGAGAG CG
C.ZA.03.03ZAPS067MB2.DQ396389     GCCAAAT--- ---------- ---------- -TTTATTTGA CTAGCGGAGG CTAGAA-GGA GAGAG--ATG GGTGCGAGAG CG
C.ZA.03.03ZAPS071MB1.DQ396376     GCCA------ ---------- --------AT TTTTATTTGA CTAGCGGAGG CTAGAA-GGA GAGAG--ATG GGTGCGAGAG CG
C.ZA.03.03ZAPS073MB1.DQ275649     GCCAAAT--- ---------- ---------- -TTTATTTGA CTAGCGGAGG CTAGAA-GGA GAGAG--ATG GGTGCGAGAG CG
C.ZA.03.03ZAPS074MB2.DQ351228     GCCAAAA--- ---------- ---------- TTTTATTTGA CTAGCGGAGG CTAGAA-GGA GAGAG--ATG GGTGCGAGAG CG
C.ZA.03.03ZAPS077B1.DQ093591      GCCGAAT--- ---------- ---------- -TTTATTTGA CTAGCGGAGG CTAGAA-GGA GAGAG--ATG GGTGCGAGTG CG
C.ZA.03.03ZAPS079B1.DQ093592      GCCAAAT--- ---------- ---------- -TTTATTTGA CTAGCGGAGG CTAGGA-GGA GAGAG--ATG GGTGTGAGAG CG
C.ZA.03.03ZAPS081MB1.DQ351219     GCCAATT--- ---------- ---------- --TTATTTGA CTAGCGGAGG CTAGAA-GGA GAGAG--ATG GGTGCGAGAG CG
C.ZA.03.03ZAPS083MB1.DQ351229     GCCAAAT--- ---------- ---------- -TTTATTTGA CTAGCAGAGG CTAGAA-GGA GAGAG--ATG GGTGCGAGAG CG
C.ZA.03.03ZAPS086MB1.DQ275654     GCCA------ ---------- --------AT TTTTATTTGA CTAGCGGAGG CTAGAA-GGA GAGAG--ATG GGTGCGAGAG CG
C.ZA.03.03ZAPS088MB1.DQ275651     GCCAATT--- ---------- ---------- TTTATTTTGA CTAGCGGAGG CTAGAA-GGA GAGAG--ATG GGTGCGAGAG CG
C.ZA.03.03ZAPS089MB1.DQ351216     GCCAAAT--- ---------- ---------- --TTATTGGA CTAGCGGAGG CTAGAA-GGA GAGAG--ATG GGTGCGAGAG CG
C.ZA.03.03ZAPS091MB1.DQ275645     GCCA------ ---------- --------AT TTTTATTTGA CTAGCGGAGG CTAGAA-GGA GAGAG--ATG GGTGCGAGAG CG
C.ZA.03.03ZAPS094MB1.DQ396377     GCCAAAT--- ---------- ---------- -TTTATTTGA CTAGCGGAGG CTAGGA-GGA GAGAG--ATG GGTGCGAGAG CG
C.ZA.03.03ZAPS095MB1.DQ275652     GCCAAAT--- ---------- ---------- -TTCATTTGA CTAGCGGAGG CTAGGA-GGA GAGAG--ATG GGTGCGAGAG CG
C.ZA.03.03ZAPS097MB1.DQ351230     GCCGATT--- ---------- ---------- --TTATTTGA CTAGCGGAGG CTAGAA-GGA GAGAG--ATG GGTGCGAGAG CG
C.ZA.03.03ZAPS099MB1.DQ275655     GCCA------ ---------- --------AT TTTTATTTGA CTAGCGGAGG CTAGAA-GGA GAGAG--ATG GGTGCGAGAG CG
C.ZA.03.03ZAPS103MB2.DQ275656     GCCAAAA--- ---------- ---------- TTTTATTTGA CTAGCGGAGG CTAGAA-GGA GAGAG--ATG GGTGCGAGAG CG
C.ZA.03.03ZAPS104MB1.DQ369990     GCCA------ ---------- --------AT TTTTATTTGA CTAGCGGAGG CTAGAA-GGA GAGAG--ATG GGTGCGAGAG CG
C.ZA.03.03ZAPS105MB2.DQ445632     GCCAAAT--- ---------- ---------- -TTTATTTGA CTAGCGGAGG CTAGAA-GGA GAGAG--ATG GGTGCGAGAG CG
C.ZA.03.03ZAPS108MB1.DQ396378     GCCAATT--- ---------- ---------- --TTATTTGA CTAGCGGAGG CTAGAA-GGA GAGAG--ATG GGTGCGAGAG CG
C.ZA.03.03ZAPS112MB2.DQ396386     GCCA------ ---------- ---------- ATTTTTTTGA CTAGCGGAGG CTAGAA-GGA GAGAG--ATG GGTGCGAGAG CG
C.ZA.03.03ZAPS113MB2.DQ396365     GCCAAAT--- ---------- ---------- TTTATTTTGA CTAGCGGAGG CTAGAA-GGA GAGAG--ATG GGTGCGAGAG CG
C.ZA.03.03ZAPS116MB1.DQ445635     GCCAAAT--- ---------- ---------- -TTTATTTGA CTAGCGGAGG CTAGGA-GGA GAGAG--ATG GGTGCGAGAG CG
C.ZA.03.03ZAPS118MB1.DQ396368     GCCGAAT--- ---------- ---------- -TTTATTTGA CTAGCGGAGG CTAGAA-GGA GAGAG--ATG GGTGCGAGAG CG
C.ZA.03.03ZAPS122MB1.DQ396370     GCCGAAT--- ---------- ---------- -TTTATTTGA CTAGCGGAGG CTAGGA-GGA GAGAG--ATG GGTGCGAGAG CG
C.ZA.03.03ZAPS123MB1.DQ396369     GCCAAAT--- ---------- ---------- -TTTATTTGA CTAGCGGAGG CTAGAA-GGA GAGAG--ATG GGTGCGAGAG CG
```

```
C.ZA.03.03ZAPS124MB1.DQ369976    GCCA------ ---------- --------AT TTTTATTTGA CCAGCGGAGG CTAGAA-GGA GAGAG--ATG GGTGCGAGAG CG
C.ZA.03.03ZAPS125MB1.DQ396390    GCCAAAT--- ---------- ---------- TTTATTTTGA CTAGCGGAGG CTAGAA-GGA GAGAG--ATG GGTGCGAGAG CG
C.ZA.03.03ZAPS126MB1.DQ275657    GCCAAAT--- ---------- ---------- -TTTATTTGA CTAGCGGAGG CTAGAA-GGA GAGAG--ATG GGTGCGAGAG CG
C.ZA.03.03ZAPS128MB1.DQ275643    GCCAATT--- ---------- ---------- --TTATTTGA CTAGCGGTGG CTAGAA-GGA GAGAG--ATG GGTGCGAGAG CG
C.ZA.03.03ZAPS130MB1.DQ275658    GCCA------ ---------- --------AT TTTTATTTGA CTAGCGGAGG CTAGAA-GGA GAGAG--ATG GGTGCGAGAG CG
C.ZA.03.03ZAPS131MB1.DQ396380    GCCAAAT--- ---------- ---------- -TTTATTTGA CTAGCGGAGG CTAGAA-GGA GAGAG--ATG GGTGCGAGAG CG
C.ZA.03.03ZAPS133MB1.DQ275646    GCCAAAT--- ---------- ---------- -TTTATTTGA CTAGCGGAGG CTAGAA-GGA GAGAG--ATG GGTGCGAGAG CG
C.ZA.03.03ZAPS136MB1.DQ351231    GCCAAAT--- ---------- ---------- -TTTATTTGA CTAGCGGAGG CTAGGA-GGA GAGAG--ATG GGTGCGAGAG CG
C.ZA.03.03ZAPS140MB1.DQ369981    GCCAAAT--- ---------- ---------- -TTTATTTGA CTAGCGGAGG CTAGAA-GGA GAGAG--ATG GGTGCGAGAG CG
C.ZA.03.03ZAPS143MB1.DQ396391    GCCAATT--- ---------- ---------- --TTATTTGA CTAGCGGAGG CTAGAA-GGA GAGAG--ATG GGTGCGAGAG CG
C.ZA.03.03ZAPS151MB1.DQ396392    GCCAAAT--- ---------- ---------- -TTTATTTGA CTAGCGGAGG CTAGAA-GGA GAGAG--ATG GGTGCGAGAG CG
C.ZA.03.03ZAPS152MB1.DQ396399    GCCAATT--- ---------- ---------- --TTATTTGA CTAGCGGAGG CTAGAA-GGA GAGAG--ATG GGTGCGAGAG CG
C.ZA.03.03ZAPS155MB1.DQ396371    GCCAATT--- ---------- ---------- --TTATTTGA CTAGCGGAGG CTAGAA-GGA GAGAG--ATG GGTGCGAGAG CG
C.ZA.03.03ZASK005B2.DQ011175     GCCGAAT--- ---------- ---------- -TTTATTTGA CTAGCGGAGG CTAGAA-GGA GAGAG--ATG GGTGCGAGAG CG
C.ZA.03.03ZASK006B2.AY878056     GCCAAAT--- ---------- ---------- -TTTATTTGA CTAGCGGAGG CTAGAA-GGA GAGAG--ATG GGTGCGAGAG CG
C.ZA.03.03ZASK010B2.DQ164104     GCCA------ ---------- --------AT TTTTATTTGA CTAGCGGAGG CTAGAA-GGA GAGAG--ATG GGTGCGAGAG CG
C.ZA.03.03ZASK011B2.AY901965     GCCAAAT--- ---------- ---------- -TTTATTTGA CTAGCGGAGG CTAGAA-GGA GAGAG--ATG GGTGCGAGAG CG
C.ZA.03.03ZASK013B2.DQ275660     GCCG------ ---------- --------AT TTTTATTTGA CTAGCGGAGG CTAGAA-GGA GAGAG--ATG GGTGCGAGTG CG
C.ZA.03.03ZASK016MB2.DQ351233    GCCAAAT--- ---------- ---------- --TTATTTGA CTAGCGGAGG CTAGAA-GGA GAGAG--ATG GGTGCGAGAG CG
C.ZA.03.03ZASK019B2.AY878063     GCCA------ ---------- --------AT TTTATTTTGA CTAGCGGAGG CTAGAG-GGA GAGAG--ATG GGTGCGAGAG CG
C.ZA.03.03ZASK020B2.AY878064     GCCA------ ---------- --------TT TTTTATTTGA CTAGCGGAGG CTAGAA-GGA GAGAG--ATG GGTGCGAGAG CG
C.ZA.03.03ZASK026B2.DQ011165     GCCAATT--- ---------- ---------- --TTATTTGA CTAGCGGAGG CTAGAA-GGA GAGAG--ATG GGTGCGAGAG CG
C.ZA.03.03ZASK034B1.AY878065     GCCAAAT--- ---------- ---------- -TTTATTTGA CTAGCGGAGG CTAGAA-GGA GAGAG--ATG GGTGCGAGAG CG
C.ZA.03.03ZASK036B1.AY901966     GCCT------ ---------- ---------- -TTTATTTGA CTAGCGGAGG CTAGAA-GGA GAGAG--ATG GGTGCGAGAG CG
C.ZA.03.03ZASK039B2.AY878068     GCCAATT--- ---------- ---------- --TTATTTGA CTAGTGGAGG CTAGGA-GGA GAGAG--ATG GGTGCGAGAG CG
C.ZA.03.03ZASK058B2.AY901967     GCCA------ ---------- --------AT TTTTATTTGA CTAGCGGAGG CTAGAA-GGA GAGAG--ATG GGTGCGAGAG CG
C.ZA.03.03ZASK061B1.AY901968     GCCAATT--- ---------- ---------- --TTATTTGA CTAGCGGAGG CTAGAA-GGA GAGAG--ATG GGTGCGAGAG CG
C.ZA.03.03ZASK062B1.DQ164113     GCCAAAT--- ---------- ---------- -TTTATTTGA CTAGCGGAGG CTAGAA-GGA GAGAG--ATG GGTGCGAGAG CG
C.ZA.03.03ZASK066B1.AY901969     GCCAATT--- ---------- ---------- --TTATTTGA CTAGCGGAGG CTAGAA-GGA GAGAG--ATG GGTGCGAGAG CG
C.ZA.03.03ZASK067B1.DQ275642     GCCAATT--- ---------- ---------- --TTATTTGA CTAGCGGAGG CTAGAA-GGA GAGAG--ATG GGTGCGAGAG CG
C.ZA.03.03ZASK072B1.DQ093593     GCCA------ ---------- --------AT TTTTATTTGA CTAGCGGAGG CTAGAA-GGA GAGAG--ATG GGTGCGAGAG CG
C.ZA.03.03ZASK073B1.AY901970     GCCAATT--- ---------- ---------- --TTATTTGA CTAGCGGAGG CTAGAA-GGA GAGAG--ATG GGTGCGAGAG CG
C.ZA.03.03ZASK076B1.AY901975     GCCA------ ---------- --------AT TTTTATTTGA CTAGCGGAGG CTAGAA-GGA GAGAG--ATG GGTGCGAGAG CG
C.ZA.03.03ZASK078B1.AY901971     GCCAAAT--- ---------- ---------- -TTTATTTGA CTAGCGGAGG CTAGAA-GGA GAGAG--ATG GGTGCGAGAG CG
C.ZA.03.03ZASK084B1.AY901981     GCCG------ ---------- --------AT TTTATTTTGA CTAGCGGAGG CTAGAA-GGA GAGAG--ATG GGTGCGAGAG CG
C.ZA.03.03ZASK092B1.AY878057     GCCAAAT--- ---------- ---------- -TTTATTTGA CTAGCGGAGG CTAGAA-GGA GAGAG--ATG GGTGCGAGAG CG
C.ZA.03.03ZASK094B1.AY878070     GCCG------ ---------- --------AT TTTTATTTGA CTAGCGGAGG CTAGAA-GGA GAGAG--ATG GGTGCGAGAG CG
C.ZA.03.03ZASK097B1.AY878060     GCCA------ ---------- --------AT TTTTATTTGA CCAGCGGAGG CTAGAA-GGA GAGAG--ATG GGTGCGAGAG CG
C.ZA.03.03ZASK098B1.AY878061     GCCA------ ---------- --------AT TTTTATTTGA CTAGCGGAGG CTAGAA-GGA GAGAG--ATG GGTGCGAGAG CG
C.ZA.03.03ZASK103B1.DQ164106     GCCAAAT--- ---------- ---------- -TTTATTTGA CTAGCGGAGG CTAGAA-GGA GAGAG--ATG GGTGCGAGAG CG
C.ZA.03.03ZASK104B1.DQ396395     GCCAAAT--- ---------- ---------- -TTTATTTGA CTAGCGGAGG CTAGAA-GGA GAGAG--ATG GGTGCGAGAG CG
C.ZA.03.03ZASK107B1.DQ056410     GCCGAAT--- ---------- ---------- -TTTATTTGA CTAGCGGAGG CTAGAA-GGA GAGAG--ATG GGTGCGAGAG CG
C.ZA.03.03ZASK110B1.DQ056411     GCCAAAT--- ---------- ---------- -TTTATTTGA CTAGCGGAGG CTAGAA-GGA GAGAG--ATG GGTGCGAGTG CG
C.ZA.03.03ZASK111B1.DQ056404     GCCAAAT--- ---------- ---------- -TTTATTTGA CTAGCGGAGG CTAGAA-GGA GAGAG--ATG GGTGCGAGAG CG
C.ZA.03.03ZASK113B1.DQ351237     GCCA------ ---------- --------AT TTTTATTTGA CTAGCGGAGG CTAGAA-GGA GAGAG--ATG GGTGCGAGAG CG
C.ZA.03.03ZASK117B1.DQ056408     GCCA------ ---------- --------AT TTTTATTTGA CTAGCGGAGG CTAGAA-GGA GAGAG--ATG GGTGCGAGAG CG
C.ZA.03.03ZASK118B1.DQ011169     GCCA------ ---------- ---------- TTTTATTTGA CTAGCGGAGG CTAGAA-GGA GAGAG--ATG GGTGCGAGAG CG
C.ZA.03.03ZASK120B1.DQ011176     GCCA------ ---------- --------AT TTTTATTTGA CTAGCGGAGG CTAGAA-GGA GAGAG--ATG GGTGCGAGTG CG
C.ZA.03.03ZASK211B1.DQ093601     GCCA------ ---------- --------AT TTTTATTTGA CTAGCGGAGG CTAGAA-GGA GAGAG--ATG GGTGCGAGAG CG
C.ZA.03.03ZASK212B.DQ978981      GCCAAAT--- ---------- ---------- -TTTATTTGA CTAGCGGAGG CTAGAA-GGA GAGAG--ATG GGTGCGAGAG CG
C.ZA.03.03ZASK213B1.DQ093607     GCCAAAT--- ---------- ---------- -TTTATTTGA CTAGCGGAGG CTAGAA-GGA GAGAG--ATG GGTGCGAGAG CG
C.ZA.03.03ZASK215M6W.DQ275661    GCCAATT--- ---------- ---------- --TTATTTGA CTAGCGGAGG CTAGAA-GGA GAGAG--ATG GGTGCGAGAG CG
C.ZA.03.03ZASK223B1.DQ093597     GCCGAAT--- ---------- ---------- -TTTATTTGA CTAGCGGAGG CTAGGA-GGA GAGAG--ATG GGTGCGAGAG CG
C.ZA.03.03ZASK224MB1.DQ275664    GCCA------ ---------- --------AT TTTTATTTGA CTAGCGGAGG CTAGAA-GGA GAGAG--ATG GGTGCGAGAG CG
C.ZA.03.03ZASK226B1.DQ164108     GCCAATT--- ---------- ---------- --TTATTTGA CTAGCGGAGG CTAGAA-GGA GAGAG--ATG GGTGCGAGAG CG
C.ZA.03.03ZASK232B1.DQ093589     GCCAAAC--- ---------- ---------- TTTTATTTGA CTAGCGGAGG CTAGAA-GGA GAGAG--ATG GGTGCGAGAG CG
C.ZA.03.03ZASK233B1.DQ351234     GCCA------ ---------- --------AT TTTTATTTGA CTAGCGGAGG CTAGAA-GGA GAGAG--ATG GGTGCGAGAG CG
C.ZA.03.04ZAPS194MB1.DQ275650    GCCAAAT--- ---------- ---------- -TTTATTTGA CTAGCGGAGG CTAGAA-GGA GAGAG--ATG GGTGCGAGAG CG
C.ZA.03.04ZASK082B1.AY901972     GCCT------ ---------- ---------- -TTTATTTGA CTAGCGGAGG CTAGAA-GGA GAGAG--ATG GGTGCGAGAG CG
C.ZA.03.SK023B2.AY772690         GCCA------ ---------- --------AT TTTTATTTGA CTAGCGGAGG CTAGAA-GGA GAGAG--ATG GGTGCGAGAG CG
C.ZA.03.SK029B2.AY772691         GCCAAAT--- ---------- ---------- -TTTATTTGA CTAGCGGAGG CTAGAA-GGA GAGAG--ATG GGTGCGAGAG CG
C.ZA.03.SK033B2.AY772692         GCCAATT--- ---------- ---------- --TTATTTGA CTAGCGGAGG CTAGAA-GGA GAGAG--ATG GGTGCGAGAG CG
C.ZA.03.SK040B1.AY703908         GCCA------ ---------- ---------- TTTTATTTGA CTAGCAGAGG CTAGAA-GGA GAGAG--ATG GGTGCGAGAG CG
C.ZA.03.SK041B1.AY772693         GCCAATT--- ---------- ---------- --TTATTTGA CTAGCGGAGG CTAGAA-GGA GAGAG--ATG GGTGCGAGAG CG
C.ZA.03.SK043B1.AY772700         GCCAAAT--- ---------- ---------- -TTTACTTGA CTAGCGGAGG CTAGAA-GGA GAGAG--ATG GGTGCGAGAG CG
C.ZA.03.SK065B1.AY772694         GCCA------ ---------- --------AT TTTTATTTGA CTAGCGGAGG CTAGAA-GGA GAGAG--ATG GGTGCGAGAG CG
C.ZA.03.SK112B1.AY772695         GCCAATT--- ---------- ---------- --TTATTTGA CTAGCGGAGG CTAGAA-GGA GAGAG--ATG GGTGCGAGAG CG
C.ZA.03.SK116B1.AY772696         GCCAAAT--- ---------- ---------- -TTTATTTGA CTAGCGGAGG CTAGAA-GGA GAGAG--ATG GGTGCGAGAG CG
C.ZA.04.04ZAPS157MB1.DQ351232    GCCATTT--- ---------- ---------- --TTATTTGA CTAGCGGAGG CTAGAA-GGA GAGAG--ATG GGTGCGAGAG CG
C.ZA.04.04ZAPS160B1.DQ164107     GCCTAAT--- ---------- ---------- -TTTATTTGA CTAGCGGAGG CTAGGA-GGA GAGAG--ATG GGTGCGAGAG CG
C.ZA.04.04ZAPS161B1.DQ164115     GCCA------ ---------- --------AT TTTTATTTGA CTAGCGGAGG CTAGAA-GGA GAGAG--ATG GGTGCGAGAG CG
C.ZA.04.04ZAPS165MB1.DQ351221    GCCA------ ---------- --------AT TTTTATTTGA CTAGCGGAGG CTAGAA-GGA GAGAG--ATG GGTGCGAGAG CG
C.ZA.04.04ZAPS168B1.DQ164121     GCCAATT--- ---------- ---------- --TTATTTGA CTAGCGGAGG CTAGAA-GGA GAGAG--ATG GGTGCGAGAG CG
C.ZA.04.04ZAPS169MB1.DQ396393    GCCAAAT--- ---------- ---------- -TTTATTTGA CTAGCGGAGG CTAGAA-GGA GAGAG--ATG GGTGCGAGAG CG
C.ZA.04.04ZAPS172MB1.DQ275659    GCCAAAT--- ---------- ---------- -TTTATTTGA CTAGCGGAGG CTAGAA-GGA GAGAG--ATG GGTGCGAGAG CG
C.ZA.04.04ZAPS177MB1.DQ396394    GCCAAAA--- ---------- ---------- -TTTATTTGA CTAGCGGAGG CTAGAA-GGA GAGAG--ATG GGTGCGAGAG CG
C.ZA.04.04ZAPS188B1.DQ164122     GCCAATT--- ---------- ---------- --TTATTTGA CTAGCGGAGG CTAGAA-GGA GAGAG--ATG GGTGCGAGAG CG
C.ZA.04.04ZAPS189B1.DQ164117     GCCGAAA--- ---------- ---------- TTTTATTTGA CTAGCGGAGG CTAGGA-GGA GAGAG--ATG GGTGCGAGAG CG
C.ZA.04.04ZAPS190B1.DQ093602     GCCAATT--- ---------- ---------- --TTATTTGA CTAGCGGAGG CTAGAA-GGA GAGAG--ATG GGTGCGAGAG CG
C.ZA.04.04ZAPS195B1.DQ164118     GCCAAAT--- ---------- ---------- -TTTATTTGA CTAGCAGAGG CTAGAA-GGA GAGAG--ATG GGTGCGAGAG CG
C.ZA.04.04ZAPS197MB1.DQ369997    GCCA------ ---------- --------AT TTTTAGTTGA CTAGCGGAGG CTAGGA-GGA GAGAG--ATG GGTGCGAGAG CG
C.ZA.04.04ZAPS198MB1.DQ445637    GCCA------ ---------- --------AT TTTTATTTGA CTAGCGGAGG CTAGAA-GGA GAGAG--ATG GGTGCGAGAG CG
C.ZA.04.04ZAPS199B1.DQ164110     GCCAAAT--- ---------- ---------- -TTTATTTGA CTAGCGGAGG CTAGAA-GGA GAGAG--ATG GGTGCGAGAG CG
C.ZA.04.04ZAPS202B1.DQ093598     GCCAAAT--- ---------- ---------- TTTATTTTGA CTAGCGGAGG CTAGAA-GGA GAGAG--ATG GGTGCGAGAG CG
C.ZA.04.04ZAPS205B1.DQ093599     GCCA------ ---------- --------AT TTTTATTTGA CTAGCGGAGG CTAGAA-GGA GAGAG--ATG GGTGCGAGAG CG
C.ZA.04.04ZAPS206B1.DQ164126     GCCT------ ---------- ---------- -TTTATTTGA CTAGCGGAGG CTAGAA-GGA GAGAG--ATG GGTGCGAGAG CG
C.ZA.04.04ZAPS208B1.DQ093603     GCCAAAT--- ---------- ---------- -TTTATTTGA CTAGCGGAGG CTAGAA-GGA GAGAG--ATG GGTGCGAGAG CG
C.ZA.04.04ZAPS214B1.DQ093588     GCCAATT--- ---------- ---------- --TTATTTGA CTAGCGGAGG CTAGAA-GGA GAGAG--ATG GGTGCGAGAG CG
C.ZA.04.04ZAPS216B1.DQ093600     GCCGAAA--- ---------- ---------- TTTTATTTGA CTAGCGGAGG CTAGAA-GGA GAGAG--ATG GGTGCGAGAG CG
C.ZA.04.04ZAPS217B1.DQ164119     GCCAATT--- ---------- ---------- --TTATTTGA CTAGCGGAGG CTAGAA-GGA GAGAG--ATG GGTGCGAGAG CG
C.ZA.04.04ZASK031B2.AY901974     GCCAAAT--- ---------- ---------- -TTTATTTGA CTAGCGGAGG CTAGAA-GGA GAGAG--ATG GGTGCGAGTG CG
C.ZA.04.04ZASK083B2.DQ093585     GCCAAAT--- ---------- ---------- -TTTATTTGA CTAGCGGAGG CTAGAA-GGA GAGAG--ATG GGTGCGAGAG CG
C.ZA.04.04ZASK127B1.DQ011172     GCCGAAT--- ---------- ---------- -TTTATTTGA CTAGCGGAGG CTAGAA-GGA GAGAG--GTG GGTGCGAGAG CG
C.ZA.04.04ZASK128B1.DQ093586     GCCAAAT--- ---------- ---------- -TTTATTTGA CTAGCGGAGG CTAGGA-GGA GAGAG--ATG GGTGCGAGAG CG
C.ZA.04.04ZASK131B1.DQ056412     GCCAATT--- ---------- ---------- TTTTATTTGA CTAGCGGAGG CTAGAA-GGA GAGAG--ATG GGTGCGAGTG CG
C.ZA.04.04ZASK132B1.DQ093594     GCCAAAT--- ---------- ---------- -TTTATTTGA CTAGCGGAGG CTAGGA-GGA GAGAG--ATG GGTGCGAGAG CG
C.ZA.04.04ZASK135B1.DQ011166     GCCA------ ---------- --------AT TTTATCTTGA CTAGCGGAGG CTAGAA-GGA GAGAG--ATG GGTGCGAGAG CG
C.ZA.04.04ZASK136B1.DQ011177     GCCGAAT--- ---------- ---------- -TTTATTTGA CTAGCGGAGG CTAGAA-GGA GAGAG--ATG GGCGCGAGAG CG
C.ZA.04.04ZASK139B1.AY878072     GCCGAAT--- ---------- ---------- -TTTATTTGA CTAGCGGAGG CTAGAA-GGA GAGAG--ATG GGTGCGAGAG CG
C.ZA.04.04ZASK142B1.DQ011167     GCCAATT--- ---------- ---------- --TTATTTGA CTAGCAGAGG CTAGAA-GGA GAGAG--ATG GGTGCGAGAG CG
C.ZA.04.04ZASK145B1.AY901976     GCCATAT--- ---------- ---------- -TTTATTTGA CTAGCGGAGG CTAGAA-GGA GAGAG--ATG GGTGCGAGAG CG
C.ZA.04.04ZASK146.AY772699       GCCA------ ---------- --------AT TTTTATTTGA CTAGCGGAGG CTAGAA-GGA GAGAG--ATG GGTGCGAGAG CG
C.ZA.04.04ZASK147B1.DQ164114     GCCAATT--- ---------- ---------- --TTATTTGA CTAGCGGAGG CTAGAA-GGA GAGAG--ATG GGTGCGAGAG CG
C.ZA.04.04ZASK148B1.DQ093590     GCCAACT--- ---------- ---------- -TTTATTTGA CTAGCGGAGG CTAGAA-GGA GAGAG--ATG GGTGCGAGAG CG
C.ZA.04.04ZASK150B1.DQ011178     GCCAATT--- ---------- ---------- --TTATTTGA CTAGCGGAGG CTAGAA-GGA GAGAG--ATG GGTGCGAGAG CG
```

```
C.ZA.04.04ZASK151B1.AY901977     GCCAAAT--- ---------- ---------- -TTTATTTGA CTAGCGGAGG CTAGAA-GGA GAGAG--ATG GGTGCGAGTG CG
C.ZA.04.04ZASK154B1.AY878071     GCCAATT--- ---------- ---------- --TTATTTGA CTAGCGGAGG CTAGAA-GGA GAGAG--ATG GGTGCGAGAG CG
C.ZA.04.04ZASK155B1.AY901978     GCCAATT--- ---------- ---------- --TTATTTGA CTAGCGGAGG CTAGAA-GGA GAGAG--ATG GGTGCGAGAG CG
C.ZA.04.04ZASK156B1.DQ011171     GCCAAAA--- ---------- ---------T TTTTATTTGA CTAGCGGAGG CTAGAA-GGA GAGAG--ATG GGTGCGAGAG CG
C.ZA.04.04ZASK159B1.DQ011179     GCCA------ ---------- --------AT TTTTATTTGA CTAGCGGAGG CTAGAA-GGA GAGAG--ATG GGTGCGAGAG CG
C.ZA.04.04ZASK160B1.DQ011173     GCCAATT--- ---------- ---------- --TTATTTGA CTAGCGGAGG CTAGAA-GGA GAGAG--ATG GGTGCGAGAG CG
C.ZA.04.04ZASK161B1.DQ011170     GCCAAAT--- ---------- ---------- -TTCATTTGA CTAGCGGAGG CTAGAA-GGA GAGAG--ATG GGTGCGAGAG CG
C.ZA.04.04ZASK163B1.AY901979     GCCA------ ---------- --------AT TTTTATTTGA CTAGCGGGGG CTAGAA-GGA GAGAG--ATG GGTGCGAGAG CG
C.ZA.04.04ZASK164B1.DQ056405     GCCA------ ---------- --------AT TTTTATTTGA CTAGCGGAGG CTAGAA-GGA GAGAG--ATG GGTGCGAGAG CG
C.ZA.04.04ZASK165B1.DQ396387     GCCAAAT--- ---------- ---------- -TTTTCTTGA CTAGCGGAGG CTAGAA-GGA GAGAG--ATG GGTGCGAGAG CG
C.ZA.04.04ZASK167B1.DQ164127     GCCAAAT--- ---------- ---------- --TTATTTGA CTAGCGGAGG CTAGAA-AGA GAGAG--ATG GGTGCGAGAG CG
C.ZA.04.04ZASK168B1.AY878058     GCCGAAT--- ---------- ---------- -TTTATTTGA CTAGCGGAGG CTAGAA-GGA GAGAG--ATG GGTGCGAGAG CG
C.ZA.04.04ZASK169B1.DQ396381     GCCA------ ---------- --------AT TTTTATTTGA CTAGCGGAGG CTAGAA-GGA GAGAG--ATG GGTGCGAGAG CG
C.ZA.04.04ZASK170B1.DQ093595     GCCAAAT--- ---------- ---------- TTTCTTTTGA CTAGCGGAGG CTAGAA-GGA GAGAG--ATG GGTGCGAGAG CG
C.ZA.04.04ZASK171B1.DQ351217     GCCAAAT--- ---------- ---------- -TTTATTTGA CTAGCGGAGG CTAGAA-GGA GAGAG--ATG GGTGCGAGCG CG
C.ZA.04.04ZASK172B1.DQ369998     GCCA------ ---------- --------AT TTTTATTTGA CTAGCGGAGG CTAGAA-GGA GAGAG--ATG GGTGCGAGAG CG
C.ZA.04.04ZASK173B1.DQ093604     GCCA------ ---------- --------AT TTCTATTTGA CTAGCGGAGG CTAGAA-GGA GAGAG--ATG GGTGCGAGAG CG
C.ZA.04.04ZASK174B1.AY901980     GCCA------ ---------- --------AT TTTTATTTGA CTAGCGGAGG CTAGGA-GGA GAGAG--ATG GGTGCGAGAG CG
C.ZA.04.04ZASK175B1.DQ164129     GCCGAAT--- ---------- ---------- -TTTATTTGA CTAGCGGAGG CTAGAA-GGA GAGAG--ATG GGTGCGAGAG CG
C.ZA.04.04ZASK176B1.DQ056416     GCCGAAT--- ---------- ---------- --TTTATTGA CTAGCGGAGG CTAGAA-GGA GAGAG--ATG GGTGCGAGAG CG
C.ZA.04.04ZASK178B1.DQ093587     GCCAAAT--- ---------- ---------- -TTTATTTGA CTAGCGGAGG CTAGAA-GGA GAGAG--ATG GGTGCGAGCG CG
C.ZA.04.04ZASK180B1.AY878059     GCCAAAT--- ---------- ---------- -TTTATTTGA CTAGCGGAGG CTAGAA-GGA GAGAG--ATG GGTGCGAGAG CG
C.ZA.04.04ZASK181B1.AY878062     GCCTAAT--- ---------- ---------- -TTTATTTGA CTAGCGGAGG CTAGAA-GGA GAGAG--ATG GGTGCGAGAG CG
C.ZA.04.04ZASK182B1.AY878054     GCCGAAT--- ---------- ---------- -TTTATTTGA CTAGCGGAGG CTAGAA-GGA GAGAG--ATG GGTGCGAGAG CG
C.ZA.04.04ZASK183B1.AY878055     GCCAAAT--- ---------- ---------- -TTTATTTGA CTAGCGGAGG CTAGAA-GGA GAGAG--ATG GGTGCGAGAG CG
C.ZA.04.04ZASK184B1.DQ056418     GCCATTT--- ---------- ---------- --TTATTTGA CTAGCGGAGG CTAGAA-GGA GAGAG--ATG GGTGCGAGAG CG
C.ZA.04.04ZASK185B1.DQ011174     GCCGAAT--- ---------- ---------- -TTTATTTGA CTAGCGGAGG CTAGAA-GGA GAGAG--ATG GGTGCGAGAG CG
C.ZA.04.04ZASK190B1.DQ056409     GCCAAAT--- ---------- ---------- -TTTATTTGA CTAGCGGAGG CTAGAA-GGA GAGAG--ACG GGTGCGAGAG CG
C.ZA.04.04ZASK191B1.DQ369993     GCCAATT--- ---------- ---------- --TTATTTGA CTAGCGGAGG CTAGAA-GGA GAGAG--ATG GGTGCGAGAG CG
C.ZA.04.04ZASK192B1.DQ396382     GCCA------ ---------- --------AT TTTATTTTGA CTAGCAGAGG CTAGAA-GGA GAGAG--ATG GGTGCGAGAG CG
C.ZA.04.04ZASK193B1.DQ396396     GCCAAAT--- ---------- ---------- -TTTATTTGA CTAGCGGAGG CTAGAA-GGA GAGAG--ATG GGTGCGAGAG CG
C.ZA.04.04ZASK196B1.DQ056413     GCCA------ ---------- --------AT TTTTATTTGA CTAGCGGAGG CTAGAA-GGA GAGAG--ATG GGTGCGAGAG CG
C.ZA.04.04ZASK200B1.DQ396383     GCCAAAT--- ---------- ---------- -TTTATTTGA CTAGCGGAGG CTAGAA-GGA GAGAG--ATG GGTGCGAGAG CG
C.ZA.04.04ZASK201B1.DQ396397     GCCA------ ---------- --------AT TTTTATTTGA CTAGCGGAGG CTAGAA-GGA GAGAG--ATG GGTGCGAGAG CG
C.ZA.04.04ZASK202B1.DQ011180     GCCTAAT--- ---------- ---------- --TTATTTGA CTAGCGGAGG CTAGAA-GGA GAGAG--ATG GGTGCGAGAG CG
C.ZA.04.04ZASK204B1.DQ056414     GCCAAAT--- ---------- ---------- -TTTATTTGA CTAGCGGAGG CTAGAA-GGA GAGAG--ATG GGTGCGAGAG CG
C.ZA.04.04ZASK206B1.DQ056415     GCCAATT--- ---------- ---------- --TTATTTGA CTAGCGGAGG CTAGAA-GGA GAGAG--ATG GGTGCGAGAG CG
C.ZA.04.04ZASK208B1.DQ056406     GCCAAAT--- ---------- ---------- --TTATTTGA CTAGCGGAGG CTAGAA-GGA GAGAG--ATG GGTGCGAGAG CG
C.ZA.04.04ZASK217B1.DQ056417     GCCA------ ---------- --------AT TTTTATTTGA CTAGCGGAGG CTAGAA-GGA GAGAG--ATG GGTGCGAGAG CG
C.ZA.04.04ZASK234B1.DQ093605     GCCGAAT--- ---------- ---------- -TTTATTTGA CTAGCGGAGG CTAGAA-GGA GAGAG--ATG GGTGCGAGAG CG
C.ZA.04.CAP30_5w_F4.GQ999973     GCCAAAT--- ---------- ---------- -TTTATTTGA CTAGCGGAGG CTAGGG-GGA GAGAG--ATG GGTGCGAGAG CG
C.ZA.04.CAP61_8w_F3.GQ999975     GCCAAAT--- ---------- ---------- -TTTATTTGA CTAGCGGAGG CTAGAA-GGA GAGAG--ATG GGTGCGAGAG CG
C.ZA.04.SK133B1.AY772698         GCCA------ ---------- --------AT TTTTATTTGA CTAGCGGAGG CTAGAA-GGA GAGAG--ATG GGTGCGAGAG CG
C.ZA.04.SK134B1.AY703909         GCCATTT--- ---------- ---------- --TTATTTGA CTAGCGGAGG CTAGAA-GGA GAGAG--ATG GGTGCGAGAG CG
C.ZA.04.SK140B1.AY901973         GCCGAAA--- ---------- ---------- TTTTATTTGA CTAGCGGAGG CTAGAA-GGA GAGAG--ATG GGTGCGAGAG CG
C.ZA.04.SK143B1.AY703910         GCCA------ ---------- --------AT TTTTATTTGA CTAGCGGAGG CTAGAA-GGA GAGAG--ATG GGTGCGAGAG CG
C.ZA.04.SK144B1.AY703911         GCCAAAA--- ---------- ---------- TTTTATTTGA CTAGCGGAGG CTAGAA-GGA GAGAG--ATG GGTGCGAGAG CG
C.ZA.05.05ZAPSK240B1.DQ369991    GCCA------ ---------- --------AT TTTTATTTGA CTAGCGGAGG CTAGAA-GGA GAGAG--ATG GGTGCGAGAG CG
C.ZA.05.05ZASK243B1.DQ396372     GCCA------ ---------- --------AT TTTTATTTGA CTAGCGGAGG CTAGAA-GGA GAGAG--ATG GGTGCGAGAG CG
C.ZA.05.05ZASK244B1.DQ369992     GCCTATT--- ---------- ---------- --TTATTTGA CTAGCGGAGG CTAGAA-GGA GAGAG--ATG GGTGCGAGAG CG
C.ZA.05.05ZASK245B1.DQ369982     GCCAAAT--- ---------- ---------- -TTTATTTGA CTAGCGGAGG CTAGAA-GGA GAGAG--ATG GGTGCGAGAG CG
C.ZA.05.05ZASK246B1.DQ369983     GCCAAAT--- ---------- ---------- -TTTATTTGA CTAGCGGAGG CTAGAA-GGA GAGAG--ATG GGTGCGAGAG CG
C.ZA.05.05ZASK247B1.DQ369994     GCCA------ ---------- --------AT TTTTATTTGA CTAGCGGAGG CTAGAA-GGA GAGAG--ATG GGTGCGAGAG CG
C.ZA.05.CAP174_4w.GQ999981       GCCAATT--- ---------- ---------- --TTCTTTGA CTAGCGGAGG CTAGAA-GGA GAGAG--ATG GGTGCGAGAG CG
C.ZA.05.CAP206_8w_F1.GQ999982    GCCAAAT--- ---------- ---------- --TTATTTGA CTAGCGGAGG CTAGAA-GGA GGGAG--ATG GGTGCGAGAG CG
C.ZA.05.CAP210_5w.GQ999983       GCCAAAT--- ---------- ---------- -TTTATTTGA CTAGCGGAGG CTAGAA-GGA GAGAG--ATG GGTGCGAGAG CG
C.ZA.05.CAP228_8w_F2.GQ999984    GCCAAAT--- ---------- ---------- -TTTATTTGA CTAGCGGAGG CTAGAA-GGA GAGAG--ATG GGTGCGAGAG CG
C.ZA.05.CAP229_7w.GQ999985       GCCAAAT--- ---------- ---------- -TTTATTTGA CTGGCGGAGG CTAGAA-GGA GAGAG--ATG GGTGCGAGAA CG
C.ZA.05.CAP239_5w_F1.GQ999991    GCCGATT--- ---------- ---------- --TTATTTGA CTAGCGGAGG CTAGAA-GGA GAGAG--ATG GGTGCGAGAG CG
C.ZA.05.CAP244_8w_F1.GQ999986    GCCATTT--- ---------- ---------- --TATTTTGA CTAGCGGAGG CTAGAA-GGA GAGAG--ATG GGTGCGAGAG CG
C.ZA.05.CAP248_9w.GQ999987       GCCAATT--- ---------- ---------- --TTATTTGA CTAGCGGAGG CTAGAA-GGA GAGAG--ATG GGTGCGAGAG CG
C.ZA.05.CAP255_8w_F1.GQ999988    GCCAAAT--- ---------- ---------- -TTTATTTGA CTAGCGGAGG CTAGAA-GGA GAGAG--ATG GGTGCGAGAG CG
C.ZA.05.CAP256_6w.GQ999989       ACCAATT--- ---------- ---------- -TTTATTTGA CTAGCGGAGG CTAGAA-GGA GAGAG--ATG GGTGCGAGAG CG
C.ZA.05.CAP257_7w_F1.GQ999990    GCCAAAA--- ---------- ---------- TTTTATTTGA CTAGCGGAGG CTAGGA-GGA GAGAG--ATG GGTGCGAGAG CG
C.ZA.05.CAP45_5w_F1.GQ999974     GCCAAAT--- ---------- ---------- --TTATTTGA CTAGCGGAGG CTAGAA-GGA GAGAG--ATG GGTGCGAGAG CG
C.ZA.05.CAP63_5w_F4.GQ999976     GCCAATT--- ---------- ---------- -TTTATTTGA CTAGCGGAGG CTAGAA-GGA GAGAG--ATG GGTGCGAGAG CG
C.ZA.05.CAP65_6w.GQ999977        GCCAAAT--- ---------- ---------- TTTTATTTGA CTAGCGGAGG CTAGAA-GGA GAGAG--ATG GGTGCGAGAG CG
C.ZA.05.CAP84_3w_F2.GQ999978     GCCAATT--- ---------- ---------- --TTATTTGA CTAGCGGGGG CTAGAA-GGA GAGAG--ATG GGTGCGAGTG CG
C.ZA.05.CAP85_5w_F1.GQ999979     GCCAAAT--- ---------- ---------- -TTTATTTGA CTAGCGGAGG CTAGAA-GGA GAGAG--ATG GGTGCGAGAG CG
C.ZA.05.CAP88_5w_F2.GQ999980     GCCGATT--- ---------- ---------- -TGTATTTGA CTAGCGGAGG CTAGAA-GGA GAGAG--ATG GGTGCGAGAG CG
C.ZA.05.CAP8_3w_F2.GQ999972      GCCAATT--- ---------- ---------- -TTTATTTGA CTAGCGGAGG CTAGAA-GGA GAGAG--ATG GGTGCGAGAG CG
C.ZA.07.DEMC07ZA011.JX140664     GCCAAAT--- ---------- ---------- --TTTTTTGA CTAGCGGAGG CTAGAA-GGA GAGAG--ATG GGTGCGAGAG CG
C.ZA.08.DEMC08ZA011.JX140666     GCCTATT--- ---------- ---------- --TTATTTGA CTAGCGGAGG CTAGAA-GGA GAGAG--ATG GGTGCGAGAG CG
C.ZA.09.DEMC09ZA008.JX140667     GCCAAAT--- ---------- ---------- -TTTATTTGA CTAGCGGAGG CTAGAA-GGA GAGAG--ATG GGTGCGAGAG CG
C.ZA.09.DEMC09ZA009.JX140668     GCCAATT--- ---------- ---------- -TTTATTTGA CTAGCGGAGG CTAGAA-GGA GAGAG--ATG GGTGCGAGAG CG
C.ZA.10.DEMC10ZA001.JX140669     GCCAAAT--- ---------- ---------- -TTTATTTGA CTAGCGGAGG CTAGAA-GGA GAGAG--ATG GGTGCGAGAG CG
C.ZA.90.pZAC_R3714.JN188292      GCCGAAT--- ---------- ---------- -TTTATTTGA CTAGCGGAGG CTAGAA-GGA GAGAG--ATG GGTGCGAGAG CG
C.ZA.97.97ZA003.AY118165         GCCAAAT--- ---------- ---------- -TTTATTTGA CTAGCGGAGG CTAGAA-GGA GAGAG--ATG GGTGCGAGAG CG
C.ZA.97.97ZA009.AY118166         GCCAAAT--- ---------- ---------- -TTTATTTGA CTAGCGGAGG CTAGAA-GGA GAGAG--ATG GGTGCGAGAG CG
C.ZA.97.97ZA012.AF286227         GCTG------ ---------- --------AT TTTTATTTGA CTAGCGGAGG CTAGAA-GGA GAGAG--ATG GGTGCGAGAG CG
C.ZA.98.98ZA445.AY158533         GCCAAAT--- ---------- ---------- -TTTATTTGA CTAGCGGAGG CTAGAA-GGA GAGAG--ATG GGTGCGAGAG CG
C.ZA.98.98ZA502.AY158534         GCCA------ ---------- --------AT TTTTATTTGA CTAGCGGAGG CTAGAA-GGA GAGAG--ATG GGTGCGAGAG CG
C.ZA.98.98ZA528.AY158535         GCCAATT--- ---------- ---------- -TTTATTTGA CTAGCGGTGG CTAGAA-GGA GAGAAG-ATG GGTGCGAGAG CG
C.ZA.98.TV001.AY162223           GCCAATT--- ---------- ---------- --TTACTTGA CTAGCGGAGG CTAGAA-GGA GAGAG--ATG GGTGCGAGAG CG
C.ZA.98.TV002.AY162224           GCCAAAA--- ---------- ---------- TTTTTTTTGA CTAGCGGAGG CTAGAA-GGA GAGAG--ATG GGTGCGAGAG CG
C.ZA.98.TV012.AY162225           GCCAAAT--- ---------- ---------- -TTTATTTGA CTAGCGGAGG CTAGAA-GGA GAGAG--ATG GGTGCGAGAG CG
C.ZA.99.99ZACM9.AF411967         GCCGAAT--- ---------- ---------- -TTTATTTGA CTAGCGGAGG CTAGAA-GGA GAGAG--ATG GGTGCGAGAG CG
C.ZA.99.99ZALT21.EU293446        GCCAAAT--- ---------- ---------- -TTTATTTGA CTAGCGGAGG CTAGAA-GGA GAGAG--ATG GGTGCGAGAG CG
C.ZA.99.99ZALT39.EU293447        GCCAAAT--- ---------- ---------- -TTTATTTGA CTAGCGGAGG CTAGAA-GGA GAGAG--ATG GGTGCGAGAG CG
C.ZA.99.99ZALT42.EU293448        GCCA------ ---------- ---------- TTTTATTTGA CTAGCGGAGG CTAGAA-GGA GAGAG--ATG GGTGCGAGAG CG
C.ZA.99.99ZALT45.EU293449        GCCAAAT--- ---------- ---------- -TTTATTTGA CTAGCGGAGG CTAGAA-GGA GAGAG--ATG GGTGCGAGAG CG
C.ZA.99.99ZALT46.EU293450        GCCA------ ---------- --------AT TTTTATTTGA CTAGCGGAGG CTAGAA-GGA GAGAG--ATG GGTGCGAGAG CG
C.ZA.99.99ZALT4.EU293444         GCCAATT--- ---------- ---------- --TTATTAGA CTAGCGGAGG CTAGAA-GGA GAGAG--ATG GGTGCGAGAG CG
C.ZA.99.99ZALT5.EU293445         GCCA------ ---------- --------AT TTTTATTTGA CTAGCGGAGG CTAGAA-GGA GAGAG--ATG GGTGCGAGAG CG
C.ZA.99.99ZATM10.AY228556        GCCA------ ---------- --------AT TTTTATTTGA CTAGCGGAGG CTAGAA-GGA GAGAG--ATG GGTGCGAGAG CG
C.ZA.99.ZASW7.AF411966           GCCAAAT--- ---------- ---------- -TTTATTTGA CTAGCGGAGG CTAGAA-GGA GAGAG--ATG GGTGCGAGAG CG
C.ZM.02.02ZM108.AB254141         GCCA------ ---------- ---------- TTTTATTTGA CTAGCGGAGG CTAGAA-GGA GAGTG--ATG GGTGCGAGAG CG
C.ZM.02.02ZM110.AB254142         GCCAATT--- ---------- ---------- --TTATTTGA CTAGCGGAGG CTAGAA-GGA GAGAG--ATG GGTGCGGGAG CG
C.ZM.02.02ZM114.AB254146         GCCCAAT--- ---------- ---------- -TTTATTTGA CTAGCGGAGG CTAGAA-GGA GAGAG--ATG GGTGCGAGAG CG
C.ZM.02.02ZM115.AB254148         GCCA------ ---------- --------AT TTTTATTTGA CTAGCGGAGG CTAGAA-GGA GAGAG--ATG GGTGCGAGAG CG
C.ZM.02.02ZMBC.AB254149          GCCAAAT--- ---------- ---------- -TTTATTTGA CTAGCGGAGG CTAGAA-GGA GAGAG--ATG GGTGCGAGAG CG
C.ZM.02.02ZMDB.AB254153          GCCAAAT--- ---------- ---------- -TTTATTTGA CTAGCGGAGG CTAGAA-GGA GAGAG--ATG GGTGCGAGAG CG
C.ZM.02.02ZMJC.AB254155          GCCATTT--- ---------- ---------- --TATTTTGA CTAGCGGAGG CTAGAA-GGA GAGAG--ATG GGTGCGAGAG CG
C.ZM.03.ZM246F_flA10.FJ496186    GCCA------ ---------- --------AT TTTTATTTGA CTAGCGGAGG CTAGAA-GGA GAGAG--ATG GGTGCGAGAG CG
C.ZM.03.ZM247F_flA1.FJ496195     GCCGAAT--- ---------- ---------- TTTTGTCTGA CTAGCGGAGG CTAGAA-GGA GAGAG--ATG GGTGCGAGAG CG
```

C.ZM.03.ZM249M_flC1.FJ496209	GCCAAAT---	----------	----------	-TTTATTTGA	CTAGCGGAGG	CTAGAA-GGA	GAGAG--ATG	GGTGCGAGAG	CG
C.ZM.89.ZAM18.AB485645	GCCA------	----------	--------AT	TTTTATTTGA	CTAGCGGAGG	CTAGAA-GGA	GAGAG--ATG	GGTGCGAGAG	CG
C.ZM.96.96ZM651.AF286224	GCCAAAT---	----------	----------	-TTTATTTGA	CTAGCGGAGG	CTAGAA-GGA	GAGAG--ATG	GGTGCGAGAG	CG
C.ZM.96.96ZM751.AF286225	GCCAATT---	----------	----------	--TTATTTGA	CTAGCGGAGG	CTAGAA-GGA	GAGAG--ATG	GGTGCGAGAG	CG
C.ZM.x.HIV1084i.AY805330	GCCAAAT---	----------	----------	-TTTATTTGA	CTAGCGGAGG	CTAGGA-GGA	GAGAG--ATG	GGTGCGAGAG	CG
D.CD.83.ELI.K03454	GCTAAAA---	----------	----------	---TTTTTGA	CTAGCGGAGG	CTAGAA-GGA	GAGAG--ATG	GGTGCGAGAG	CG
D.CD.83.NDK.M27323	GCTGAAA---	----------	----------	---TTTTTGA	CTAGCGGAGG	CTAGAA-GGA	GAGAG--ATG	GGTGCGAGAG	CG
D.CD.84.84ZR085.U88822	GCTGAAAA--	----------	----------	--TTTTTTGA	CTAGCGGAGG	CTAGAA-GGA	GAGAG--ATG	GGTGCGAGAG	CG
D.CD.85.Z2Z6_Z2_CDC_Z34.M22639	GCTAAAA---	----------	----------	---ATTTTGA	CTAGCGGAGG	CTAGAA-GGA	GAGAG--ATG	GGTGCGAGAG	CG
D.CM.10.DEMD10CM009.JX140670	GCTAAAA---	----------	----------	--ATTTTTGA	CTAGCAGAGG	CTAGAA-GGA	GAGAG--ATG	GGTGCGAGAG	CG
D.KE.93.MB2059.AF133821	GCTAAACA--	----------	----------	--ACTTTTGA	CTAGCGGAGG	CTAGAA-GGA	GAGAG--ATG	GGTGCGAGAG	CG
D.KE.97.ML415_2.AY322189	GCTGAAA---	----------	----------	---TTTTTGA	CTAGCGGAGG	CTAGAA-GGA	GAGAG--ATG	GGTGCGAGAG	CG
D.KR.04.04KBH8.DQ054367	GCTAAAAA--	----------	----------	--TTTTTTGA	CTAGCAGAGG	CTAGAA-GGG	GAAAG--ATG	GGTGCAAGAG	CG
D.SN.90.SE365.AB485648	GCTGAAA---	----------	----------	---TTTTTGA	CTAGCGGAGG	CTAGAA-GGA	GAGAG--ATG	GGTGCGAGAG	CG
D.UG.05.p190049.JX236668	GCAAAAT---	----------	----------	----TTTTGA	CTAGCGGAGG	CTAGAA-GGA	GAGAG--ATG	GGTGCGAGAG	CG
D.UG.07.p191647.JX236670	GCAAAAA---	----------	----------	--TTTTTTGA	CTAGCGGAGG	CTAGAA-GGA	GAGAG--ATG	GGTGCGAGAG	CG
D.UG.07.p191882.JX236673	GCTGAAT---	----------	----------	---TTTTTGA	CTAGCGGAGG	CTAGAA-GGA	GAGAG--ATG	GGTGCGAGAG	CG
D.UG.07.pSC191727.JX236679	GCTGAAA---	----------	----------	---TTTTTGA	CTAGCGGAGG	CTAGAA-GGA	GAGAG--ATG	GGTGCGAGAG	CG
D.UG.08.p191859.JX236672	GCTGAAA---	----------	----------	---TTTTTGA	CTAGCGGAGG	CTAGAA-GGA	GAGAG--ATG	GGTGCGAGAG	CG
D.UG.91.UG270.AB485650	GCTGAAA---	----------	----------	---AATTTGA	CTAGCGGAGG	CTAGAA-GGA	GAGAG--ATG	GGTGCGAGAG	CG
D.UG.92.92UG001.AJ320484	GCTGAAA---	----------	----------	--TTTTTTGA	CTAGCGGAGG	CTAGAA-GGA	GAGAG--ATG	GGTGCGAGAG	CG
D.UG.94.94UG114.U88824	GCTAAAAA--	----------	----------	--ATTTTTGA	CTAGCGGAGG	CTAGAA-GGA	GAGAG--ATG	GGTGCGAGAG	CG
D.ZA.84.R2.AY773338	GCTAAAA---	----------	----------	---ATTTTGA	CTAGCGGAGG	CTAGAA-GGA	GAGAG--ATG	GGTGCGAGAG	CG
D.ZA.85.R286.AY773340	GCTAAAA---	----------	----------	---TTTTTGA	CTAGCGGAGG	CTAGAA-GGA	GAGAG--ATG	GGTGCGAGAG	CG
D.ZA.86.R482.AY773341	GCTAAAA---	----------	----------	---TTTTTGA	CTAGCGGAGG	CTAGAA-GGA	GAGAG--ATG	GGTGCGAGAG	CG
D.ZA.90.R1.EF633445	GCTAAAA---	----------	----------	-TAATTTTGA	CTAGCGGAGG	CTAAAA-GGA	GAGAG--ATG	GGTGCGAGAG	CG
F1.AR.02.ARE933.DQ189088	GCCAA-----	----------	----------	---ATTTTGA	CTAGCAGAGG	CTAGAA-GGA	GAGAG--ATG	GGTGCGAGAG	CG
F1.BE.93.VI850.AF077336	GCCGAAT---	----------	---------T	TTTTTTTTGA	CTAGCGGAGG	CTAGAA-GGA	GAGAG--ATG	GGTGCGAGAG	CG
F1.BR.02.02BR082.FJ771006	GCCAA-----	----------	----------	---ATTTTGA	CTAGCAGAGG	CTAGAA-GGA	GAGAG--ATG	GGTGCGAGAG	CG
F1.BR.02.02BR170.FJ771007	GCCAA-----	----------	----------	---ATTTTGA	CTAGCGGAGG	CTAGAA-GGA	GAGAG--ATG	GGTGCGAGAG	CG
F1.BR.06.06BR564.FJ771008	GCCAA-----	----------	----------	---ATTTTGA	CTAGCGGAGG	CTAGAA-GGA	GAGAG--ATG	GGTGCGAGAG	CG
F1.BR.06.06BR579.FJ771009	GCCAAA----	----------	----------	---TTTTTGA	CTAGCGGAGG	CTAGAA-GGA	GAGAG--ATG	GGCGCGAGAG	CG
F1.BR.07.07BR844.FJ771010	GCCTA-----	----------	----------	---ATTTTGA	CTAGCGGAGG	CTAGAA-GGA	GAGAG--ATG	GGTGCGAGAG	CG
F1.BR.89.BZ126.AY173957	GCCAA-----	----------	----------	---ATTTTGA	CTAGCGGAGG	CTAGAA-GGA	GAGAG--ATG	GGTGCGAGAG	CG
F1.BR.93.93BR020_1.AF005494	GCCAAAA---	----------	----------	-----TTTGA	CTAGCAGAGG	CTAGAA-GGA	GAGAG--ATG	GGTGCGAGAG	CG
F1.DE.x.MVP_30846.EU446022	GCCAA-----	----------	----------	---TTTTTGA	CTAGCGGAGG	CTAGAA-GGA	GAGAG--ATG	GGTGCGAGAG	CG
F1.ES.02.ES_X845_4.FJ670516	GCCAA-----	----------	----------	---TTTTTGA	CTAGCGGAGG	CTAGAA-GGA	GAGAG--ATG	GGTGCGAGAG	CG
F1.ES.11.DEMF110ES001.JX140671	GCCAAAA---	----------	----------	---ATTTTAA	CTAGCAGAGG	CTAGAA-GGA	GAGAG--ATG	GGTGCGAGAG	CG
F1.ES.x.P1146.DQ979023	GCCAA-----	----------	----------	---TTTTTGA	CTAGCGGAGG	CTAGAA-GGA	GAGAG--ATG	GGTGCGAGAG	CG
F1.ES.x.X1093_2.DQ979025	GCCRA-----	----------	----------	---TTTTTGA	CTAGCGGAGG	CTAGAA-GGA	GAGAG--ATG	GGTGCGAGAG	CG
F1.ES.x.X1670.DQ979024	GCCAA-----	----------	----------	---TTTTTGA	CTAGCGGAGG	CTAGAA-GGA	GAGAG--ATG	GGTGCGAGAG	CG
F1.FI.93.FIN9363.AF075703	GCCAA-----	----------	----------	---TTTTTGA	CTAGCGGAGG	CTAGAA-GGA	GAGAG--ATG	GGTGCGAGAG	CG
F1.RO.96.BCI_R07.AB485658	GCCAA-----	----------	----------	---TTTTTGA	CTAGCGGAGG	CTAGAA-GGA	GAG-G--ATG	GGTGCGAGAG	CG
F1.RU.08.D88_845.GQ290462	GCCA------	----------	----------	ATTTTTTTGA	CTAGCGGAGG	CTAGAA-GGA	GAGAG--ATG	GGTGCGAGAG	CG
F2.CM.10.DEMF210CM007.JX140673	GCCAAAA---	----------	----------	---TTTTTGA	CTAGCAGAGG	CTAGAA-GGA	GAGAG--ATG	GGTGCGAGAG	CG
G.BE.96.DRCBL.AF084936	GCCAAAA---	----------	----------	---TTTTTGA	CTAGCGGAGG	CTAGAA-GGA	GAGAG--ATG	GGTGCGAGAG	CG
G.CM.01.A1786.FJ389367	GCCAAAA---	----------	----------	---TTTTTGA	CTAGCGGAGG	CTAGAA-GGA	GAGAG--ATG	GGTGCGAGAG	CG
G.CM.04.178_15.FJ389363	GCCAAAA---	----------	----------	---TTTTTGA	CTAGCGGAGG	CTAGAA-GGA	GAGAG--ATG	GGTGCGAGAG	CG
G.CM.04.314_40.FJ389364	GCCCAAA---	----------	--------AA	ATTTTTTTGA	CTAGCGGAGG	CTAGAA-GGA	GAGAG--ATG	GGTGCGAGAG	CG
G.CM.04.515_28.FJ389365	GCCAW-----	----------	----------	---TTTTTGA	CTAGCGGAGG	CTAGAA-GGA	GAGAG--ATG	GGTGCGAGAG	CG
G.CM.04.944_5.FJ389366	GCCAAAA---	----------	----------	-ATTTTTTGA	CTAGCGGAGG	CTAGAA-GGA	GAGAG--ATG	GGTGCGAGAG	CG
G.CM.10.DEMG10CM008.JX140676	GCCAAAA---	----------	----------	----TTTTGA	CTAGCAGAGG	CTAGAA-GGA	GAGAG--ATG	GGTGCGAGAG	CG
G.CM.96.96CMABB55.AY772535	GCCAAAA---	----------	----------	--ATTTTTGA	CTAGCGGGGG	CTAGAA-GGA	GAGAG--ATG	GGTGCGAGAG	CG
G.CN.06.sh52.HM067749	GCCAAAA---	----------	----------	-AATTTTTGA	CTAGCGGAGG	CTAGAA-GGA	GAGAG--ATG	GGTGCGAGAG	CG
G.CU.99.Cu74.AY586547	GCCAAAC---	----------	----------	---TTTTTGA	CTAGCGGAGG	CTAGAA-GGA	GAGAG--ATG	GGTGCGAGAG	CG
G.CU.99.Cu85.AY586548	GCCAAAT---	----------	----------	---TTTTTGA	CTAGCGGAGG	CTAGAA-GGA	GAGAG--ATG	GGTGCGAGAG	CG
G.CU.99.Cu87.AY586549	GCCA------	----------	----------	---TTTTTGA	CTAGCGGAGG	CTAGAA-GGA	GAGAG--ATG	GGTGCGAGAG	CG
G.ES.05.P962.EU786670	GCCAAAC---	----------	----------	---TTTTTGA	CTAGCGGAGG	CTAGAA-GGA	GAGAG--ATG	GGTGCGAGAG	CG
G.ES.08.P1981_2.FJ670530	GCCAAAT---	----------	----------	---TTTTTGA	CTAGCGGAGG	CTAGAA-GGA	GAGAG--ATG	GGTGCGAGAG	CG
G.ES.09.X2634_2.GU362882	GCCATAA---	----------	----------	---ATTTTGA	CTAGCGGAGG	CTAGAA-GGA	GAGAG--ATG	GGTGCGAGAG	CG
G.ES.99.X138.AF450098	GCCATAA---	----------	----------	---ATTTTGA	CTAGCGGAGG	CTAGAA-GGA	GAGAG--ATG	GGTGCGAGAG	CG
G.GH.03.03GH175G.AB287004	GCCAAAT---	----------	----------	--CCTTTTGA	CTAGCGGAGG	CTAGAA-GGA	GAGAG--ATG	GGTGCGAGAG	CG
G.KE.93.HH8793_12_1.AF061641	GCCAAAC---	----------	----------	---TTTTTGA	CTAGCGGAGG	CTAGAA-GGA	GAGAG--ATG	GGTGCGAGAG	CS
G.NG.92.92NG083_JV10832.U88826	GCCA------	----------	----------	-TTTTTTGAC	TAGCGGAGGG	CTAGAA-GGA	GAGAG--GTG	GGTGCGAGAG	CG
G.PT.x.PT2695.AY612637	GCCATAA---	----------	----------	--AATTTTGA	CTAGCGGAGG	CTAGAA-GGA	GAGAG--ATG	GGTGCGAGAG	CG
G.PT.x.PT3037.FR846408	GCCATAA---	----------	----------	---ATTTTGA	CTAGCGGAGG	CTAGAA-GGA	GAGAG--ATG	GGTGCGAGAG	CG
G.PT.x.PT3306.FR846409	GCCA------	----------	----------	--TTTTTTGA	CTAGCGGAGG	CTAGAA-GGA	GAGAG--ATG	GGTGCGAGAG	CG
G.PT.x.PT988.FR846410	GCCATAA---	----------	----------	---ATTTTGA	CTAGCGGAGG	CTAGAA-GGA	GAGAG--GTG	GGTGCGAGAG	CG
G.SE.93.SE6165_G6165.AF061642	GCCAAAA---	----------	----------	---TTTTTGA	CTAGCGGAGG	CTAGAA-GGG	GAGAG--ATG	GGTGCGAGAG	CG
H.BE.93.VI991.AF190127	GCCGAAA---	----------	-------ATT	TTTATTTTGA	CTAGCGGAGG	CTAGAA-GGA	GAGAG--ATG	GGTGCGAGAG	CG
H.BE.93.VI997.AF190128	GCCGAAA---	----------	-------ATT	TTTATTTTGA	CTAGCGGAGG	CTAGAA-GGA	GAGAG--ATG	GGTGCGAGAG	CG
H.CF.90.056.AF005496	GCCATTT---	----------	----------	--TGTTTTGA	CTAGCGGAGG	CTAGAA-GGA	GAGAG--ATG	GGTGCGAGAG	CG
H.GB.00.00GBAC4001.FJ711703	GCCGAAA---	----------	-----AATTT	TTGTTTTTGA	CTAGCGGAGG	CTAGAA-GGA	GAGAG--ATG	GGTGCGAGAG	CG
J.CM.04.04CMU11421.GU237072	GCCATTT---	----------	----------	TGTTTTTTGA	CTAGCGGAGG	CTAGAA-GGA	GAGAG--ATG	GGTGCGAGAG	CG
J.SE.93.SE9280_7887.AF082394	GCCAAAA---	----------	----------	ATATTTTTGA	CTAGCGGAGG	CTAGAA-GGA	GAGAG--ATG	GGTGCGAGAG	CG
J.SE.94.SE9173_7022.AF082395	GCCAAAA---	----------	---------A	TATTTTTTGA	CTAGCGGAGG	CTAGAA-GGA	GAGAG--ATG	GGTGCGAGAG	CG
01_AE.CF.90.90CF11697.AF197340	GCCAAT----	----------	----------	---TTTCTGA	CTAGCGGAGG	CTAGAA-GGA	GAGAG--ATG	GGTGCGAGAG	CG
01_AE.CF.90.90CF4071.AF197341	GCCAAA----	----------	----------	---TTTTTGA	CTAGCGGAGG	CTAGAA-GGA	GAGAG--ATG	GGTGCGAGAG	CG
01_AE.CF.90.90CR402_CAR_E_4002	GCCAAAA---	----------	----------	---ATTTTGA	CTAGCGGAGG	CTAGAA-GGA	GAGAG--ATG	GGTGCGAGAG	CG
01_AE.CN.05.FJ051.DQ859178	GCCAA-----	----------	----------	---ATTTTGA	CTAGCGGAGG	CTAGAA-GGA	GAGAG--ATG	GGTGCGAGAG	CG
01_AE.CN.05.FJ053.DQ859179	GCCAA-----	----------	----------	---ATTTTGA	CTAGCGGAGG	CTAGAA-GGA	GAGAG--ATG	GGTGCGAGAG	CG
01_AE.CN.05.Fj052.EF036528	GCCAA-----	----------	----------	---ATTTTGA	CTAGCGGAGG	CTAGAA-GGA	GAGAG--ATG	GGTGCGAGAG	CG
01_AE.CN.05.Fj055.EF036527	GCCAA-----	----------	----------	---TTTTTGA	CTAGCGGAGG	CTAGAA-GGA	GAGAG--ATG	GGTGCGAGAG	CG
01_AE.CN.05.Fj056.EF036529	GCCAA-----	----------	----------	---TTTTTGA	CTAGCGGAGG	CTAGAA-GGA	GAG-G--ATG	GGTGCGAGAG	CG
01_AE.CN.05.Fj057.EF036530	GCCAA-----	----------	----------	---TTTTTGA	CTAGCGGAGG	CTAGAA-GGA	GAG-G--ATG	GGTGCGAGAG	CG
01_AE.CN.05.Fj065.EF036534	GCCAA-----	----------	----------	---TTTTTGA	CTAGCGGAGG	CTAGAA-GGA	GAGAG--ATG	GGTGCGAGAG	CG
01_AE.CN.05.Fj066.EF036535	GCCAA-----	----------	----------	---ATTTTGA	CTAGCGGAGG	CTAGAA-GGA	GAGAG--ATG	GGTGCGAGAG	CG
01_AE.CN.06.FJ054.DQ859180	GCCAA-----	----------	----------	---ATTTTGA	CTAGCGGAGG	CTAGAA-GGA	GAGAG--ATG	GGTGCGAGAG	CG
01_AE.CN.06.Fj062.EF036531	GCCAA-----	----------	----------	---TTTTTGA	CTAGCGGAGG	CTAGAA-GGA	GGGAG--ATG	GGTGCGAGAG	CG
01_AE.CN.06.Fj063.EF036532	GCCAA-----	----------	----------	---TTTTTGA	CTAGCGGAGG	CTAGAA-GGA	GAGAG--ATG	GGTGCGAGAG	CG
01_AE.CN.06.Fj064.EF036533	GCCAA-----	----------	----------	---TTTTTGA	CTAGCAGAAG	CTAGAA-GGA	GAGAG--ATG	GGTGCGAGAG	CG
01_AE.HK.04.HK001.DQ234790	GCCAA-----	----------	----------	---ATTTTGA	CTAGCGGAGG	CTAGAA-GGA	GAGAG--ATG	GGTGCGAGAG	CG
01_AE.JP.x.DR0492.AB253423	GCCAA-----	----------	----------	---ATTTTGA	CTAGCGGTGG	CTAGAA-GGA	GAGAAG-ATG	GGTGCGAGAG	CG
01_AE.JP.x.DR2594.AB253660	GCCAA-----	----------	----------	---TTTTTGA	CTAGCGGAGG	CTAGAA-GGA	GAGAA--ATG	GGTGCGAGAG	CG
01_AE.JP.x.DR6824.AB253426	GCCAA-----	----------	----------	---TTTTTGA	CTAGCGGAGG	CTAGAA-GGA	GAGAG--ATG	GGTGCGAGAG	CG
01_AE.JP.x.JRC77AE.AB565504	GCCAAAT---	----------	----------	---TTTTTGA	CTAGCGGAGG	CTAGAA-GGA	GAGAG--ATG	GGTGCGAGAG	CG
01_AE.TH.04.AA027a_wg4.JX44702	GCCAA-----	----------	----------	---ATTTTGA	CTAGCAGAGG	CTAGAA-GGA	GAGAG--ATG	GGTGCGAGAG	CG
01_AE.TH.04.AA075a_WG7.JX44754	GCCAA-----	----------	----------	---ACTTTGA	CTAGCGGAGG	CTAGAA-GGA	GAAAG--ATG	GGTGCGAGAG	CG
01_AE.TH.04.BKM.DQ314732	GCCAA-----	----------	----------	---GTTTTGA	CTAGCGGAGG	CTAGAA-GGA	GAGAG--ATG	GGTGCGAGAG	CG
01_AE.TH.05.AA004a_wg4a.JX4467	GCCAAAG---	----------	----------	---TTTTTGA	CTAGCGGAGG	CTAGAA-GGA	GAGAG--ATG	GGTGCGAGAG	CG
01_AE.TH.05.AA033a_wg6a.JX4470	GCCAA-----	----------	----------	--TTTTTTGA	CTAGCGGAGG	CTAGAA-GGA	GAGAG--ATG	GGTGCGAGAG	CG
01_AE.TH.05.AA049a_WG13.JX4472	GCCAA-----	----------	----------	---ATTTTGA	CTAGCGGAGG	CTAGAA-GGA	GAGAG--ATG	GGTGCGAGAG	CG
01_AE.TH.05.AA064a_WG2.JX44741	GCCAA-----	----------	----------	---ATTTTGA	CTAGCGGAGG	CTAGAA-GGA	GAGAG--ATG	GGTGCGAGAG	CG
01_AE.TH.05.AA079a_WG4.JX44759	GCCAA-----	----------	----------	---TTTTTGA	CTAGCGGAGG	CTAGAA-GGA	GAGAG--ATG	GGTGCGAGAG	CG
01_AE.TH.05.AA101a_WG1.JX44793	GCCAA-----	----------	----------	---ATTTTGA	CTAGCGGAGG	CTAGAA-GGA	GAGAAG-ATG	GGTGCGAGAG	CG

```
01_AE.TH.05.AA107a_wg4.JX44802   GCCA------ ---------- ---------- ---ATTTTGA CTAGCAGAGG CTAGAA-GGA GAGAG--ATG GGTGCGAGAG CG
01_AE.TH.06.AA002a_WG1.JX44666   GCCAA----- ---------- ---------- ---ATTTTGA CTAGCGGAGG CTAGAA-GGA GAGAG--ATG GGTGCGAGAG CG
01_AE.TH.06.AA017a_wg1.JX44689   GCCAAAA--- ---------- ---------- ---TTTTTGA CTAGCGGAGG CTAGAA-GGA GAGAG--ATG GGTGCGAGAG CG
01_AE.TH.06.AA034a_wg2.JX44708   GCCAA----- ---------- ---------- ---ATTTTGA CTAGCGGAGG CTAGAA-GGA GAGAG--ATG GGTGCGAGAG CG
01_AE.TH.06.AA038a_WG3.JX44713   GCCAA----- ---------- ---------- ---ATTTTGA CTAGCGGAGG CTAGAA-GGA GAGAG--ATG GGTGCGAGAG CG
01_AE.TH.06.AA055a_WG4.JX44731   GCCAA----- ---------- ---------- ---ATTTTGA CTAGCGGAGG CTAGAA-GGA GAGAG--ATG GGTGCGAGAG CG
01_AE.TH.06.AA056a_WG5.JX44731   GCCAA----- ---------- ---------- ---ATTTTGA CTAGCGGAGG CTAGAA-GGA GAGAG--ATG GGTGCGAGAG CG
01_AE.TH.06.AA059a_WG5.JX44735   GCCAAAA--- ---------- ---------- ---ATTTTGA CTAGCGGAGG CTAGAA-GGA GAGAG--ATG GGTGCGAGAG CG
01_AE.TH.06.AA063a_WG37.JX4474   GCCAA----- ---------- ---------- ---ATTTTGA CTAGCGGAGG CTAGAA-GGA GAGAG--ATG GGTGCGAGAG CG
01_AE.TH.06.AA068a_14.JX447465   GCCAA----- ---------- ---------- ---ATTTTGA CTAGCGGAGG CTAGAA-GGA GAGAG--ATG GGTGCGAGAG CG
01_AE.TH.06.AA082a_WG9.JX44764   GCCAA----- ---------- ---------- ---ATTTTGA CTAGCGGAGG CTAGAA-GGA GAGAG--ATG GGTGCGAGAG CG
01_AE.TH.06.AA085a_wg2.JX44768   GCCAA----- ---------- ---------- ---ATTTTGA CTAGCGGAGG CTAGAA-GGA GAGAG--ATG GGTGCGAGAG CG
01_AE.TH.06.AA088a_wg14.JX4477   GCCAA----- ---------- ---------- ---TTTTTGA CTAGCGGAGG CTAGAA-GGA GAGAG--ATG GGTGCGAGAG CG
01_AE.TH.06.AA099a_WG9.JX44789   GCCAA----- ---------- ---------- ---ATTTTGA CTAGCGGAGG CTAGAA-GGA GAGAG--ATG GGTGCGAGAG CG
01_AE.TH.07.AA015a_WG4.JX44687   GCCAA----- ---------- ---------- ---TTTTTGA CTAGCGGAGG CTAGAA-GGA GAGAG--ATG GGTGCGAGAG CG
01_AE.TH.07.AA019a_WG11.JX4469   GCCAA----- ---------- ---------- ---ATTTTGA CTAGCGGAGG CTAGAA-GGA GAGAG--ATG GGTGCGAGAG CG
01_AE.TH.07.AA028a_wg3.JX44702   GCCAA----- ---------- ---------- ---ATTTTGA CTAGCAGAGG CTAGAA-GGA GAGAG--ATG GGTGCGAGAG CG
01_AE.TH.07.AA050a_WG7.JX44728   GCCAAAAA-- ---------- ---------- --AATTTTGA CTAGCGGAGG CTAGAA-GGA GAGAG--ATG GGTGCGAGAG CG
01_AE.TH.08.AA007a_WG10.JX4467   GCCAA----- ---------- ---------- ---ATTTTGA CTAGCGGAGG CTAGAA-GGA GAGAG--ATG GGTGCGAGAG CG
01_AE.TH.08.AA037a_WG6.JX44712   GCCAA----- ---------- ---------- ---ATTTTGA CTAGCGGAGG CTACAA-GGA GAGAG--ATG GGTGCGAGAG CG
01_AE.TH.08.AA060a_WG1.JX44735   GCCAC----- ---------- ---------- ---ATTTTGA CTAGCAGAGG CTAGAA-GGA GAGAG--ATG GGTGCGAGAG CG
01_AE.TH.08.AA067a_WG12.JX4474   GCCA------ ---------- ---------- ---ATTTTGA CTAGCGGAGG CTAGAA-GGA GAGAG--ATG GGTGCGAGAG CG
01_AE.TH.08.AA108a_WG6.JX44802   GCCAA----- ---------- ---------- ---ATTTTGA CTAGCGGAGG CTAGAA-GGA GAGAG--ATG GGTGCGAGAG CG
01_AE.TH.09.AA090a_WG11.JX4477   GCCA------ ---------- ---------- ---ATTTTGA CTAGCAGAGG CTAGAA-GGA GAGAG--ATG GGTGCGAGAG CG
01_AE.TH.09.AA111a_WG11.JX4480   GCCAA----- ---------- ---------- ---ATTTTGA CTAGCGGAGG CTAGAA-GGA GAGAG--ATG GGTGCGAGAG CG
01_AE.TH.90.CM240.U54771         GCCAA----- ---------- ---------- ---ATTTTGA CTAGCGGAGG CTAGAA-GGA GAGAG--ATG GGTGCGAGAG CG
01_AE.TH.93.93TH253.U51189       GCCAA----- ---------- ---------- ---ATTTTGA CTAGCGGAGG CTAGAA-GGA GAGAG--ATG GGTGCGAGAG CG
01_AE.TH.93.93TH9021.AF164485    GCCAA----- ---------- ---------- ---ATTTTGA CTAGCGGAGG CTAGAA-GGA GAGAG--ATG GGTGCGAGAG CT
01_AE.TH.95.95TNIH022.AB032740   GCCAA----- ---------- ---------- ---ATTTTGA CTAGCAGAGG CTAGAA-GGA GAGAG--ATG GGTGCGAGAG CG
01_AE.TH.95.95TNIH047.AB032741   GCCAA----- ---------- ---------- ---ATTTTGA CTAGCGGAGG CTAGAA-GGA GAGAG--ATG GGTGCGAAAG CG
01_AE.TH.x.NP03.AB485654         GCCAA----- ---------- ---------- ---ATTTTGA CTAGCGGAGG CTAGAA-GGA GAGAG--ATG GGTGCGAGAG CG
01_AE.VN.97.97VNAG204.FJ185247   GCCAA----- ---------- ---------- ---ATTTTGA CTAGCGGAGG CTAGAA-GGA GAGAG--ATG GGTGCGAGAG CG
01_AE.VN.97.97VNAG206.FJ185248   GCCAA----- ---------- ---------- ---ATTTTGA CTAGCGGAGG CTAGAA-GGA GAGAG--ATG GGTGCGAGAG CG
01_AE.VN.97.97VNAG207.FJ185249   GCCAA----- ---------- ---------- ---ATTTTGA CTAGCGGAGG CTAGAA-GGA GAGAG--ATG GGTGCGAGAG CG
01_AE.VN.97.97VNAG210.FJ185251   GCCAA----- ---------- ---------- ---ATTTTGA CTAGCGGAGG CTAGAA-GGA GAGAG--ATG GGTGCGAGAG CG
01_AE.VN.97.97VNAG212.FJ185252   GCCAA----- ---------- ---------- ---ATTTTGA CTAGCGGAGG CTAGAA-GGA GAGAG--ATG GGTGCGAGAG CG
01_AE.VN.97.97VNAG214.FJ185253   GCCAA----- ---------- ---------- ---ATTTTGA CTAGCGGAGG CTAGAA-GGA GAGAG--ATG GGTGCGAGAG CG
01_AE.VN.97.97VNAG218.FJ185255   GCCAA----- ---------- ---------- ---ATTTTGA CTAGCGGAGG CTAGAA-GGA GAGAG--ATG GGTGCGAGAG CG
01_AE.VN.97.97VNHCM314.FJ18524   GCCAA----- ---------- ---------- ---ATTTTGA CTAGCAGAGG CTAGAA-GGA GAGAG--ATG GGTGCGAGAG CG
01_AE.VN.97.97VNHCM319.FJ18524   GCCAA----- ---------- ---------- ---ATTTTGA CTAGCGGAGG CTAGAA-GGA GAGGG--ATG GGTGCGAGAG CG
01_AE.VN.97.97VNHCM343.FJ18524   GCCAA----- ---------- ---------- ---ATTTTGA CTAGCGGAGG CTAGCA-GGA GAGAG--ATG GGTGCGAGAG CG
01_AE.VN.97.97VNHCM345.FJ18524   GCCAA----- ---------- ---------- ---TTTTTGA CTAGCGGAGG CTAGAA-GGA GAGAG--ATG GGTGCGAGAG CG
01_AE.VN.98.98VNND15.FJ185235    GCCAA----- ---------- ---------- ---ATTTTGA CTAGCGGAGG CTAGAA-GGA GAGAG--ATG GGTGCGAGAG CG
02_AG.CM.08.DE00208CM001.JX140   GCCAA----- ---------- ---------- ---ATTTTGA CTAGTGGAGG CTAGAA-GGA GAGAG--ATG GGTGCGAGAG CG
02_AG.CM.08.DE00208CM004.JX140   GCCAA----- ---------- ---------- ---ATTTTGA CTAGCGGAGG CTAGAA-GGA GAGAG--ATG GGTGCGAGAG CG
02_AG.CM.99.pBD6_15.AY271690     GCCAA----- ---------- ---------- ---TTTTTGA CTAGCGGAGG CTAGAA-GGA GAGAG--ATG GGTGCGAGAG CG
02_AG.ES.06.P1261.EU786671       GCCAA----- ---------- ---------- ---ATTTTGA CTAGCGGAGG CTAGAA-GGA GAGAG--ATG GGTGCGAGAG CG
02_AG.ES.06.P1423.EU884501       GCCAA----- ---------- ---------- ---TTTTTGA CTAGCGGAGG CTAGAA-GGA GAGAG--ATG GGTGCGAGAG CG
02_AG.FR.91.DJ263.AF063223       GCCAA----- ---------- ---------- ---ATTTTGA CTAGCGGAGG CTAGAA-GGA GAGAG--ATG GGTGCGAGAG CG
02_AG.GH.03.03GH181AG.AB286855   GCCAA----- ---------- ---------- ---TTTTTGA CTAGCGGAGG CTAGAA-GGA GAGAG--ATG GGTGCGAGAG CG
02_AG.GH.03.03GH182AG.AB286857   GCCA------ ---------- ---------- ---ATTTTGA CTAGCGGAGG CTAGAA-GGA GAGAG--ATG GGTGCGAGAG CG
02_AG.GH.03.03GH189AG.AB286862   GCCA------ ---------- ---------- ---ATTTTGA CTAGCGGAGG CTAGAA-GGA GAGAG--ATG GGTGCGAGAG CG
02_AG.GH.03.03GH197AG.AB286863   GCCAAAA--- ---------- ---------- ----TTTTGA CTAGCGGAGG CTAGAA-GGA GAGAG--ATG GGTGCGAGAG CG
02_AG.GH.03.GHNJ185.AB231895     GCCAATT--- ---------- ---------- ----TTTGAC TAGACGGAGG CTAGAA-GGA GAAGAG-ATG GGTGCGAGAG CG
02_AG.GH.03.GHNJ188.AB231896     GCCAA----- ---------- ---------- ---TTTTTGA CTAGCGGAGG CTAGAA-GGA GAGAG--ATG GGTGCGAGAG CG
02_AG.GH.03.GHNJ196.AB231898     GCCAA----- ---------- ---------- ---TTTTTGA CTAGCGGAGG CTAGAA-GGA GAGAG--ATG GGTGCGAGAG CG
02_AG.GH.97.97GH_AG1.AB049811    GCCAA----- ---------- ---------- ---ATTTTGA CTAGCGGAGG CTAGAA-GGA GAGAG--ATG GGTGCGAGAG CG
02_AG.GH.x.I_2496.AB485633       GCCAA----- ---------- ---------- ---TTTTTGA CTAGCGGAGG CTAGAA-GGA GAGAG--ATG GGTGCGAGAG CG
02_AG.GW.04.CC_0030.FJ694791     GCCAA----- ---------- ---------- ---WTTTTGA CTAGCGGAGG CTAGAA-GGA GAGAG--ATG GGTGCGAGAG CG
02_AG.GW.05.CC_0048.FJ694792     GCCA------ ---------- ---------- ---ATTTTGA CTAGCGGAGG CTAGAA-GGA GAGAG--ATG GGTGCGAGAG CG
02_AG.KR.07.07MHI10.JQ316136     GCCAA----- ---------- ---------- ---ATTTTGA CTAGCGGAGG CTAGAA-GGA GAGAG--ATG GGTGCGAGAG CG
02_AG.LR.x.POC44951.AB485636     GCCAA----- ---------- ---------- ---TTTTTGA CTAGCGGAGG CTAGAA-GGA GAGAG--ATG GGTGCGAGAG CG
02_AG.NG.x.IBNG.L39106           GCCAA----- ---------- ---------- ---ATTTTGA CTAGCGGAGG CTAGAA-GGA GAGAG--ATG GGTGCGAGAG CG
02_AG.SE.94.SE7812.AF107770      GCCAA----- ---------- ---------- ---TTTTTGA CTAGCGGAGG CTAGAA-GGA GAGAG--ATG GGTGCGAGAG CG
03_AB.BY.00.98BY10443.AF414006   GCCTAAAGA- ---------- ---------- ---TTTTTGA CTAACGGAGG CTAGAA-GGA GAGAG--ATG GGTGCGAGAG CG
03_AB.RU.98.RU98001_98RU001.AF   GCCTAAAGA- ---------- ---------- ---TTTTTGA CTAGCGGAGG CTAGAA-GGA GAGAG--ATG GGTGCGAGAG CG
04_cpx.CY.94.94CY032_3.AF04933   GCCAAAT--- ---------- ---------- ---TTTTTGA CTAGCGGAGG CTAGAA-GGA GAGAG--ATG GGTGCGAGAG CG
04_cpx.GR.00.DE00400GR002.JX14   GCCAAAW--- ---------- ---------- ---TTTTTGA CTAGCGGAGG CTAGAA-GGA GAGAG--ATG GGTGCGAGAG CG
04_cpx.GR.91.GR11_97PVCH.AF119   GCCAAAT--- ---------- ---------- ---TTTTTGA CTAGCGGAGG CTAGAA-GGA GAGAG--ATG GGTGCGAGAG CG
04_cpx.GR.97.GR84_97PVMY.AF119   GCCAAA---- ---------- ---------- ---TTTTTGA CTAGCGGAGG CTAGAA-GGA GAGAG--ATG GGTGCGAGAG CG
05_DF.BE.93.VI961.AF076998       GCTAAAA--- ---------- ---------- ---CTTGTGA CTAGCGGAGG CTAGAA-GGA GAGAG--ATG GGTGCGAGAG CG
05_DF.BE.x.VI1310.AF193253       GCTAAAA--- ---------- ---------- ---TTTTTGA CTAGCGGAGG CTAGAA-GGA GAGAG--ATG GGTGCGAGAG CG
05_DF.ES.99.X492.AY227107        GCTAAAA--- ---------- ---------- ---TTTTTGA CTAGCGGAGG CTAGAA-GGA GAGTG--ATG GGTGCGAGAG CG
06_cpx.AU.96.BFP90.AF064699      GCCA------ ---------- ---------- ---TTTTTGA CTAGCAGAGG CTAGAA-GGA GAGAG--ATG GGTGCGAGAG CG
06_cpx.CD.x.BCF_Dioum.AB485660   GCCAA----- ---------- ---------- ---ATTTTGA CTAGCAGAGG CTAGAA-GGA GAGAG--ATG GGTGCGAGAG CG
06_cpx.EE.01.EE0359.AY535659     GCCAAAA--- ---------- ---------- -GTTTGTTGA CTAGCAGAGG CTAGAA-GGA GAGAG--ATG GGTGCGAGAG CG
06_cpx.GH.03.03GH173_06.AB2868   GCCAA----- ---------- ---------- ---TTTTTGA CTAGCAGAGG CTAGAA-GGA GAGAG--ATG GGTGCGAGAG CG
06_cpx.ML.95.95ML127.AJ288982    GCCAAAA--- ---------- ---------- ----AATTTC CTAGCAGAGG CTAGAA-GGA GAGAG--ATG GGTGCGAGAG CG
06_cpx.ML.95.95ML84.AJ245481     GCCAAAA--- ---------- ---------- -ATTTTTTGA CTAGCGGAGG CTAGAA-GGA GAGAG--ATG GGTGCGAGAG CG
06_cpx.SN.97.97SE1078.AJ288981   GCCA------ ---------- --------AT TTTTGTTTGA CTAGCGGAGG CTAGAA-GGA GAGAG--ATG GGTGCGAGAG CG
07_BC.CN.05.XJDC6431_2.EF36837   GCCATTT--- ---------- ---------- --ATATTTGA CTAGCGGAGG CTAGAA-GGA GAGAG--ATG GGTGCGAGAG CG
07_BC.CN.05.XJDC6441.EF368370    GCCAAAT--- ---------- ---------- --ATATTTGA CTAGCGGAGG CTAGAA-GGA GAGAG--ATG GGTGCGAGAG CG
07_BC.CN.06.Sichuan_2006_SC006   GCCAATT--- ---------- ---------- --GTATTTGA CTAGCGGAGG CTAGAA-GGA GAGAG--ATG GGTGCGAGAG CG
07_BC.CN.06.Sichuan_2006_SC008   GCCAATT--- ---------- ---------- --ATATTTGA CTAGCGGAGG CTAGAA-GGA GAGAG--ATG GGTGCGAGAG CG
07_BC.CN.06.Xinjiang_2006_709.   GCCAATT--- ---------- ---------- --ATATTTGA CTAGCGGAGG CTAGAA-GGA GAGAG--ATG GGTGCGAGAG CG
07_BC.CN.07.CNGZD.JQ423923       GCCAATT--- ---------- ---------- --ATATTTGA CTAGCGGAGG CTAGAA-GGA GAGAG--ATG GGTGCGAGAG CG
07_BC.CN.98.98CN009.AF286230     GCCAATT--- ---------- ---------- --ATATTTGA CTAGCGGAGG CTAGAA-GGA GAGAG--ATG GGTGCGAGAG CG
08_BC.CN.00.p00CH_HH090_08_BC3   GCCAATT--- ---------- ---------- --TTATTTGA CTAGCGGAGG CTAGAA-GGA GAGAG--ATG GGTGCGAGAG CG
08_BC.CN.00.p00CH_WS035_08_BC5   GCCAATT--- ---------- ---------- --TTATTTGA CTAGCGGAGG CTAGAA-GGA GAGAG--ATG GGTGCGAGAG CG
08_BC.CN.01.p01CH_DL001_08_BC0   GCCAATT--- ---------- ---------- --TTATTTGA CTAGCGGAGG CTAGAA-GGA GAGAG--ATG GGTGCGAGAG CG
08_BC.CN.06.nx2.HM067748         GCCAATT--- ---------- ---------- --TTATTTGA CTAGCGGAGG CTAGAA-GGA GAGAG--ATG GGTGCGAGAG CG
08_BC.CN.98.98CN006.AF286229     GCCAATT--- ---------- ---------- --TTATTTGA CTAGCGGAGG CTAGAA-GGA GAGAG--ATG GGTGCGAGAG CG
09_cpx.CI.00.00IC_10092.AJ8665   GCCATAT--- ---------- ---------- ---TTTTTGA CTAGCGGAGG CTAGAA-GGA GAGAG--ATG GGTGCGAGAG CG
10_CD.TZ.96.96TZ_BF061.AF28954   GCTAAAA--- ---------- ---------- --TTTTTTGA CTAGCGGAGG CTAGAA-GGA GAGAG--ATG GGTGCGAGAG CG
10_CD.TZ.96.96TZ_BF071.AF28954   GCTAAAA--- ---------- ---------- ---TTTTTGA CTAGCGGAGG CTAGAA-GGA GAGAG--ATG GGTGCGAGAG CG
10_CD.TZ.96.96TZ_BF110.AF28955   GCTGAAA--- ---------- ---------- ---TTTTTGG CTAGCGGAGG CTAGAA-GGA GAGAG--ATG GGTGCGAGAG CG
11_cpx.CM.95.95CM_1816.AF49262   GCCAA----- ---------- ---------- ---TTTTTGA CTAGCAGAGG CTAGAA-GGA GAGAG--ATG GGTGCGAGAG CG
11_cpx.CM.96.96CM_4496.AF49262   GCCAAAA--- ---------- ---------- -ATTTTTTGA CTAGCGGAGG CTAGAA-GGA GAGAG--ATG GGTGCGAGAG CG
11_cpx.CM.97.MP818.AJ291718      CGCCC----- ---------- ---------- ---TTTTTGA TTACCGGAGG TTAATA-GGA GAGAG--ATG GGTGCGAGAG CG
11_cpx.FR.99.MP1298.AJ291719     GCCAAAA--- ---------- ---------- AATTTTTTGA CTAGCAGAGG CTAAAA-GGA GAGAG--ATG GGTGCCAGAG CG
11_cpx.FR.99.MP1307.AJ291720     GCCATAA--- ---------- ---------- --ATTTTTGA CTAGCGGAGG CTAGAA-GGA GAGAG--ATG GGTGCGAGAG CG
11_cpx.GR.x.GR17.AF179368        GCCAAAA--- ---------- ---------- -AATTTTTGA CTAGCGGAGG CTAGAA-GGA GAGAG--ATG GGTGCGAGAG CG
12_BF.AR.97.A32879.AF408629      GCCAAAA--- ---------- ---------- ---TTTTTGA CTAGCGGAGG CTAGAA-GGA GAGAG--ATG GGTGCGAGAG CG
12_BF.AR.97.A32989.AF408630      GCCAAAA--- ---------- ---------- ---TTTTTGA CTAGCGGAGG CTAGAA-GGA GAGAG--ATG GGTGCGAGAG CG
12_BF.AR.99.ARMA159.AF385936     GCCAAAA--- ---------- ---------- ---ATTTTGA CTAGCGGAGG CTAGAA-GGA GAGAG--ATG GGTGCGAGAG CG
```

```
12_BF.UY.99.URTR23.AF385934       GCCAACA--- ---------- ---------- -AATTTTTGA CTAGCGGAGG CTAGAA-GGA GAGAG--ATG GGTGCGAGAG CG
12_BF.UY.99.URTR35.AF385935       GCCGAAA--- ---------- ---------- -AATTTTTGA CTAGCGGAGG CTAGAA-GGA GAGAG--ATG GGTGCGAGAG CG
13_cpx.CM.02.02CM_A1394.DQ8453    GCCGAAA--- ---------- ---------- -TTTTCTTGA CTAGCGGAGG CTAGAA-GTA GAAAG--ATG GGTGCGAGAG CG
13_cpx.CM.04.04CM_173_9.DQ8453    GCCAAAA--- ---------- ---------- ---TTTTTGA CTAGCGGAGG CTAGAA-GGA GAGAG--ATG GGTGCGAGAG CG
13_cpx.CM.04.04CM_632_28.DQ845    GCCAAAT--- ---------- ---------- -TTCTTTTGA CTAGCGGAGG CTAGAA-GGA GAGAG--ATG GGTGCGAGAG CG
13_cpx.CM.96.96CM_1849.AF46097    GCCAAAA--- ---------- ---------- -ATTTTTTGA CTAGTGGAGG CTAGAA-GGA GAGAG--ATG GGTGCGAGAG CG
13_cpx.CM.96.96CM_4164.AF46097    GCCAAAA--- ---------- ---------- -TTTTCTTGA CTAGCGGAGG CTAGAA-GGA GAGAG--ATG GGTGCGAGAG CG
14_BG.ES.00.X605.AF450096         GCCATAA--- ---------- ---------- ---ATTTTGA CTAGCGGAGG CTAGAA-GGA GAGAG--ATG GGTGCGAGAG CG
14_BG.ES.00.X623.AF450097         GCCATAA--- ---------- ---------- ---ATTTTGA CTAGCGGAGG CTAGAA-GGA GAGAG--ATG GGTGCGAGAG CG
14_BG.ES.05.X1870.FJ670522        GCCATAA--- ---------- ---------- ---ATTTTGA CTAGCGGAGG CTAGAA-GGA GAGAG--ATG GGTGCGAGAG CG
14_BG.ES.05.X772_8.FJ670528       GCCATAA--- ---------- ---------- ---ATTTTGA CTAGCGGAGG CTAGAA-GGA GAGAG--ATG GGTGCGAGAG CG
15_01B.TH.99.99TH_MU2079.AF516    GCCAA----- ---------- ---------- ---TTTTTGA CTAGCGGAGG CTAGAA-GGA GAGAG--ATG GGTGCGAGAG CG
15_01B.TH.99.99TH_R2399.AF5305    GCCAA----- ---------- ---------- ---ATTTTGA CTAGCGGAGG CTAGAA-GGA GAGAG--ATG GGTGCGAGAG CG
16_A2D.KR.97.97KR004.AF286239     GCTGAAA--- ---------- ---------- -ATTTTTTGA CTAGCGGAGG CTAAAA-GGA GAGAG--ATG GGTGCGAGAG CG
18_cpx.CU.99.CU14.AY586541        GCC------- ---------- ---------- ---TTTTTGA CTAGCGGAGG CTAGAA-GGA GAGGG--ATG GGTGCGAGAG CG
18_cpx.CU.99.CU68.AY894993        GCCGAAA--- ---------- ---------- ---TTTTTGA CTAGCGGAGG CTAGAA-GGA GAGAG--ATG GGTGCGAGAG CG
18_cpx.CU.99.CU76.AY586540        GCCAAAA--- ---------- ---------- ---TTTTTGA CTAGCGGAGG CTAGAA-GGA GAGAG--ATG GGTGCGAGAG CG
19_cpx.CU.99.CU29.AY588971        GCCA------ ---------- ---------- --TTTTTTAA CTAGCGGAGG CTAGAA-AGA GAGAG--ATG GGTGCGAGAG CG
20_BG.CU.03.CB134.DQ020274        GCCAAAC--- ---------- ---------- ---TTTTTGA CTAGCGGAGG CTAGAA-GGA GAGAG--ATG GGTGCGAGAG CG
20_BG.CU.99.Cu103.AY586545        GCCAAAC--- ---------- ---------- ---TTTTTGA CTAGCGGAGG CTAGAA-GGA GAGAG--ATG GGTGCGAGAG CG
20_BG.ES.99.R77.AY586544          GCCAAAC--- ---------- ---------- ---TTTTTGA CTAGCGGAGG CTAGAA-GGA GAGAG--ATG GGTGCGAGAG CG
22_01A1.CM.02.02CAMLT72.EU7439    GCCAA----- ---------- ---------- ---ATTTTGA CTAGCGGAGG CTAGAA-GGA GAGAG--ATG GGTGCGAGAG CG
22_01A1.CM.06.LPH27MF.JN864049    GCCAA----- ---------- ---------- ---ATTTTGA CTAGCGGAGG CTAGAA-GGA GAGAG--ATG GGTGCGAGAG CG
22_01A1.CM.10.LB054.JN864059      ACTCCCA--- ---------- ---------- -TTTTTTTGA CTAGCGGAGG CTAGAA-GGA GAGAG--ATG GGTGCGAGAG CG
23_BG.CU.03.CB118.AY900571        GCCAAAM--- ---------- ---------- ---TTTTTGA CTAGCGGAGG CTAGAA-GGA GAGAG--ATG GGTGCGAGAG CG
23_BG.CU.03.CB347.AY900572        GCCAAAC--- ---------- ---------- ---TTTTTGA CTAGCGGAGG CTAGAA-GGA GAGAG--ATG GGTGCGAGAG CG
24_BG.CU.03.CB378.AY900574        GCCAAAC--- ---------- ---------- ---TTTTTGA CTAGCGGAGG CTAGAA-GGA GAGAG--ATG GGTGCGAGAG CG
24_BG.CU.03.CB471.AY900575        GCCAAAC--- ---------- ---------- ---TTTTTGA CTAGCGGAGG CTAGAA-GGA GAGAG--ATG GGTGCGAGAG CG
24_BG.ES.08.X2456_2.FJ670526      GCCAAAC--- ---------- ---------- ----TTTTGA CTAGCGGAGG CTAGAA-GGA GAGAG--ATG GGTGCGAGAG CG
25_cpx.CM.06.06CM_BA_040.EU693    GCCAAAA--- ---------- ---------- AATTTTTTGA CTAGCGGAGG CTAGAA-GGA GAGAG--ATG GGTGCGAGAG CG
25_cpx.SA.03.J11233.EU697906      GCCATAA--- ---------- ---------- ---TTTTTGA CTAGCGGAGG CTAGAA-GGA GAGAG--ATG GGTGCGAGAG CG
25_cpx.SA.03.J11451.EU697908      GCCATAA--- ---------- ---------- ----TTTTGA CTAGCGGAGG CTAGAA-GGA GAGAG--ATG GGTGCGAGAG CG
26_AU.CD.02.02CD_KS069.FM87778    GCCAAAA--- ---------- ---------- --AGTTTTGA CTAGCGGAGG CTAAAA-GGA AAGAG--ATG GGTGCGAGAG CG
26_AU.CD.02.02CD_LBTB084.FM877    GCCAAAA--- ---------- ---------- ATTTTTTTGA CTAGCGGAGG CTAGGA-GGA GAGAG--ATG GGTGCGAGAG CG
26_AU.CD.02.02CD_MBTB047.FM877    GCCAAAA--- ---------- ---------- TATTTTTTGA CTAGCGGAGG CTAGAA-GGA GAGAG--ATG GGTGCGAGAG CG
26_AU.CD.97.97CD_KTB119.FM8777    GCCAAAA--- ---------- ---------- --TTTTTTGA CTAGCGGAGG CTAGAA-GAA GAGAAG-ATG GGTGCGAGAG CG
27_cpx.FR.04.04CD_FR_KZS.AM851    GCCAAAA--- ---------- ---------- -TTCTTTTGA CTAGCGGAGG CTAGAA-GGA GAGAG--ATG GGTGCGAGAG CG
28_BF.BR.05.0679SV.JF804812       GCCA------ ---------- ---------- ---TTTTTGA CTAGCGGAGG CTAGAA-GTA GAGAG--ATG GGTGCGAGAG CG
28_BF.BR.99.BREPM12313.DQ08587    GCCAAAT--- ---------- ---------- ---TTTTTGA CTAGCGGAGG CTAGAA-GGA GAGAG--ATG GGTGCGAGAG CG
28_BF.BR.99.BREPM12609.DQ08587    GCCAA----- ---------- ---------- ---ATTTTGA CTAGCGGAGG CTAGAA-GGA GAGAG--ATG GGTGCGAGAG CG
28_BF.BR.99.BREPM12817.DQ08587    GCCAA----- ---------- ---------- ---TTTTTGA CTAGCGGAGG CTAGAA-GGA GAGAG--ATG GGTGCGAGAG CG
29_BF.BR.01.BREPM16704.DQ08587    GCCAAAA--- ---------- ---------- ---ATTTTGA CTAGCGGAGG CTAGAA-GGA GAGAG--ATG GGTGCGAGAG CG
29_BF.BR.05.0264RI.JF804807       GCCAAAA--- ---------- ---------- ---ATTTTGA CTAGCGGAGG CTAGAA-GGA GAGAG--ATG GGTGCGAGAG CG
29_BF.BR.99.BREPM11948.DQ08587    GCCAAAA--- ---------- ---------- --ATTTTTGA CTAGCGAAGG CTAAAA-GGA GAGAG--ATG GGCGCAAGAG CT
31_BC.BR.02.110PA.EF091932        GCCA------ ---------- ---------- TTTTATTTGA CTAGCGGAGG CTAGAA-GGA GAGAG--ATG GGTGCGAGAG CG
31_BC.BR.04.04BR142.AY727527      GCCATTT--- ---------- ---------- --TTATTTGA CTAGCGGAGG CTAGAA-GGA GAGAG--ATG GGTGCGAGAG CG
32_06A1.EE.01.EE0369.AY535660     GCCAAA---- ---------- ---------- ---TTTTTGA CTAGCAGAGG CTAGAA-GGA GAGAG--ATG GGTGCGAGAG CG
32_06A1.EE.02.EST2002_1169.DQ1    GCCAAA---- ---------- ---------- ---TTTTTGA CTAGCAGAGG CTAGAA-GGA GAGAG--ATG GGTGCGAGAG CG
33_01B.ID.07.JKT189_C.AB547463    GCCA------ ---------- ---------- ---ATTTTGA CTAGCGGAGG CTAGAA-GGA GAGAG--ATG GGTGCGAGAG CG
33_01B.MY.05.05MYKL007_1.DQ366    GCCAA----- ---------- ---------- ---ATTTTGA CTAGCGGAGG CTAGAA-GGA GAGAG--ATG GGTGCGAGAG CG
33_01B.MY.05.05MYKL015_2.DQ366    GCCAA----- ---------- ---------- ---TTTTTGA CTAGCGGAGG CTAGAA-GGA GAGAG--ATG GGTGCGAGAG CG
33_01B.MY.05.05MYKL031_1.DQ366    GCCAAA---- ---------- ---------- ---TTTTTGA CTAGCAGAGG CTAGAA-GGA GAGAG--ATG GGTGCGAGAG CG
33_01B.MY.05.05MYKL045_1.DQ366    GCCAA----- ---------- ---------- ---TTTTTGA CTAGCGGAGG CTAGAA-GGA GAGAG--ATG GGTGCGAGAG CG
38_BF1.UY.03.UY03_3389.FJ21378    GCCAA----- ---------- ---------- ---ATTTTGA CTAGCGGAGG CTAGAA-GGA GAGAG--ATG GGTGCGAGAG CG
38_BF1.UY.04.UY04_3987.FJ21378    GCCAAT---- ---------- ---------- ---TTTTTGA CTAGCGGAGG CTAGAA-GGA GAGAG--ATG GGTGCGAGAG CG
38_BF1.UY.04.UY04_4022.FJ21378    GCCAA----- ---------- ---------- ---TTTTTGA CTAGCAGAGG CTAAAA-GGA GAGAG--ATG GGTGCGAGAG CG
38_BF1.UY.05.UY05_4752.FJ21378    GCCAA----- ---------- ---------- ---TTTTTGA CTAGCGGAGG CTAGAA-GGA GAGAG--ATG GGTGCGAGAG CG
39_BF.BR.03.03BRRJ103.EU735534    GCCGAAA--- ---------- ---------- ---ATTTTGA CTAGCGGAGG CTAGAA-GGA GAGAG--ATG GGTGCGAGAG CG
39_BF.BR.03.03BRRJ327.EU735536    GCCAAAA--- ---------- ---------- ---ATTTTGA CTAGCGGAGG CTAGAA-GGA GAGAG--ATG GGTGCGAGAG CG
39_BF.BR.04.04BRRJ179.EU735535    GCCA------ ---------- ---------- ---ATTTTGA CTAGCGGAGG CTAGAA-GGA GAGAG--ATG GGTGCGAGAG CG
40_BF.BR.04.04BRRJ115.EU735538    GCCAATA--- ---------- ---------- ---TTTTTGA CTAGCGGAGG CTAGAA-GGA GAGAG--ATG GGTGCGAGAG CG
40_BF.BR.04.04BRSQ46.EU735540     GCCAAAA--- ---------- ---------A TAAATTTTGA CTAGCGGAGG CTAGAA-GGA GAGAG--ATG GGTGCGAGAG CG
40_BF.BR.05.05BRRJ055.EU735537    GCCAAAA--- ---------- ---------A TAAATTTTGA CTAGCGGAGG CTAGAA-GGA GAGAG--ATG GGTGCGAGAG CG
40_BF.BR.05.05BRRJ200.EU735539    GCCAAAA--- ---------- ---------A TAAATTTTGA CTAGCGGAGG CTAGAA-GGA GAGAG--ATG GGTGCGAGAG CG
42_BF.LU.03.luBF_01_03.EU17015    GCCAAAAT-- ---------- ---------- ----TTTTGA CTAGCGGAGG CTAGAA-GGA GAGAGG---- -GTGCGAGAG CG
42_BF.LU.03.luBF_02_03.EU17015    GCCAAAAT-- ---------- ---------- ----TTTTGA CTAGCGGAGG CTAGAA-GGA GAGAGG---- -GTGCGAGAG CG
42_BF.LU.03.luBF_04_03.EU17014    GCCAAAAA-- ---------- ---------- ----TTTTGA CTAGCGGAGG CTAGAA-GGA GAGAGG---- -GTGCGAGAG CG
42_BF.LU.03.luBF_05_03.EU17015    GCCAAAAT-- ---------- ---------- ----TTTTGA CTAGCGGAGG CTAGAA-GGA RAGAGG---- -GTGCGAGAG CG
42_BF.LU.03.luBF_06_03.EU17014    GCCAAAAA-- ---------- ---------- ----TTTTGA CTAGCGGAGG CTAGAA-GGA GAGAGG---- -GTGCGAGAG CG
42_BF.LU.03.luBF_07_03.EU17014    GCCAAAAW-- ---------- ---------- ----TTTTGA CTAGCGGAGG CTAGAA-GGA GAGAGG---- -GTGCGAGAG CG
42_BF.LU.03.luBF_08_03.EU17014    GCCAAAAA-- ---------- ---------- ----TTTTGA CTAGCGGAGG CTAGAA-GGA GAGAGG---- -GTGCGAGAG CG
42_BF.LU.03.luBF_09_03.EU17015    GCCAAAAA-- ---------- ---------- ----WTTTG- ---------- ---------- ---------- -GTGCGAGAG CG
42_BF.LU.04.luBF_11_04.EU17014    GCCAAAAA-- ---------- ---------- ----TTTTGA CTAGSGGAGG CTAGAA-GGA GAGAGG---- -GTGCGAGAG CG
42_BF.LU.04.luBF_12_04.EU17014    GCCAAAAA-- ---------- ---------- ----WTTTG- ---------- ---------- ---------- -GTGCGAGAG CG
42_BF.LU.05.luBF_13_05.EU17013    GCCAAAAA-- ---------- ---------- ----TTTTGA CTAGCGGAGG CTAGAA-GGA GAGAGG---- -GTGCGAGAG CG
42_BF.LU.05.luBF_14_05.EU17014    GCCAAAAA-- ---------- ---------- ----TTTTGA CTAGSGGAGG CTAGAA-GGA GAGAGG---- -GTGCGAGAG CG
42_BF.LU.05.luBF_16_05.EU17015    GCCAAAAA-- ---------- ---------- ----WTTTG- ---------- ---------- ---------- -GTGCGAGAG CG
42_BF.LU.05.luBF_17_05.EU17014    GCCAAAAT-- ---------- ---------- ----TTTTGA CTAGCGGAGG CTAGAA-GGA GAGAGG---- -GTGCGAGAG CG
42_BF.LU.06.luBF_18_06.EU17013    GCCAAAAA-- ---------- ---------- --ATTTTTGA CTAGCGGAGG CTAGAA-GGA GAGAG----- GGTGCGAGAG CG
42_BF.LU.06.luBF_19_06.EU17013    GCCAAAAT-- ---------- ---------- ----TCTTGA CTAGCGGAGG CTAGAA-GGA GAGAG----- -GTGCGAGAG CG
43_02G.SA.03.J11223.EU697904      GCCAAT---- ---------- ---------- ---TTTTTGA CTAGCGGAGG CTAGAA-GGA GAGAG--ATG GGTGCGAGAG CG
43_02G.SA.03.J11232.EU697905      GCCAAAA--- ---------- ---------- ---TTTTTGA CTAGCGGAGG CTAGAA-GGA GAGAG--ATG GGTGCGAGAG CG
43_02G.SA.03.J11243.EU697907      GCCA------ ---------- ---------- --TTTTTTGA CTAGCGGAGG CTAGAA-GGA GAGAG--ATG GGTGCGAGAG CG
43_02G.SA.03.J11456.EU697909      GCCAAT---- ---------- ---------- ---TTTTTGC CTAGCGGAGG CTAGAA-GGA GAGAG--ATG GGTGCGAGAG CG
44_BF.CL.00.CH80.FJ358521         GCCAAT---- ---------- ---------- ---TTTTTGA CTAGCAGAGG CTAGAA-GGA GAGAG--ATG GGTGCGAGAG CG
44_BF.CL.01.CH12.AY536235         TGCCAAA--- ---------- ---------- ----TTTTGA CTAGCAGAGG CTAGAA-GGA GAGAG--ATG GGTGCGAGAG CG
45_cpx.CD.97.97CD_MBFE185.FN39    GCCAAAG--- ---------- ---------- -AATTTTTGA CTAGCGGAGG CTAGAA-GGA GAGAG--ATG GGTGCGAGAG CG
45_cpx.CD.97.97CD_MBS30.FN3928    GCCAAGA--- ---------- ---------- ---ATTTTGA CTAGCAGAGG CTAGAA-GGA GAGAG--ATG GGTGCGAGAG CG
45_cpx.CM.97.97CM_MP814.FN3928    GCCA------ ---------- ---------- --TTTTTTGA CTAGCRGRAGG CTARAA-GGA RARAG-ATG GGGGCGAGAG CG
45_cpx.FR.04.04FR_AUK.EU448295    GCCA------ ---------- ---------- ---TTTTTGA CTAGCGGAGG CTAGAA-GGA GAGAG--ATG GGTGCGAGAG CG
45_cpx.GA.97.97GA_TB45.FN39287    GCCAAAG--- ---------- ---------- AATTTTTTGA CTAGCGGTGG CTAGAA-GGA GAGAG--ATG GGTGCGAGAG CG
46_BF.BR.01.01BR087.DQ358801      GCCAA----- ---------- ---------- ---TTTTTGA CTAGCGGAGG CTAGAA-GGA GAGAG--ATG GGTGCGAGAG CG
46_BF.BR.01.01BR125.DQ358802      GCCAA----- ---------- ---------- ---ATTTTGA CTAGCAGAGG CTAGAA-GGA GAGAG--ATG GGTGCGAGAG CG
46_BF.BR.07.07BR_FPS625.HM0264    GCCAA----- ---------- ---------- ---ATTTTGA CTAGCAGAGG CTAGAA-GGA GAGAG--ATG GGTGCGAGAG CG
46_BF.BR.07.07BR_FPS742.HM0264    GCCAA----- ---------- ---------- ---ATTTTGA CTAGCAGAGG CTAGAA-GGA GAGAG--ATG GGTGCGAGAG CG
46_BF.BR.07.07BR_FPS783.HM0264    GCCAAAT--- ---------- ---------- ----TTTTGA CTAGCAGAGG CTAGAA-GGA GAGAG--ATG GGTGCGAGAG CG
46_BF.BR.07.07BR_FPS810.HM0264    GCCATAT--- ---------- ---------- ----TTTTGA CTAGCGGAGG CTAGAA-GGA GAGAG--ATG GGTGCGAGAG CG
46_BF.BR.07.07BR_FPS812.HM0264    GCCA------ ---------- ---------- ---ATTTTGA CTAGCAGAGG CTAGAA-GGA GAGAG--ATG GGTGCGAGAG CG
47_BF.ES.08.P1942.GQ372987        GCCAAAA--- ---------- ---------- ---ATTTTGA CTAGCGGAGG CTAGAA-GGA GAGAG--ATG GGTGCGAGAG CG
47_BF.ES.08.X2457_2.FJ670529      GCCTAAA--- ---------- ---------- --ATTTTTGA CTAGCGGAGG CTAGAA-GGA GAGAG--ATG GGTGCGAGAG CG
49_cpx.GM.02.N18380.HQ385477      GCCGAAT--- ---------- ---------- -ATTTTTTGA CTAGCGGAGG CTAGAA-GGA GAGAG--ATG GGTGCGAGAG CG
49_cpx.GM.03.N26677.HQ385479      GCCGAAA--- ---------- ---------- -ATTTTTTGA CTAGCGGAGG CTAGAA-GGA GAGAG--ATG GGTGCGAGAG CG
49_cpx.GM.97.N28353.HQ385478      GCCGAAA--- ---------- ---------A AATTTTTTGA CTAGCGGAGG CTAGAA-GGA GAGAG--ATG GGTGCGAGAG CG
52_01B.MY.03.03MYKL018_1.DQ366    GCCAAT---- ---------- ---------- ---TTTTTGA CTAGCGGAGG CTAGAA-GGA GAGAG--ATG GGTGCGAGAG CG
53_01B.MY.04.04MYKL016_1.DQ366    GCCAA----- ---------- ---------- ---ATTTTGA CTAGCGGAGG CTAGAA-GGA GAGAG--ATG GGTGCGAGAG CG
```

53_01B.MY.10.10MYKJ067.JX39061 GCCAA----- ---------- ---------- ---ATCTTGA CTAGCGGAGG CTAGAA-GGA GAGAG--ATG GGTGCGAGAG CG
53_01B.MY.10.10MYKJ079.JX39061 GCCAA----- ---------- ---------- ---ATTTTGA CTAGCAGAGG CTAGAA-GGA GAGAG--ATG GGTGCGAGAG CG
53_01B.MY.11.11FIR164.JX390610 GCCAA----- ---------- ---------- ---ATTTTGA CTAGCGGAGG CTAGAA-GGA GAGAG--ATG GGTGCGAGAG CG
54_01B.MY.07.07MYKLD49.EU03191 GCCAA----- ---------- ---------- ---ATTTTGA CTAGCGGAGG CTAGAA-GGA GAGAG--ATG GGTGCGAGAG CG
54_01B.MY.08.08MYKL044.JX39097 GCCAA----- ---------- ---------- ---AATTTGA CTAGCGGAGG CTAGAA-GGA GAGAG--ATG GGTGCGAGAG CG
54_01B.MY.09.09MYSB023.JX39097 GCCAA----- ---------- ---------- ---TTTTTGA CTAGCGGAGG CTAGAA-GGA GAGAG--ATG GGTGCGAGAG CG
55_01B.CN.10.HNCS102056.JX5746 GCCAA----- ---------- ---------- ---ATTTTGA CTAGCGGAGG CTAGAA-GGA GAGAG--ATG GGTGCGAGAG CG
55_01B.CN.11.GDDG318.JX574662 GCCAA----- ---------- ---------- ---ATTTTGA CTAGCGGAGG CTAGAA-GGA GAGAG--ATG GGTGCGAGAG CG
01A1.MM.99.mCSW105.AB097872 GCCAA----- ---------- ---------- ---ATTTTGA CTAGCGGAGG CTAGAA-GGA GAGAG--ATG GGTGCGAGAG CG
01B.CN.08.08CYM047.JF340054 GCCAA----- ---------- ---------- ---ATTTTGA CTAGCGGAGG CTAGAA-GGA GAGAG--ATG GGTGCGAGAG CG
01B.JP.x.pHIV_1_Y271B01AE64.AB GCCAA----- ---------- ---------- ---TTTTTGA CTAGCGGAGG CTAGAA-GGA GAGAG--ATG GGTGCGAGAG CG
01B.MM.00.mIDU502.AB097865 GCCAAAA--- ---------- ---------- AATTTTTTGA CTAGCGGAGG CTAGAA-GGA GAGAG--ATG GGTGCGAGAG CG
01B.MM.99.mCSW104.AB097867 GCCAA----- ---------- ---------- ---ATTTTGA CTAGCGGAGG CTAGAA-GGA GAGAG--ATG GGTGCGAGAG CG
01B.MY.04.04MYKL019_1.DQ366665 GCCAA----- ---------- ---------- ---ATCTTGA CTAGCGGAGG CTAGAA-GGA GAGAG--ATG GGTGCGAGAG CG
01B.MY.05.05MYKL043_1.DQ366666 GCCAA----- ---------- ---------- ---ATTTTGA CTAGCGGAGG CTAGAA-GGA GAGAG--ATG GGTGCGAGAG CG
01B.MY.06.06MMYKLD46.EF495062 GCCAA----- ---------- ---------- ---ATTTTGA CTAGCGGAGG CTAGAA-GGA GAGAG--ATG GGTGCGAGAG CG
01B.MY.07.07MYKLD47.EU031913 GCCAA----- ---------- ---------- ---TTTTTGA CTAGCGGAGG CTAGAA-GGA GAGAG--ATG GGTGCGAGAG CG
01B.MY.07.07MYKLD48.EU031914 GCCAA----- ---------- ---------- ---ATTTTGA CTAGCGGAGG CTAGAA-GGA GAGAG--ATG GGTGCGAGAG CG
01B.TH.05.05TH140456.JN631793 GCCAAAA--- ---------- ---------- ----TTTTGA CTAGCGGAGG CTAGAAGGGA GAGAG--ATG GGTGCGAGAG CG
01B.TH.05.AA095a_WG21.JX447830 GCCAA----- ---------- ---------- ---TTTTTGA CTAGCAGAGG CTAGAA-GGA GAGAG--ATG GGTGCGAGAG CG
01B.TH.06.AA020a_wg2.JX446927 GCCAA----- ---------- ---------- ---ATTTTGA CTAGCGGAGG CTAGAA-GGA GAGAG--ATG GGTGCGAGAG CG
01B.TH.06.AA025a_WG13.JX447000 GCCAA----- ---------- ---------- ---TTTTTGA CTAGCGGGGG CTAGAA-GGA GAGAG--ATG GGTGCGAGAG CG
01B.TH.06.AA084a_WG10.JX447668 GCCAA----- ---------- ---------- ---TTTTTGA CTACCGGAGG CTAGAA-GGA GAGAA--ATG GGTGCGAGAG CG
01B.TH.91.CM237.AY167123 GCCAAAA--- ---------- ---------- -AAATTTTGA CTAGCGGAGG CTAGAA-GGA GAGAG--ATG GGTGCGAGAG CG
01BC.MM.00.mCSW503.AB097866 GCCAATT--- ---------- ---------- --TTATTTGA CTAGCGGAGG CTAGAA-GGA GAGAG--ATG GGTGCGAGAG CG
01BC.MM.99.mIDU107.AB097868 GCCAATT--- ---------- ---------- --TTACTTGA CTAGCGGAGG CTAGAA-GGA GAGAG--ATG GGTGCGAGAG CG
01F2G.CM.02.LT31.JN864056 GCCAA----- ---------- ---------- ---ATTTTGA CTAGCGGGGG CTAGAA-GGA GAGAG--ATG GGTGCGAGAG CG
0206.DZ.10.DEURF10DZ001.JX1406 GCCAA----- ---------- ---------- ---TTTTTGA CTAGCGGAGG CTAGAA-GGA GAGAG--ATG GGTGCGAGAG CG
0206.GH.03.03GH195AG_06.AB2868 GCCAAAA--- ---------- ---------- ----TTTTGA CTAGCGGAGG CTAGAA-GGA GAGAG--ATG GGTGCGAGAG CG
0206.NE.00.NE36.AJ508597 GCCAA----- ---------- ---------- ---TTTTTGA CTAGCGGAGG CTAGAA-GGA GAGAG--ATG GGTGCGAGAG CG
0206.NE.00.NE95.AJ508596 GCCA------ ---------- ---------- --TTTTTTGA CTAGCAGAGG CTAGAA-GGA GAGAG--ATG GGTGCGAGAG CG
0206.NE.97.NE03.AJ508595 GCCAT----- ---------- ---------- ---ATTTTGA CTAGCAGAGG CTAGAA-GGA GAGAG--ATG GGTGCGAGAG CG
0209.CI.01.01IC_17395.AJ866554 GCCAA----- ---------- ---------- ---ATTTTGA CTAGCGGAGG CTAGAA-GGA GAGAG--ATG GGTGCGAGAG CG
0209.CI.01.01IC_PCI118.AJ86655 GCCAA----- ---------- ---------- ---TTTTTGA CTAGCGGAGG CTAGAA-GGA GAGAG--ATG GGTGCGAGAG CG
0209.CI.01.01IC_PCI127.AJ86655 GCCA------ ---------- ---------- TATTTTTTGA CTAGCGGAGG CTAGAA-GGA GAGAG--ATG GGTGCGAGAG CG
0209.CI.97.97IC_PCI3.AJ866555 GCCATAT--- ---------- ---------- ---TTTTTGA CTAGCGGAGG CTAGAA-GGA GAGAG--ATG GGTGCGAGAG CG
0222.CM.02.02CAMLT04.EU743964 GCCAA----- ---------- ---------- ---TTTTTGA CTAGCGGAGG CTAGAA-GGA GAGAG--ATG GGTGCGAGAG CG
0222.CM.08.BDSH129.JN864052 GCCAA----- ---------- ---------- ---TTTTTGA CTAGCGGAGG CTAGAA-GGA GAGAG--ATG GGTGCGAGAG CG
0222.CM.10.LB045.JN864053 GCCA------ ---------- ---------- ---ATTTTGA CTAGCGGAGG CTAGAA-GGA GAGAG--ATG GGTGCGAGAG CG
02A1.ES.05.X230_10.FJ670515 GCCAA----- ---------- ---------- ---ATTTTGA CTAGCGGAGG CTAGAA-GGA GAGAG--ATG GGTGCGAGAG CG
02A1.ES.07.ES_P1751.GQ372986 GCCG------ ---------- ---------- ---ATTTTGA CTAGCGGAGG CTAGAA-GGA GAGAG--ATG GGTGCGAGAG CG
02A1.GH.03.GHNJ176.AB231894 GCCA------ ---------- ---------- ---ATTTTGA CTAGCGGAGG CTAGAA-GGA GAGAG--ATG GGTGCGAGAG CG
02A1.GH.97.97GH_AG2.AB052867 GCCAA----- ---------- ---------- ---TTTTTGA CTAGCGGAGG CTAGAA-GGA GAGAG--ATG GGTGCGAGAG CG
02A1.RU.10.10RU6637.JN230353 GCCAA----- ---------- ---------- ---TTTTTGA CTAGCGGAGG CTAGAA-GGA GAGAG--ATG GGTGCGAGAG CG
02B.ES.99.99SP_11339.DQ926899 GCCA------ ---------- ---------- ---ATTTTGA CTAGCGGAGG CTAGAA-GGA GAGAG--ATG GGTGCGAGAG CG
02B.FR.02.URF4.JN882652 GCCA------ ---------- ---------- ---TTTTTGA CTAGCGGAGG CTAGAA-GGA GAGAG--ATG GGTGCGAGAG CG
02B.FR.09.URF2.JN882654 GCCAA----- ---------- ---------- ---ATTTTGA CTAGCGGAGG CTAGAA-RGA GAGAG--ATG GGTGCGAGGG CG
02BD.FR.08.URF1.JN882653 GCCAA----- ---------- ---------- ---TTTTTGA CTAGCGGAGG CTAGAA-GGA GAGAG--ATG GGTGCGAGAG CG
02BG.FR.10.URF5.JN882655 GCCAA----- ---------- ---------- ---CTTTTGA CTAGCGGAGG CTAGAA-GGA GAGAG--ATG GGTGCGAGAG CG
02C.BE.93.VI1035.AJ276595 GCCCA----- ---------- ---------- ---ACTTTGA CTAGCGGAGG CTAGAA-GGA GAGAG--ATG GGTGCGAGAG CG
02D.GH.03.GHNJ193.AB231897 GCCA------ ---------- ---------- ---ATTTTGA CTAGCGGAGG CTAGAA-GGA GAGAG--ATG GGTGCGAGAG CG
02D.GH.03.p03GH194AG09.AB48004 GCCAA----- ---------- ---------- ---ATTTTGA CTAGCGGAGG CTAGAA-GGA GAGAG--ATG GGTGCGAGAG CG
02F2G.CM.02.LT66.JN864057 GCCAAAA--- ---------- ---------- ---ATTTTGA CTAGCGGAGG CTAGAA-GGA GAGAG--ATG GGTGCGAGAG CG
02GK.CI.01.01IC_PCI123.AJ86655 GCCAT----- ---------- ---------- ---ATTTTGA CTAGCGGAGG CTAGAA-GGA GAGAG--ATG GGTGCGAGAG CG
06A1.BJ.x.B76.AJ293865 GCCAAAA--- ---------- ---------- -ATTTTTTGA CTAGCGGAGG CTAGAA-GGA GAGAG--ATG GGTGCGAGAG CG
1819.CU.99.CU64.AY894995 GCCGAAA--- ---------- ---------- ----TTTTGA CTAGCGGAGG CTAGAA-GGA GAGAG--ATG GGTGCGAGAG CG
26C.CD.02.02CD_LBTB032.FM87777 GCCAAAT--- ---------- ---------- ---TTTTTGA CTAGCGGAGG CTAGAA-GGA GAGAG--ATG GGTGCGAGAG CG
26C.CD.97.97CD_KFE267.FM877778 GCAAAAA--- ---------- ---------- -ATTTTTTGA CTAGCGGAGG CTAGAA-GGA GAGAG--ATG GGTGCGAGAG CG
26C.CD.97.MBFE250.FM877783 GCCAAAA--- ---------- ---------- ---TTTTTGA CTAGCGGAGG CTAGAA-GGA GAGAG--ATG GGTGCGAGAG TG
A1B.EE.02.EST2002_394.DQ167216 GCCTAAA--- ---------- ---------- -GATCTTTGA CTAGCGGAGG CTAGAA-GGA GAGAG--ATG GGTGCGAGAG CG
A1BD.GB.03.34567.JN417238 CCCCCT---- ---------- ---------- ---TTTTTGA CTACCGGAGG CTACAA-GGA GAGAG--ATG GGTGCGACAG CG
A1C.CA.04.04CA7750.EU220698 GCCT------ ---------- ---------- --TTTTTGAC TAGCGGAGGC T-AGAA-GGA GAGAG-ATTG GGTGCGAGAG CG
A1C.IN.01.1579A.DQ083238 GCCAA----- ---------- ---------- ---TTTTTGA CTAGCGGAGG CTAGAA-GGA GAGAG--ATG GGTGCGAGAG CG
A1C.IN.95.95IN21301.AF067156 GCCAAAT--- ---------- ---------- -TTTATTTGA CTAGCAGAGG CTAGAA-GGA GAGAG--ATG GGTGCGAGAG CG
A1C.RW.92.92RW009_06.U88823 GCCAAAT--- ---------- ---------- -TTTATTTGA CTAGCGGAGG CTAGAA-GGA GAGAG--ATG GGTGCGAGAG CG
A1CD.KE.02.ML2000.EU110093 GCCAA----- ---------- ---------- ---TTTTTGA CTAGCGGAGG CTAGAA-GGA GAGAG--ATG GGTGCGAGAG CG
A1CD.SE.95.SE8603.AF075702 GCAAAAA--- ---------- ---------- -ATTTTTTGA CTAGCGGAGG CTAGAA-GGA GAGAG--ATG GGTGCGAGAG CG
A1CDGKU.ZA.99.CM4_99ZACM4.AF41 GCTGAAA--- ---------- ---------- ----TTTTGA CTAGCGGAGG CTAGAA-GGA GAGAG--ATG GGTGCGAGAG CG
A1CG.KE.02.ML1979PCR.EU110096 GCCA------ ---------- ---------- --TTTTTTGA CTAGCGGAGG CTAGAA-GGA GAGAG--ATG GGTGCGAGAG CG
A1D.DK.96.FSA.DQ912822 GCTAAAA--- ---------- ---------- ---TTTTTGA CTAGCGGAGG CTAGAA-GGA GAGAG--ATG GGTGCGAGAG CG
A1D.DK.98.MA.DQ912823 GCTAAGA--- ---------- ---------- ---TTTTTGA CTAGCGGAGG CTAGAA-GGA GAGAG--ATG GGTGCGAGAG CG
A1D.GB.00.8179.JN417236 GCTAAAA--- ---------- ---------- ---TTTTTGA CTAGCGGAGG CTAGAA-GGA GAGAG--ATG GGTGCGAGAG CG
A1D.GB.03.33365.JN417239 GCTGAAA--- ---------- ---------- ---TTTTTGA CTAGCGGAGG CTAGAA-GGA GAGAG--ATG GGTGCGAGAG CG
A1D.GB.10.11762.JN417241 GCTGAAA--- ---------- ---------- ---TTTTTGA CTAGCGGAGG CTAGAA-GGA GAGAG--ATG GGTGCGAGAG CG
A1D.GB.10.12792.JN417240 GCTGAAA--- ---------- ---------- ---TTTTTGA CTAGCGGAGG CTAGAA-GGA GAGAG--ATG GGTGCGAGAG CG
A1D.KE.02.ML1974.EU110090 GCTAAAA--- ---------- ---------- --CTTTTTGA CTAGCGGAGG CTAGAA-GGA GAGAG--ATG GGTGCGAGAG CG
A1D.SE.93.SE6954.AF075701 GCTAAAA--- ---------- ---------- --ATTTTTGA CTAGCGGAGG CTAGAA-GGA GAGAG--ATG GGTGCGAGAG CG
A1D.TZ.96.TZBFL0011.AF442569 GCTGAAA--- ---------- ---------- ---TTTTTGA CTAGCGGAGG CTAGGA-GGA GAGAG--ATG GGTGCGAGAG CG
A1D.TZ.96.TZBFL0088.AF442570 GCTA------ ---------- ---------- ---TTTTTGA CTAGCGGAGG CTAGAA-GGA GAGAG--ATG GGTGCGAGAG CG
A1D.TZ.97.TZBFL0086.AF442566 GCTTAAT--- ---------- ---------- ---TTTTTGA CTAGCGGAGG CTAGAA-GGA GAGAG--ATG GGTGCGAGAG CG
A1D.UG.07.p191947.JX236674 GCCGAAC--- ---------- ---------- ---TTTTTGA CTAGCGGAGG CTAGAA-GGA GAGAG--ATG GGTGCGAGAG CG
A1D.UG.08.p191982.JX236675 GCTGAAT--- ---------- ---------- ----TTTTGA CTAGCGGAGG CTAGAA-GGA GAGAG--ATG GGTGCGAGAG CG
A1D.UG.90.UG266.AY352657 GCCTAAA--- ---------- ---------- --ATTTTTGA CTAGCGGAGG CTGGAA-GGA GAGAG--ATG GGTGCGAGAG CG
A1D.UG.92.UG035.AY352656 GCCAAGA--- ---------- ---------- -ATTTTTTGA CTAGCAGAGG CTAGAA-GGA GAGAG--ATG GGTGCGAGAG CG
A1DHK.NO.97.97NOGIL3.AJ237565 GCCGAAT--- ---------- ---------- ---TTTTTGA CTAGCGGAGG CTAGAA-GGA GAGAG--ATG GGTGCGAGAG CG
A1DK.CD.85.MAL_MALCG.X04415 GCCAA----- ---------- ---------- ---TTTTTGA CTAGCGGAGG CTAGAA-GGA GAGAG--ATG GGTGCGAGAG CG
A1G.BE.94.VI1197.AJ276596 GCCAA----- ---------- ---------- ---TTTTTGA CTAGCGGAGG CTAGAA-GGA GAGAG--ATG GGTGCGAGAG CG
A1G.NG.92.92NG003.U88825 GCCA------ ---------- ---------- ATTTTTTTGA CTAGCGGAGG CTAGAA-GGA GAGAG--ATG GGTGCGAGAG CG
A1G.RU.11.11RU6900.JX500697 GCCAA----- ---------- ---------- ---ATTTTGA CTAGCGGAGG CTAGAA-GGA GAGAG--ATG GGTGCGAGAG CG
A1G.RU.11.11RU6939.JX500706 GCCAA----- ---------- ---------- ---TTTTTGA CTAGCGGAGG CTAGAA-GGA GAGAG--ATG GGTGCGAGAG CG
A1GHU.GA.x.VI354.AF076474 GCCAA----- ---------- ---------- ---TTTTTGA CTAGCGGAGG CTAGAA-GGA GAGAG--ATG GGTGCGAGAG CG
A1GJ.BW.98.BW2117.AF192135 GCCGAAA--- ---------- --------AA TTTGTTTTGA CTAGCGGAGG CTAGAA-GGA GAGAG--ATG GGTGCGAGAG CG
A2C.ZA.98.DU178_98ZADU178.AF41 GCCAATT--- ---------- ---------- --TTATTTGA CTAGCGGAGG CTAGAA-GGA GAGAG--ATG GGTGCGAGAG CG
A2C.ZM.90.ZAM184.U86780 GCCTAAA--- ---------- ---------- -ATTTTTTGA CTAGCGGAGG CTAGAA-GGA GAGAG--ATG GGTGCGAGAG CG
A2CD.KE.01.ML1956.EU110089 GCTGAAA--- ---------- ---------- ---TTTTTGA CTAGCGGAGG CTAGAA-GGA GAGAG--ATG GGTGCGAGAG CG
AC.KE.97.ML672.AY322191 GCCAA----- ---------- ---------- ---TTTTTGA CTAGTGGAGG CTAGAA-GGA GAAAG--ATG GGTGCGAGAG CG
AC.ZA.04.04ZAPS204B1.DQ093606 GCCG------ ---------- --------AT TTTTATTTGA CTAGCGGAGG CTAGAA-GGA GAGAG--ATG GGTGCGAGAG CG
AGKU.CD.x.BCF_Kita.AB485665 GCCAAAT--- ---------- ---------- --ATTTTTGA CTAGCGGAGG CTAGAA-GGA GAGAG--ATG GGTGCGAGAG CG
BC.AR.01.ARE195FL.AY968312 GCCA------ ---------- ---------- TTTTATTTGA CTAGCGGAGG CTAGAA-GGA GAGAG--ATG GGTGCGAGAG CG
BC.BR.01.01_BR_RGS45.GQ365651 GCCATTT--- ---------- ---------- --TTATTTGA CTAGCGGAGG CTAGAA-GGA GAGAG--ATG GGTGCGAGAG CG
BC.BR.01.01_BR_RGS69.GQ365652 GCCATTT--- ---------- ---------- --TTATTTGA CTAGCGAAGG CTAGAA-GGA GAGAG--ATG GGTGCGAGTG CG
BC.BR.02.02_BR_HF31.GQ365649 GCCATTT--- ---------- ---------- --TTATTTGA CTAGCGGAGG CTAGAA-GGA GAGAG--ATG GGTGCGAGAG CG
BC.BR.05.05_BR_NSP24.GQ365650 GCCA------ ---------- --------TT TTTTATTTGA CTAGCGGAGG CTAGAA-GGA GAGAG--ATG GGTGCGAGAG CG
BC.BR.06.06BR1114.JN692478 GCCA------ ---------- --------TT TTTTATTTGA CTAGCGGAGG CTAGAA-GGA GAGAG--ATG GGTGCGAGTG CG
BC.BR.92.92BR023.HM100716 GCCATTT--- ---------- ---------- --TTATTTGA CTAGCGGAGG CTAGAA-GGA GAGAG--ATG GGTGCGAGAG CG

```
BC.CN.07.309.HM776938            GCCAATT--- ---------- ---------- --TTATTTGA CTAGCGGAGG CTAGAA-GGA GAGAG--ATG GGTGCGAGAG CG
BC.CN.07.341.HM776939            GCCAATT--- ---------- ---------- -TTTACTTGA CTAGCGGAGG CTAGAA-GGA GAGAG--ATG GGTGCGAGAG CG
BC.IN.02.INDNARI_0218440.EU000   GCCAATT--- ---------- ---------- --TTATTTGA CTAGCGGGGG CTAGAA-GGA GAGAG--ATG GGTGCGAGAG CG
BC.IN.02.NARI7_3.EU000511        GCCAATT--- ---------- ---------- --TTATTTGA CTAGCGGAGG CTAGAA-GGA GAGAG--ATG GGTGCGAGAG CG
BC.IN.02.NARI9_3.EU000508        GCCA------ ---------- ---------- ATTTTTTTGA CTAGCGGGGG CTAGAA-GGA GAGAG--ATG GGTGCGAGAG CG
BC.IN.99.NARI10_2.EU000516       GCCAATT--- ---------- ---------- --TTATTTGA CTAGCGGAGG CTAGAA-GGA GAGAG--ATG GGTGCGAGAG CG
BC.MM.99.mIDU103.AB097873        GCCAAAA--- ---------- ---------- --ATTTTTGA CTAGCGGAGG CTAGAA-GGA GAGAG--ATG GGTACGAGAG CG
BCU.FR.06.06FR_CRN.EU448296      GCCATTT--- ---------- ---------- --TTATTTGA CTAGCGGAGG CTAGAA-GGA GAGAG--ATG GGTGCGAGAG CG
BF.BR.03.BREPM1026.EF637055      GCCATAA--- ---------- ---------- ----TTTTGA CTAGCGGAGG CTAGAA-GGA GAGAG--ATG GGTGCGAGAG CG
BF.BR.03.BREPM1029.EF637052      GCCA------ ---------- ---------- ---ATTTTGA CTAGCGGAGG CTAAAA-GGA GAAAG--ATG GGTGCGAGAG CG
BF1.BR.00.BREPM13853.DQ085875    GCTGAAA--- ---------- ---------- ---ATTTTGA CTAGCGGAGG CTAGAA-GGA GAGAG--ATG GGTGCGAGAG CG
BF1.BR.01.01BR042.DQ358799       GCCAA----- ---------- ---------- ---ATTTTGA CTAGCAGAGG CTAGAA-GGA GAGAG--ATG GGTGCGAGAG CG
BF1.BR.01.01BR047.DQ358800       GCCAAAA--- ---------- ---------- ---ATTTTGA CTAGCGGAGG CTATAA-GGA GAGAG--ATG GGTGCGAGAG CG
BF1.BR.01.01BR226.DQ358803       GCCAAAA--- ---------- ---------- ---TTTTTGA CTAGCGGAGG CTAGAA-GGA GAGAG--ATG GGTGCGAGAG CG
BF1.BR.01.01BR323.DQ358804       GCCAA----- ---------- ---------- ---TTTTTGA CTAGCGGAGG CTAGAA-GGA GAGAG--ATG GGTGCGAGAG CG
BF1.BR.02.02BR005.DQ358806       GCCAAAT--- ---------- ---------- ---TTTTTGA CTAGCGGAGG CTAGAA-GGA GAGAG--ATG GGTGCGAGAG CG
BF1.BR.02.02BR006.DQ358807       GCCAAAA--- ---------- ---------- ---TTTTTGA CTAGCGGAGG CTAGAA-GGA GAGAG--ATG GGTGCGAGAG CG
BF1.BR.02.02BR033.DQ358811       GCCAAAA--- ---------- ---------- ---TTTTTGA CTAGCGGAGG CTAGAA-GGA GAGAG--ATG GGTGCGAGAG CG
BF1.BR.02.02BR034.DQ358812       GCCGAAT--- ---------- ---------- ---TTTTTGA CTAGCGGAGG CTAGAA-GGA GAGAG--ATG GGTGCGAGAG CG
BF1.BR.02.02BR2028.JN692437      GCCAAAA--- ---------- ---------- ---ATTTTGA CTAGCGGAGG CTAGAA-GGA GAGAG--ATG GGTGCGAGAG CG
BF1.BR.03.03BR2018.JN692449      GCCAAAA--- ---------- ---------- ---ATTTTGA CTAGCGGAGG CTAGAA-GGA GAGAG--ATG GGTGCGAGAG CG
BF1.BR.03.03BR2019.JN692448      GCCAAAA--- ---------- ---------- ---TTTTTGA CTAGCGGAGG CTAGAA-GGA GAGAG--ATG GGTGCGAGAG CG
BF1.BR.04.04BR1067.JN692456      GCCAAAA--- ---------- ---------- ---TTTTTGA CTAGCGGAGG CTAGAA-GGA GAGAG--ATG GGTGCGAGAG CG
BF1.BR.05.0008SP.JF804805        GCCAAAA--- ---------- ---------- -ACTTTTTGA CTAGCGGAGG CTAGAA-GGA GAGAG--ATG GGTGCGAGAG CG
BF1.BR.05.0632SV.JF804810        GCCAAAA--- ---------- ---------- ---TTTTTGA CTAGCGGAGG CTAGAA-GGA GAGCG--ATG GGTGCGAGAG CG
BF1.BR.06.06BR_FPS561.HM026455   GCCAATT--- ---------- ---------- ----TTTTGA CTAGCGGAGG CTAGAA-GGA GAGAG--ATG GGTGCGAGAG CG
BF1.BR.99.BREPM107.AY771588      GCCAAAA--- ---------- ---------- ---TTTTTGA CTAGCGGAGG CTAGAA-GGA GAGAG--ATG GGTGCGAGAG CG
BF1.BR.99.BREPM108.AY771589      GCCGAAA--- ---------- --------AT AAAATTTTGA CTAGCGGAGG CTAGAA-GGA GAGAG--ATG GGTGCGAGAG CG
BF1.BR.99.BREPM11931.DQ085869    GCCAAAA--- ---------- ---------- ----TTTTGA CTAGCGGAGG CTAGAA-GGA GAGAG--ATG GGTGCGAGAG CG
BF1.ES.08.ES_X2524_2.GQ372989    RCCAAAA--- ---------- ---------- -AAATTTTGA CTAGCGGAGG CTAGGA-GGA GAGAG--ATG GGTGCGAGAG CG
BF1.ES.08.X2432_2.FJ853621       GCCTAAT--- ---------- ---------- -TTATTTTGA CTAGCGGAGG CTAGAA-GGA GAGAG--ATG GGTGCGAGAG CG
BF1.ES.09.DEMBF09ES003.JX14066   GCCAAAA--- ---------- ---------- ---TTTTTGA CTAGCGGAGG CTAGAA-GGA GAGAG--ATG GGTGCGAGAG CG
BF1.ES.09.DEMBF09ES006.JX14066   GCCAAAA--- ---------- ---------- ---TTTTTGA CTAGCGGAGG CTAGAA-GGA GAGAG--ATG GGTGCGAGAG CG
BF1.IT.01.53143.GU595149         CCCAAAA--- ---------- ---------- ---ATTTTGA CTAGCGGAGG CTAGAA-GGA GAGAG--ATG GGTGCGAGAG CG
BF1.IT.02.30638.GU595148         GCCATAA--- ---------- ---------- ---TTTTTGA CTAGCGGAGG CTAGAA-GGA GAGAG--ATG GGTGCGAGAG CG
BF1.IT.02.57954.GU595150         GCHA------ ---------- ---------- ---ATTTTGA CTAGCGGAGG TCAGAA-GGA GAGAG--ATG GGTGCGAGAG CG
BF1.IT.02.58736.GU595160         GCCAAAA--- ---------- ---------- ---TTTTTTA CTAGCGGAGG CTAGAA-GGA GAGAG--ATG GGTGCGAGAG CG
BF1.IT.02.59211.GU595151         GCCAAAC--- ---------- ---------- ---ATTTTGA CTAGCGGAGG CTAGAA-GGA GAGAG--ATG GGTGCGAGAG CG
BF1.IT.05.83166.GU595152         GCCAAAA--- ---------- ---------- ---ATTTTGA CTAGCGGAGG CTAGAA-GGA GAGAG--ATG GGTGCGAGAG CG
BF1.IT.06.89072.GU595153         GCCAAAA--- ---------- ---------- ---ATTTTGA CTAGCGGAGG CTAGAA-GGA GAGAG--ATG GGTGCGAGAG CG
BF1.JP.04.DR6082.AB480298        GCCAA----- ---------- ---------- ---TTTTTGA CTAGTGGAGG CTAGAA-GGA GAGAG--ATG GGTGCGAGAG CG
BF1.JP.04.DR6190.AB480300        GCCAAT---- ---------- ---------- ---TTTTTGA CTAGCAGAGG CTAGAA-GGA GAGAG--ATG GGTGCGAGAG CG
BF1.JP.x.DR0769.AB253430         GCCA------ ---------- ---------- --TTTTTTGA CTAGCGGAGG CTAGAA-GGA GAGAG--ATG GGTGCGAGAG CG
BFG.MO.05.MO108.GU207884         GCCAA----- ---------- ---------- ---TTTTTGA CTAGCGGAGG CTAGAA-GGA GAGAG--ATG GGTGCGAGAG CG
BG.CU.x.Cu100.AY586546           GCCAAAC--- ---------- ---------- ---TTTTTGA CTAGCGGAGG CTAGAA-GGA GAGAG--ATG GGTGCGAGAG CG
BG.DE.01.9196_01.AY882421        GCCATAA--- ---------- ---------- ---ATTTTGA CTAGCGGAGG CTAGAA-GGA GAGAG--ATG GGTGCGAGAG CG
CD.KE.01.ML1076.EU110086         GCCAA----- ---------- ---------T TTTTATTTGA CTAGCGGAGG CTAGAA-GGA GAGAG--ATG GGTGCGAGAG CG
CU.JP.04.DR5782.AB286849         GCCAAAA--- ---------- ---------- ---TTTTTGA CTAGCGGAGG CTAGAA-GGA GAGAG--ATG GGTGCGAGAG CG
DF1G.ES.04.X963_4.FJ670527       GCTGAAA--- ---------- ---------- ----TTTTGA CTAGCGGAGG CTAGAA-GGA GAGAG--ATG GGTGCGAGAG CG
DO.FR.08.RBF208.GQ351296         ---------W TTTGCTGGCG GTGGCCAGAC CTAGGGGAAG -GGCGAAGTC CCTAGG-GGA GGAAG--ATG GGTGCGAGTG CG
```

Figure S1. Alignment of all HIV-1 group M sequences described in the Los Alamos HIV sequence database (Premade web alignment of complete sequences 2012; www.hiv.lanl.gov) and used in the phylogenetic analysis (Figure 2). The region encompassing the nucleotides involved in the SD, SDa, SD-3WJ and U5-AUG interactions (positions 554-804 in the proviral HXB2 reference sequence) is shown. Sequences with ambiguous nts, nt insertions and deletions and nt variations in the analyzed region that are observed only in a single virus isolate, were excluded from the phylogenetic analysis, as these may represent reverse transcription, PCR or sequencing errors.

© 2016 by the authors. Submitted for possible open access publication under the terms and conditions of the Creative Commons Attribution (CC-BY) license (http://creativecommons.org/licenses/by/4.0/).
